# Bacterial Transcription Factors Bind to Coding Regions and Regulate Internal Cryptic Promoters

**DOI:** 10.1128/mbio.01643-22

**Published:** 2022-10-06

**Authors:** Canfeng Hua, Jiadai Huang, Tingting Wang, Yue Sun, Jingui Liu, Linfeng Huang, Xin Deng

**Affiliations:** a Department of Biomedical Sciences, City University of Hong Kong, Hong Kong, China; b Division of Natural and Applied Sciences, Duke Kunshan University, Kunshan, Jiangsu, China; c Global Health Research Center, Duke Kunshan University, Kunshan, Jiangsu, China; d Shenzhen Research Institute, City University of Hong Kong, Shenzhen, Guangdong, China; e Tung Biomedical Sciences Center, City University of Hong Kong, Hong Kong, China; College of Veterinary Medicine, Cornell University

**Keywords:** ChIP-seq, coding region binding TFs, cryptic promoter, transcriptional regulation

## Abstract

Transcription factors (TFs) regulate transcription by binding to the specific sequences at the promoter region. However, the mechanisms and functions of TFs binding within the coding sequences (CDS) remain largely elusive in prokaryotes. To this end, we collected 409 data sets for bacterial TFs, including 104 chromatin immunoprecipitation sequencing (ChIP-seq) assays and 305 data sets from the systematic evolution of ligands by exponential enrichment (SELEX) in seven model bacteria. Interestingly, these TFs displayed the same binding capabilities for both coding and intergenic regions. Subsequent biochemical and genetic experiments demonstrated that several TFs bound to the coding regions and regulated the transcription of the binding or adjacent genes. Strand-specific RNA sequencing revealed that these CDS-binding TFs regulated the activity of the cryptic promoters, resulting in the altered transcription of the corresponding antisense RNA. TF RhpR hindered the transcriptional elongation of a subgenic transcript within a CDS. A ChIP-seq and Ribo-seq coanalysis revealed that RhpR influenced the translational efficiency of binding genes. Taken together, the present study reveals three regulatory mechanisms of CDS-bound TFs within individual genes, operons, and antisense RNAs, which demonstrate the variability of the regulatory mechanisms of TFs and expand upon the complexity of bacterial transcriptomes.

## INTRODUCTION

The fast development of the chromatin immunoprecipitation sequencing (ChIP-seq) approach has revealed the genome-wide occupation of transcription factors (TFs) (1–4). Previous studies have shown that eukaryotic TFs bind to both promoters and gene bodies. For instance, 104 TFs have 49.1% (554,613) of their binding peaks in gene bodies in maize (5). Similarly, the yeast TF Gcn4 and the mouse TF ATOH1 have half of their binding peaks in exonic or intronic regions (6, 7). The eukaryotic TFs also exhibit similar binding activities for all regions (6).

Bacterial transcriptomic landscapes change in response to dynamic external environments, which are regulated by TFs (8). To reveal the biological functions of bacterial TFs, our previous studies performed ChIP-seq for 20 and 16 TFs in the human pathogen Pseudomonas aeruginosa and the plant pathogen Pseudomonas syringae, respectively (9–13). In addition, ChIP-seq has shown genome-wide TF-binding sites in other model strains, such as Mycobacterium tuberculosis, Vibrio cholerae, Salmonella enterica, and Escherichia coli (14 to 20). However, few studies have focused on the mechanisms of bacterial TFs binding to coding regions.

Generally, TFs regulate transcription by binding to the promoter regions that are located upstream of the transcription start sites (TSSs) (21, 22). The distribution of TSSs is identified using differential RNA-seq (dRNA-seq) and SMRT-Cappable-seq in bacteria (23, 24), suggesting the complexity of the bacterial transcriptome. For example, one-third of all transcripts are initiated within the coding regions in E. coli (25), while 1,288 TSSs are distributed over 630 coding genes in Clostridium difficile (26). It has been proposed that antisense RNAs (aRNAs) are transcribed by internal promoters inside coding regions (27), resulting in the presence of both sense and antisense transcripts within genes. Strand-specific RNA-seq has revealed that the ratio of antisense:sense RNA is variable (0% to 35.8%) among different bacteria (28). For instance, the transcriptomes of E. coli and Staphylococcus aureus include 22% and 1.3% antisense transcripts, respectively (27, 29). The transcription of aRNAs is initiated within coding regions, indicating an underlying association between the aRNAs and CDS-binding TFs in bacteria (27, 30).

The protein-coding regions account for more than 90% of the bacterial genomes, but their regulatory roles in transcription remain mostly unclear. To date, hundreds of ChIP-seq studies have indicated that TF-binding sites (TFBSs) are extensively scattered across the coding areas, suggesting that TFs can regulate the transcription of coding regions (9, 10, 14). To evaluate this hypothesis, we reanalyzed these ChIP-seq data sets and performed experiments to demonstrate that CDS-binding TFs indeed modulate the expression of subgenic transcripts and aRNAs by interacting with cryptic promoters within coding regions. These findings demonstrate the complexity of the bacterial transcriptomes and reveal significant biological functions of CDS-binding TFs.

## RESULTS

### Majority of binding peaks of bacterial TFs are located in CDS *in vivo*.

**FIG 1:**
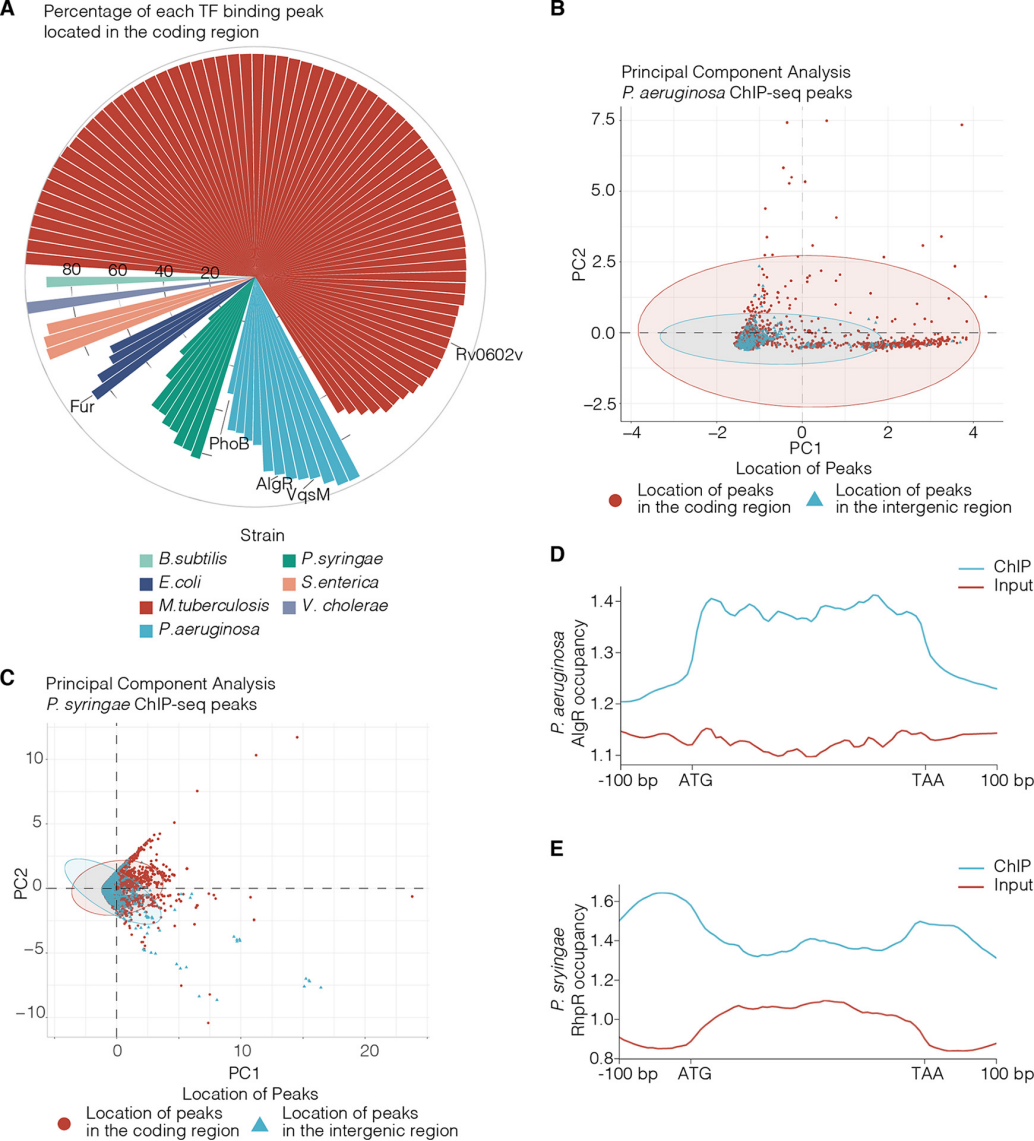
Over 90% of the binding peaks of bacterial TFs are located in CDS *in vivo.* (A) The circular bar plot displays the percentage of each TF’s binding peak located in the coding regions. The different colors indicate different strains. (B and C) Principal components analysis of the peaks generated from the P. aeruginosa and P. syringae ChIP-seq results. (D and E) Visualization of transcriptional regulator (AlgR and RhpR) occupancy around the coding regions. ATG indicates the translation start codon, and TAA indicates the translation terminal codon.

To examine the distribution of TFBSs *in vivo*, we collected 104 ChIP-seq data sets from six model bacterial species: M. tuberculosis H37Rv (14), P. aeruginosa PAO1 (9), S. enterica SL1344 (16, 17), P. syringae 1448A (10), Bacillus subtilis AG174 (31), and E. coli K-12 (18–20). After annotating the binding peaks, we found that the TFs from these strains have 92.6% (19,707) of their binding peaks located in CDS (Fig. 1A and Fig. S1A). Unlike previous studies on the interactions between TFs and promoters, the present study focuses on TFs binding to CDS, which reveals that bacterial TFs frequently bind to gene bodies.

10.1128/mbio.01643-22.1 FIG S1 EMSA validation of AlgR bound to the coding region. (A) Total number of peaks for each TF from seven different strains (detail added to the figure). (B) AlgR bound to the coding region of *tse5*, *pqsA*, and *rocsS2*. In contrast, AlgR did not bound to *dadX*. Download FIG S1, PDF file, 2.0 MB. Copyright © 2022 Hua et al. 2022 Hua et al. https://creativecommons.org/licenses/by/4.0/ This content is distributed under the terms of the Creative Commons Attribution 4.0 International license.

To further elucidate the molecular mechanism process employed by the CDS-binding TFs in bacteria, we used P. aeruginosa and P. syringae as models in the following study. The TF-binding peaks generated by the model-based analysis of ChIP-seq (MACS) have several descriptive characteristics, including the q value and the fold enrichment (32). We compared the intergenic and CDS-localized peaks using a principal components analysis (PCA) to reduce the dimensionality of these characteristics and simultaneously increase their interpretability (33). The PCA results revealed that TFBS found in coding and intergenic regions share similar characteristics in P. aeruginosa and P. syringae (Fig. 1B and C). AlgR is a well-studied TF that regulates the expression of various virulence factors in P. aeruginosa, while RhpR is a repressor of P. syringae virulence (9, 11, 34). To profile the occupation of TFs in coding regions, deepTools was used to visualize the ChIP-seq results of these two crucial TFs (Fig. 1D and E) (35). The AlgR-associated peaks were found to be enriched in coding regions (322 peaks out of 361 peaks) (Fig. 1D). To validate this interaction, electrophoretic mobility shift assays (EMSAs) were performed, and these validated that AlgR bound to the coding regions of *tse5*, *pqsA*, and *rocsS2*, but not to the coding region of the negative-control (*dadX*) (Fig. S1B). The RhpR-binding peaks were more enriched in the untranslated regions than in the coding regions (Fig. 1E). These findings demonstrate that the binding peaks of these two TFs are enriched in the coding regions, indicating the potential initiation of cryptic transcription in the coding regions.

TFs regulate various biological processes by recognizing and interacting with specific DNA sequences (36). We then uncovered the binding sites of AlgR and RhpR, and we determined their consensus motifs using Multiple Em for Motif Elicitation (MEME) (11, 34, 37, 38). Both the find individual motif occurrences (FIMO) scores and q values of these motifs were significantly higher in intergenic regions than in coding regions (Fig. S2A and B). However, peak occupancy analyses performed using deepTools revealed that the peak shape of each TF around the motifs was similar for both regions (Fig. S2C and D) (35).

10.1128/mbio.01643-22.2 FIG S2 Comparison of the motif sites between the coding and intergenic regions. (A and B) Comparison of the FIMO scores and the q values of the peaks around motif sites (AlgR and RhpR) located in the coding and intergenic regions. (C and D) Comparison of the peak occupancy profiling around motif sites (AlgR and RhpR) distributed in the coding and intergenic regions. Download FIG S2, TIF file, 1.1 MB. Copyright © 2022 Hua et al. 2022 Hua et al. https://creativecommons.org/licenses/by/4.0/ This content is distributed under the terms of the Creative Commons Attribution 4.0 International license.

### More than half of bacterial TFBSs are found in CDS *in vitro*.

**FIG 2:**
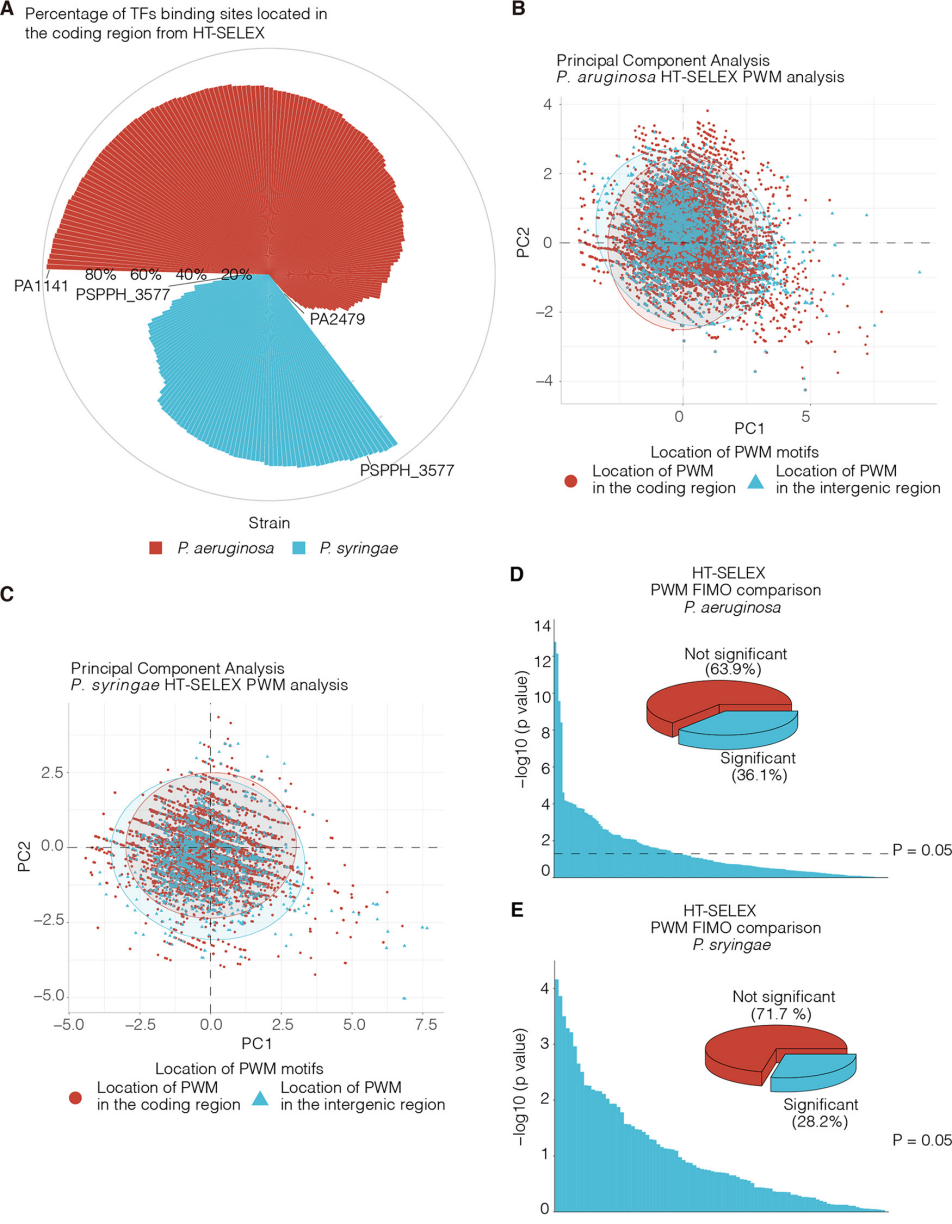
More than half of the bacterial TFBSs are found in CDS *in vitro.* (A) The circular bar plot displays the percentage of each TF’s binding site distributed in the coding region. (B and C) Principal components analysis of the PWM motifs generated from the P. aeruginosa and P. syringae HT-SELEX. (D and E) The bar plot displays the *P*-value generated from the comparison of the FIMO scores in the coding region and intergenic region. The inset pie plot displays the percentage of significant and insignificant TFs. The statistical test used was the Wilcoxon signed-rank test.

Although ChIP-seq allows for the detection of DNA-protein interactions at a genome-wide scale *in vivo*, the DNA-binding specificity of TFs can be influenced by protein-protein interactions, which are avoided in the systematic evolution of ligands by exponential enrichment (SELEX) approaches (39). To date, only three studies have decoded the binding specificities of bacterial TFs using SELEX (40–42). To determine the binding profile of each TF, we scanned and mapped the position weight matrix (PWM) generated from a high-throughput SELEX of 281 TFs in P. aeruginosa and P. syringae to their respective genomes (40, 41). Surprisingly, in both strains, 81.0% (228) of the TFs had more than half of their binding sites located in CDS (Fig. 2A), regardless of the total number of TF peaks (Fig. S3A). For example, 98.2% (896) and 91.4% (1,081) of PA1141- and PSPPH_3577-binding sites were located in CDS, respectively. In contrast, several TFs had less than 20% of their binding sites located in CDS. For example, PA2479 and PSPPH_2432 had 5.3% (2) and 15% (2) CDS-localized peaks, respectively (Fig. 2A). In addition, we reanalyzed the SELEX data for E. coli, which showed that more than 80% of the TFBSs were localized to CDS (Fig. S3B and C) (42).

10.1128/mbio.01643-22.3 FIG S3 In total, there were 12,293 predicted TFBS from the SELEX data from P. aeruginosa, P. syringae, and E. coli. (A) The total binding number for each TF from the HT-SELEX data from P. aeruginosa and P. syringae. (B) Percentage of the TFs’ binding sites located in the coding region, based on genomic-SELEX. (C) The total binding number for each TF, based on E. coli genomic-SELEX data. Download FIG S3, PDF file, 0.7 MB. Copyright © 2022 Hua et al. 2022 Hua et al. https://creativecommons.org/licenses/by/4.0/ This content is distributed under the terms of the Creative Commons Attribution 4.0 International license.

The TF-binding motif was generated from the PWM to represent the likelihood of each base in a motif (43). The binding sites of individual TFs were identified using FIMO-generated genomic PWMs (44). PCA was used to compare binding-site features, such as FIMO scores and *q* values, across the coding and intergenic regions (Fig. 2B and C) (33). Interestingly, the TFs showed similar binding preferences across the coding and intergenic regions. To explore the similarity of the TFBSs between these two regions, FIMO scores were compared for each TF in P. aeruginosa and P. syringae (Data Set S1 and S2) (44). We found that 63.9% (192) and 71.7% (71) of the TFBSs were highly similar between the coding and intergenic regions in these two strains (Fig. 2D and E). Taken together, the results indicate that the TFBSs located in coding regions share similar characteristics with those found in intergenic regions, suggesting that the CDS-binding TFs have potential biological functions.

10.1128/mbio.01643-22.7 DATA SET S1 Comparing the FIMO score of TFs between the intergenic region and the coding region in P. aeruginosa. Download Data Set S1, PDF file, 0.9 MB. Copyright © 2022 Hua et al. 2022 Hua et al. https://creativecommons.org/licenses/by/4.0/ This content is distributed under the terms of the Creative Commons Attribution 4.0 International license.

10.1128/mbio.01643-22.8 DATA SET S2 Comparing the FIMO score of TFs between the intergenic region and the coding region in P. syringae. Download Data Set S2, PDF file, 0.5 MB. Copyright © 2022 Hua et al. 2022 Hua et al. https://creativecommons.org/licenses/by/4.0/ This content is distributed under the terms of the Creative Commons Attribution 4.0 International license.

### CDS-bound TFs regulate the expression of bound and surrounding genes.

**FIG 3:**
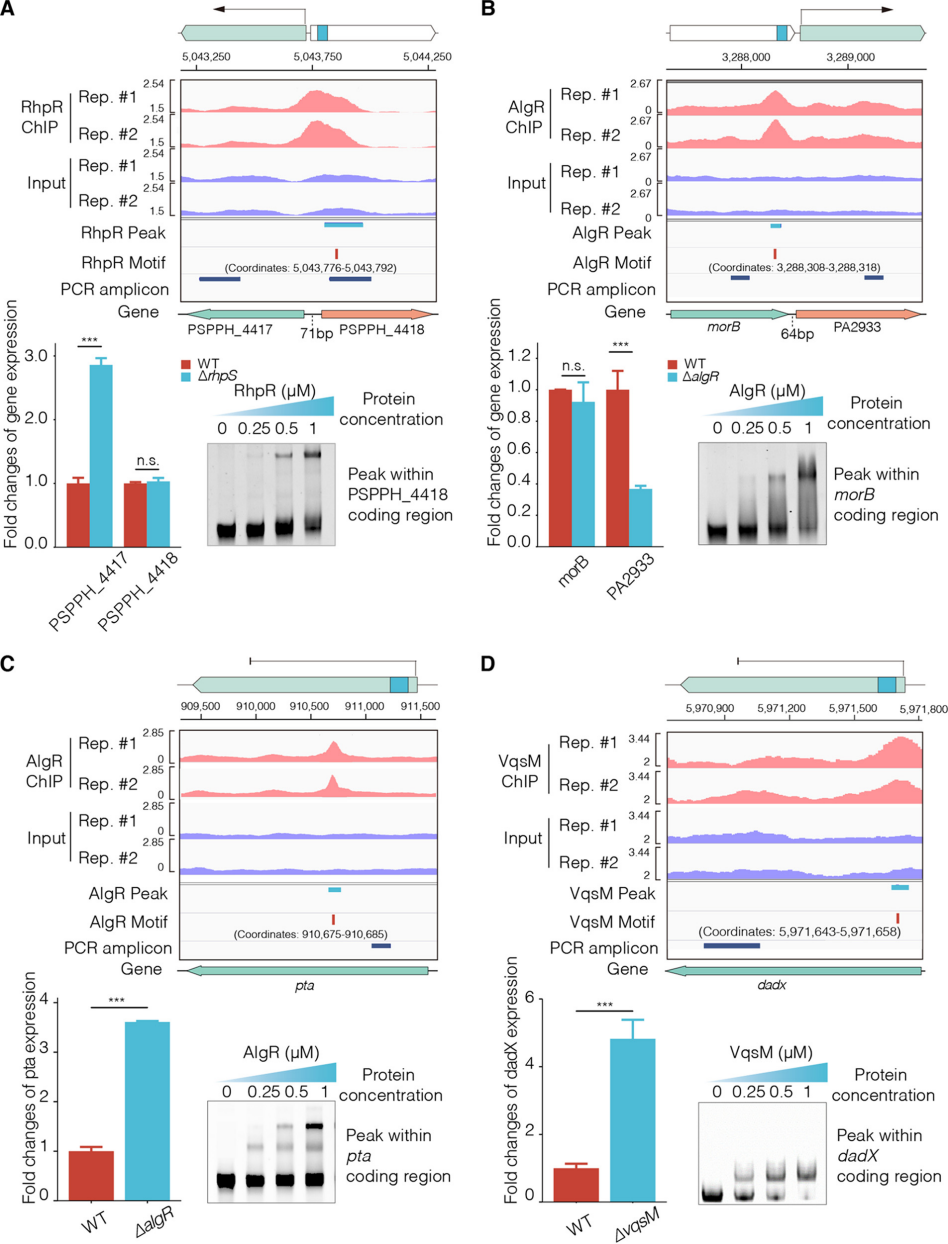
CDS-bound TFs regulate the expression of bound and surrounding genes. The TFs’ occupancies from the ChIP-seq IP and input groups were visualized for two replicates. The peak and motif sites were obtained by using MACS and MEME, respectively. The lane entitled PCR amplicon shows the RT-qPCR amplification fragments. (A and B) The CDS-binding TFs (RhpR in P. syringae and AlgR in P. aeruginosa) activated the adjacent genes. (C and D) The CDS-binding TFs (AlgR and VqsM in P. aeruginosa) repressed the transcription of the CDS. The *t* test is the Student’s *t* test with *, *P* < 0.05; **, *P* < 0.01; and ***, *P* < 0.001.

To explore the association between the TFs and CDS-localized binding peaks, we used the integrative genomics viewer (IGV) to visualize the binding peaks of RhpR, AlgR, and VqsM from their ChIP-seq data (Fig. 3A–D, showing the ChIP-seq immunoprecipitation and input samples) (9, 11, 45). The peaks and locations are shown in the lanes labeled with “RhpR or AlgR peak”, and the TF motifs were generated by MACS and MEME (Fig. 3A–D). We performed RT-qPCR to quantify the transcript levels of the genes adjacent to or containing the binding peaks (Fig. 3A–D). The “DNA fragment” lane shows the location of the real-time quantitative polymerase chain reaction (RT-qPCR) products (Fig. 3A–D). The auto-inhibitor RhpR and the histidine kinase RhpS belong to a crucial two-component system that regulates P. syringae virulence and metabolism. The deletion of *rhpS* (Δ*rhpS*) leads to a 10-fold increase in *rhpR* expression compared to that observed in the wild-type (WT) strain (11, 21, 38, 46). In the present study, RhpR bound to the coding region of PSPPH_4418, resulting in a higher transcriptional level of its flanking gene (PSPPH_4417) in the Δ*rhpS* strain compared to the WT strain (Fig. 3A; Fig. S4A). Similarly, AlgR bound to the coding region of *morB*, which led to a lower transcriptional level of the flanking gene (PA2933) in Δ*algR* than in the WT strain (Fig. 3B). These results suggest that these CDS-binding TFs promote the expression of genes next to the binding sites.

10.1128/mbio.01643-22.4 FIG S4 AlgR influenced the expression of PA0324 by binding to the PA0323-6 operon. (A) Negative-control EMSA result for RhpR. (B) Negative-control EMSA results for VqsM. (C) AlgR bound to the coding region of PA0324 and PA0325, and the transcription level of PA0324 was higher in the Δ*algR* strain than in the WT strain. Download FIG S4, TIF file, 1.2 MB. Copyright © 2022 Hua et al. 2022 Hua et al. https://creativecommons.org/licenses/by/4.0/ This content is distributed under the terms of the Creative Commons Attribution 4.0 International license.

To further verify whether AlgR regulates these targets in CDS, DNA fragments carrying the ChIP-seq binding peaks were tested using EMSA (Fig. 3C). We confirmed that AlgR interacts with the coding region of *pta*, which encodes phosphate acetyltransferase. Furthermore, the mRNA level of *pta* was 3-fold higher in the Δ*algR* strain than in the WT strain (Fig. 3C). VqsM bound to the coding region of *dadX* (encoding catabolic alanine racemase), and its expression was higher in the Δ*vqsM* strain than in the WT strain (Fig. 3D; Fig. S4B). These results indicated that CDS-binding TFs can activate the expression of the flanking genes while also repressing the expression of the bound genes.

### Bacterial operons are regulated by CDS-binding TFs.

**FIG 4:**
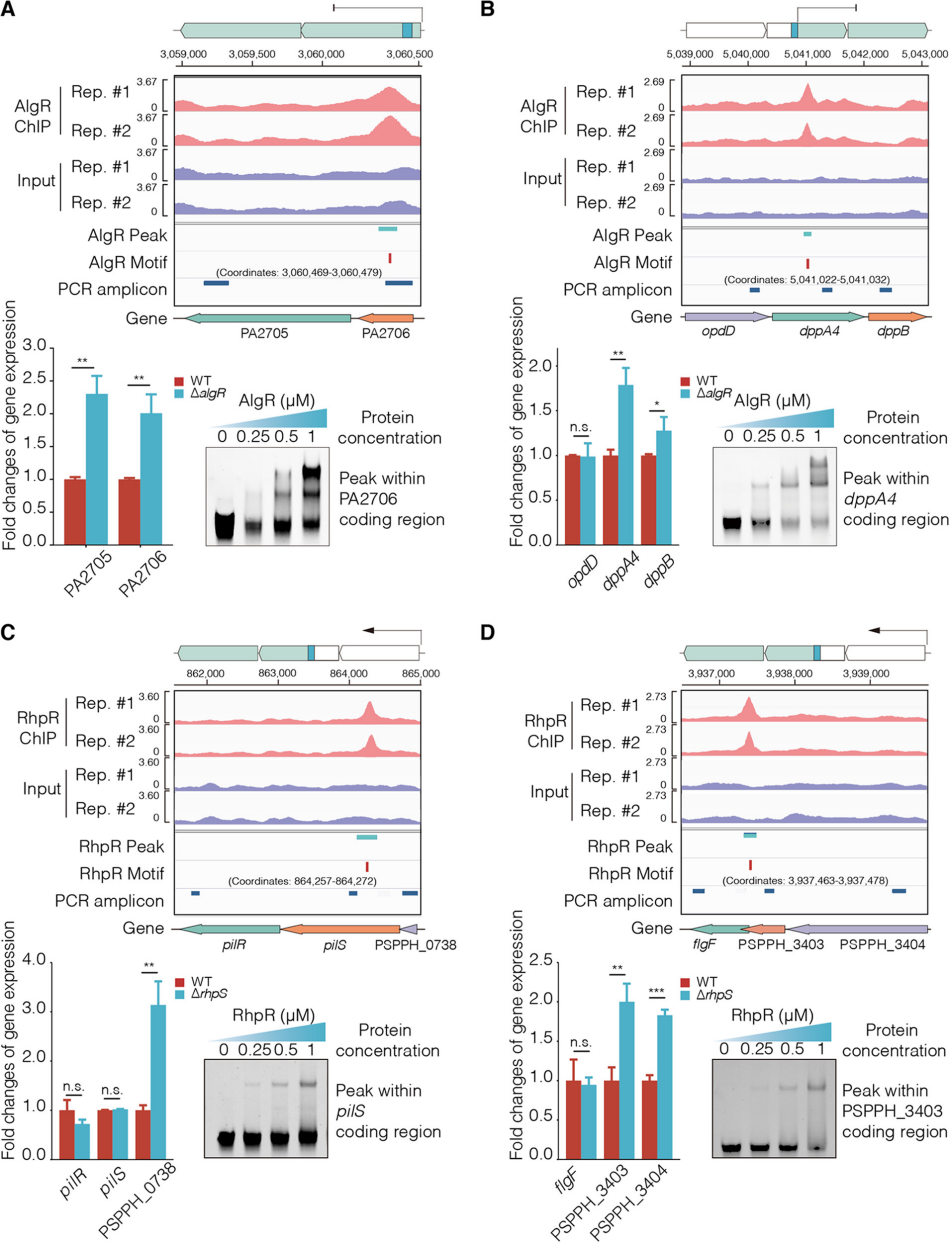
Bacterial operons are regulated by CDS-binding TFs. The TFs occupancies from the ChIP-seq IP and input groups were visualized for two replicates. The peak and motif sites were obtained by using MACS and MEME, respectively. The lane entitled PCR amplicon shows the RT-qPCR amplification fragments. (A) AlgR repressed the transcription of the operon via binding to the first structured gene. (B) AlgR inhibited the transcription of different operon genes in the *dppA4* and *dppB* via binding to the coding region of *dppA4*. (C) RhpR blocked the transcription of *pilR* and *pilS* in the PSPPH_0738_*pilRS* operon via binding to *pilS*. (D) RhpR blocked the transcription of *flgF* in the PSPPH_3403-4_*flgF* operon via binding to the PSPPH_3403. The *t* test is the Student’s *t* test with *, *P* < 0.05; **, *P* < 0.01; and ***, *P* < 0.001.

In prokaryotes, an operon is a cluster of genes under the control of a shared promoter upstream of its first gene. In the PA2705-2706 operon, AlgR bound to the coding region of the first gene (PA2706). Notably, the expression level of the entire operon was higher in the Δ*algR* strain than in the WT strain (Fig. 4A), suggesting that CDS-bound TFs can inhibit the transcription of all genes in an operon.

In the *opdD*-*dppA4B* operon, AlgR bound to the coding region of *dppA4*, resulting in higher transcriptional levels of *dppA4* and *dppB* in the Δ*algR* strain than in the WT strain (Fig. 4B). In the PA0323-0326 operon, AlgR interacted with the coding region of PA0324 and PA0325, which led to higher expression of PA0324 in the Δ*algR* strain than in the WT strain (Fig. S4C). These findings demonstrated that the CDS-binding of AlgR altered the transcriptional levels of different genes within the operons. In addition, RhpR bound to the coding region of *pilS* in the PSPPH_0738*-pilSR* operon. The mRNA level of the first gene in the operon (PSPPH_0738) was higher in the Δ*rhpS* strain than in the WT strain, but the transcription of the two downstream genes did not change (Fig. 4C). In the *flgF-*PSPPH_3403-PSPPH_3404 operon, RhpR bound to the coding region of PSPPH_3403 (Fig. 4D). As a result, the expression levels of PSPPH_3403 and PSPPH_3404 were higher in the Δ*rhpS* strain than in the WT strain, while the mRNA level of *flgF* remained the same for both strains (Fig. 4D). Taken together, these results showed that TFs differently regulate the transcription of genes within an operon by binding to their CDS.

### CDS-bound TFs activate aRNA transcription via cryptic promoters.

**FIG 5:**
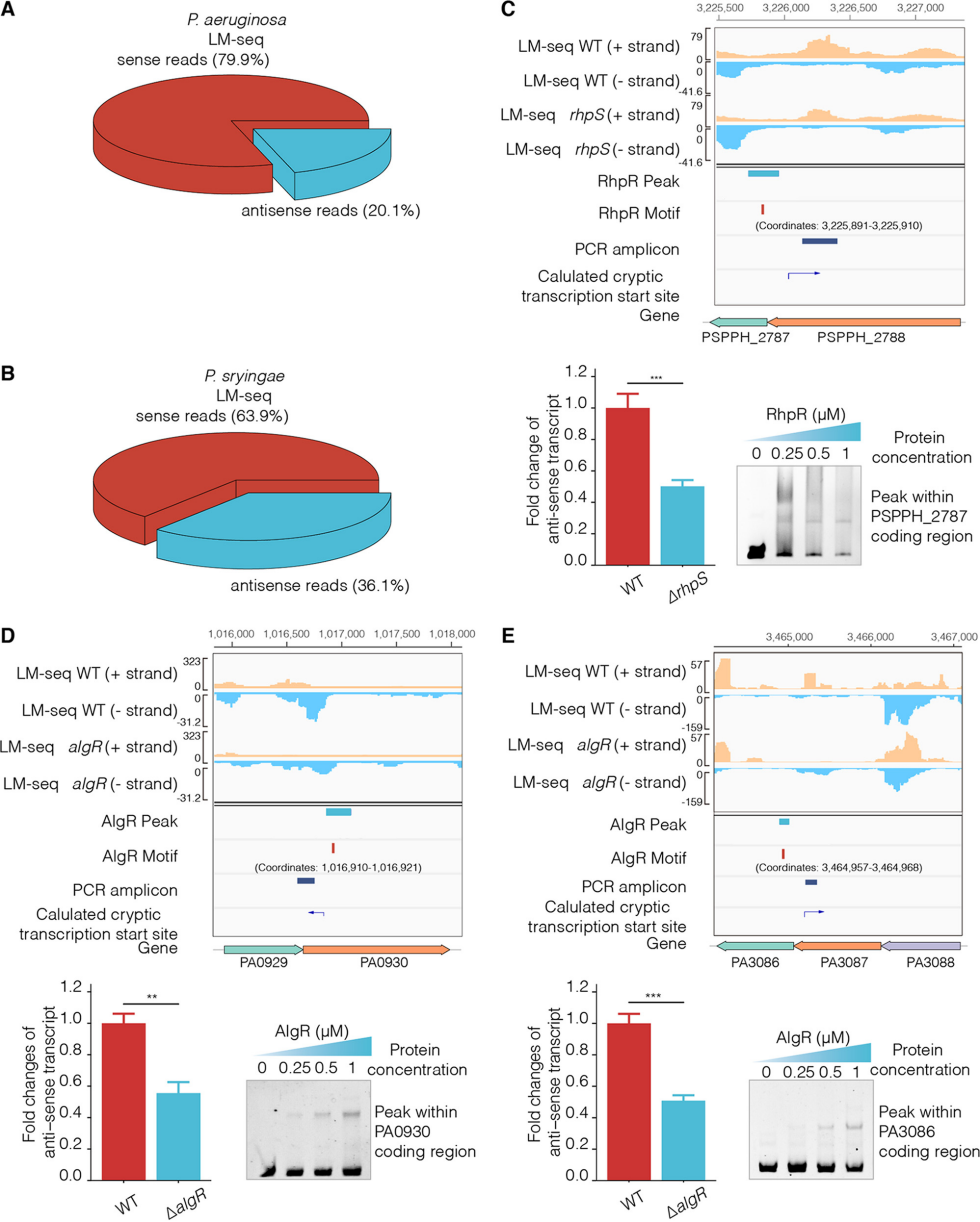
CDS-bound TFs activate aRNA transcription by regulating cryptic promoters. (A and B) The pie plot showed the percentage of sense and antisense reads from the P. aeruginosa and P. syringae WT strains generated by LM-seq. (C to E) The Watson strand (+ strand) reads and Crick strand (− strand) reads from the WT and mutant strains (*rhpS* and *algR*) were generated by LM-seq. The peak and motif sites were shown in tracks 7 and 8, and the peak fragments were validated by EMSA. The lane entitled “PCR amplicon” shows the RT-qPCR amplification fragments. Additionally, IGV also visualized the calculated cryptic transcription start sites and direction. (C) RhpR repressed the antisense RNA expression by repressing the cryptic promoter within the PSPPH_2788. (D and E) AlgR activated the antisense RNA expression by binding to the PA0930 and PA3086. The *t* test is the Student’s *t* test with *, *P* < 0.05; **, *P* < 0.01; and ***, *P* < 0.001.

Stranded-RNA sequencing approaches have revealed the widespread presence of aRNAs in many bacterial species (47), including E. coli, C. difficile, and S. aureus (27, 29). To identify the antisense transcripts in P. aeruginosa and P. syringae, strand-specific ligation mediated RNA sequencing (LM-seq) was performed. We found that 20.1% and 36.1% of all reads were mapped to antisense transcripts in P. aeruginosa and P. syringae, respectively (Fig. 5A and B).

To validate the aRNA transcription, Rockhopper was used to determine the cryptic transcription start sites (TSSs) (48). Interestingly, the TFBSs were located next to the TSSs in coding regions, which led us to propose that TFs play an important role in regulating transcription via activating cryptic promoters in CDS. To further investigate the mechanisms of the TF-mediated regulation of aRNA transcription, we performed LM-seq in Δ*rhpS*, Δ*algR*, and WT strains (Fig. 5C–E). IGV was used to visualize the density of reads in the Watson (+) and Crick (−) strands. In the CDS, cryptic promoters initiate transcription on the forward or reverse DNA strands (49). TFBSs located near these cryptic TSSs were validated using EMSA. The transcription levels of the aRNAs were tested using strand-specific RT-qPCR, which synthesized complementary DNA (cDNA) using a downstream reverse primer. RhpR bound to the coding region of PSPPH_2788 and activated the transcription of an antisense transcript (Fig. 5C). In contrast, AlgR interacted with the coding regions of PA0930 and PA3086. The transcription levels of two antisense transcripts (overlapping with PA0930 and PA3087) were lower in the Δ*algR* strain than in the WT strain (Fig. 5D and E). Overall, bacterial TFs were found to control antisense transcription initiation by binding and regulating cryptic promoters in CDS.

The ChIP-qPCR results showed that RNAP occupancy was elevated by AlgR as a positive regulator, whereas it was inhibited by AlgR as a negative regulator (Fig. S4E). In the Δ*algR* strain, the RNAP occupancy inside PA0930 was lower than that observed in the WT, which correlated with the expression level of the corresponding aRNA in both strains (Fig. 5D). The RNAP occupancy was reduced inside PA2706 and in the *algD* promoter in the Δ*algR* strain, compared to the WT, suggesting that the co-occupancy feature of RNAP with TFs is similar in both the promoter regions and the CDS. We also had the same observation for RhpR. When serving as a positive regulator, RhpR recruited RNAP to trigger the expression of downstream genes from the binding site within *pilS* (Fig. 4C). While serving as a negative regulator, RhpR inhibited RNAP-binding to reduce the expression of downstream genes via the binding sites within PSPPH 2787 and the *hrpR* promoter. Taken together, our results showed that TFs modulate gene expression by regulating RNAP-binding in both promoter and coding regions.

### RhpR negatively regulated the expression of a subgenic transcript within the CDS of PSPPH_3675 by inhibiting transcriptional elongation.

**FIG 6:**
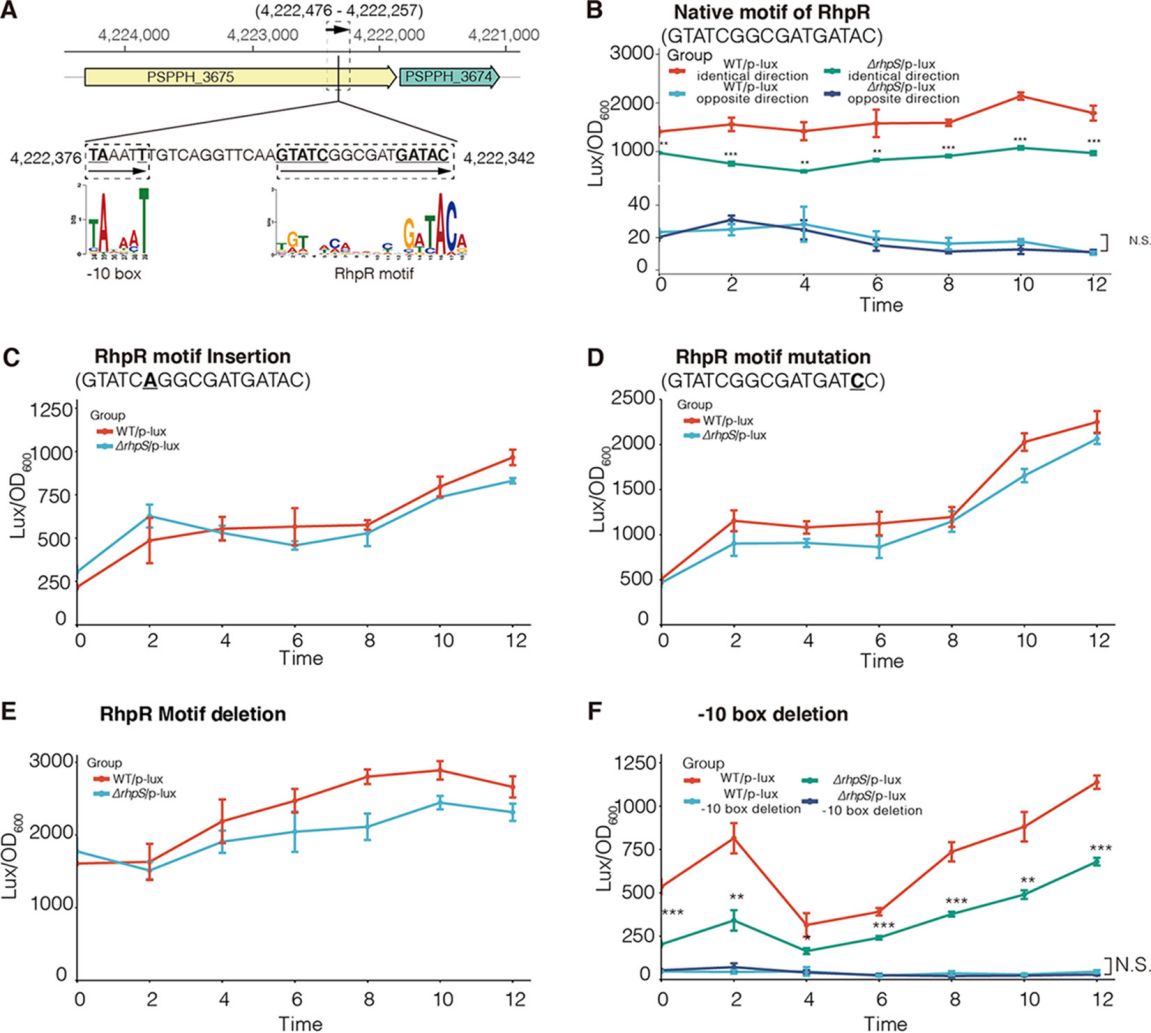
RhpR obstructed the transcriptional elongation of a subgenic transcript within the CDS of PSPPH_3675. (A) The position of the PSPPH_3674 and PSPPH_3675 in the P. syringae genome. The locations of the RhpR motif and the −10 box are labeled in the diagram, and the conserved sites are highlighted in bold and underlined. The direction of the subgenic transcript is indicated with an arrow, and the position of the reporter is showed in the dashed box. (B) Lux activity of the subgenic transcript, in which the RhpR motif was identical to the sequence within the PSPPH_3675. The reporter with the same transcriptional direction as PSPPH_3675 was labeled as the identical direction. The reporter whose transcriptional direction was opposite to that of PSPPH_3675 was labeled as the opposing direction. (C) Lux activity of the subgenic transcript. An “A” was inserted into the RhpR motif. (D) Lux activity of the subgenic transcript. In the RhpR motif, an “A” was replaced by a “C”. (E) Lux activity of the subgenic transcript. The RhpR motif was deleted in the reporter. (F) Lux activity of the subgenic transcript. The −10 box was deleted in the reporter.

Following a genome-wide search, we identified a conserved motif of RhpR located in the coding region of PSPPH_3675 (Fig. 6A) (41). Since the transcriptional direction of the subgenic transcript cannot be retrieved directly from the ChIP-seq and RNA-seq data, two plasmids with opposing orientations were generated to test their corresponding transcripts. The reporter plasmids were centered on the RhpR motif and extended by 100 bp on both sides (Fig. 6A). An extra conserved ribosome binding site (AGGAGG) was inserted at the 3′ end of the fragments to promote translation by preventing rho-mediated transcription termination and stabilizing the RNA (50). According to the results, RhpR negatively regulated a subgenic transcript with the same transcription direction as that of PSPPH_3675 (Fig. 6B). We did not detect a strong signal from the reporter whose transcription direction was opposite to that of PSPPH_3675 (Fig. 6B). To further investigate the biological role of the RhpR with respect to this subgenic transcript, we inserted, mutated, and deleted the RhpR motif in the reporters (Fig. 6C to E). As expected, no significant differences were observed in these various motif reporters, indicating that RhpR regulated this subgenic transcript by binding to the coding region of PSPPH_3675. At the 5′ end upstream of the RhpR motif, a conserved −10 box was identified (Fig. 6A). To better study the mechanism, a reporter without this −10 box was made such that it showed a reduced signal compared to that of the original reporter (Fig. 6F). In conclusion, RhpR negatively regulated the expression of a subgenic transcript within the CDS of PSPPH_3675 by impeding the transcription elongation.

To determine whether RhpR-binding affects the transcription of PSPPH_3675, two more reporters were constructed. In the WT strain and the Δ*rhpS* strain, both reporters were derived from the promoter region of PSPPH 3675, with the 3′ end positioned either before or after the RhpR motif (Fig. S5A). The results demonstrated that RhpR had no effect on PSPPH_3675 transcription (Fig. S5B). We next investigated whether this subgenic transcript is an independent transcript that does not share its sequence with other transcripts. Two primers were designed, one of which was placed upstream of the RhpR motif, whereas the other was located at the coding region of PSPPH_3674 (Fig. S5A). Through PCR using both genome DNA and cDNA as the templates, the products had identical length, suggesting that the subgenic transcript is not an independent transcript (Fig. S5C).

10.1128/mbio.01643-22.5 FIG S5 The subgenic transcript overlapped with PSPPH_3675, and RhpR had no effect on PSPPH_3675 expression. (A) The diagram shows the position of the PSPPH_3674 and the PSPPH_3675 in the P. syringae genome. The position and sequence of the RhpR motif was labeled in the diagram. The arrows indicate different reporters. The locations of different primers are also shown in the diagram. (B) Lux activities of different reporters located before or after the RhpR motif in the WT strain and the Δ*rhpS* strain. (C) Agarose gel showed PCR products from the genome and cDNA. D. The EMSA results were obtained using 1 μg DIDC in each reaction. (E) The ChIP-qPCR results of RNAP occupancy at the AlgR binding sites. (F) The ChIP-qPCR results of RNAP occupancy at the RhpR binding sites. Download FIG S5, TIF file, 0.5 MB. Copyright © 2022 Hua et al. 2022 Hua et al. https://creativecommons.org/licenses/by/4.0/ This content is distributed under the terms of the Creative Commons Attribution 4.0 International license.

### RhpR affected translational efficiency after binding to coding regions.

**FIG 7:**
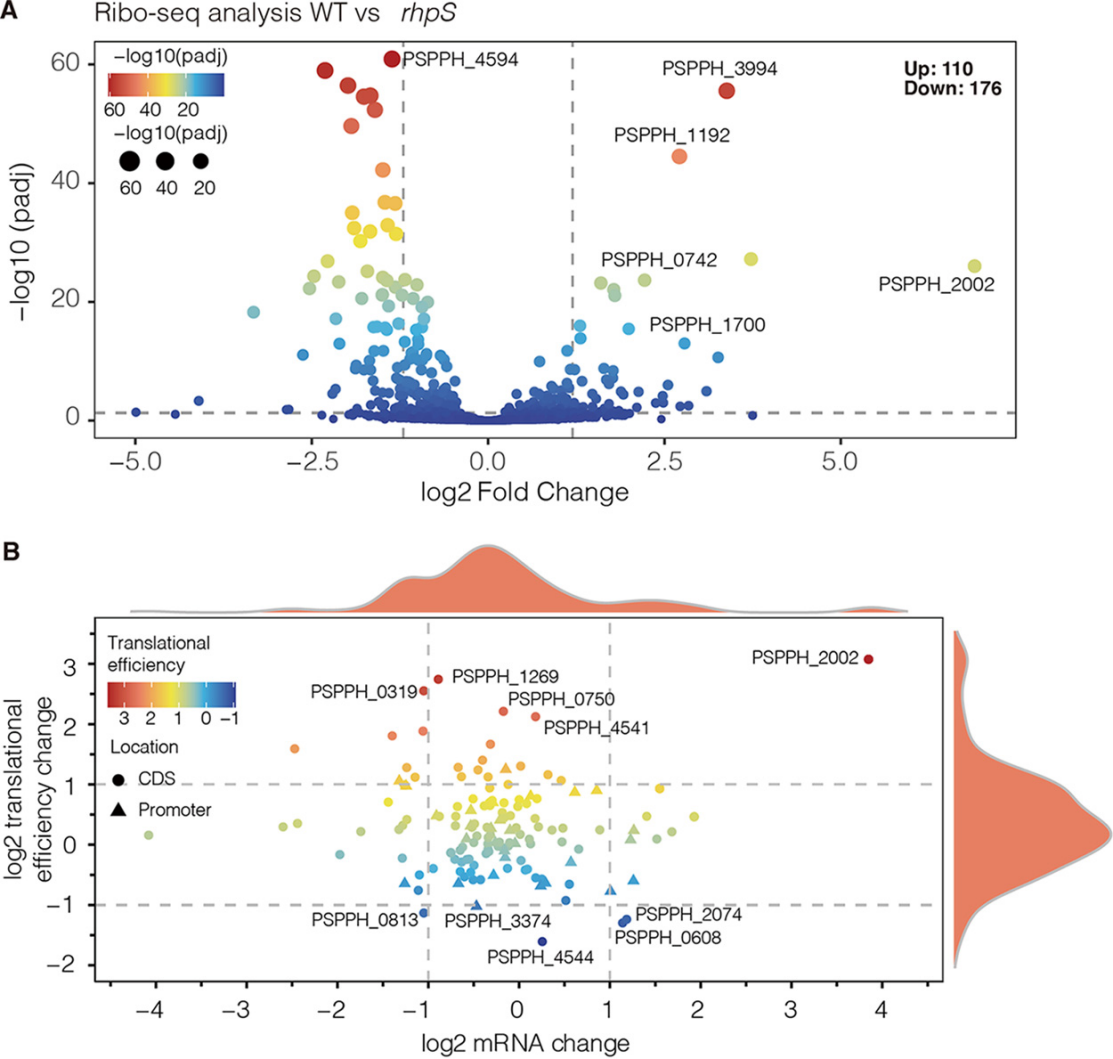
RhpR influenced the translation efficiency of mRNA after binding. (A) Volcano plot of Ribo-seq results. The *x* axis shows the log_2_ (fold change) of the Ribo-seq results. The *y* axis shows −log_10_ adjusted *P*-values. The different colors and sizes indicate different −log_10_ adjusted *P* values. (B) The scatterplot shows the translational efficiency and mRNA change, which had RhpR binding peaks located at its promoter or coding regions. The *x* axis shows the log_2_ (fold change) of the mRNA level, and the *y* axis shows the log_2_ (fold change) of the translational efficiency. The different colors indicated different translational efficiencies. The greater TE was represented in red, whereas the lesser TE was displayed in blue. Triangles represent genes with RhpR-binding sites in their promoter regions. The circles represent genes with RhpR-binding sites in the coding areas.

To further examine the biological roles of CDS-binding TFs, ribosome profiling, also known as Ribo-seq, was performed in the P. syringae WT strain and the Δ*rhpS* strain (51). Comparing the WT strain with the Δ*rhpS* strain, the translation efficiencies (TE) of 286 genes were altered significantly (Fig. 7A). The TE of 176 genes, including the elongation factor PSPPH 4594, was downregulated. The TEs of 110 genes, including alcohol dehydrogenase PSPPH_3994 and calcium-binding protein PSPPH_2002, were upregulated. These results indicated that RhpR influenced the efficiency of mRNA transcription.

To investigate the influence of RhpR-binding on genome-wide transcription, we combined the Ribo-seq and ChIP-seq analyses. Genes were classified into two categories based on the locations of their RhpR binding sites (CDS or promoter). RhpR impacted the TEs of the CDS binding genes, such as PSPPH_1269, which encodes lytic murein transglycosylase (Fig. 7B). RhpR-binding also improved the TEs of CDS-binding genes, such as PSPPH_4544, which encodes pancortin. Interestingly, in the promoter-binding group, the majority of the genes displayed no difference in translational efficiency. These results provided strong evidence that RhpR impacted the translational efficiency of some mRNAs by binding to the coding regions.

## DISCUSSION

The findings of the present study provide a systematic view of binding loci for bacterial TFs, which demonstrates that bacterial CDS-binding TFs influence transcription. Important functions of CDS-binding TFs have been reported in eukaryotes (6, 50). For example, Gcn4 is one of the CDS-binding TFs in yeast, and it shares similar mechanisms with the bacterial TFs discussed in this study. Gcn4 and RhpR regulate the transcription of bound or adjacent genes (6). By regulating the activity of cryptic promoters, both TFs regulate the transcription of antisense transcripts. However, Gcn4 activates internal bidirectional transcription (6), which we did not observe in the bacteria.

The present study shows that bacterial CDS-binding TFs regulate the transcription of antisense RNA by regulating cryptic promoters, suggesting an understudied function of bacterial TFs. The abundance of cryptic transcripts has been underestimated in both eukaryotes and prokaryotes. In yeast, TFs interact with CDS to activate cryptic promoters, resulting in unannotated cryptic transcripts, most of which are unstable (1). The transcriptional termination of these unstable RNAs is mediated by the RNA-binding proteins Nrd1 and Nab3 (52). In bacteria, transcriptional termination occurs via both rho-dependent and rho-independent pathways, the former of which halts antisense transcription (53). Given that rho-terminated RNA is typically unstable, the importance of aRNAs is underestimated in bacteria (54).

We also found that some TFs did not regulate the genes in or near their CDS-binding sites. We reason that transcription-related factors also influence cryptic promoter activity. For example, the transcription elongation factor Spt6 represses transcription initiation within the coding regions in S. cerevisiae DNA (55). A previous study identified 55 transcription-related factors capable of repressing cryptic transcription (2). Most of these transcription-related factors are not TFs; rather, they are histones, histone deacetylation proteins, or DNA replication factors (2). The yeast TF Gcn4 was also found to regulate the activity of cryptic transcriptional promoters (6). Given these findings, we propose that many other transcription-related factors, such as NusA and DksA (56), may play important roles in regulating cryptic promoter activity in bacteria.

TFs do not always affect the transcription of the CDS-binding genes, and this can be explained by the following potential reasons. First, TFs and RNAP complexes compete for access to the same DNA. Since the RNAP complex is so vast, and since many transcriptions occur simultaneously, the coding region of the DNA is constantly in an untwisted state, which inhibits TF-binding. Second, this may be the result of heterogeneity (57). In a vast population, only a small proportion of strains express subgenic transcripts, resulting in an extremely weak signal. These genes may have crucial roles under certain conditions (57).

**FIG 8:**
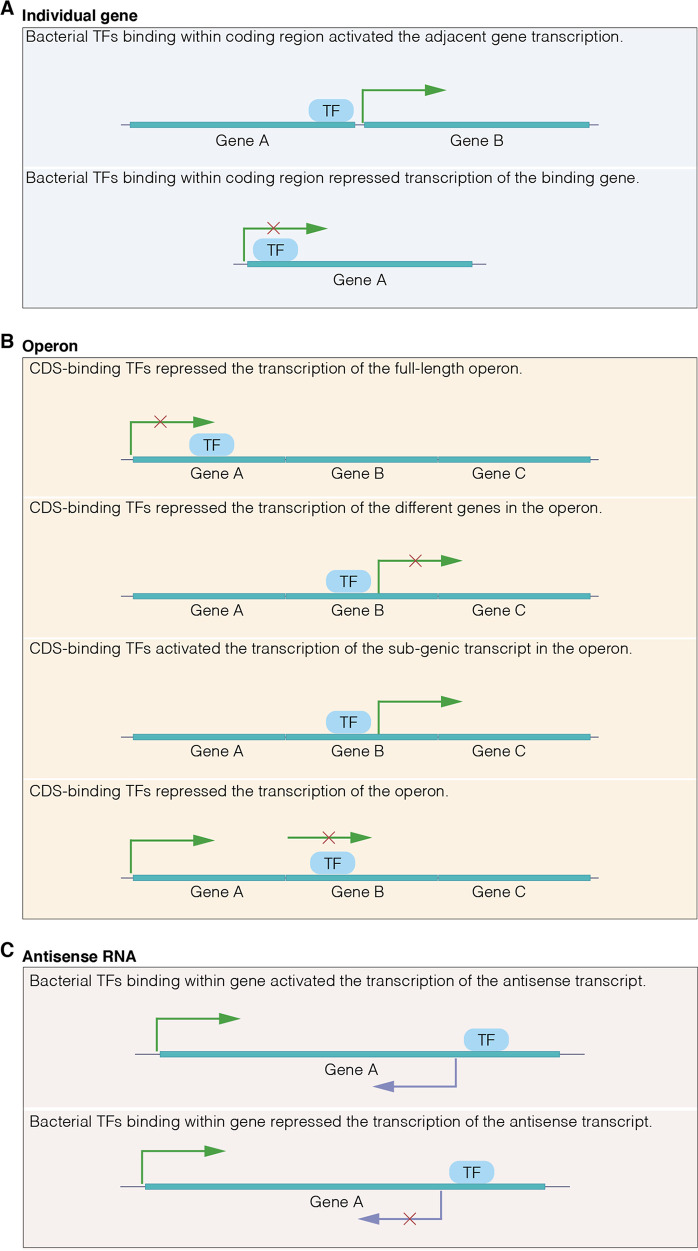
Proposed regulation models of bacterial CDS-binding TFs. (A) The CDS-binding TFs activated the adjacent genes and repressed the binding gene transcription. (B) Binding within the coding region, bacterial TFs regulated the transcription of the full-length or different genes in the operon. Additionally, the CDS-binding TFs repressed the transcription of the operon. (C) Binding to the coding region, bacterial TFs regulated the expression of antisense RNA by controlling the activity of the cryptic promoter.

Taken together, this work revealed three important regulatory mechanisms of CDS-bound TFs within individual genes, operons, or aRNAs. (i) The transcription of a target gene is induced by TFs (e.g., RhpR) by binding within the coding region of an adjacent gene (PSPPH_4418) (Fig. 8A). CDS-bound TFs (e.g., AlgR) can downregulate the transcription of the bound gene (*pta*) (Fig. 8A). (ii) The CDS-bound TFs (e.g., AlgR) can reduce the transcription of either a whole operon (PA2705-PA2706) or sub-transcripts (*dppA4* and *dppB*) (Fig. 8B). We propose that CDS-bound TFs block RNA polymerase movement and thereby reduce the transcription of individual genes or operons. Moreover, CDS-binding TFs can also activate subgenic transcripts (PSPPH_3675). (iii) CDS-bound TFs (e.g., RhpR) regulate the expression of aRNA by repressing cryptic promoter activity (Fig. 8C). We propose that CDS-bound TFs recruit or inhibit RNA polymerase to activate or inhibit the transcription of the adjacent gene or aRNA. This work demonstrates the variability of the transcriptional regulation mechanisms of CDS-bound TFs and expands upon the complexity of bacterial transcriptomes. Further identification and characterization of these CDS-bound TFs and their downstream gene targets will help elucidate their biological functions, which can be further extended to all prokaryotic TFs.

## MATERIALS AND METHODS

### Reanalyses of ChIP-seq and SELEX results.

We downloaded public fastq data (AlgR ChIP, VqsM ChIP, and P. aeruginosa input, as well as RhpR ChIP and P. syringae input) from GEO and then mapped them to the P. aeruginosa (NC_002516) and P. syringae (NC_005773.3) genomes using Bowtie2, respectively (58). The uniquely mapped reads were applied to the subsequent analyses. The motifs were identified via MEME, and the peaks were identified via MACS (32). We also download the MACS-generated peak files from the public data set (9, 10, 14, 15, 17–19, 31). The subsequence motif was identified by MEME. The SELEX PWM motifs data were downloaded from a public database (40–42).

BEDTools was used to distribute the peaks and motifs into the coding and intergenic regions (59). The peak occupation profiling was visualized and normalized using deepTools (35). The PCA analysis was performed using the factoextra package in R. The comparison and visualization of peak the enrichment and the FIMO scores were made using the tidyverse package in R. The IGV was used to visualize the density of the reads (60).

### Real-time quantitative PCR (RT-qPCR).

The primer sequence can be found in Table S1. The bacteria were pelleted when the OD_600_ reached 0.6 (Table S1), and the total RNA was extracted using a total RNA isolation kit (Sangon Biotech). The RNA concentration was measured using a Nanodrop 2000 spectrophotometer (Thermo Fisher) before cDNA synthesis using a FastKing RT Kit (Tiangen Biotech). RT-qPCR was performed using a SuperReal Premix Plus (SYBR green) Kit (Tiangen Biotech). We calculated the relative fold changes using 2^−(ΔΔCt)^, with 16S rRNA as the reference. All the reactions were conducted with three biological repeats.

10.1128/mbio.01643-22.6 TABLE S1 Strains, plasmids and primers used in this study. Download Table S1, DOCX file, 0.03 MB. Copyright © 2022 Hua et al. 2022 Hua et al. https://creativecommons.org/licenses/by/4.0/ This content is distributed under the terms of the Creative Commons Attribution 4.0 International license.

For the antisense RNA RT-qPCR, the specific forward primers were designed to do reverse transcription using a FastKing RT Kit (Tiangen Biotech). Coupling with the corresponding reverse primer, RT-qPCR was performed using the SuperReal Premix Plus (SYBR green) Kit (Tiangen Biotech). We calculated the relative fold changes using 2^−(ΔΔCt)^ with 16S rRNA as the reference. All of the reactions were conducted in three biological repeats.

### Electrophoretic mobility shift assay (EMSA).

The primer sequence can be found in Table S1. The EMSA was performed with 1 μg DIDC-DIDC, and the primer was modified by FITC. The EMSA binding reaction took place in EMSA binding buffer (10 mM Tris-HCl [pH 7.4] 50 mM KCl, 5 mM MgCl_2_, 10% glycerol) with a DNA probe and target protein for 30 min. Before running the reaction for 60 min at 100 V, we pre-ran the gel for 30 min. We used GelRed nucleic acid dye to visualize the DNA. The EMSA results were photographed by using a gel imaging system (Bio-Rad). For the EMSA performed with DIDC-DIDC, the probes were FITC modified, and the results were visualized through florescence.

### LM-seq and data analysis.

The LM-seq procedure was done following a previous study with slight modifications (61). In brief, the overnight culture was transferred to a fresh medium (1:100) (Table S1) until the OD_600_ reached 0.6. Then, the strain was pelleted and washed before RNA extraction using a bacteria total RNA isolation kit (Sangon Biotech). Subsequently, the mRNA was enriched by MICROBExpress (Thermo Fisher) kit. The fragmentation and reverse transcription were done using the Clontech Smart Scribe Kit (TaKaRa). The RNA was removed using RNase H and RNase A before cleanup using VAHTS DNA Clean Beads (Vazyme). Phusion was used in the final PCR before the adapter was ligated to the cDNA by T4 RNA ligase 1 (NEB).

The raw data were mapped to the Watson strand and the Crick strand of the P. aeruginosa (NC_002516) and P. syringae (NC_005773.3) genomes by Bowtie2 (58), respectively. The sense and antisense reads were selected using BEDTools (59) and visualized using deepTools (35). The RNA reads were normalized, and the average read density per nucleotide was set to one. The TSSs were calculated using Rockhopper (48).

### Ribo-seq library construction and analysis.

The construction of the library followed a previous protocol. In brief, overnight cultures of P. syringae WT and Δ*rhpS* strains were transferred into fresh KB medium. After 6 h of culture, chloramphenicol was added before centrifugation. The pellet was resuspended in lysis buffer (RLT buffer [Qiagen], B-mercaptoethanol, Superase-In, and chloramphenicol) and fast-frozen in liquid nitrogen. Sodium deoxycholate was added after thawing the lysate on ice. The supernatant was transferred into a new tube and digested with MNase in MNase buffer (Tris-HCl [pH 8.0], NH_4_Cl, CaCl_2_, MgOAc, and chloramphenicol). Sephacryl S400 MicroSpin columns were used to purify the MNase-digested products. The sRNA was separated using a Zymo RNA kit, and the rRNA was removed using a Ribo-Zero-rRNA Removal Kit. The final library was constructed using the NEBNext Small RNA Library Prep Set. The library was analyzed using the MetaRiboSeq pipeline (https://github.com/bhattlab/bhattlab_workflows/tree/master/metariboseq). The translational efficiency was calculated by dividing the normalized Ribo-seq counts by the normalized RNA counts.

### ChIP-qPCR.

The overnight bacteria cultures were transferred to a fresh medium containing the appropriate antibiotics until the mid-log-phase (OD_600_ = 0.6) was reached. The cross-link was performed by adding formaldehyde to a 1% concentration for 10 min and quenching with glycine. Then, the bacteria were pelleted and washed with a Tris buffer (20 mM Tris-HCl [pH 7.5] and 150 mM NaCl). Prior to sonication, the bacteria were resuspended in in IP buffer (50 mM HEPES–KOH [pH 7.5], 150 mM NaCl, 1 mM EDTA, 1% Triton X-100, 0.1% sodium deoxycholate, 0.1% SDS, and mini-protease inhibitor cocktail [Roche]). The cell lysis was centrifuged, and the supernatant was incubated with RNA Polymerase ImmunoAffinity Resin (number 673601). The RNAP binding DNA fragments were eluted after proteinase K digestion. The purified DNA fragments were used to perform downstream qPCR.

### Statistical analysis.

Student's *t* tests were performed to analyze the RT-qPCR results in R. The Wilcoxon test was used to analyze the FIMO score differences in R. *, *P* < 0.05; **, *P* < 0.01; and ***, *P* < 0.001. Results are presented as means ± standard deviations. All experiments were repeated at least three times.

### Data availability.

The data generated in this study were downloaded from public databases (9, 10, 14, 15, 17–19, 31). The LM-seq data were uploaded to GSE175852. Codes are available upon reasonable request.
