# Supplementary figures and images for: Bacterial Transcription Factors Bind to Coding Regions and Regulate Internal Cryptic Promoters
Source: mBio. 2022 Oct 6;13(5):e01643-22. doi: 10.1128/mbio.01643-22 (PMC9600179; doi:10.1128/mbio.01643-22)

**A**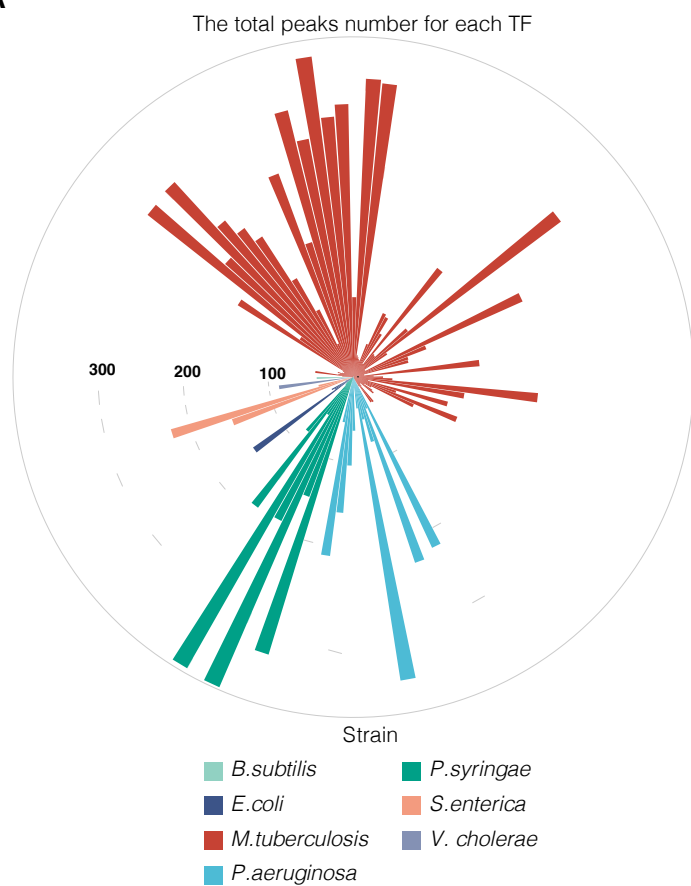**B**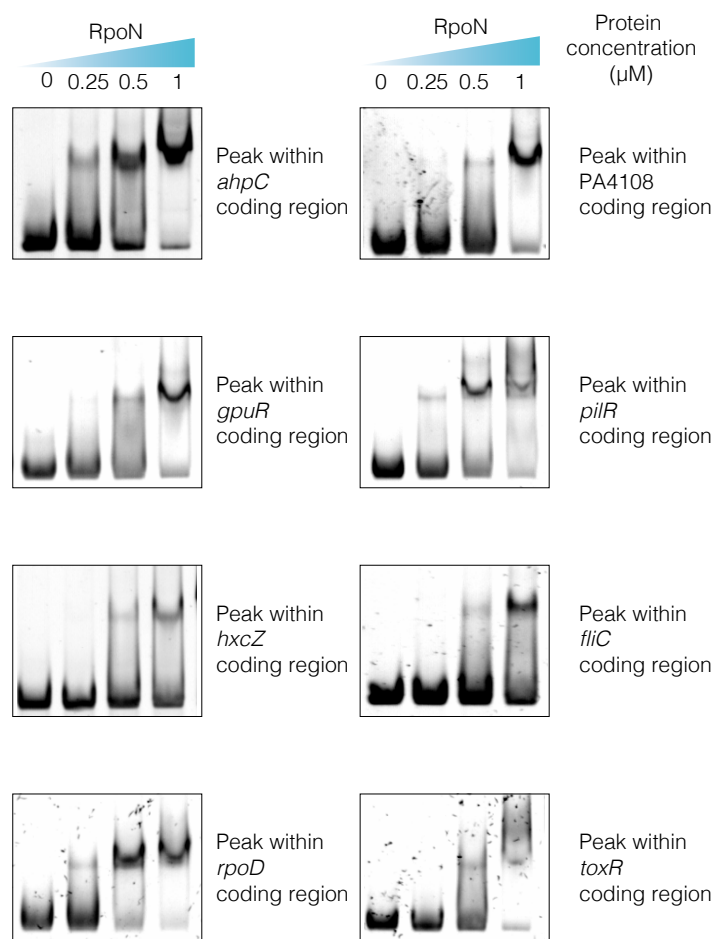**C**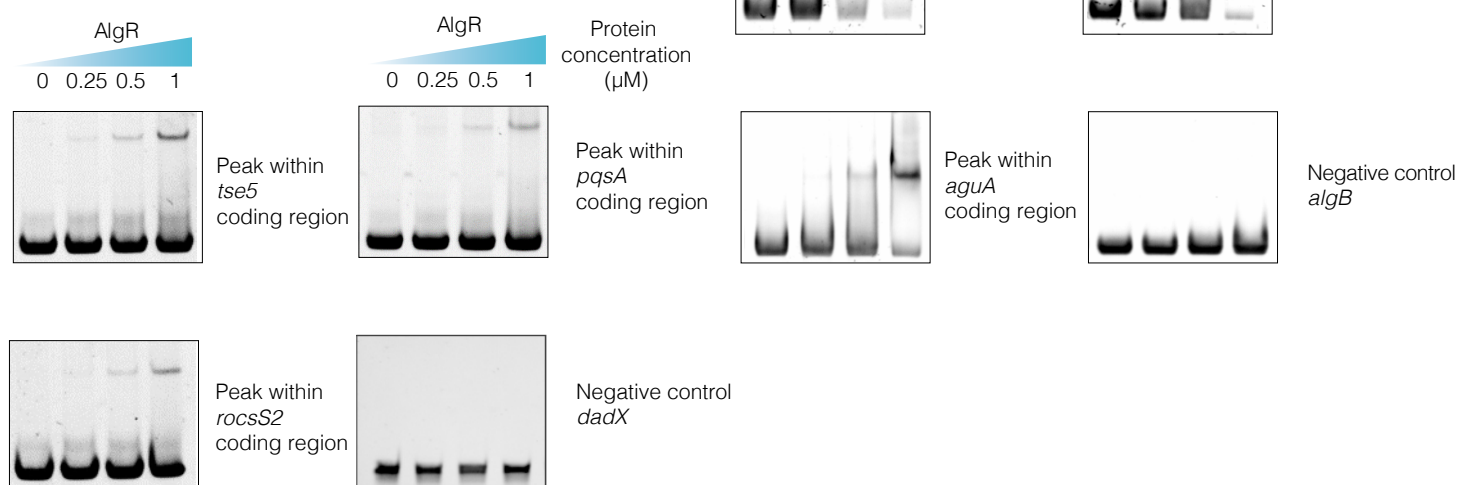

Supplement: FIG S1 [file mbio.01643-22-s0001.pdf]

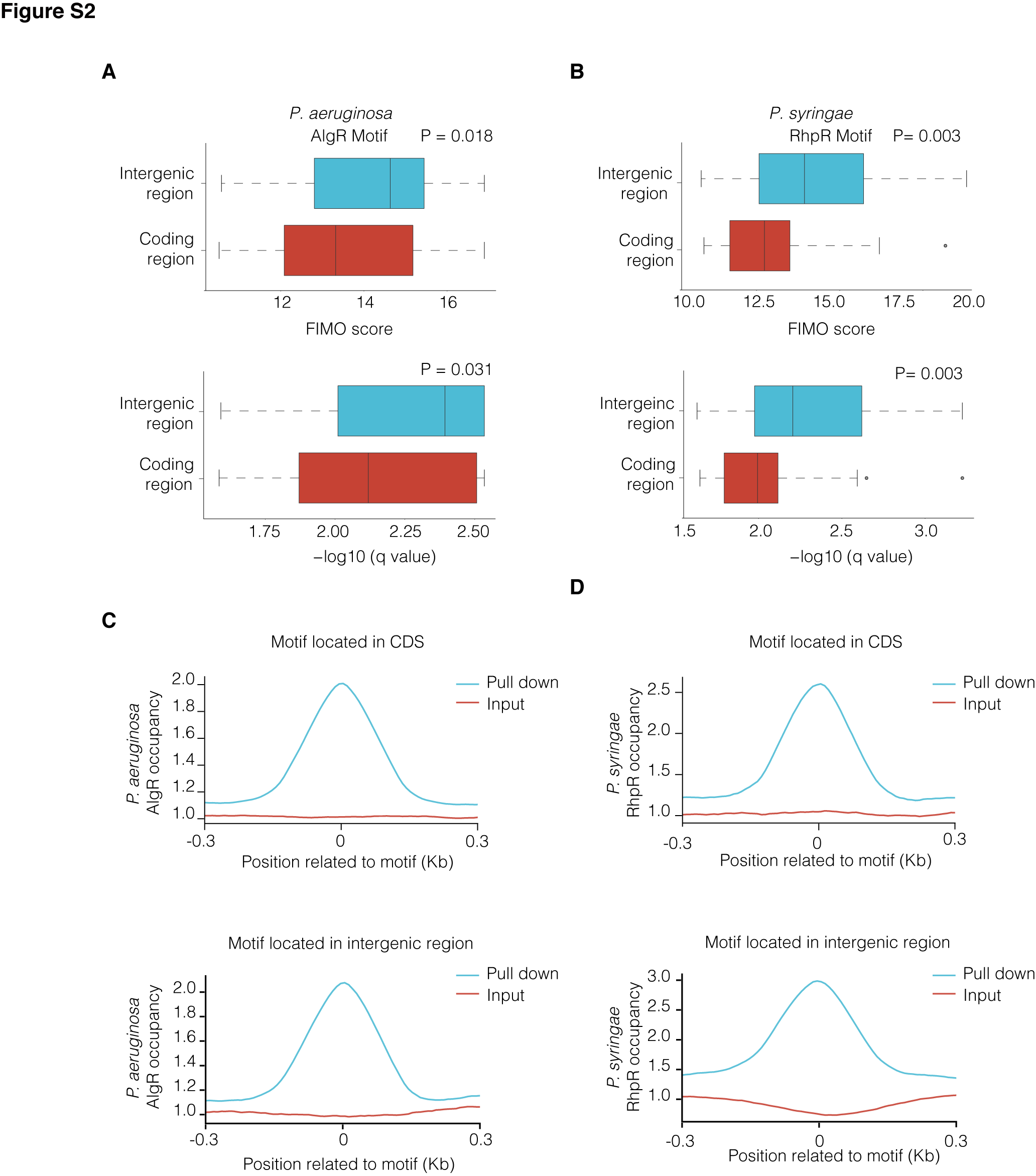

Supplement: FIG S2 [file mbio.01643-22-s0002.tif]

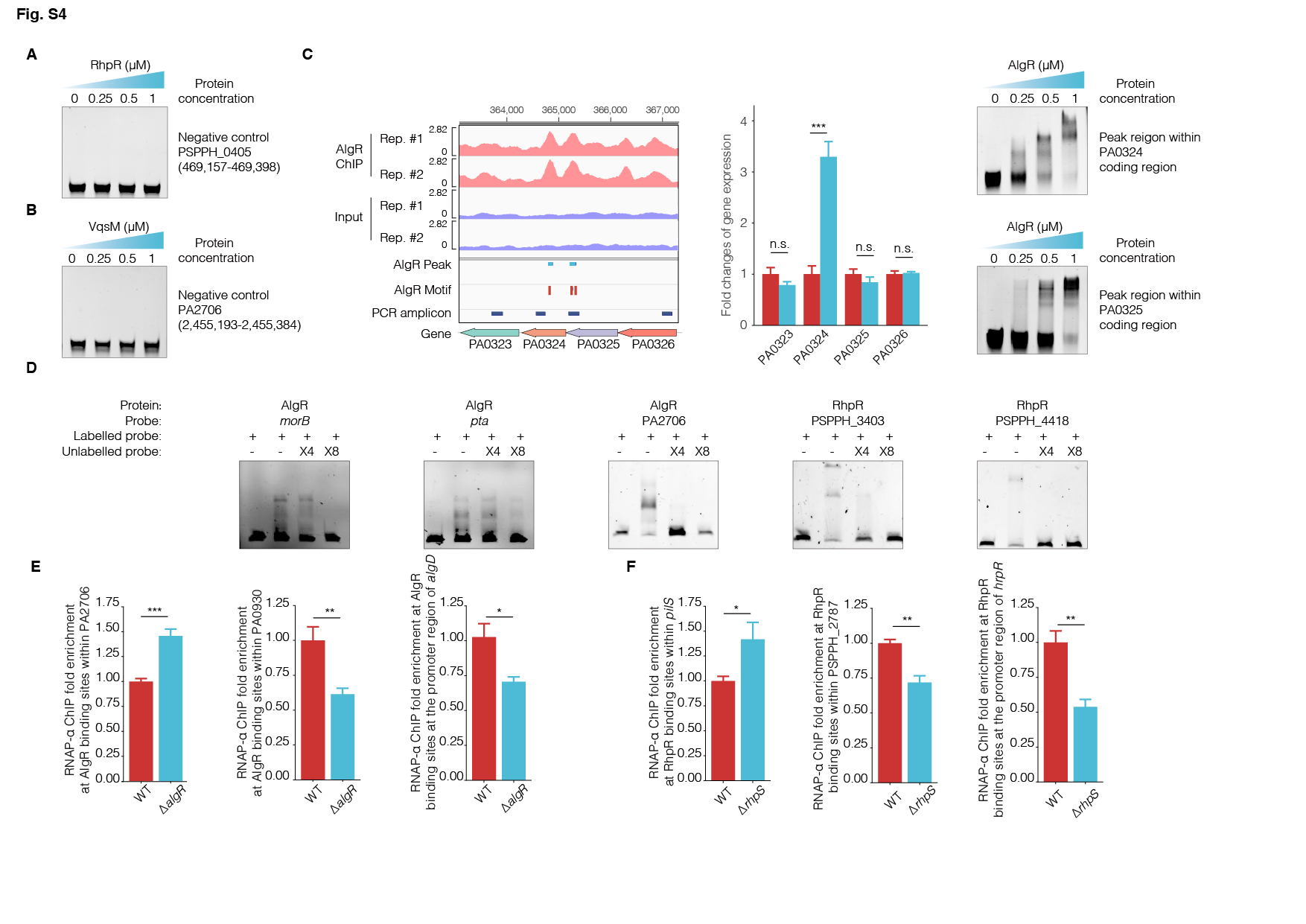

Supplement: FIG S4 [file mbio.01643-22-s0004.tif]

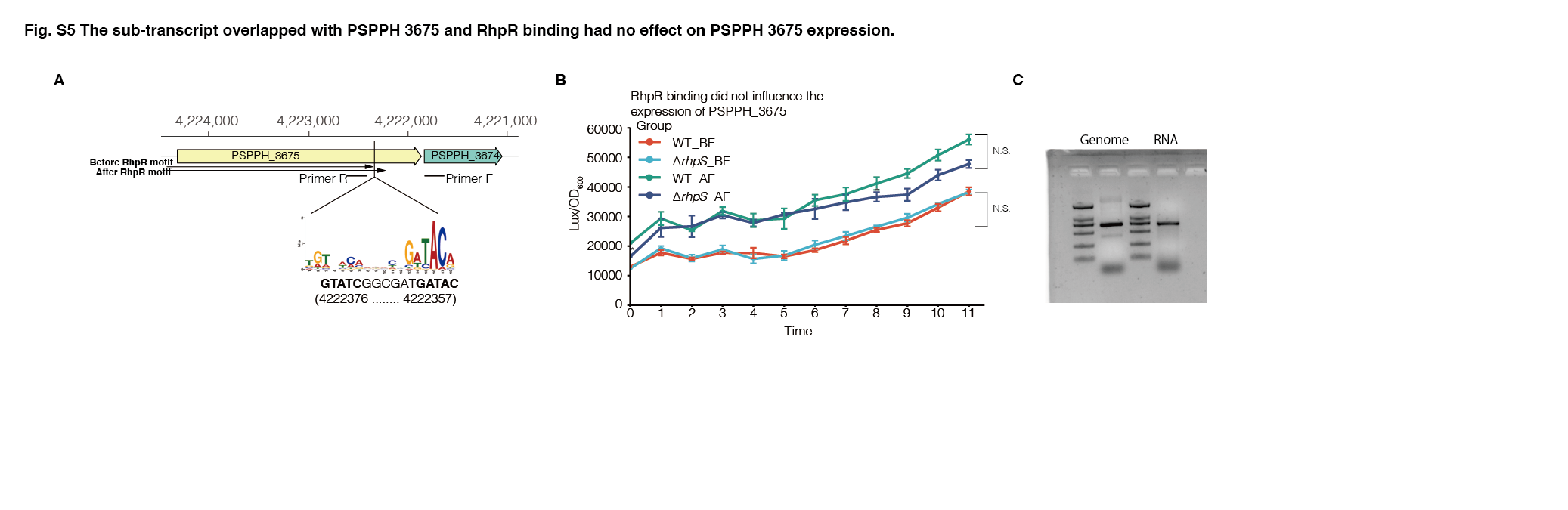

Supplement: FIG S5 [file mbio.01643-22-s0005.tif]
