# Supplementary material for: Bacterial Transcription Factors Bind to Coding Regions and Regulate Internal Cryptic Promoters
Source: mBio. 2022 Oct 6;13(5):e01643-22. doi: 10.1128/mbio.01643-22 (PMC9600179; doi:10.1128/mbio.01643-22)
Supplement: FIG S3 [file mbio.01643-22-s0003.pdf]

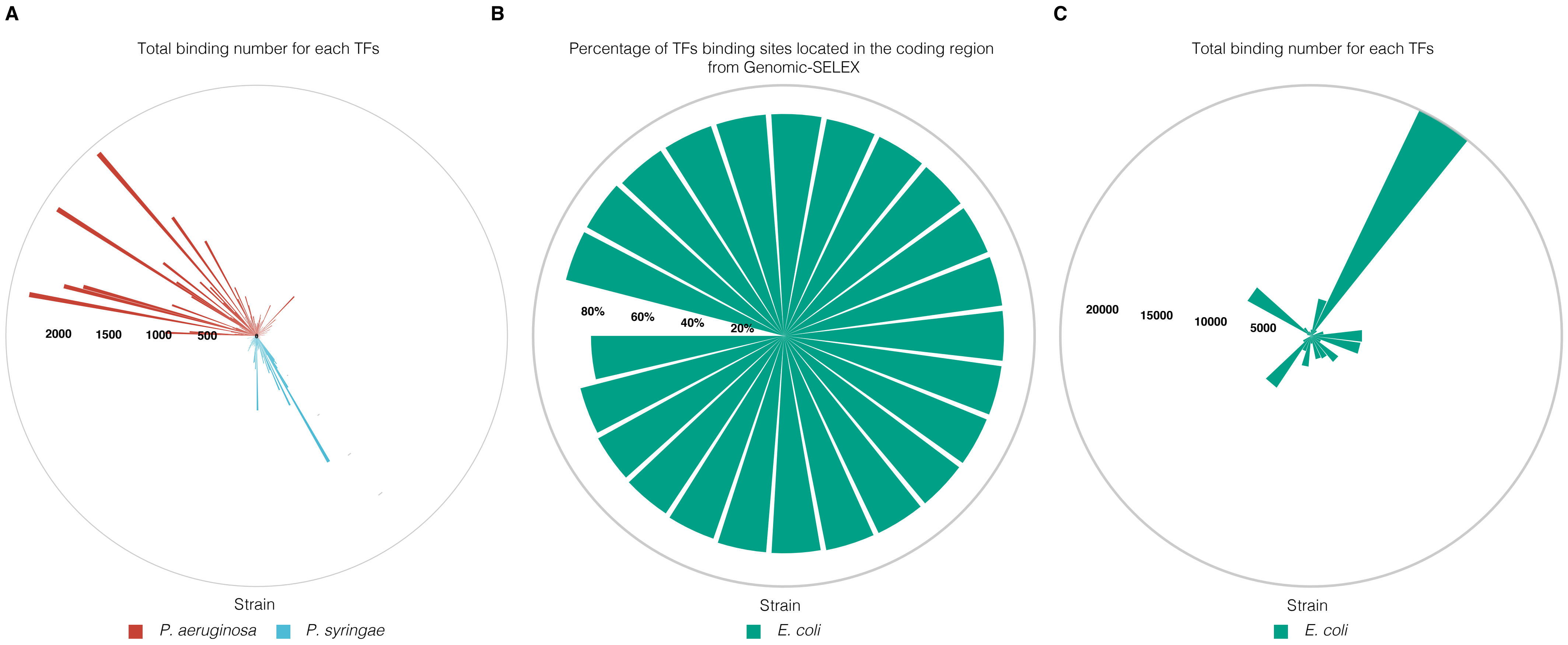
A
Total binding number for each TFs
2000
1500
1000
500
Strain
P. aeruginosa
P. syringae
B
Percentage of TFs binding sites located in the coding region from Genomic-SELEX
80%
60%
40%
20%
Strain
E. coli
C
Total binding number for each TFs
20000
15000
10000
5000
Strain
E. coli
