# Supplementary material for: Bacterial Transcription Factors Bind to Coding Regions and Regulate Internal Cryptic Promoters
Source: mBio. 2022 Oct 6;13(5):e01643-22. doi: 10.1128/mbio.01643-22 (PMC9600179; doi:10.1128/mbio.01643-22)
Supplement: DATA SET S1 [file mbio.01643-22-s0007.pdf]

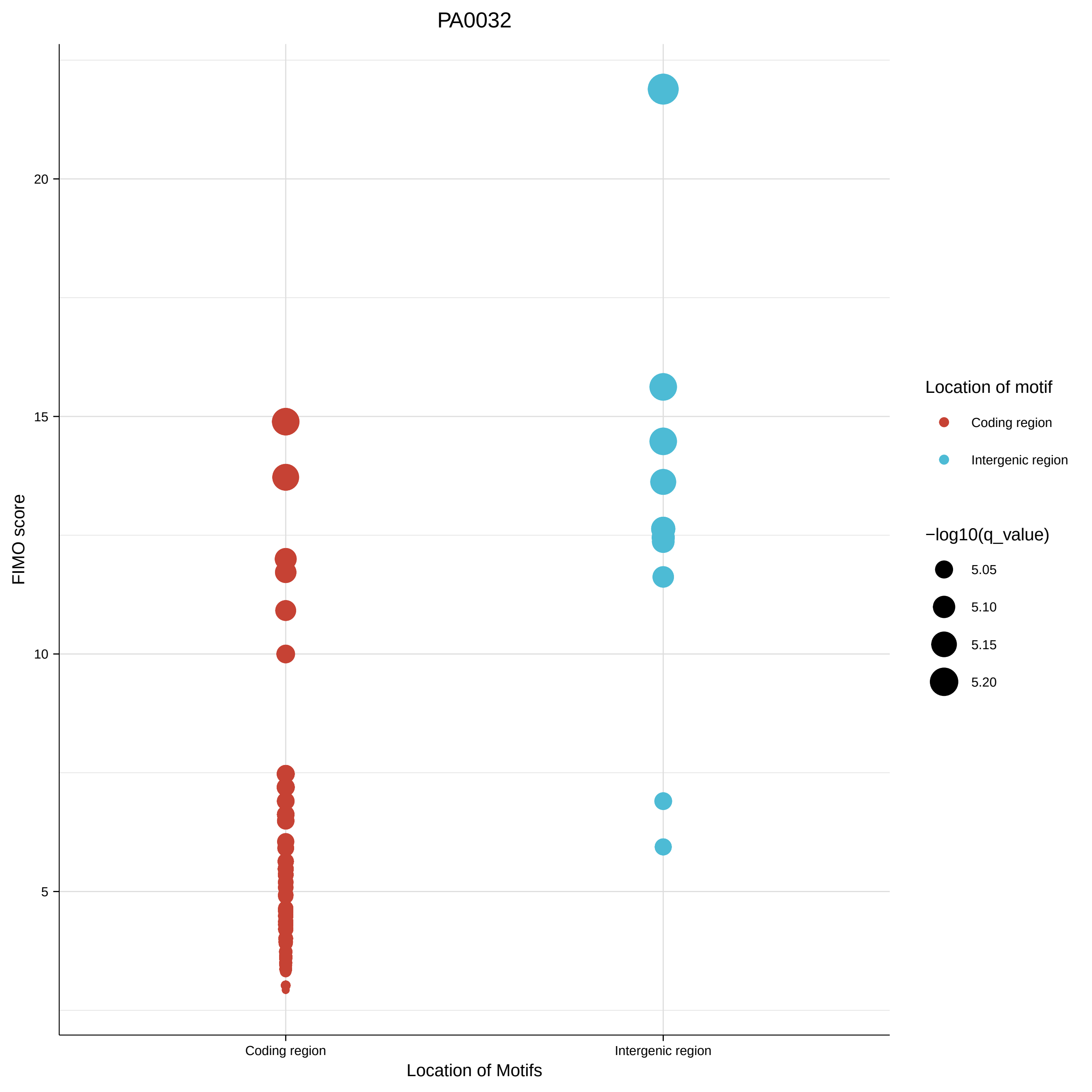

PA0032
FIMO score
20
15
10
5
Coding region
Intergenic region
Location of Motifs
Location of motif
Coding region
Intergenic region
−log10(q_value)
5.05
5.10
5.15
5.20

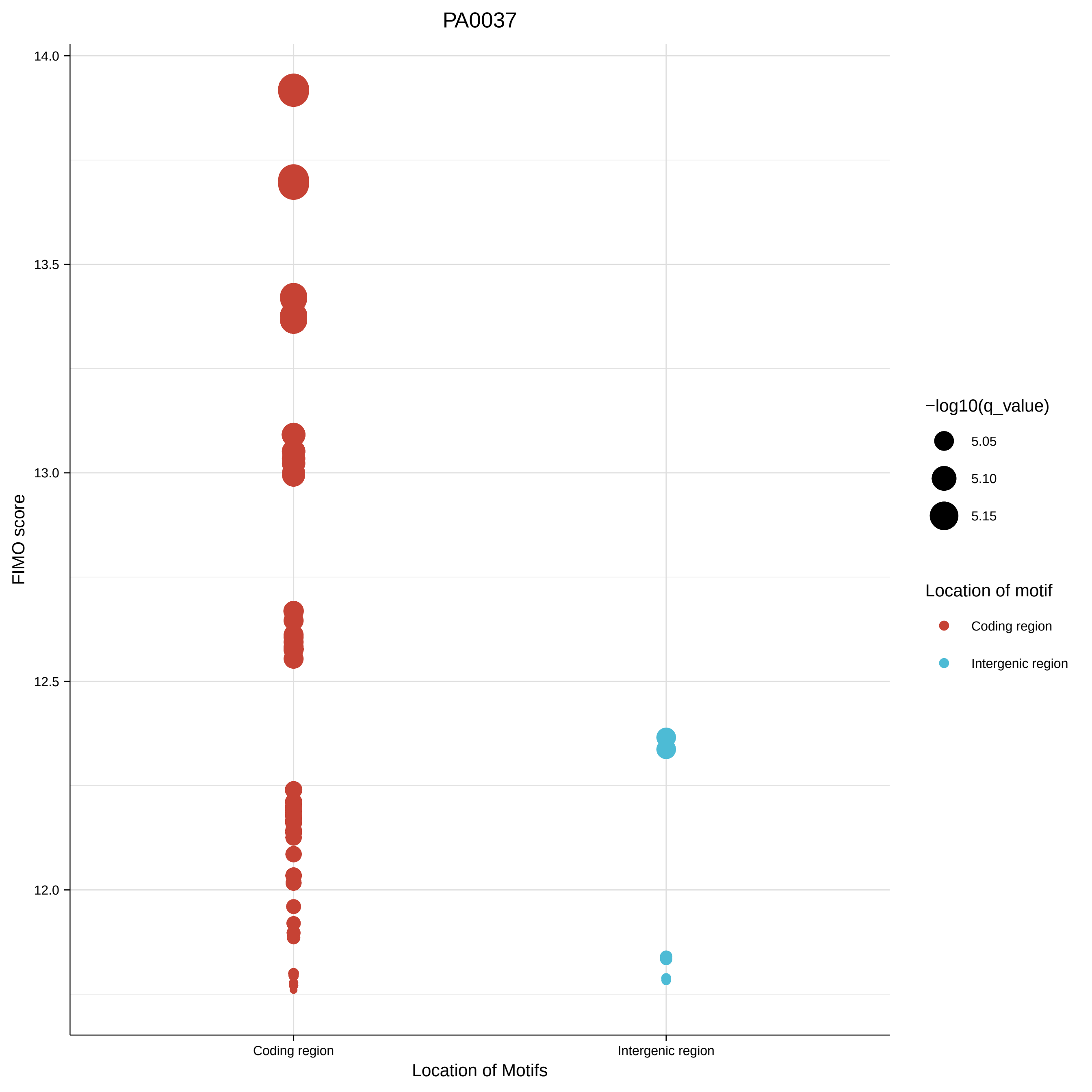
PA0037
FIMO score
14.0
13.5
13.0
12.5
12.0
Coding region
Intergenic region
Location of Motifs
−log10(q_value)
5.05
5.10
5.15
Location of motif
Coding region
Intergenic region

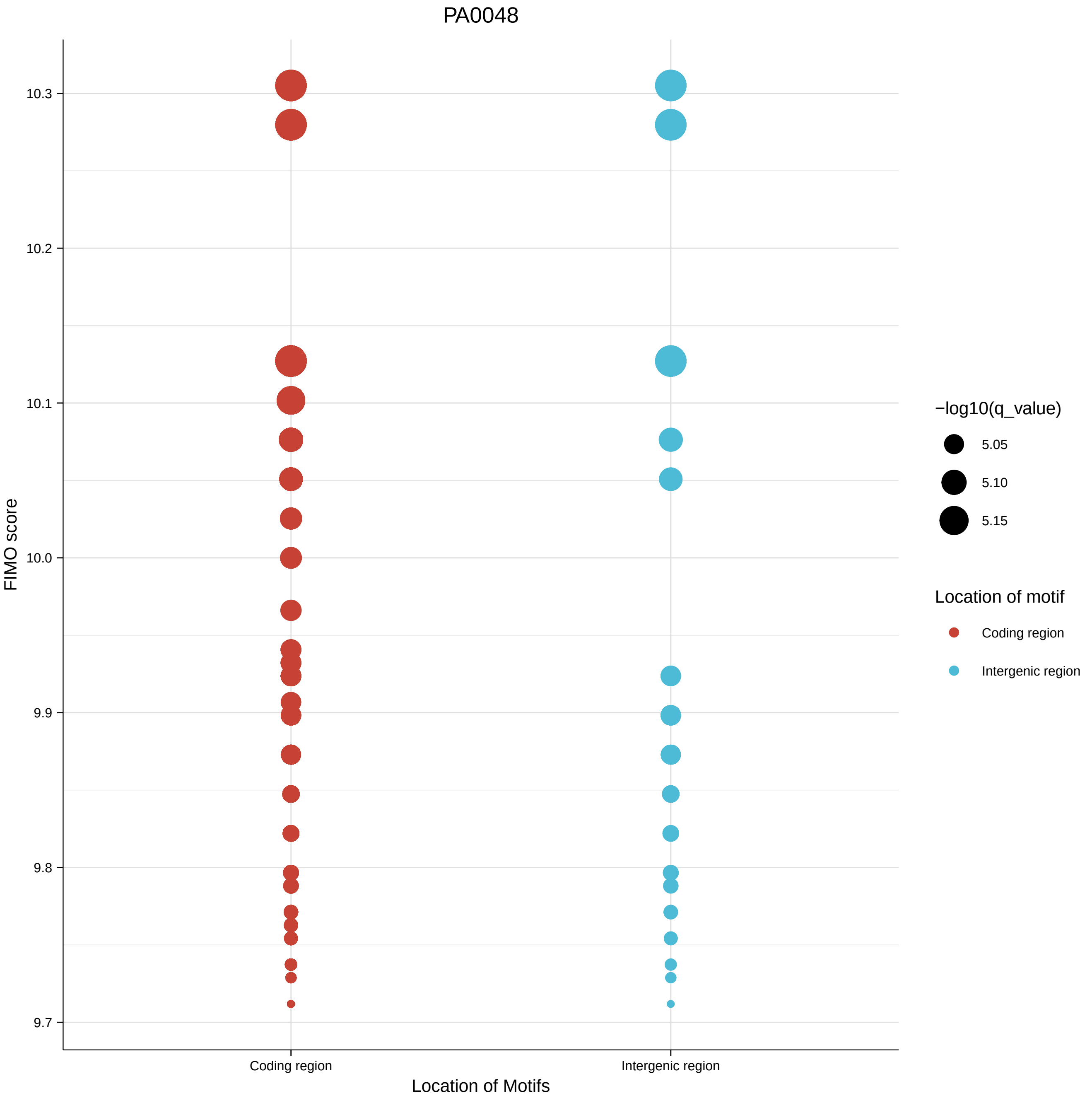
PA0048
FIMO score
10.3
10.2
10.1
10.0
9.9
9.8
9.7
Coding region
Intergenic region
Location of Motifs
−log10(q_value)
5.05
5.10
5.15
Location of motif
Coding region
Intergenic region

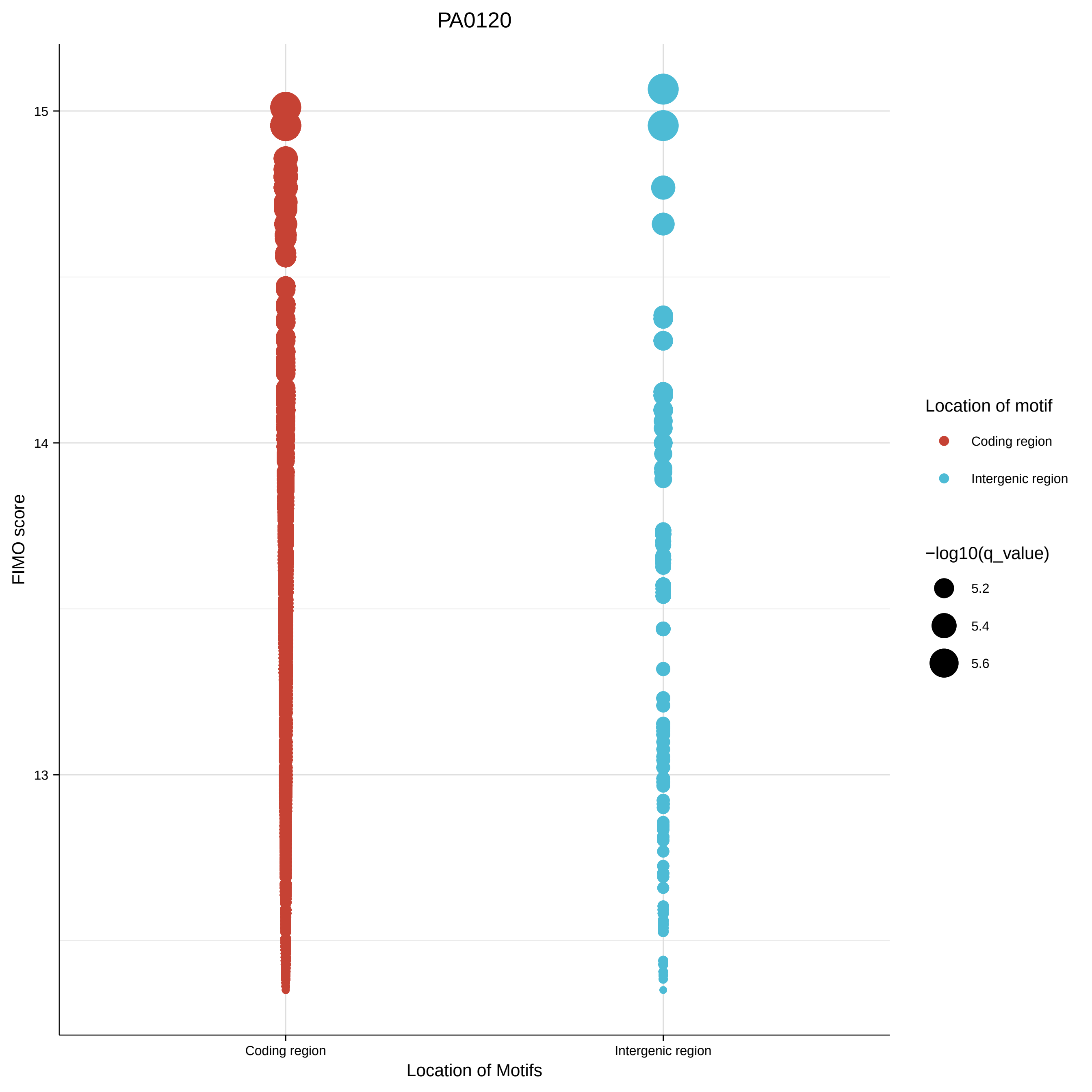

PA0120
FIMO score
15
14
13
Coding region
Intergenic region
Location of Motifs
Location of motif
Coding region
Intergenic region
−log10(q_value)
5.2
5.4
5.6

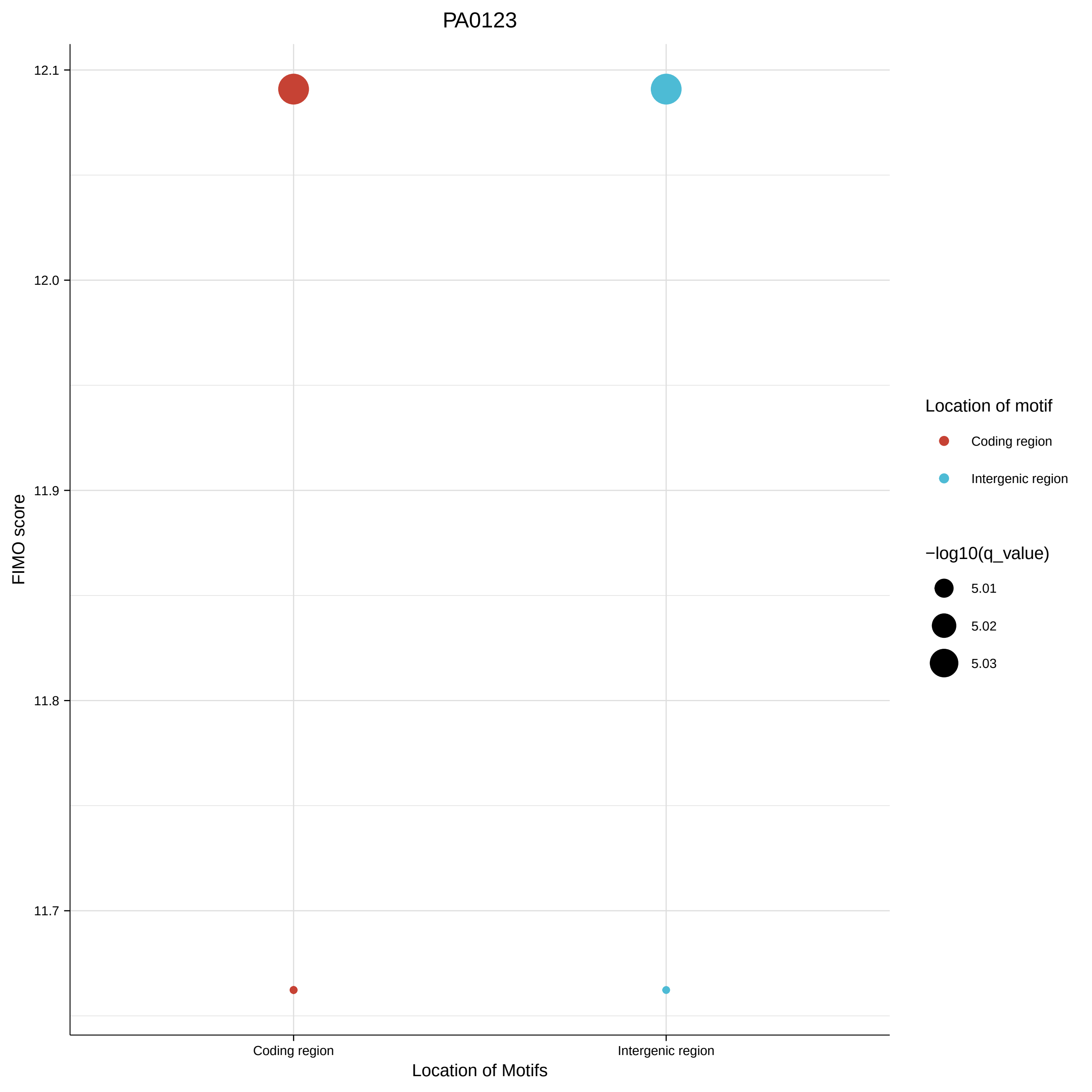
PA0123
FIMO score
12.1
12.0
11.9
11.8
11.7
Coding region
Intergenic region
Location of Motifs
Location of motif
Coding region
Intergenic region
−log10(q_value)
5.01
5.02
5.03

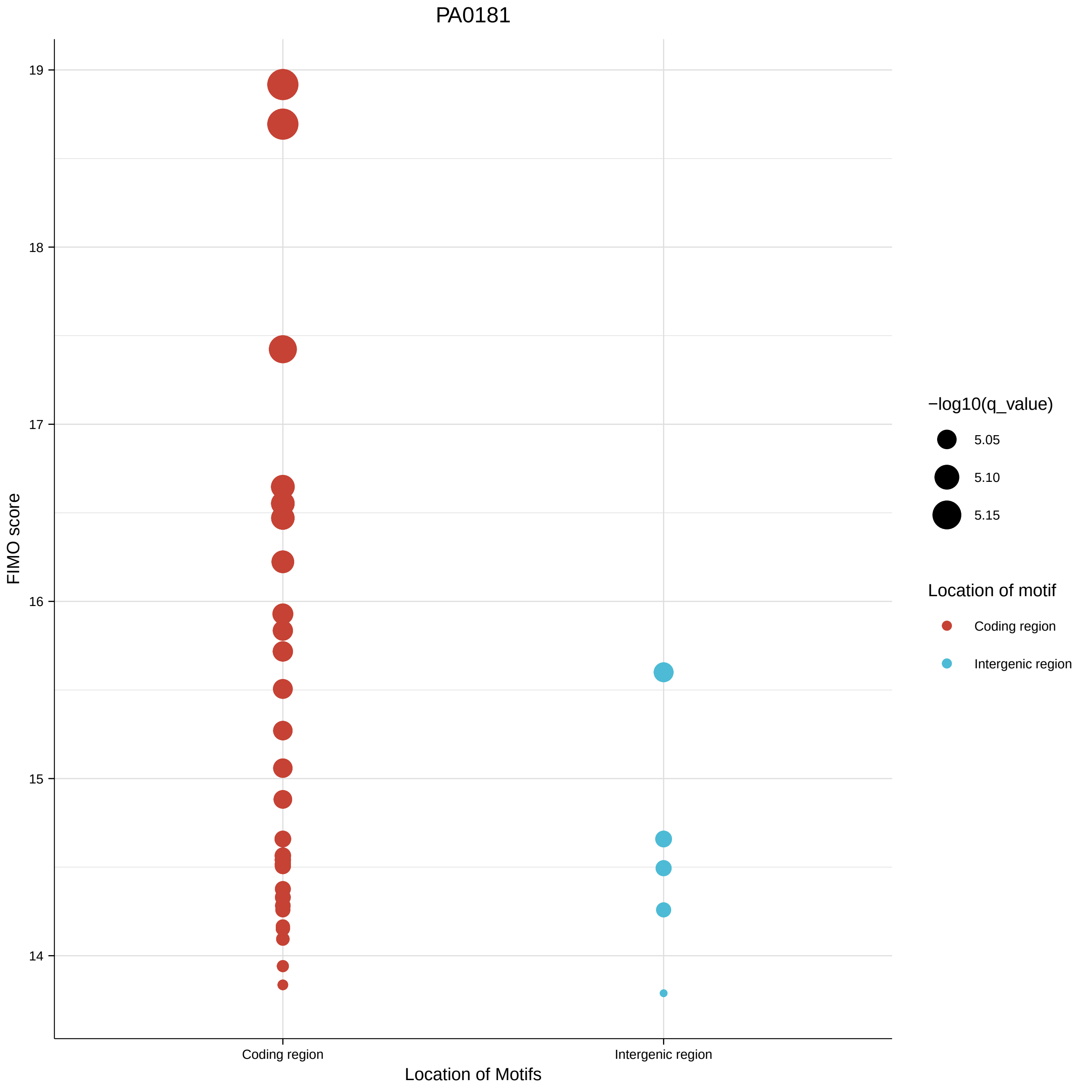

PA0181
FIMO score
19
18
17
16
15
14
Coding region
Intergenic region
Location of Motifs
−log10(q_value)
5.05
5.10
5.15
Location of motif
Coding region
Intergenic region

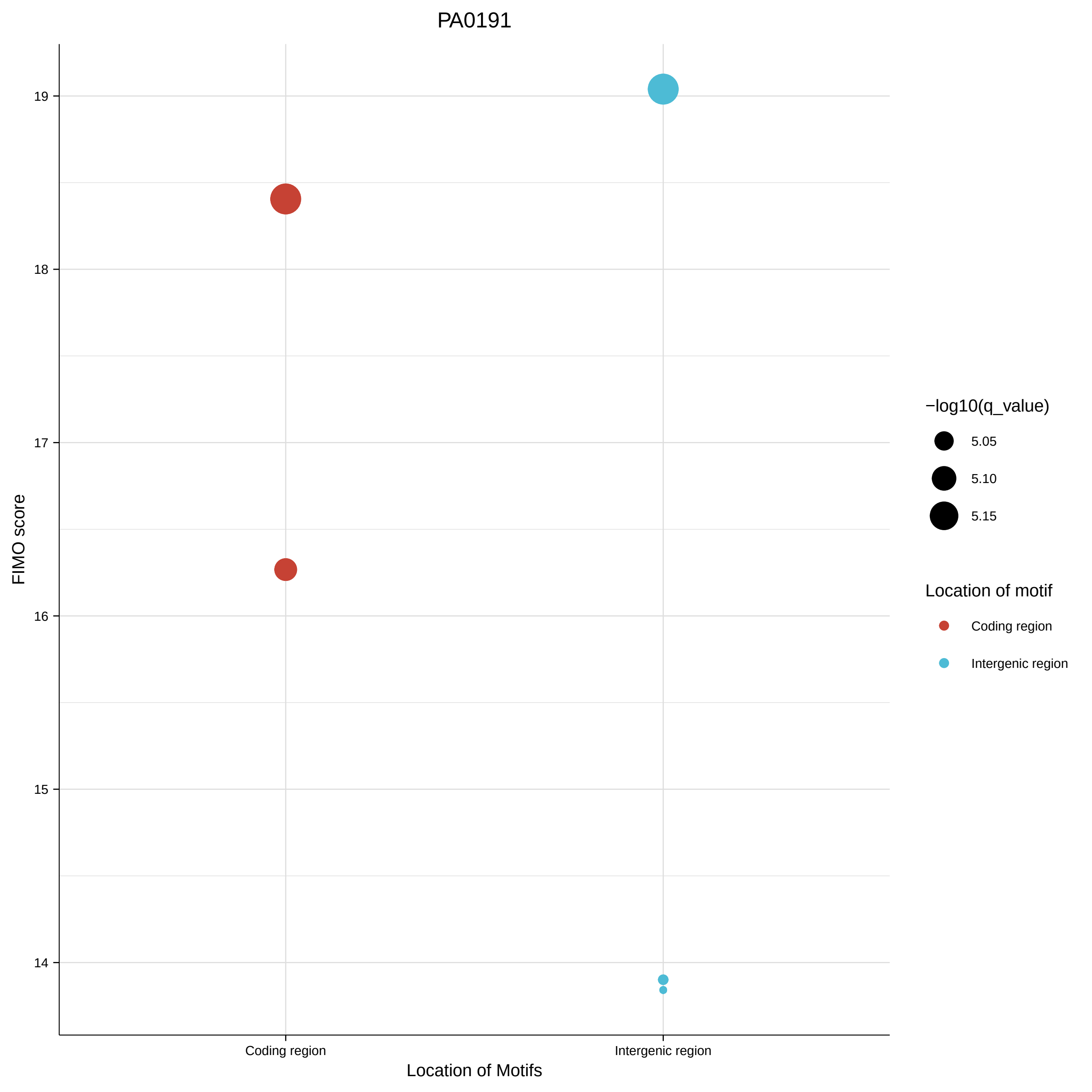

PA0191
FIMO score
19
18
17
16
15
14
Coding region
Intergenic region
Location of Motifs
−log10(q_value)
5.05
5.10
5.15
Location of motif
Coding region
Intergenic region

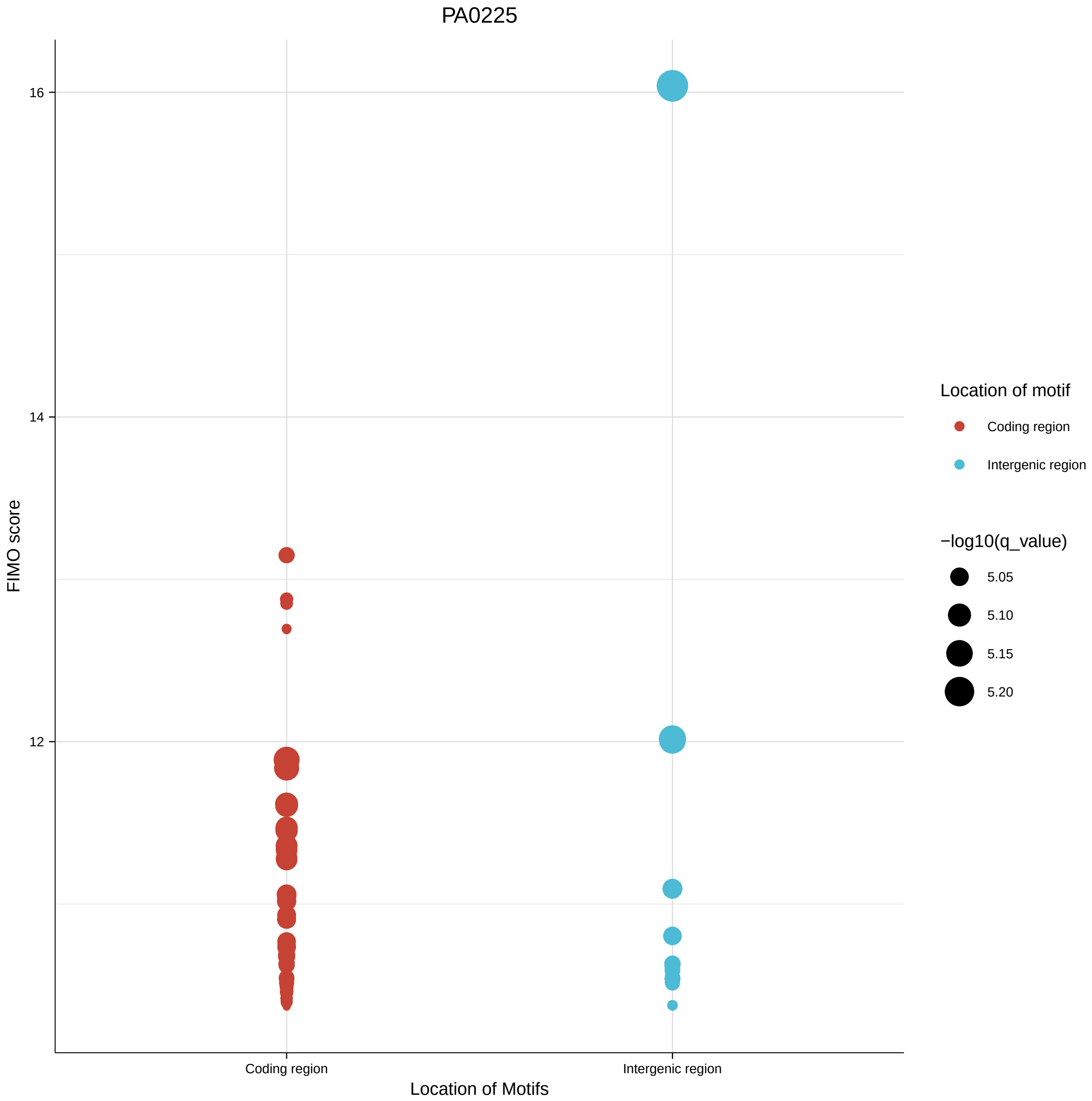
PA0225
FIMO score
16
14
12
Coding region
Intergenic region
Location of Motifs
Location of motif
Coding region
Intergenic region
−log10(q_value)
5.05
5.10
5.15
5.20

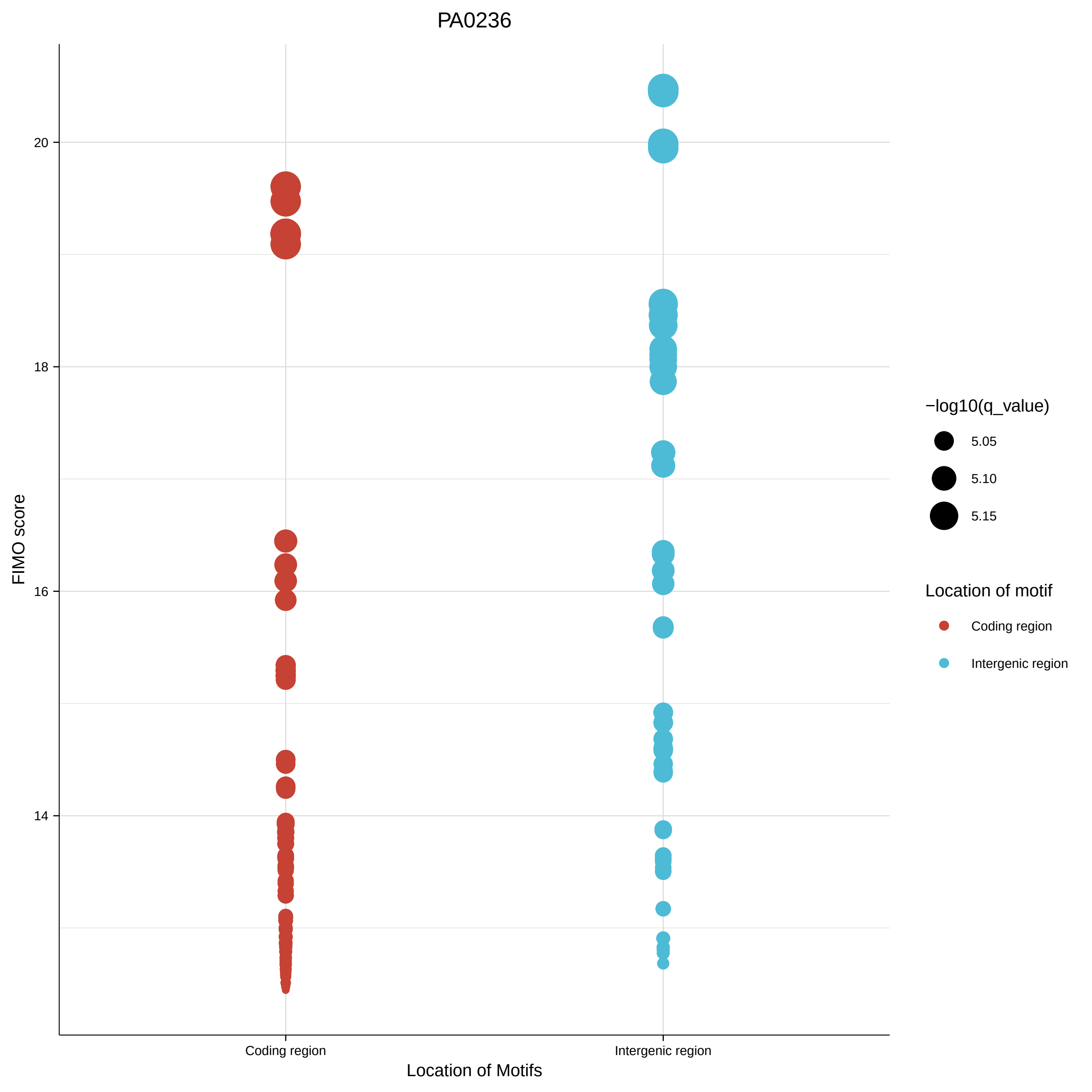

PA0236
FIMO score
20
18
16
14
Coding region
Intergenic region
Location of Motifs
−log10(q_value)
5.05
5.10
5.15
Location of motif
Coding region
Intergenic region

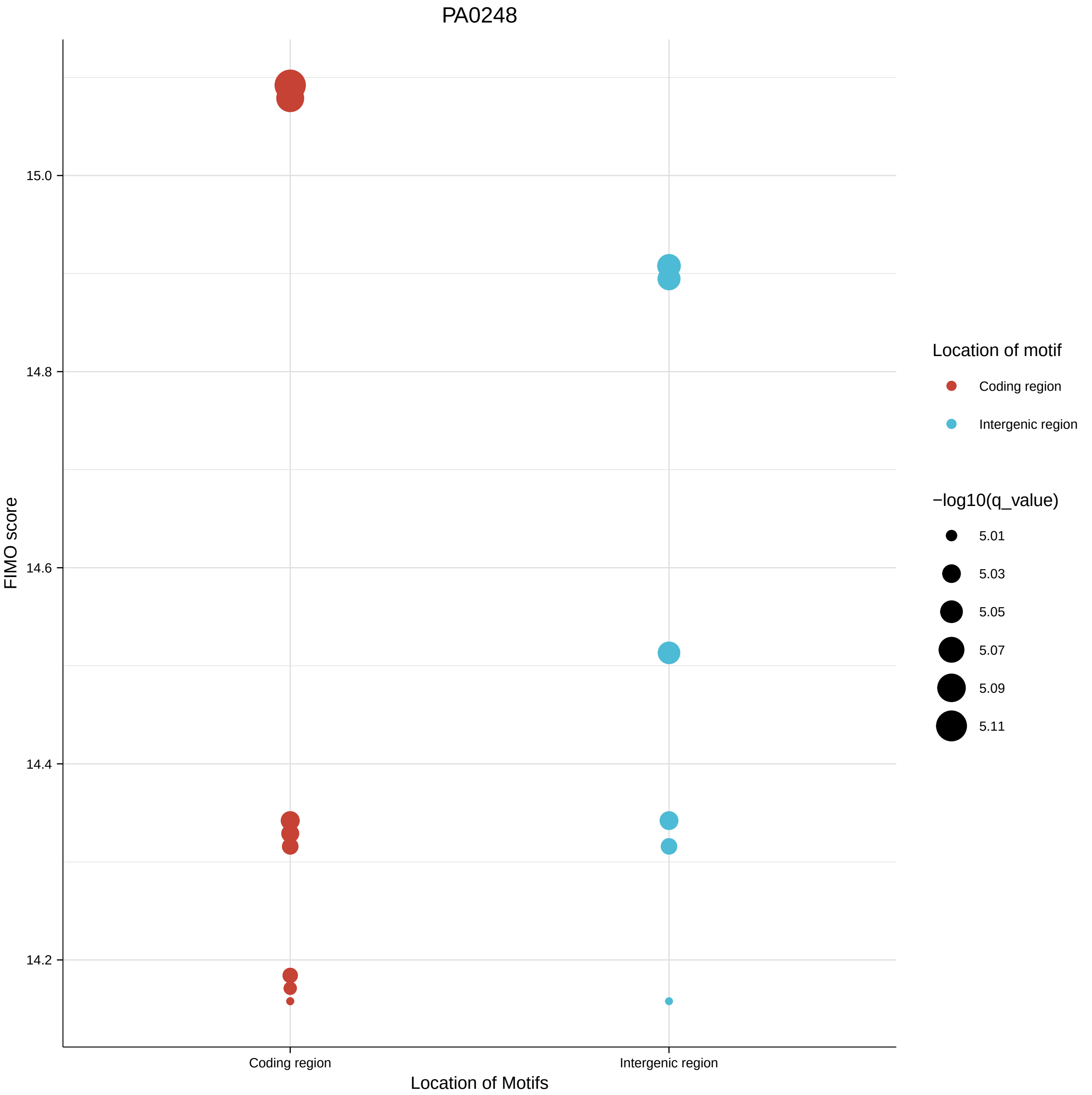
PA0248
FIMO score
15.0
14.8
14.6
14.4
14.2
Coding region
Intergenic region
Location of Motifs
Location of motif
Coding region
Intergenic region
−log10(q_value)
5.01
5.03
5.05
5.07
5.09
5.11

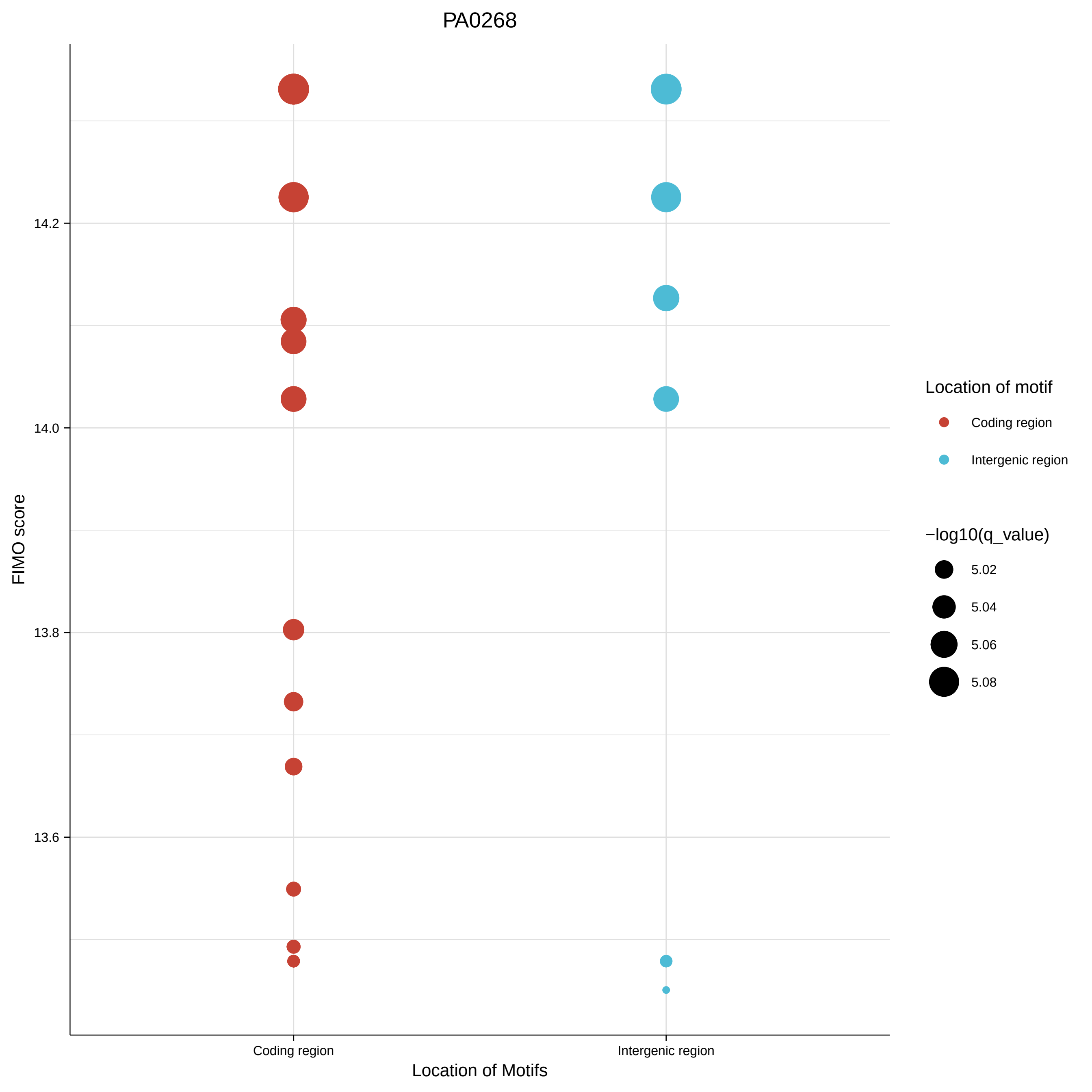
PA0268
FIMO score
14.2
14.0
13.8
13.6
Coding region
Intergenic region
Location of Motifs
Location of motif
Coding region
Intergenic region
−log10(q_value)
5.02
5.04
5.06
5.08

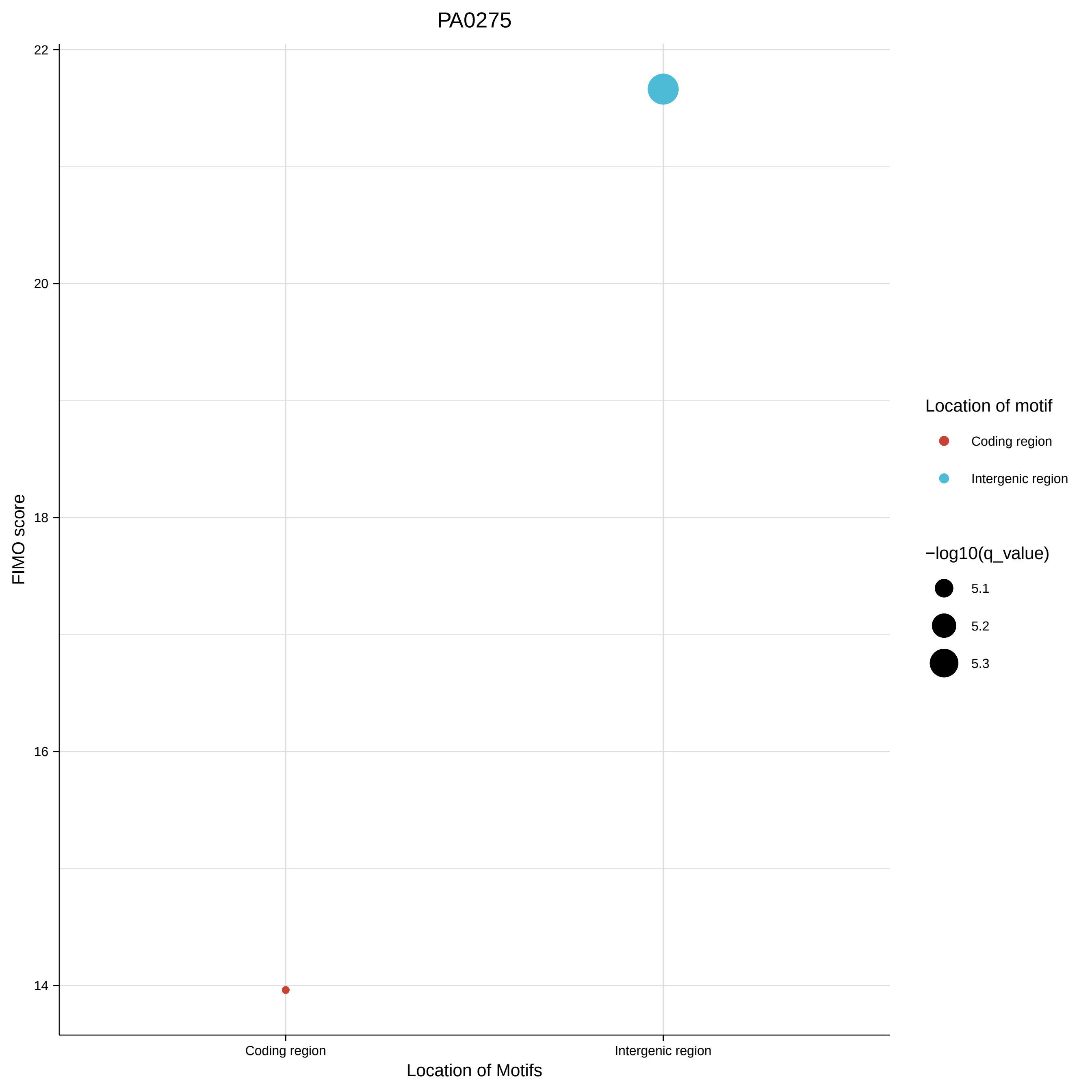

PA0275
22
20
18
16
14
FIMO score
Coding region
Intergenic region
Location of Motifs
Location of motif
Coding region
Intergenic region
−log10(q_value)
5.1
5.2
5.3

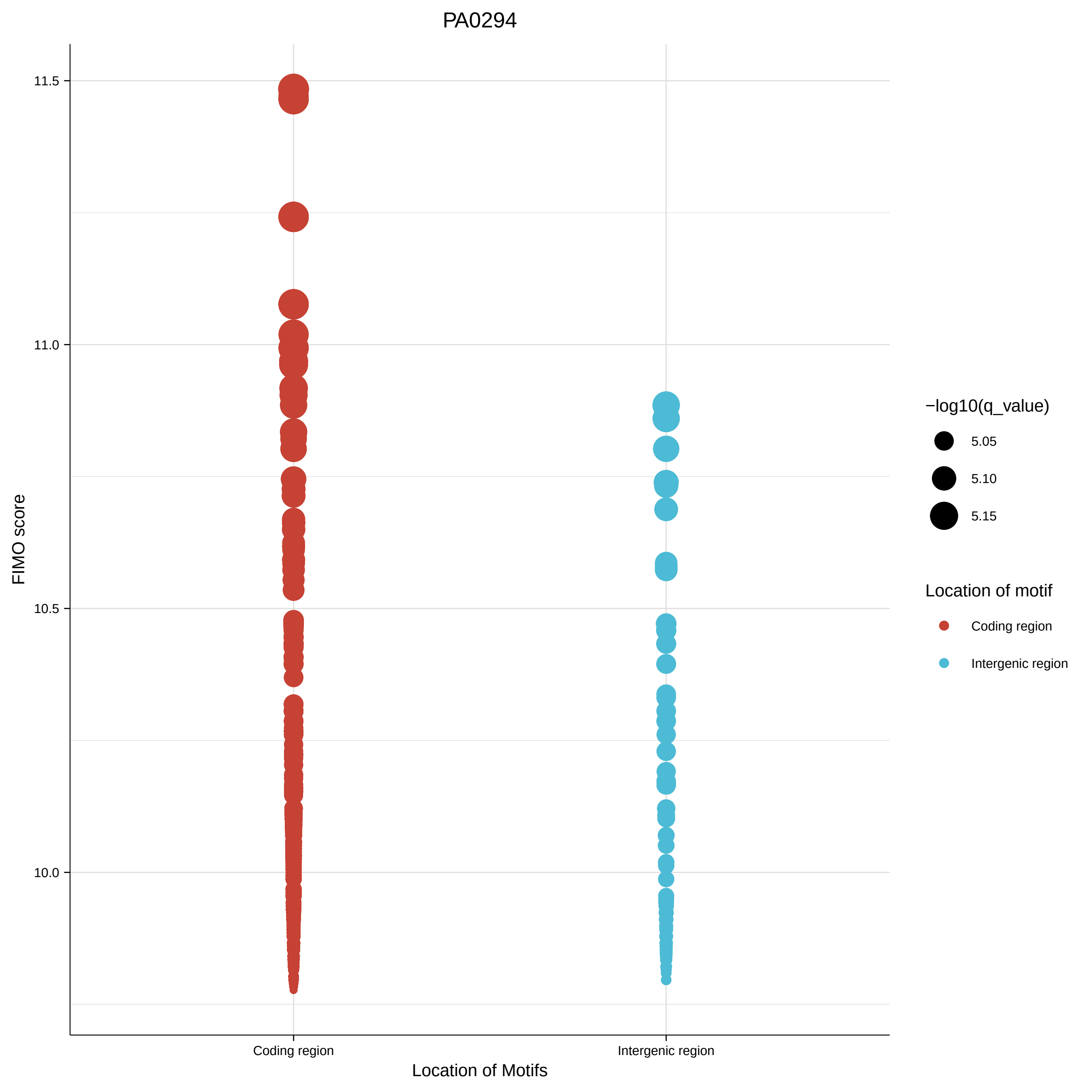

PA0294
FIMO score
11.5
11.0
10.5
10.0
Coding region
Intergenic region
Location of Motifs
−log10(q_value)
5.05
5.10
5.15
Location of motif
Coding region
Intergenic region

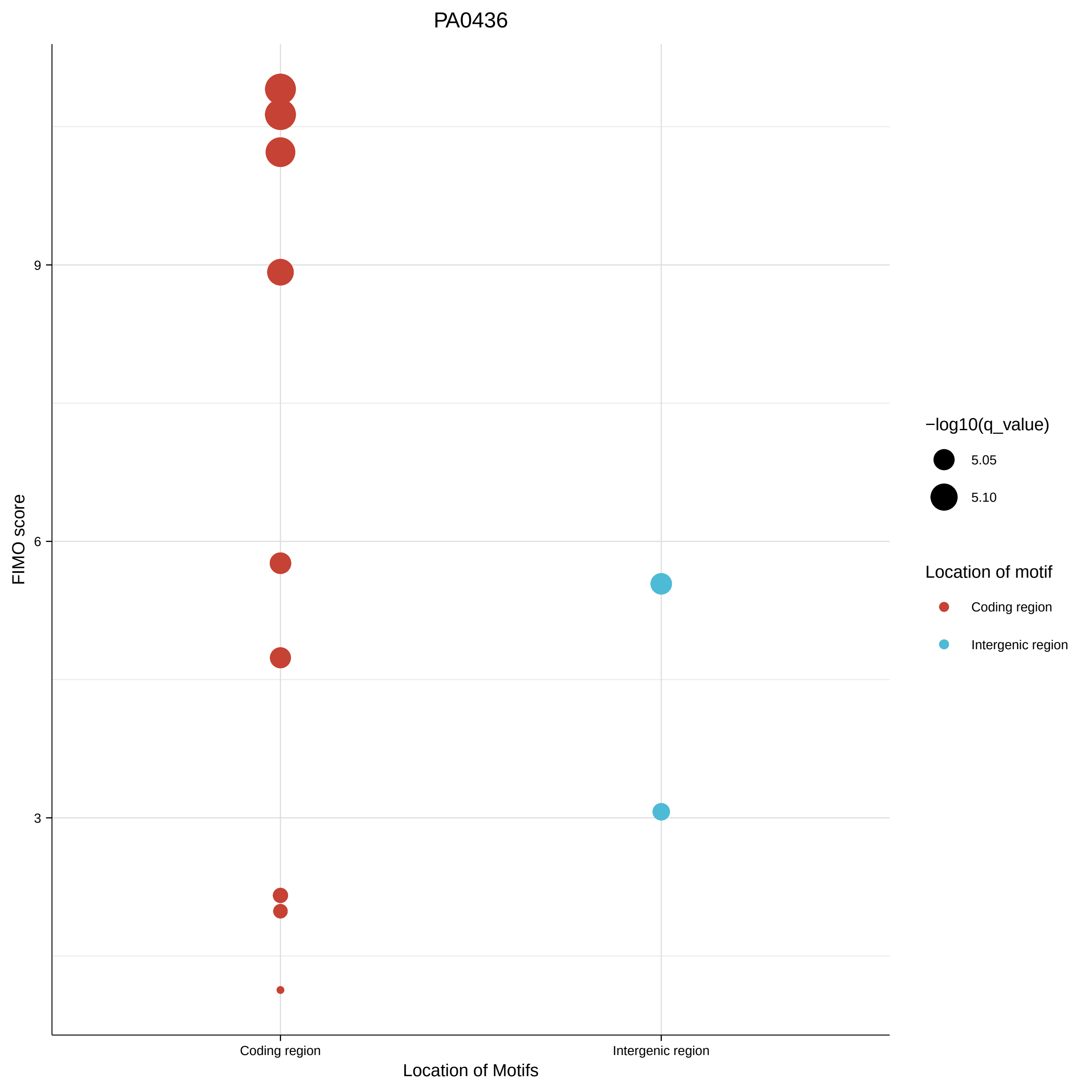
PA0436
FIMO score
9
6
3
Coding region
Intergenic region
Location of Motifs
−log10(q_value)
5.05
5.10
Location of motif
Coding region
Intergenic region

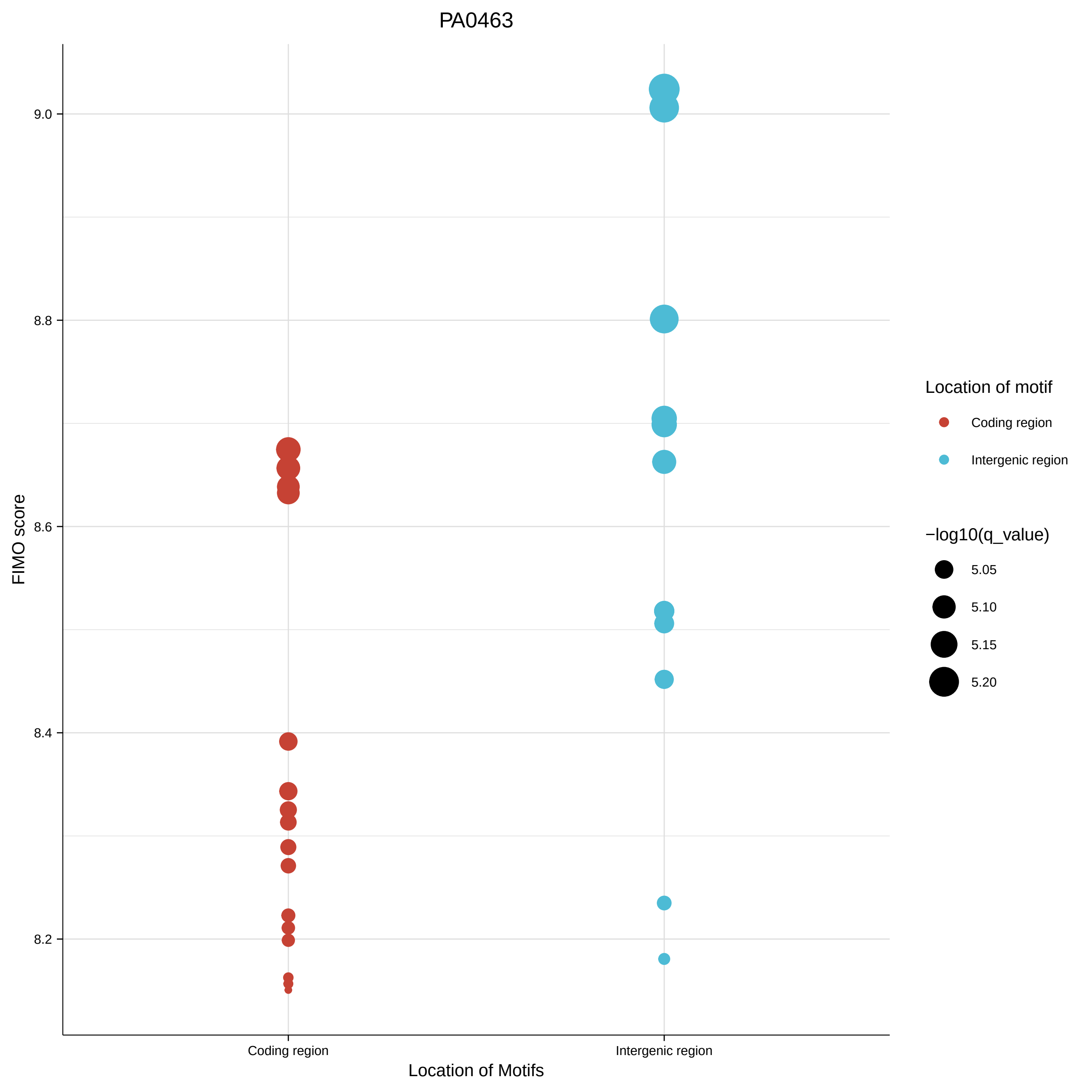

PA0463
FIMO score
9.0
8.8
8.6
8.4
8.2
Coding region
Intergenic region
Location of Motifs
Location of motif
Coding region
Intergenic region
−log10(q_value)
5.05
5.10
5.15
5.20

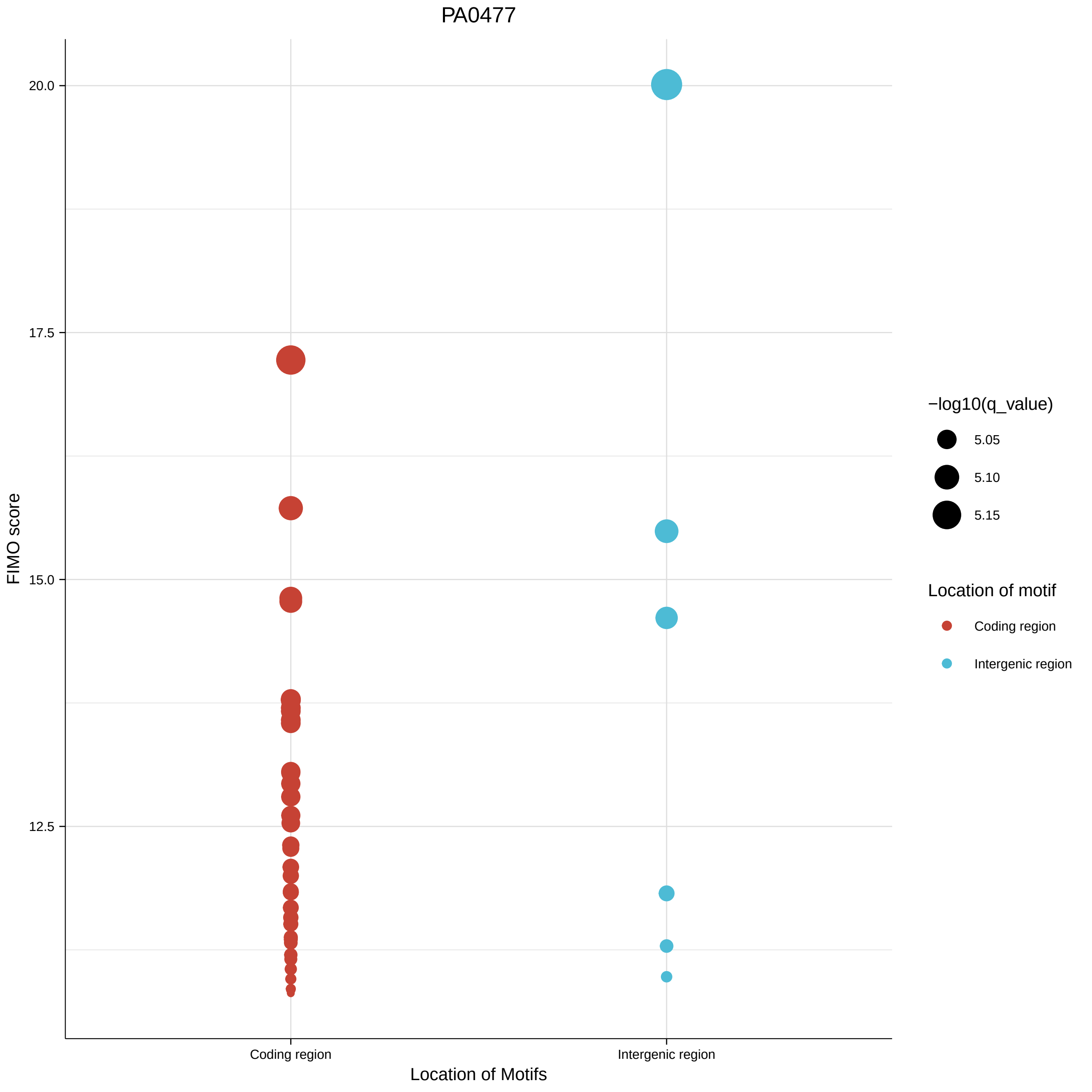

PA0477
20.0
17.5
15.0
12.5
FIMO score
Coding region
Intergenic region
Location of Motifs
−log10(q_value)
5.05
5.10
5.15
Location of motif
Coding region
Intergenic region

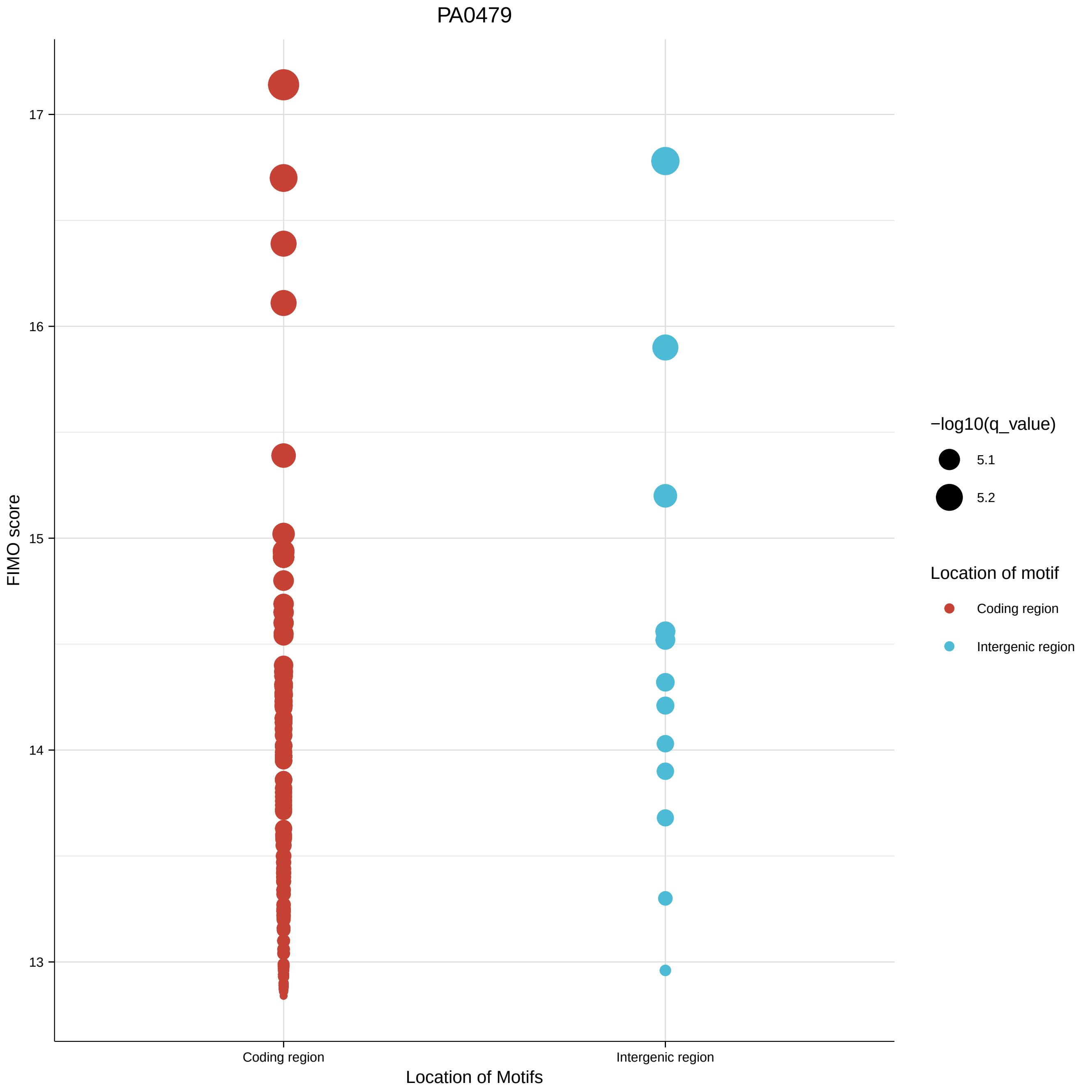

PA0479
FIMO score
17
16
15
14
13
Coding region
Intergenic region
Location of Motifs
−log10(q_value)
5.1
5.2
Location of motif
Coding region
Intergenic region

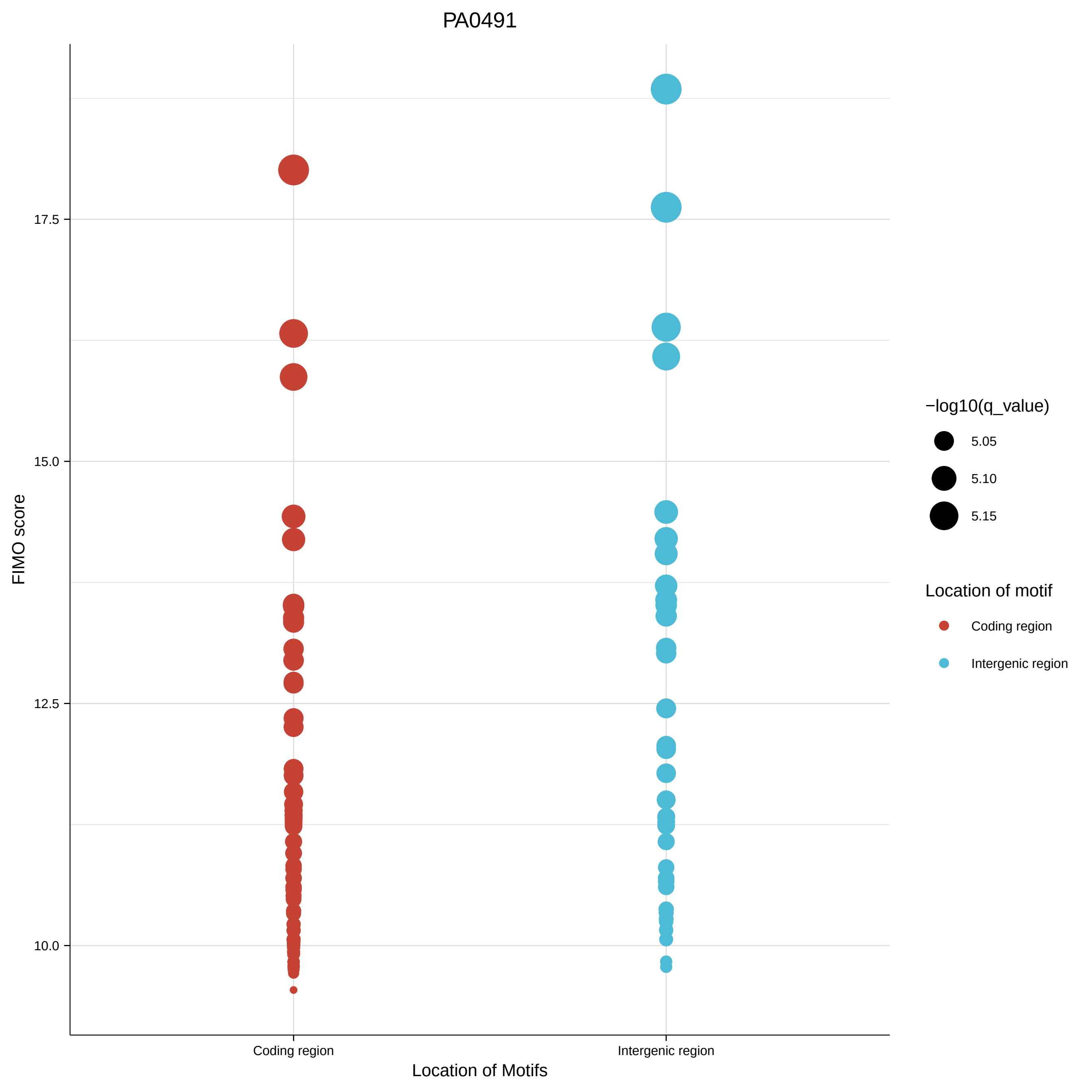

PA0491
FIMO score
17.5
15.0
12.5
10.0
Coding region
Intergenic region
Location of Motifs
−log10(q_value)
5.05
5.10
5.15
Location of motif
Coding region
Intergenic region

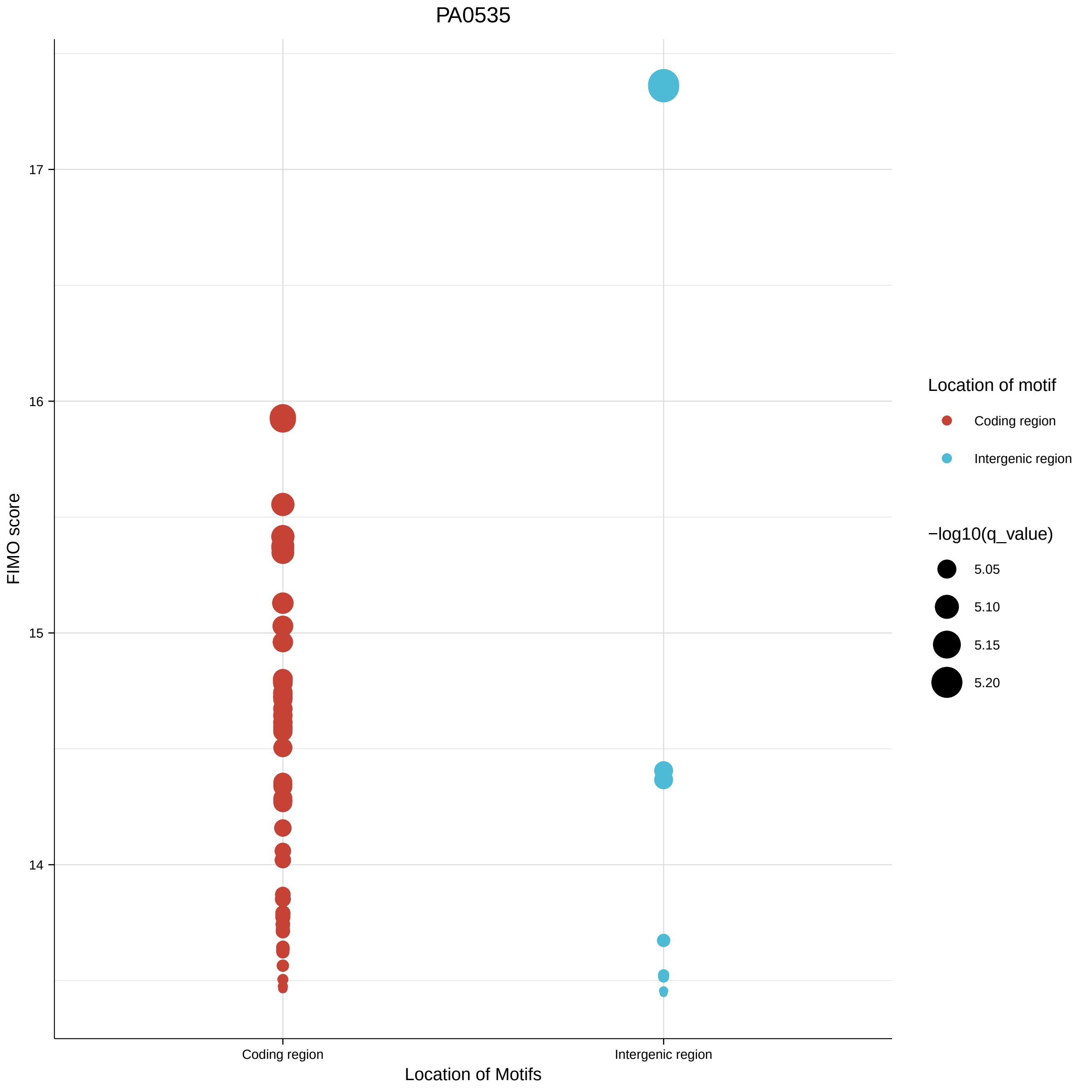
PA0535
FIMO score
17
16
15
14
Coding region
Intergenic region
Location of Motifs
Location of motif
Coding region
Intergenic region
−log10(q_value)
5.05
5.10
5.15
5.20

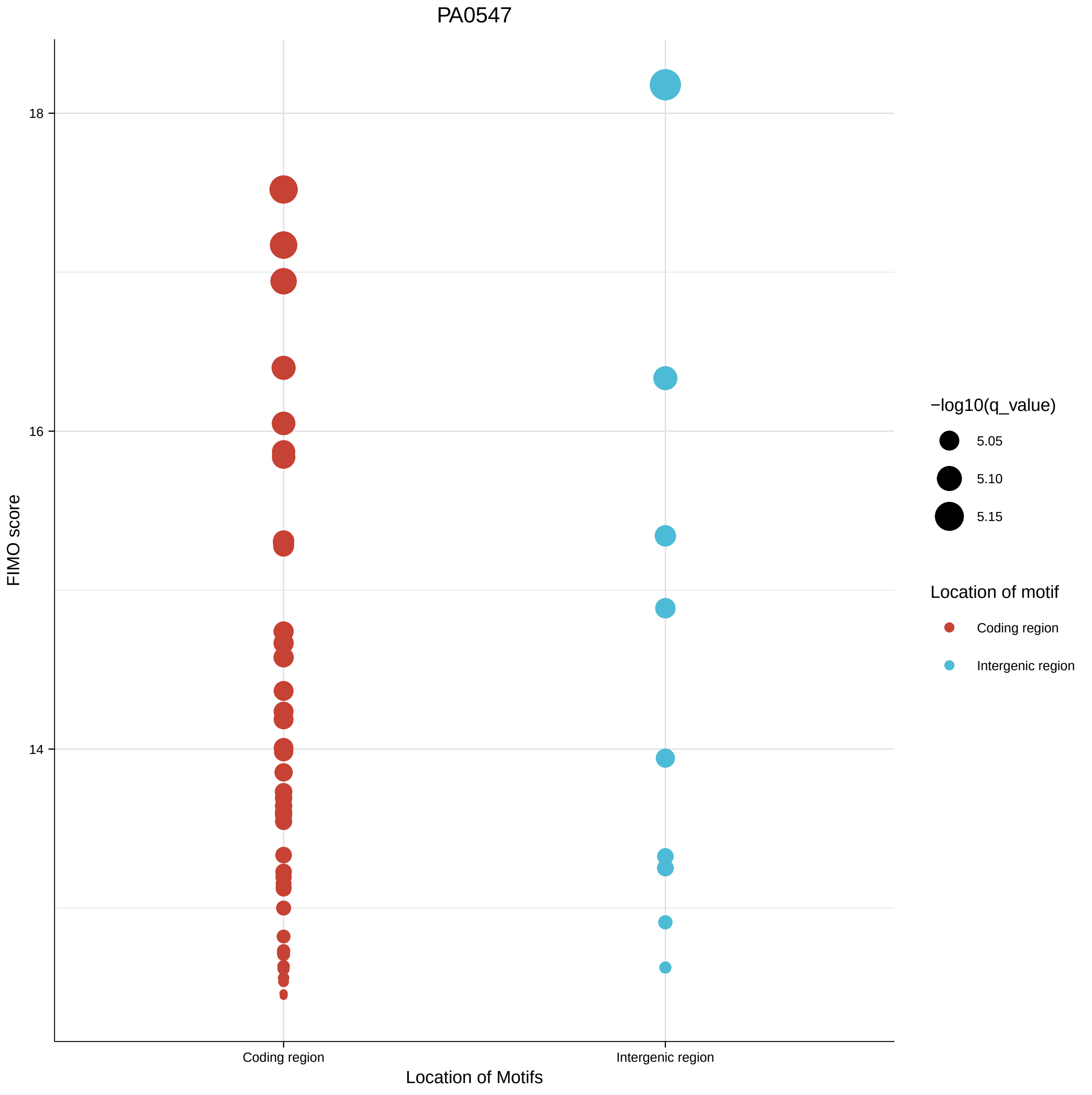
PA0547
FIMO score
18
16
14
Coding region
Intergenic region
Location of Motifs
−log10(q_value)
5.05
5.10
5.15
Location of motif
Coding region
Intergenic region

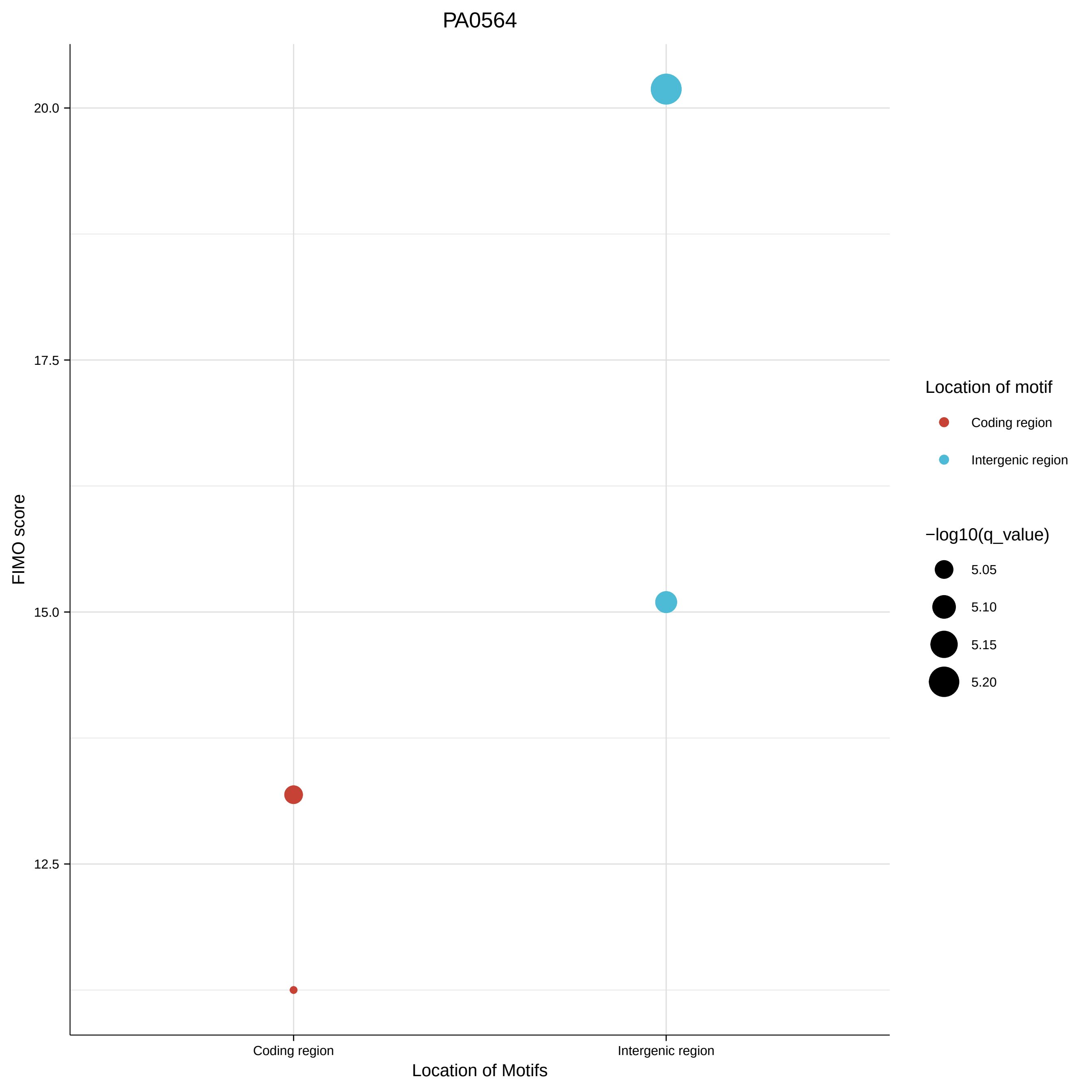
PA0564
FIMO score
20.0
17.5
15.0
12.5
Coding region
Intergenic region
Location of Motifs
Location of motif
Coding region
Intergenic region
−log10(q_value)
5.05
5.10
5.15
5.20

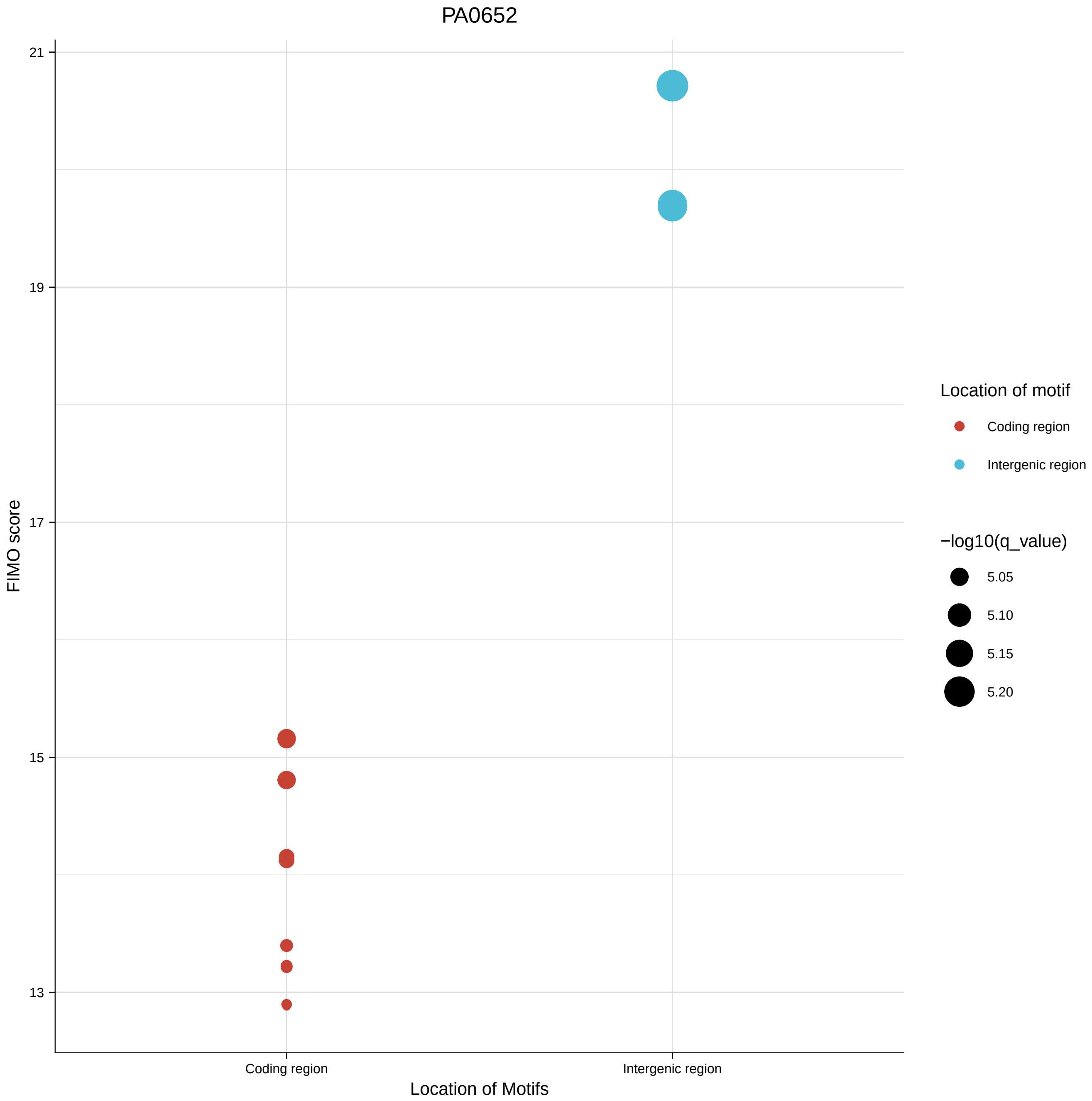

PA0652
FIMO score
21
19
17
15
13
Coding region
Intergenic region
Location of Motifs
Location of motif
Coding region
Intergenic region
−log10(q_value)
5.05
5.10
5.15
5.20

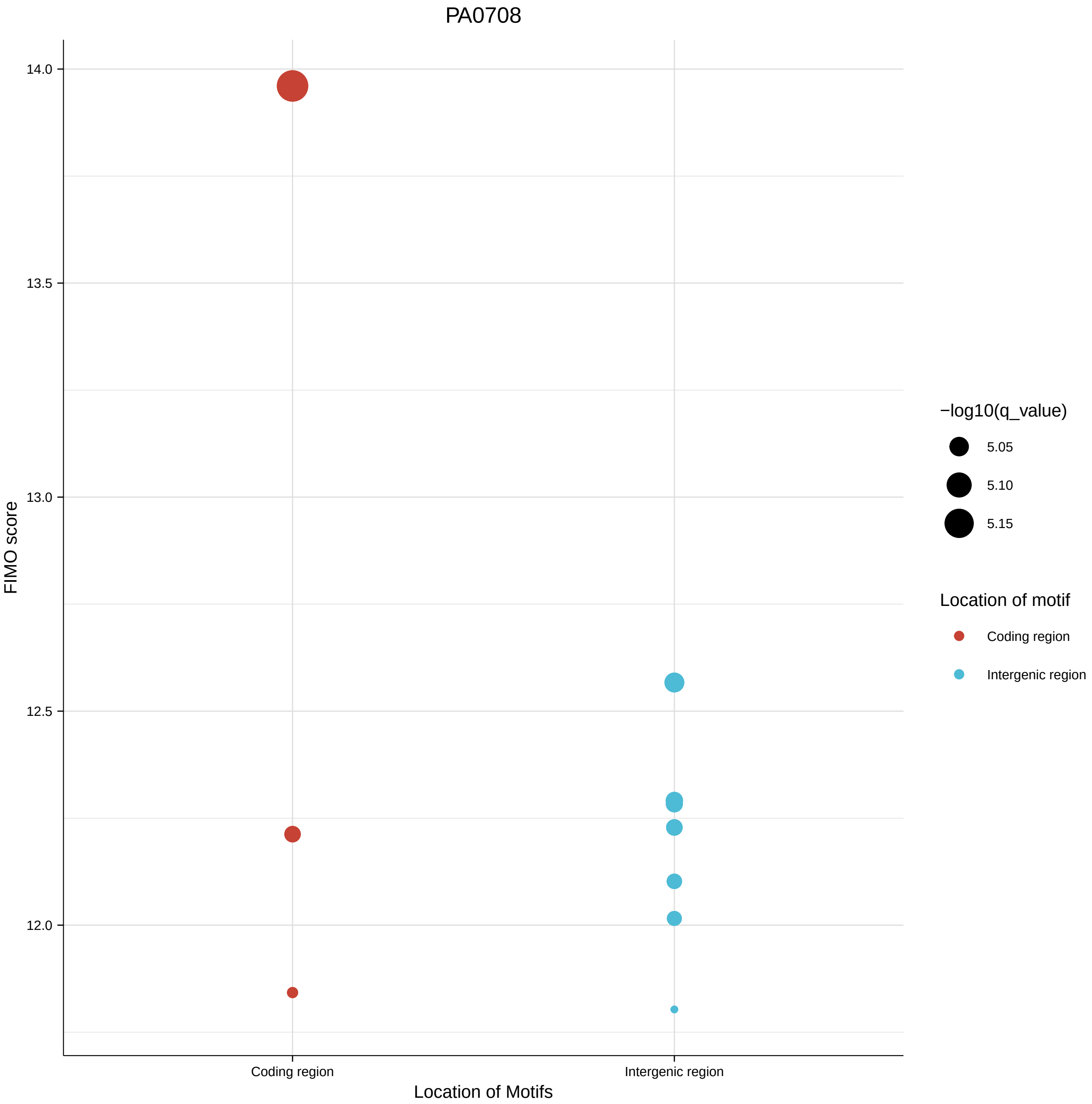
PA0708
FIMO score
14.0
13.5
13.0
12.5
12.0
Coding region
Intergenic region
Location of Motifs
−log10(q_value)
5.05
5.10
5.15
Location of motif
Coding region
Intergenic region

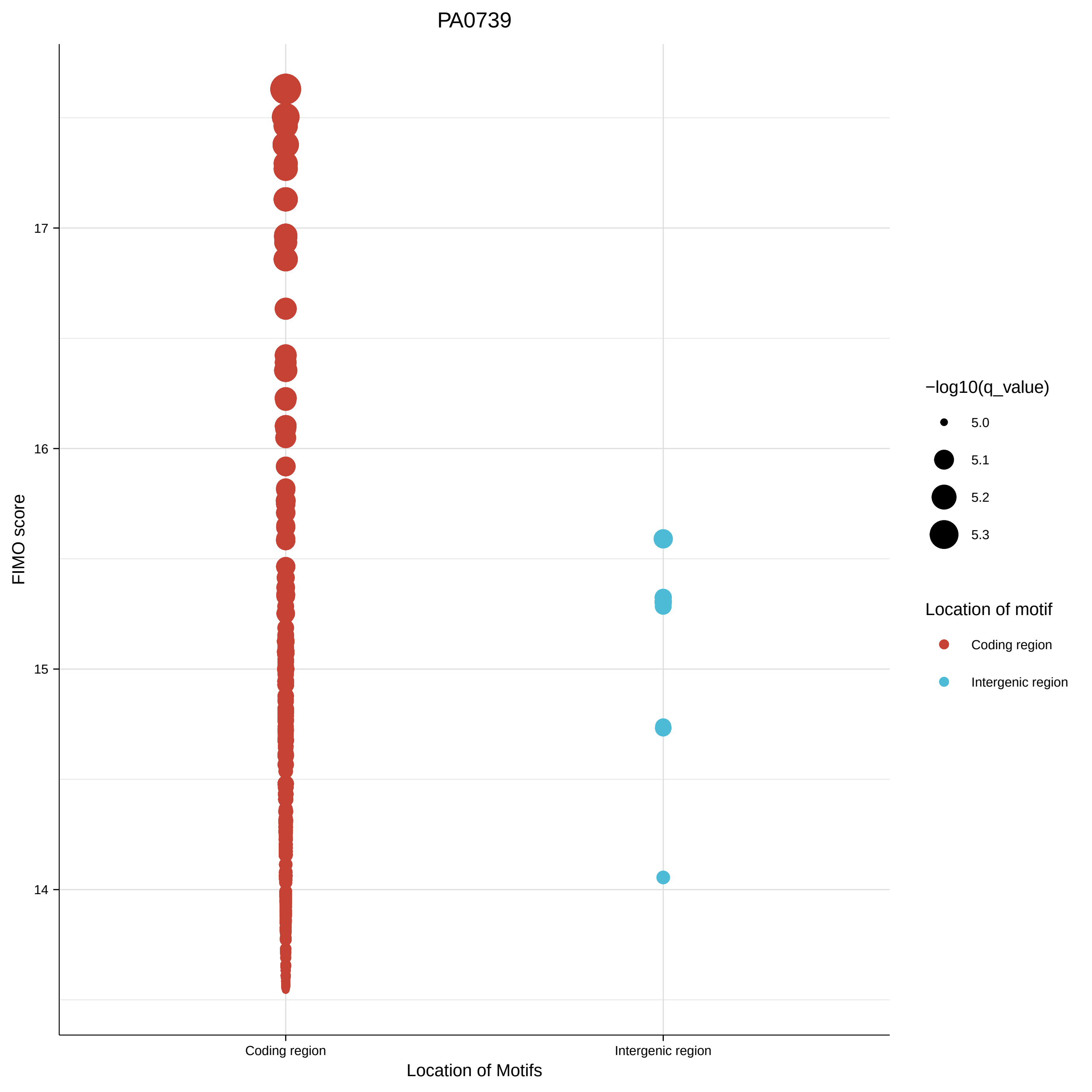
PA0739
FIMO score
17
16
15
14
Coding region
Intergenic region
Location of Motifs
−log10(q_value)
5.0
5.1
5.2
5.3
Location of motif
Coding region
Intergenic region

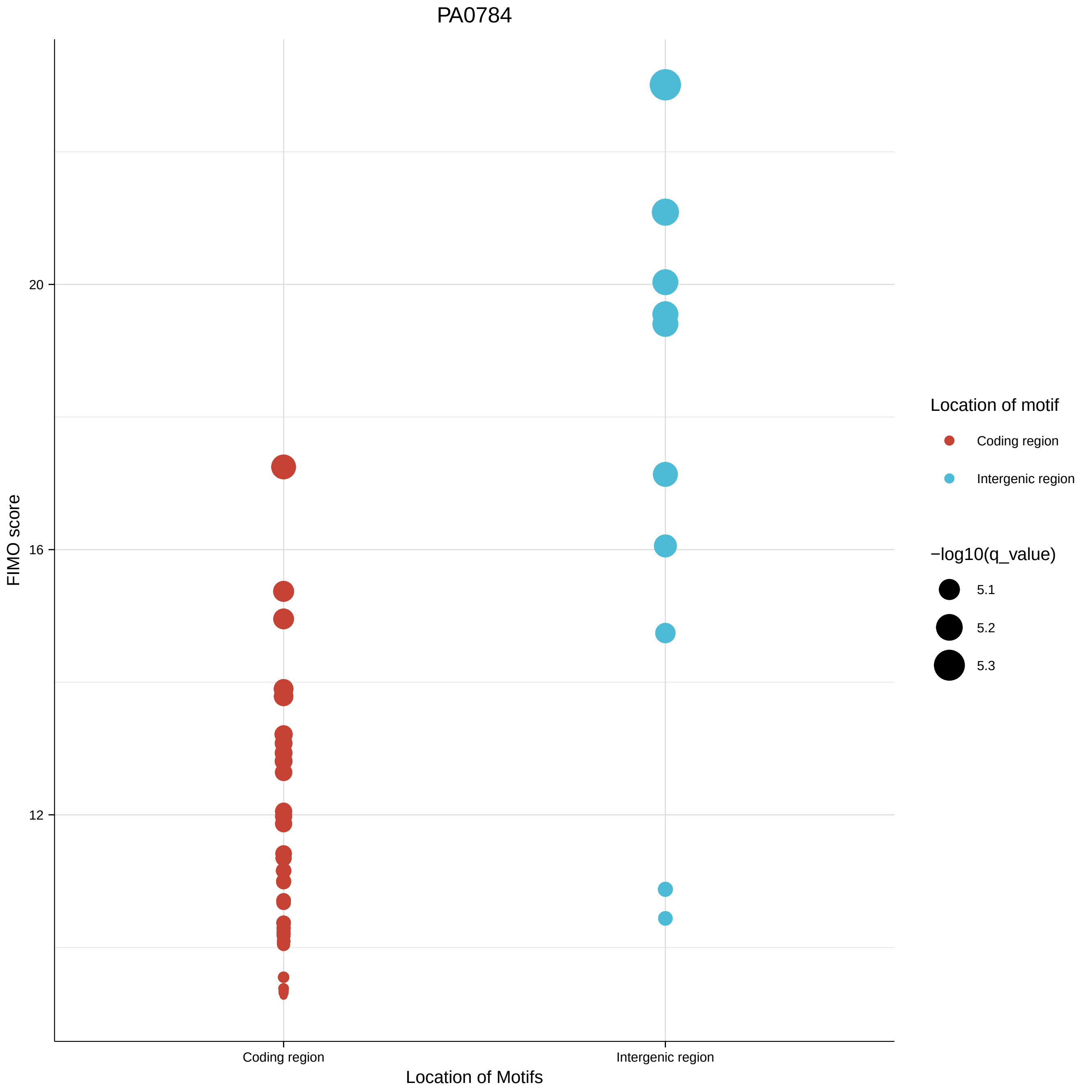
PA0784
FIMO score
20
16
12
Coding region
Intergenic region
Location of Motifs
Location of motif
Coding region
Intergenic region
−log10(q_value)
5.1
5.2
5.3

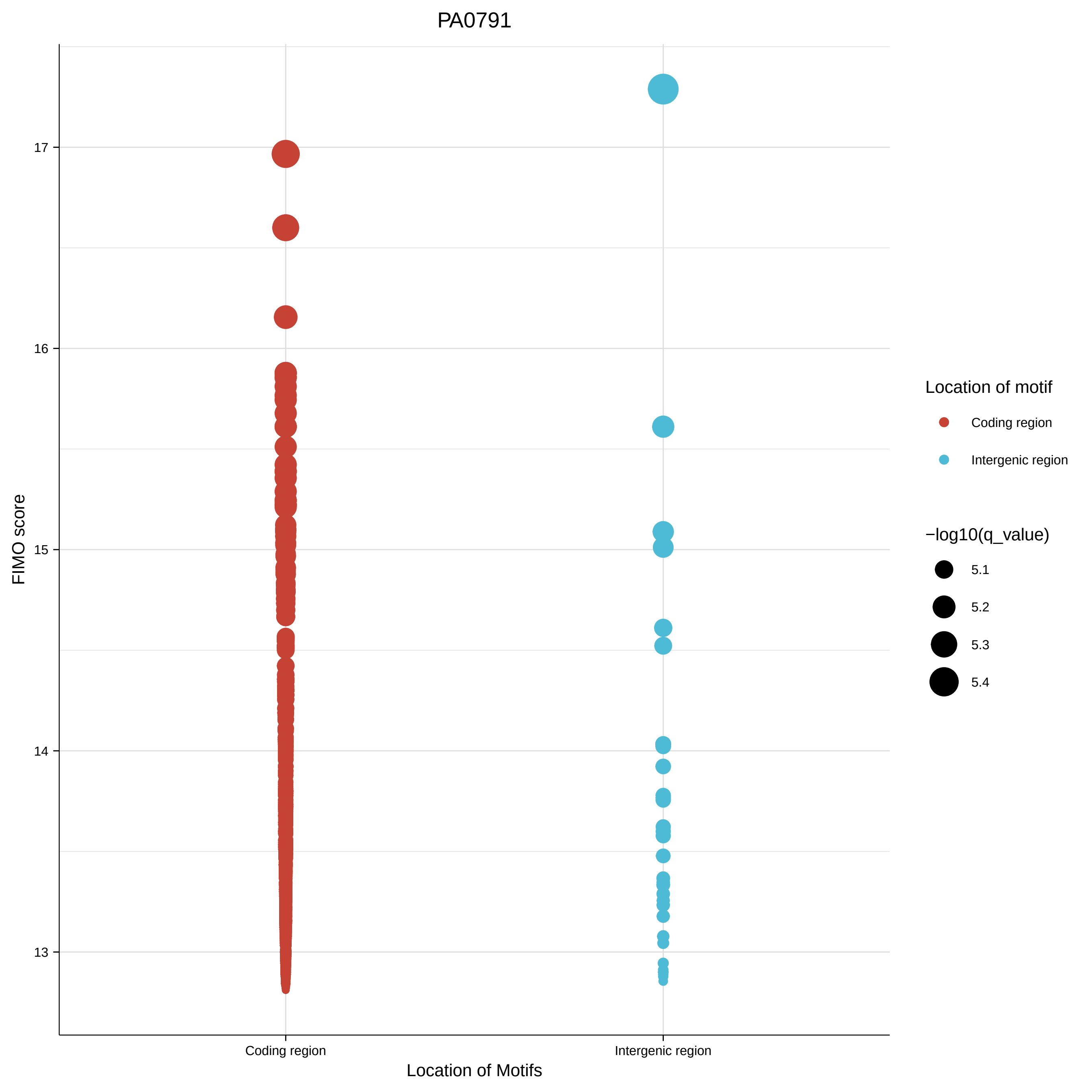
PA0791
FIMO score
17
16
15
14
13
Coding region
Intergenic region
Location of Motifs
Location of motif
Coding region
Intergenic region
−log10(q_value)
5.1
5.2
5.3
5.4

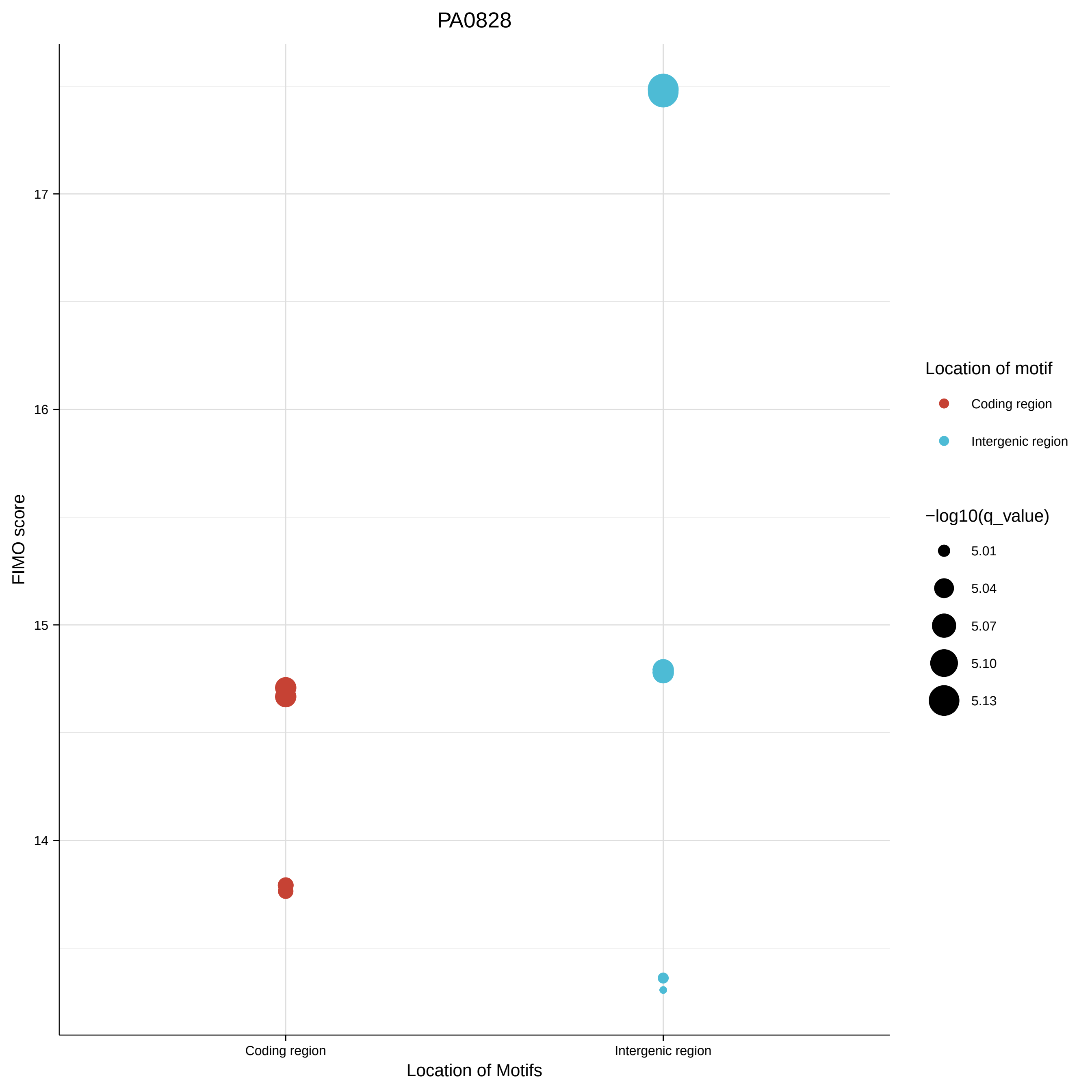

PA0828
FIMO score
17
16
15
14
Coding region
Intergenic region
Location of Motifs
Location of motif
Coding region
Intergenic region
−log10(q_value)
5.01
5.04
5.07
5.10
5.13

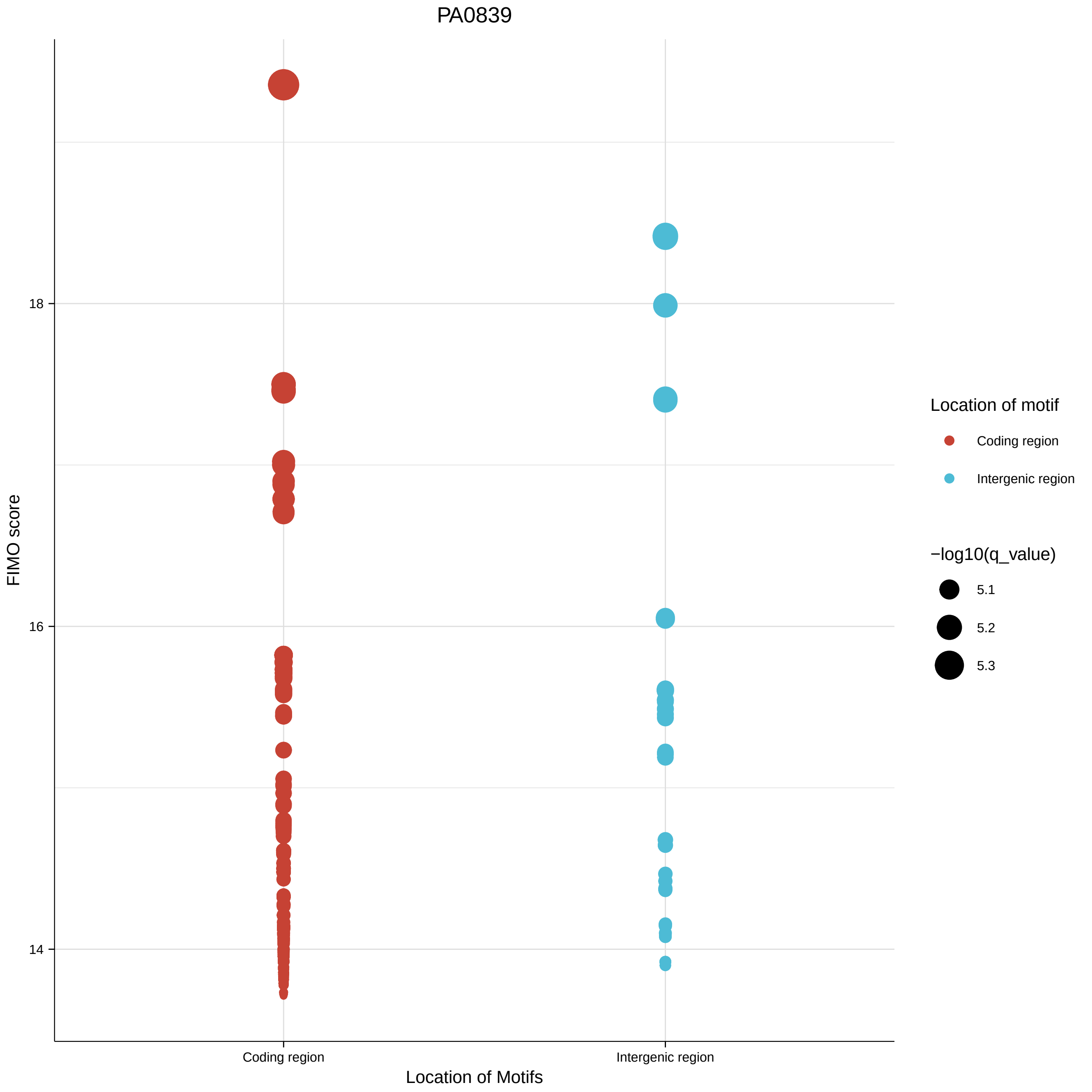

PA0839
FIMO score
18
16
14
Coding region
Intergenic region
Location of Motifs
Location of motif
Coding region
Intergenic region
−log10(q_value)
5.1
5.2
5.3

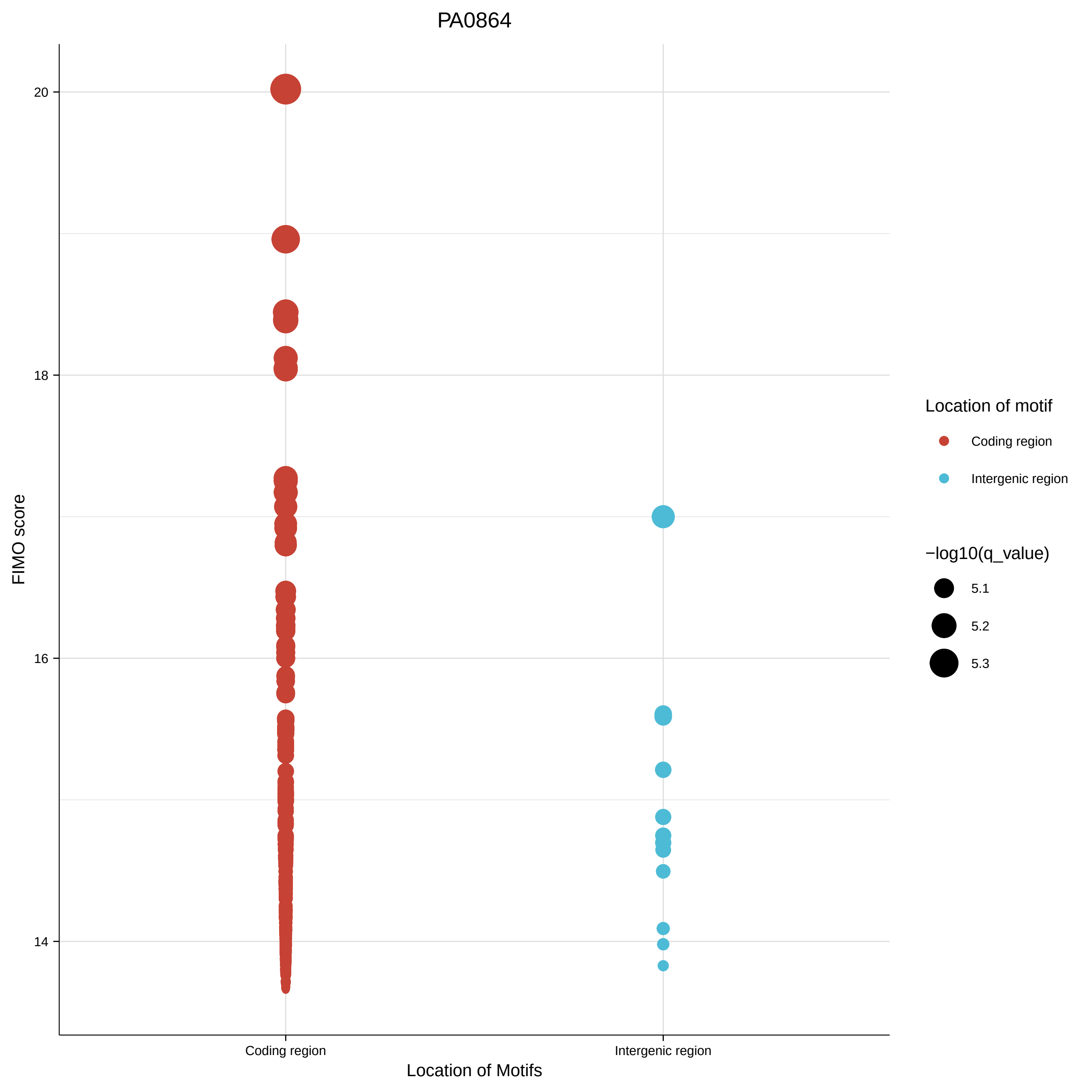

PA0864
FIMO score
20
18
16
14
Coding region
Intergenic region
Location of Motifs
Location of motif
Coding region
Intergenic region
−log10(q_value)
5.1
5.2
5.3

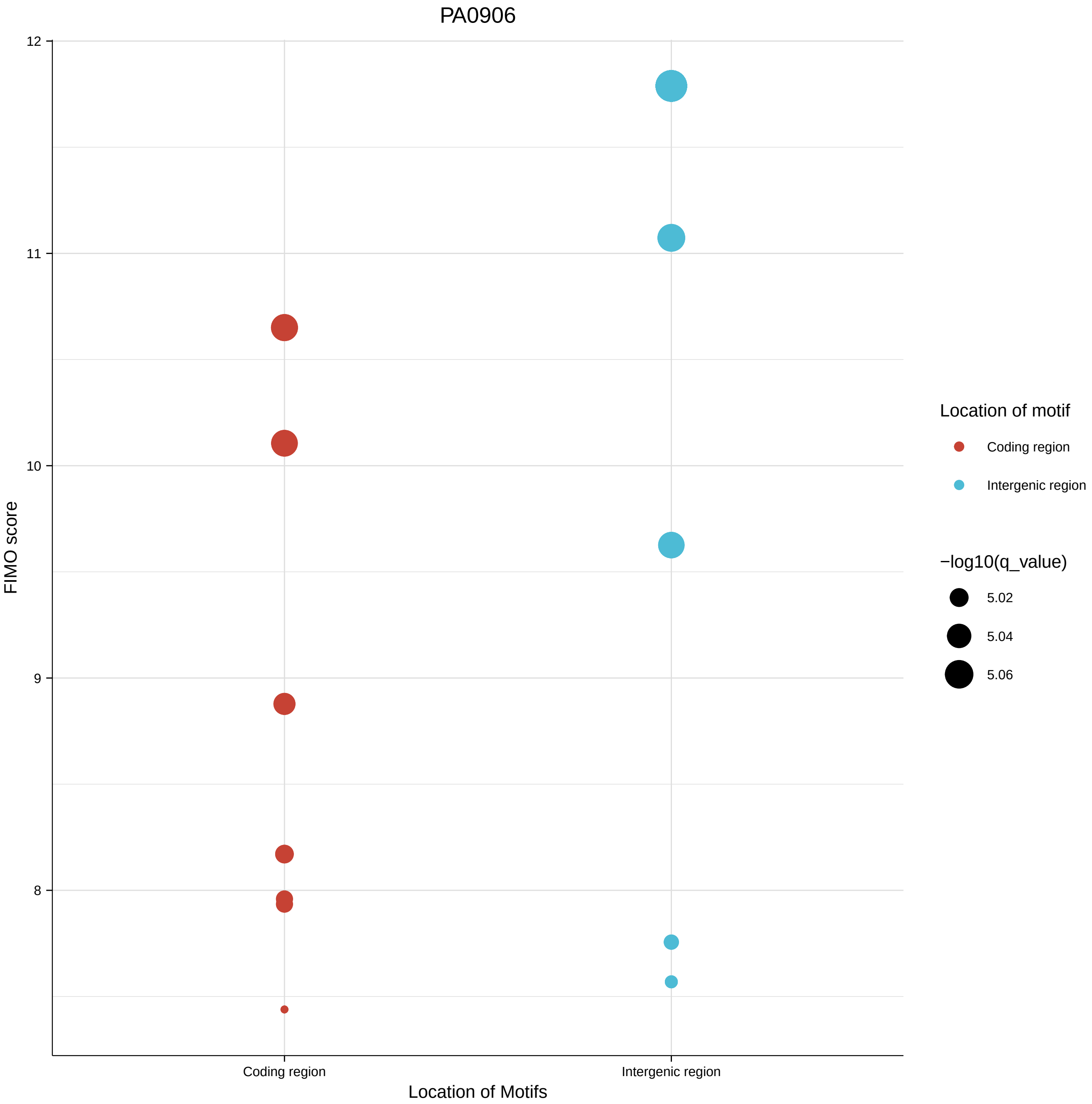
PA0906
FIMO score
12
11
10
9
8
Coding region
Intergenic region
Location of Motifs
Location of motif
Coding region
Intergenic region
−log10(q_value)
5.02
5.04
5.06

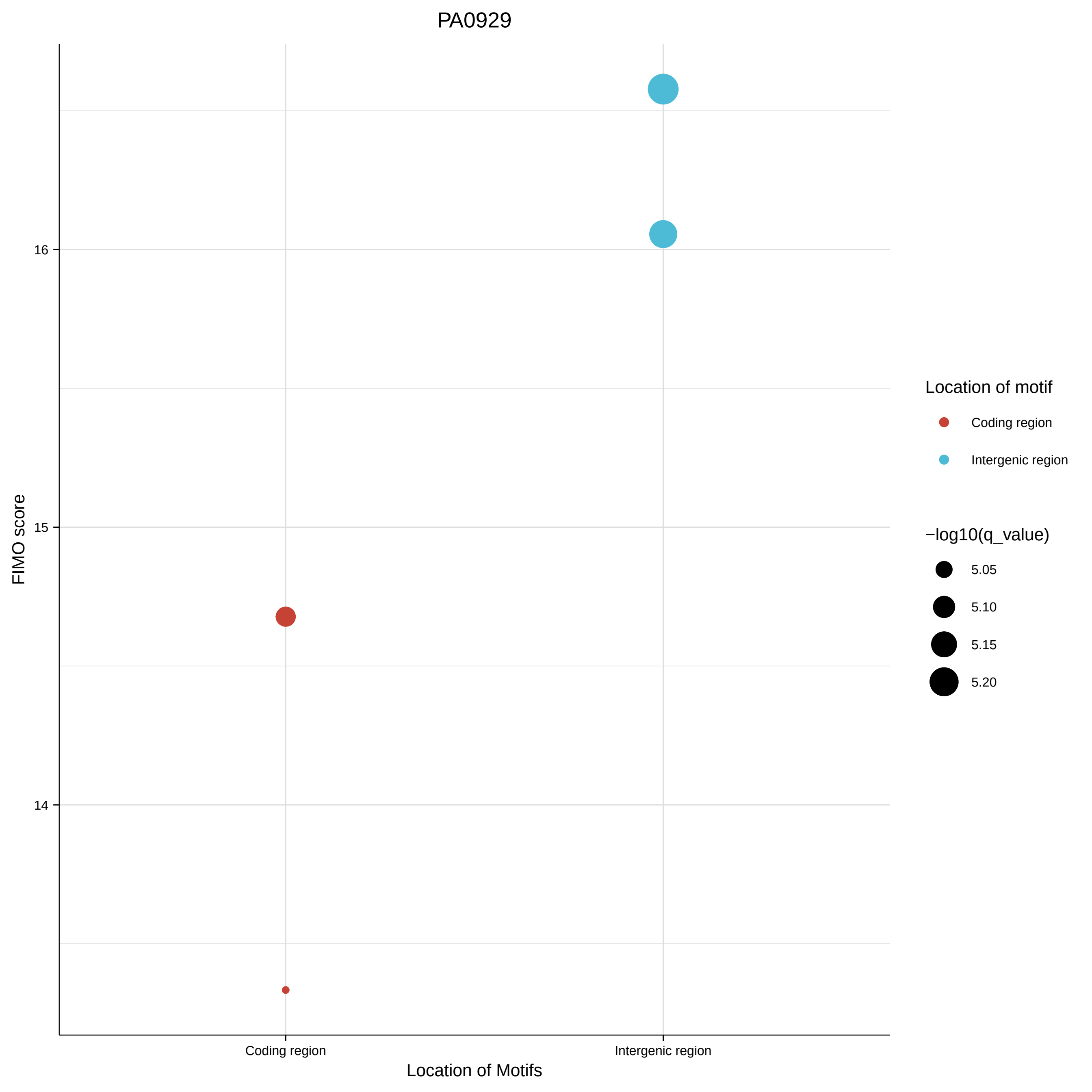
PA0929
FIMO score
16
15
14
Coding region
Intergenic region
Location of Motifs
Location of motif
Coding region
Intergenic region
−log10(q_value)
5.05
5.10
5.15
5.20

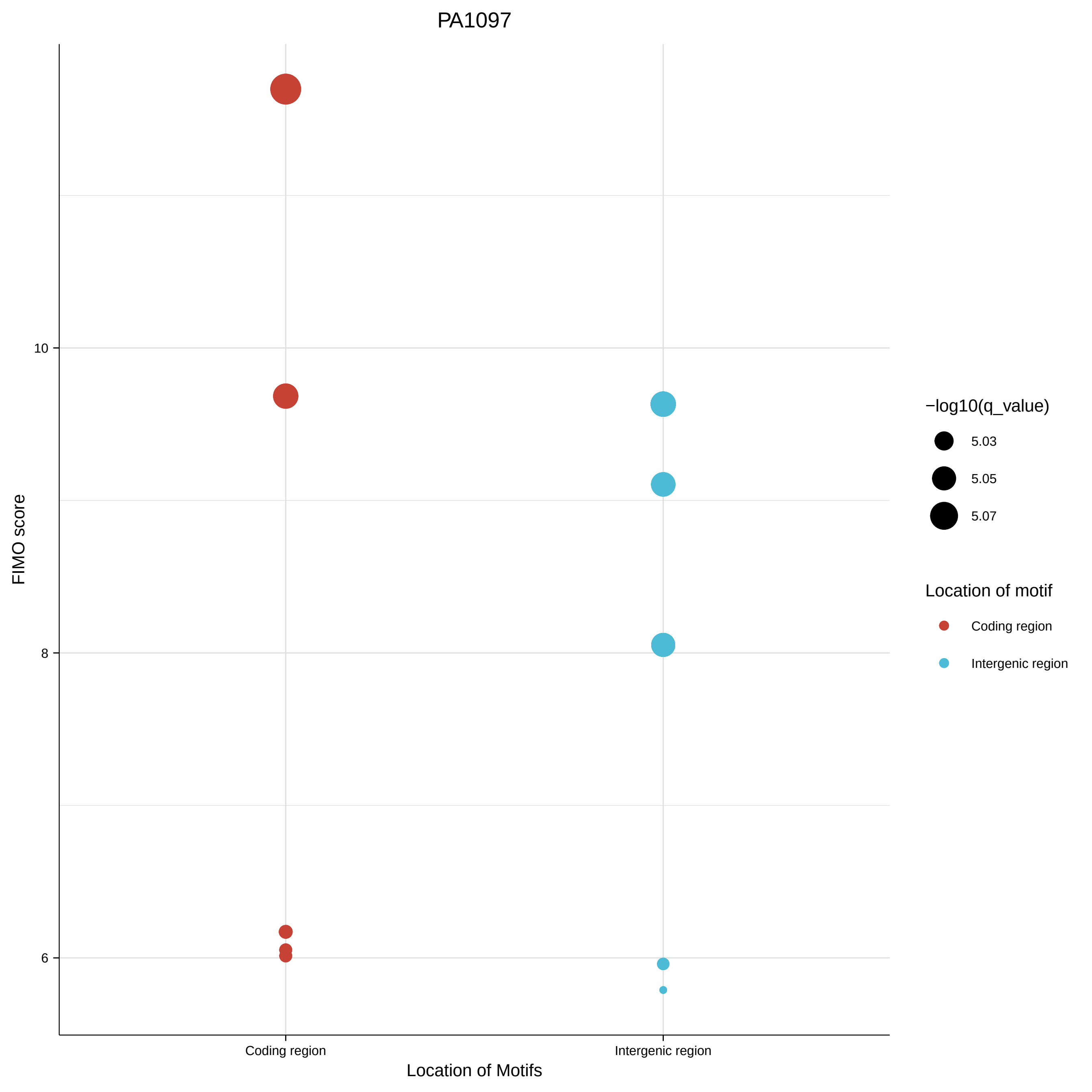

PA1097
FIMO score
10
8
6
Coding region
Intergenic region
Location of Motifs
−log10(q_value)
5.03
5.05
5.07
Location of motif
Coding region
Intergenic region

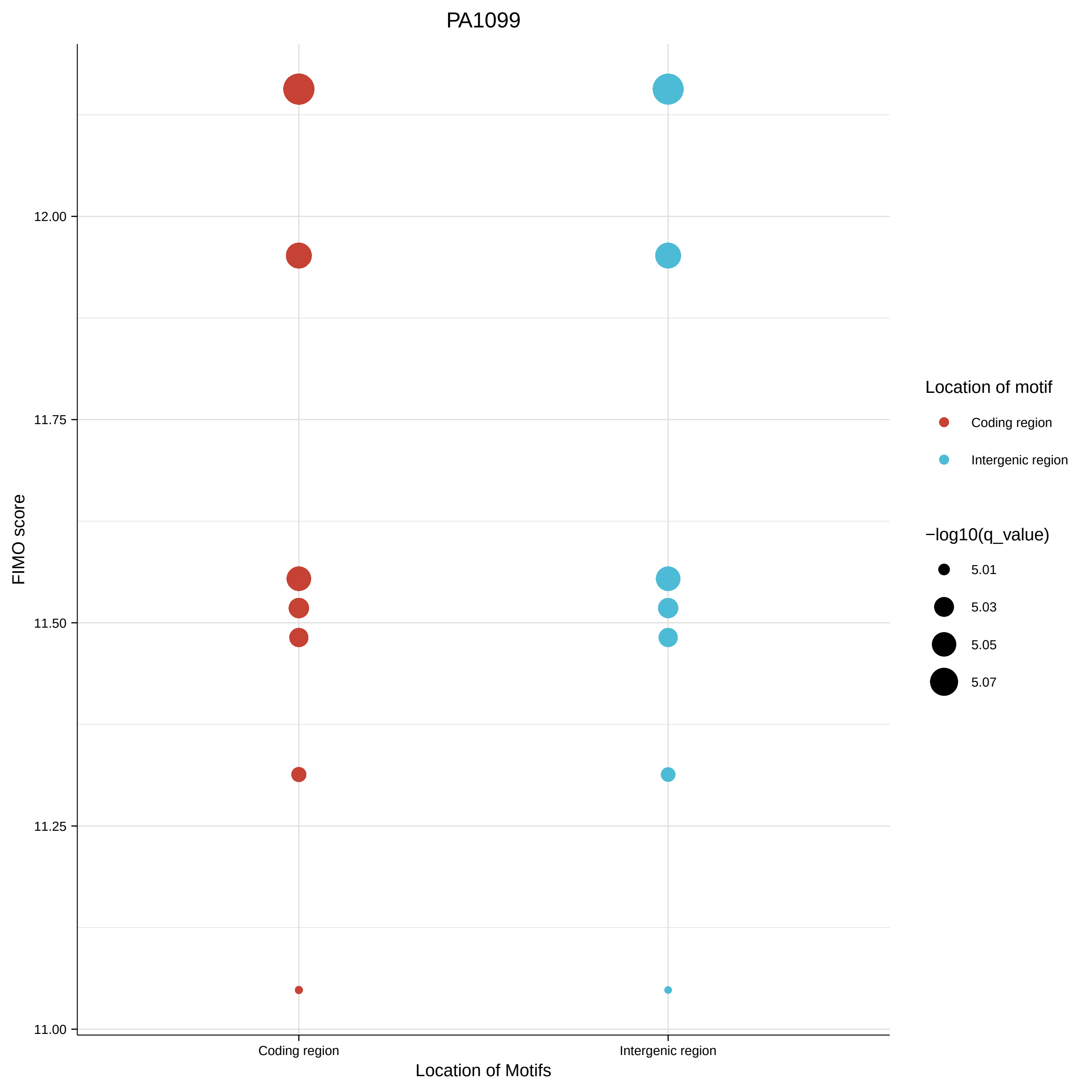
PA1099
FIMO score
12.00
11.75
11.50
11.25
11.00
Coding region
Intergenic region
Location of Motifs
Location of motif
Coding region
Intergenic region
−log10(q_value)
5.01
5.03
5.05
5.07

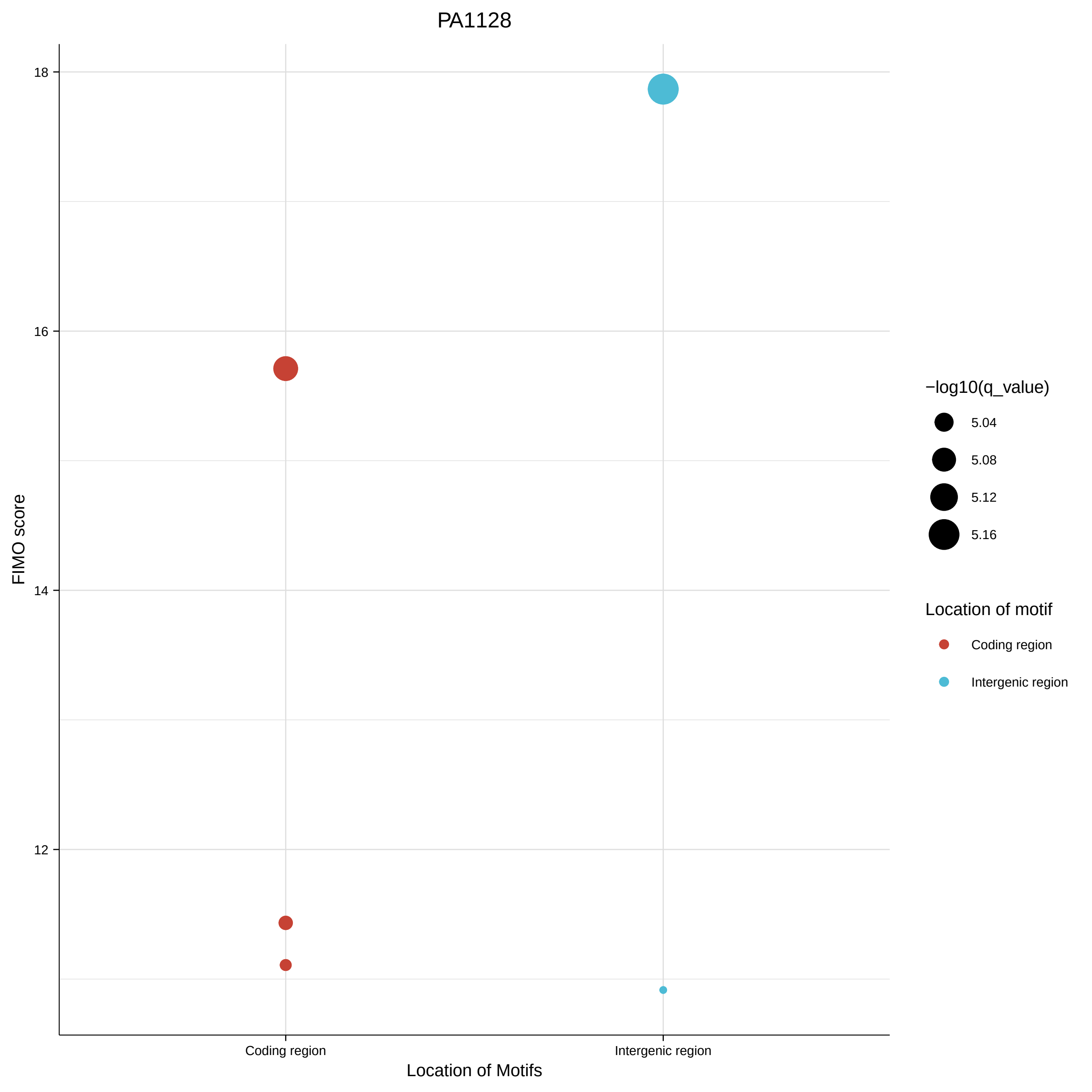
PA1128
FIMO score
18
16
14
12
Coding region
Intergenic region
Location of Motifs
−log10(q_value)
5.04
5.08
5.12
5.16
Location of motif
Coding region
Intergenic region

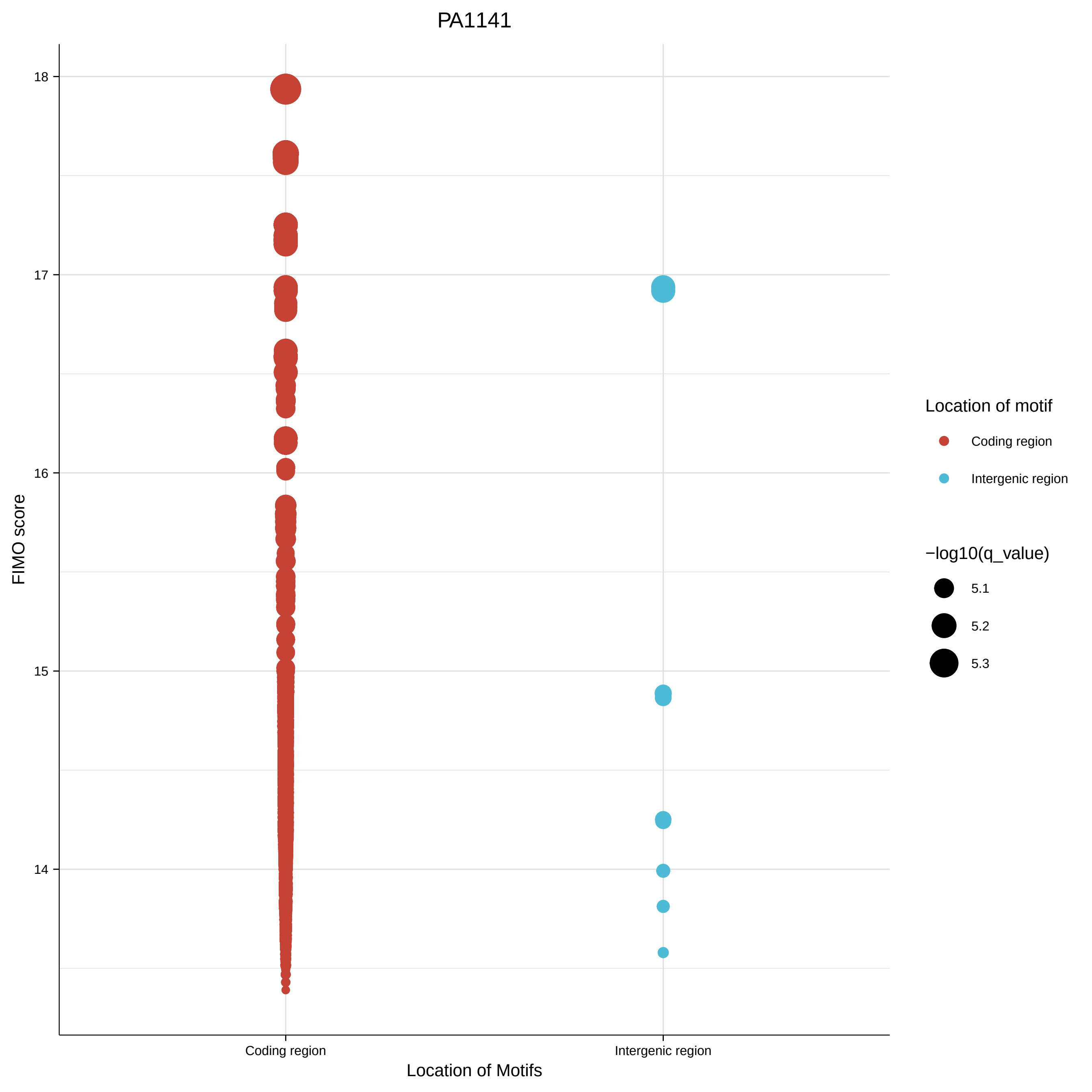
PA1141
FIMO score
18
17
16
15
14
Coding region
Intergenic region
Location of Motifs
Location of motif
Coding region
Intergenic region
−log10(q_value)
5.1
5.2
5.3

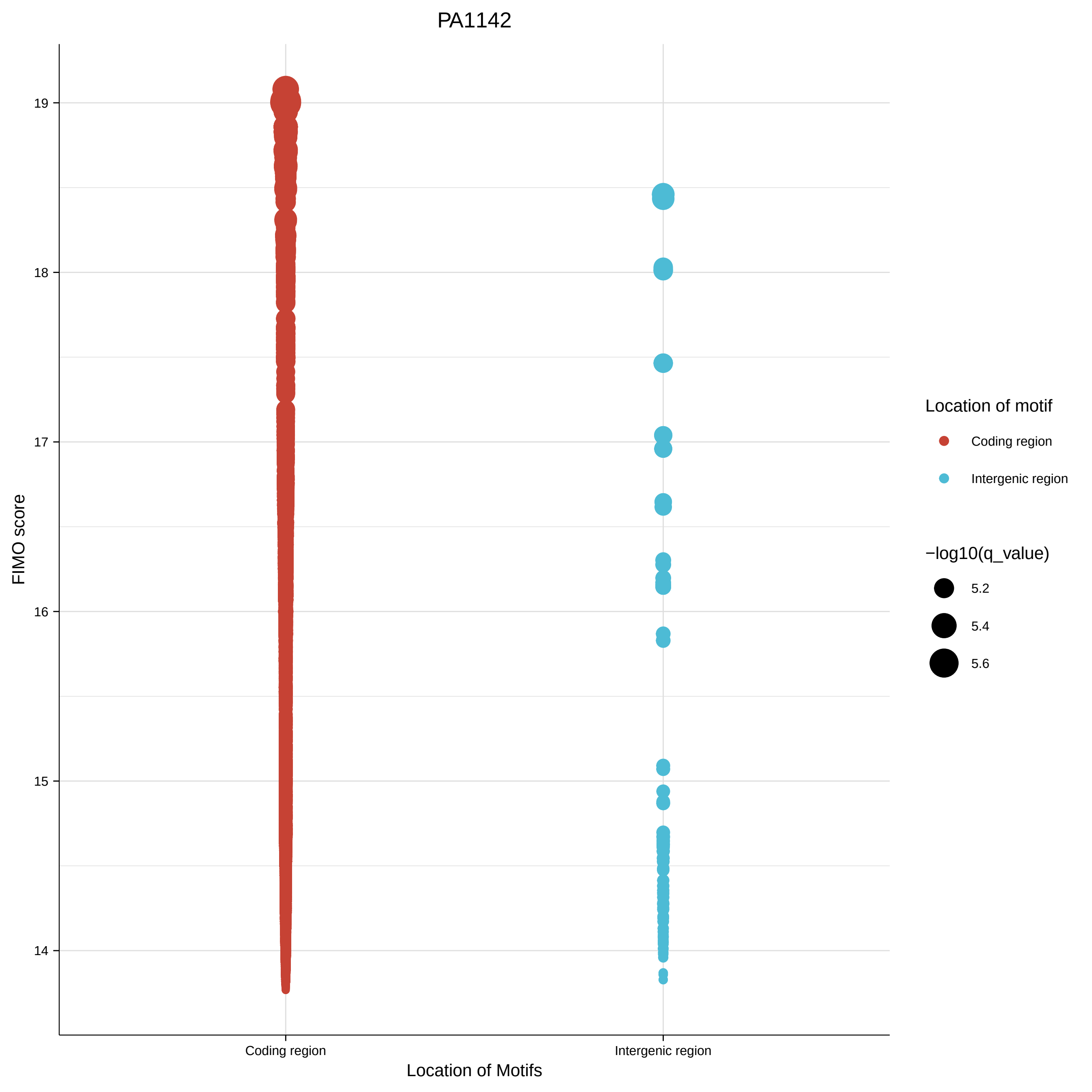
PA1142
FIMO score
19
18
17
16
15
14
Coding region
Intergenic region
Location of Motifs
Location of motif
Coding region
Intergenic region
−log10(q_value)
5.2
5.4
5.6

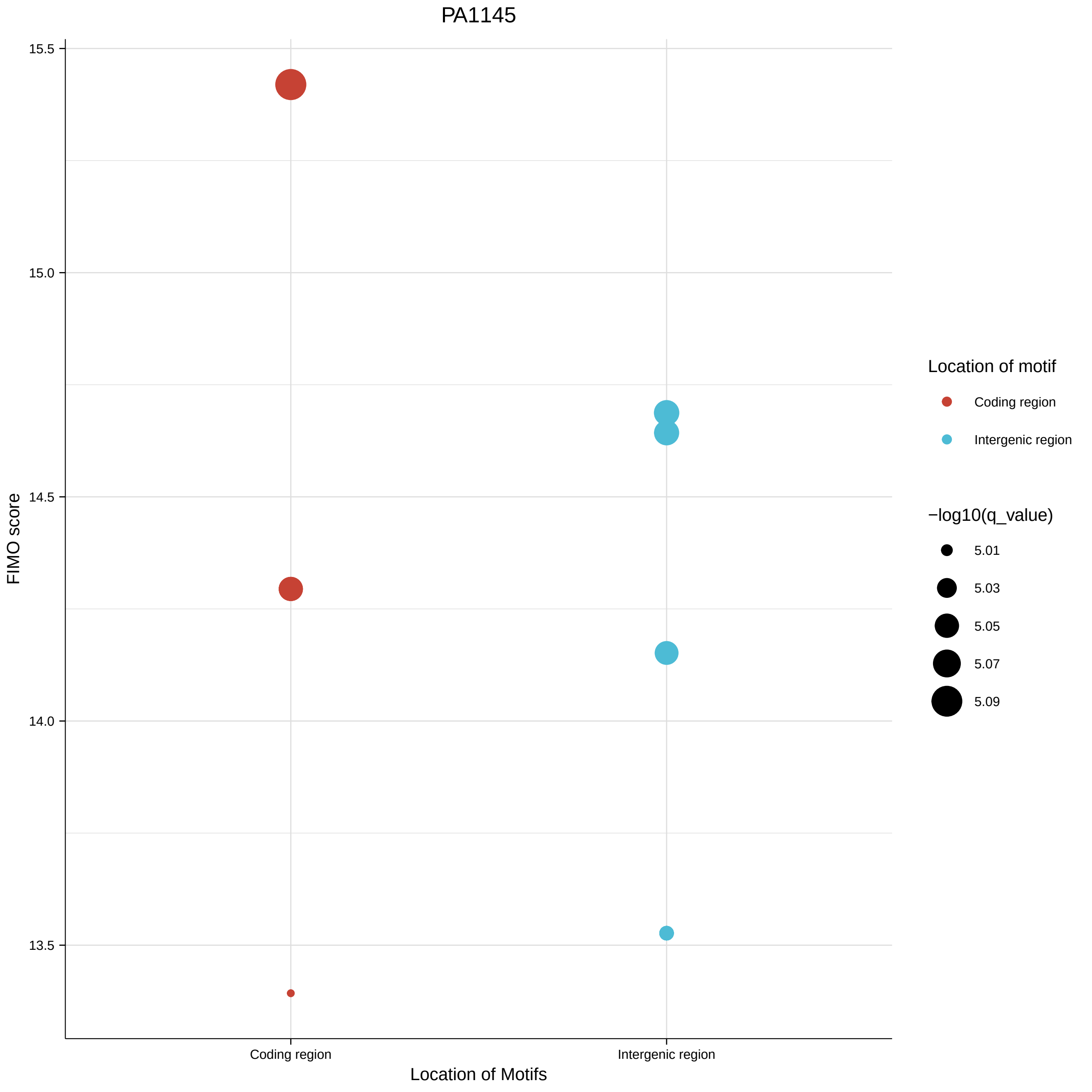
PA1145
FIMO score
15.5
15.0
14.5
14.0
13.5
Coding region
Intergenic region
Location of Motifs
Location of motif
Coding region
Intergenic region
−log10(q_value)
5.01
5.03
5.05
5.07
5.09

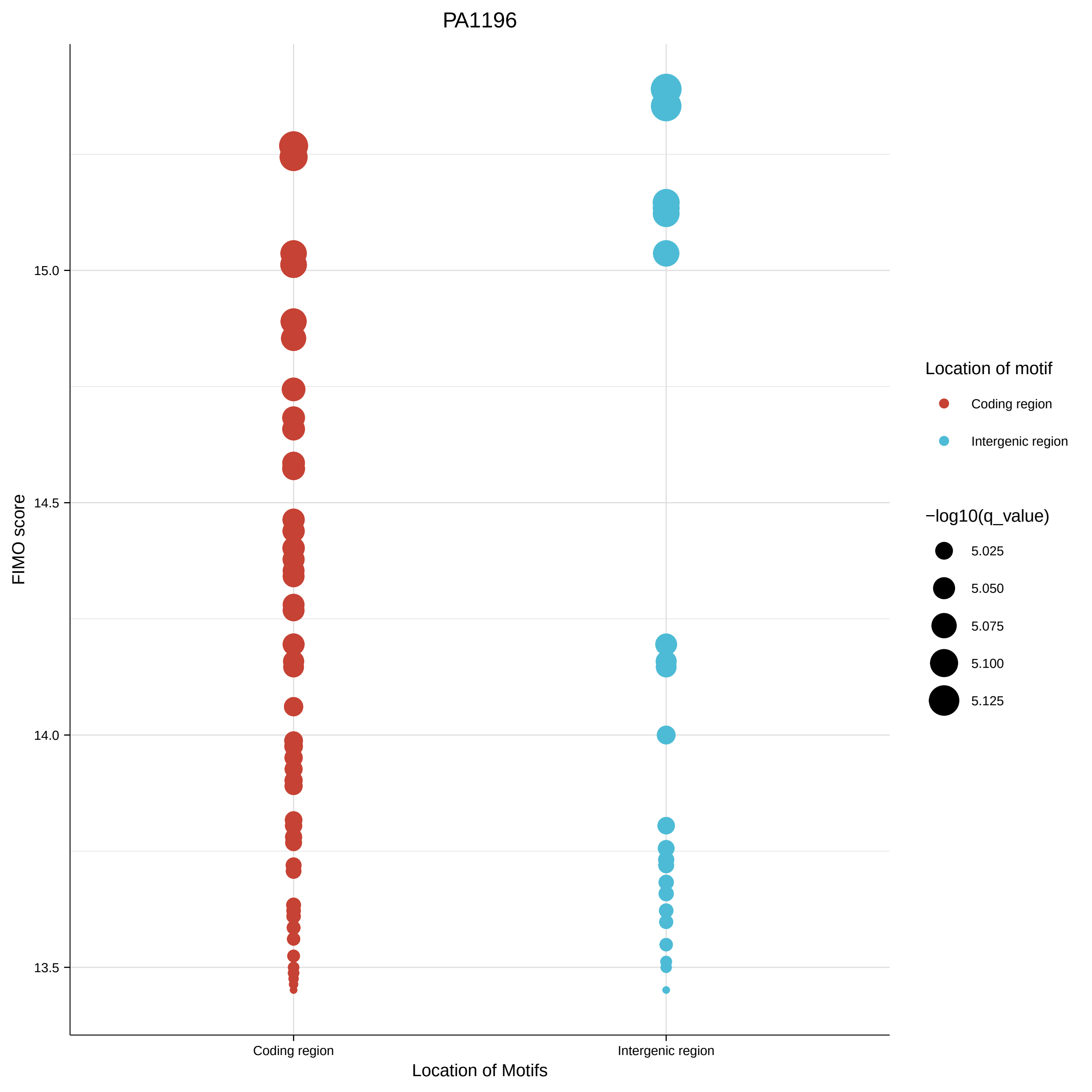
PA1196
FIMO score
15.0
14.5
14.0
13.5
Coding region
Intergenic region
Location of Motifs
Location of motif
Coding region
Intergenic region
−log10(q_value)
5.025
5.050
5.075
5.100
5.125

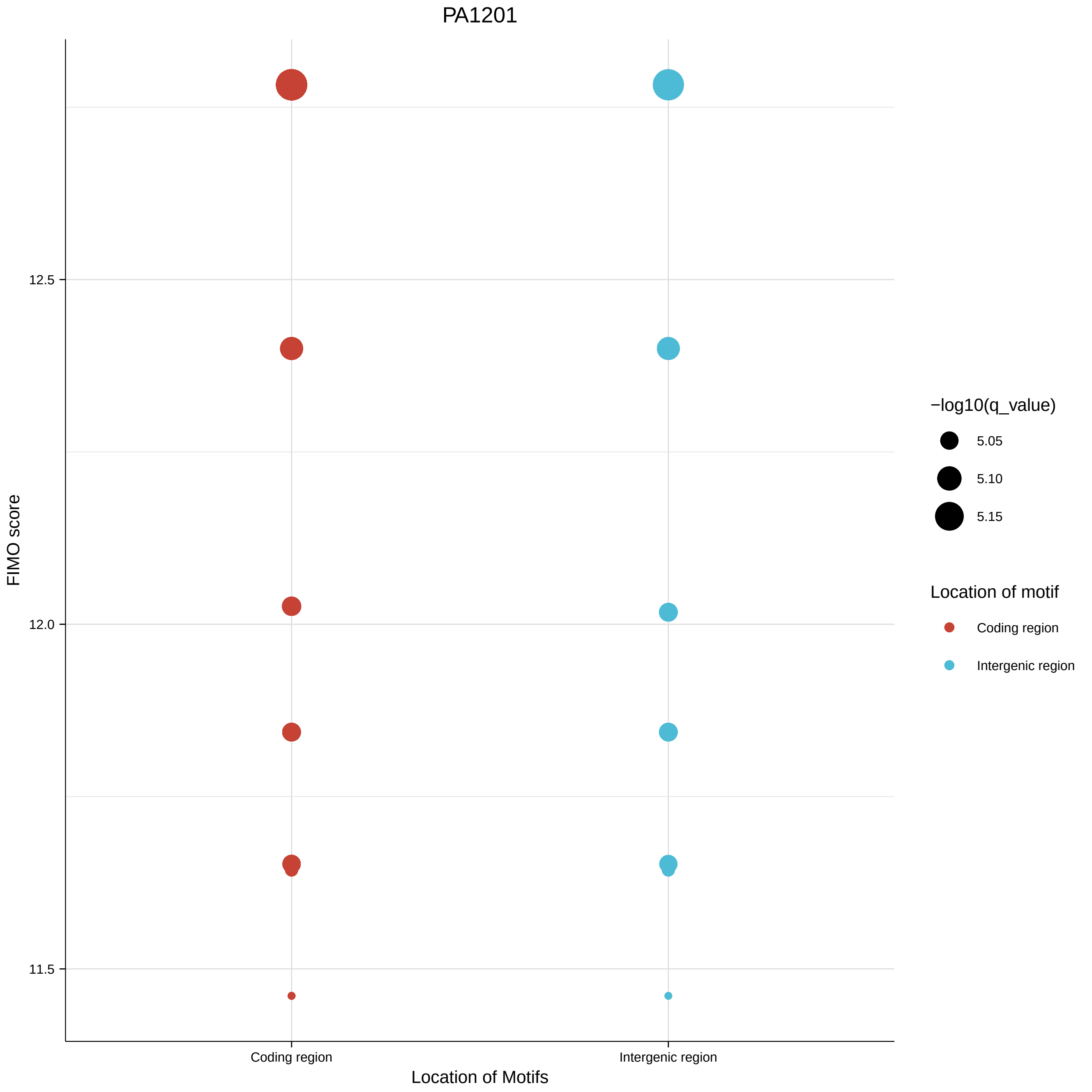
PA1201
12.5
12.0
11.5
FIMO score
Coding region
Intergenic region
Location of Motifs
−log10(q_value)
5.05
5.10
5.15
Location of motif
Coding region
Intergenic region

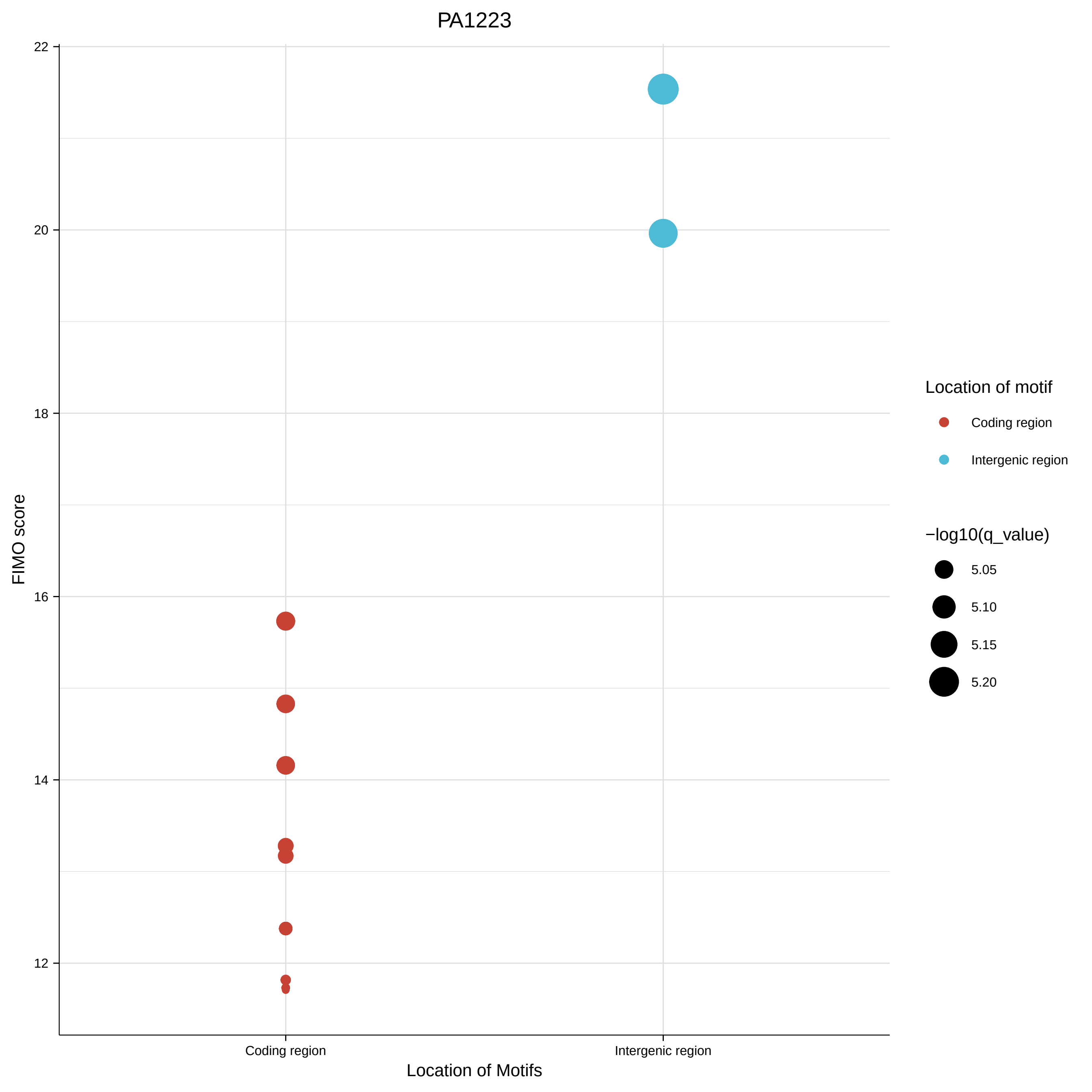

PA1223
FIMO score
22
20
18
16
14
12
Coding region
Intergenic region
Location of Motifs
Location of motif
Coding region
Intergenic region
−log10(q_value)
5.05
5.10
5.15
5.20

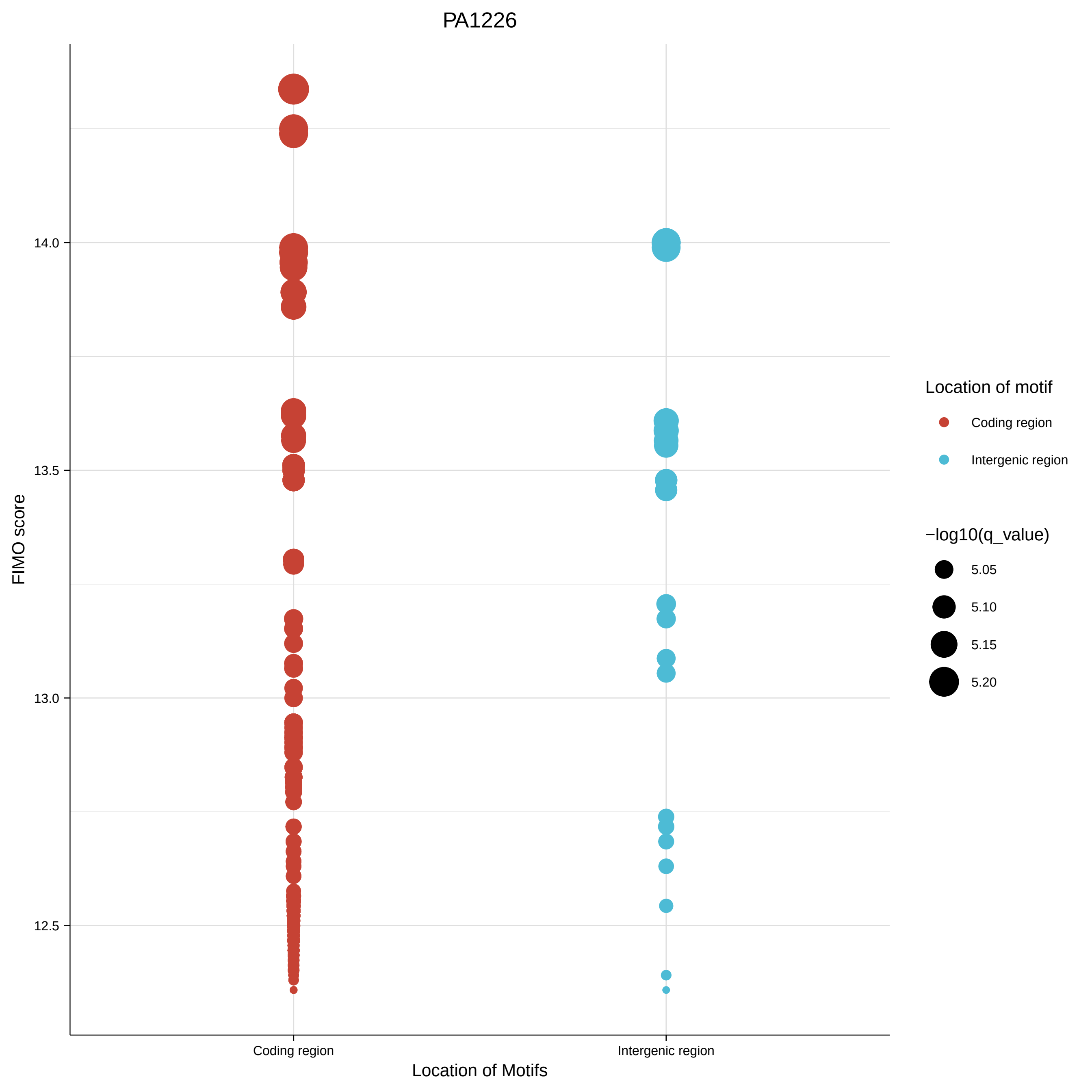

PA1226
FIMO score
14.0
13.5
13.0
12.5
Coding region
Intergenic region
Location of Motifs
Location of motif
Coding region
Intergenic region
−log10(q_value)
5.05
5.10
5.15
5.20

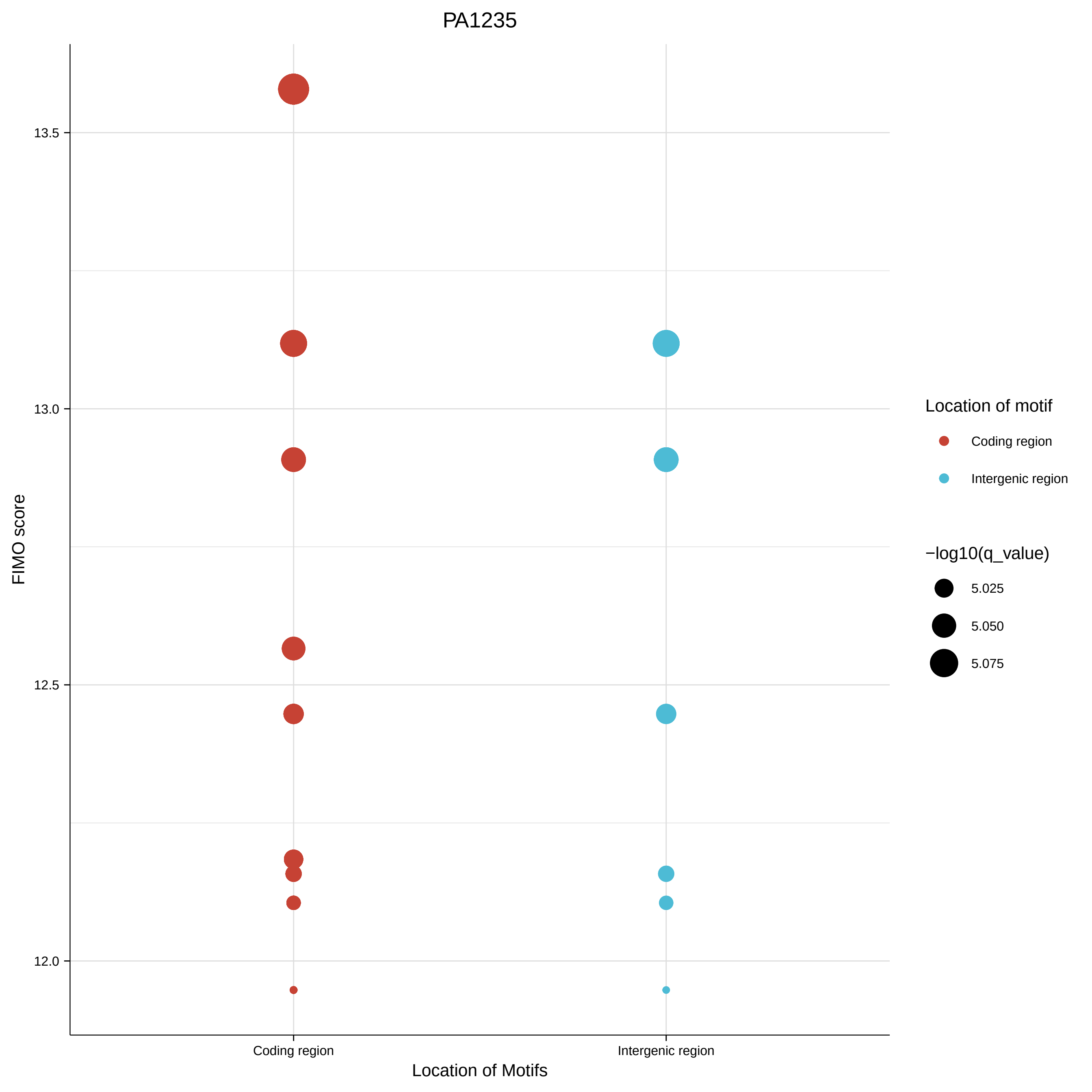
PA1235
FIMO score
13.5
13.0
12.5
12.0
Coding region
Intergenic region
Location of Motifs
Location of motif
Coding region
Intergenic region
−log10(q_value)
5.025
5.050
5.075

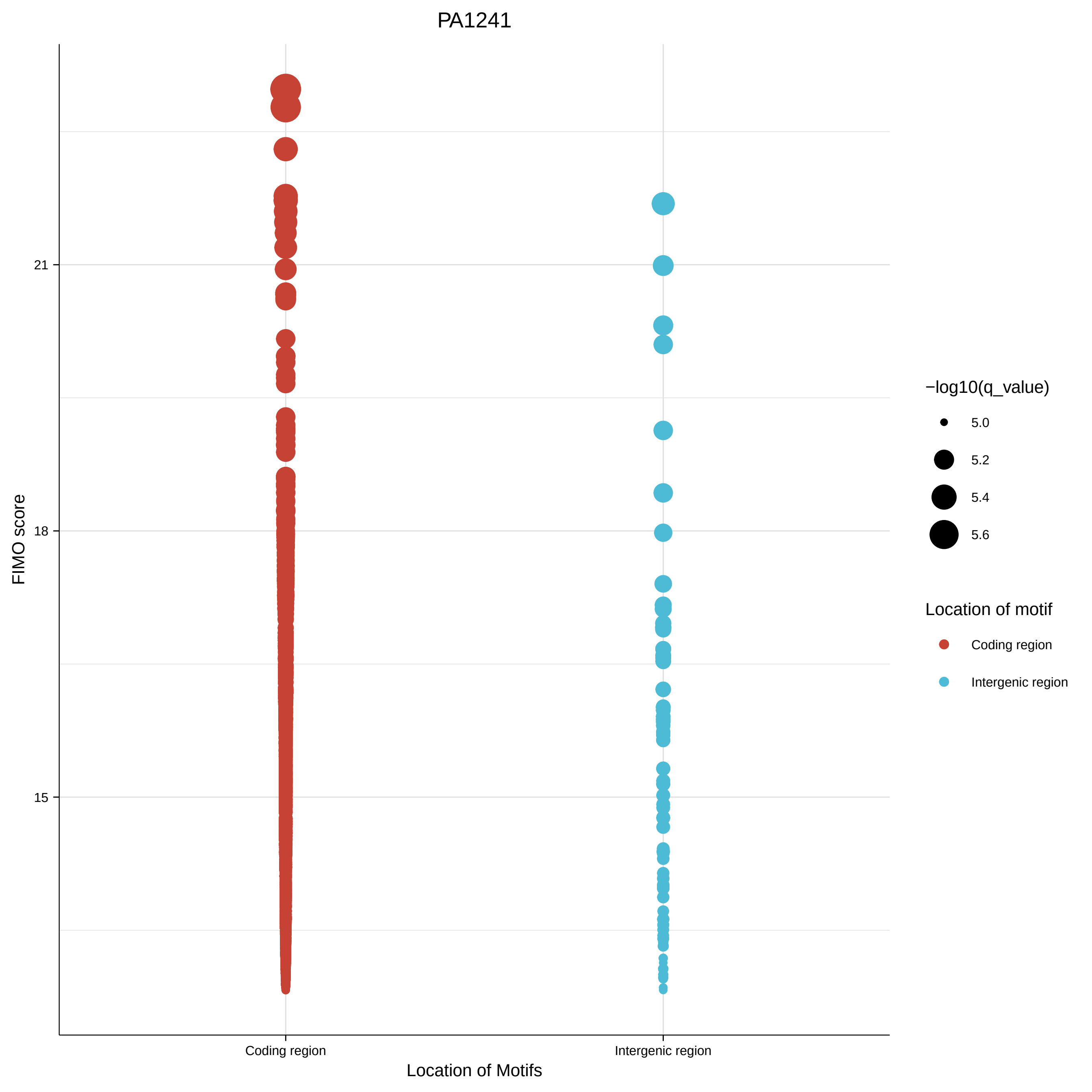
PA1241
FIMO score
21
18
15
Coding region
Intergenic region
Location of Motifs
−log10(q_value)
5.0
5.2
5.4
5.6
Location of motif
Coding region
Intergenic region

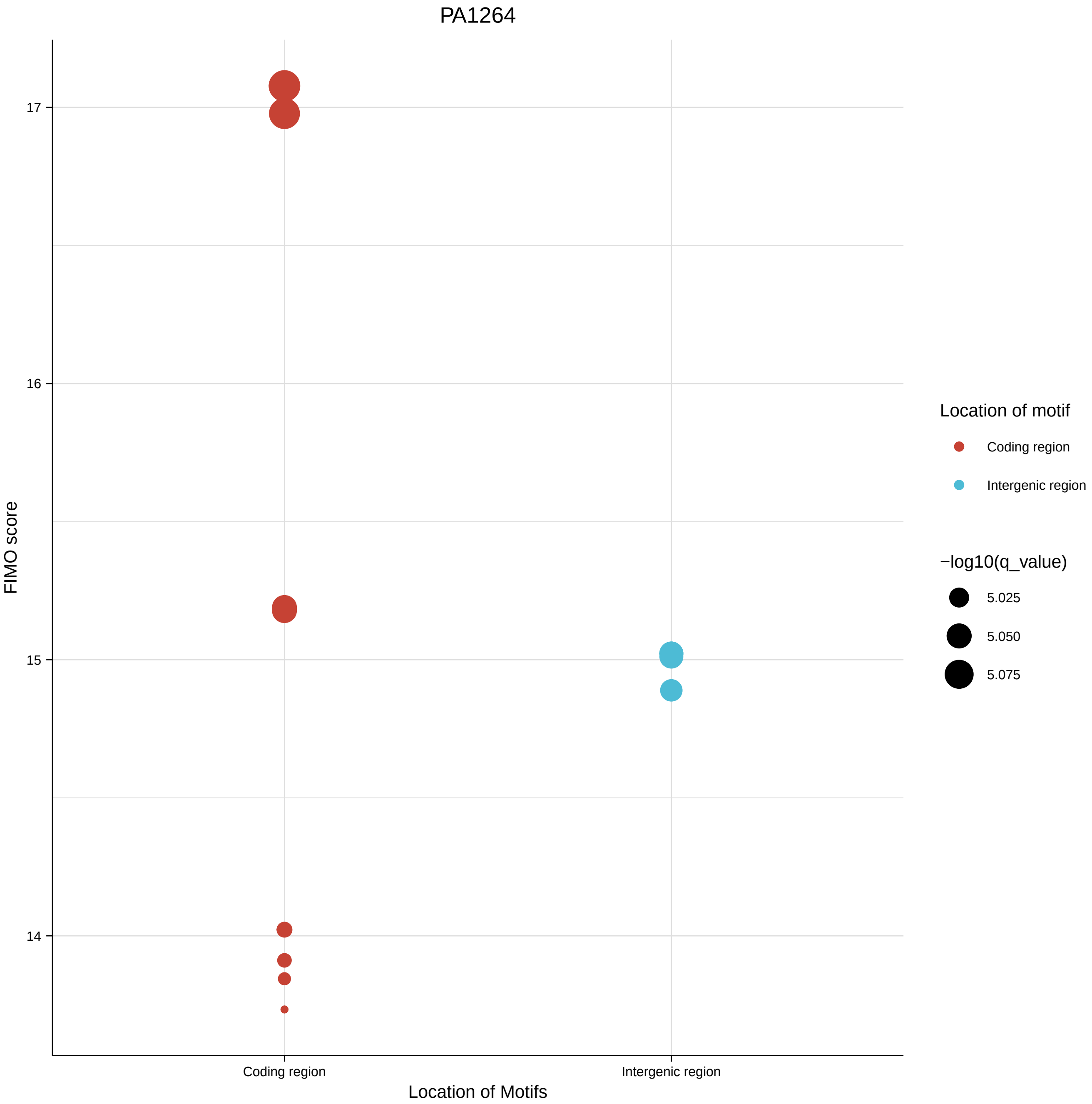

PA1264
FIMO score
17
16
15
14
Coding region
Intergenic region
Location of Motifs
Location of motif
Coding region
Intergenic region
−log10(q_value)
5.025
5.050
5.075

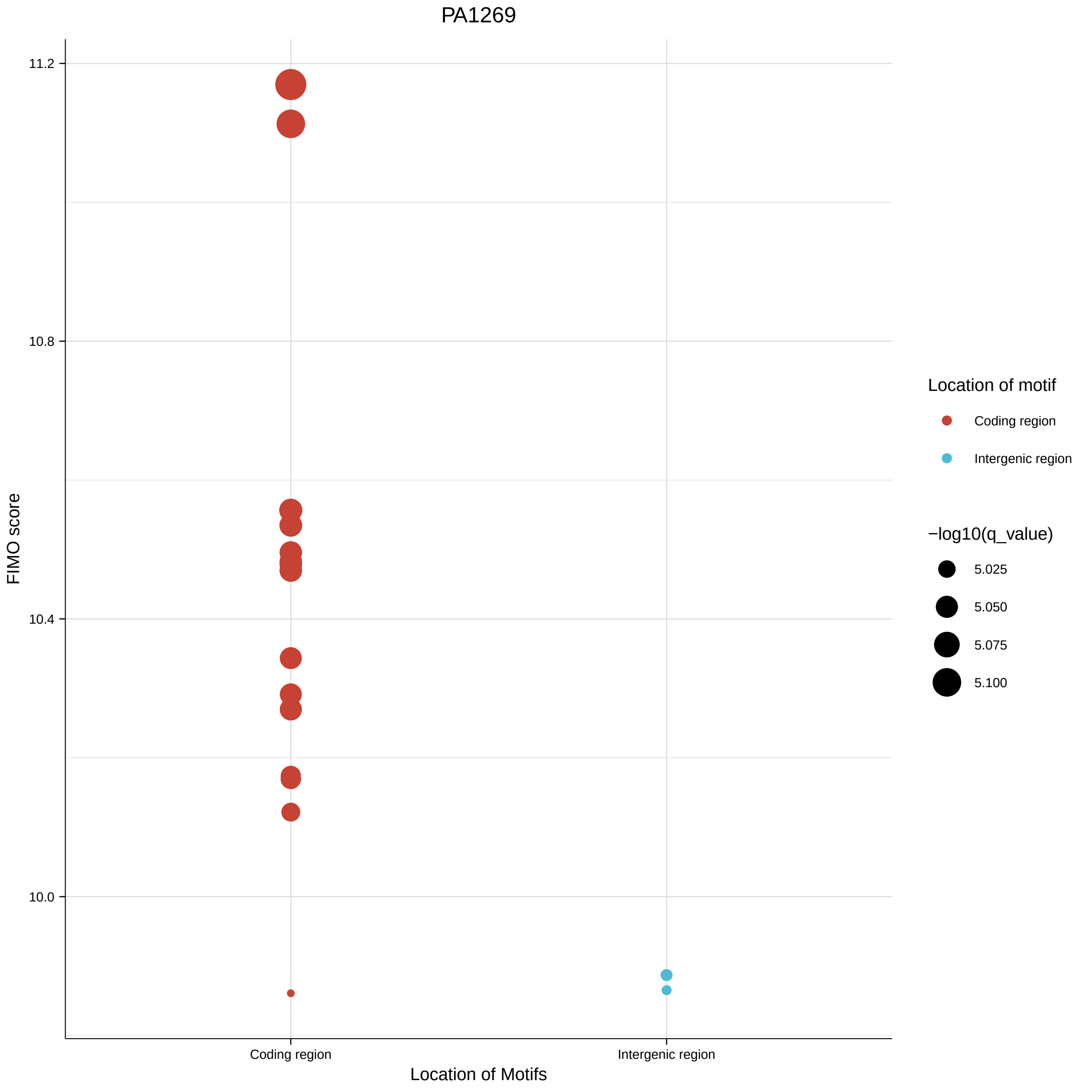

PA1269
FIMO score
11.2
10.8
10.4
10.0
Coding region
Intergenic region
Location of Motifs
Location of motif
Coding region
Intergenic region
−log10(q_value)
5.025
5.050
5.075
5.100

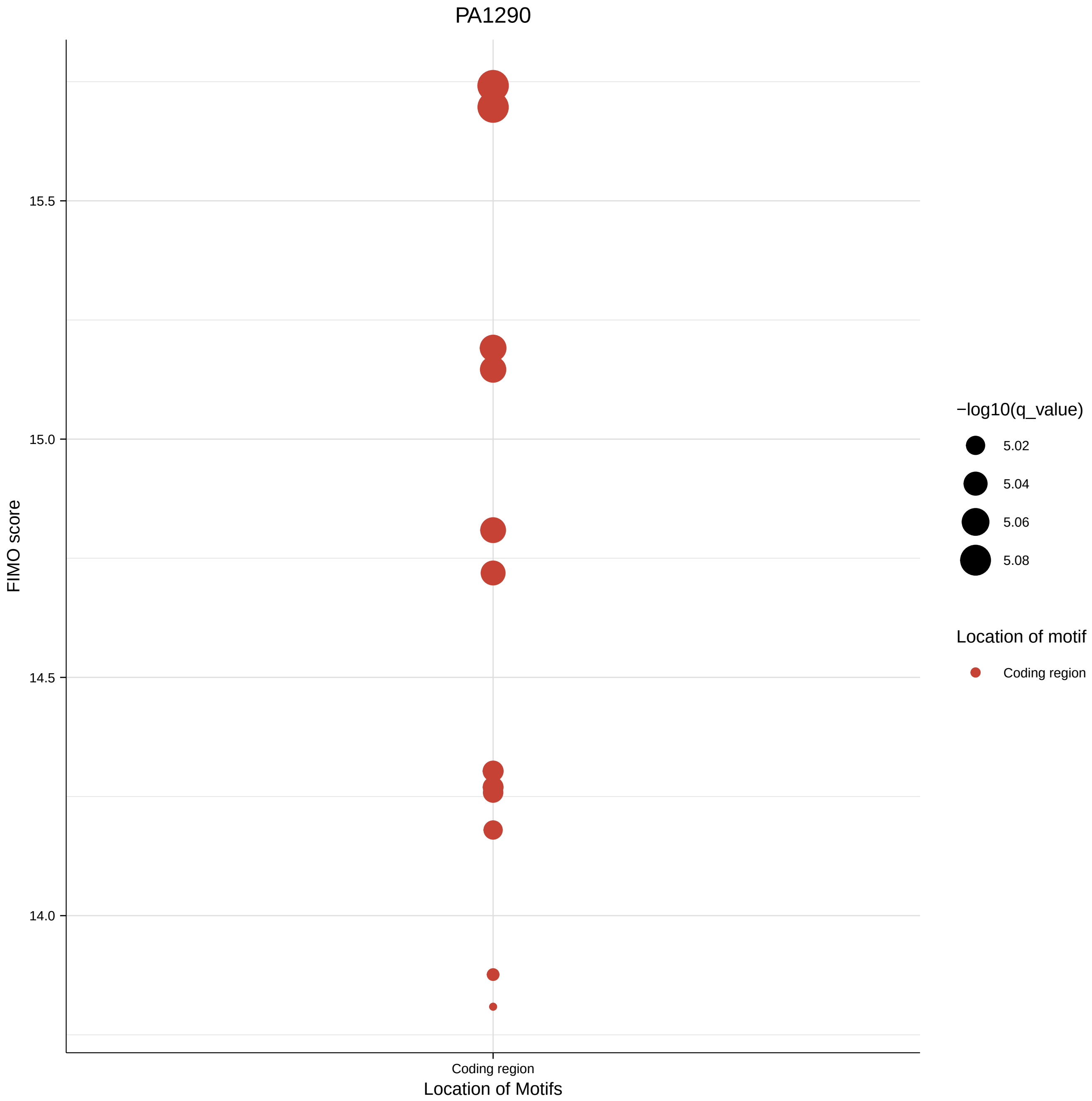
PA1290
FIMO score
15.5
15.0
14.5
14.0
Coding region
Location of Motifs
−log10(q_value)
5.02
5.04
5.06
5.08
Location of motif
Coding region

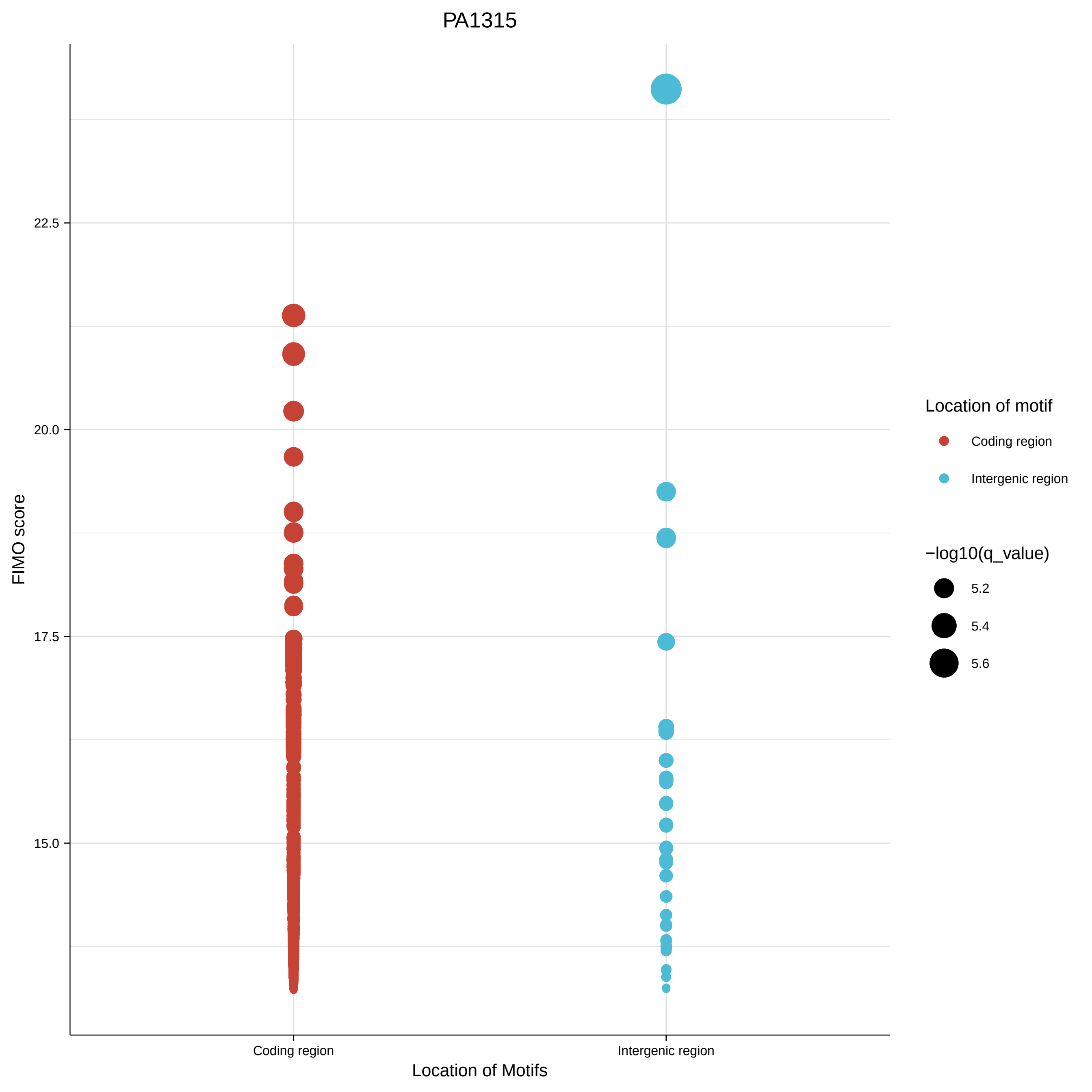

PA1315
FIMO score
22.5
20.0
17.5
15.0
Coding region
Intergenic region
Location of Motifs
Location of motif
Coding region
Intergenic region
−log10(q_value)
5.2
5.4
5.6

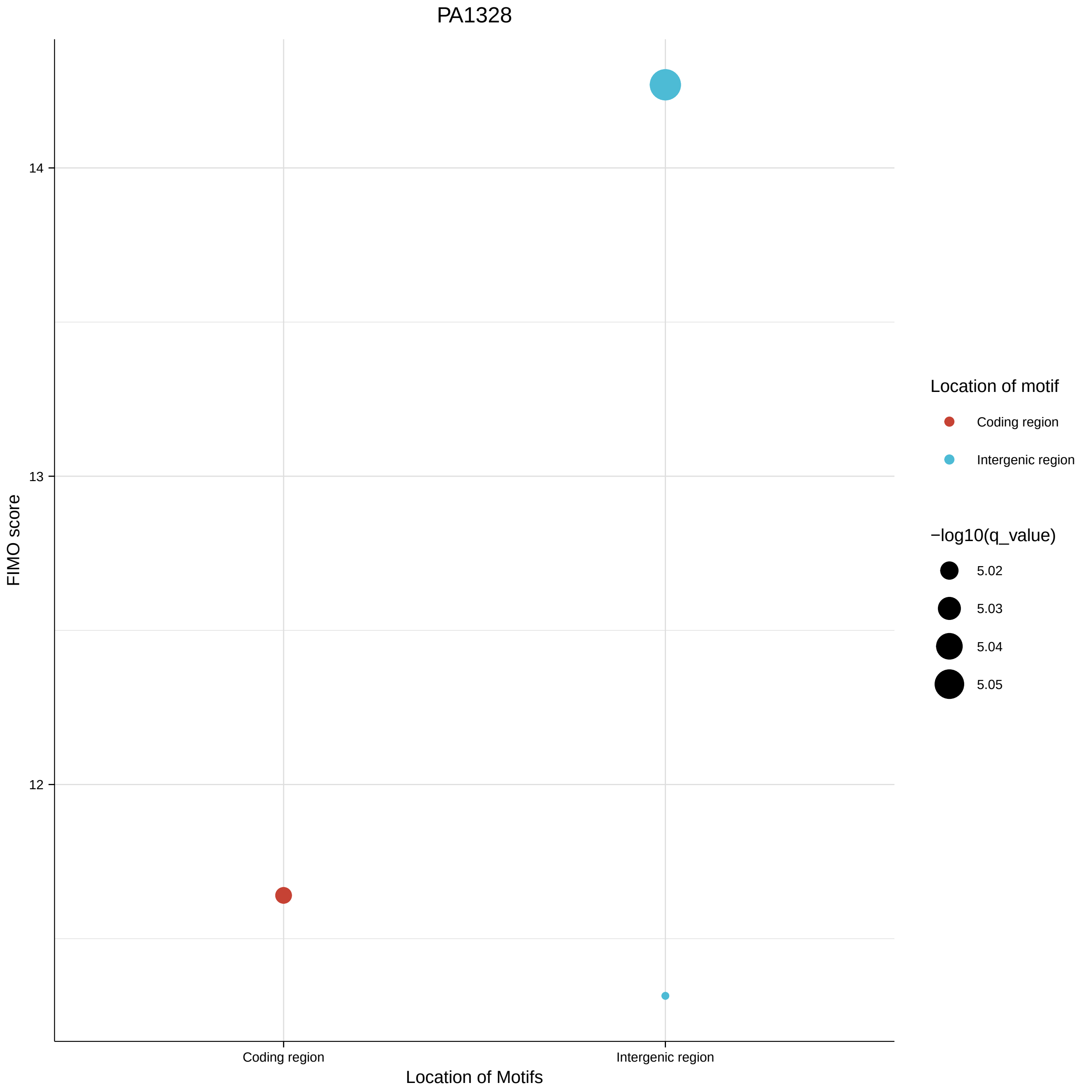

PA1328
FIMO score
14
13
12
Coding region
Intergenic region
Location of Motifs
Location of motif
Coding region
Intergenic region
−log10(q_value)
5.02
5.03
5.04
5.05

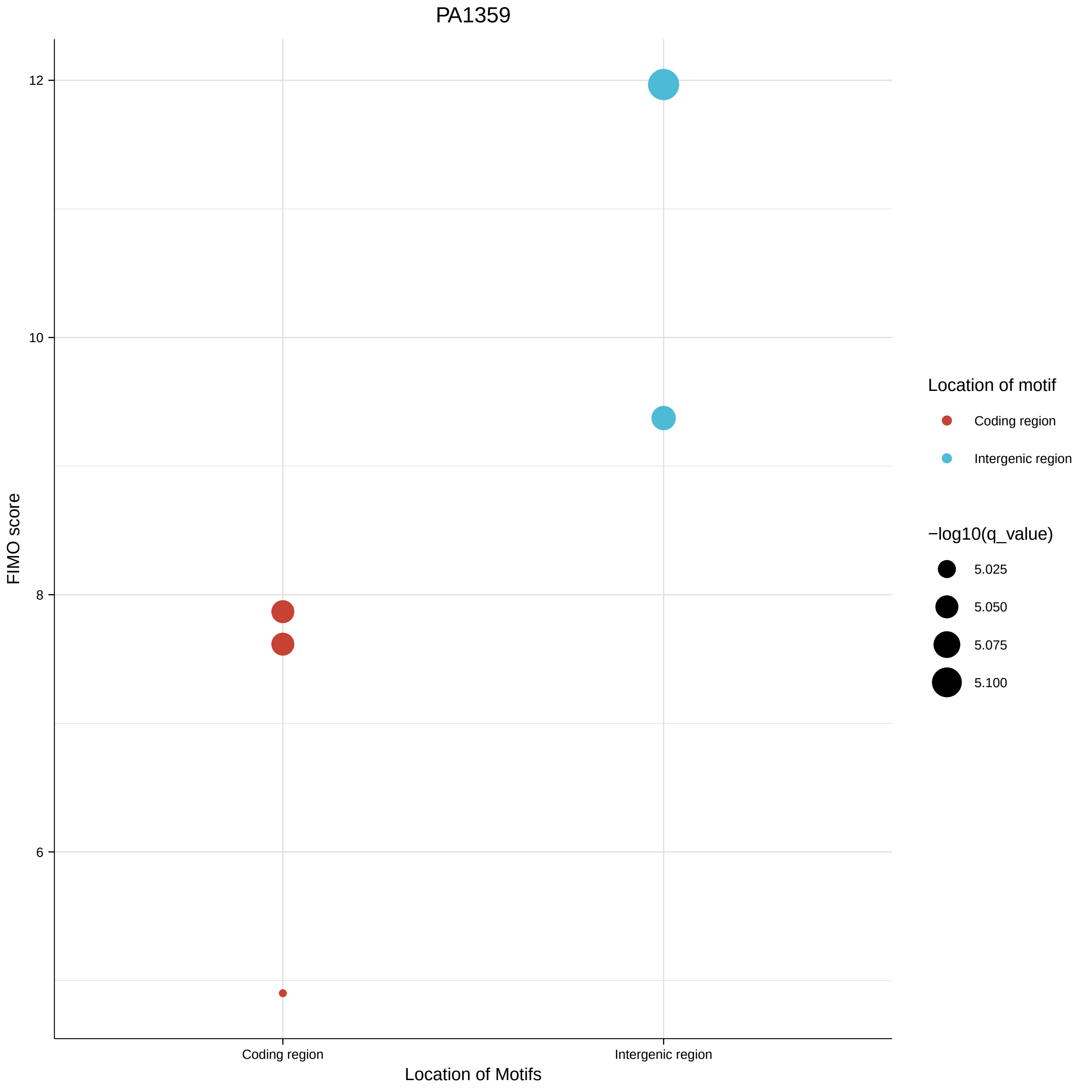

PA1359
FIMO score
12
10
8
6
Coding region
Intergenic region
Location of Motifs
Location of motif
Coding region
Intergenic region
−log10(q_value)
5.025
5.050
5.075
5.100

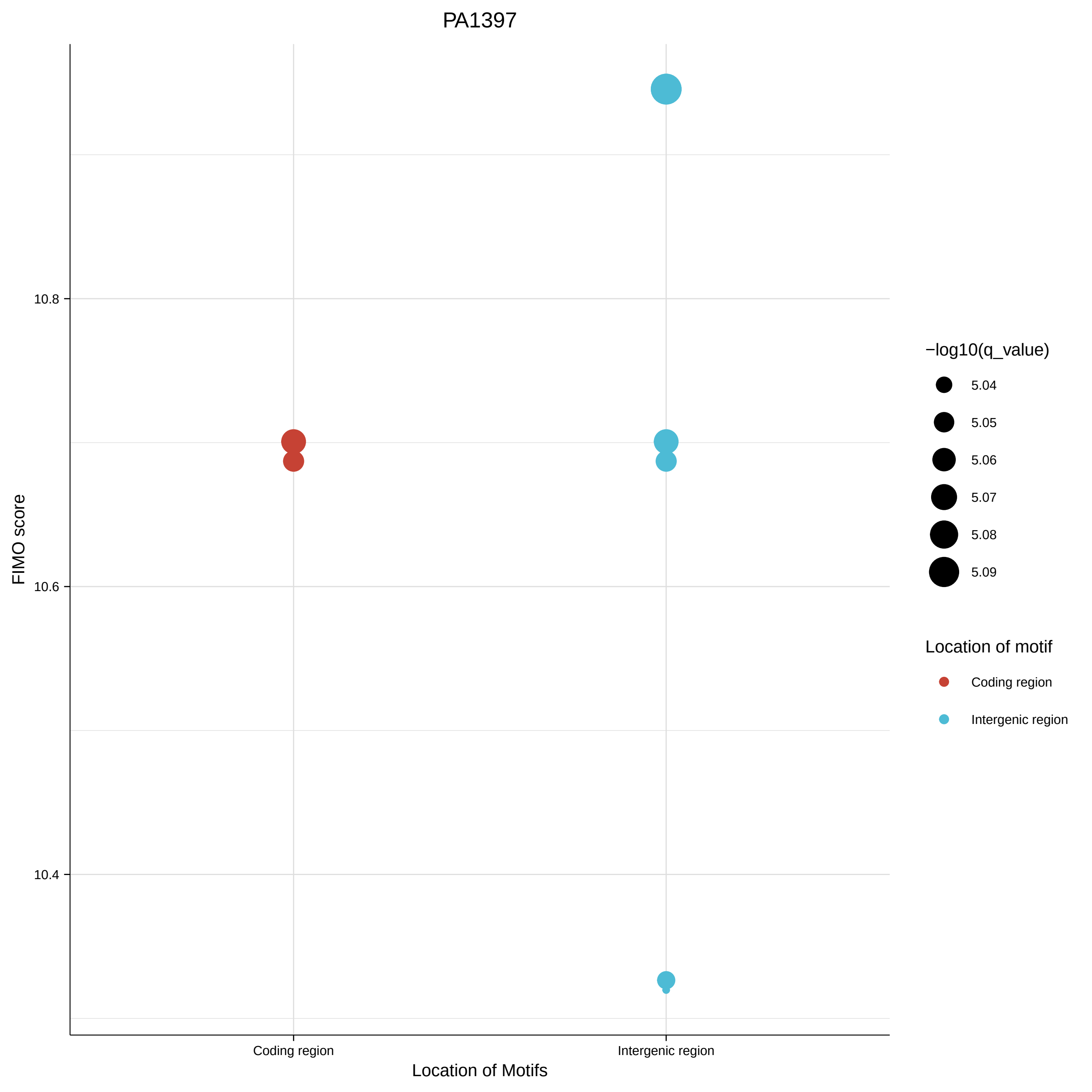

PA1397
FIMO score
10.8
10.6
10.4
Coding region
Intergenic region
Location of Motifs
−log10(q_value)
5.04
5.05
5.06
5.07
5.08
5.09
Location of motif
Coding region
Intergenic region

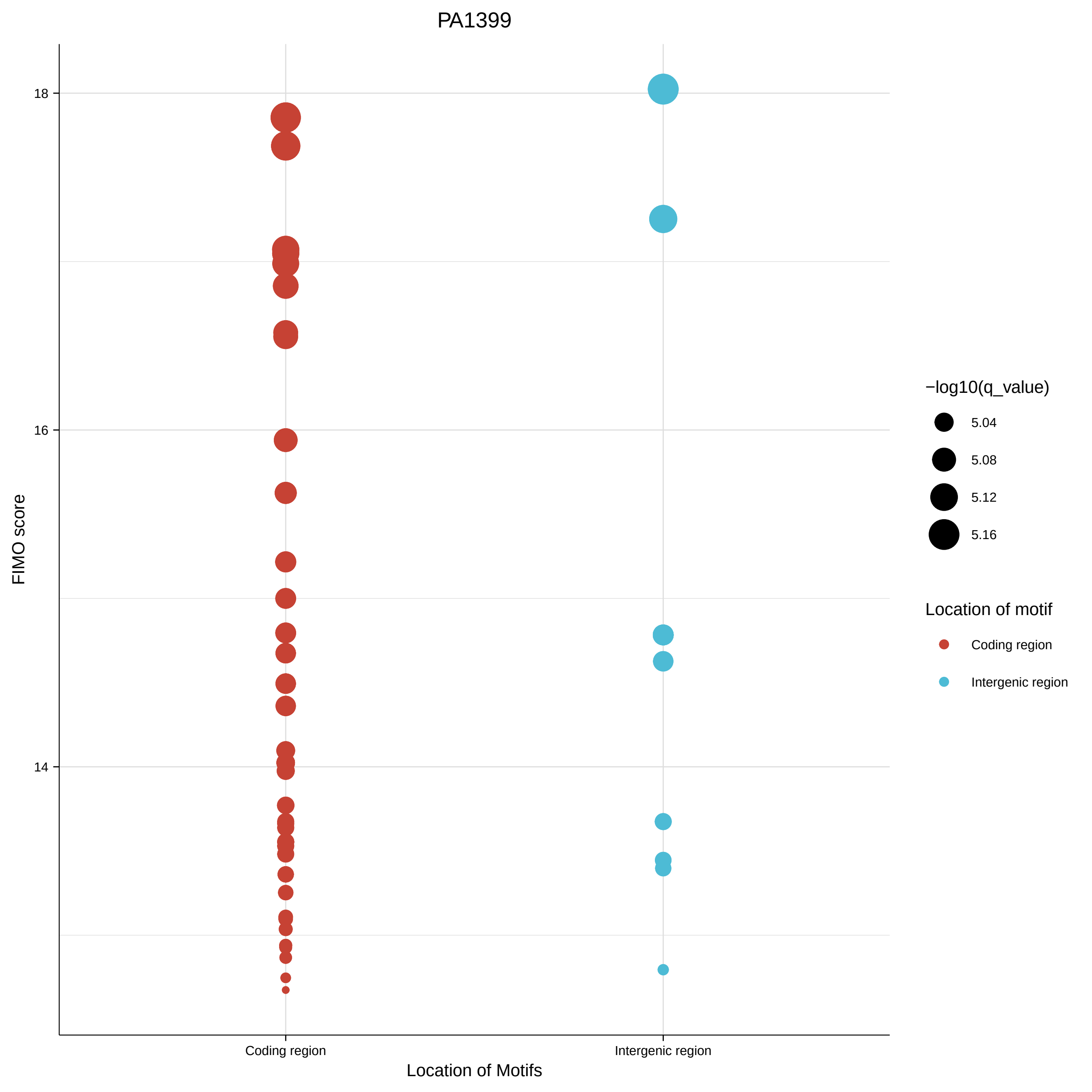

PA1399
FIMO score
18
16
14
Coding region
Intergenic region
Location of Motifs
−log10(q_value)
5.04
5.08
5.12
5.16
Location of motif
Coding region
Intergenic region

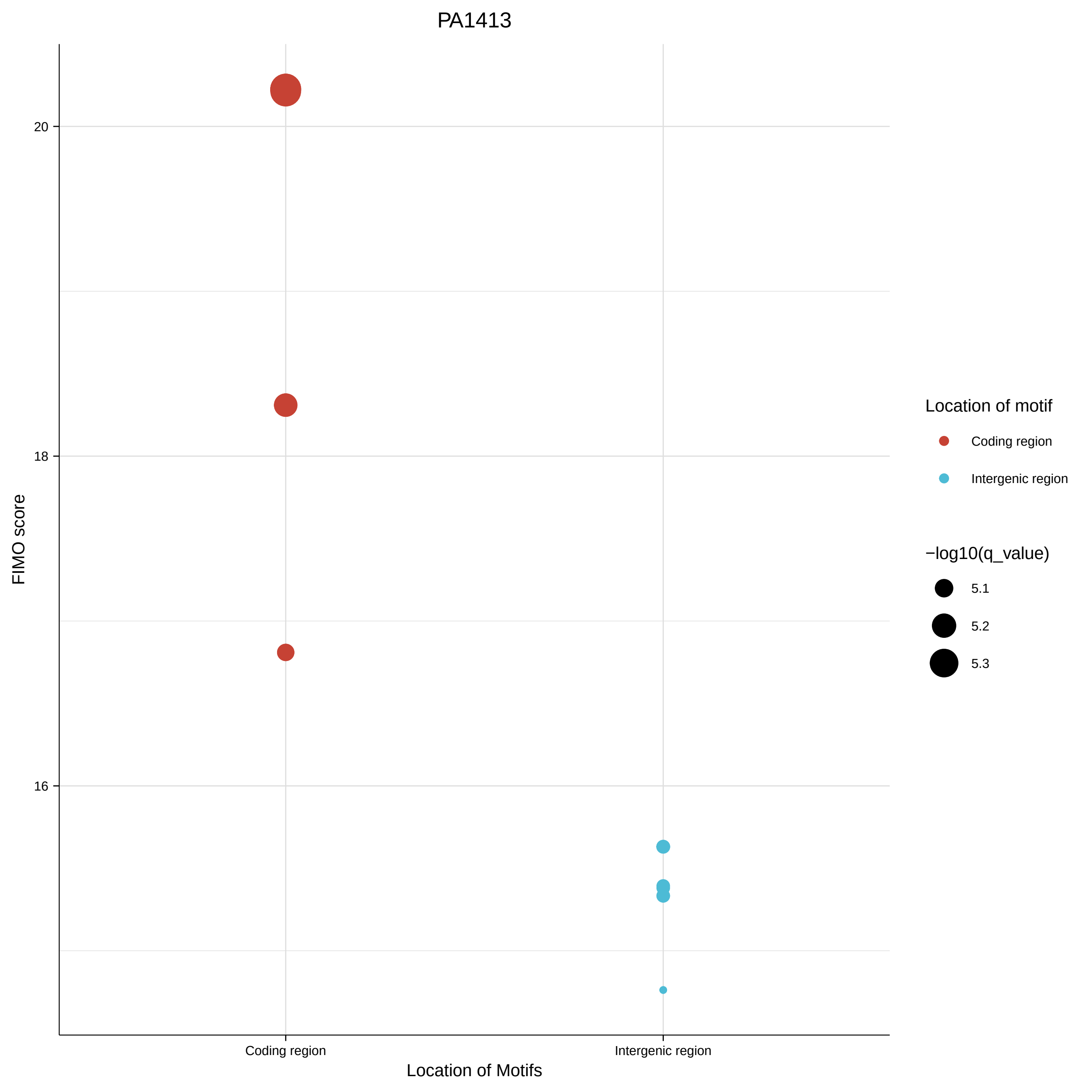
PA1413
FIMO score
20
18
16
Coding region
Intergenic region
Location of Motifs
Location of motif
Coding region
Intergenic region
−log10(q_value)
5.1
5.2
5.3

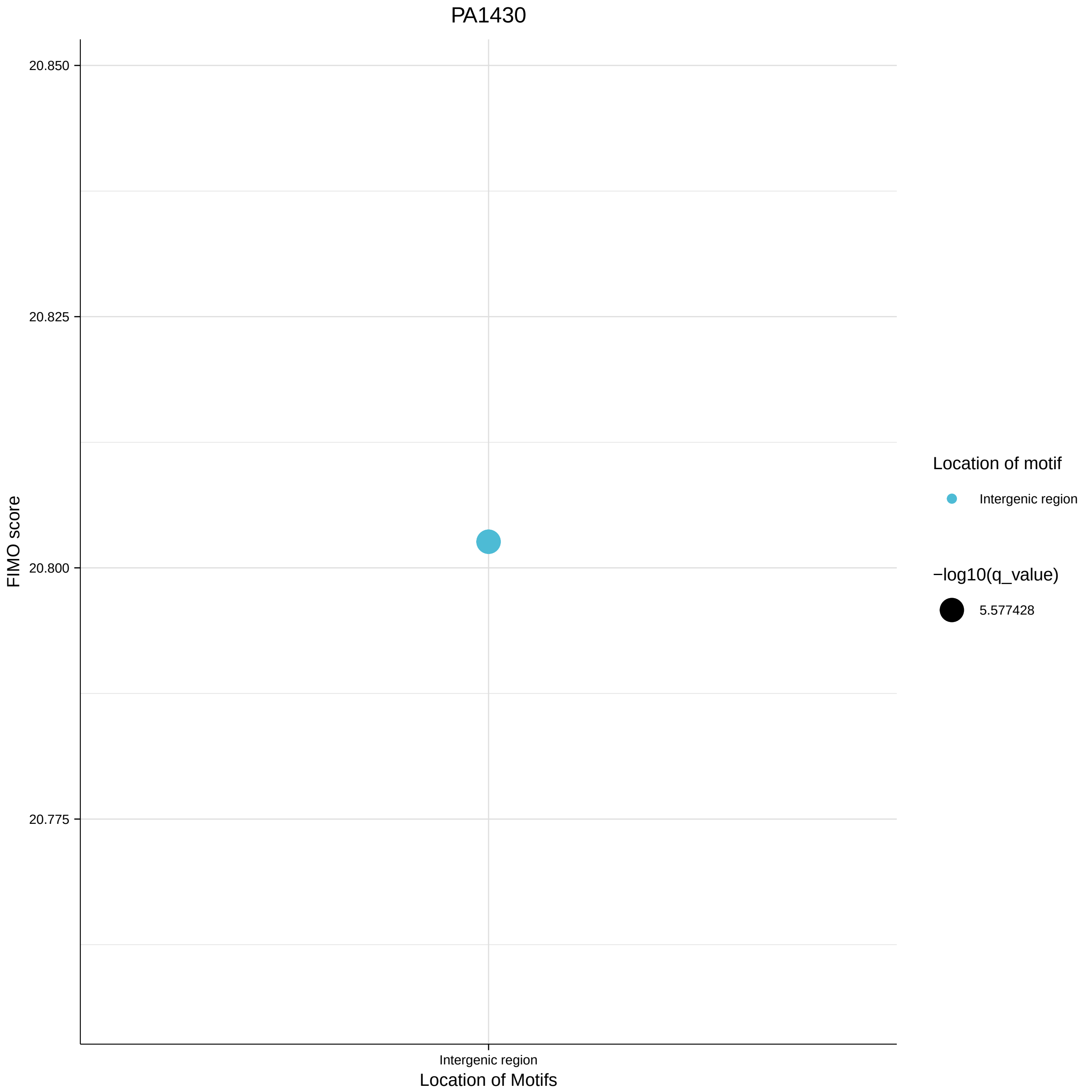
PA1430
FIMO score
20.850
20.825
20.800
20.775
Intergenic region
Location of Motifs
Location of motif
Intergenic region
−log10(q_value)
5.577428

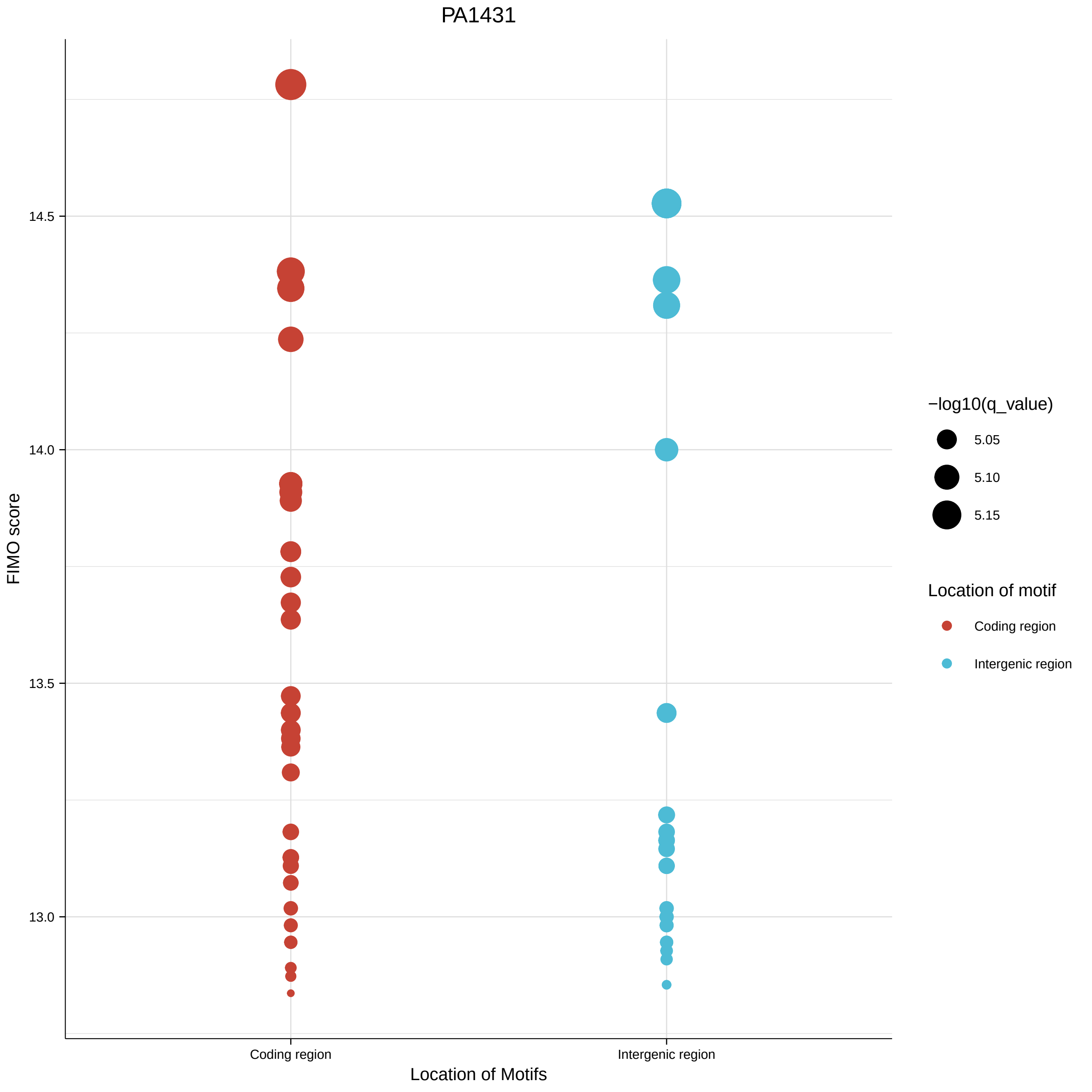
PA1431
FIMO score
14.5
14.0
13.5
13.0
Coding region
Intergenic region
Location of Motifs
−log10(q_value)
5.05
5.10
5.15
Location of motif
Coding region
Intergenic region

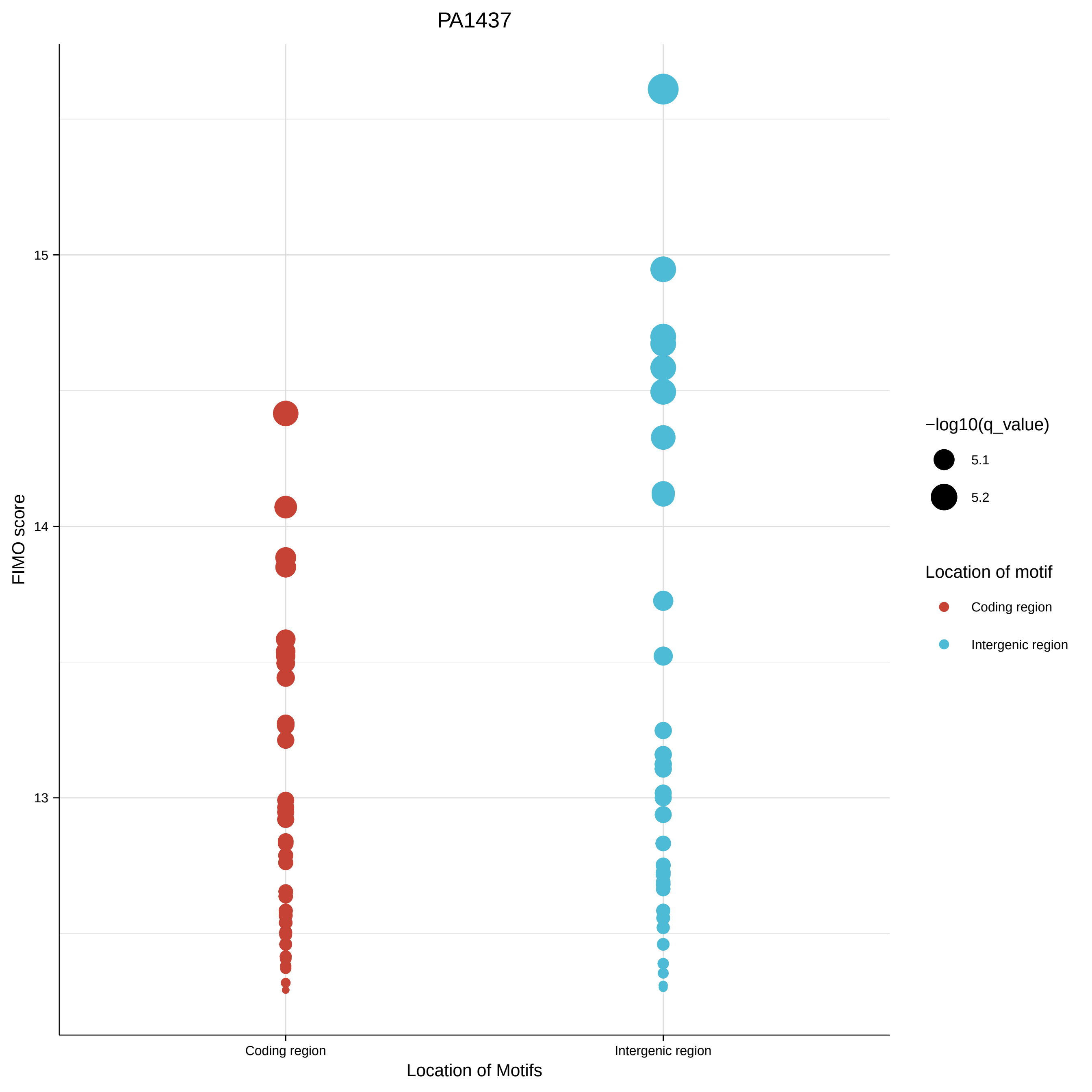
PA1437
FIMO score
15
14
13
Coding region
Intergenic region
Location of Motifs
−log10(q_value)
5.1
5.2
Location of motif
Coding region
Intergenic region

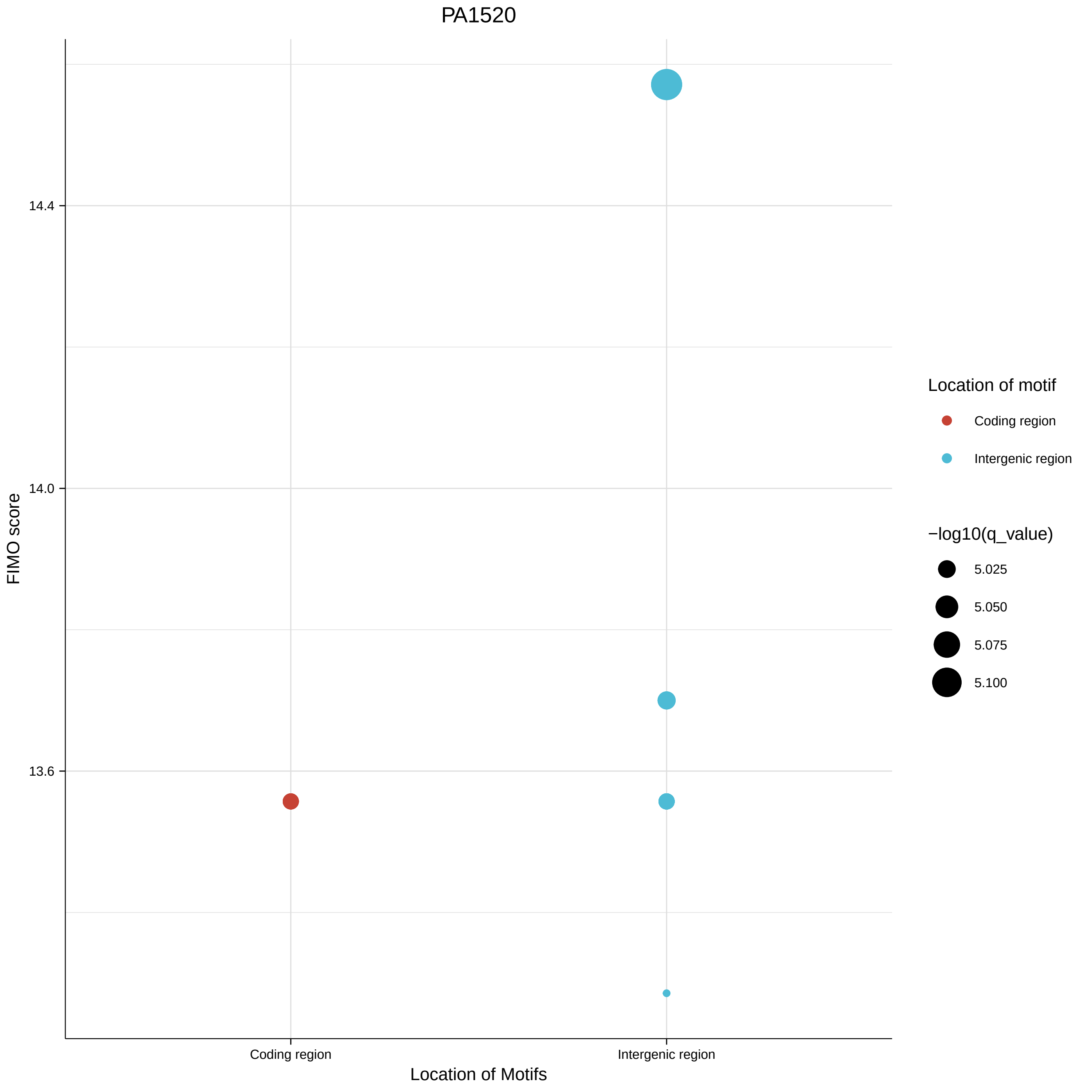

PA1520
FIMO score
14.4
14.0
13.6
Coding region
Intergenic region
Location of Motifs
Location of motif
Coding region
Intergenic region
−log10(q_value)
5.025
5.050
5.075
5.100

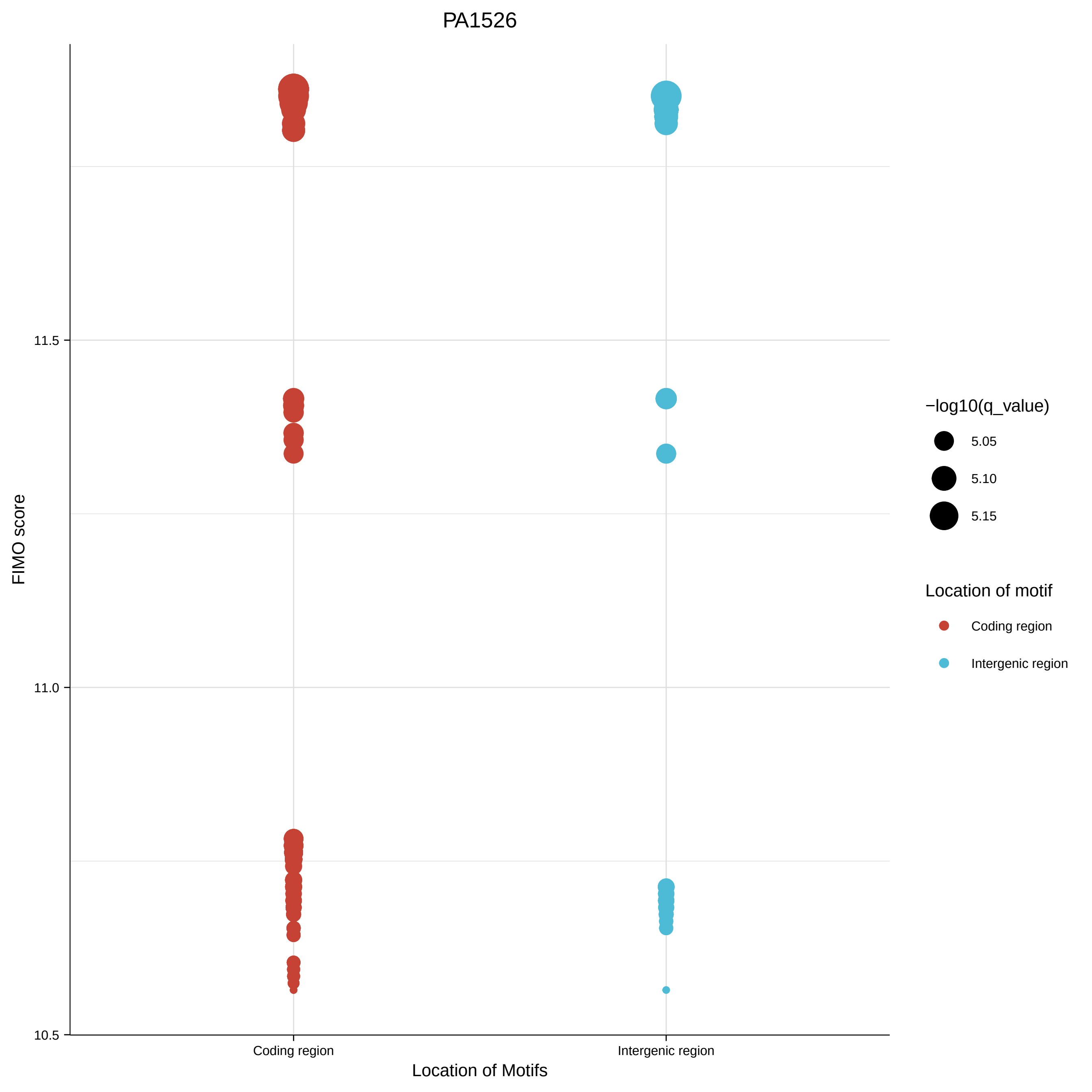
PA1526
FIMO score
11.5
11.0
10.5
Coding region
Intergenic region
Location of Motifs
−log10(q_value)
5.05
5.10
5.15
Location of motif
Coding region
Intergenic region

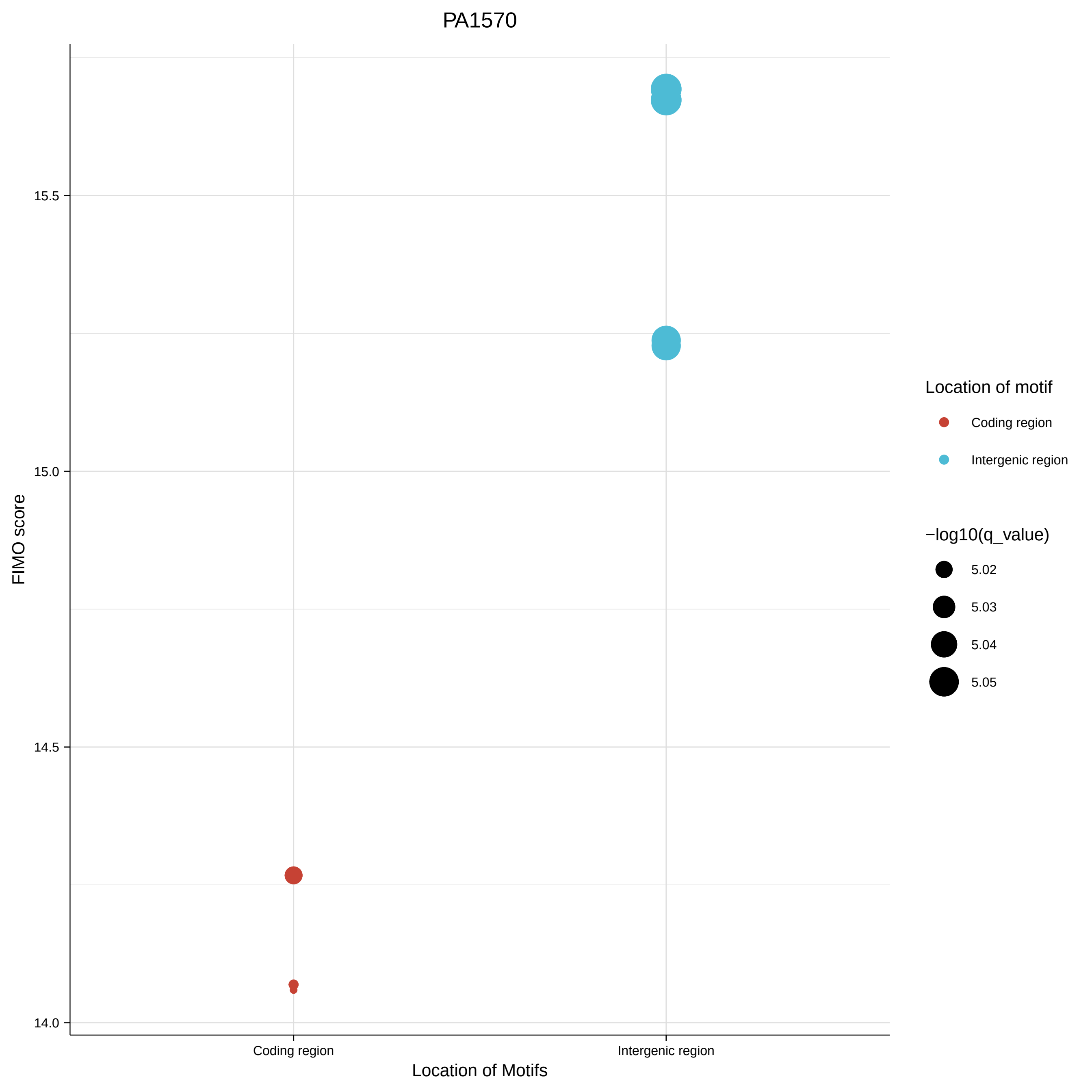
PA1570
FIMO score
15.5
15.0
14.5
14.0
Coding region
Intergenic region
Location of Motifs
Location of motif
Coding region
Intergenic region
−log10(q_value)
5.02
5.03
5.04
5.05

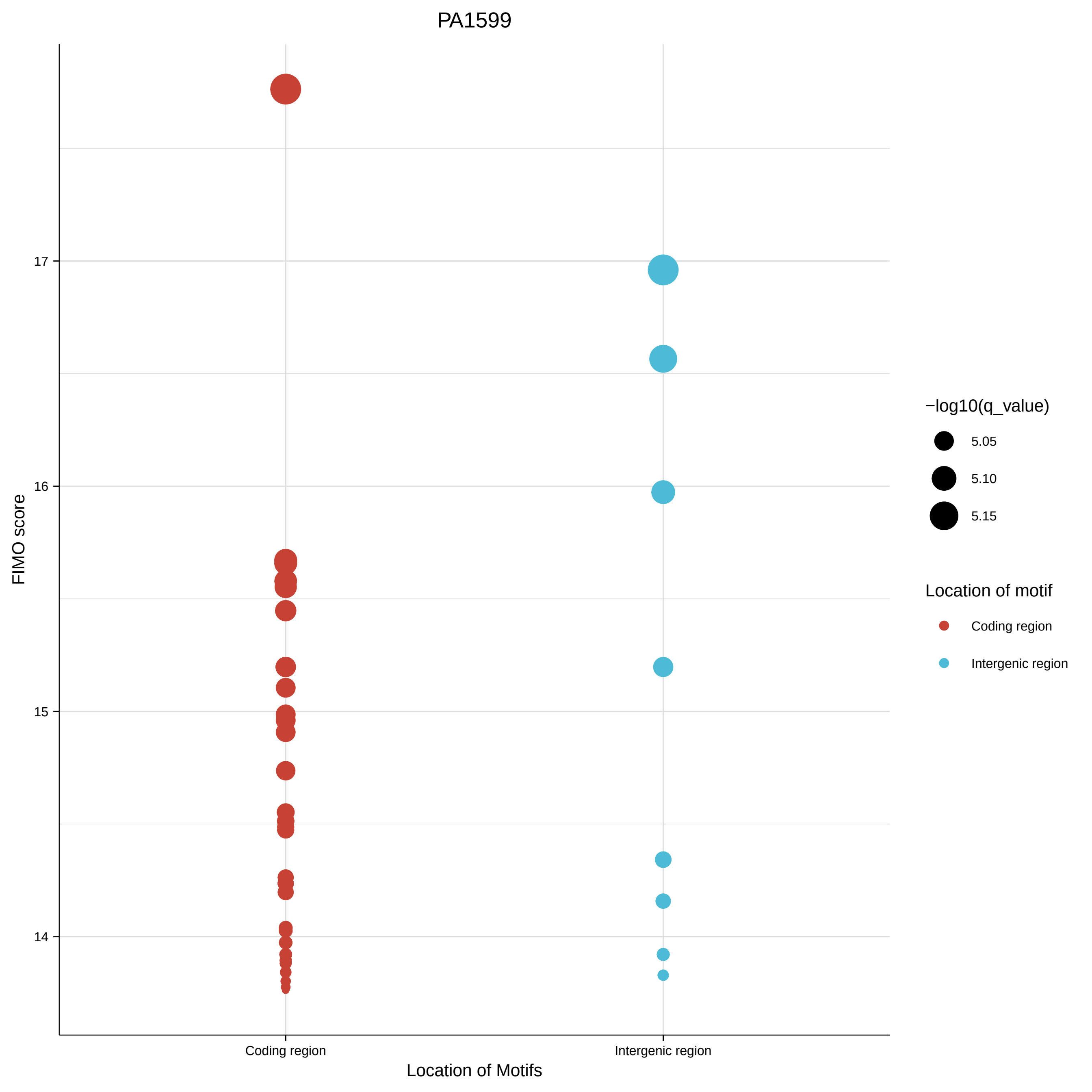
PA1599
17
16
15
14
FIMO score
Coding region
Intergenic region
Location of Motifs
−log10(q_value)
5.05
5.10
5.15
Location of motif
Coding region
Intergenic region

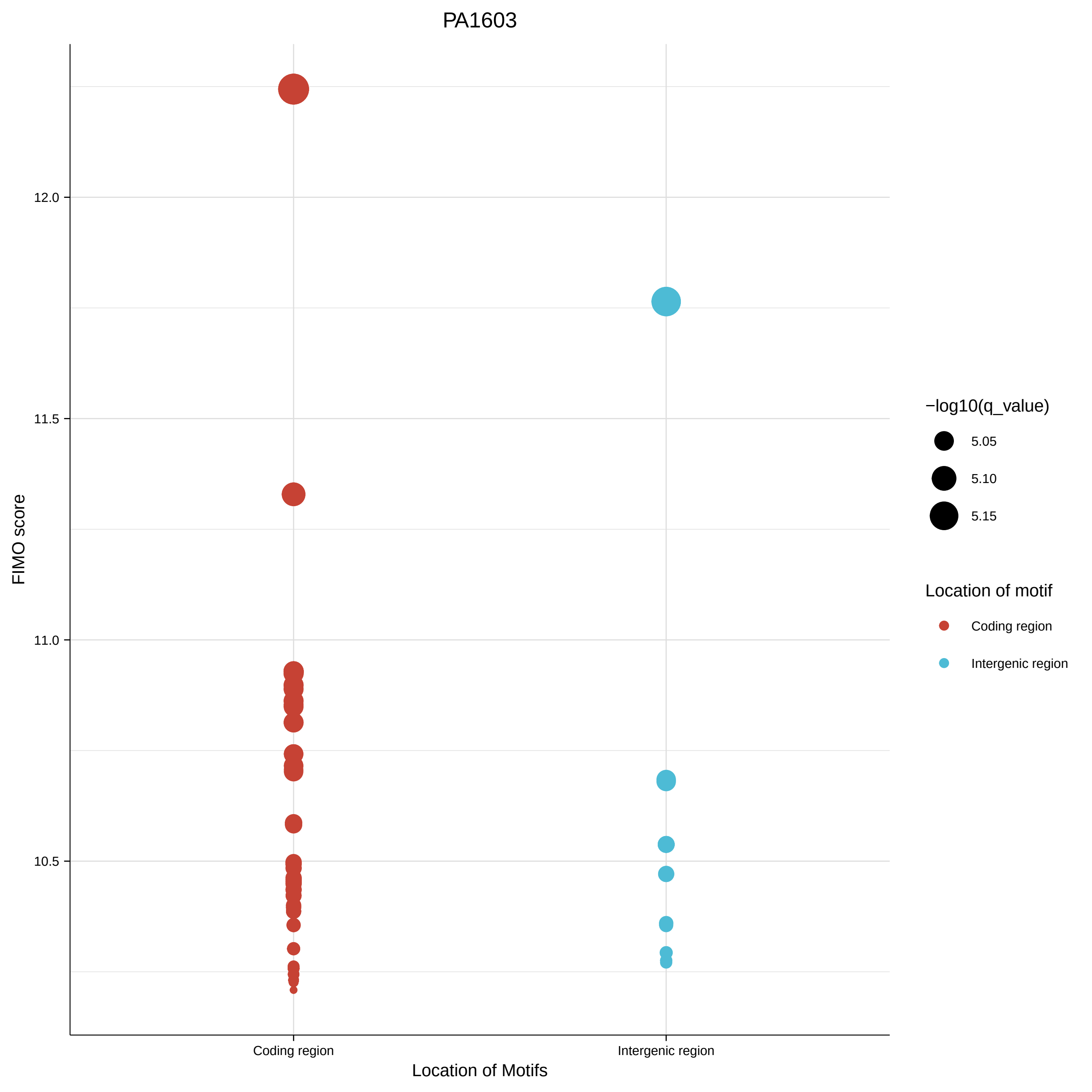
PA1603
FIMO score
12.0
11.5
11.0
10.5
Coding region
Intergenic region
Location of Motifs
−log10(q_value)
5.05
5.10
5.15
Location of motif
Coding region
Intergenic region

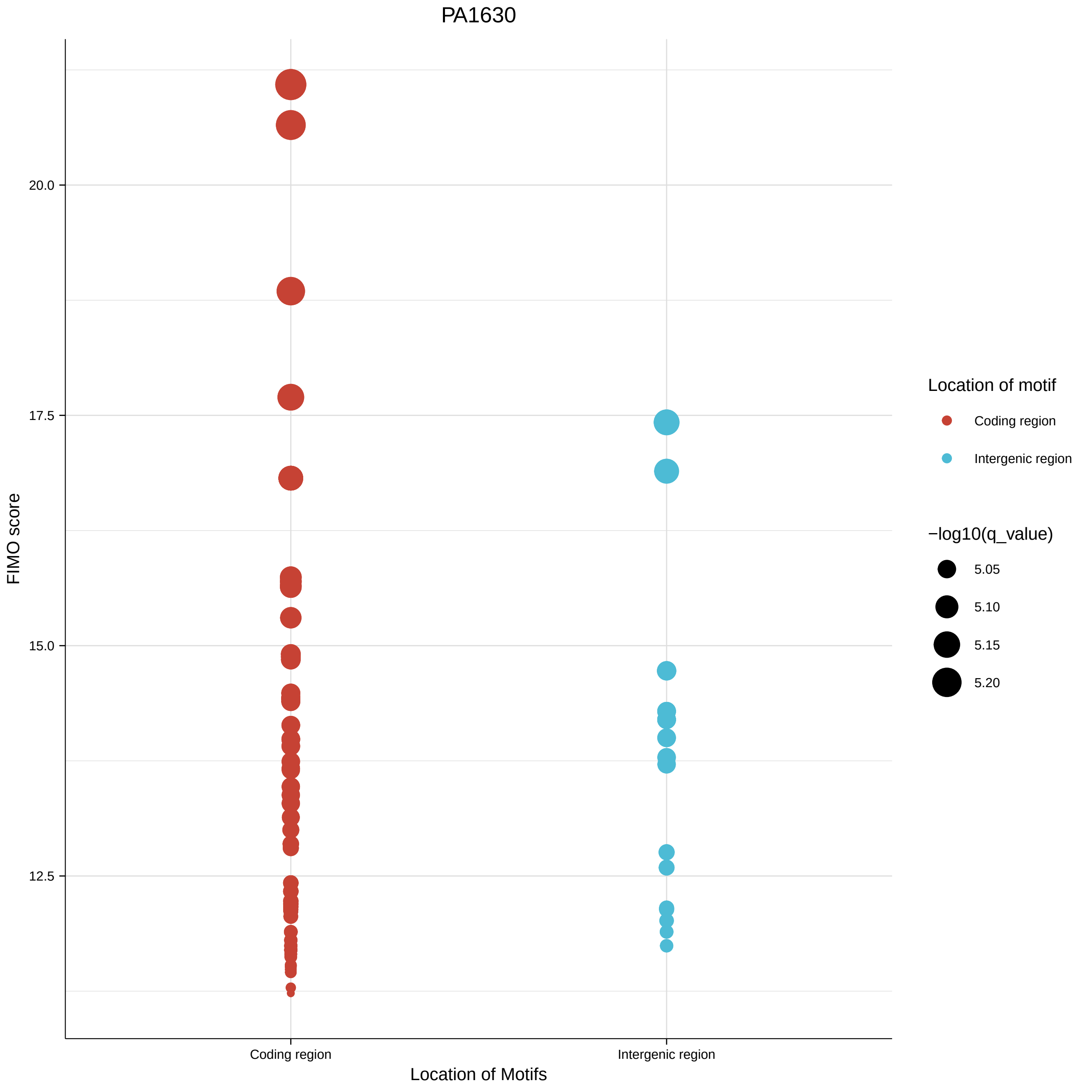
PA1630
FIMO score
20.0
17.5
15.0
12.5
Coding region
Intergenic region
Location of Motifs
Location of motif
Coding region
Intergenic region
−log10(q_value)
5.05
5.10
5.15
5.20

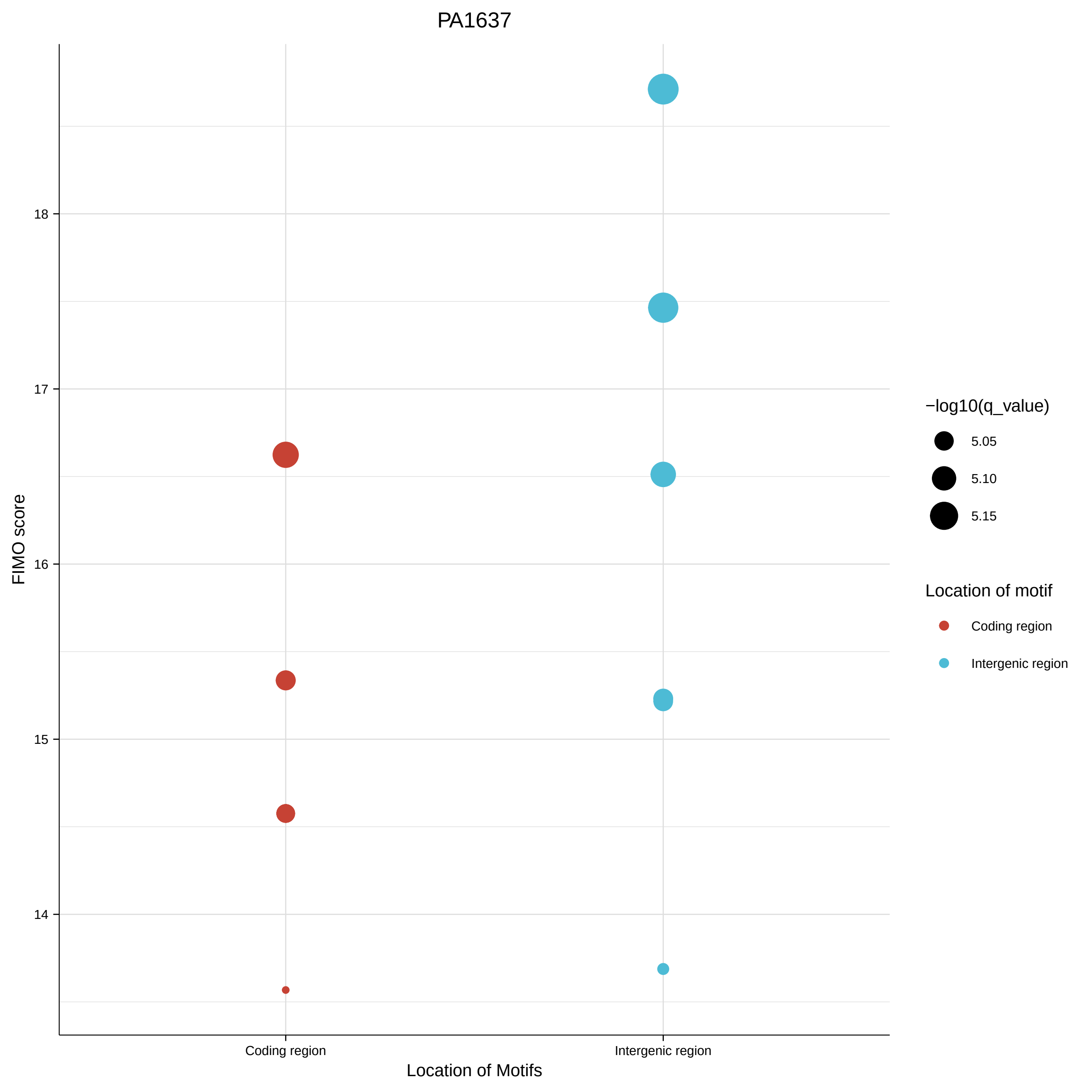
PA1637
FIMO score
18
17
16
15
14
Coding region
Intergenic region
Location of Motifs
−log10(q_value)
5.05
5.10
5.15
Location of motif
Coding region
Intergenic region

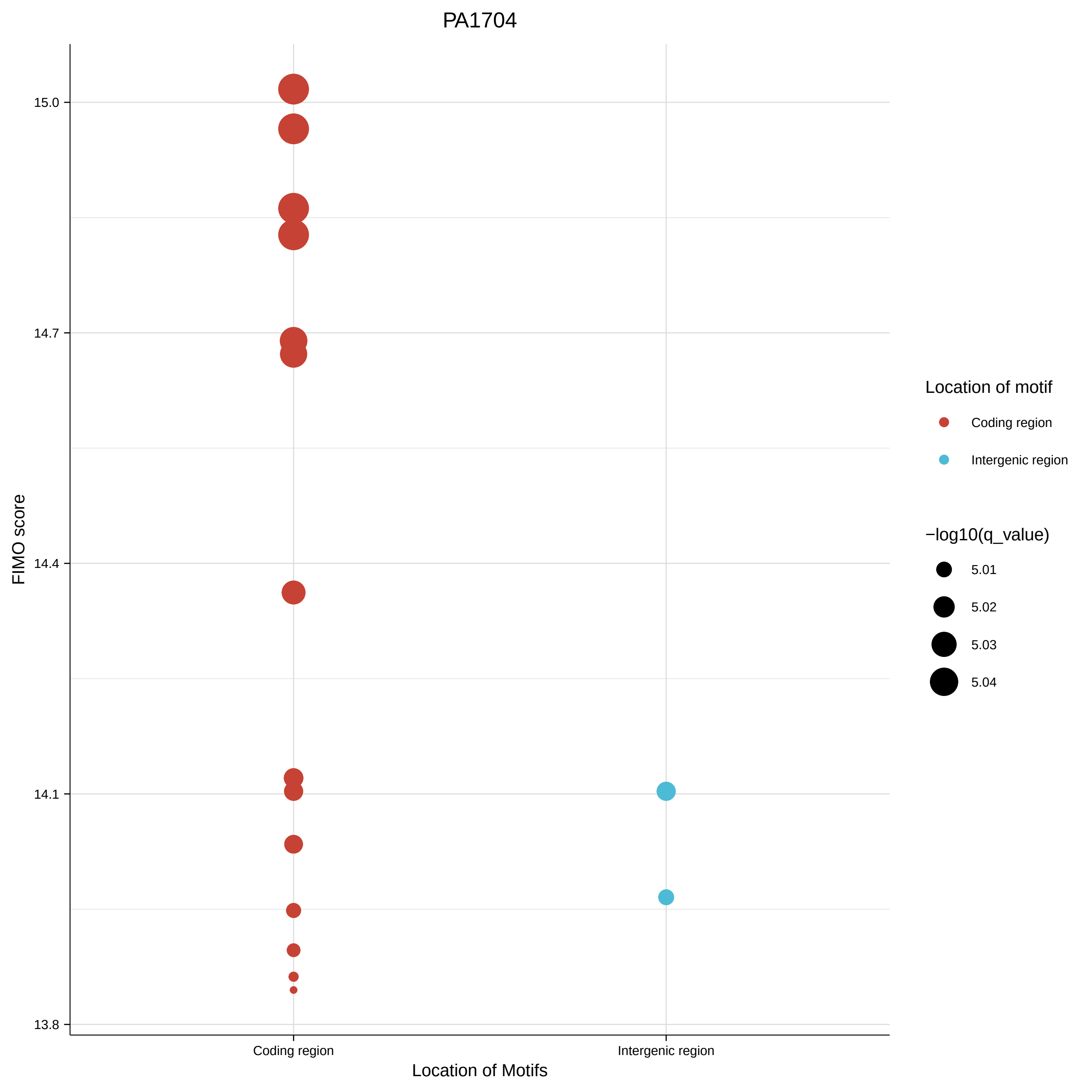

PA1704
FIMO score
15.0
14.7
14.4
14.1
13.8
Coding region
Intergenic region
Location of Motifs
Location of motif
Coding region
Intergenic region
−log10(q_value)
5.01
5.02
5.03
5.04

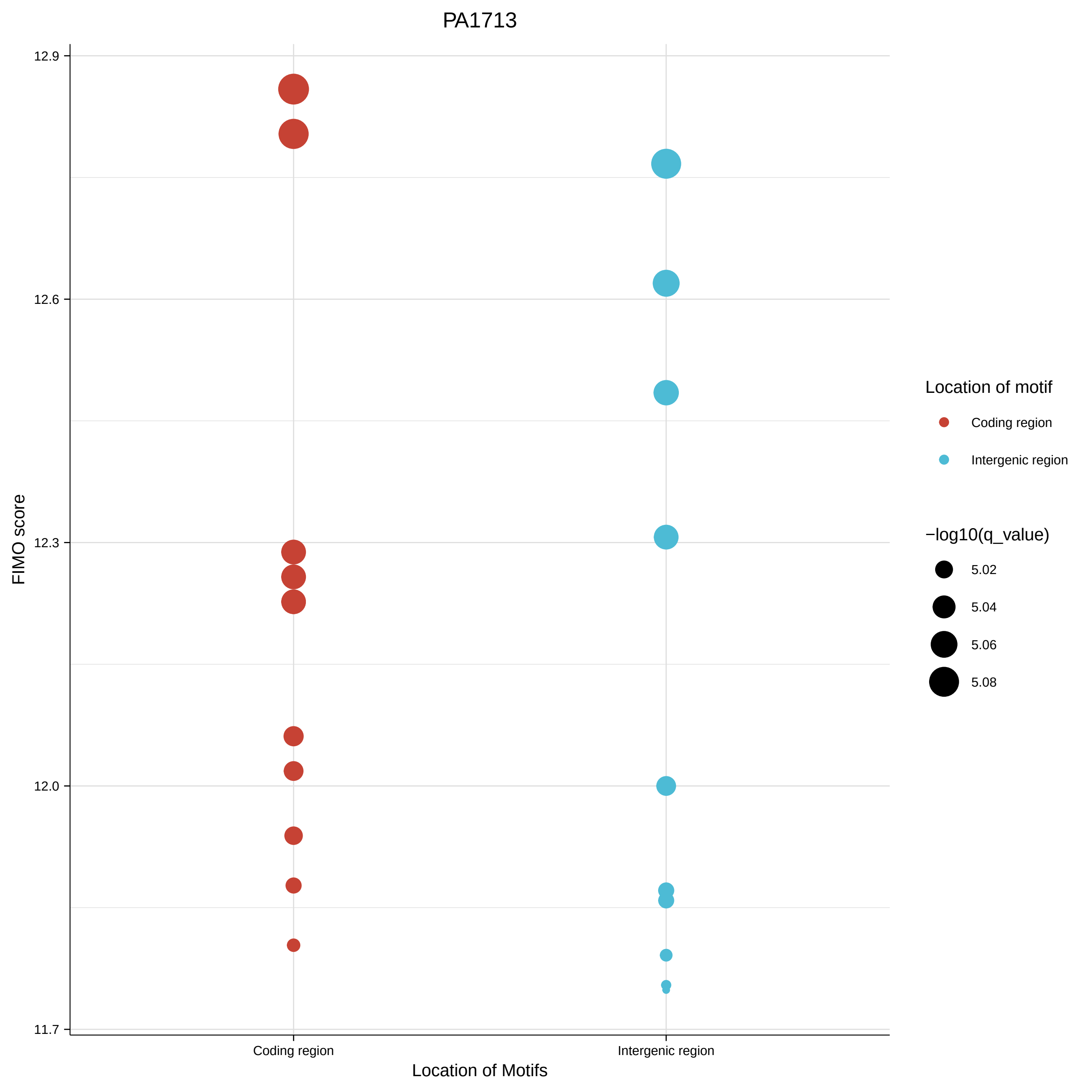
PA1713
12.9
12.6
12.3
12.0
11.7
FIMO score
Coding region
Intergenic region
Location of Motifs
Location of motif
Coding region
Intergenic region
−log10(q_value)
5.02
5.04
5.06
5.08

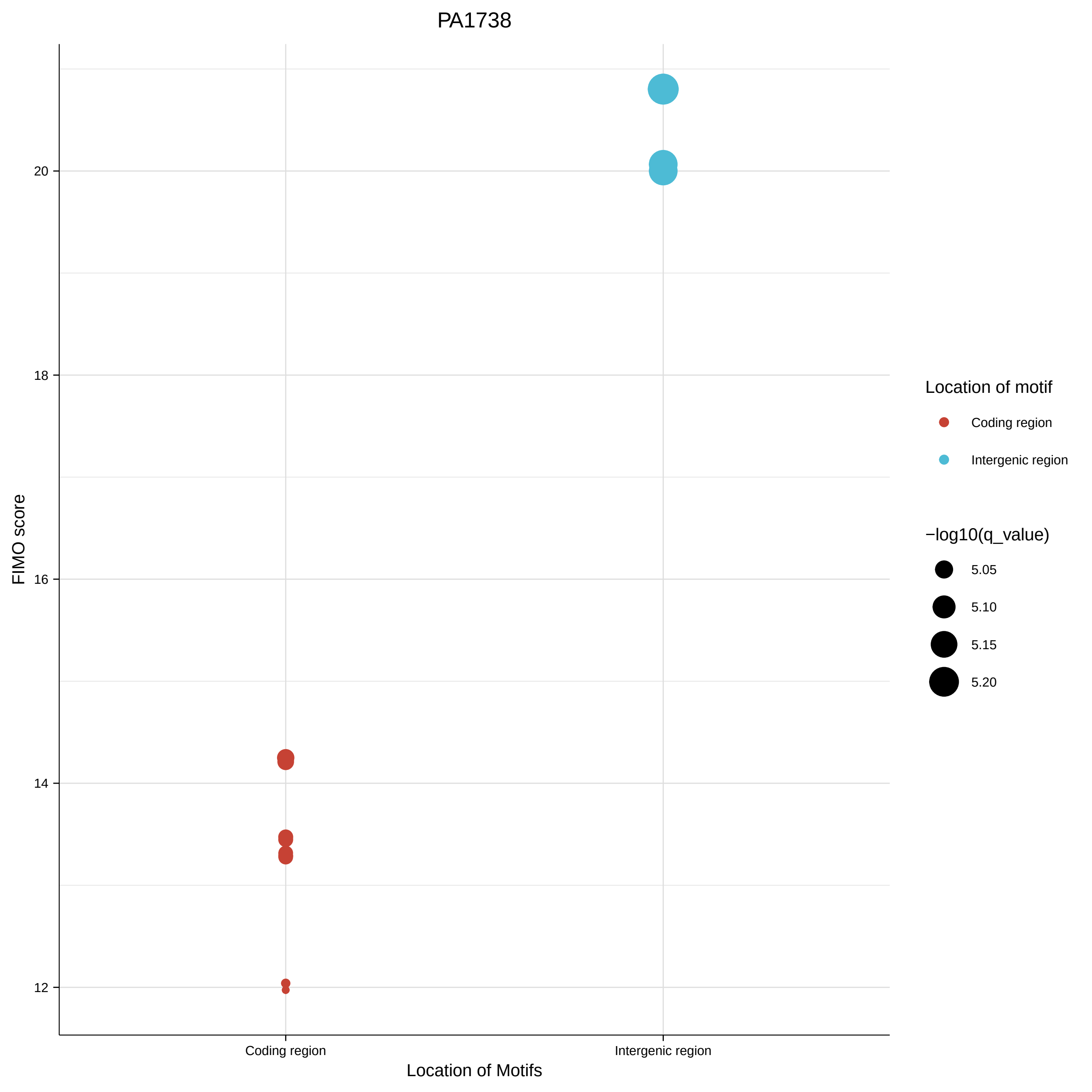
PA1738
FIMO score
20
18
16
14
12
Coding region
Intergenic region
Location of Motifs
Location of motif
Coding region
Intergenic region
−log10(q_value)
5.05
5.10
5.15
5.20

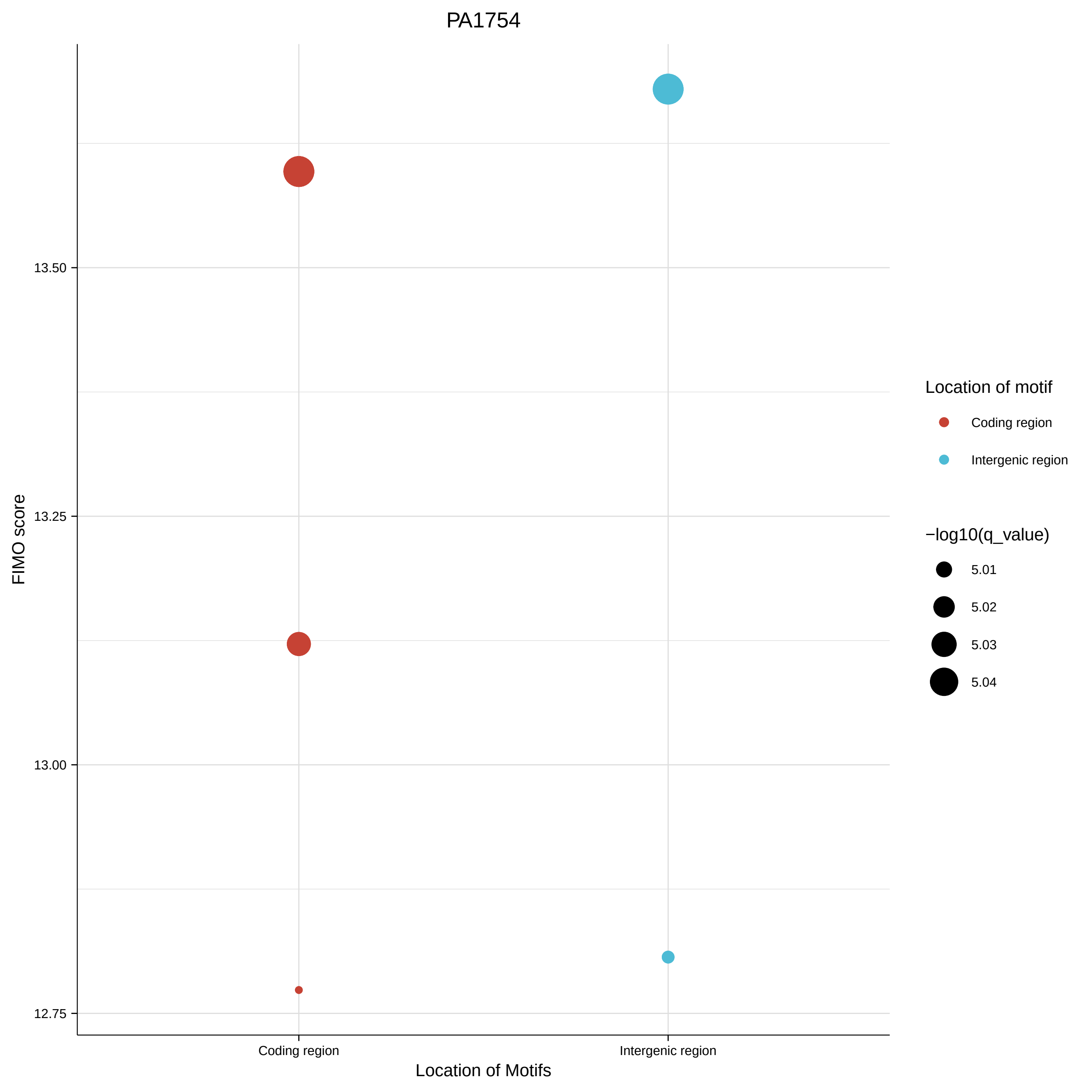
PA1754
FIMO score
13.50
13.25
13.00
12.75
Coding region
Intergenic region
Location of Motifs
Location of motif
Coding region
Intergenic region
−log10(q_value)
5.01
5.02
5.03
5.04

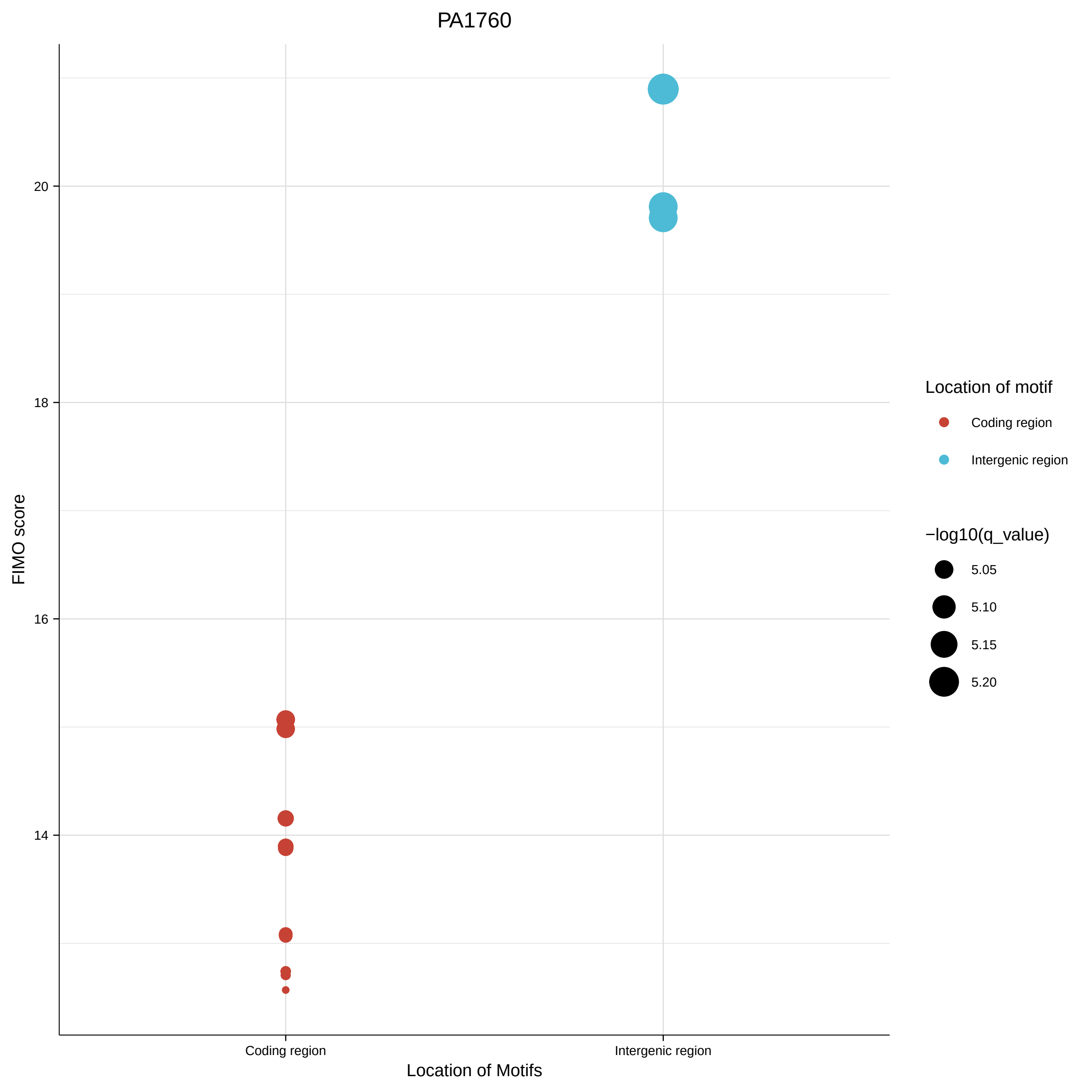
PA1760
FIMO score
20
18
16
14
Coding region
Intergenic region
Location of Motifs
Location of motif
Coding region
Intergenic region
−log10(q_value)
5.05
5.10
5.15
5.20

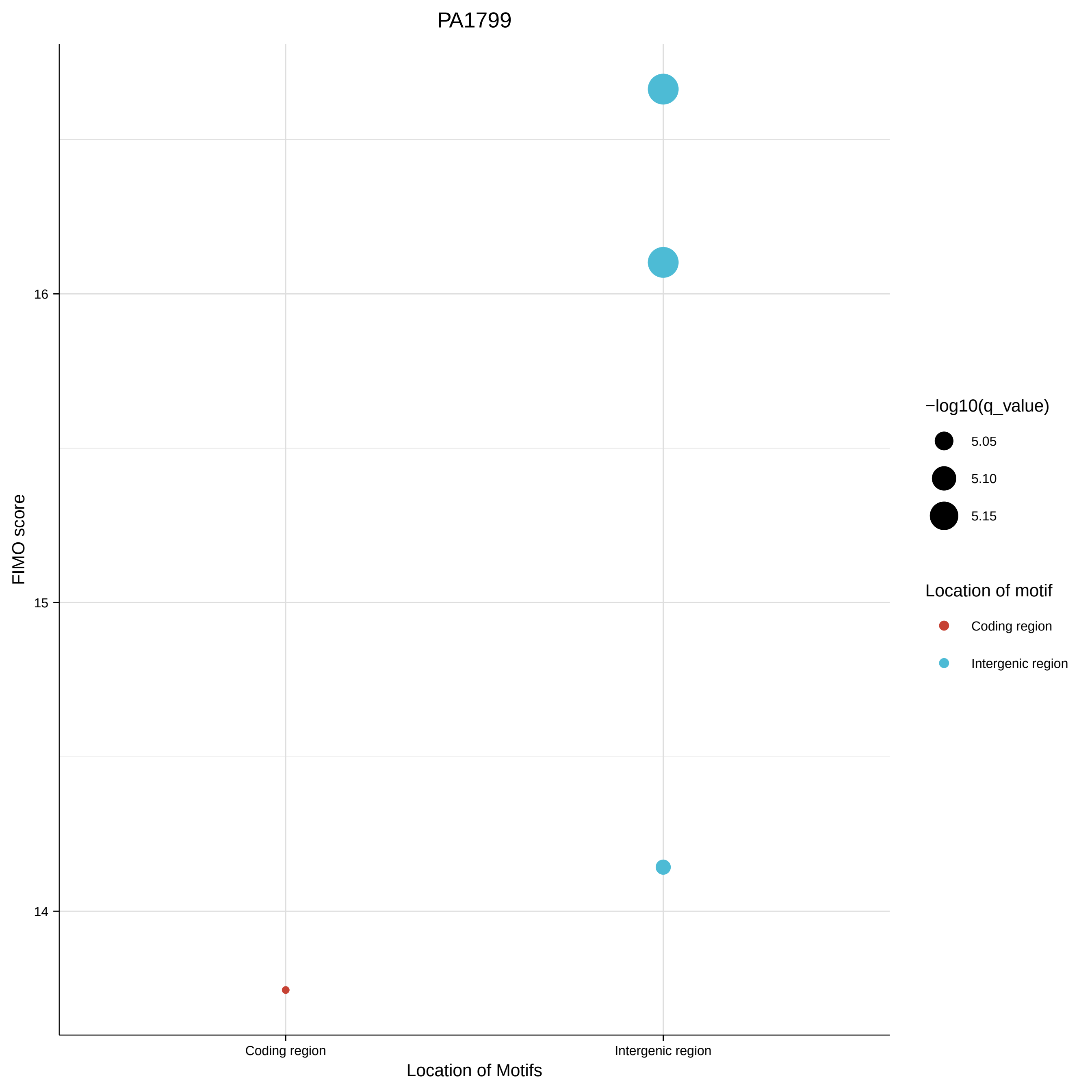
PA1799
FIMO score
16
15
14
Coding region
Intergenic region
Location of Motifs
−log10(q_value)
5.05
5.10
5.15
Location of motif
Coding region
Intergenic region

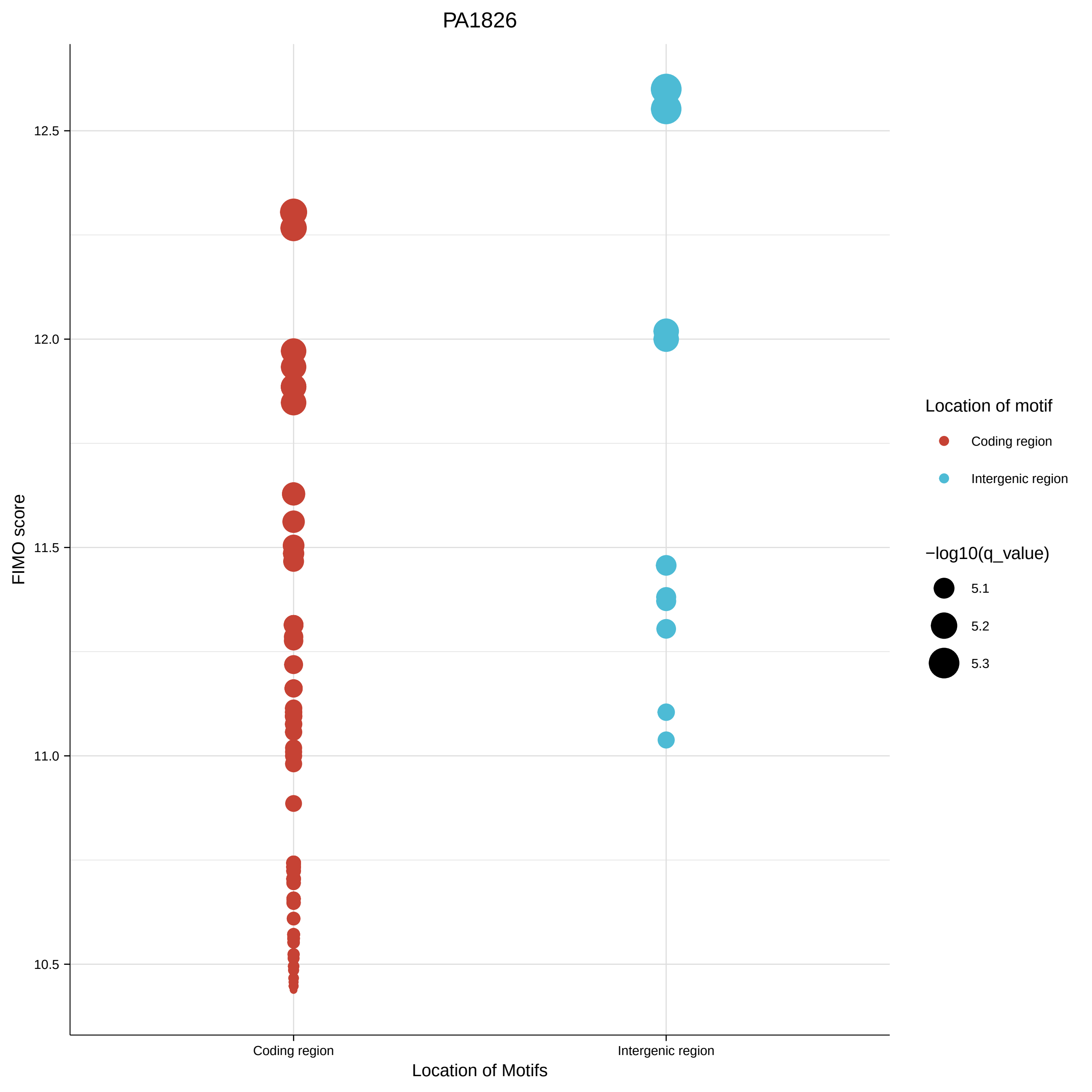

PA1826
FIMO score
12.5
12.0
11.5
11.0
10.5
Coding region
Intergenic region
Location of Motifs
Location of motif
Coding region
Intergenic region
−log10(q_value)
5.1
5.2
5.3

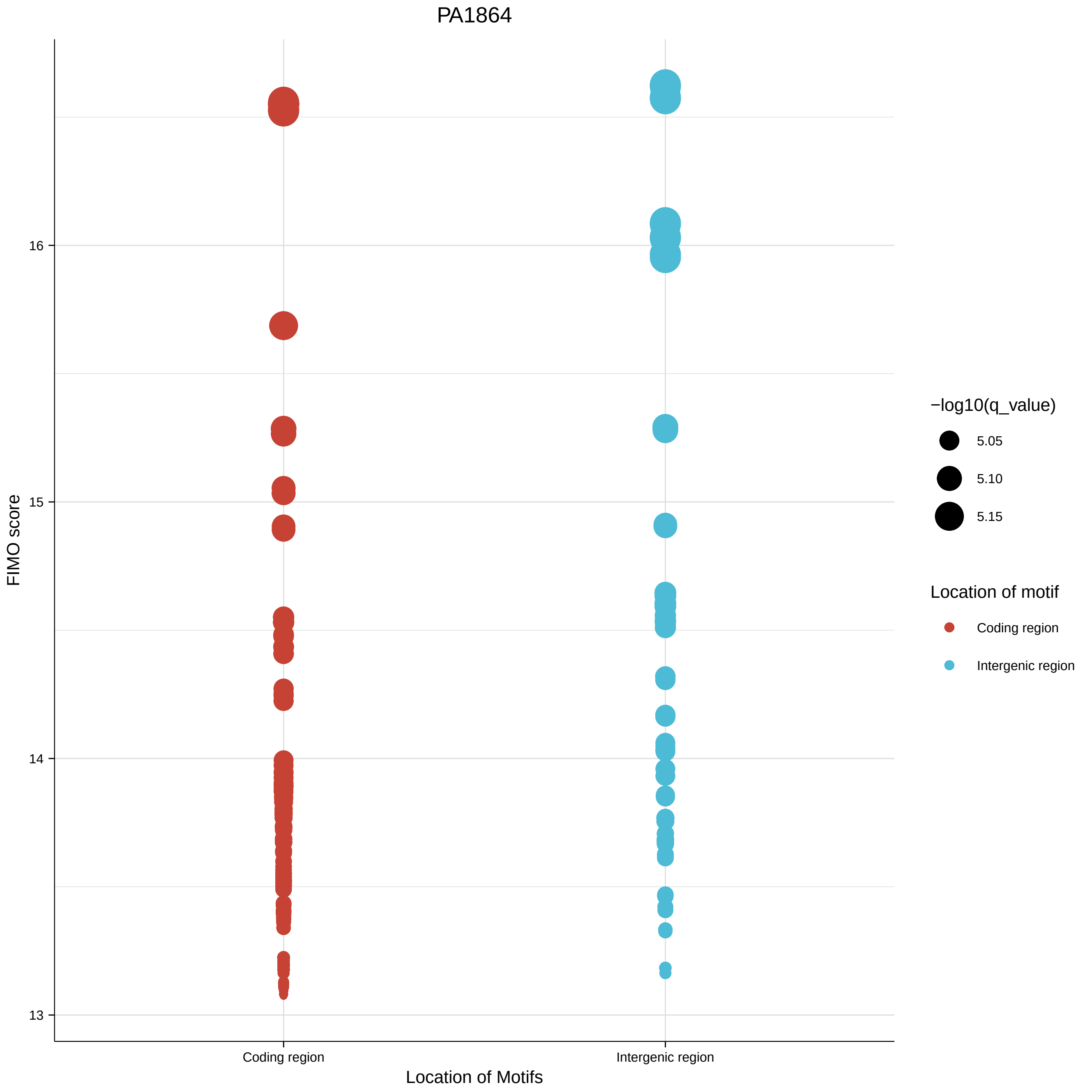
PA1864
FIMO score
16
15
14
13
Coding region
Intergenic region
Location of Motifs
−log10(q_value)
5.05
5.10
5.15
Location of motif
Coding region
Intergenic region

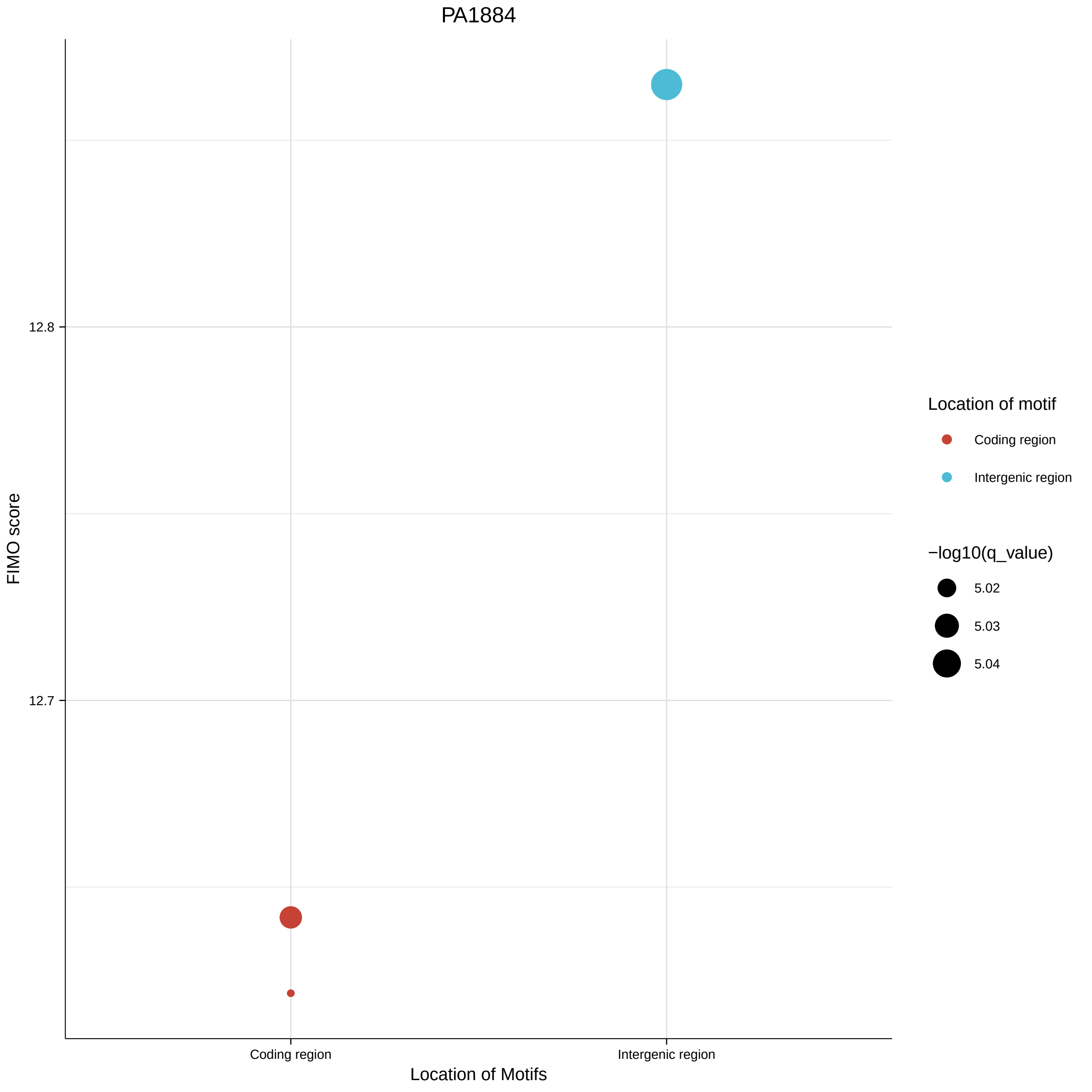

PA1884
FIMO score
12.8
12.7
Coding region
Intergenic region
Location of Motifs
Location of motif
Coding region
Intergenic region
−log10(q_value)
5.02
5.03
5.04

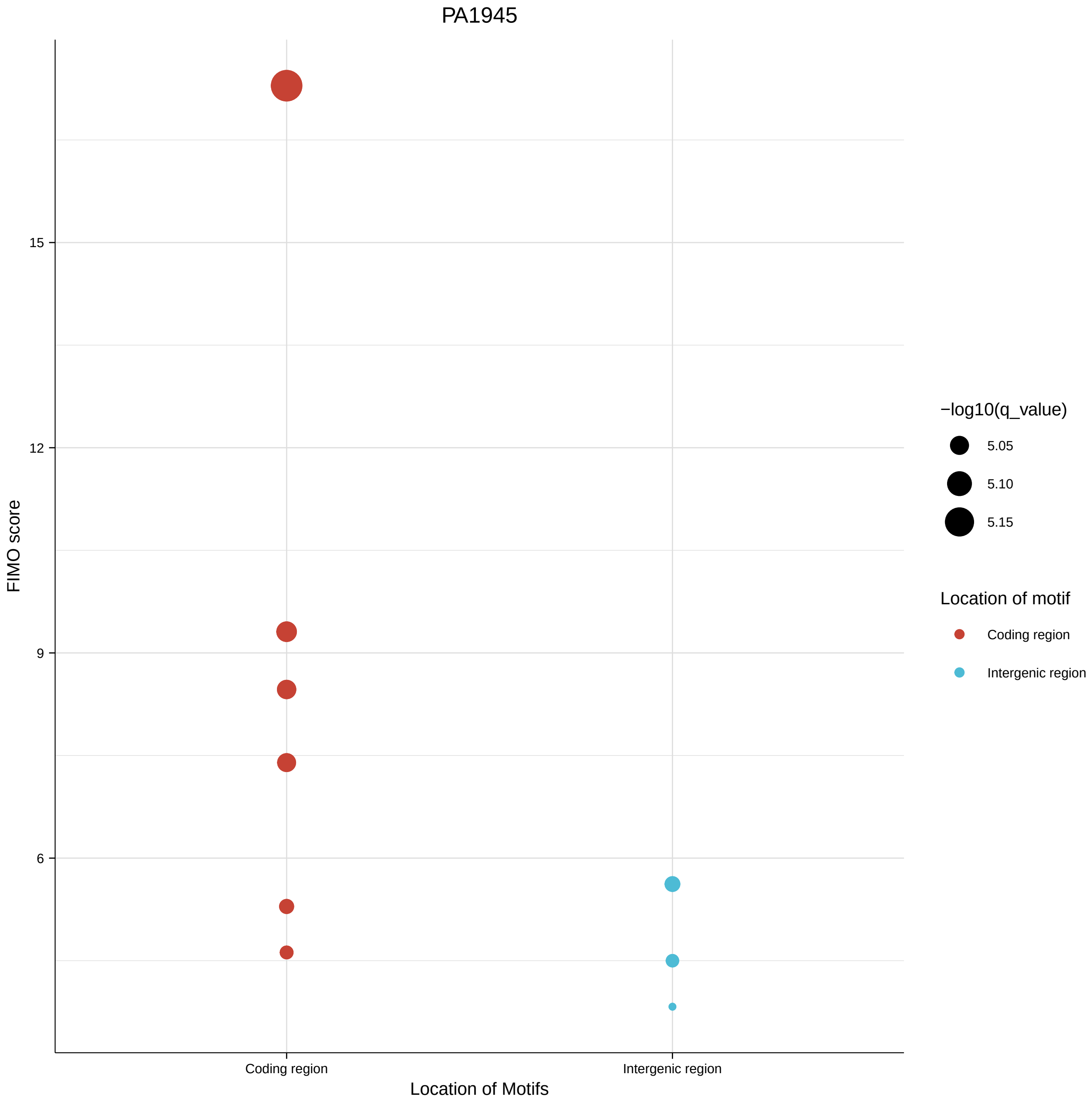
PA1945
FIMO score
15
12
9
6
Coding region
Intergenic region
Location of Motifs
−log10(q_value)
5.05
5.10
5.15
Location of motif
Coding region
Intergenic region

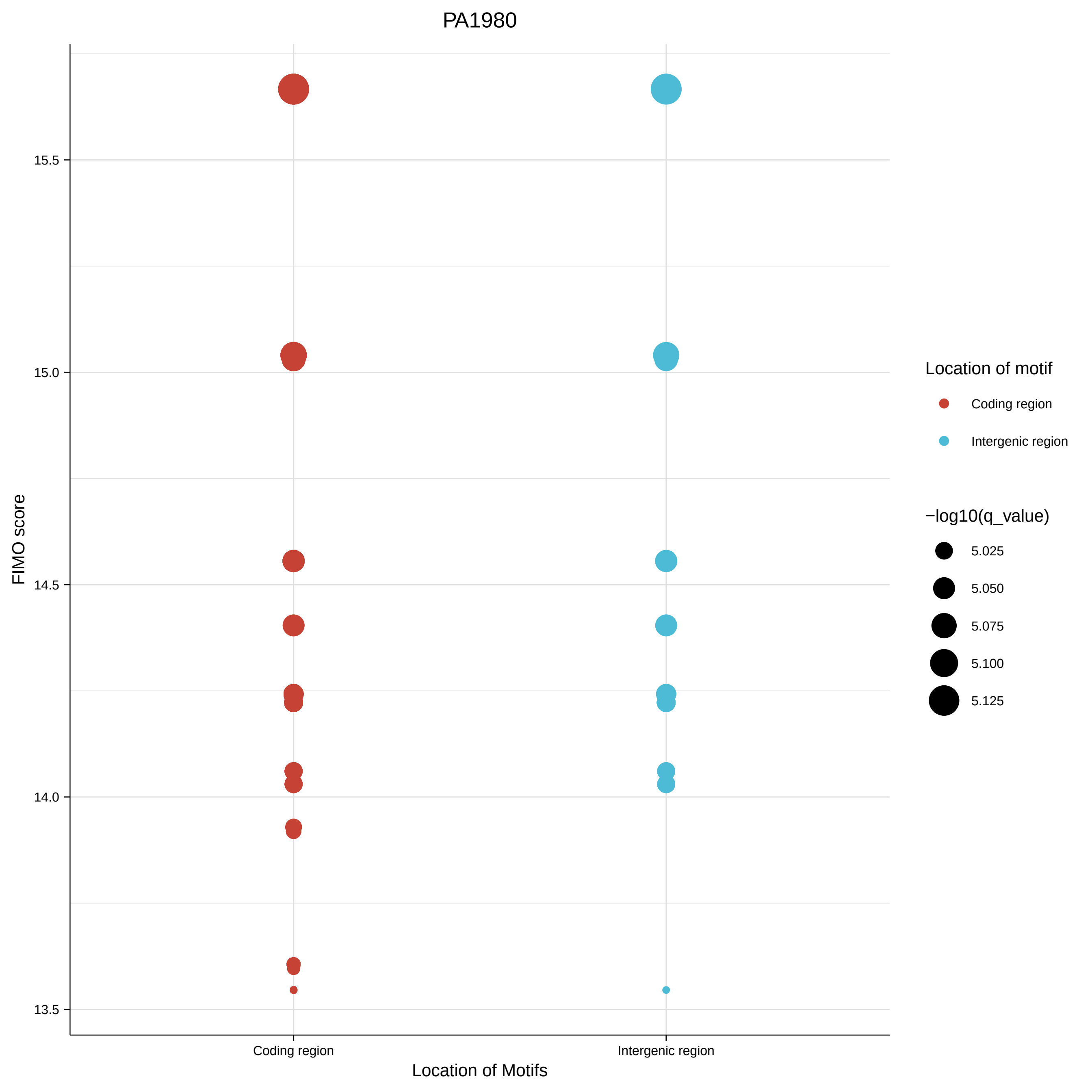
PA1980
FIMO score
15.5
15.0
14.5
14.0
13.5
Coding region
Intergenic region
Location of Motifs
Location of motif
Coding region
Intergenic region
−log10(q_value)
5.025
5.050
5.075
5.100
5.125

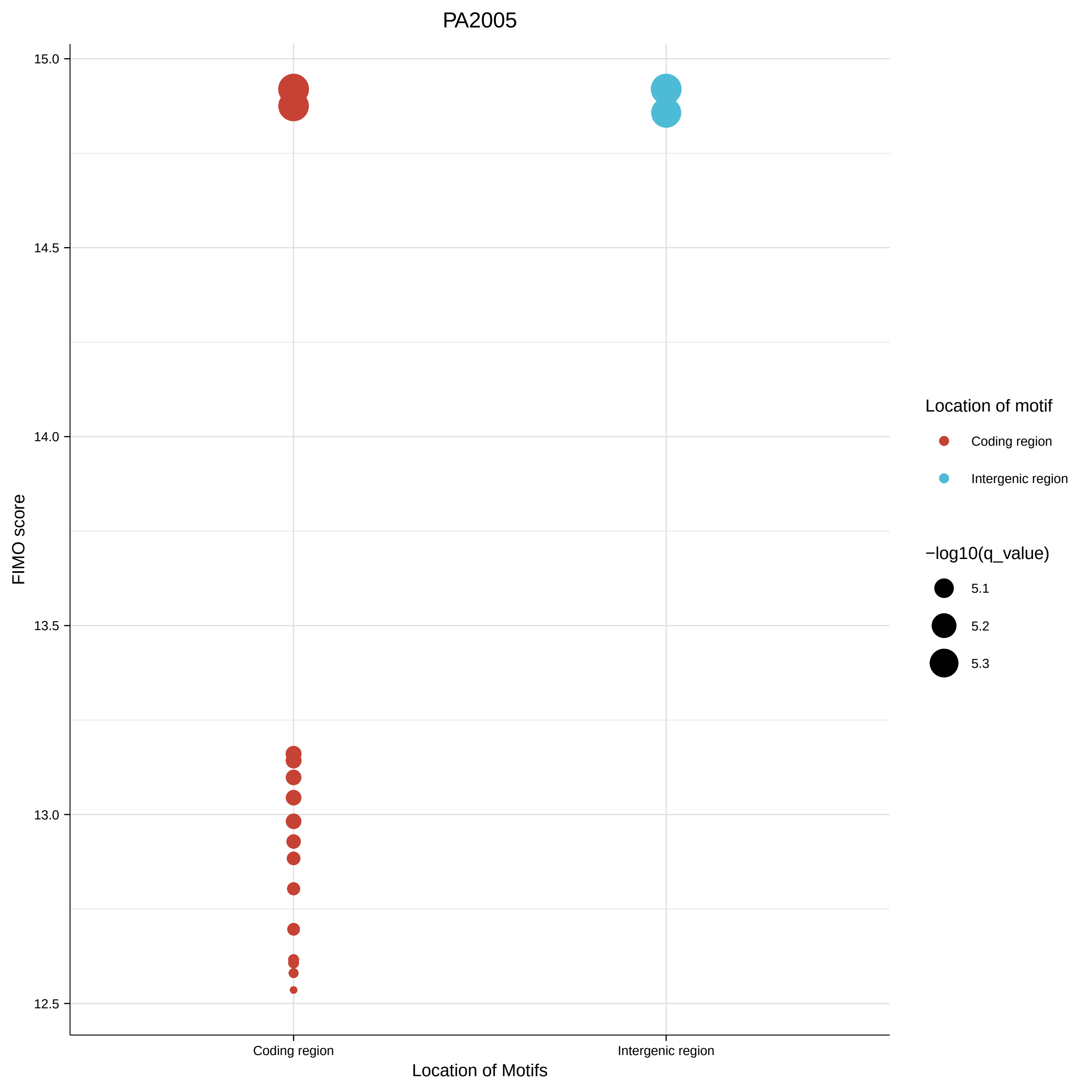
PA2005
FIMO score
15.0
14.5
14.0
13.5
13.0
12.5
Coding region
Intergenic region
Location of Motifs
Location of motif
Coding region
Intergenic region
−log10(q_value)
5.1
5.2
5.3

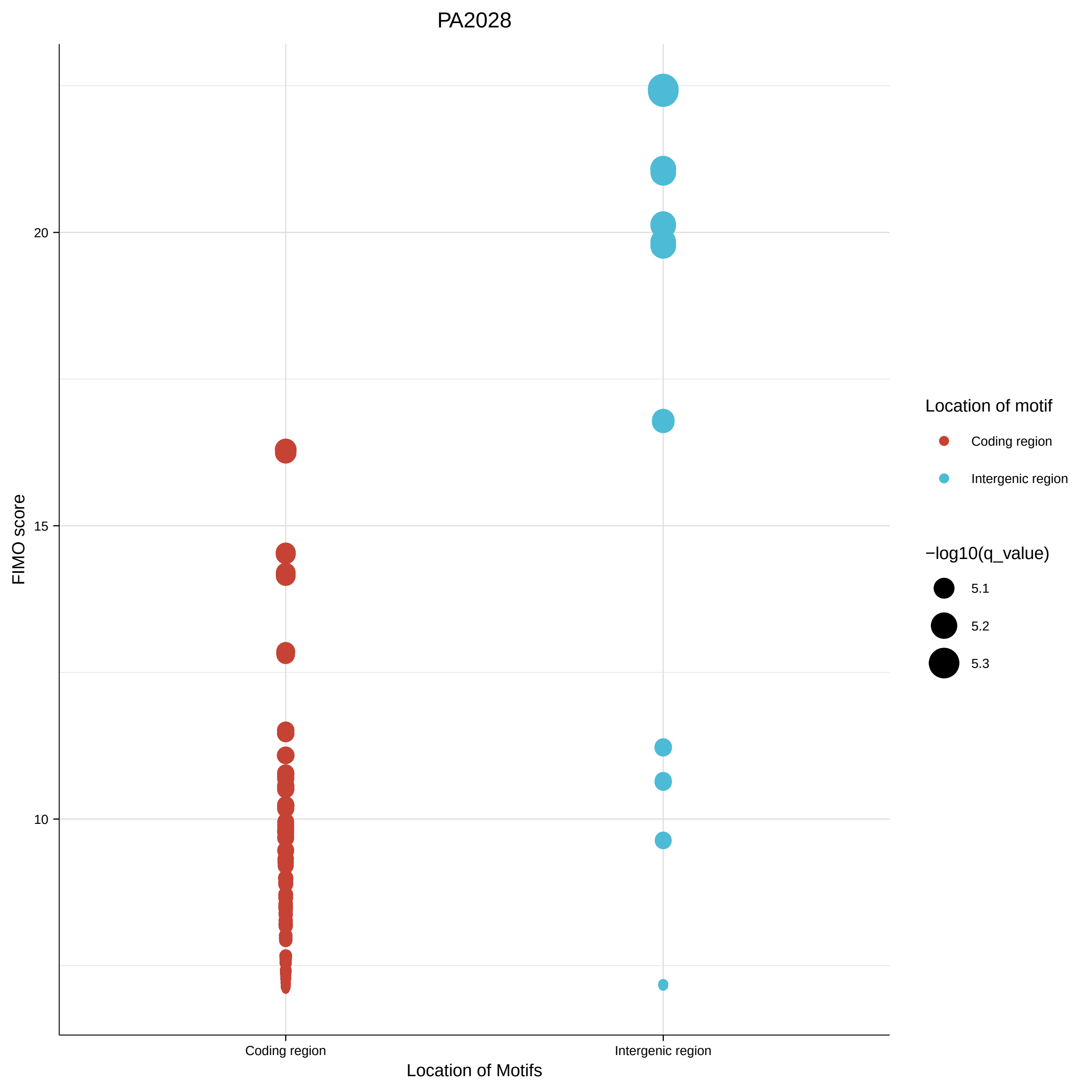

PA2028
FIMO score
20
15
10
Coding region
Intergenic region
Location of Motifs
Location of motif
Coding region
Intergenic region
−log10(q_value)
5.1
5.2
5.3

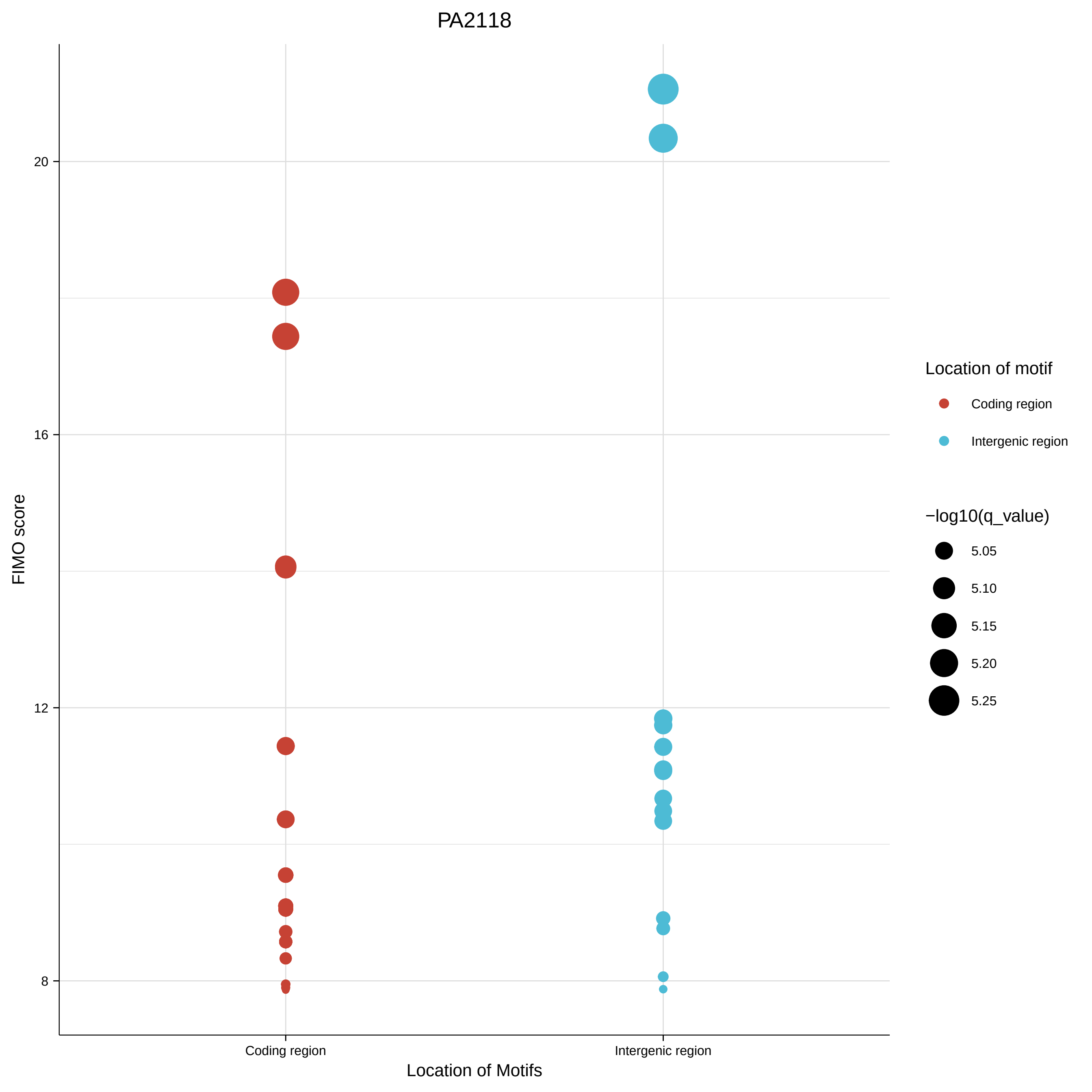
PA2118
FIMO score
20
16
12
8
Coding region
Intergenic region
Location of Motifs
Location of motif
Coding region
Intergenic region
−log10(q_value)
5.05
5.10
5.15
5.20
5.25

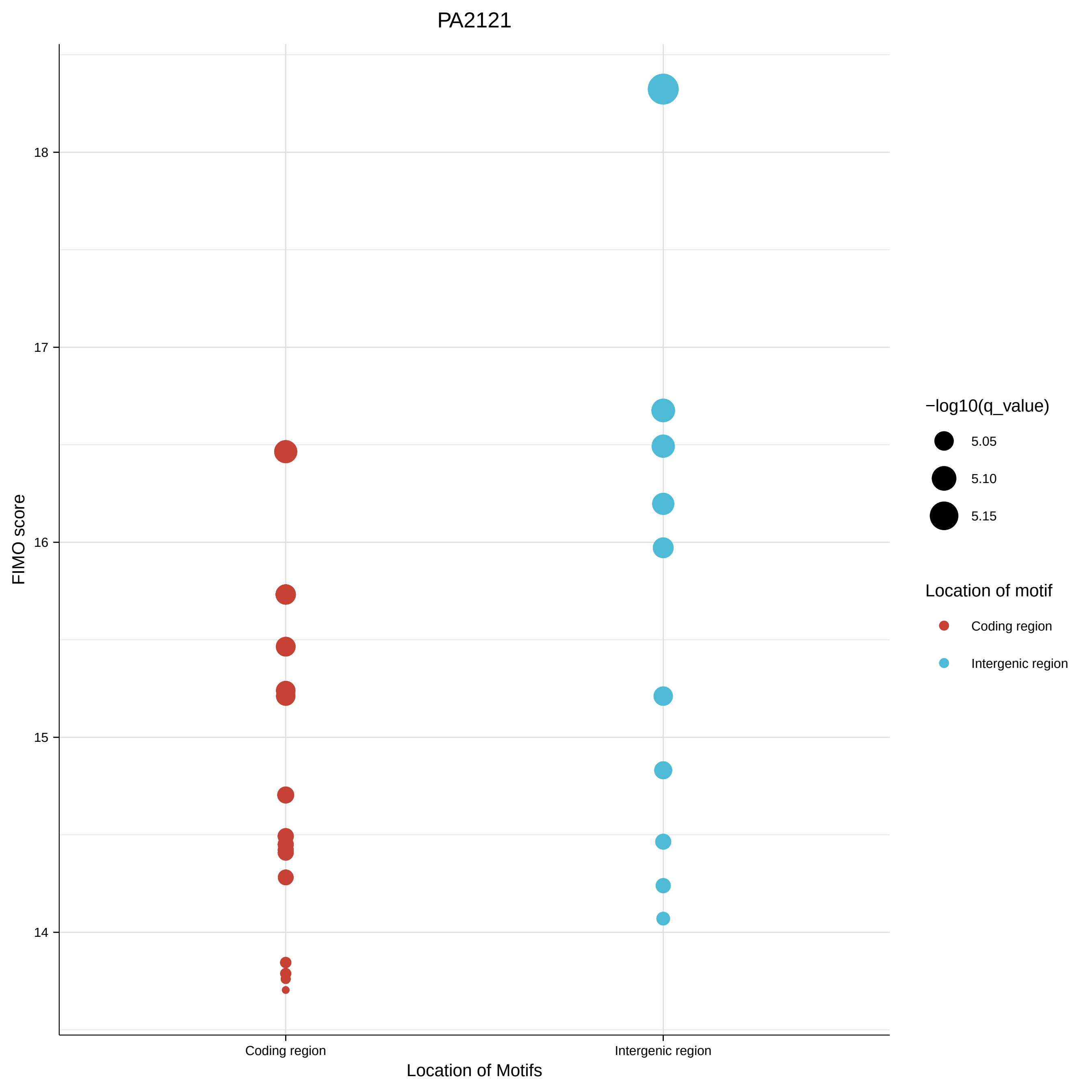
PA2121
FIMO score
18
17
16
15
14
Coding region
Intergenic region
Location of Motifs
−log10(q_value)
5.05
5.10
5.15
Location of motif
Coding region
Intergenic region

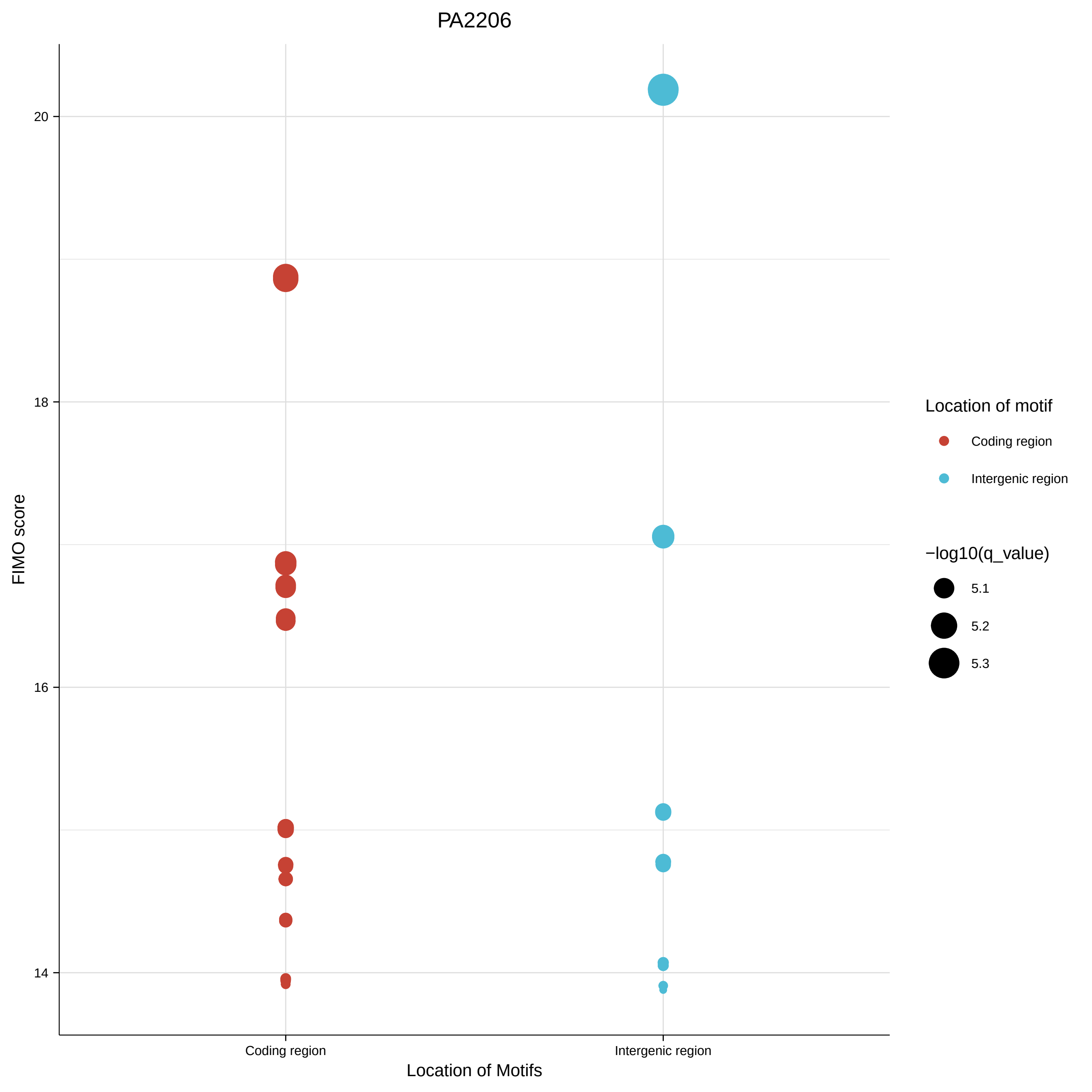

PA2206
FIMO score
20
18
16
14
Coding region
Intergenic region
Location of Motifs
Location of motif
Coding region
Intergenic region
−log10(q_value)
5.1
5.2
5.3

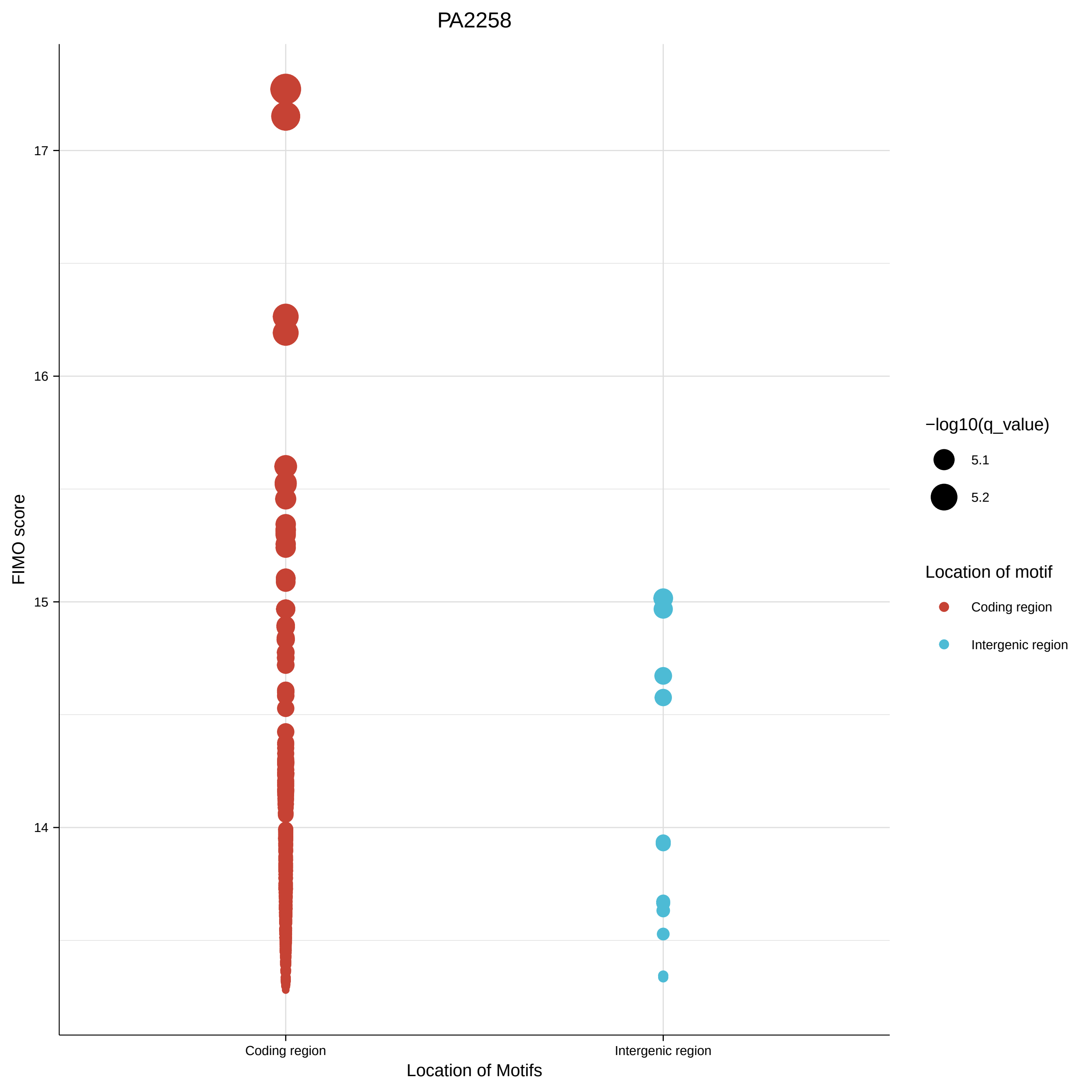
PA2258
FIMO score
17
16
15
14
Coding region
Intergenic region
Location of Motifs
−log10(q_value)
5.1
5.2
Location of motif
Coding region
Intergenic region

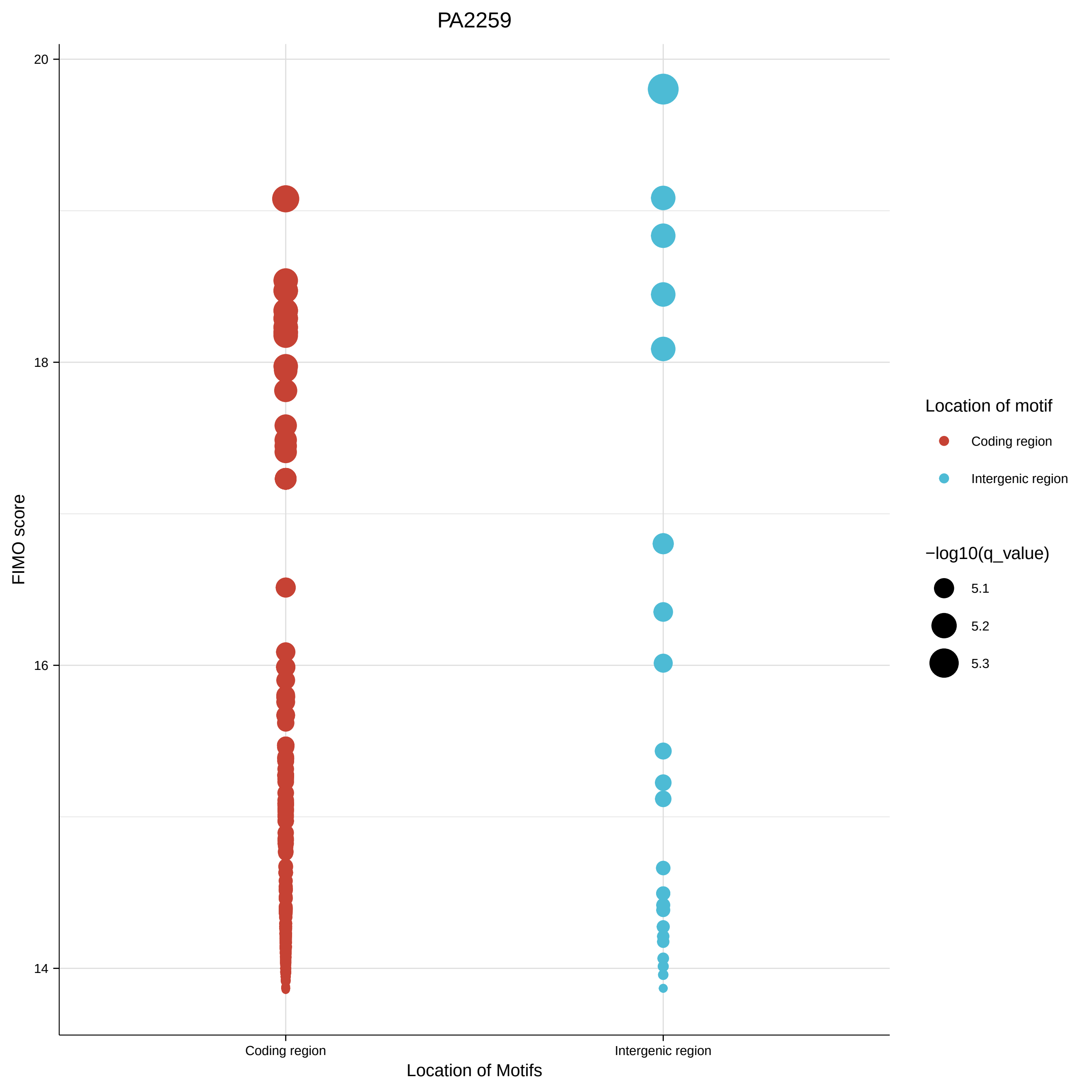
PA2259
FIMO score
20
18
16
14
Coding region
Intergenic region
Location of Motifs
Location of motif
Coding region
Intergenic region
−log10(q_value)
5.1
5.2
5.3

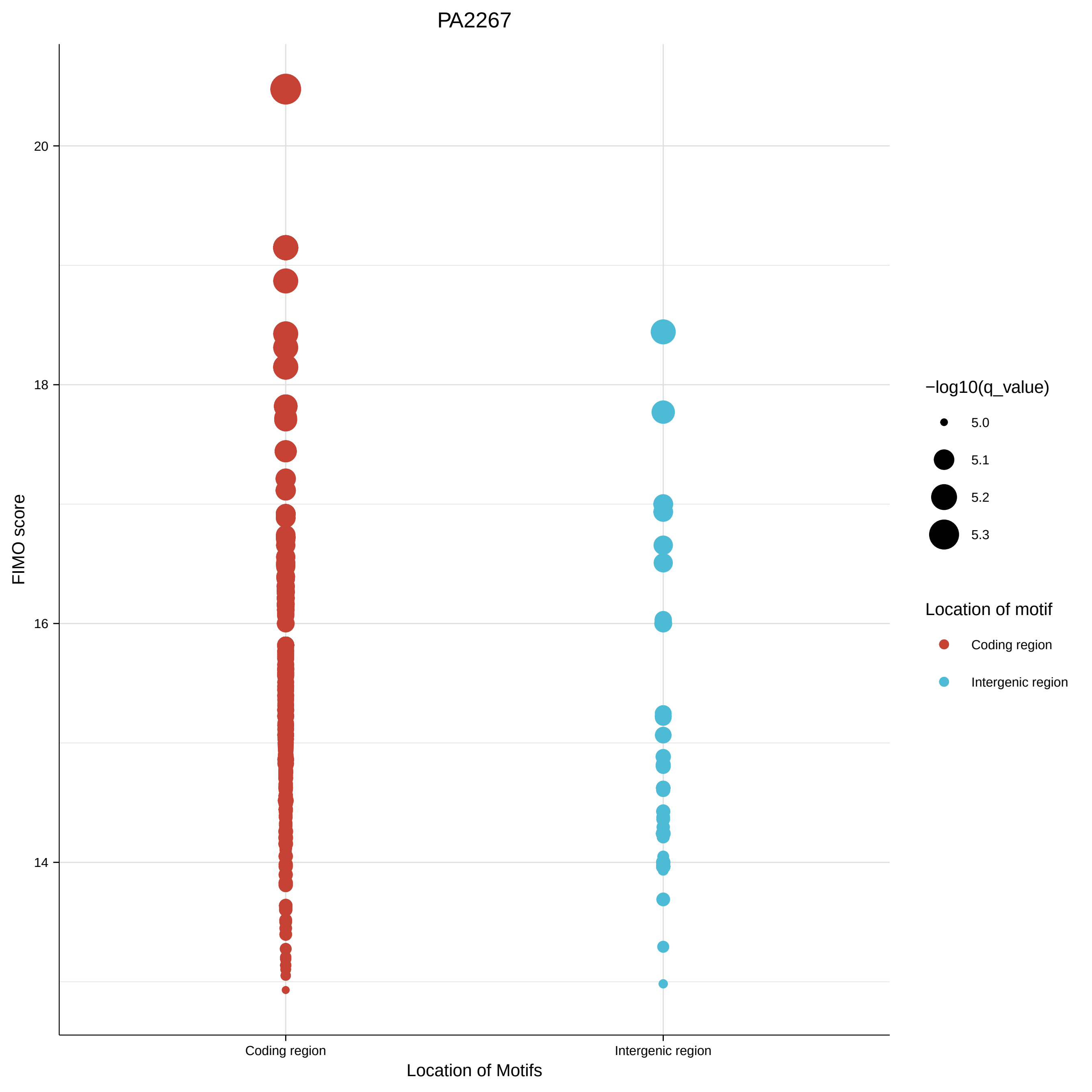

PA2267
FIMO score
20
18
16
14
Coding region
Intergenic region
Location of Motifs
−log10(q_value)
5.0
5.1
5.2
5.3
Location of motif
Coding region
Intergenic region

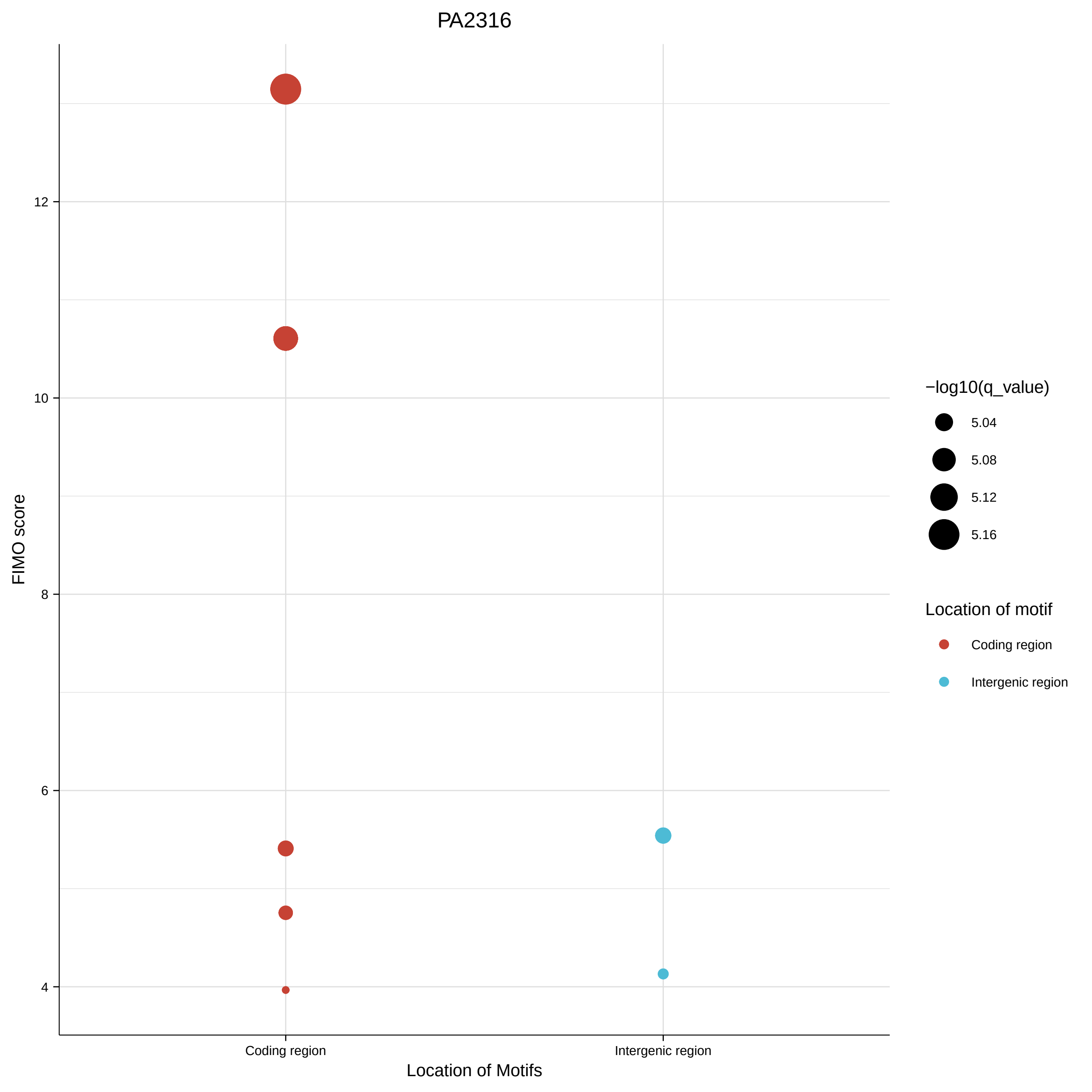
PA2316
FIMO score
12
10
8
6
4
Coding region
Intergenic region
Location of Motifs
−log10(q_value)
5.04
5.08
5.12
5.16
Location of motif
Coding region
Intergenic region

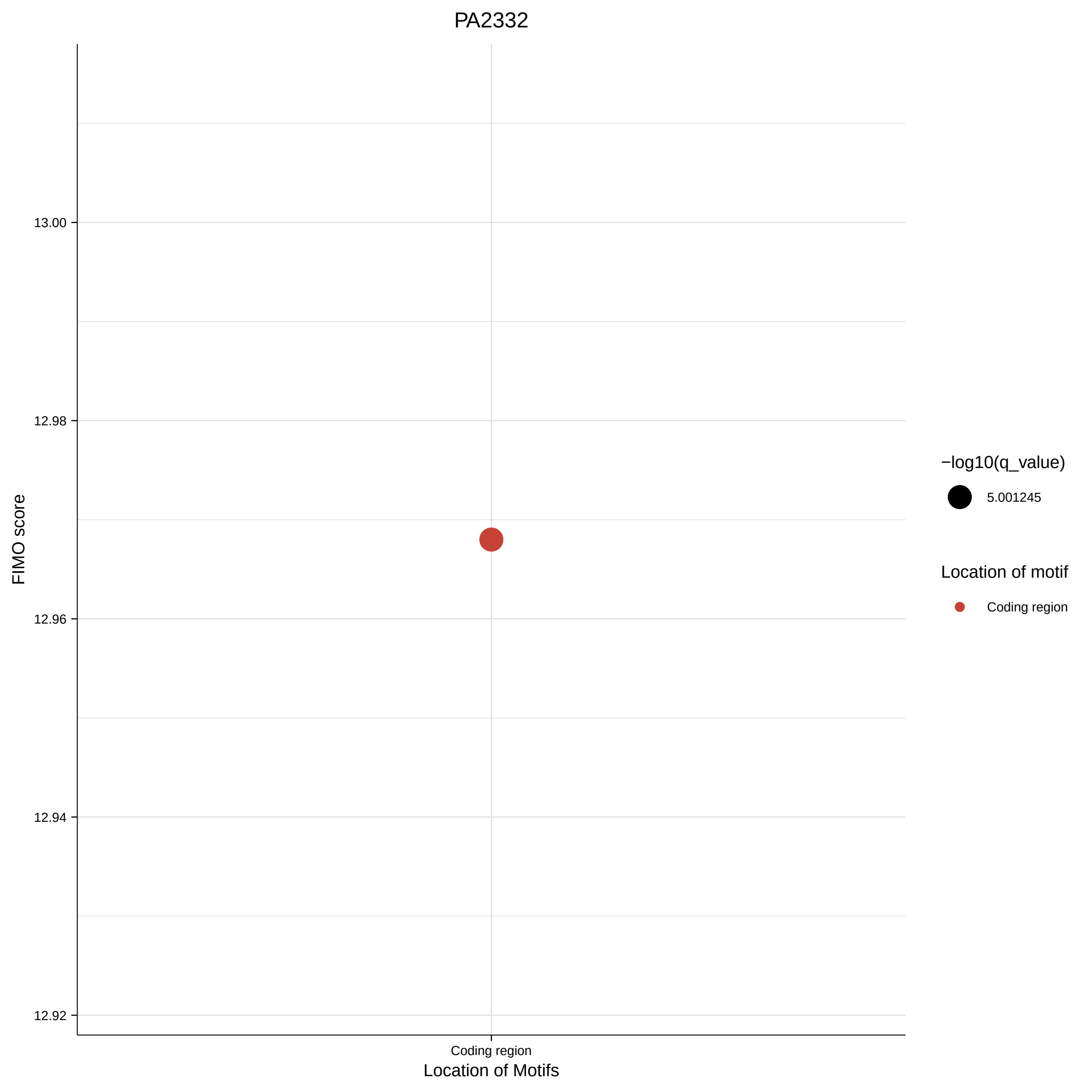
PA2332
FIMO score
13.00
12.98
12.96
12.94
12.92
Coding region
Location of Motifs
−log10(q_value)
5.001245
Location of motif
Coding region

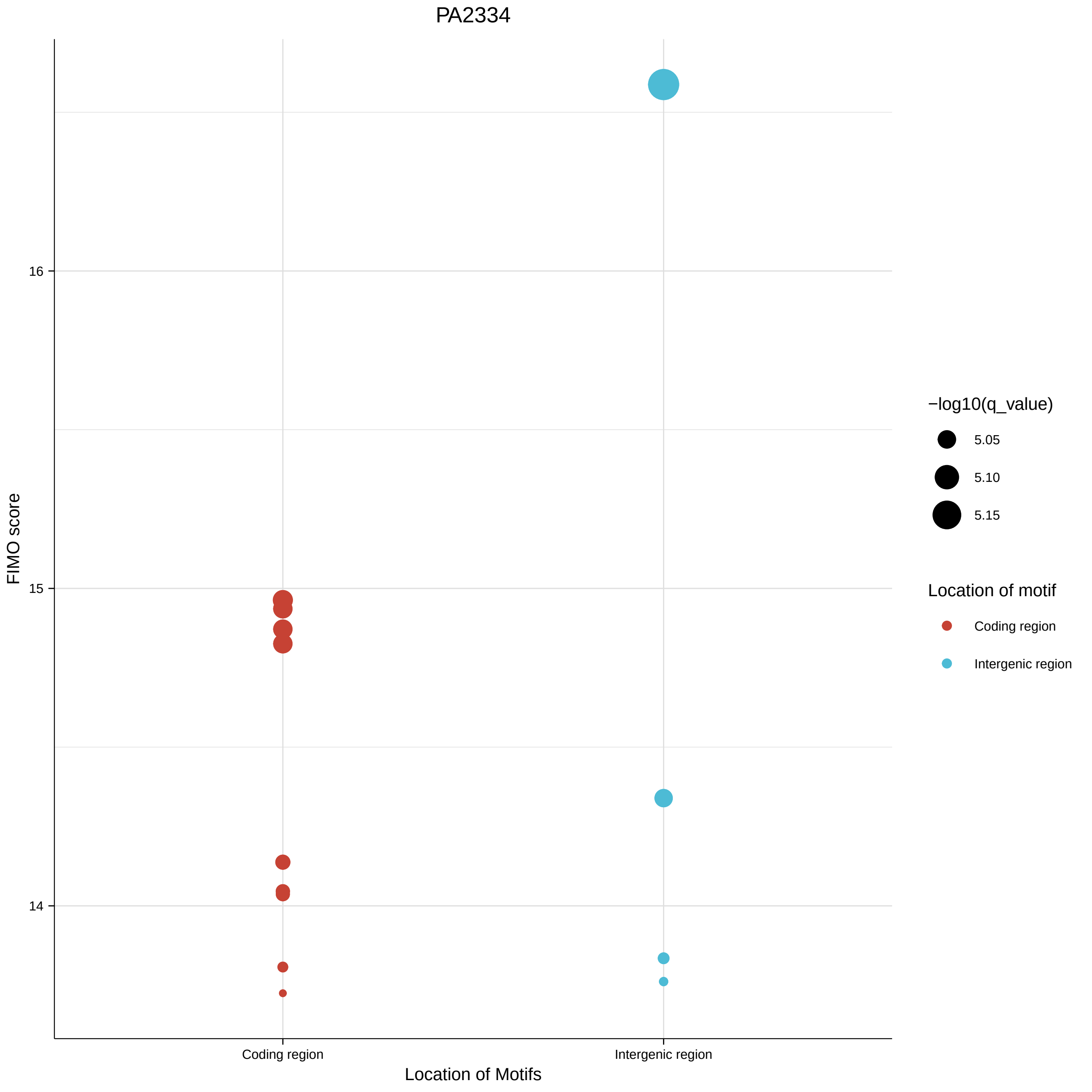

PA2334
FIMO score
16
15
14
Coding region
Intergenic region
Location of Motifs
−log10(q_value)
5.05
5.10
5.15
Location of motif
Coding region
Intergenic region

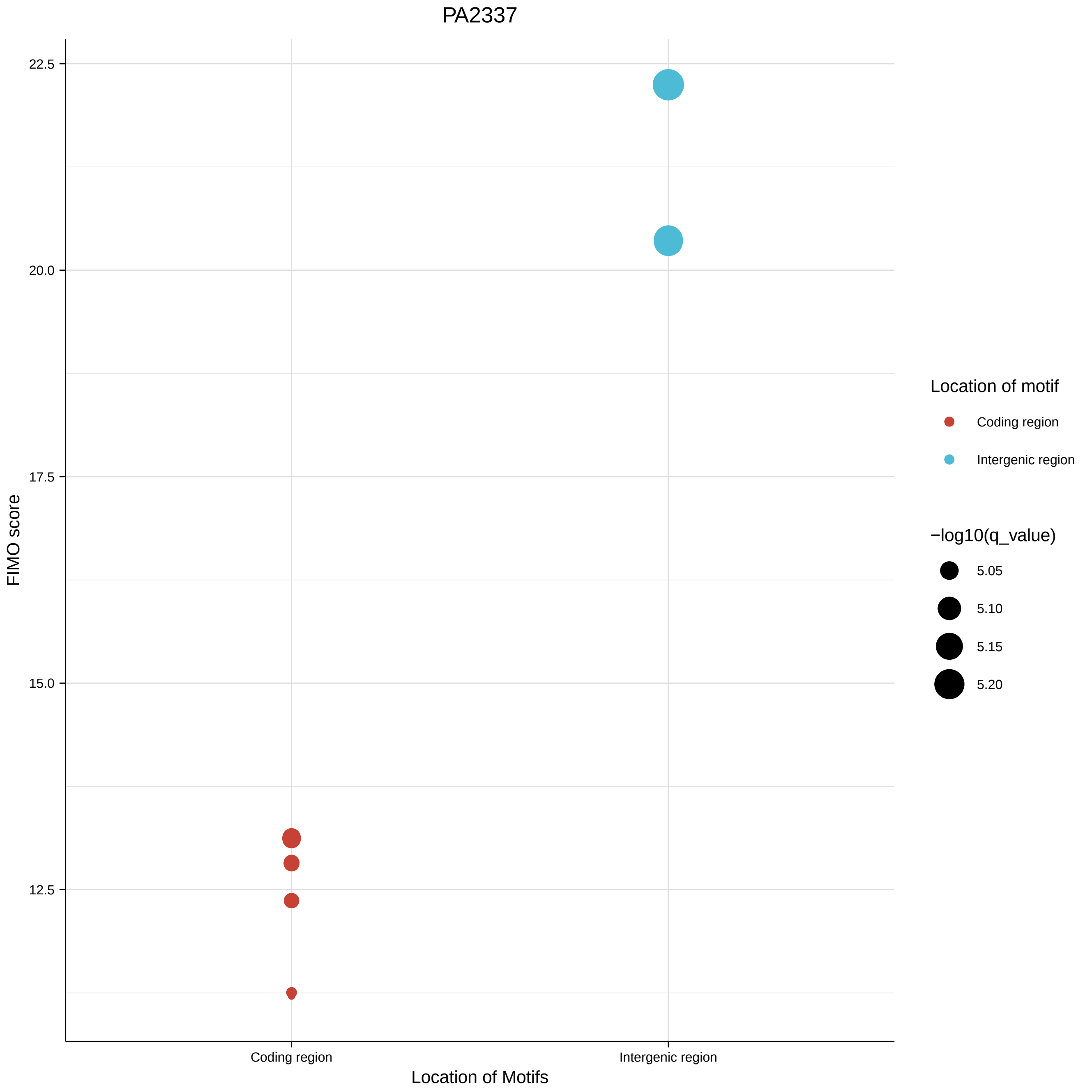
PA2337
FIMO score
22.5
20.0
17.5
15.0
12.5
Coding region
Intergenic region
Location of Motifs
Location of motif
Coding region
Intergenic region
−log10(q_value)
5.05
5.10
5.15
5.20

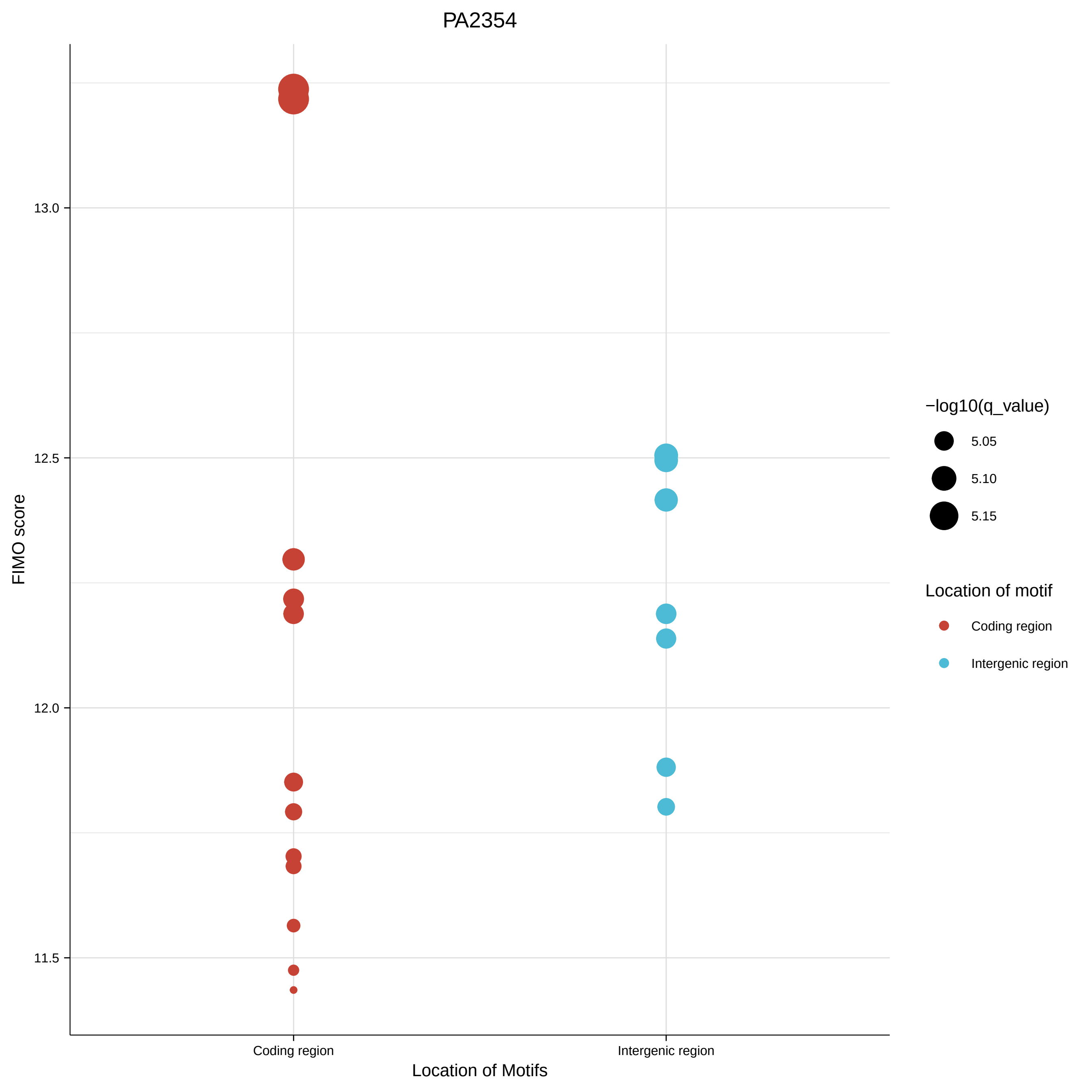

PA2354
FIMO score
13.0
12.5
12.0
11.5
Coding region
Intergenic region
Location of Motifs
−log10(q_value)
5.05
5.10
5.15
Location of motif
Coding region
Intergenic region

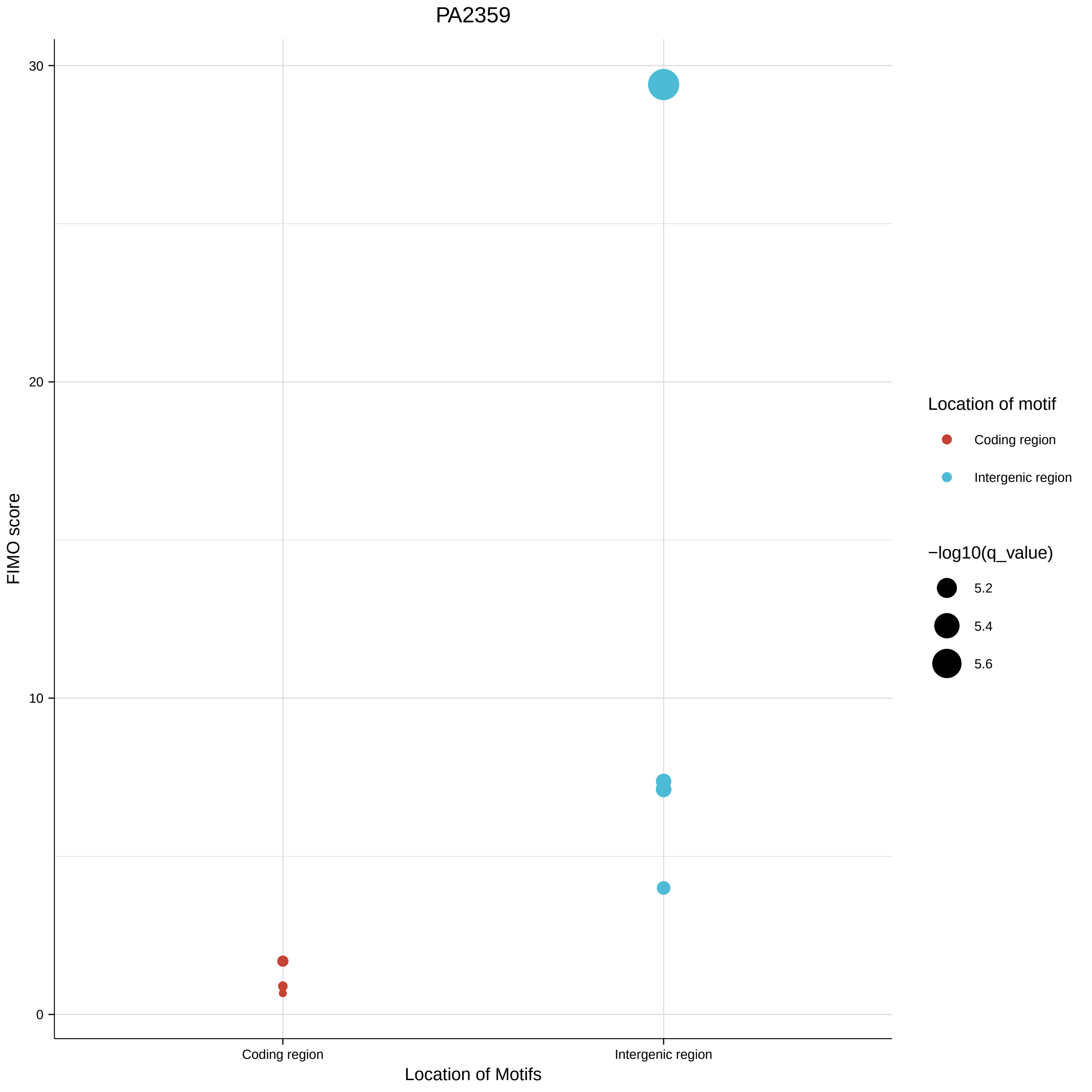

PA2359
FIMO score
30
20
10
0
Coding region
Intergenic region
Location of Motifs
Location of motif
Coding region
Intergenic region
−log10(q_value)
5.2
5.4
5.6

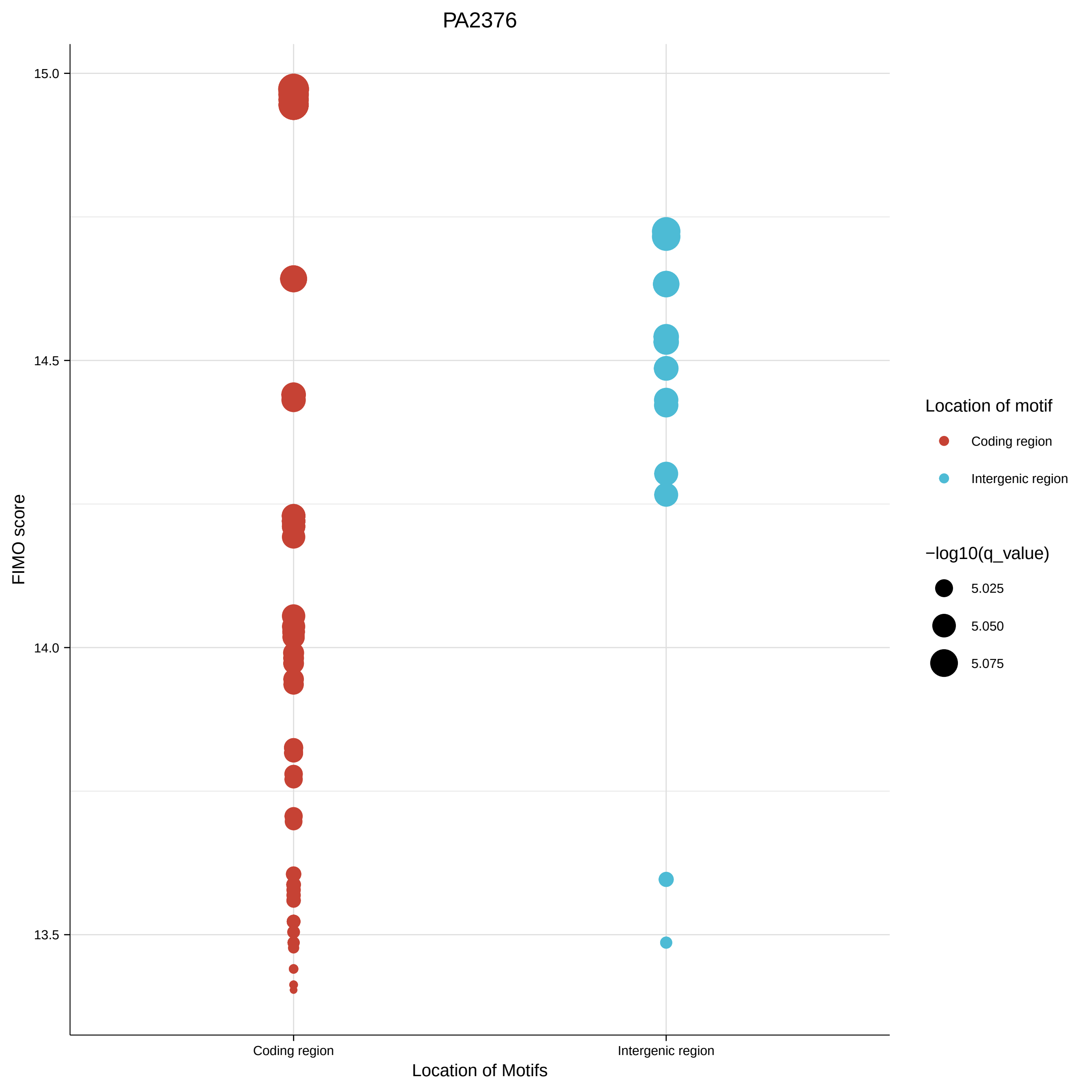

PA2376
FIMO score
15.0
14.5
14.0
13.5
Coding region
Intergenic region
Location of Motifs
Location of motif
Coding region
Intergenic region
−log10(q_value)
5.025
5.050
5.075

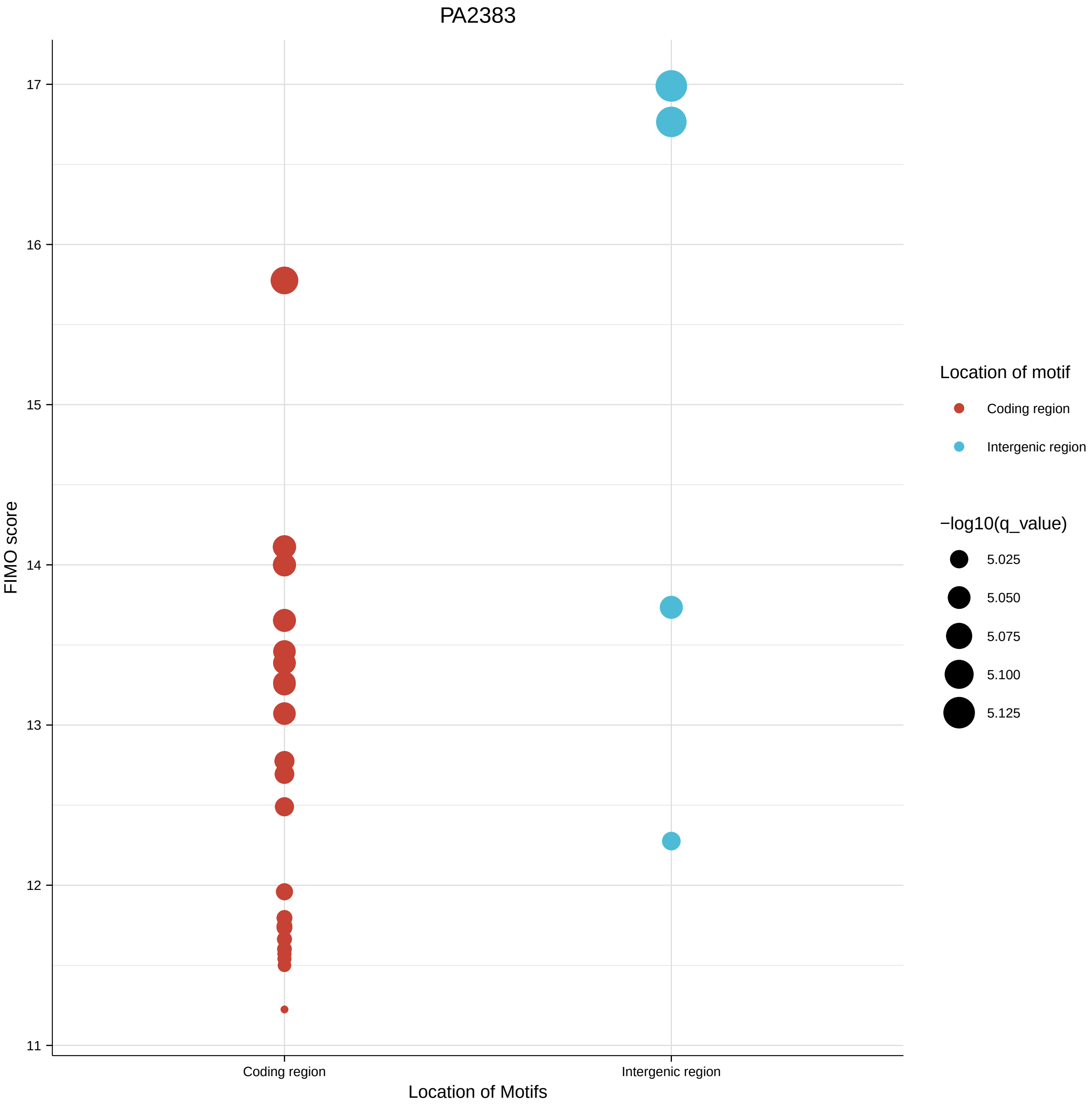
PA2383
FIMO score
17
16
15
14
13
12
11
Coding region
Intergenic region
Location of Motifs
Location of motif
Coding region
Intergenic region
−log10(q_value)
5.025
5.050
5.075
5.100
5.125

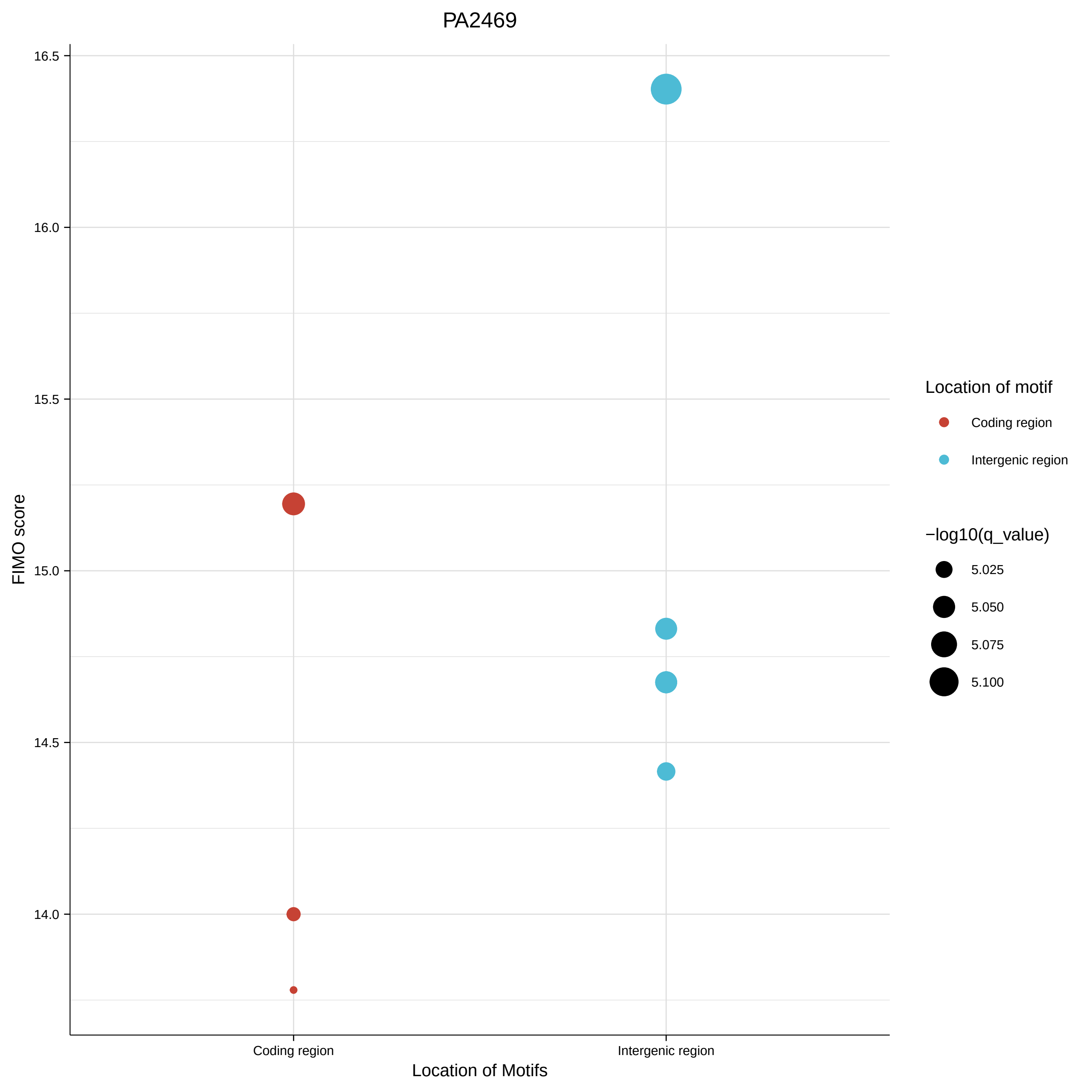
PA2469
FIMO score
16.5
16.0
15.5
15.0
14.5
14.0
Coding region
Intergenic region
Location of Motifs
Location of motif
Coding region
Intergenic region
−log10(q_value)
5.025
5.050
5.075
5.100

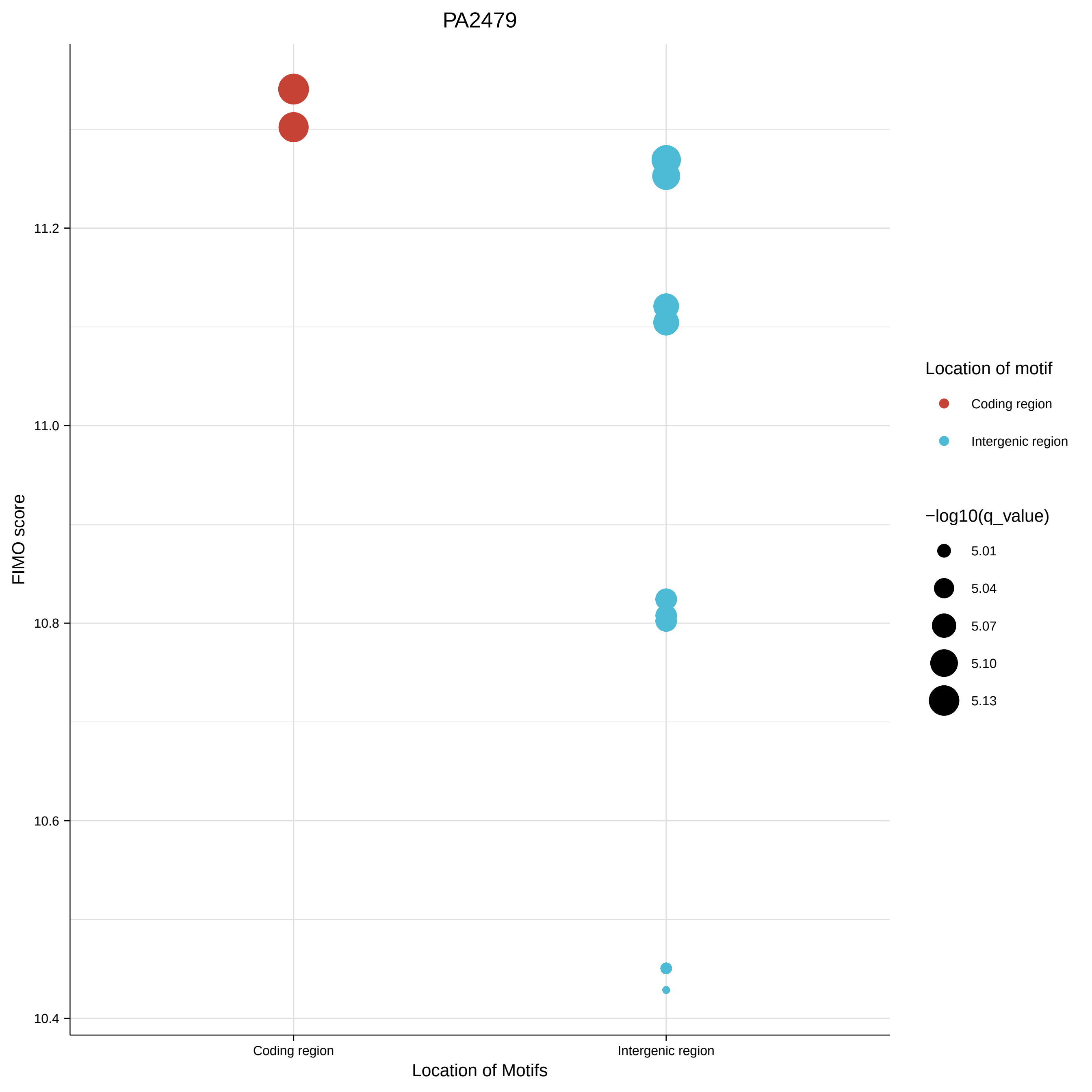
PA2479
FIMO score
11.2
11.0
10.8
10.6
10.4
Coding region
Intergenic region
Location of Motifs
Location of motif
Coding region
Intergenic region
−log10(q_value)
5.01
5.04
5.07
5.10
5.13

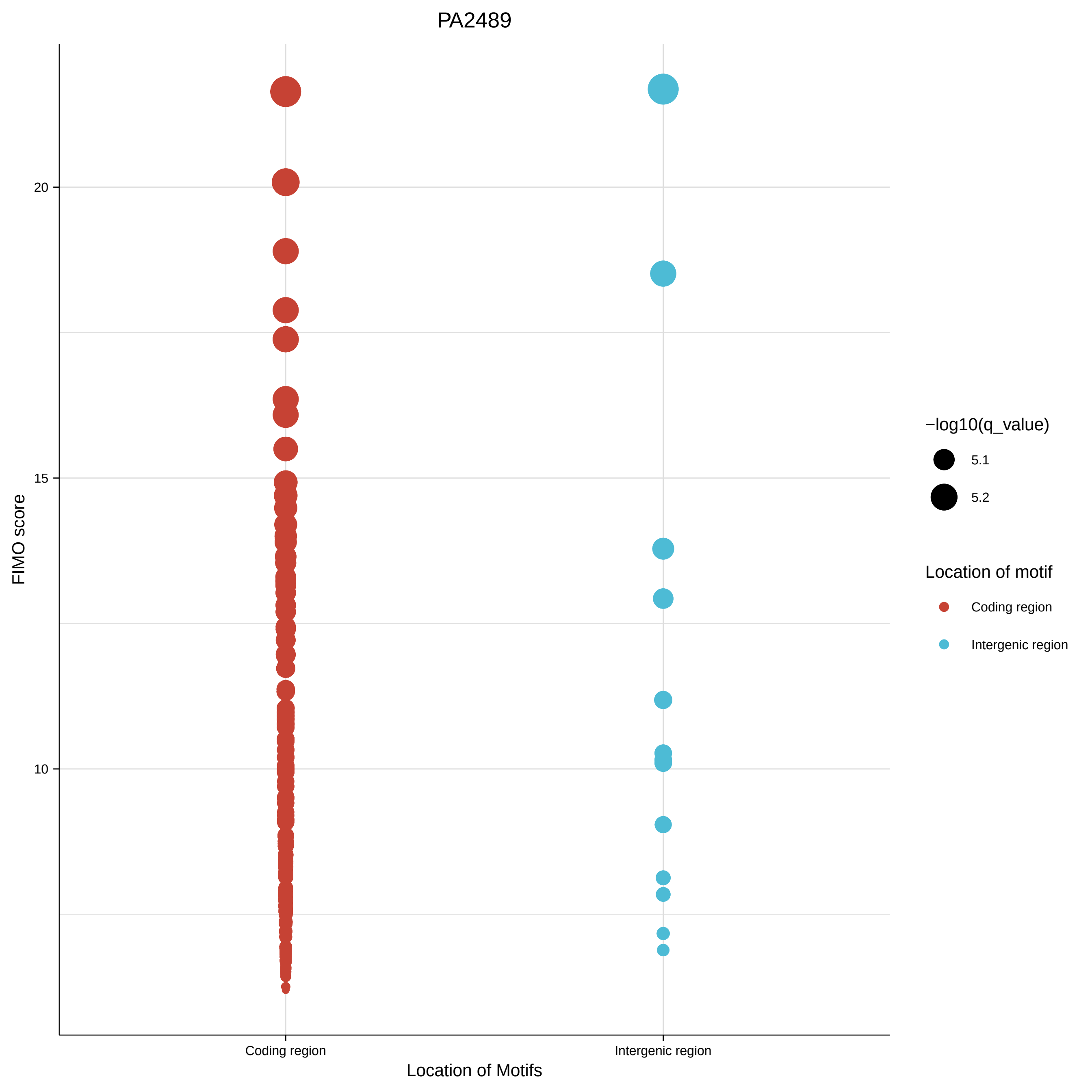
PA2489
FIMO score
20
15
10
Coding region
Intergenic region
Location of Motifs
−log10(q_value)
5.1
5.2
Location of motif
Coding region
Intergenic region

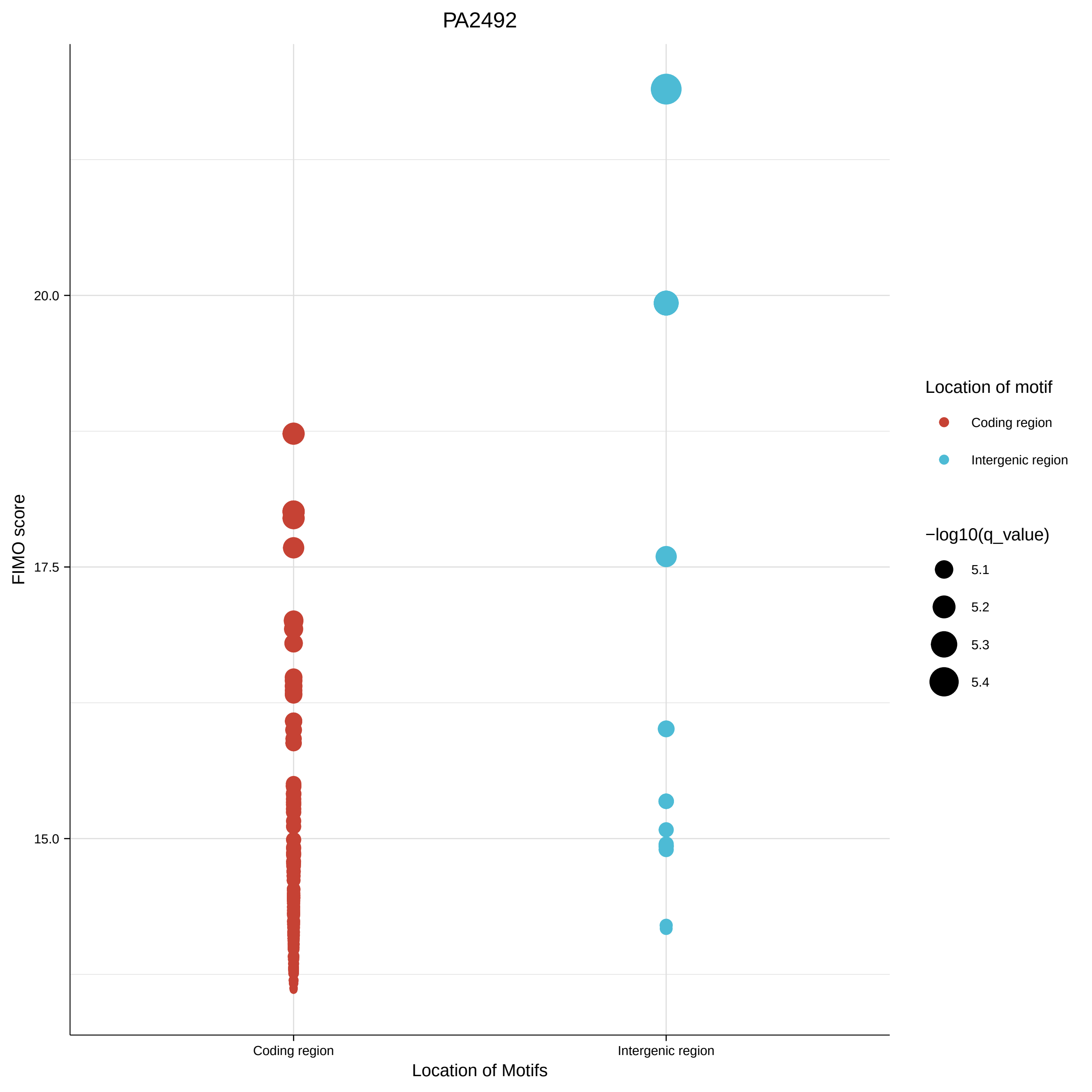
PA2492
FIMO score
20.0
17.5
15.0
Coding region
Intergenic region
Location of Motifs
Location of motif
Coding region
Intergenic region
−log10(q_value)
5.1
5.2
5.3
5.4

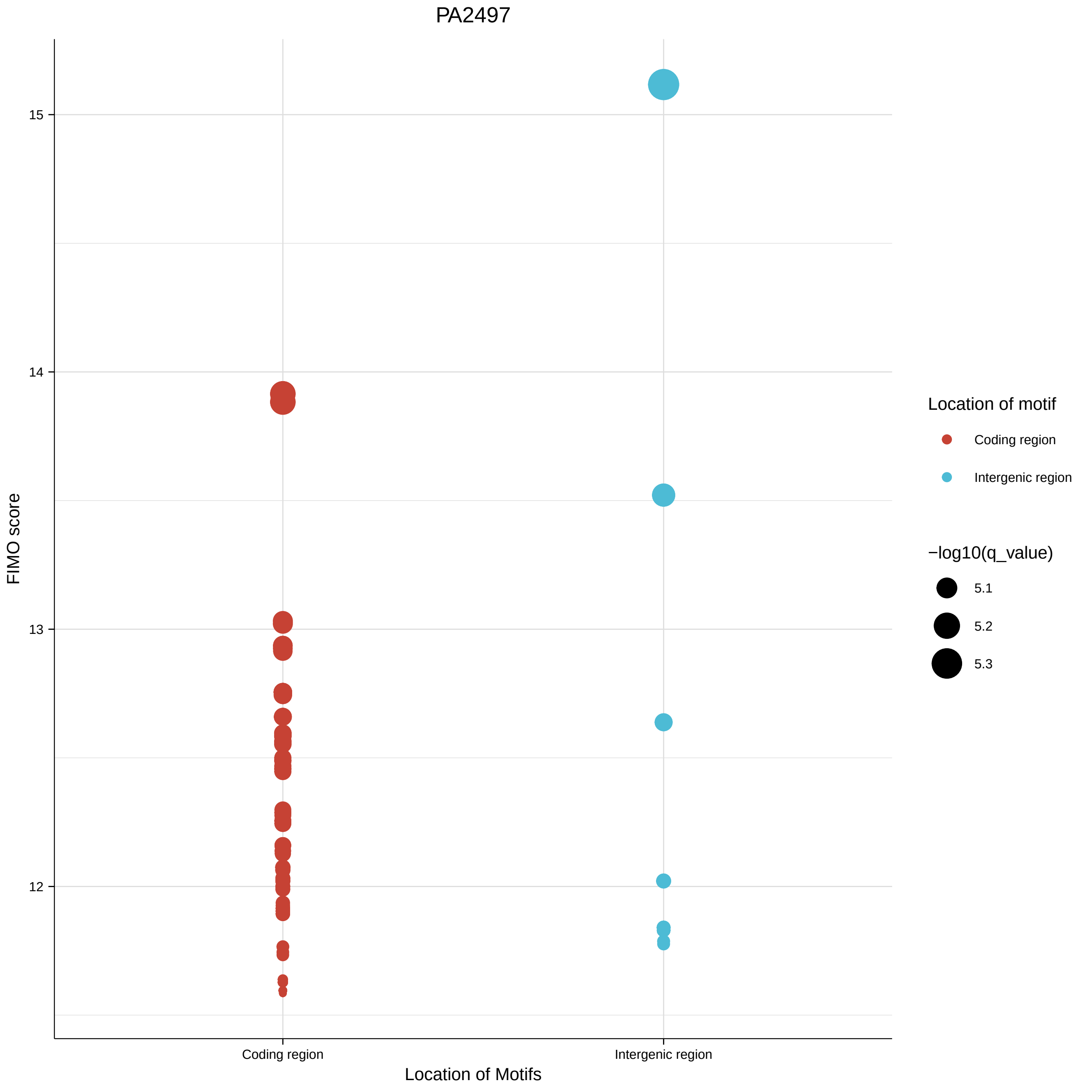
PA2497
FIMO score
15
14
13
12
Coding region
Intergenic region
Location of Motifs
Location of motif
Coding region
Intergenic region
−log10(q_value)
5.1
5.2
5.3

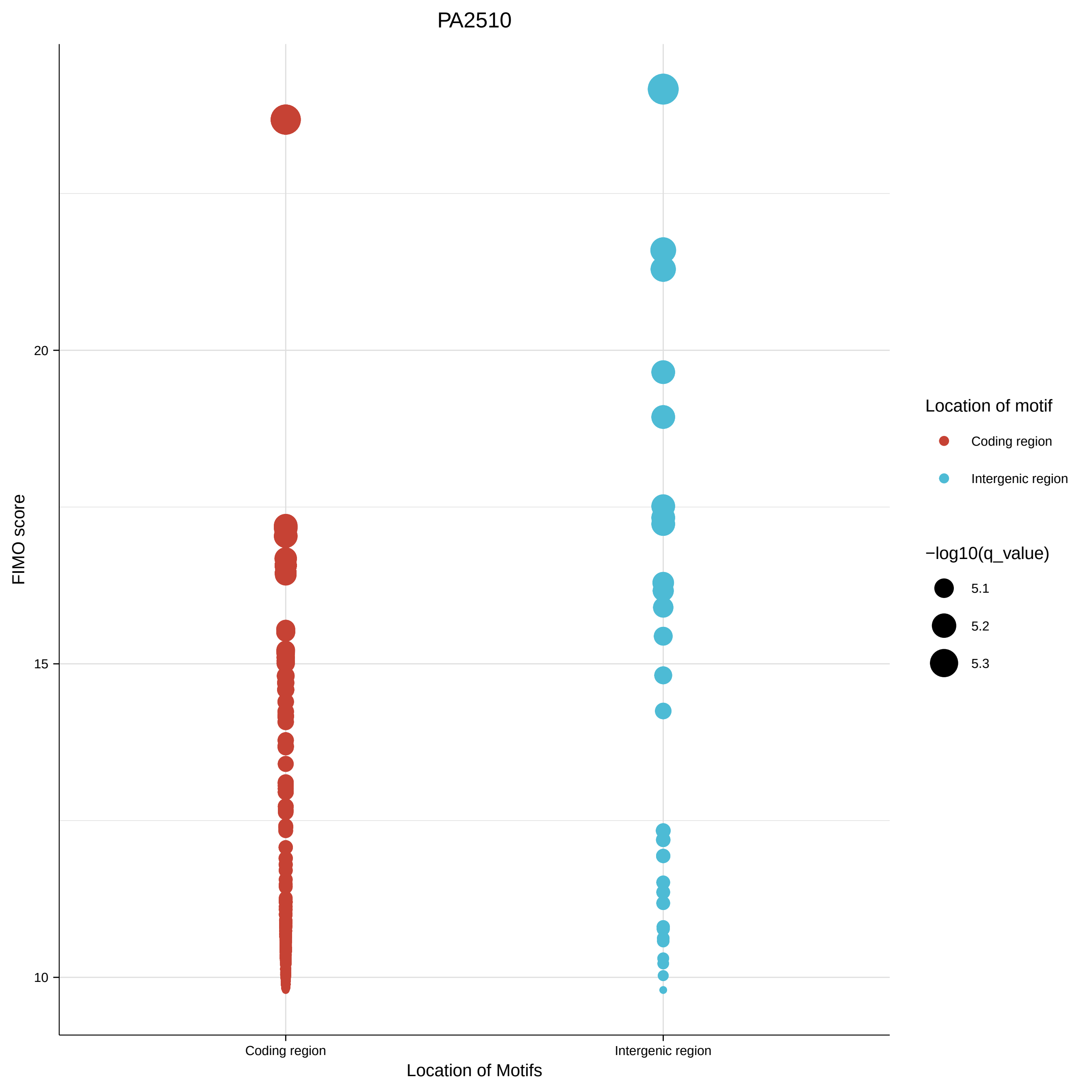

PA2510
FIMO score
20
15
10
Coding region
Intergenic region
Location of Motifs
Location of motif
Coding region
Intergenic region
−log10(q_value)
5.1
5.2
5.3

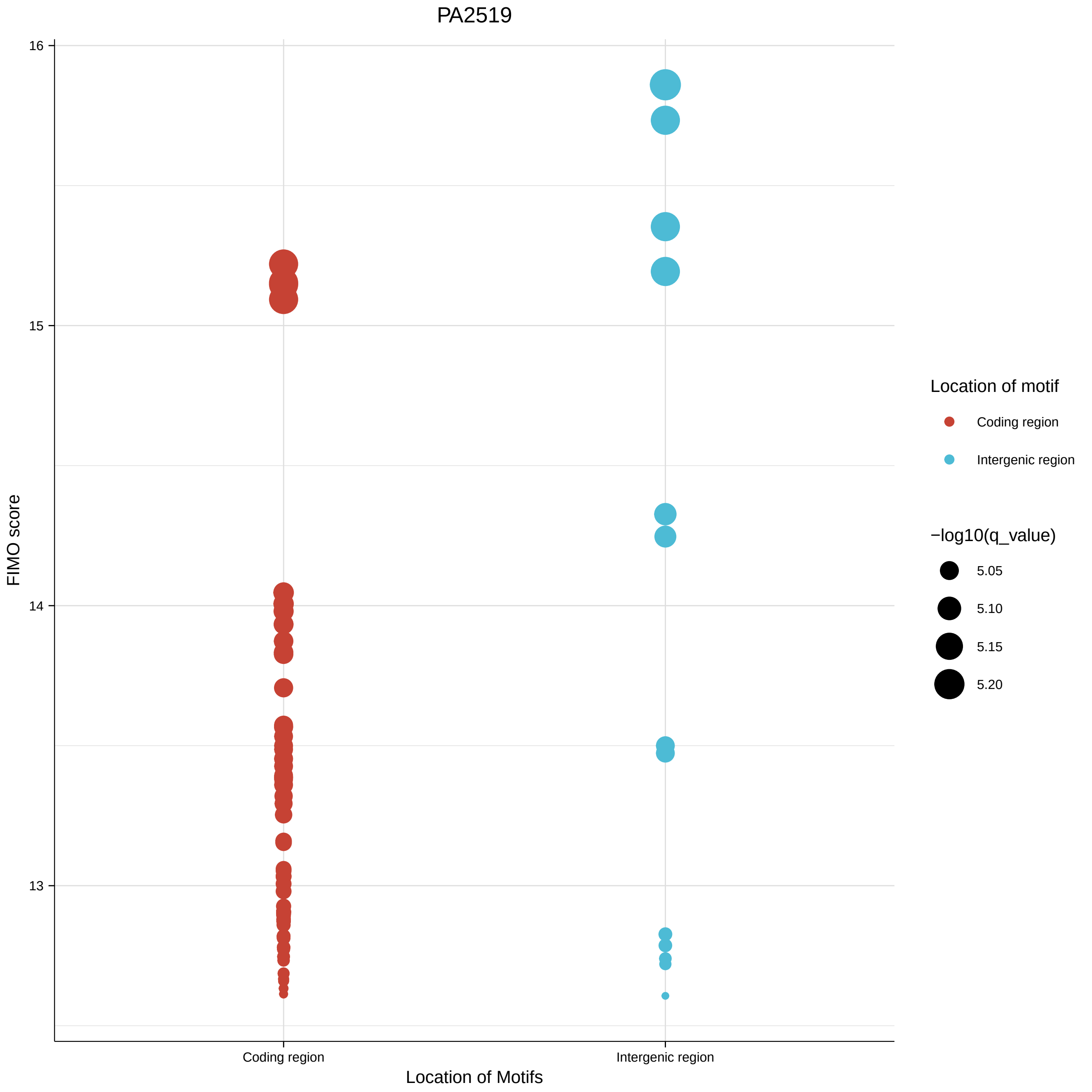
PA2519
16
15
14
13
FIMO score
Coding region
Intergenic region
Location of Motifs
Location of motif
Coding region
Intergenic region
−log10(q_value)
5.05
5.10
5.15
5.20

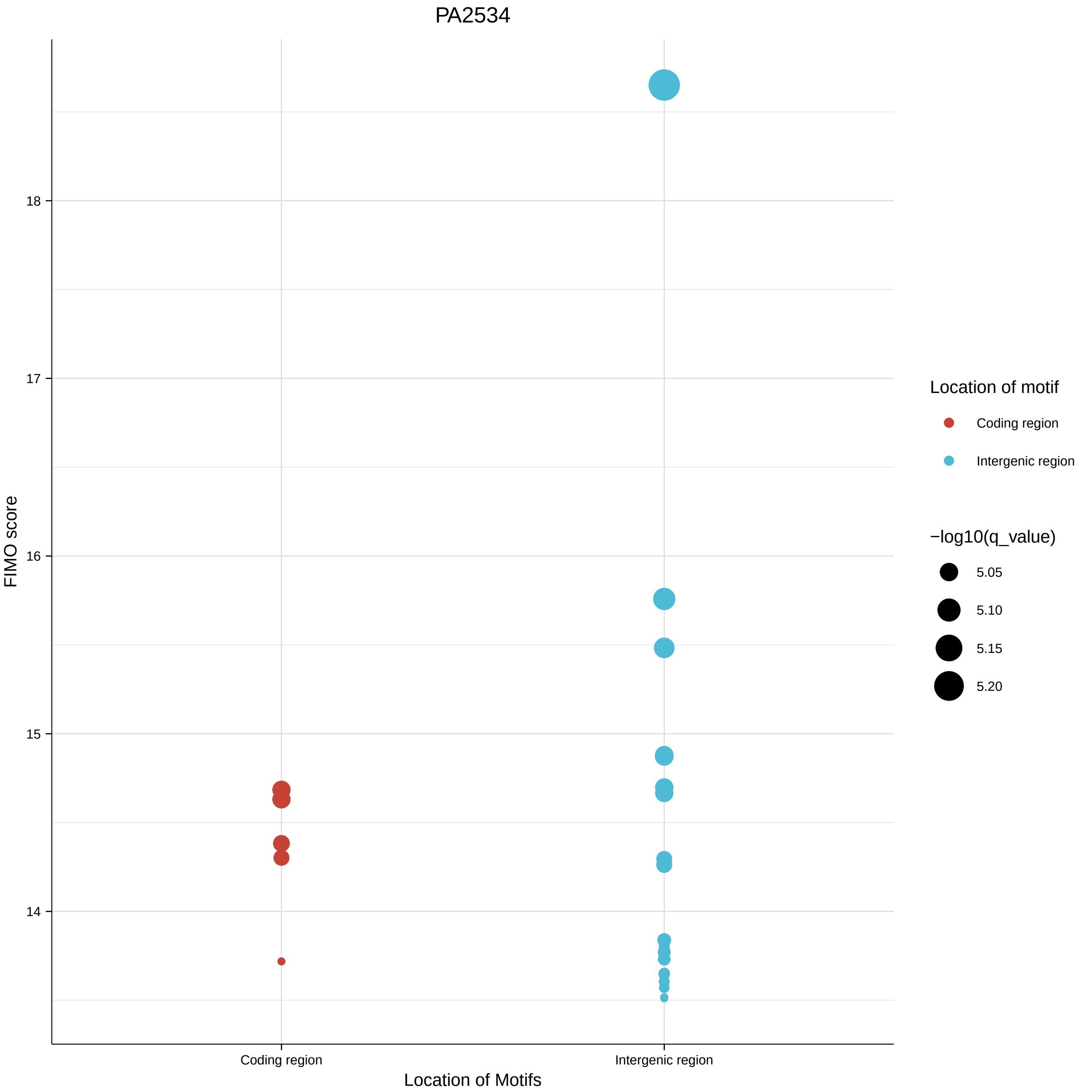

PA2534
FIMO score
18
17
16
15
14
Coding region
Intergenic region
Location of Motifs
Location of motif
Coding region
Intergenic region
−log10(q_value)
5.05
5.10
5.15
5.20

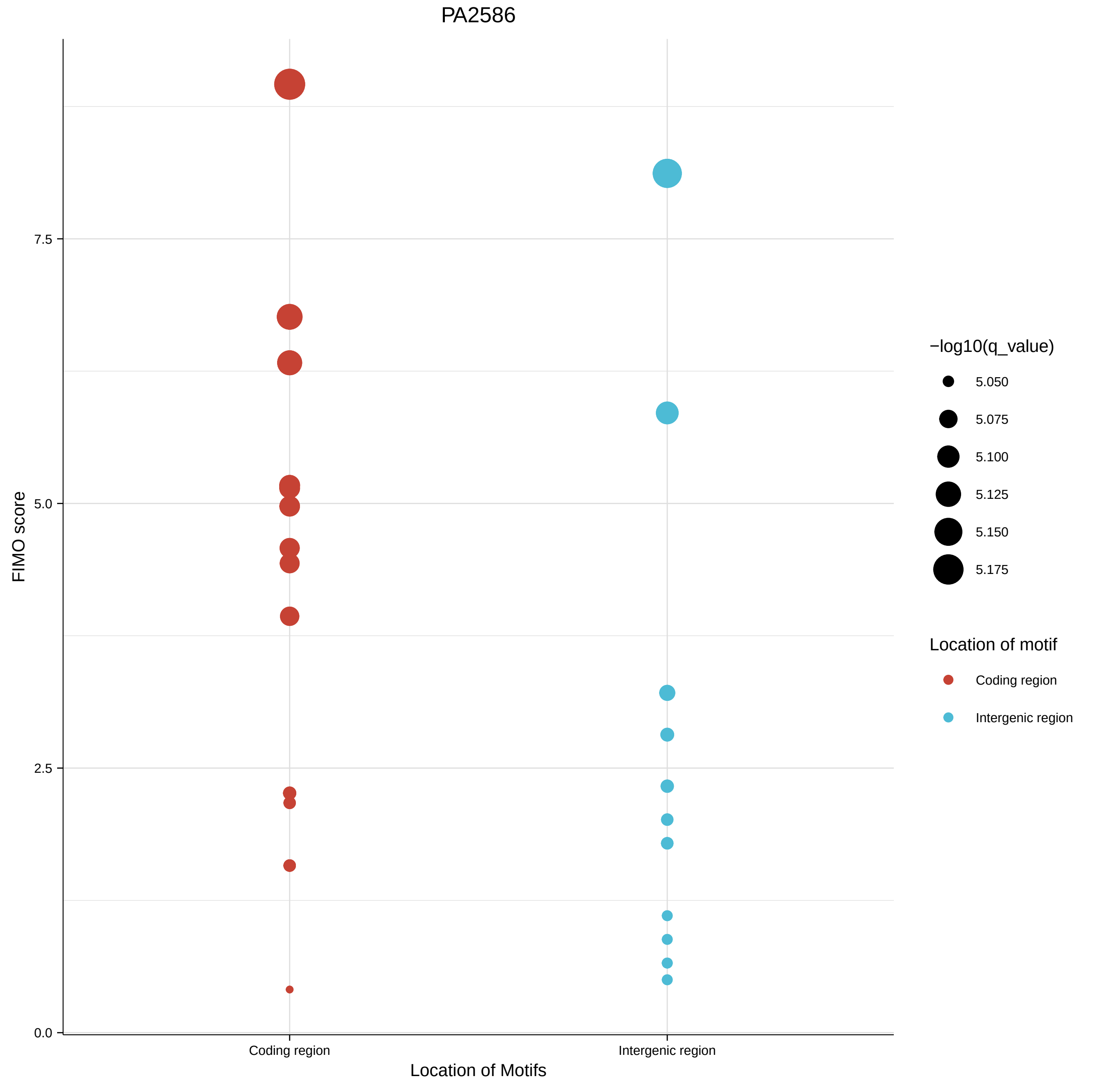
PA2586
FIMO score
7.5
5.0
2.5
0.0
Coding region
Intergenic region
Location of Motifs
−log10(q_value)
5.050
5.075
5.100
5.125
5.150
5.175
Location of motif
Coding region
Intergenic region

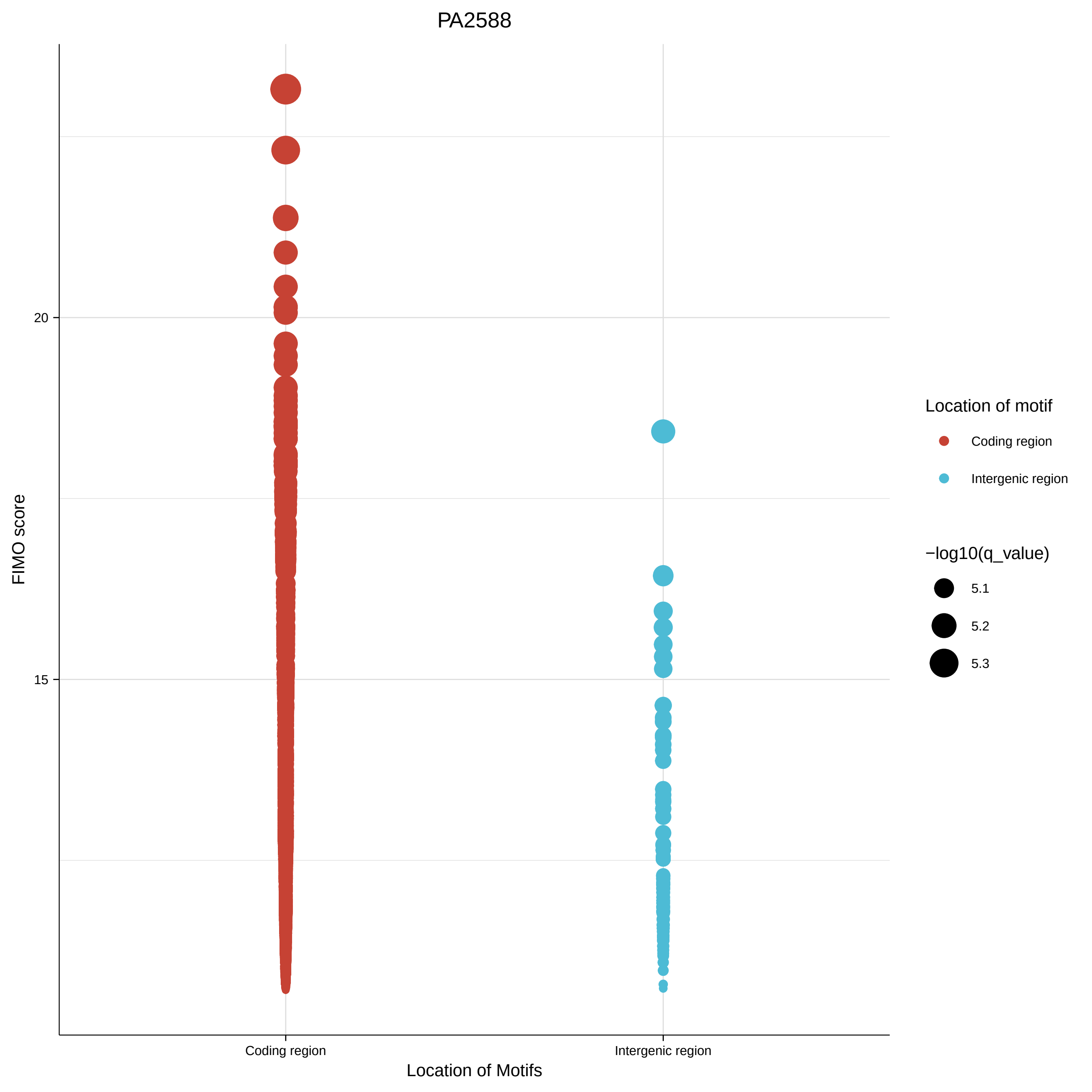
PA2588
FIMO score
20
15
Coding region
Intergenic region
Location of Motifs
Location of motif
Coding region
Intergenic region
−log10(q_value)
5.1
5.2
5.3

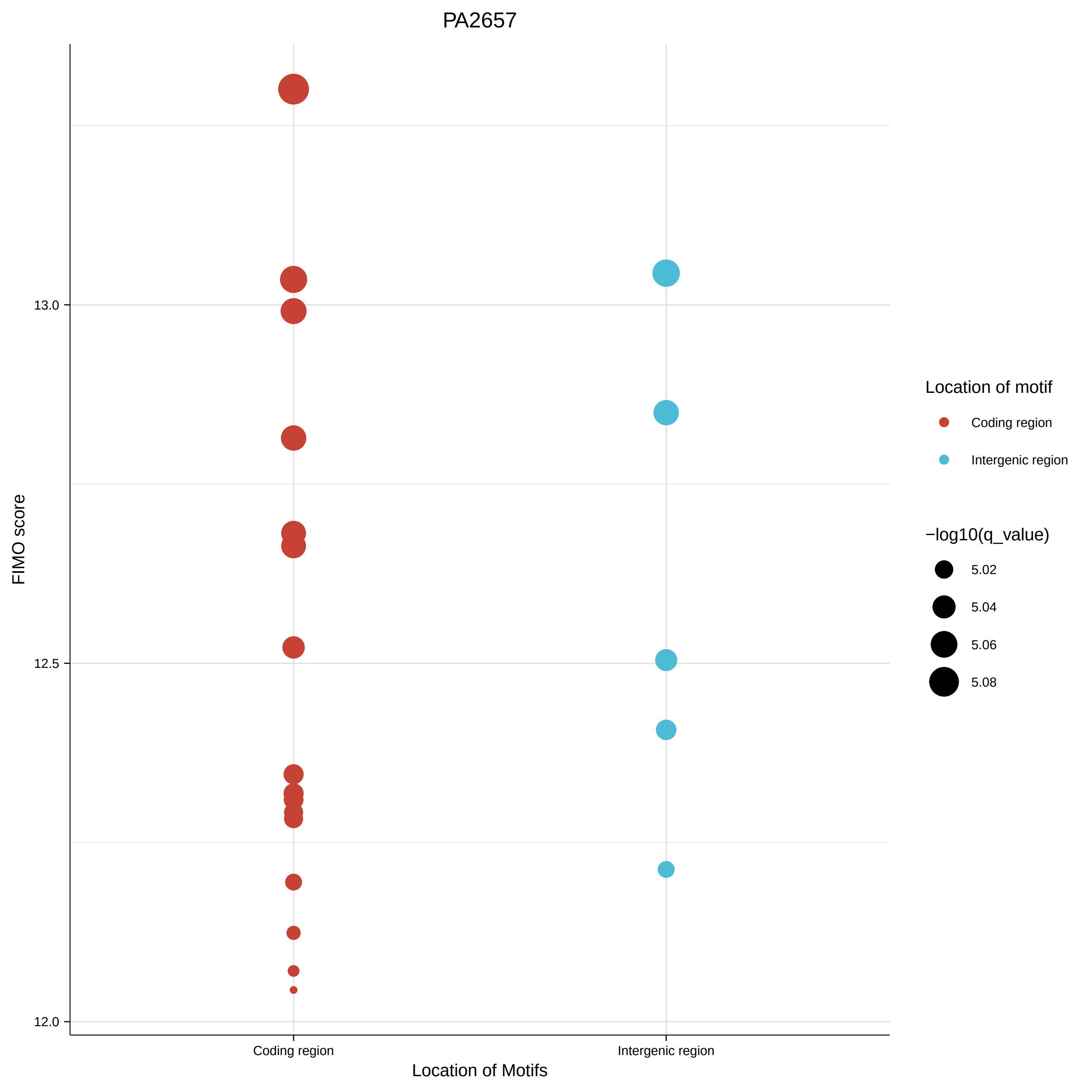

PA2657
FIMO score
13.0
12.5
12.0
Coding region
Intergenic region
Location of Motifs
Location of motif
Coding region
Intergenic region
−log10(q_value)
5.02
5.04
5.06
5.08

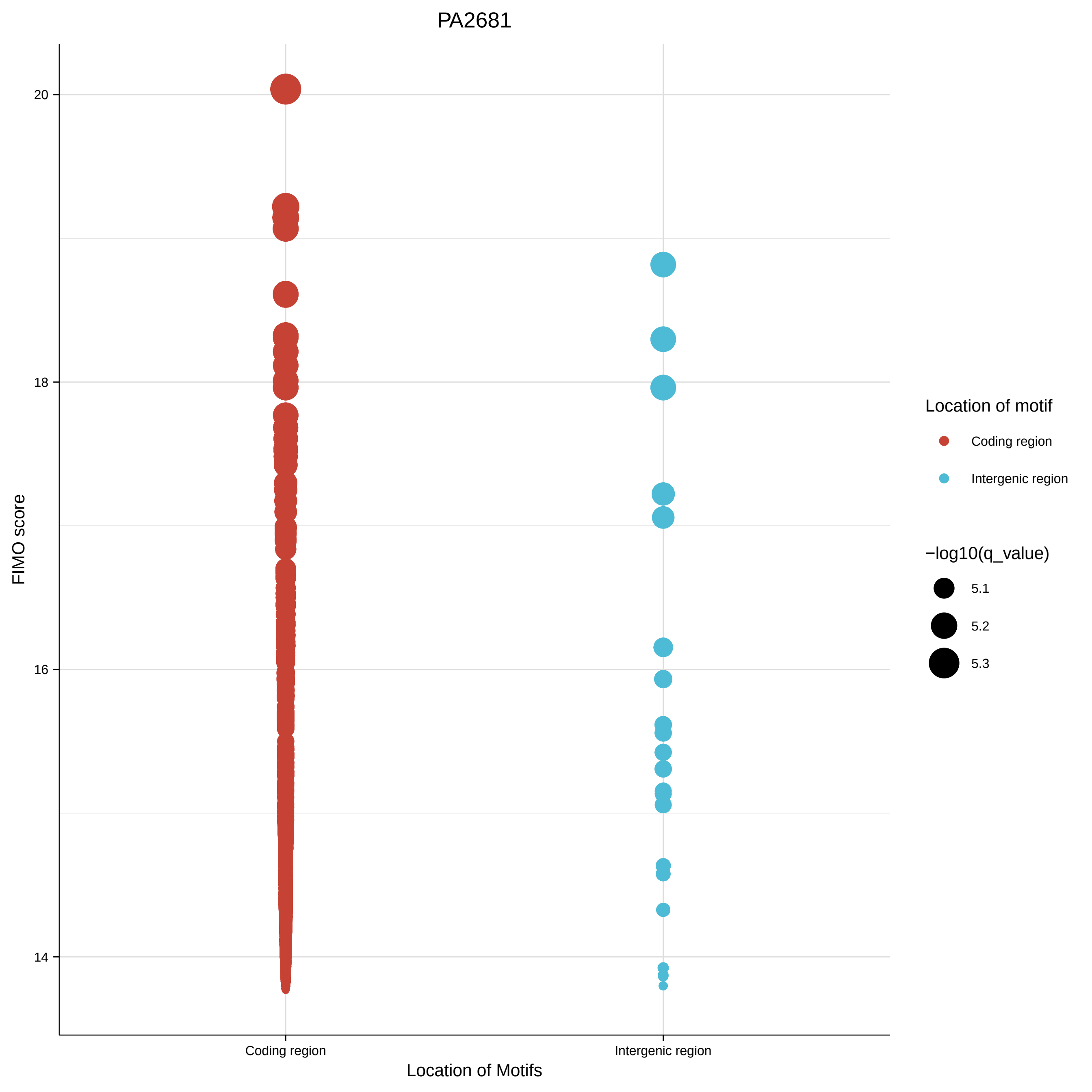
PA2681
FIMO score
20
18
16
14
Coding region
Intergenic region
Location of Motifs
Location of motif
Coding region
Intergenic region
−log10(q_value)
5.1
5.2
5.3

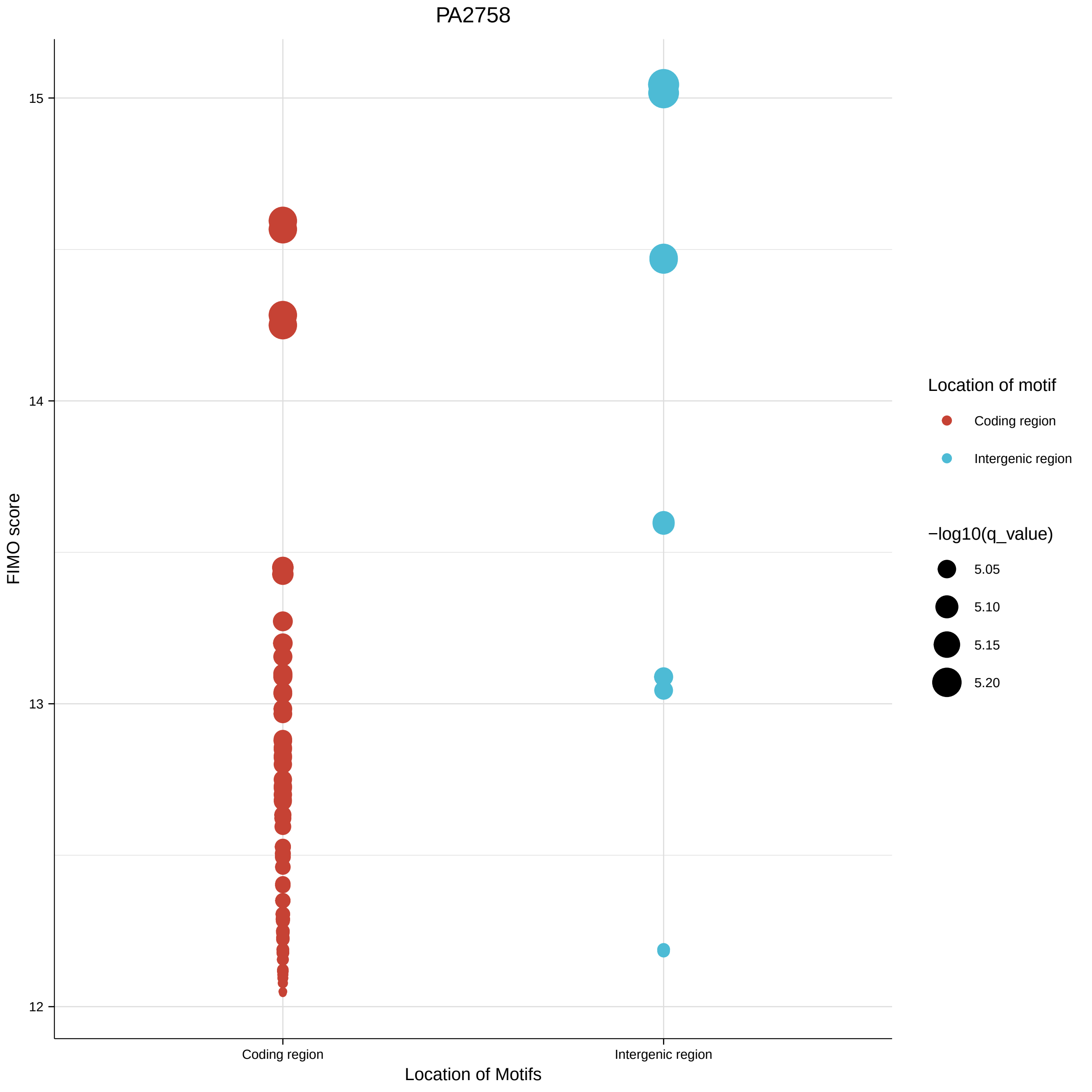

PA2758
FIMO score
15
14
13
12
Coding region
Intergenic region
Location of Motifs
Location of motif
Coding region
Intergenic region
−log10(q_value)
5.05
5.10
5.15
5.20

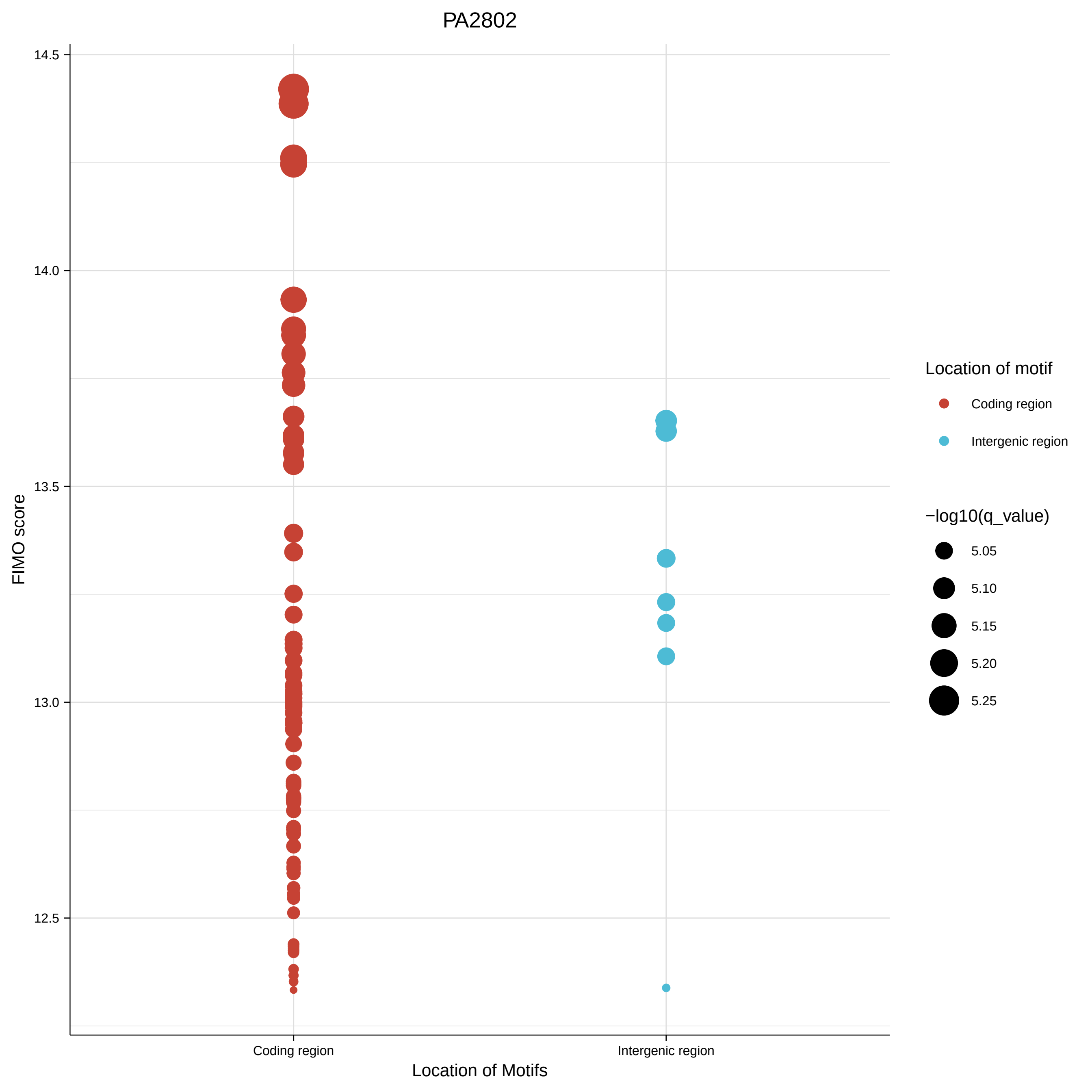
PA2802
FIMO score
14.5
14.0
13.5
13.0
12.5
Coding region
Intergenic region
Location of Motifs
Location of motif
Coding region
Intergenic region
−log10(q_value)
5.05
5.10
5.15
5.20
5.25

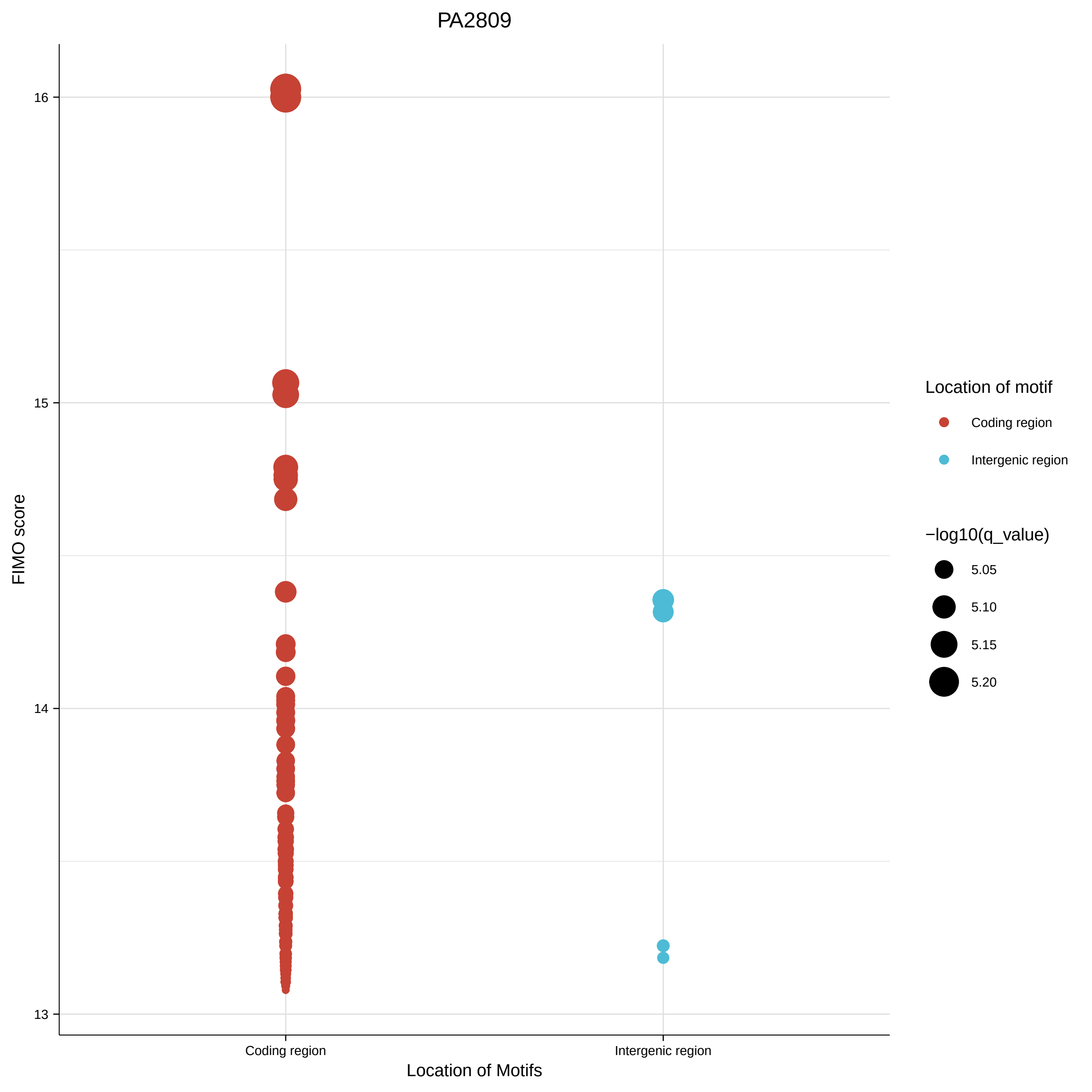

PA2809
FIMO score
16
15
14
13
Coding region
Intergenic region
Location of Motifs
Location of motif
Coding region
Intergenic region
−log10(q_value)
5.05
5.10
5.15
5.20

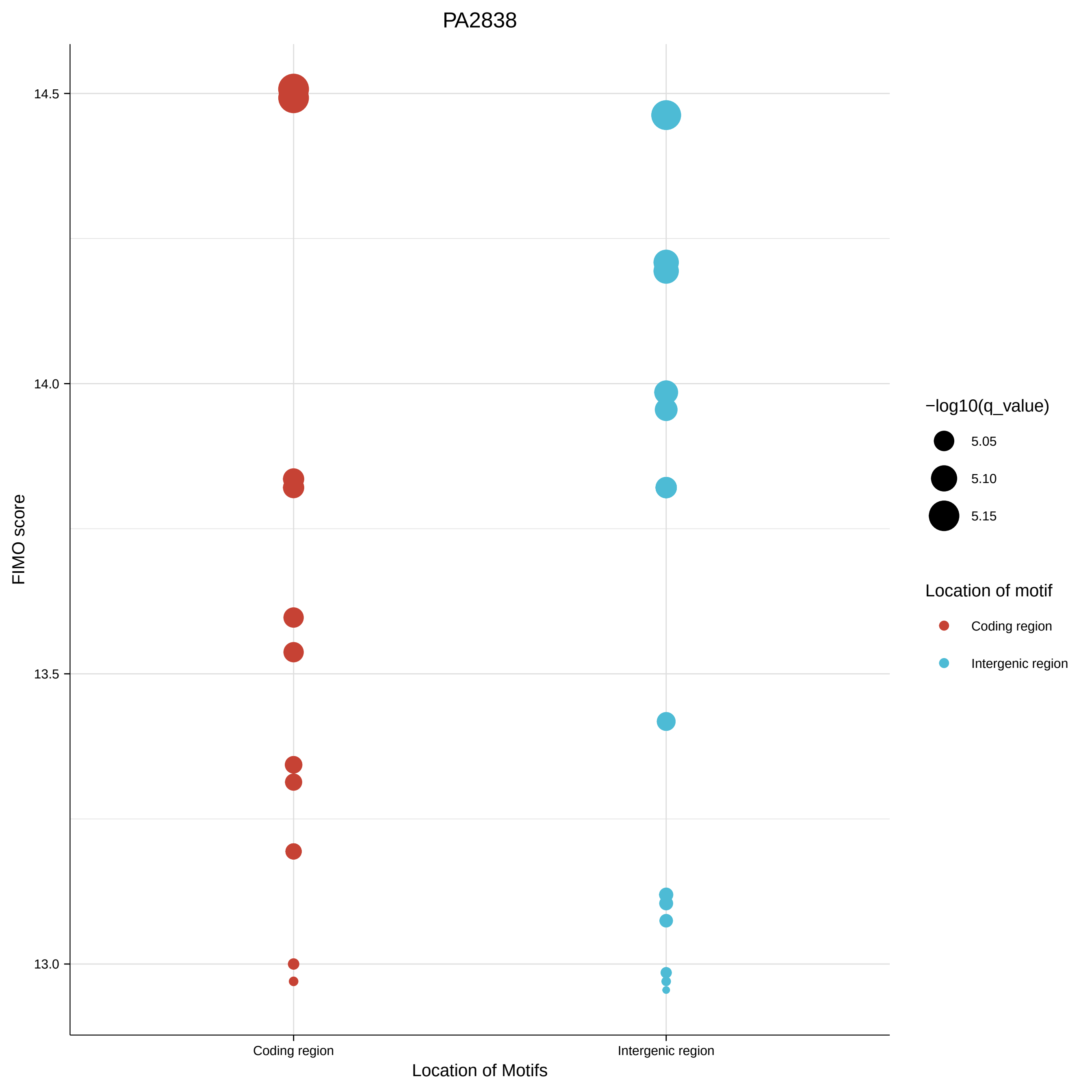
PA2838
FIMO score
14.5
14.0
13.5
13.0
Coding region
Intergenic region
Location of Motifs
−log10(q_value)
5.05
5.10
5.15
Location of motif
Coding region
Intergenic region

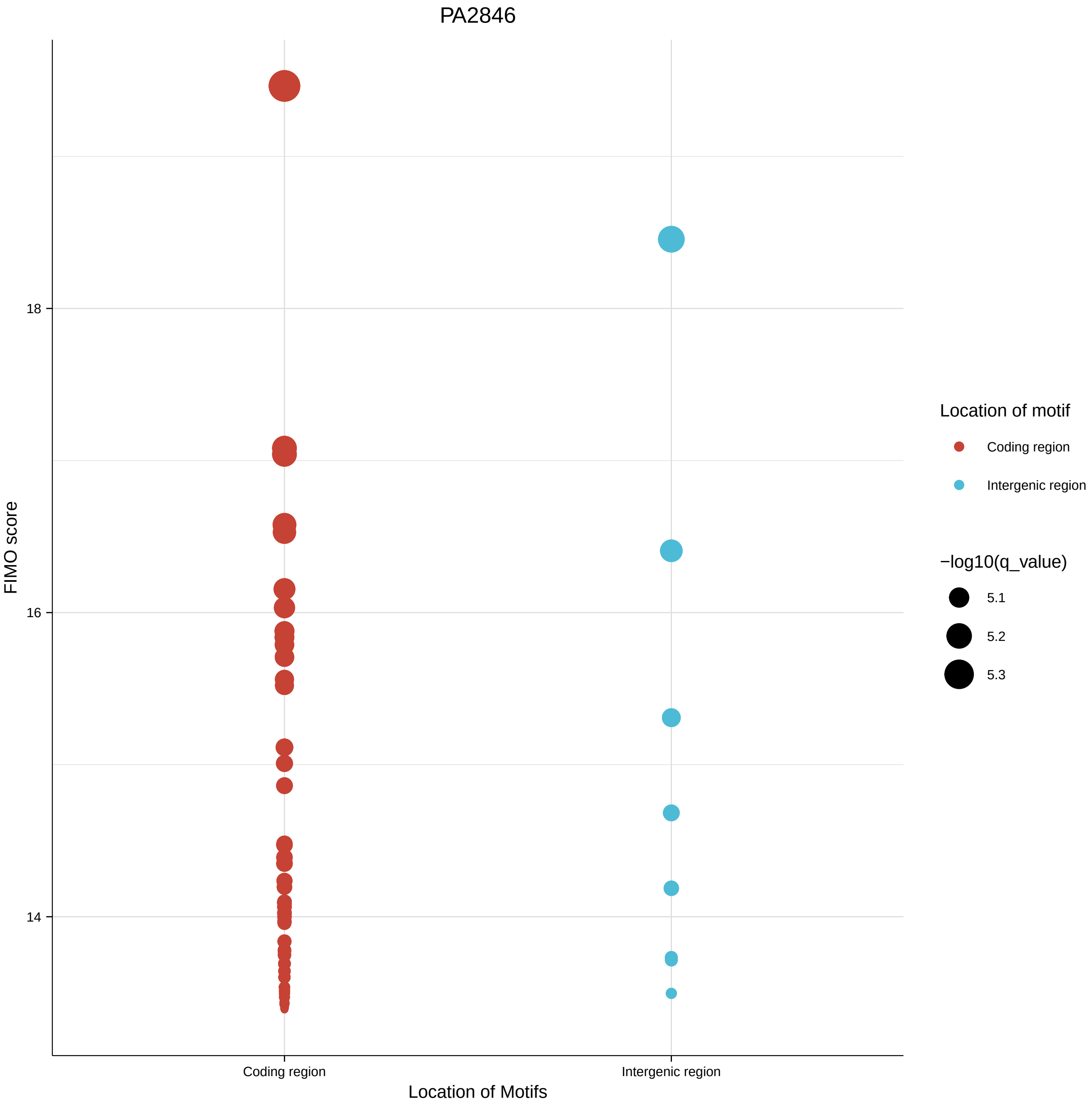
PA2846
FIMO score
18
16
14
Coding region
Intergenic region
Location of Motifs
Location of motif
Coding region
Intergenic region
−log10(q_value)
5.1
5.2
5.3

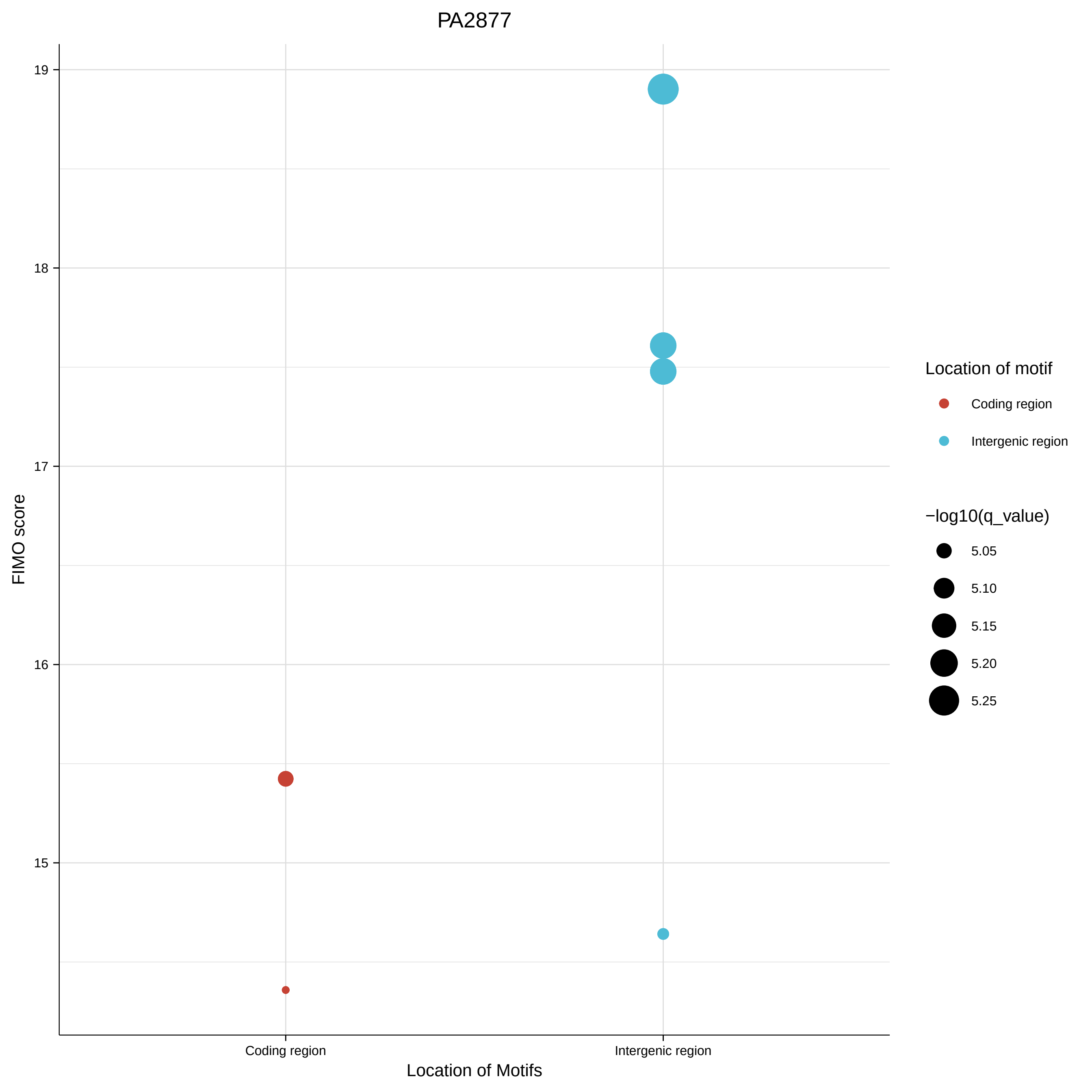
PA2877
FIMO score
19
18
17
16
15
Coding region
Intergenic region
Location of Motifs
Location of motif
Coding region
Intergenic region
−log10(q_value)
5.05
5.10
5.15
5.20
5.25

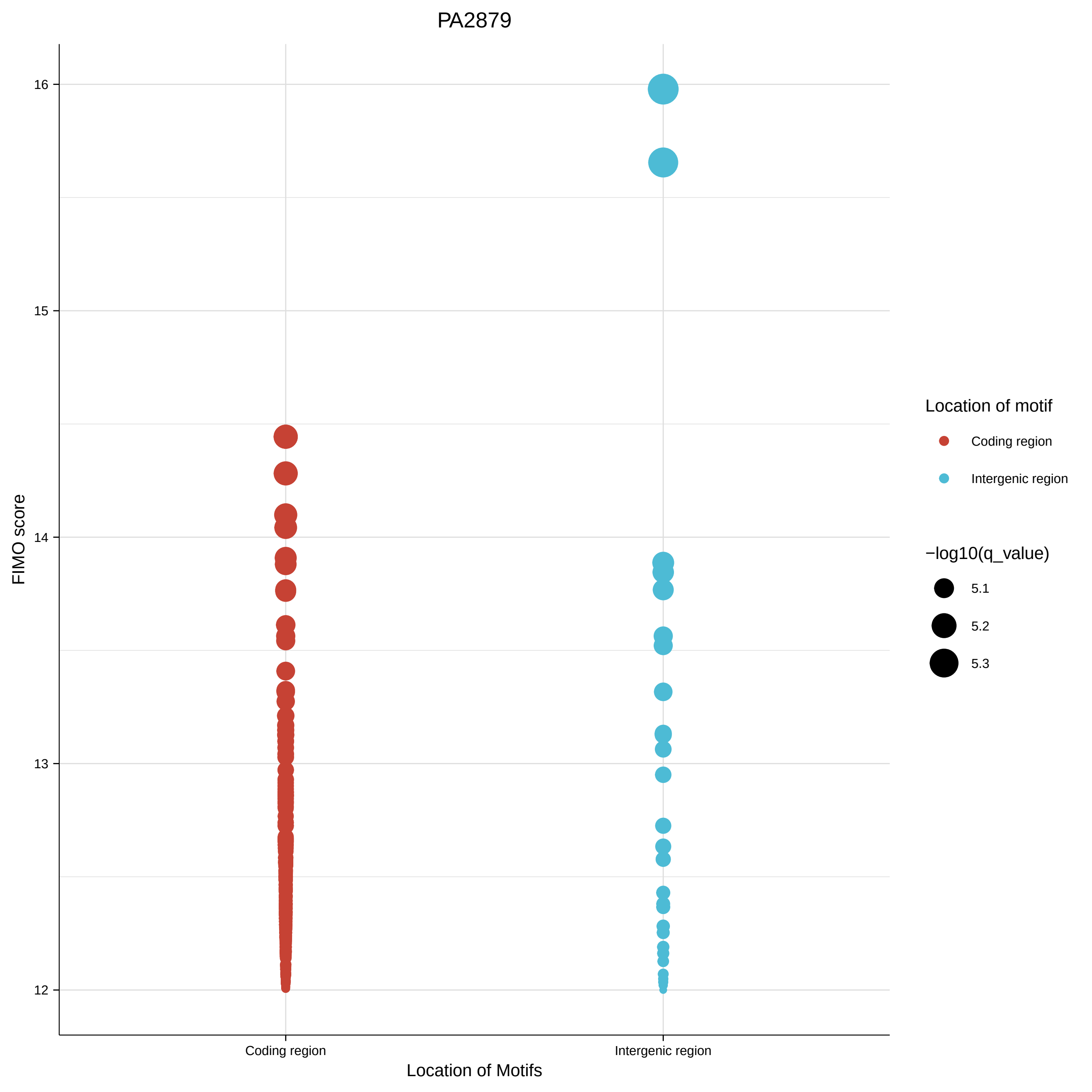

PA2879
FIMO score
16
15
14
13
12
Coding region
Intergenic region
Location of Motifs
Location of motif
Coding region
Intergenic region
−log10(q_value)
5.1
5.2
5.3

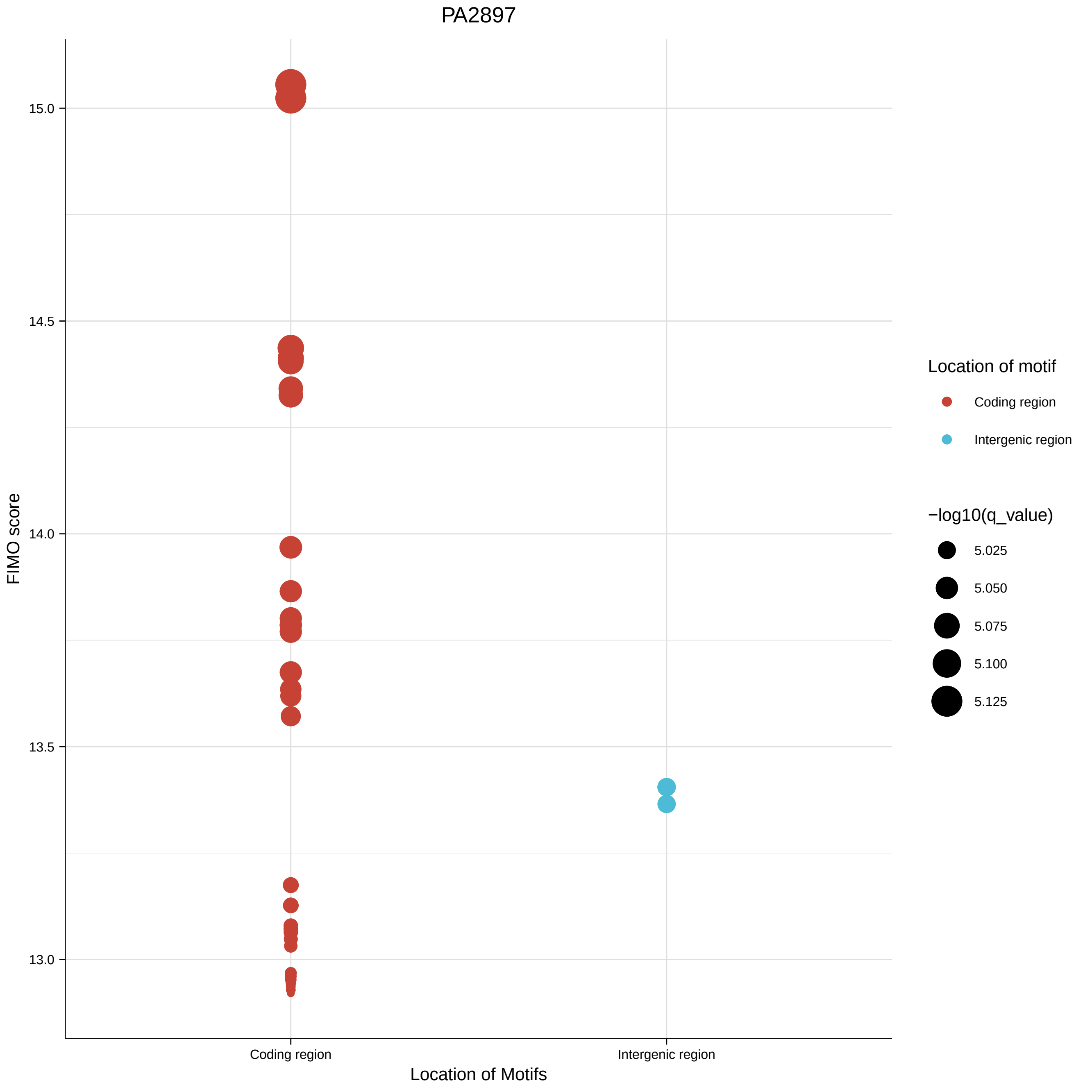

PA2897
FIMO score
15.0
14.5
14.0
13.5
13.0
Coding region
Intergenic region
Location of Motifs
Location of motif
Coding region
Intergenic region
−log10(q_value)
5.025
5.050
5.075
5.100
5.125

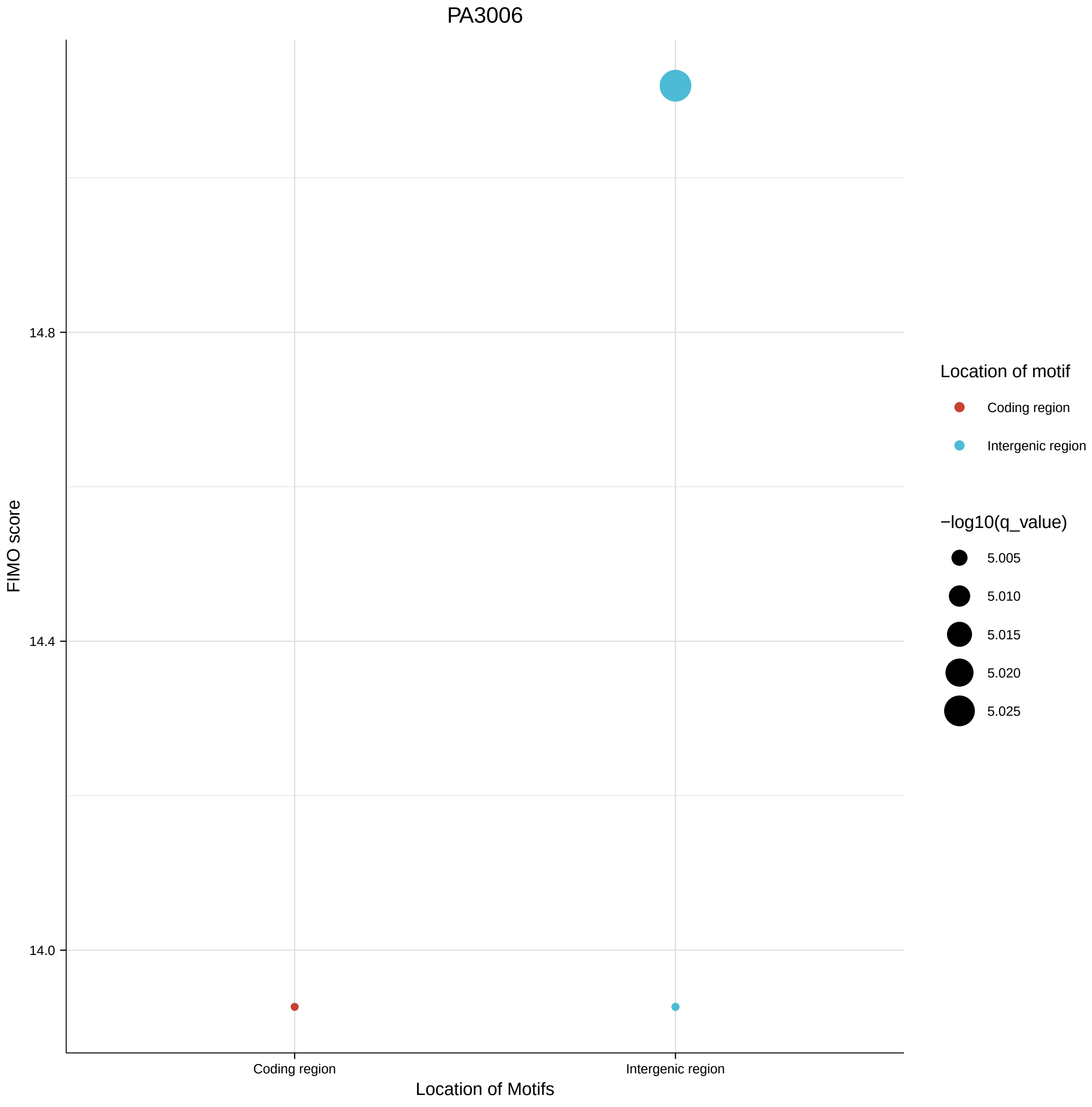
PA3006
FIMO score
14.8
14.4
14.0
Coding region
Intergenic region
Location of Motifs
Location of motif
Coding region
Intergenic region
−log10(q_value)
5.005
5.010
5.015
5.020
5.025

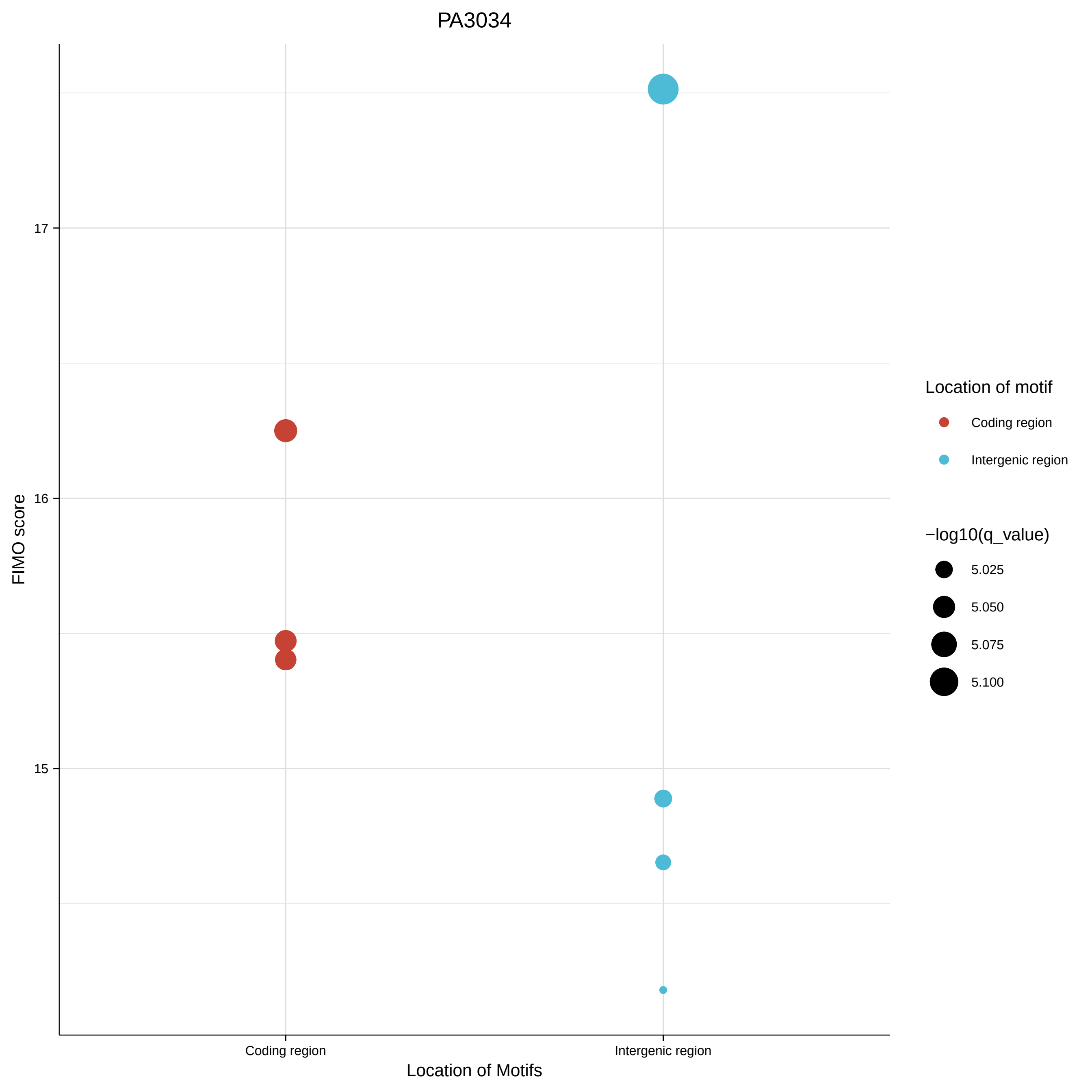

PA3034
17
16
15
FIMO score
Coding region
Intergenic region
Location of Motifs
Location of motif
Coding region
Intergenic region
−log10(q_value)
5.025
5.050
5.075
5.100

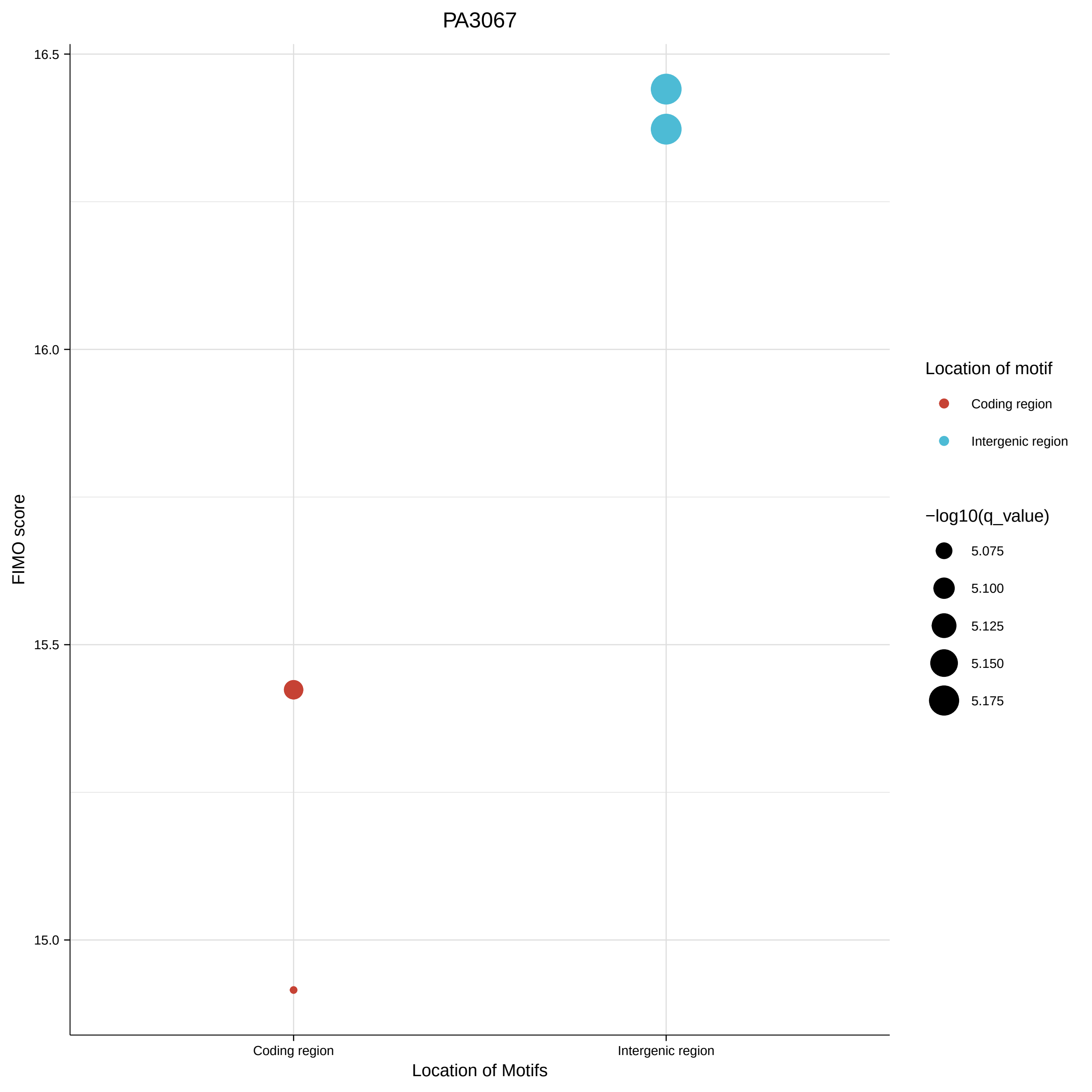

PA3067
FIMO score
16.5
16.0
15.5
15.0
Coding region
Intergenic region
Location of Motifs
Location of motif
Coding region
Intergenic region
−log10(q_value)
5.075
5.100
5.125
5.150
5.175

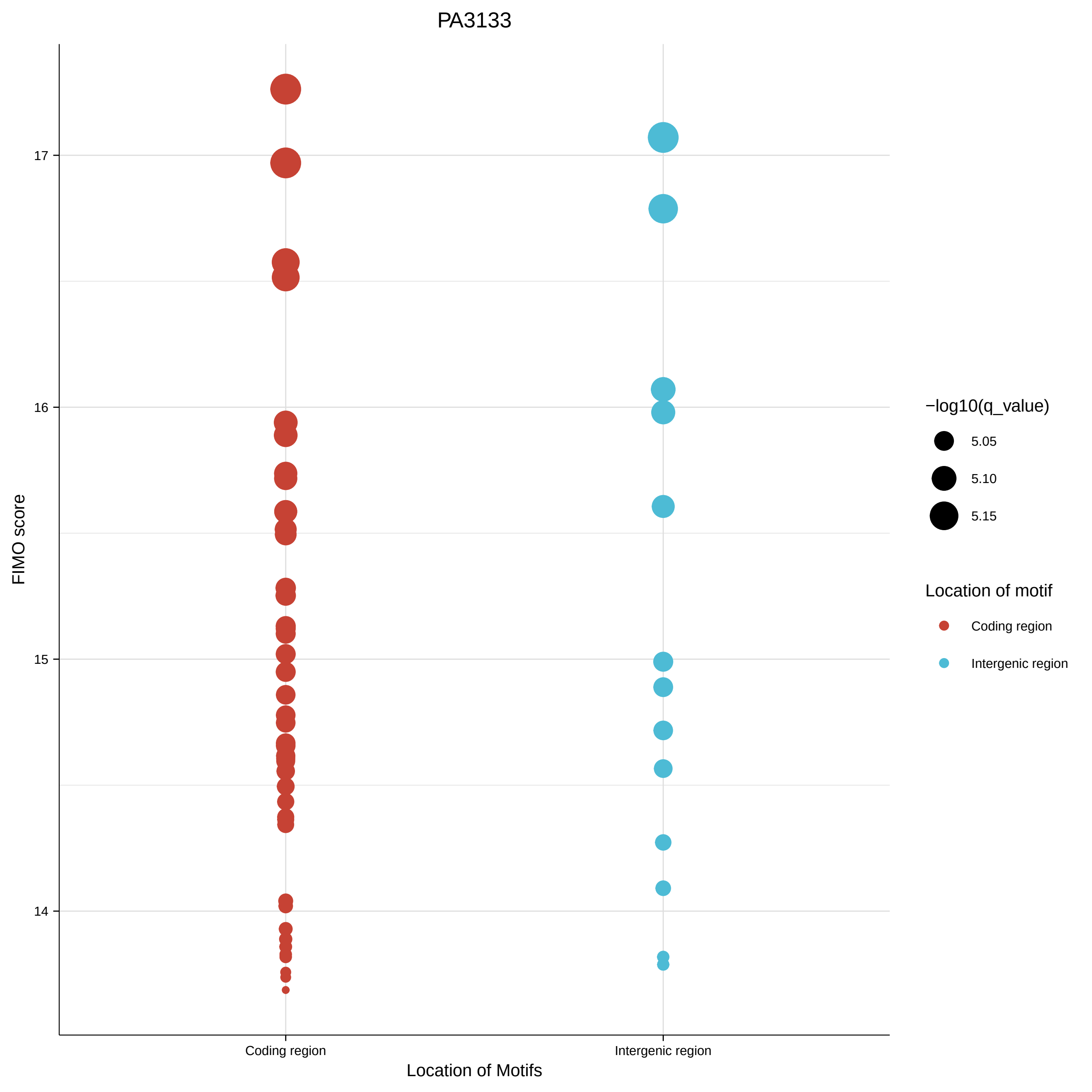

PA3133
FIMO score
17
16
15
14
Coding region
Intergenic region
Location of Motifs
−log10(q_value)
5.05
5.10
5.15
Location of motif
Coding region
Intergenic region

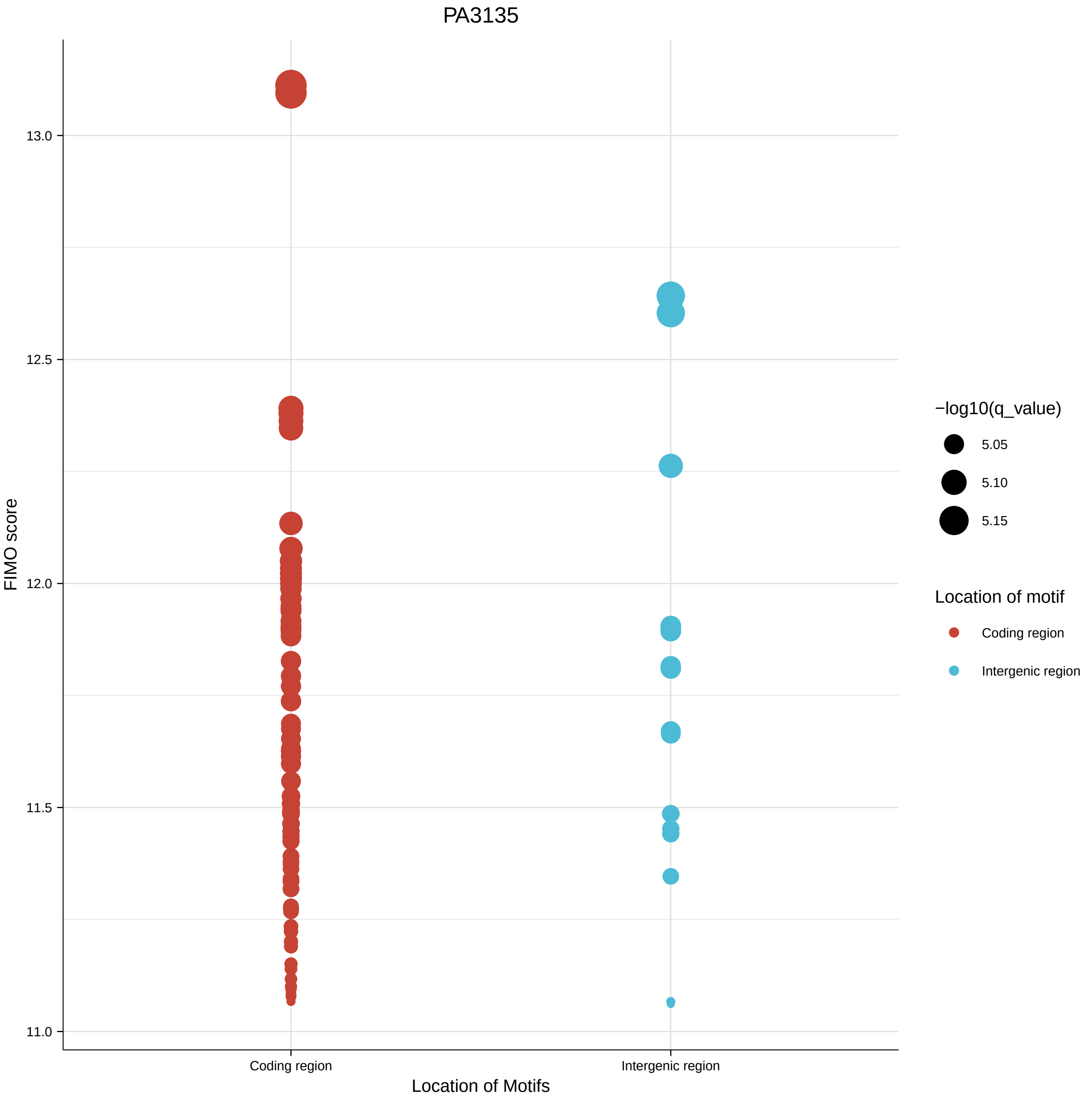
PA3135
FIMO score
13.0
12.5
12.0
11.5
11.0
Coding region
Intergenic region
Location of Motifs
−log10(q_value)
5.05
5.10
5.15
Location of motif
Coding region
Intergenic region

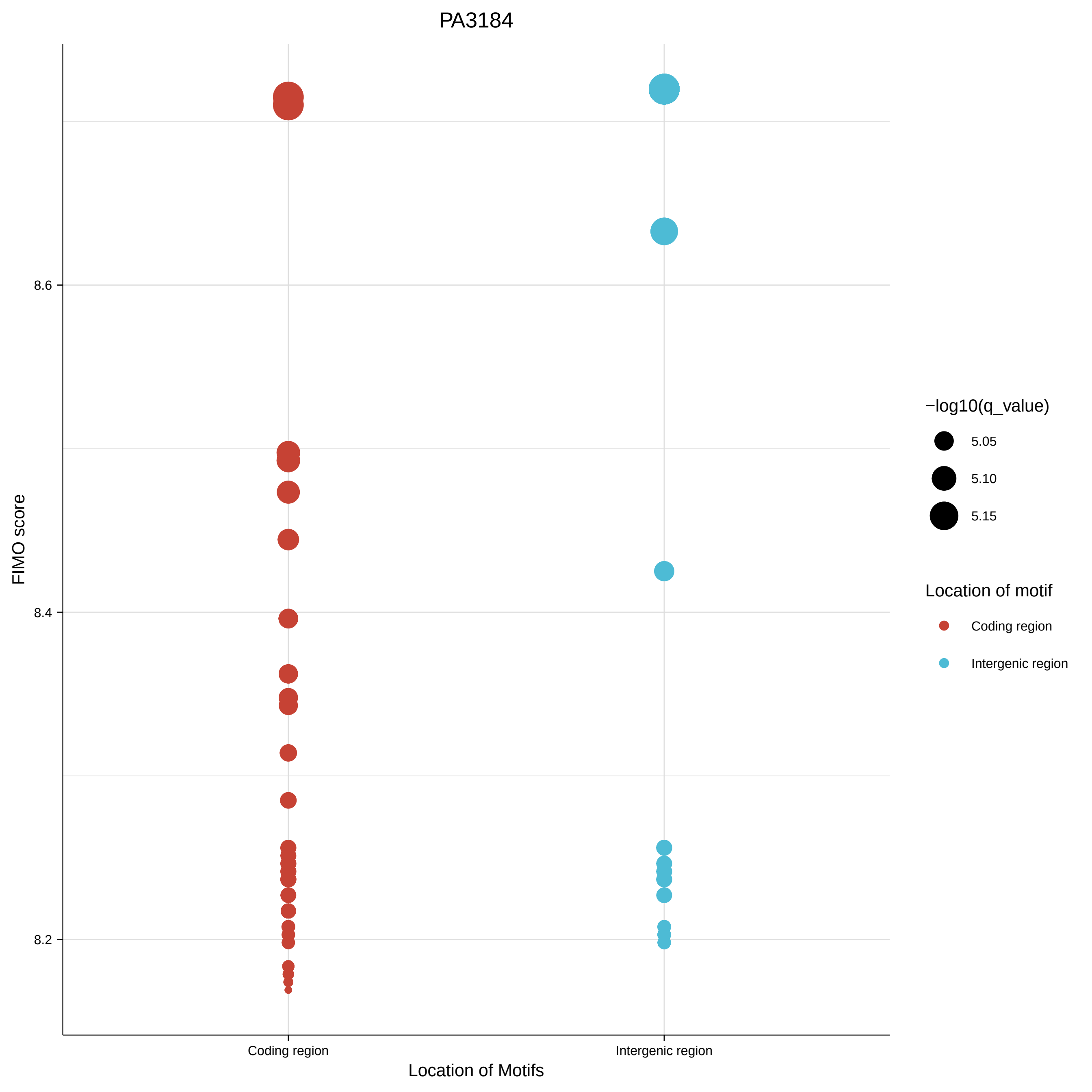

PA3184
FIMO score
8.6
8.4
8.2
Coding region
Intergenic region
Location of Motifs
−log10(q_value)
5.05
5.10
5.15
Location of motif
Coding region
Intergenic region

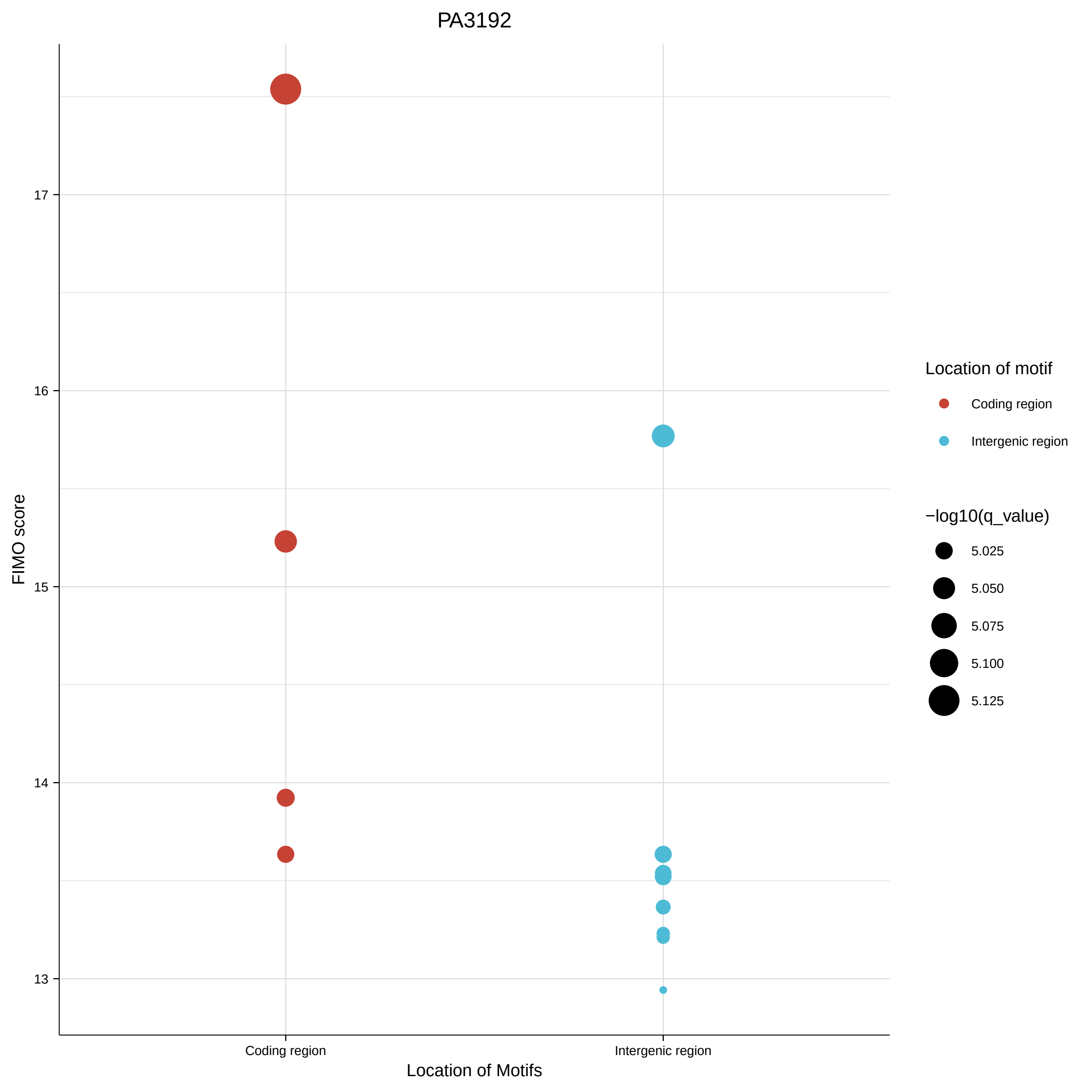

PA3192
FIMO score
17
16
15
14
13
Coding region
Intergenic region
Location of Motifs
Location of motif
Coding region
Intergenic region
−log10(q_value)
5.025
5.050
5.075
5.100
5.125

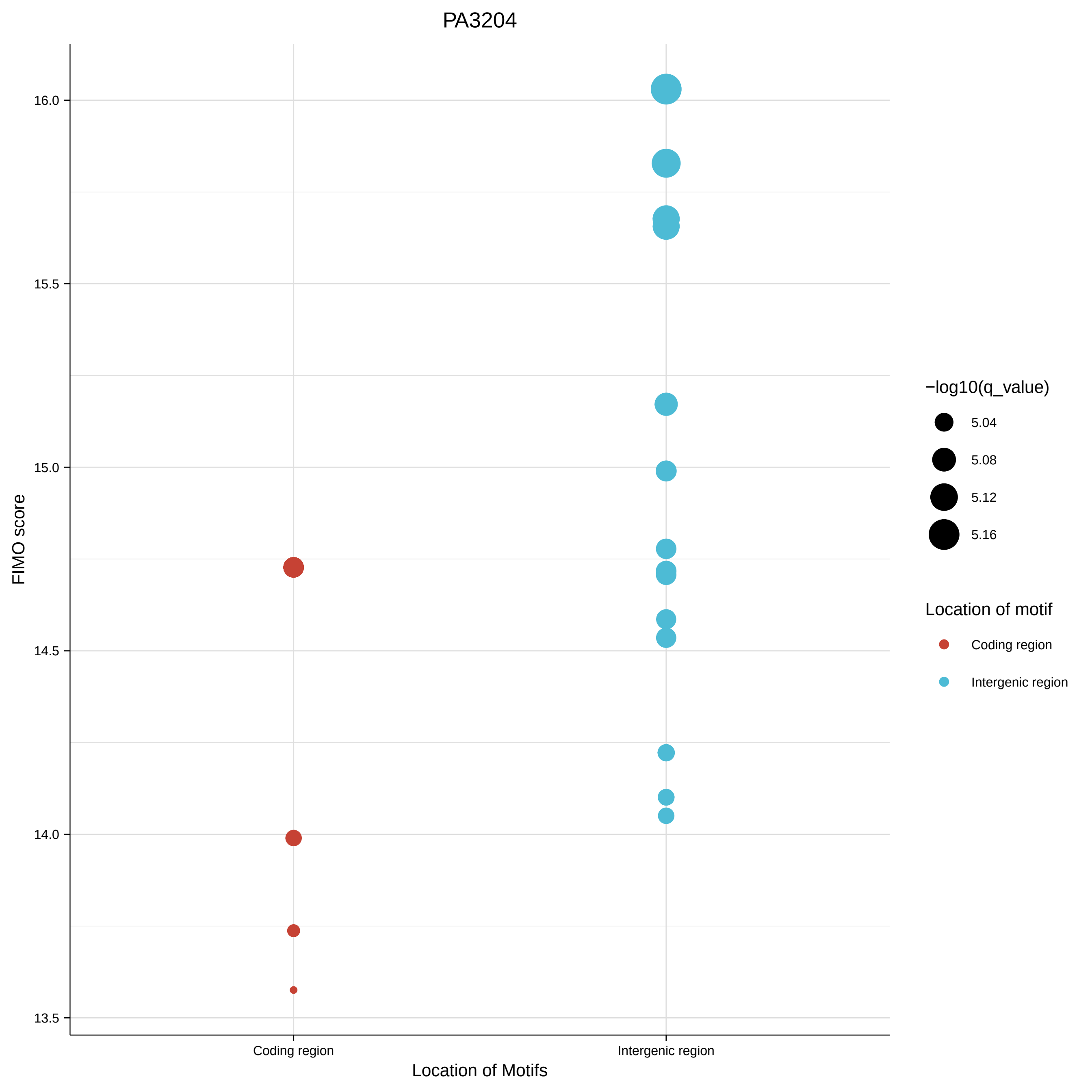
PA3204
FIMO score
16.0
15.5
15.0
14.5
14.0
13.5
Coding region
Intergenic region
Location of Motifs
−log10(q_value)
5.04
5.08
5.12
5.16
Location of motif
Coding region
Intergenic region

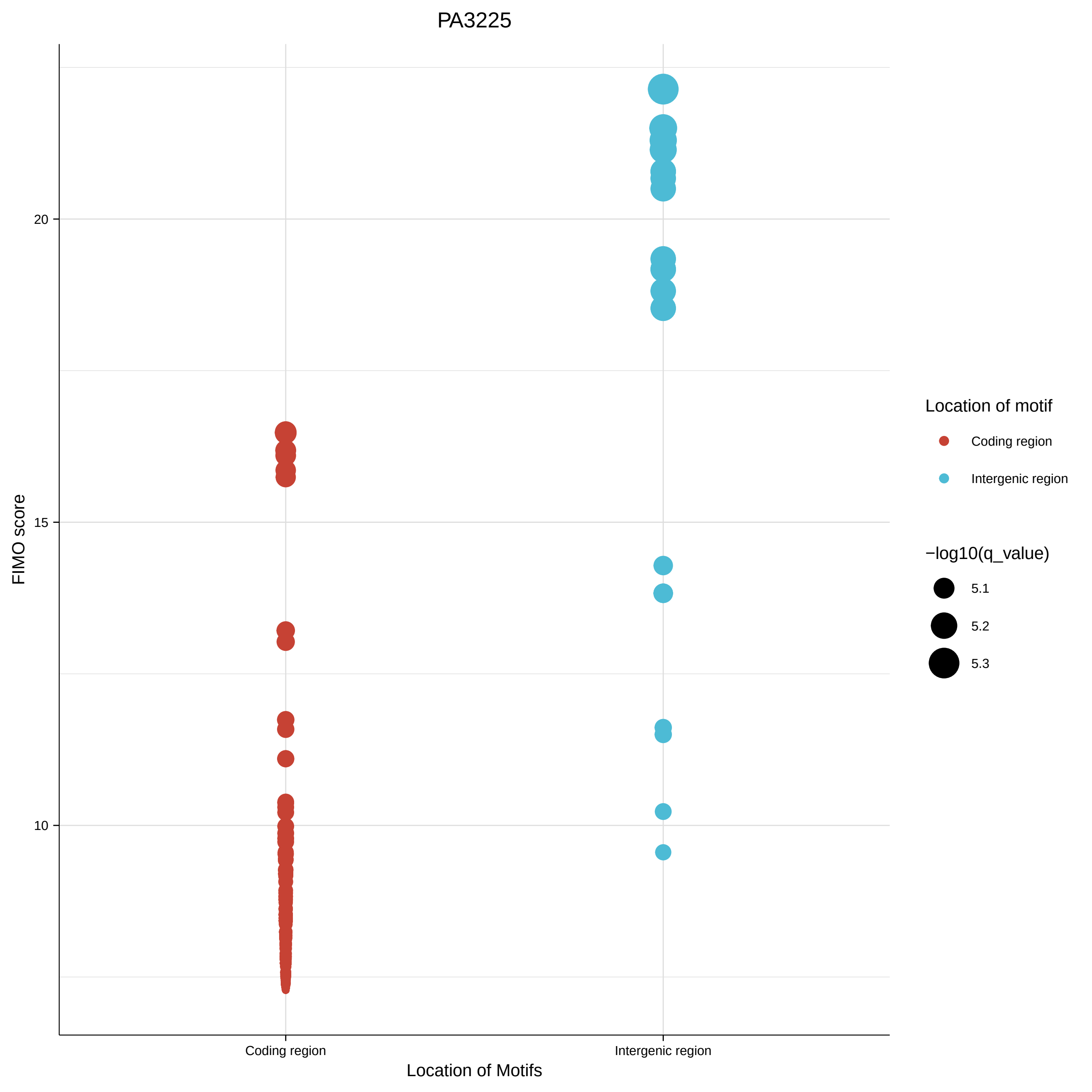

PA3225
FIMO score
20
15
10
Coding region
Intergenic region
Location of Motifs
Location of motif
Coding region
Intergenic region
−log10(q_value)
5.1
5.2
5.3

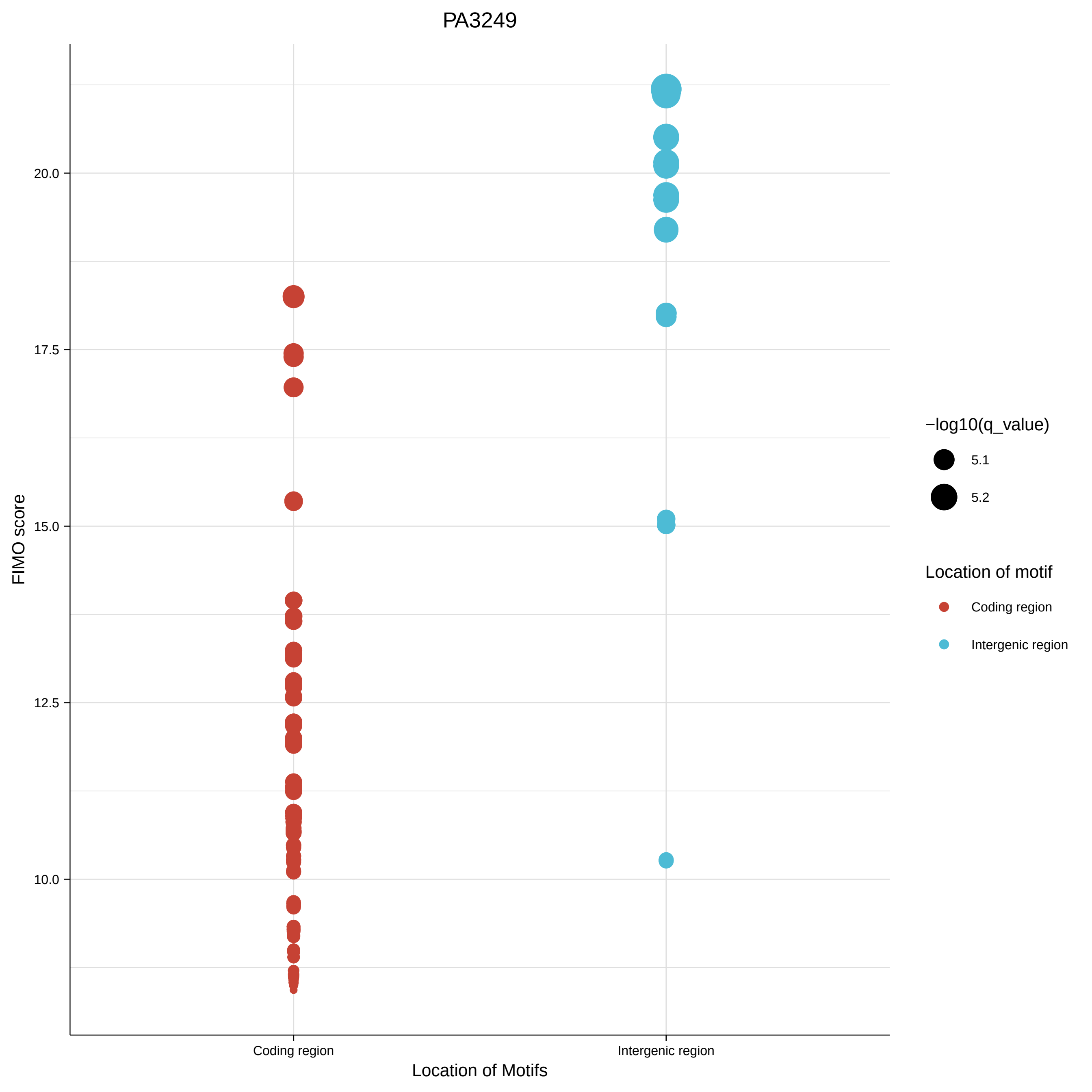

PA3249
FIMO score
20.0
17.5
15.0
12.5
10.0
Coding region
Intergenic region
Location of Motifs
−log10(q_value)
5.1
5.2
Location of motif
Coding region
Intergenic region

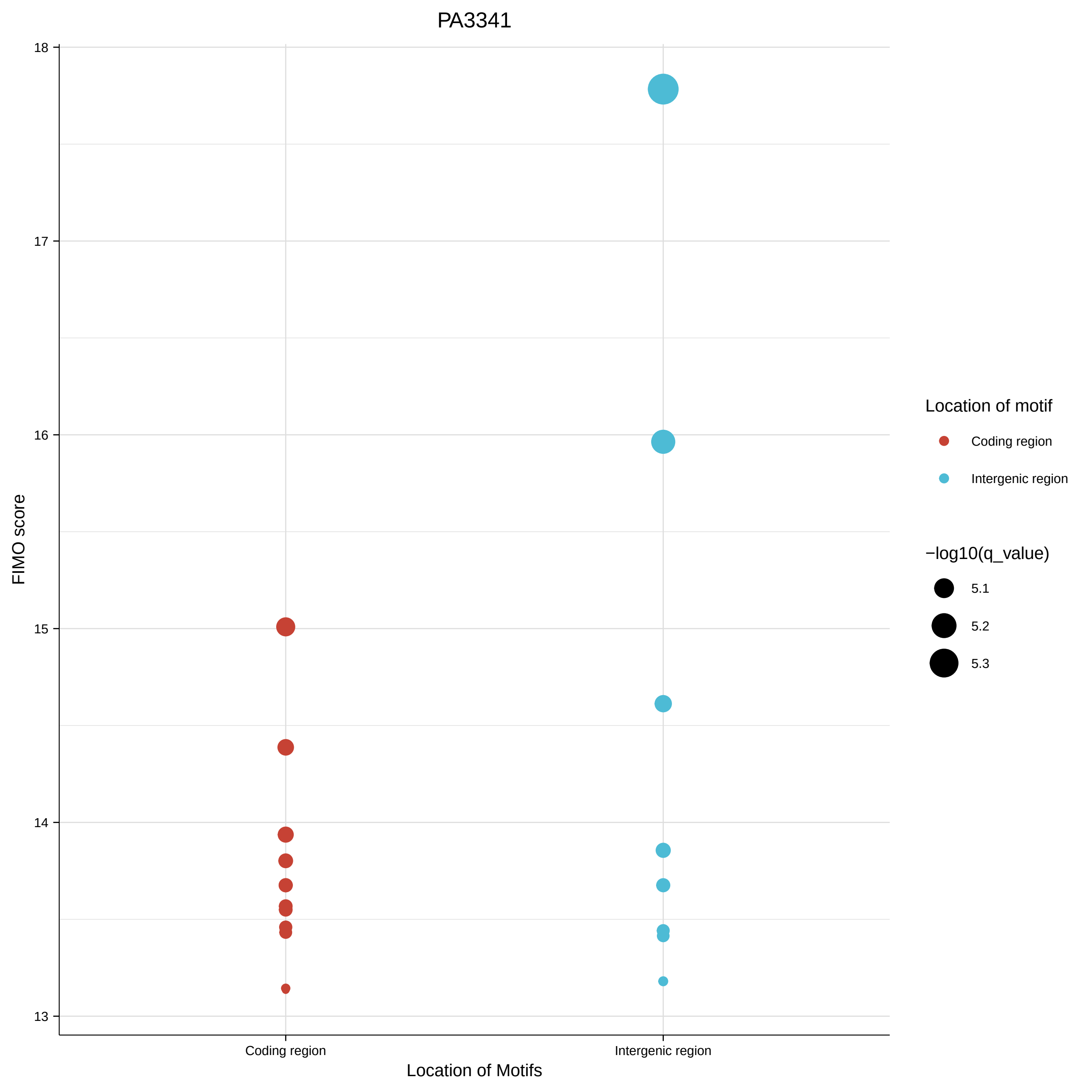

PA3341
FIMO score
18
17
16
15
14
13
Coding region
Intergenic region
Location of Motifs
Location of motif
Coding region
Intergenic region
−log10(q_value)
5.1
5.2
5.3

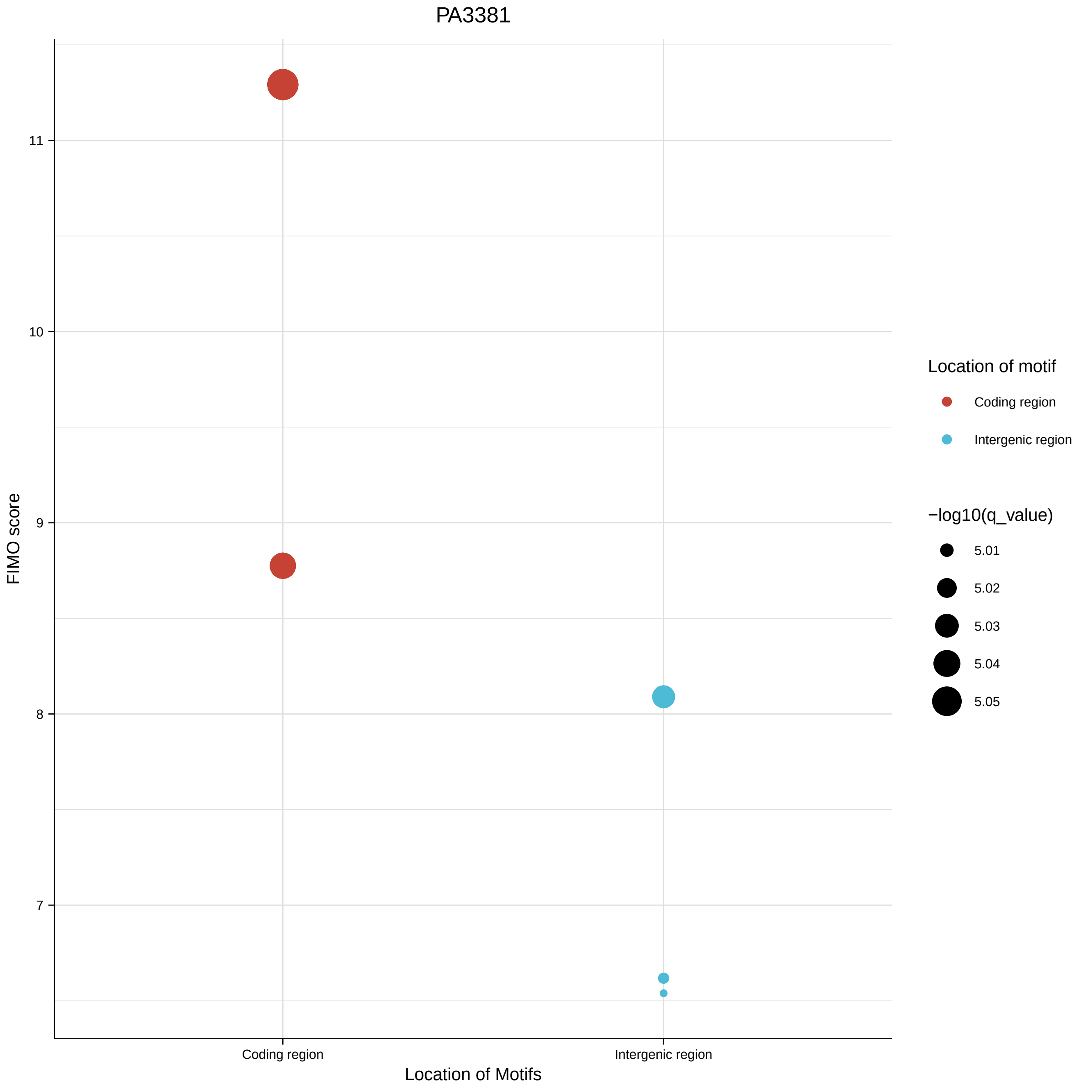
PA3381
FIMO score
11
10
9
8
7
Coding region
Intergenic region
Location of Motifs
Location of motif
Coding region
Intergenic region
−log10(q_value)
5.01
5.02
5.03
5.04
5.05

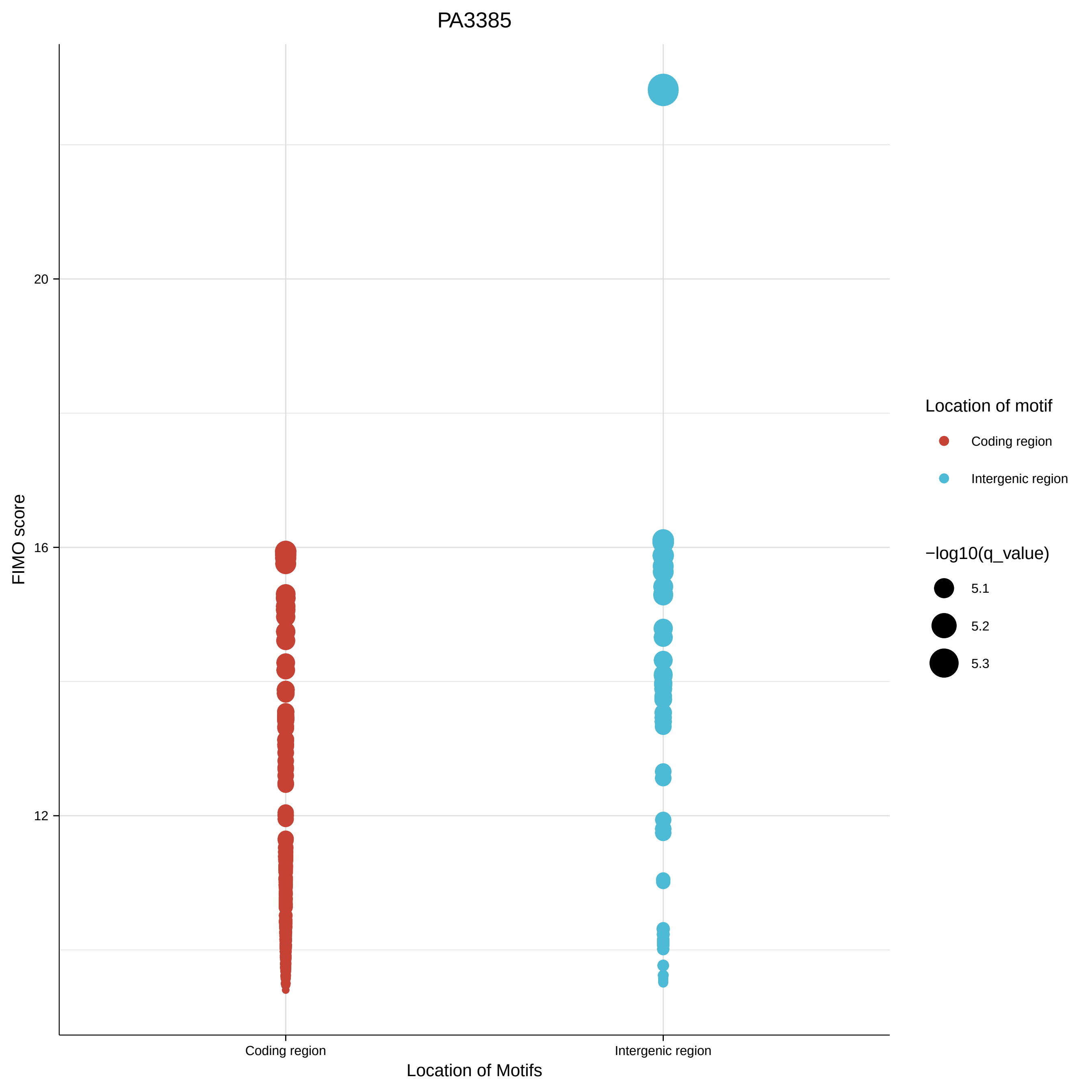

PA3385
FIMO score
20
16
12
Coding region
Intergenic region
Location of Motifs
Location of motif
Coding region
Intergenic region
−log10(q_value)
5.1
5.2
5.3

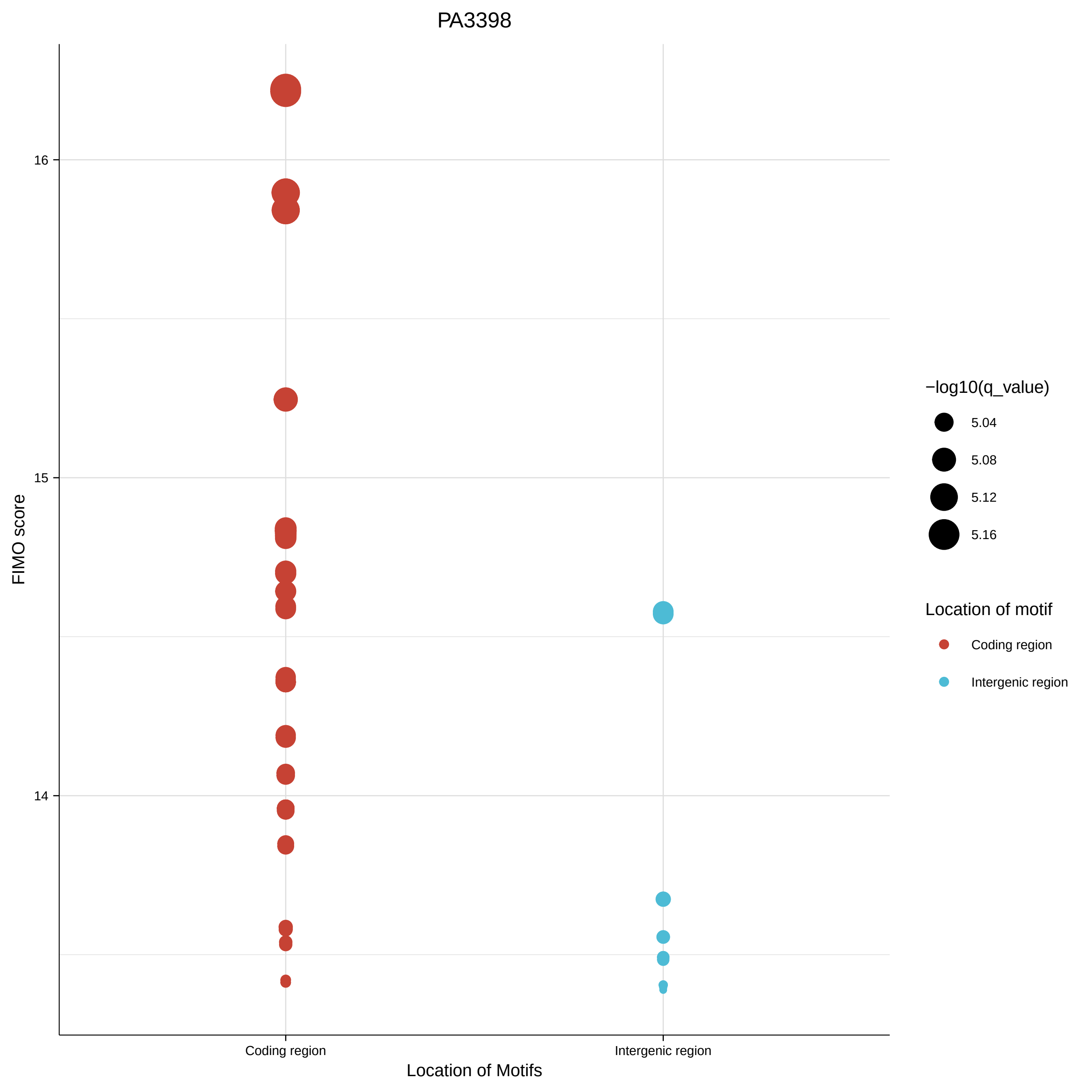
PA3398
FIMO score
16
15
14
Coding region
Intergenic region
Location of Motifs
−log10(q_value)
5.04
5.08
5.12
5.16
Location of motif
Coding region
Intergenic region

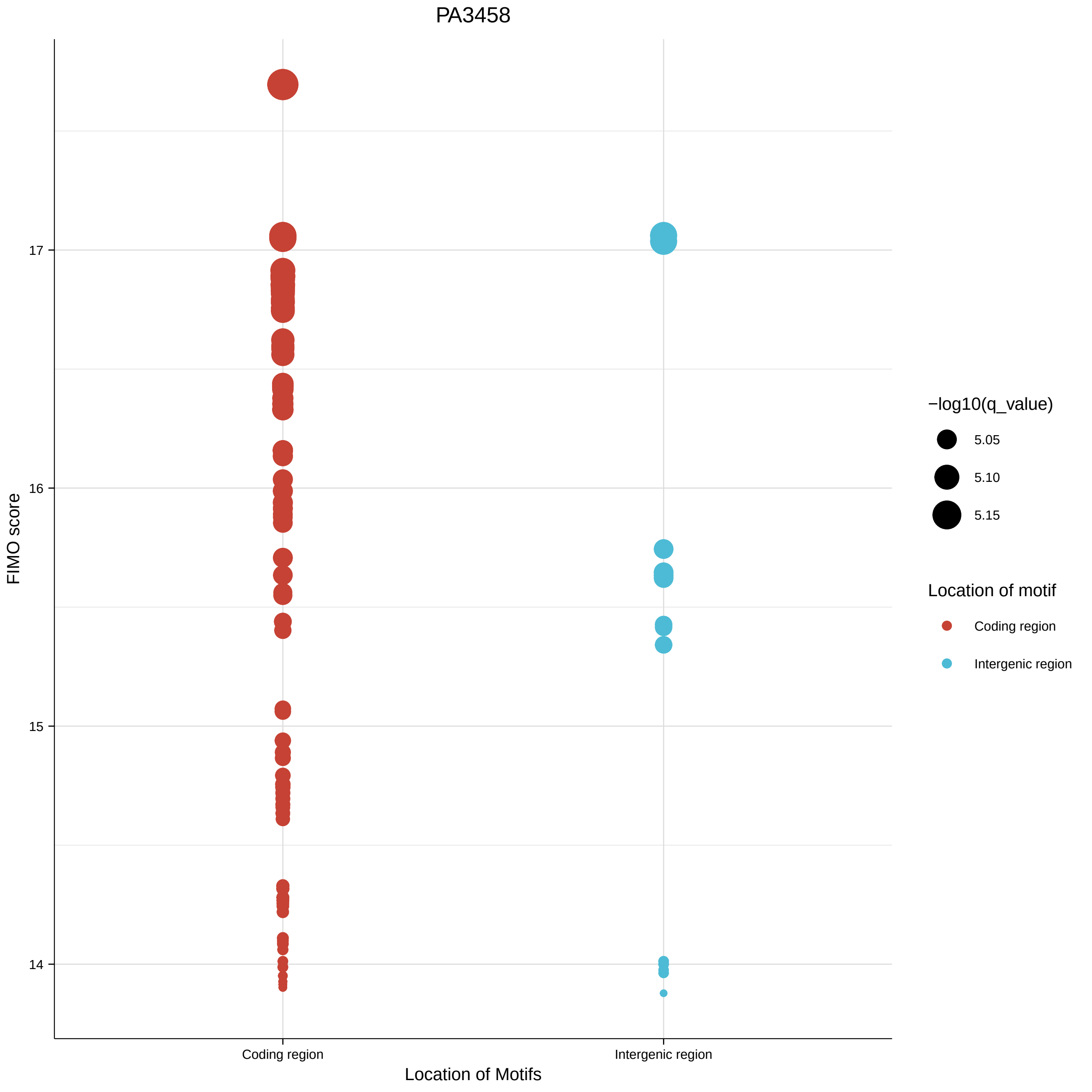
PA3458
FIMO score
17
16
15
14
Coding region
Intergenic region
Location of Motifs
−log10(q_value)
5.05
5.10
5.15
Location of motif
Coding region
Intergenic region

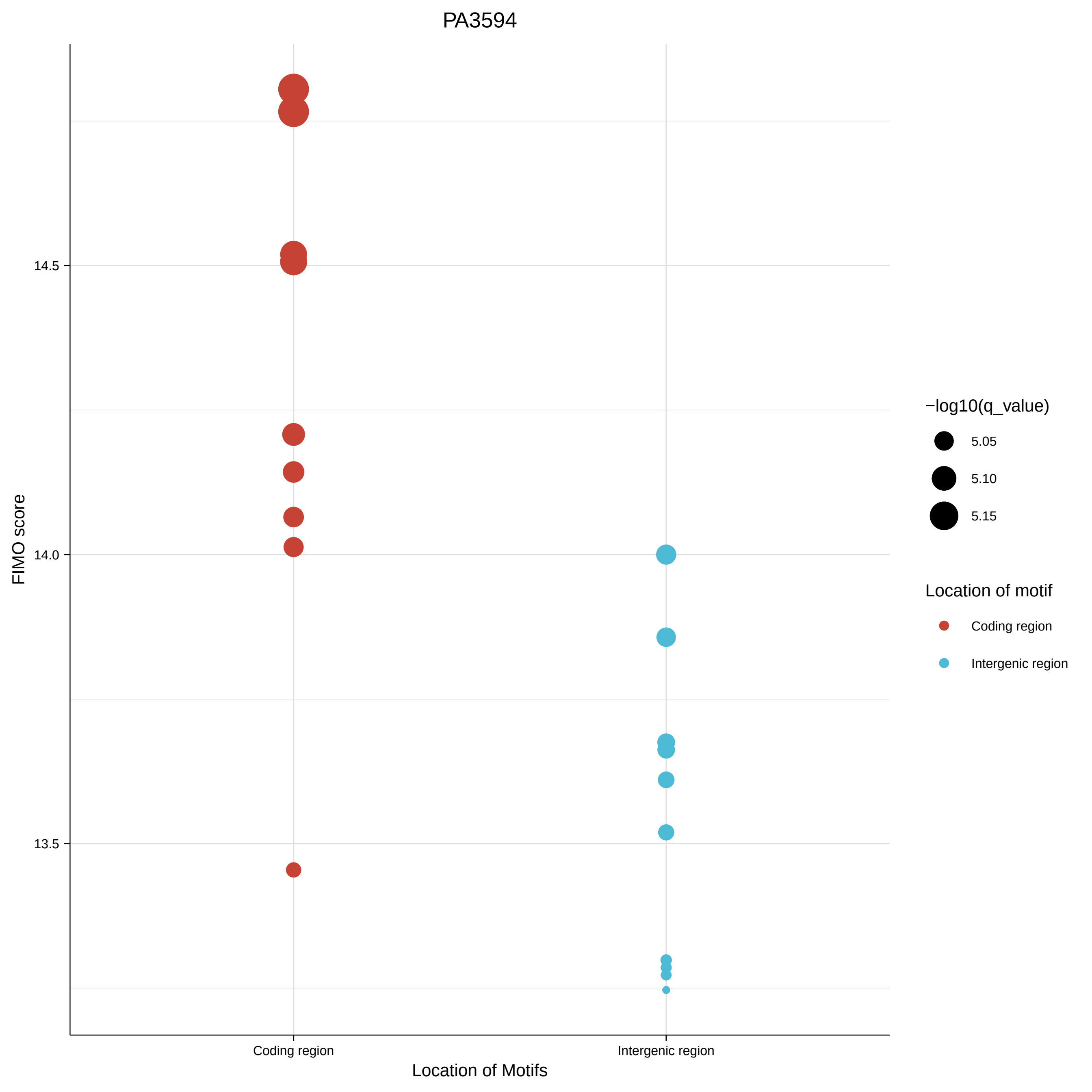

PA3594
FIMO score
14.5
14.0
13.5
Coding region
Intergenic region
Location of Motifs
−log10(q_value)
5.05
5.10
5.15
Location of motif
Coding region
Intergenic region

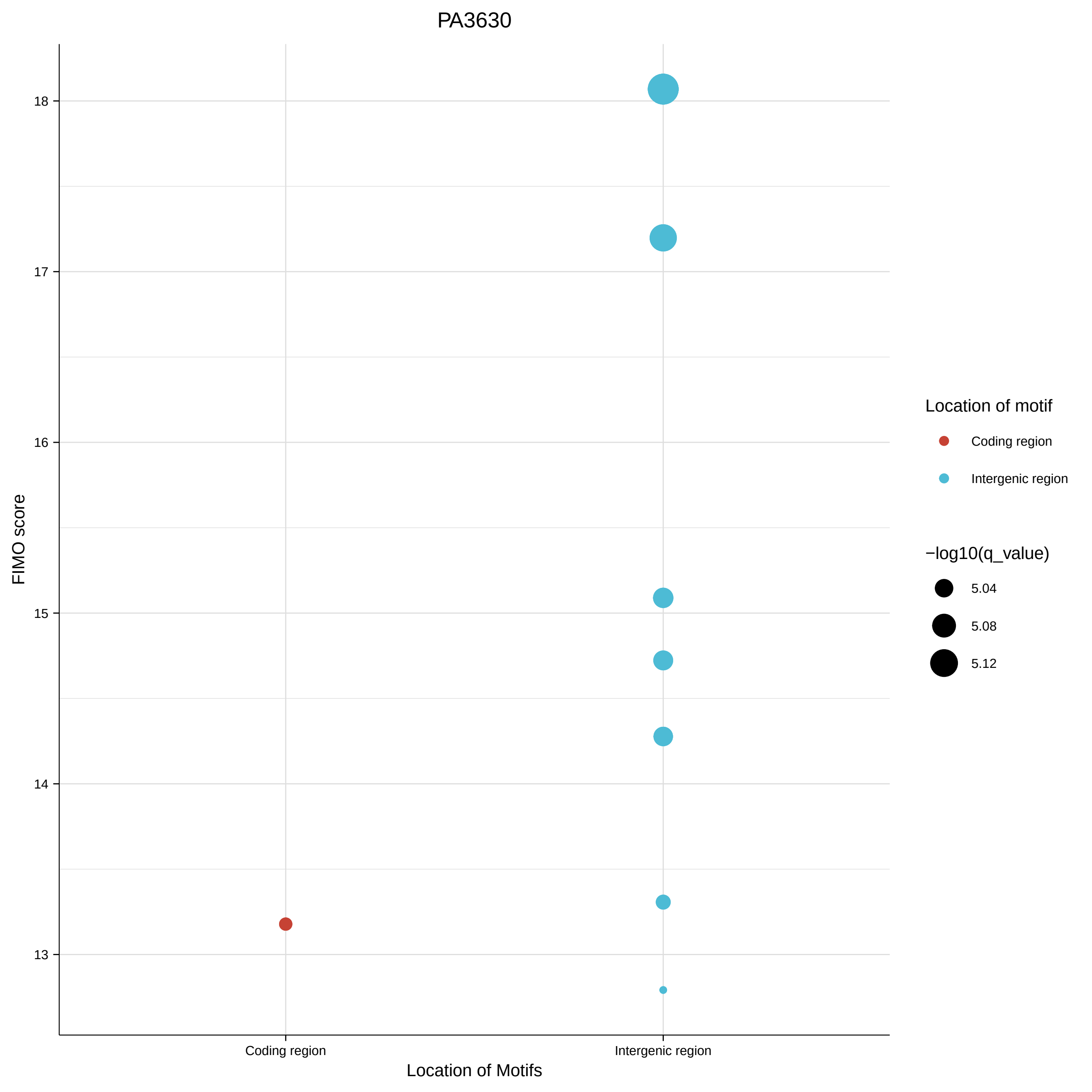
PA3630
FIMO score
18
17
16
15
14
13
Coding region
Intergenic region
Location of Motifs
Location of motif
Coding region
Intergenic region
−log10(q_value)
5.04
5.08
5.12

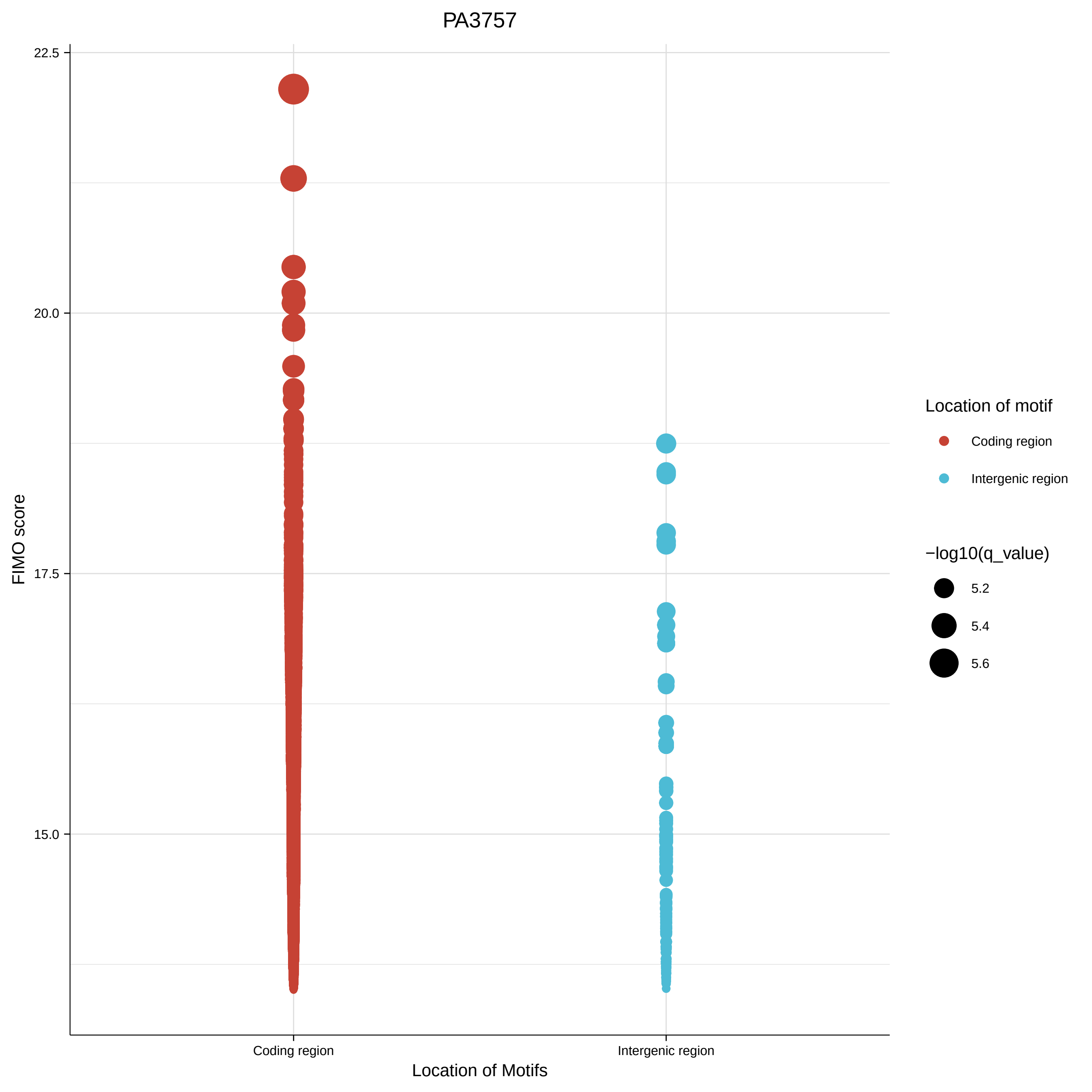

PA3757
22.5
20.0
17.5
15.0
FIMO score
Coding region
Intergenic region
Location of Motifs
Location of motif
Coding region
Intergenic region
−log10(q_value)
5.2
5.4
5.6

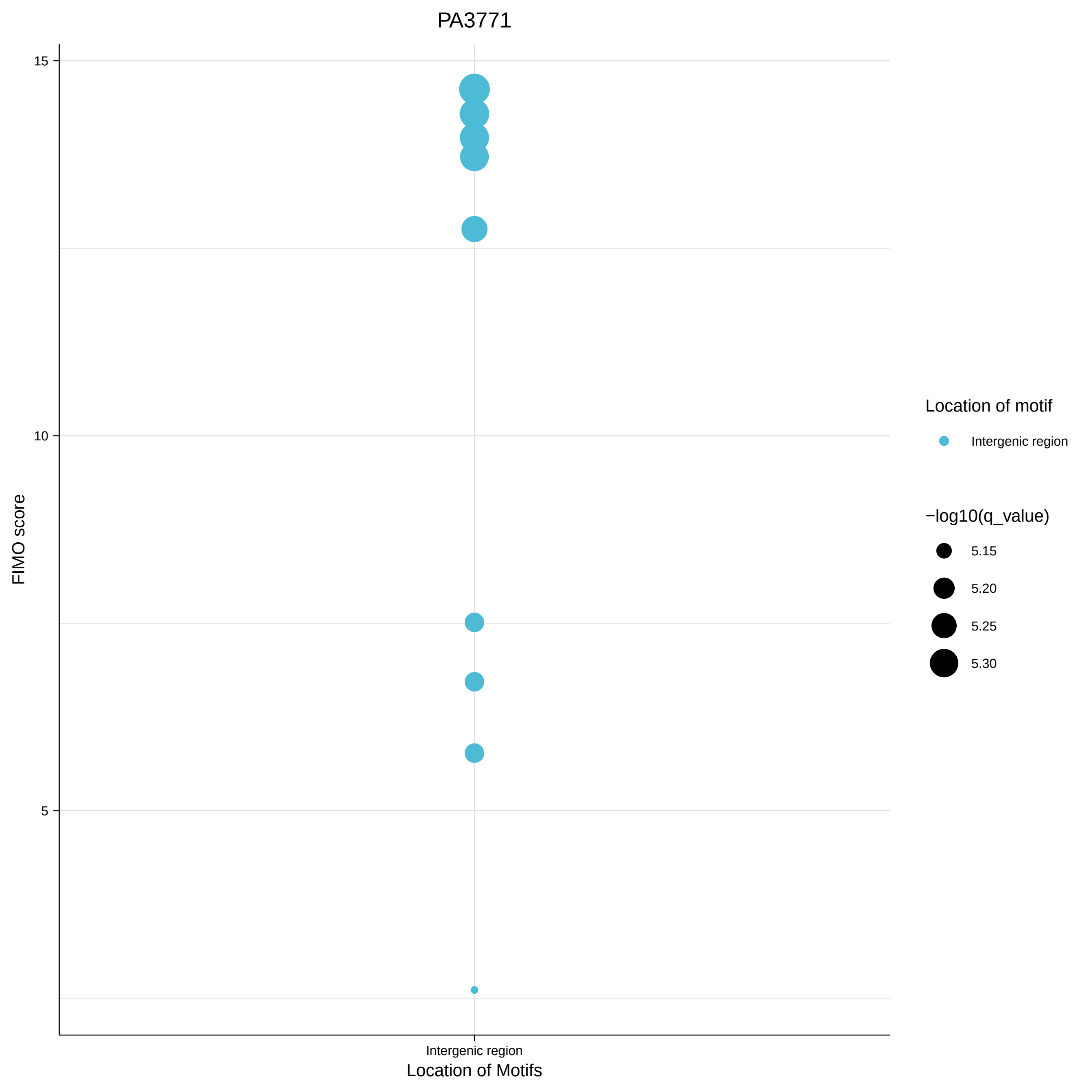
PA3771
FIMO score
15
10
5
Intergenic region
Location of Motifs
Location of motif
Intergenic region
−log10(q_value)
5.15
5.20
5.25
5.30

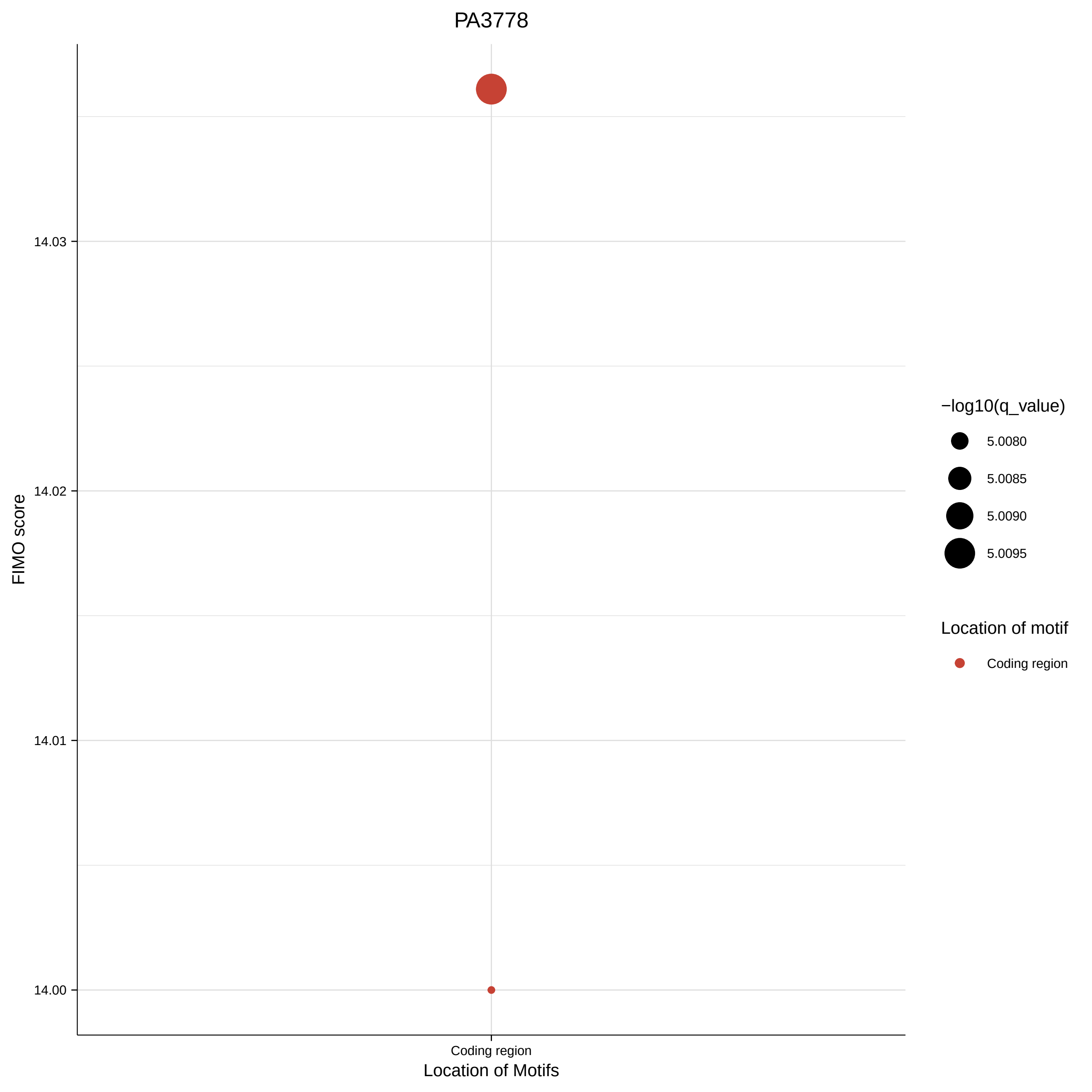
PA3778
FIMO score
14.03
14.02
14.01
14.00
Coding region
Location of Motifs
−log10(q_value)
5.0080
5.0085
5.0090
5.0095
Location of motif
Coding region

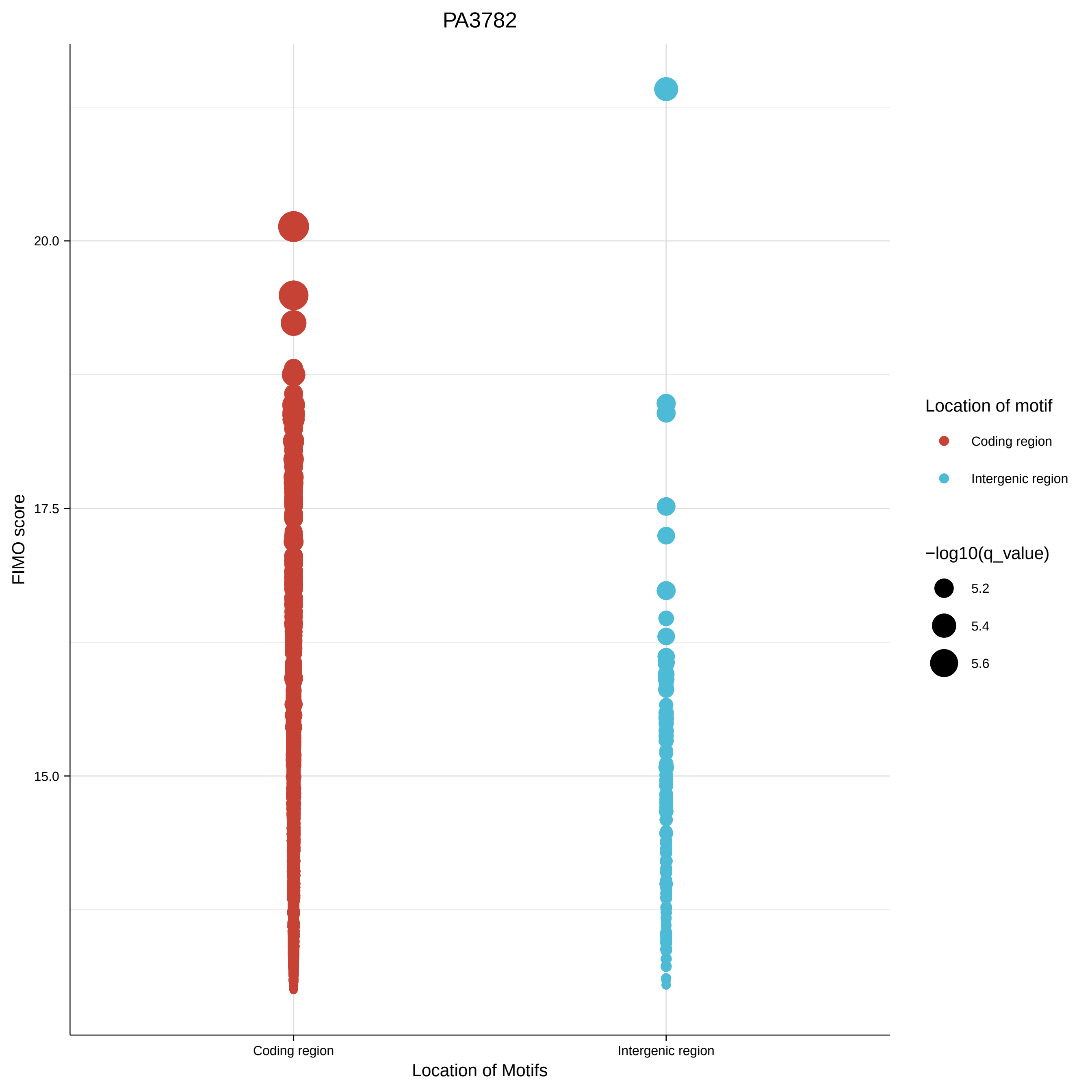
PA3782
FIMO score
20.0
17.5
15.0
Coding region
Intergenic region
Location of Motifs
Location of motif
Coding region
Intergenic region
−log10(q_value)
5.2
5.4
5.6

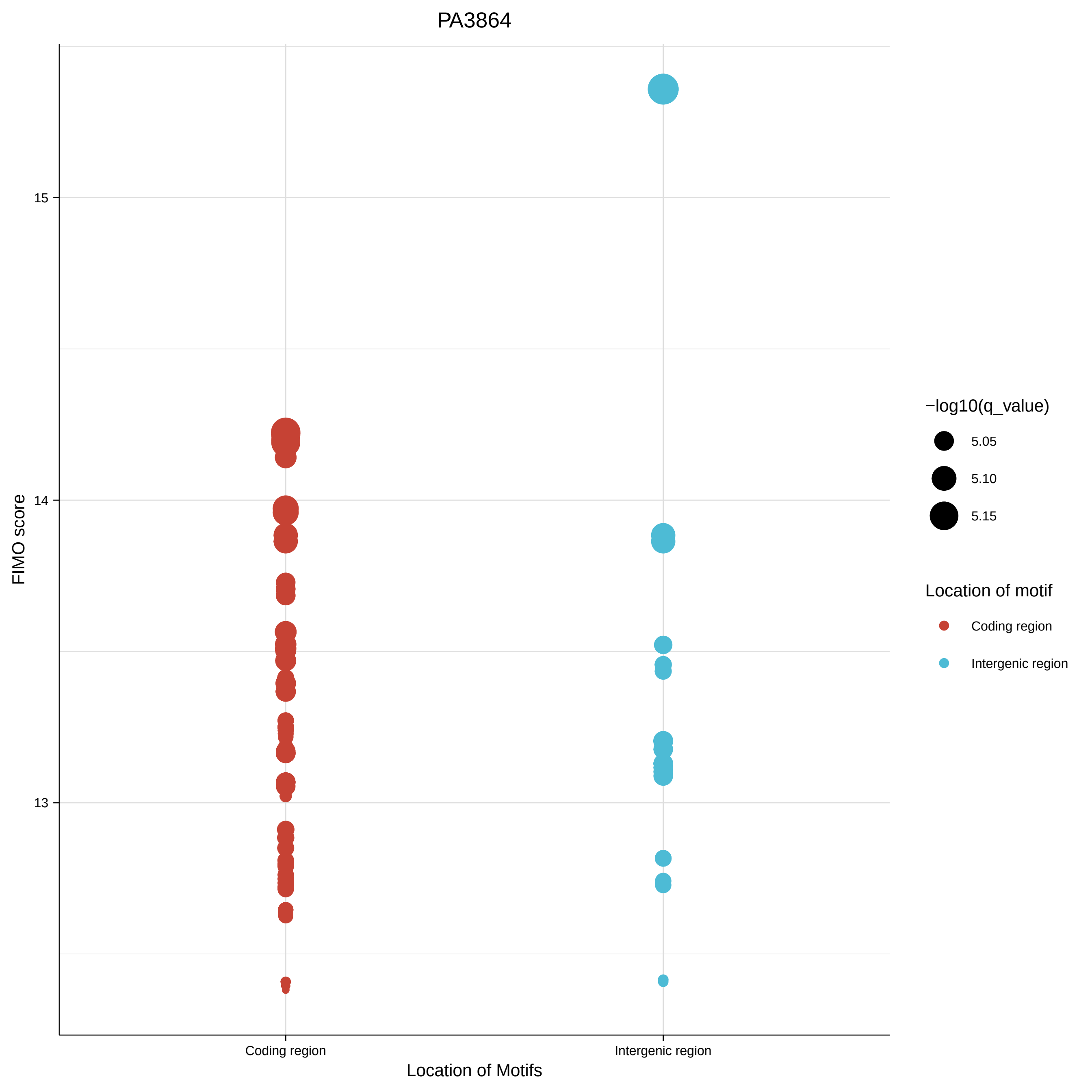
PA3864
FIMO score
15
14
13
Coding region
Intergenic region
Location of Motifs
−log10(q_value)
5.05
5.10
5.15
Location of motif
Coding region
Intergenic region

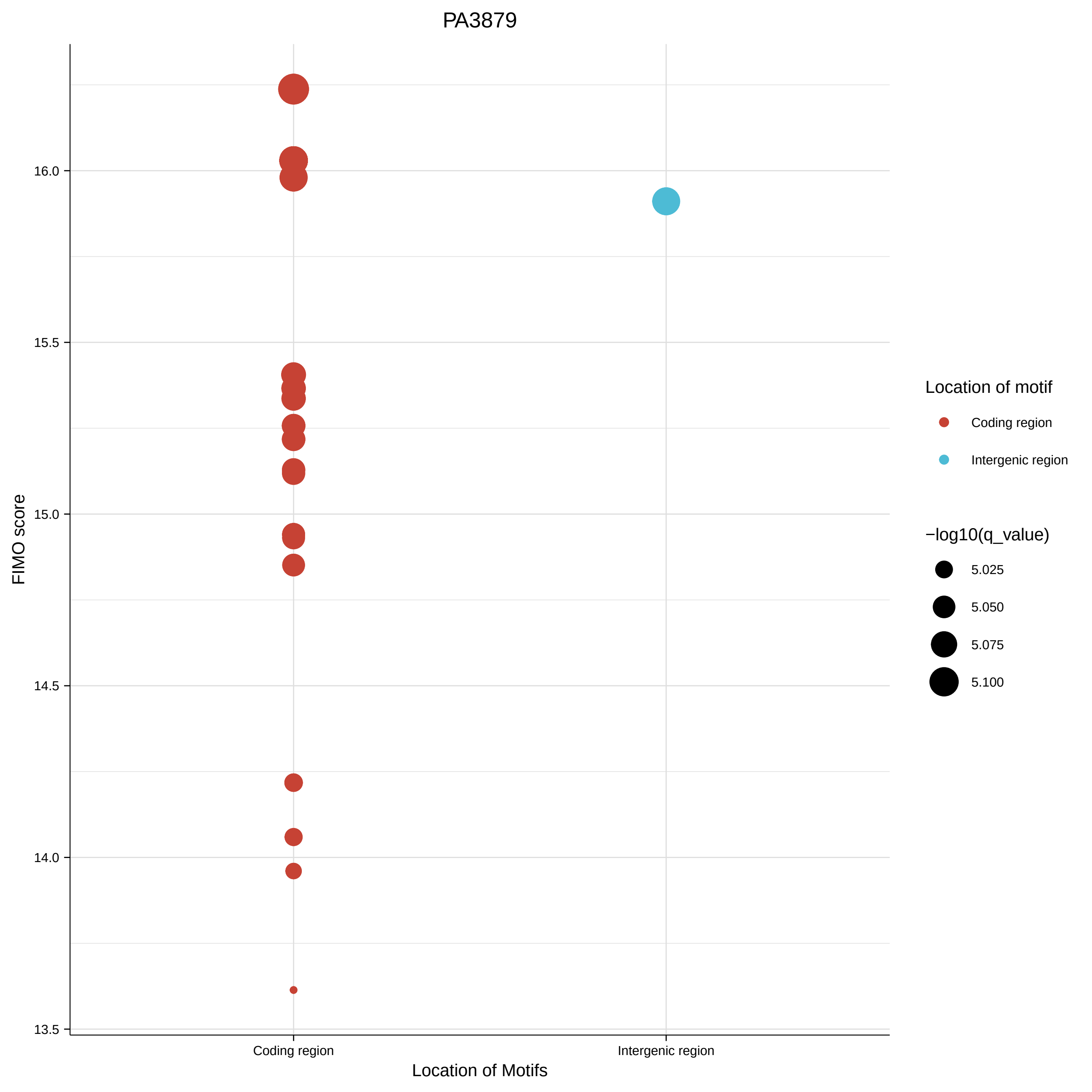

PA3879
FIMO score
16.0
15.5
15.0
14.5
14.0
13.5
Coding region
Intergenic region
Location of Motifs
Location of motif
Coding region
Intergenic region
−log10(q_value)
5.025
5.050
5.075
5.100

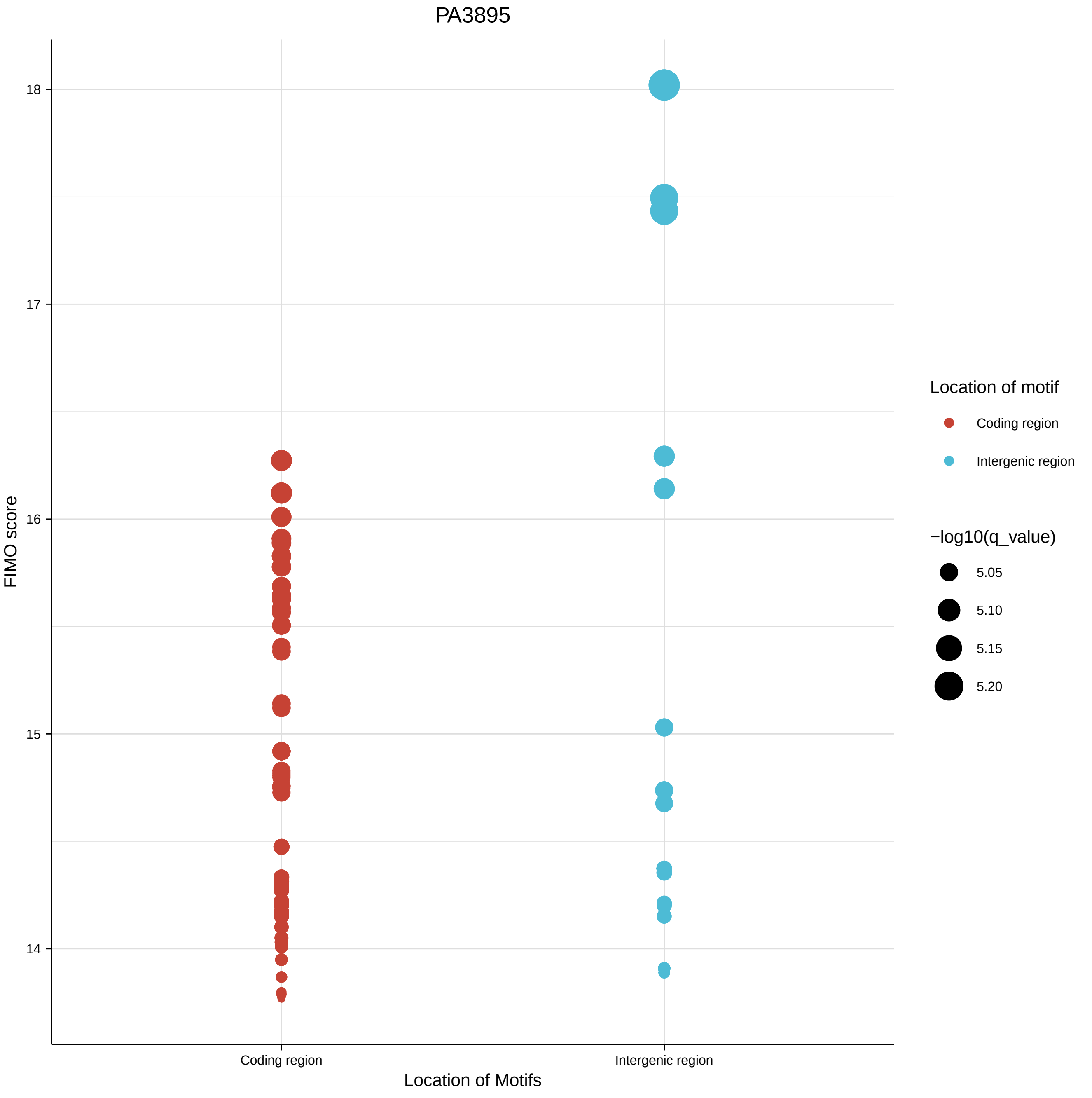

PA3895
FIMO score
18
17
16
15
14
Coding region
Intergenic region
Location of Motifs
Location of motif
Coding region
Intergenic region
−log10(q_value)
5.05
5.10
5.15
5.20

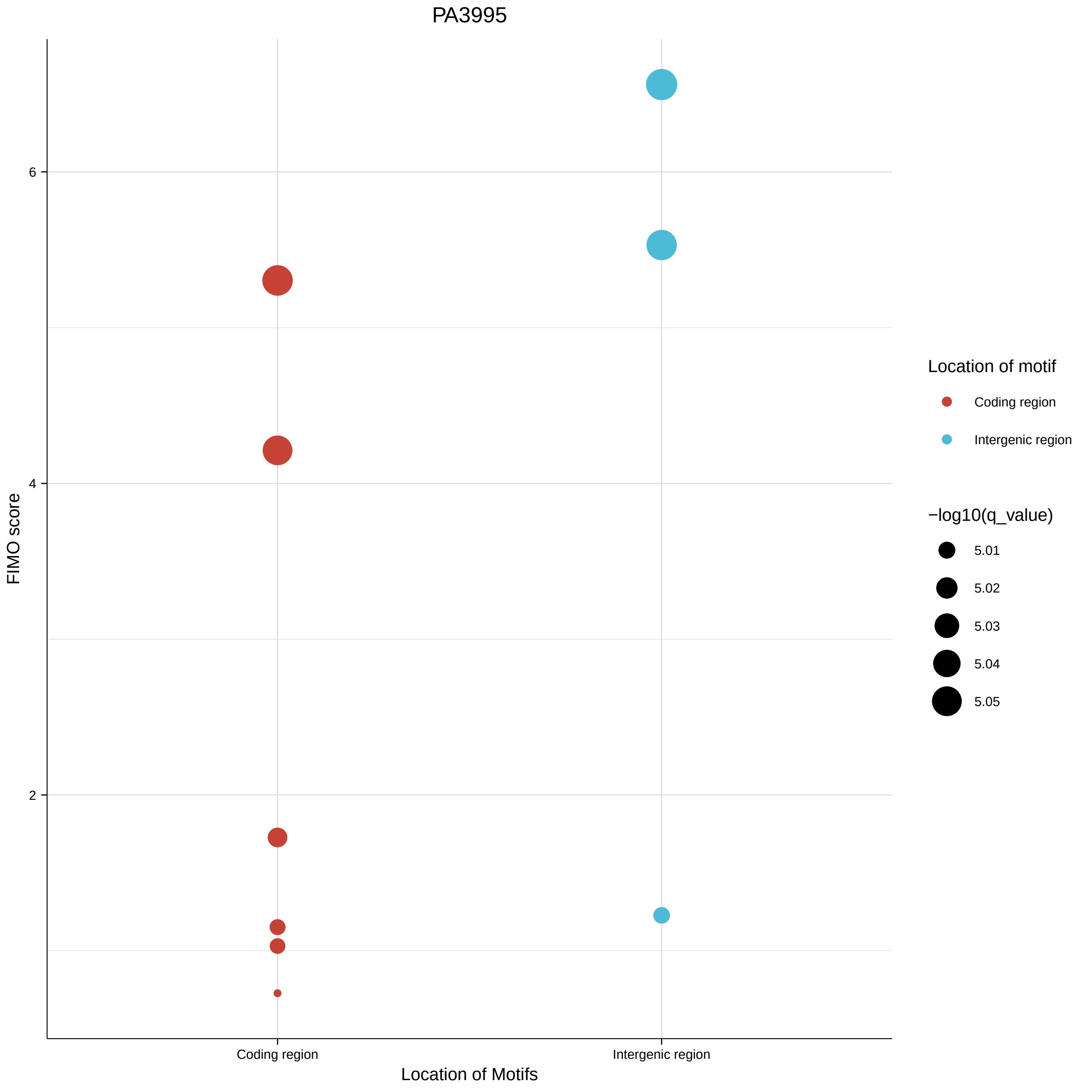
PA3995
FIMO score
6
4
2
Coding region
Intergenic region
Location of Motifs
Location of motif
Coding region
Intergenic region
−log10(q_value)
5.01
5.02
5.03
5.04
5.05

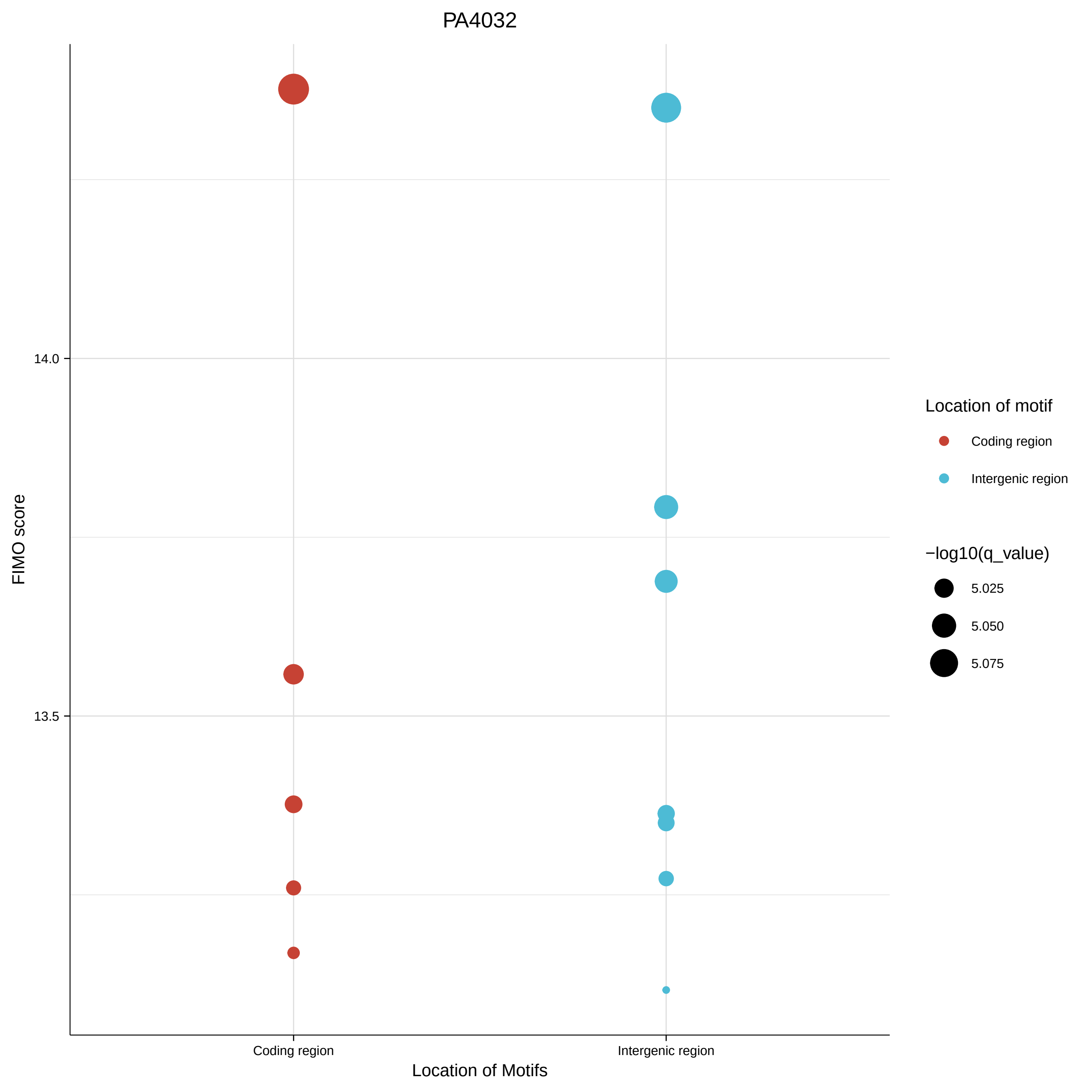

PA4032
FIMO score
14.0
13.5
Coding region
Intergenic region
Location of Motifs
Location of motif
Coding region
Intergenic region
−log10(q_value)
5.025
5.050
5.075

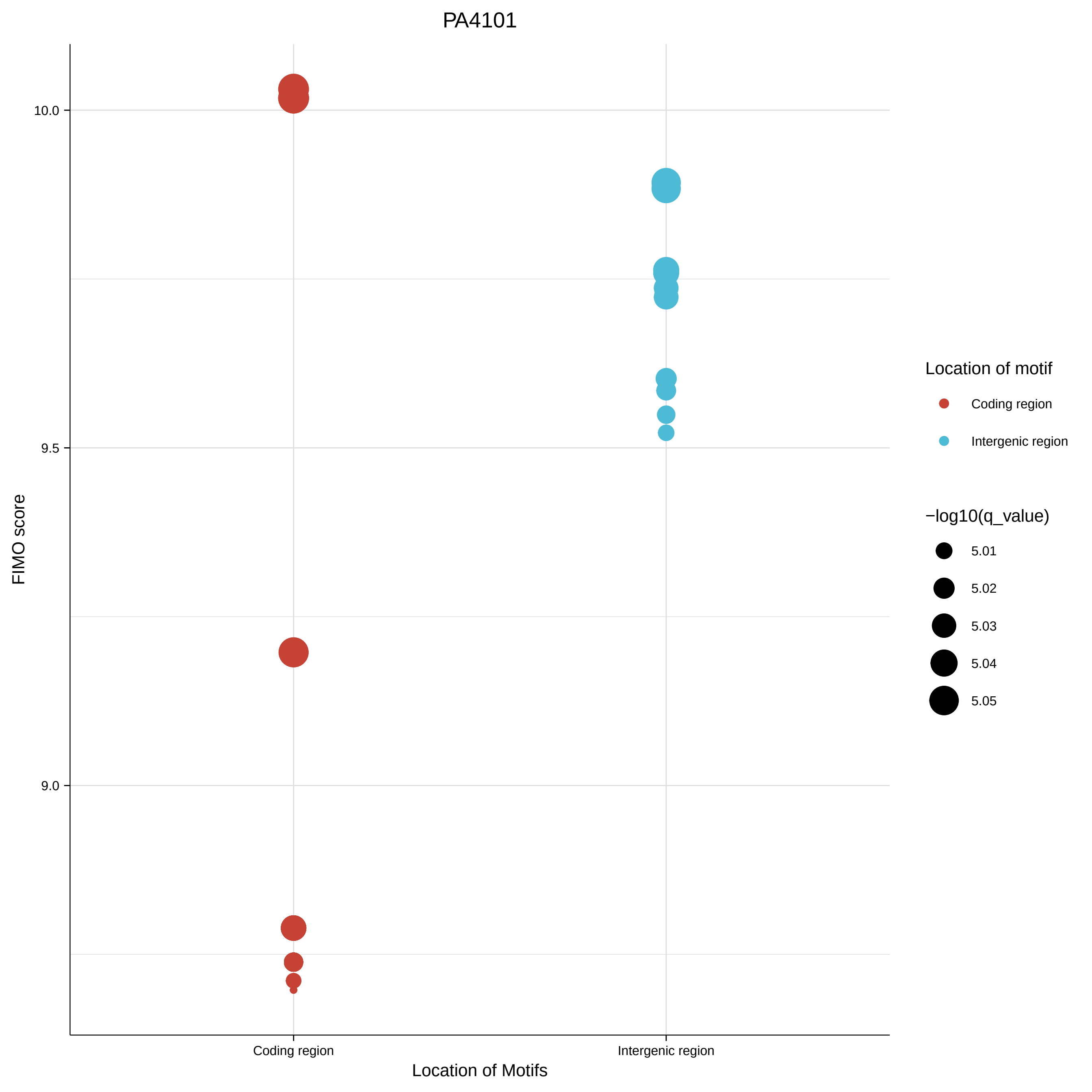
PA4101
FIMO score
10.0
9.5
9.0
Coding region
Intergenic region
Location of Motifs
Location of motif
Coding region
Intergenic region
−log10(q_value)
5.01
5.02
5.03
5.04
5.05

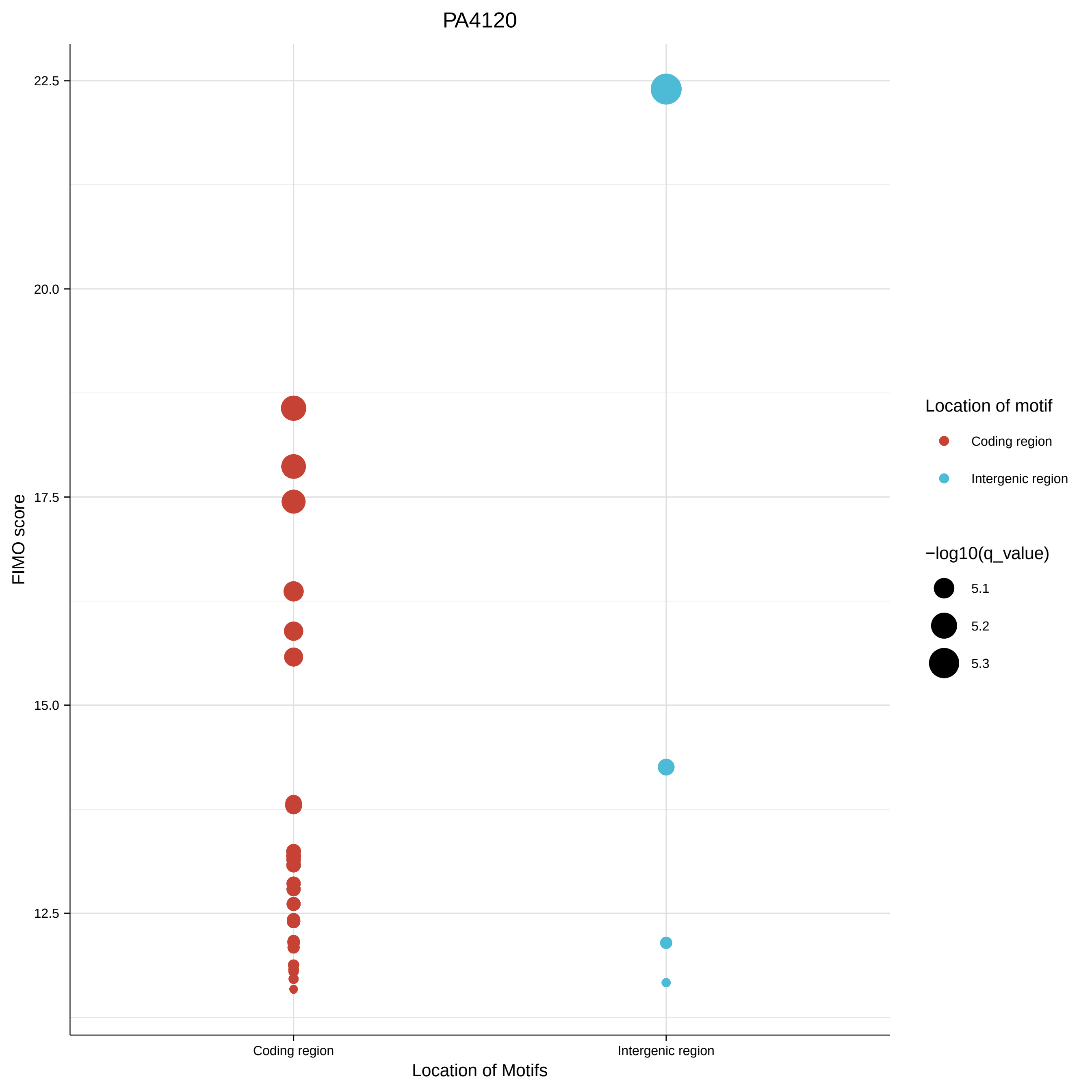
PA4120
FIMO score
22.5
20.0
17.5
15.0
12.5
Coding region
Intergenic region
Location of Motifs
Location of motif
Coding region
Intergenic region
−log10(q_value)
5.1
5.2
5.3

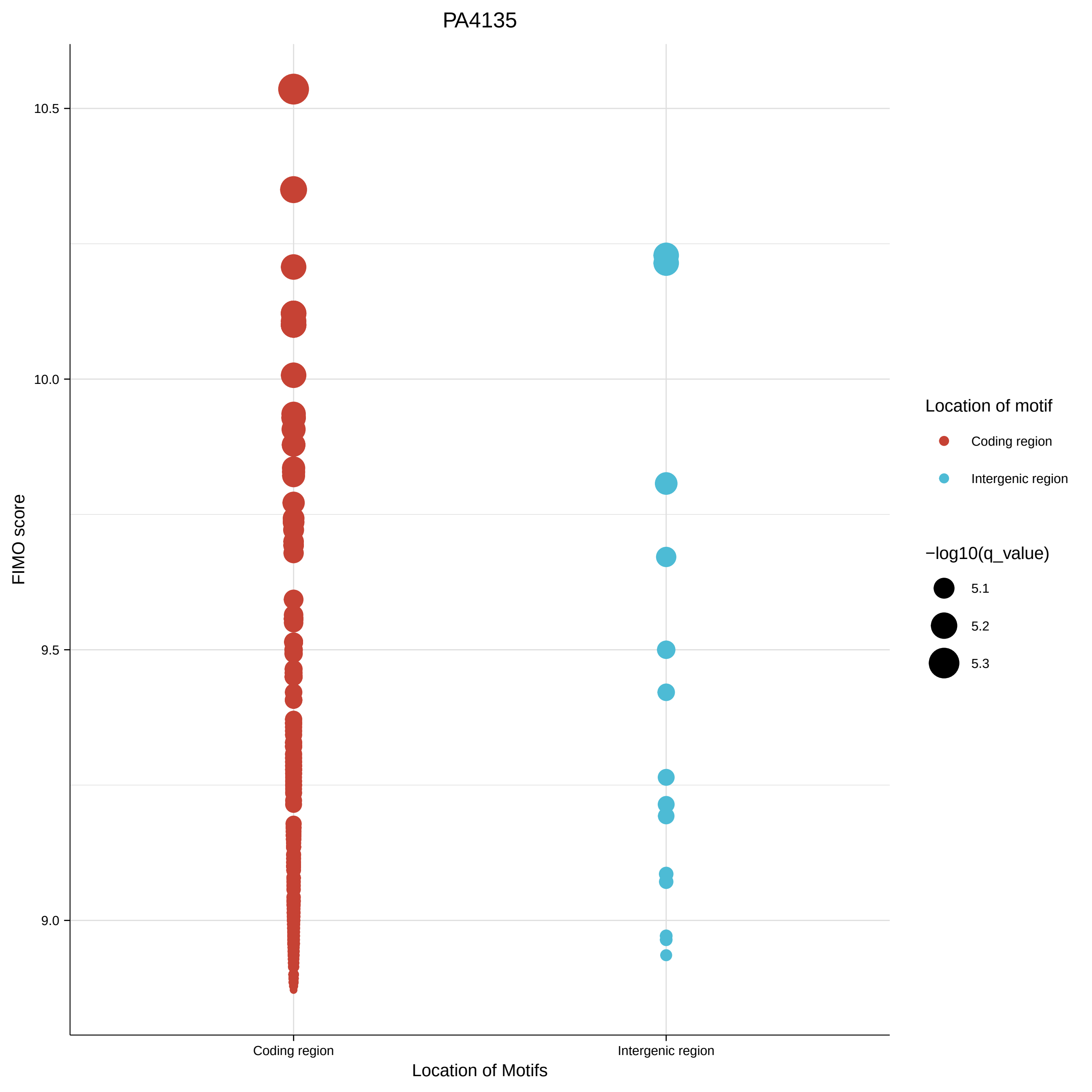

PA4135
FIMO score
10.5
10.0
9.5
9.0
Coding region
Intergenic region
Location of Motifs
Location of motif
Coding region
Intergenic region
−log10(q_value)
5.1
5.2
5.3

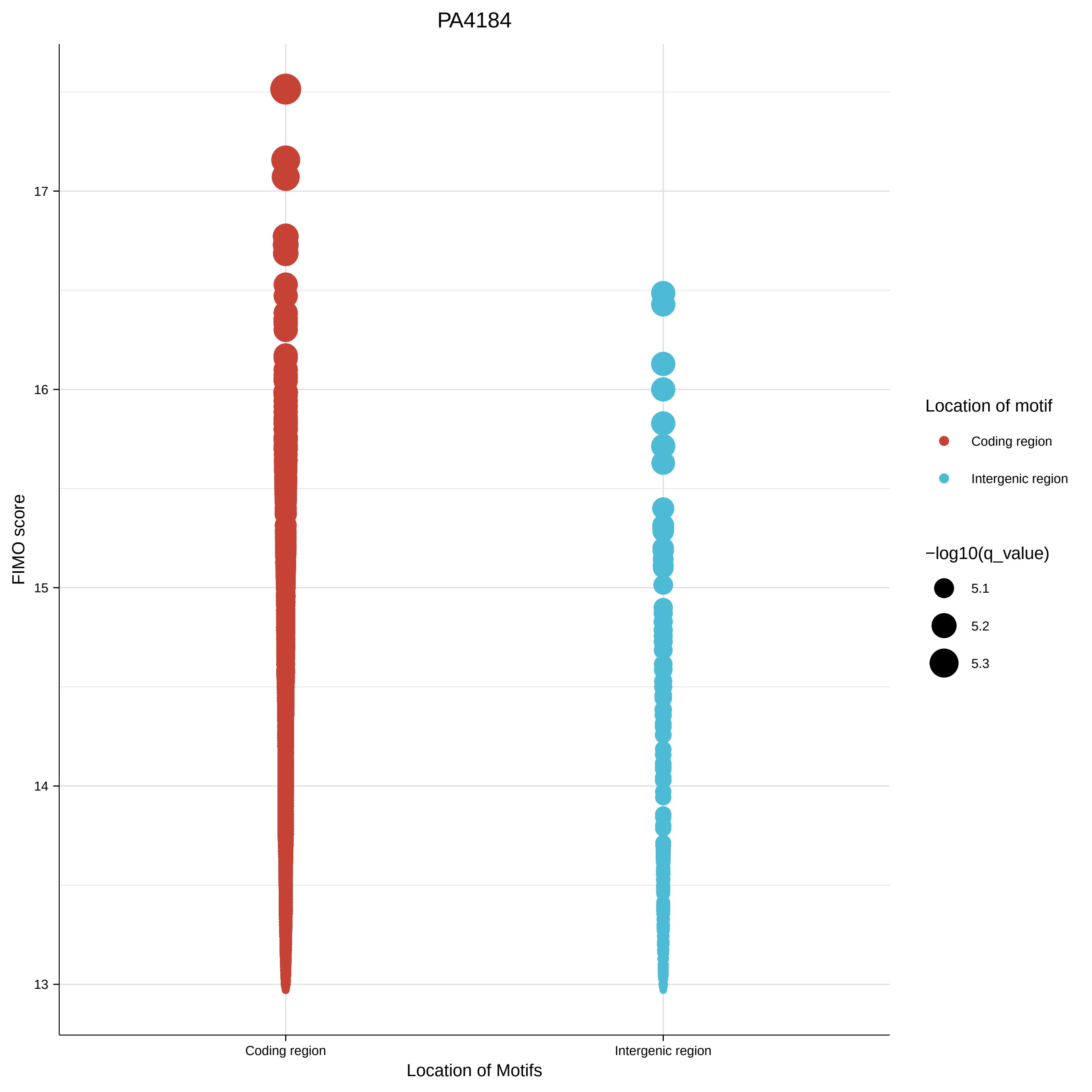

PA4184
FIMO score
17
16
15
14
13
Coding region
Intergenic region
Location of Motifs
Location of motif
Coding region
Intergenic region
−log10(q_value)
5.1
5.2
5.3

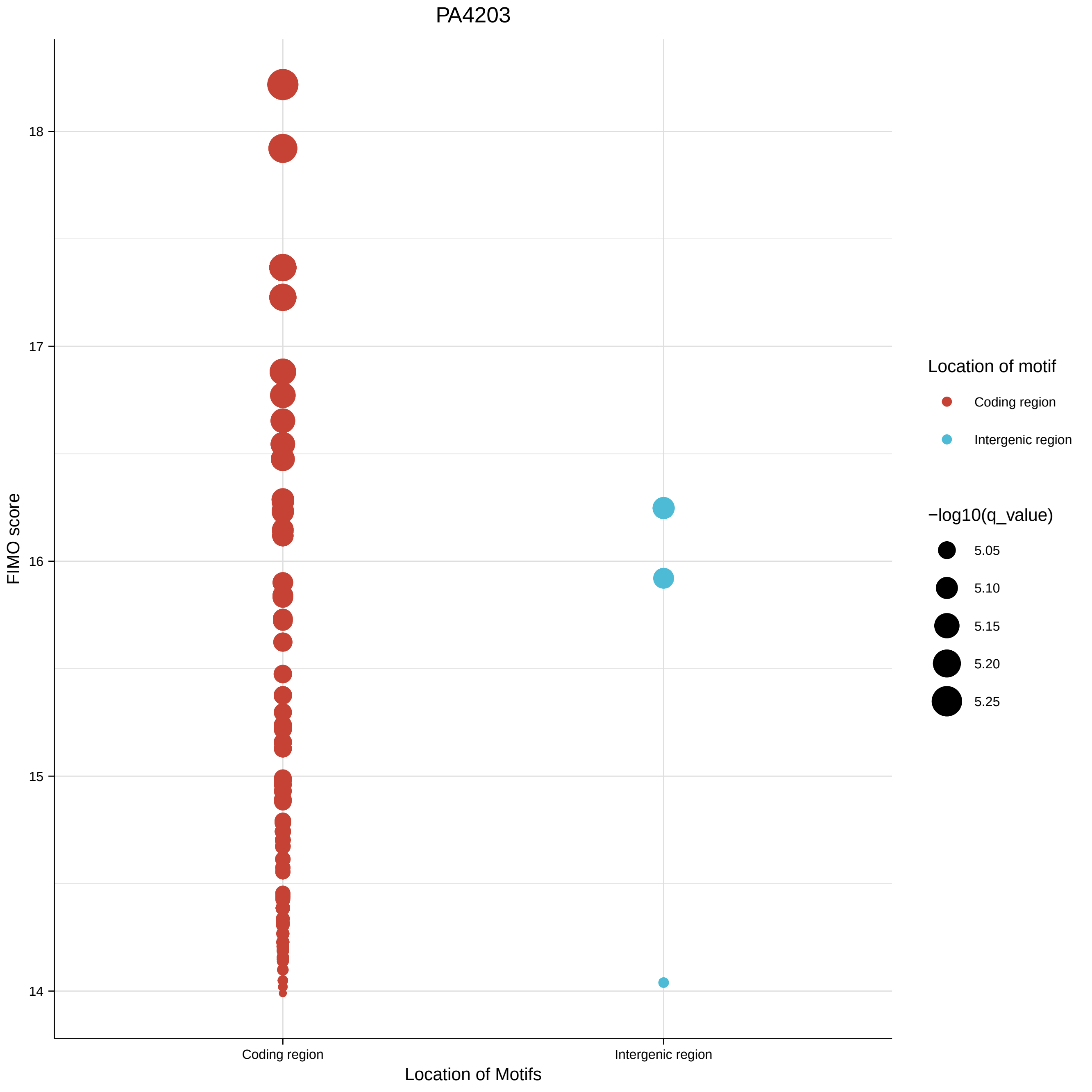
PA4203
FIMO score
18
17
16
15
14
Coding region
Intergenic region
Location of Motifs
Location of motif
Coding region
Intergenic region
−log10(q_value)
5.05
5.10
5.15
5.20
5.25

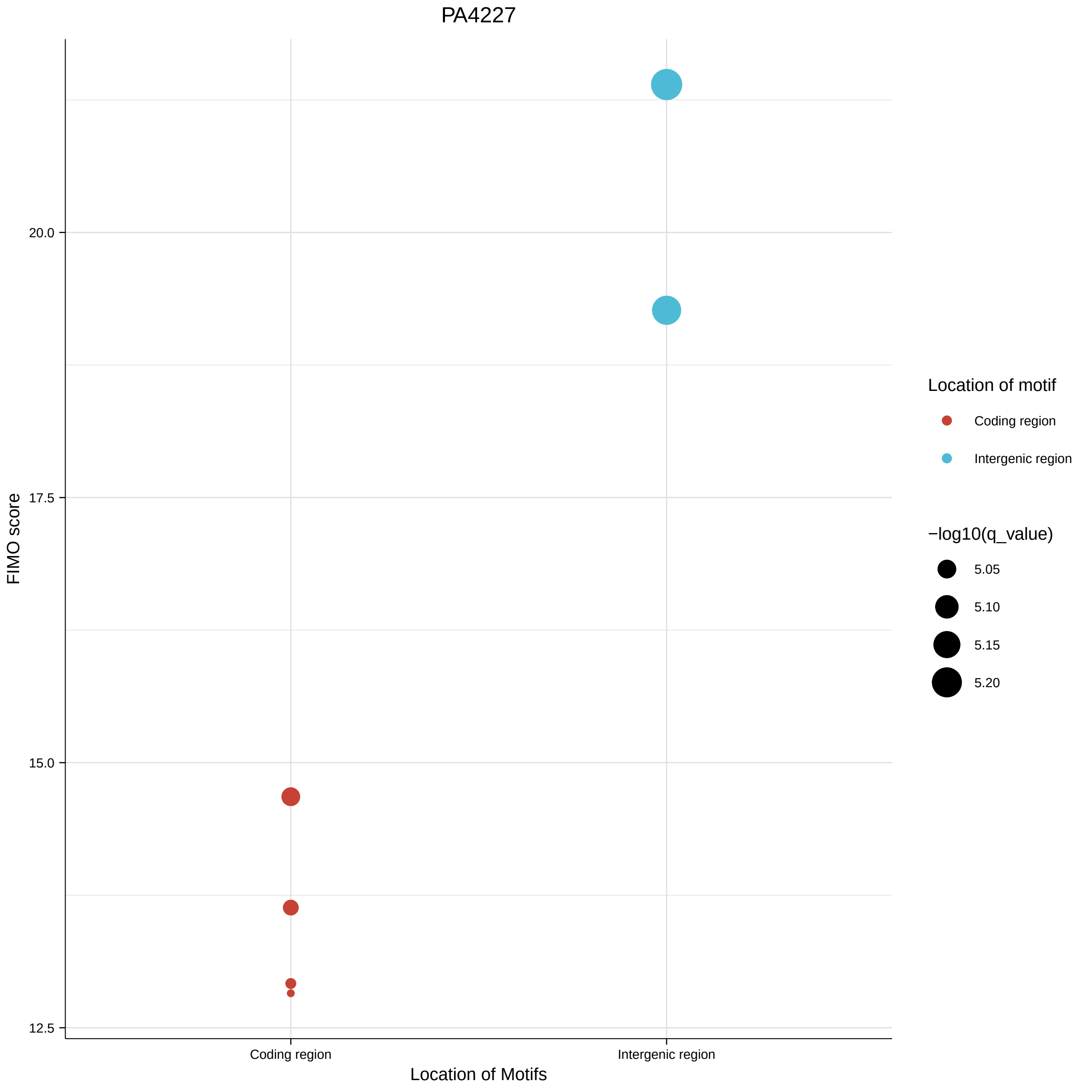
PA4227
FIMO score
20.0
17.5
15.0
12.5
Coding region
Intergenic region
Location of Motifs
Location of motif
Coding region
Intergenic region
−log10(q_value)
5.05
5.10
5.15
5.20

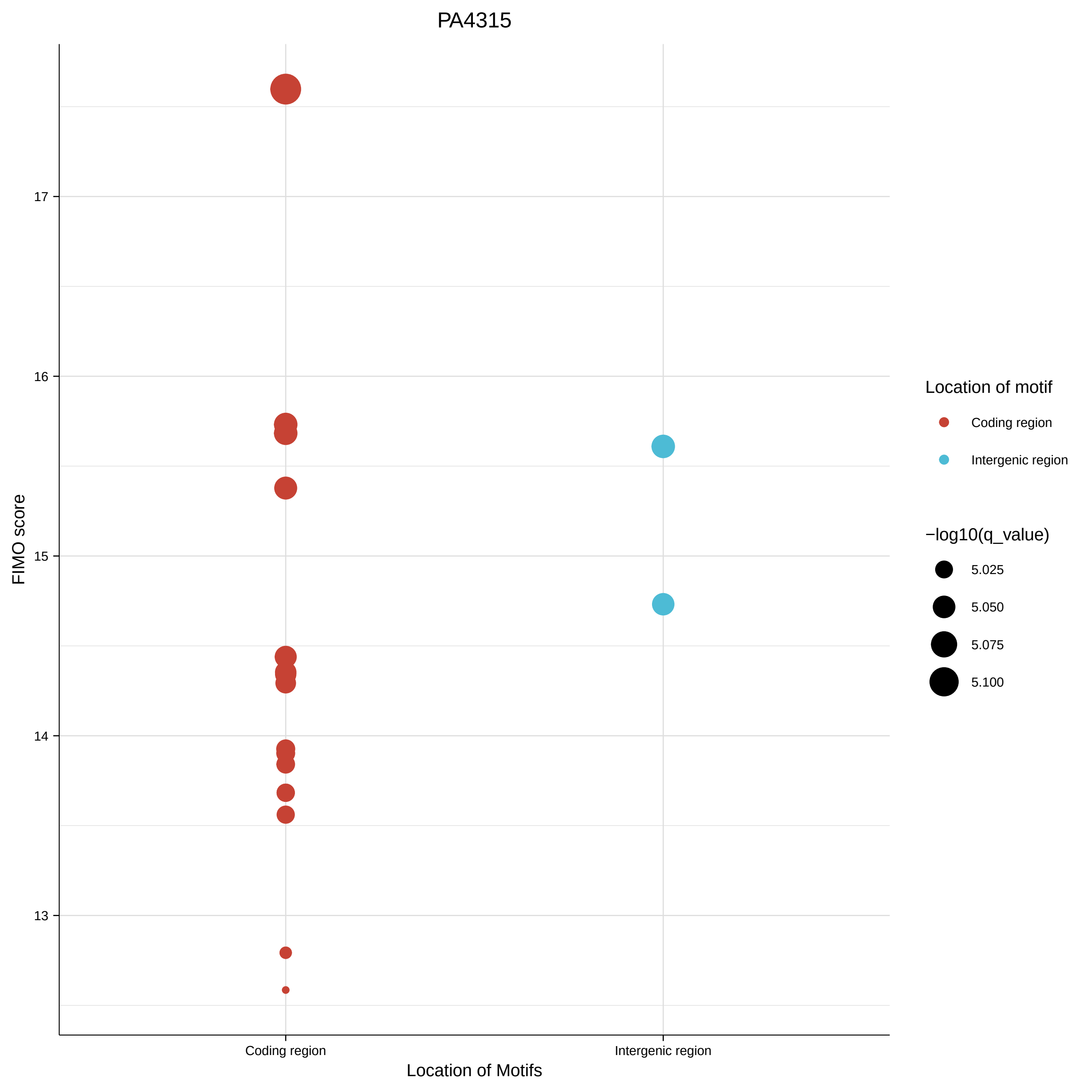

PA4315
FIMO score
17
16
15
14
13
Coding region
Intergenic region
Location of Motifs
Location of motif
Coding region
Intergenic region
−log10(q_value)
5.025
5.050
5.075
5.100

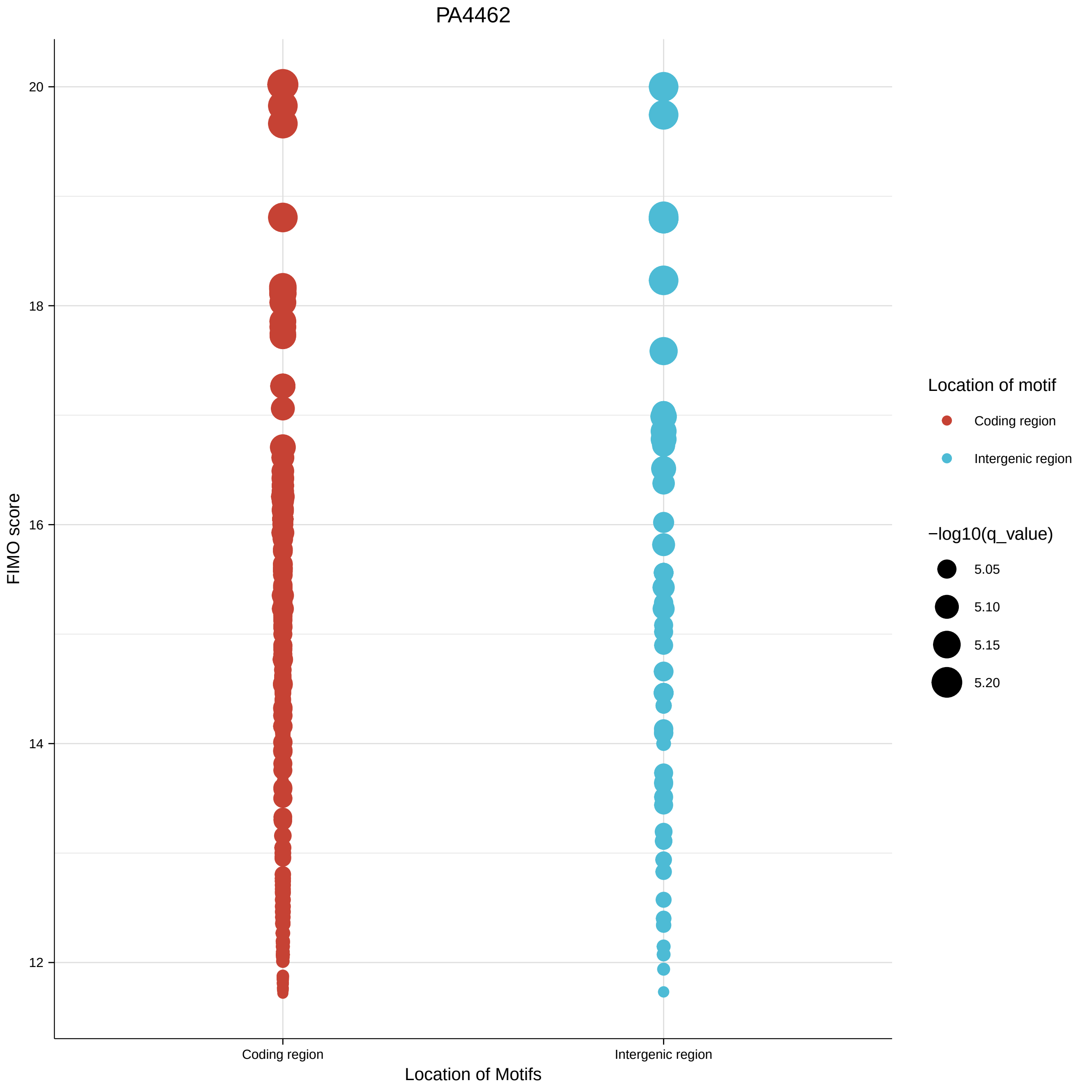
PA4462
FIMO score
20
18
16
14
12
Coding region
Intergenic region
Location of Motifs
Location of motif
Coding region
Intergenic region
−log10(q_value)
5.05
5.10
5.15
5.20

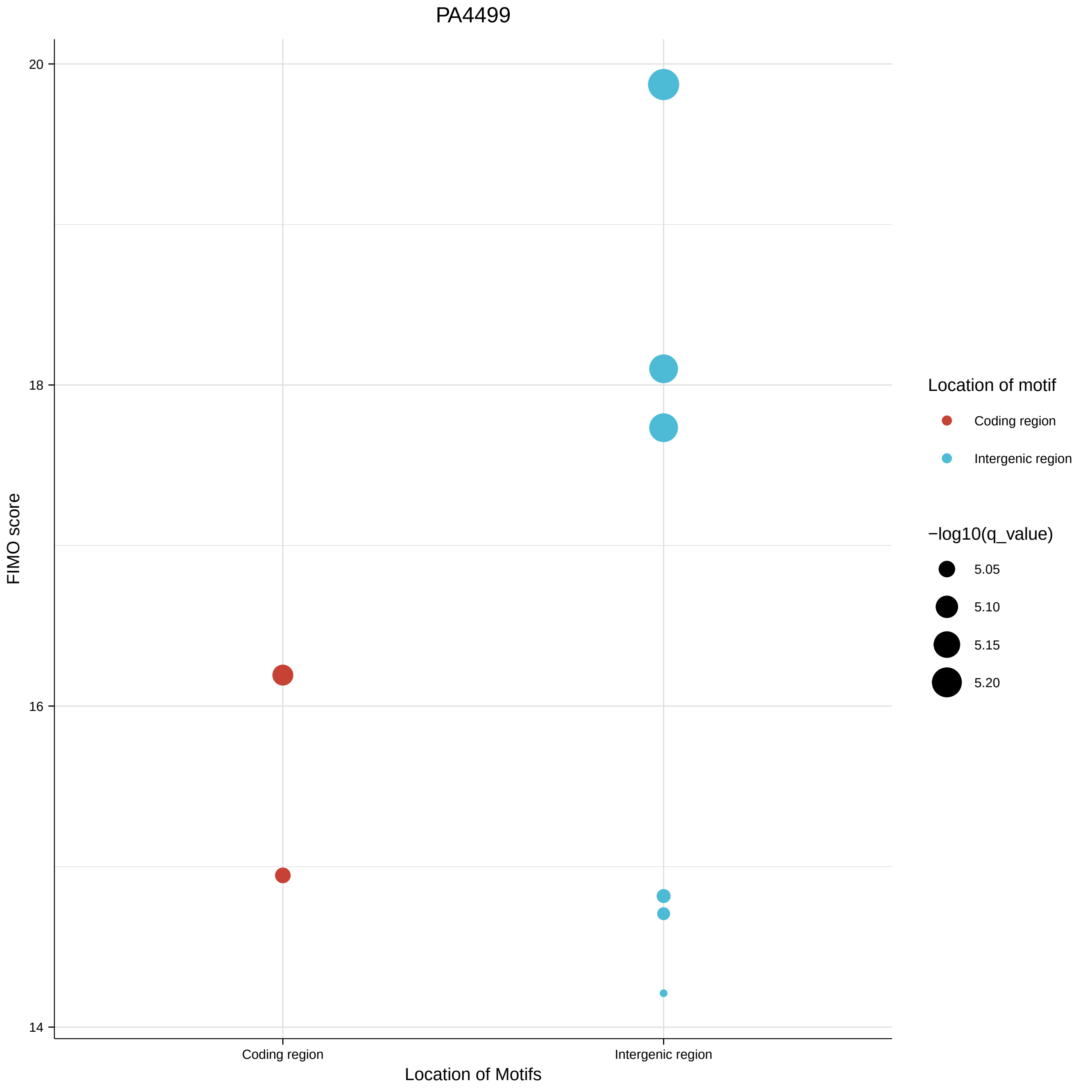
PA4499
FIMO score
20
18
16
14
Coding region
Intergenic region
Location of Motifs
Location of motif
Coding region
Intergenic region
−log10(q_value)
5.05
5.10
5.15
5.20

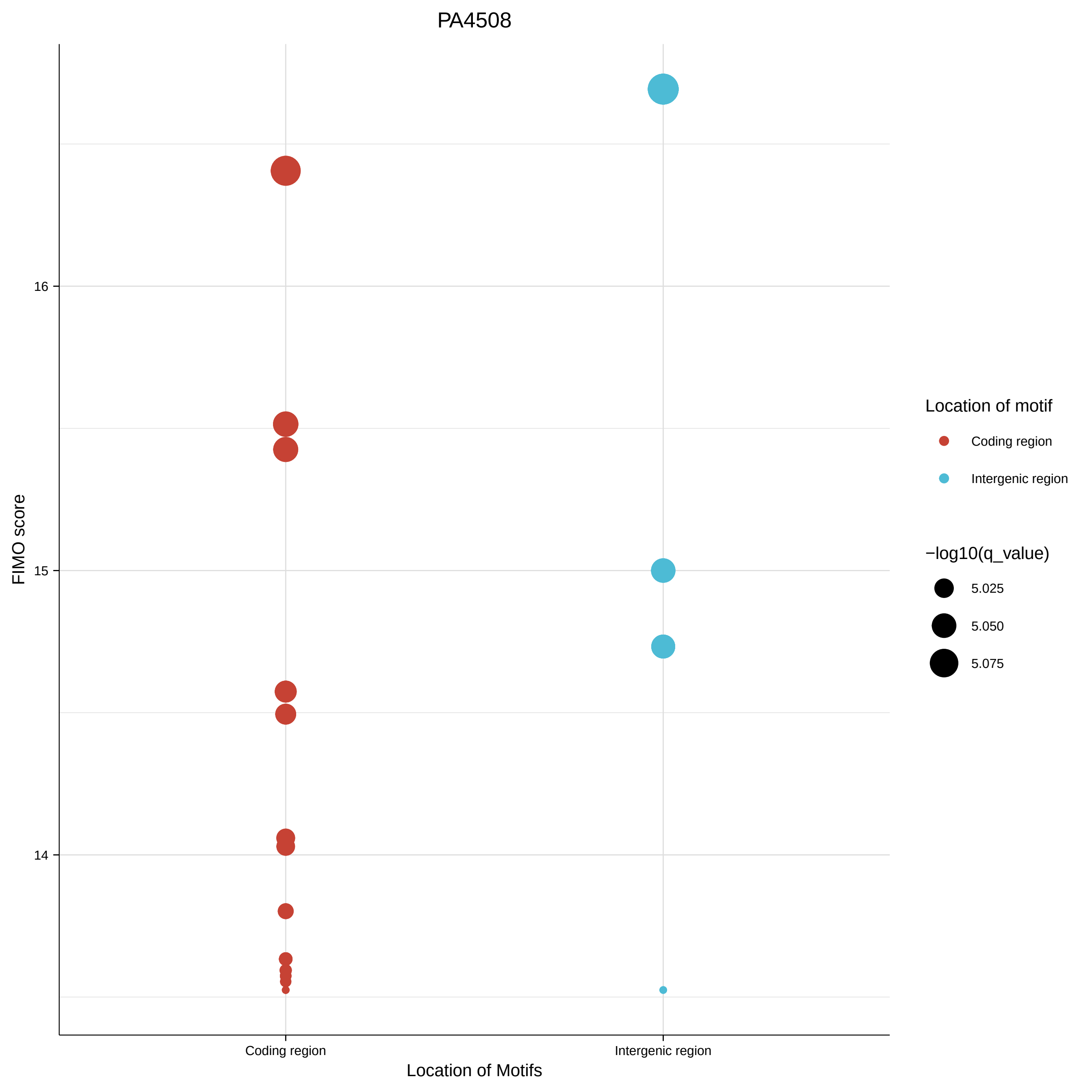
PA4508
FIMO score
16
15
14
Coding region
Intergenic region
Location of Motifs
Location of motif
Coding region
Intergenic region
−log10(q_value)
5.025
5.050
5.075

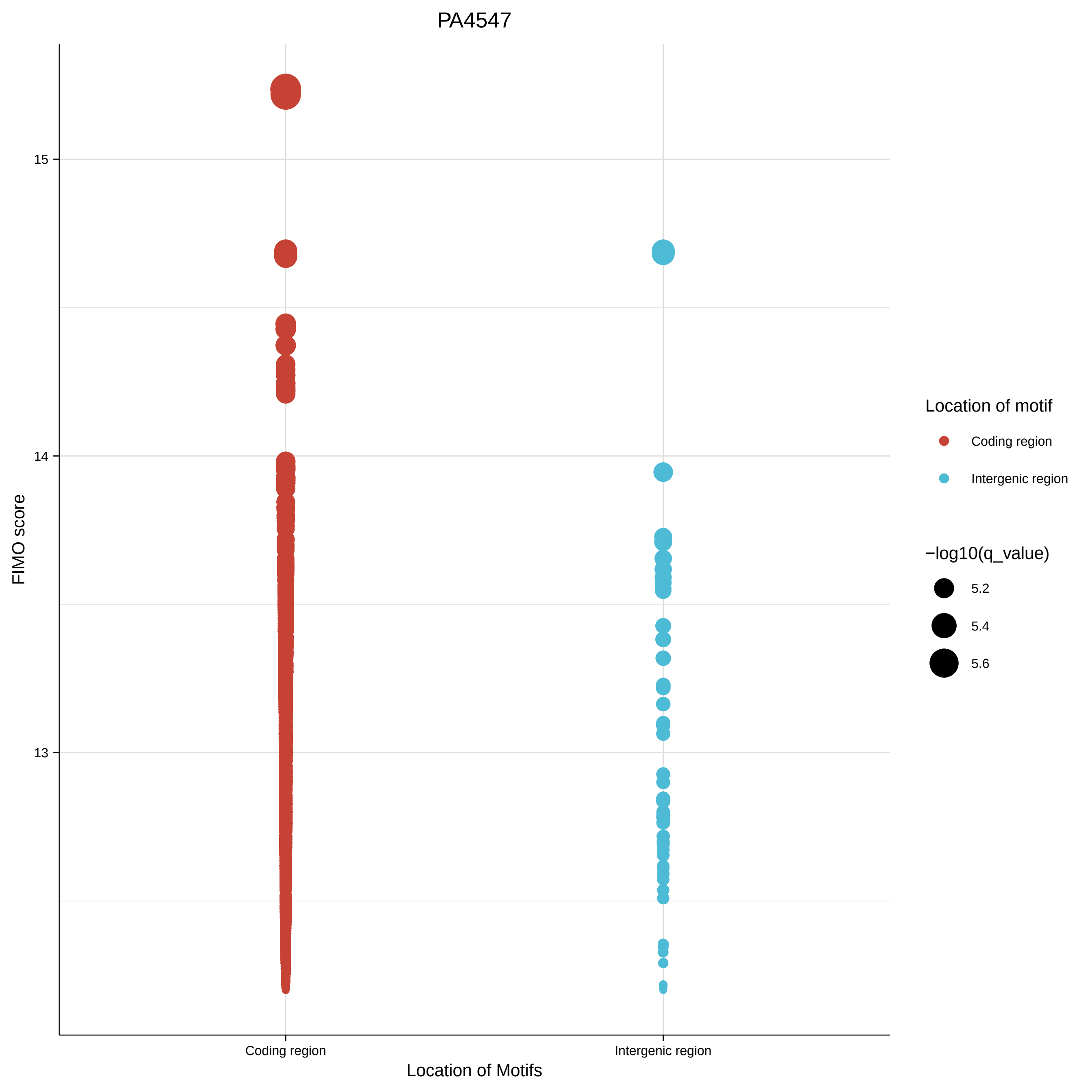

PA4547
FIMO score
15
14
13
Coding region
Intergenic region
Location of Motifs
Location of motif
Coding region
Intergenic region
−log10(q_value)
5.2
5.4
5.6

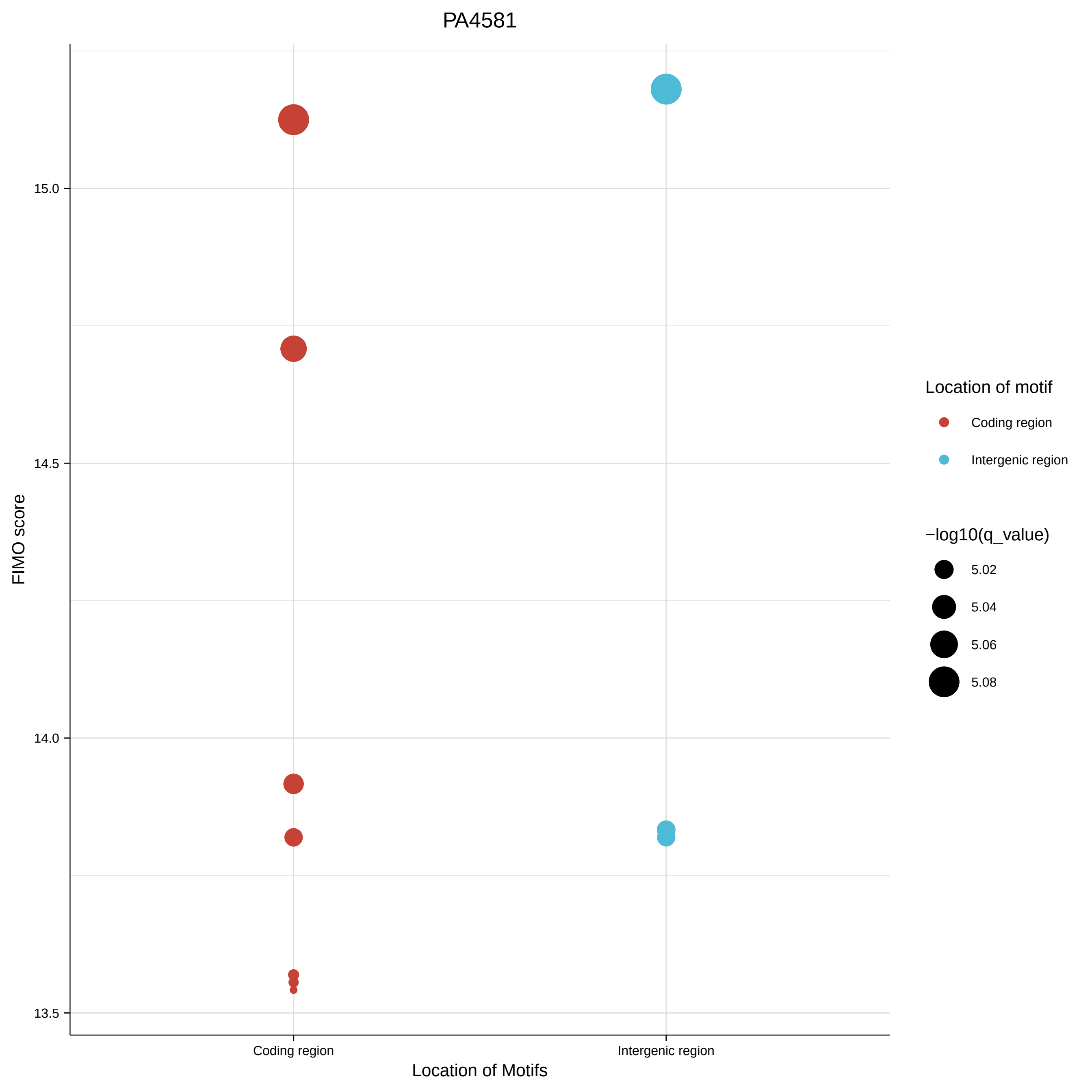

PA4581
FIMO score
15.0
14.5
14.0
13.5
Coding region
Intergenic region
Location of Motifs
Location of motif
Coding region
Intergenic region
−log10(q_value)
5.02
5.04
5.06
5.08

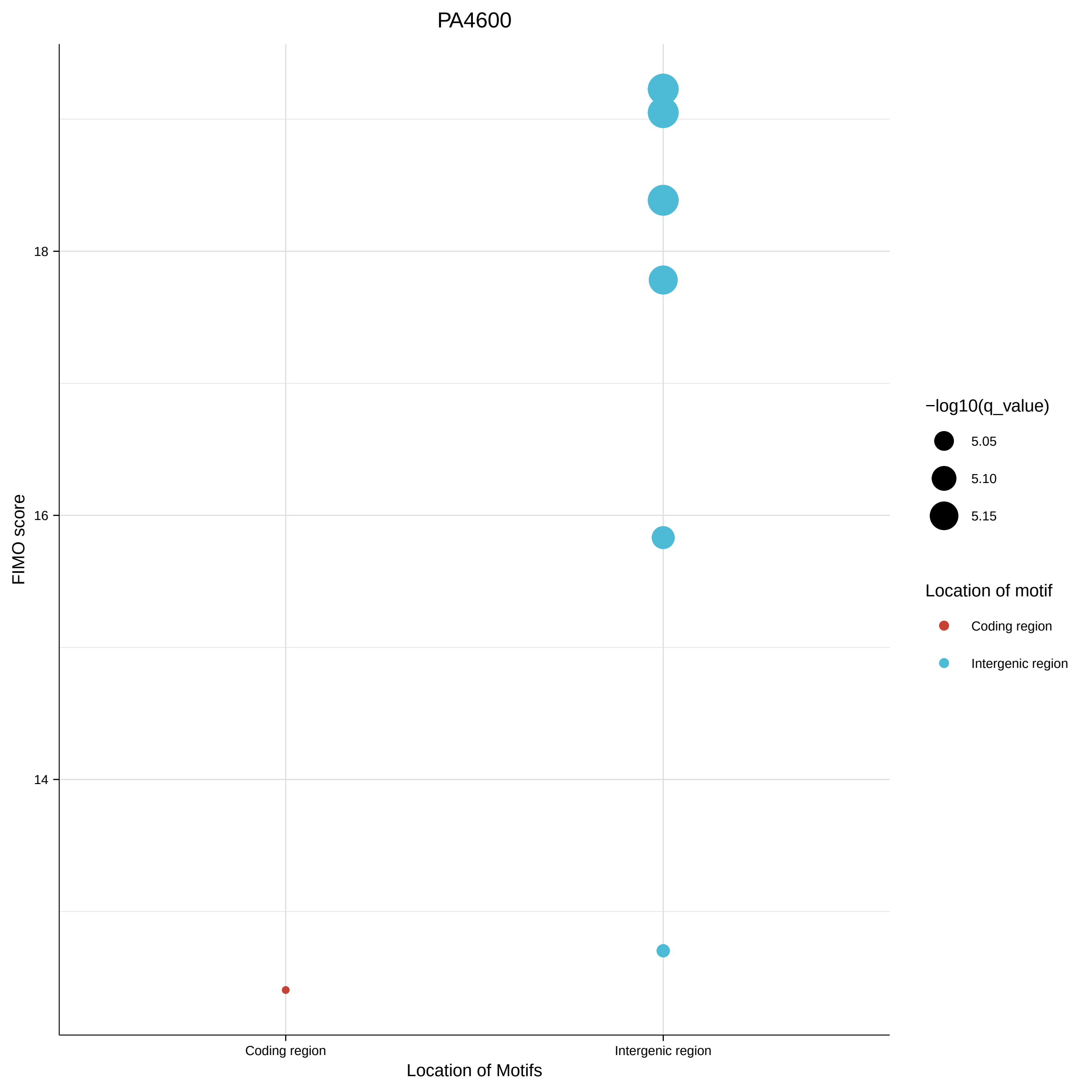
PA4600
FIMO score
18
16
14
Coding region
Intergenic region
Location of Motifs
−log10(q_value)
5.05
5.10
5.15
Location of motif
Coding region
Intergenic region

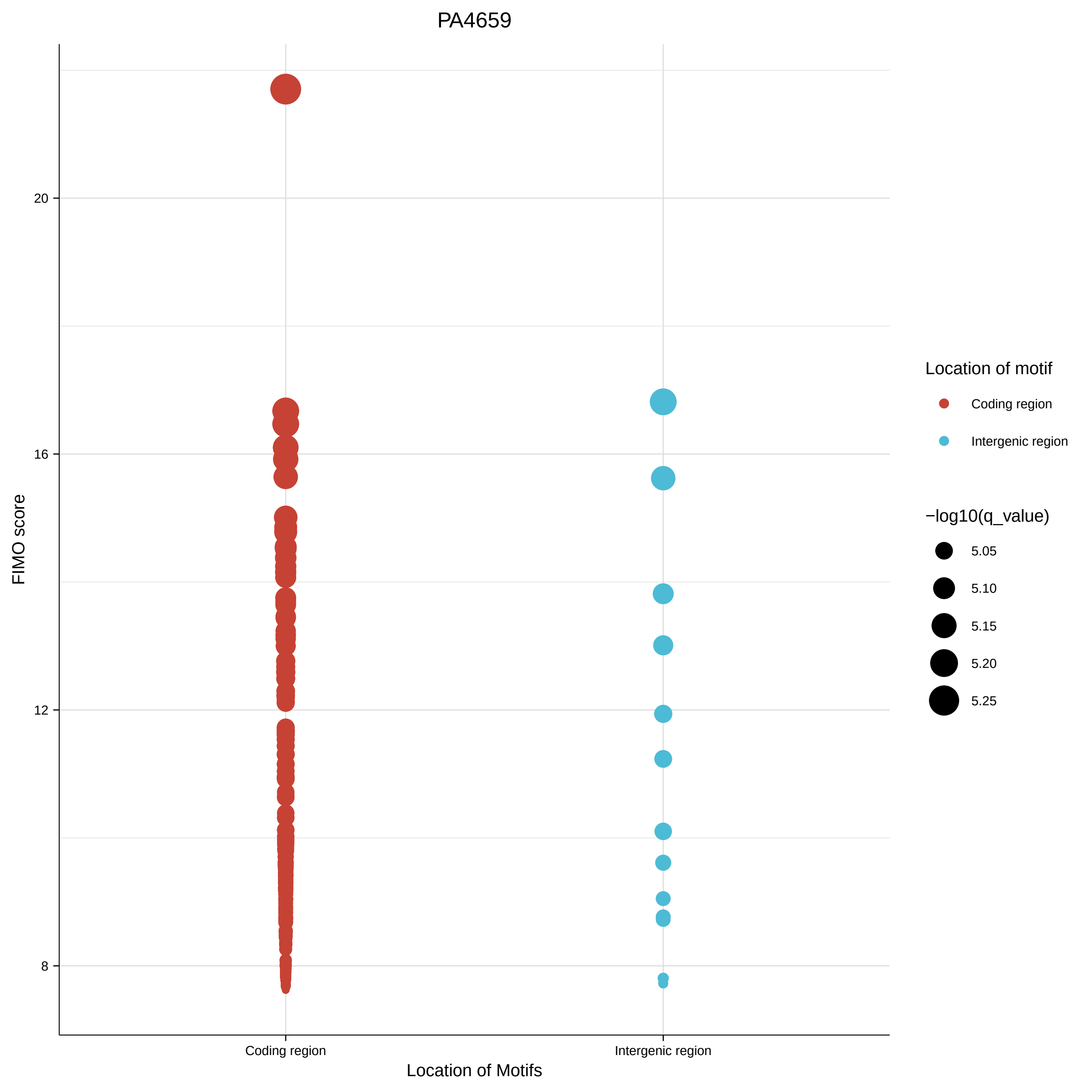

PA4659
FIMO score
20
16
12
8
Coding region
Intergenic region
Location of Motifs
Location of motif
Coding region
Intergenic region
−log10(q_value)
5.05
5.10
5.15
5.20
5.25

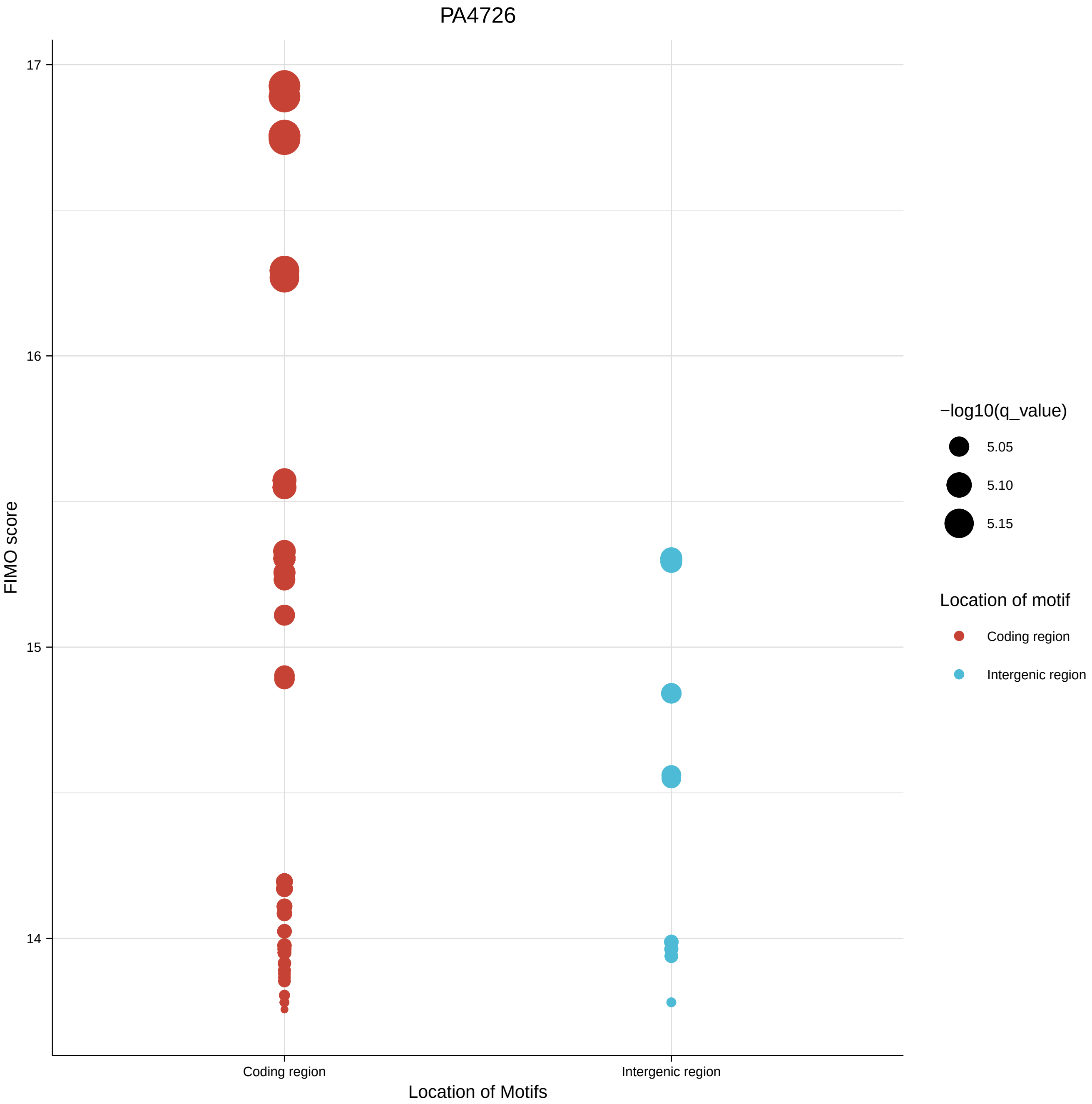
PA4726
FIMO score
17
16
15
14
Coding region
Intergenic region
Location of Motifs
−log10(q_value)
5.05
5.10
5.15
Location of motif
Coding region
Intergenic region

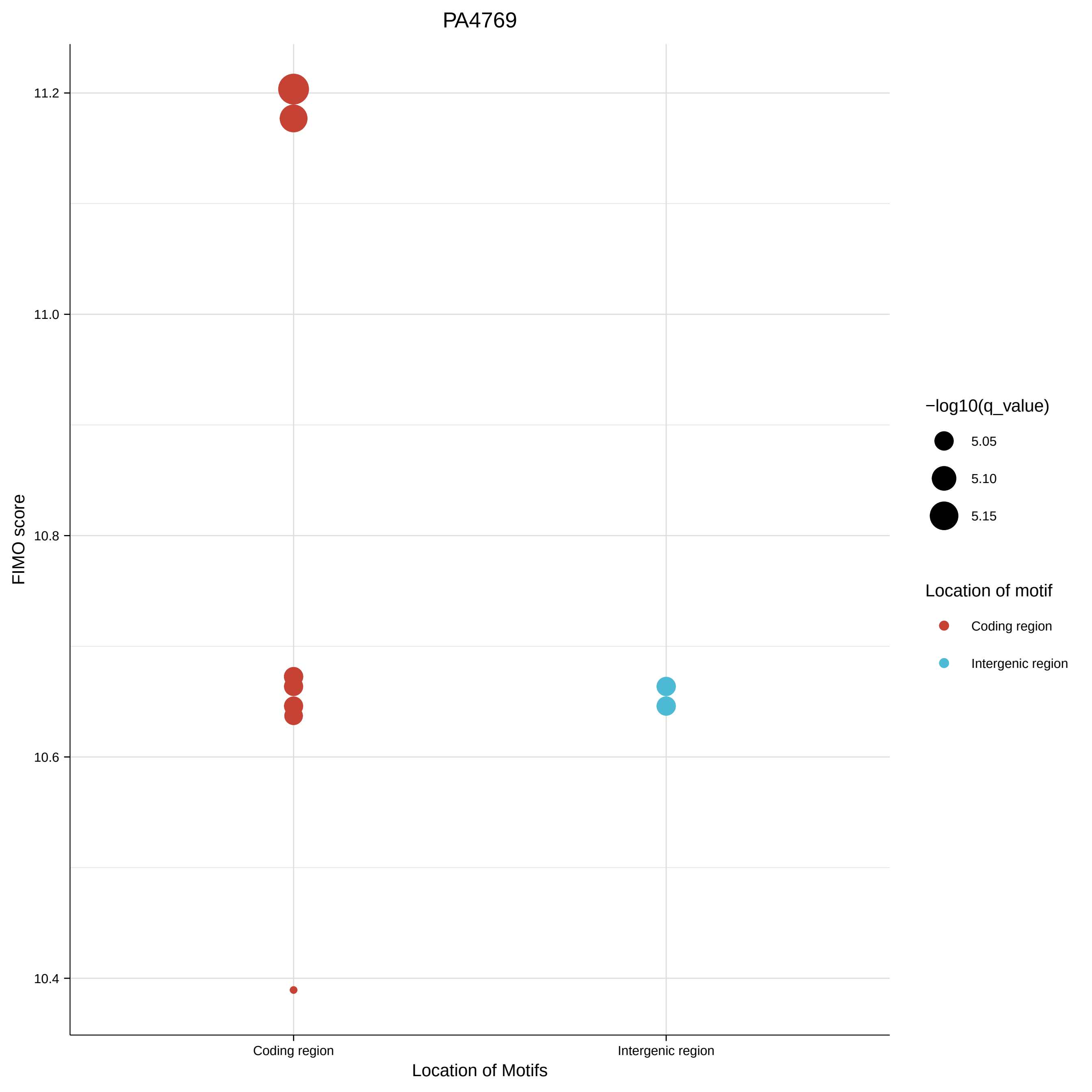
PA4769
FIMO score
11.2
11.0
10.8
10.6
10.4
Coding region
Intergenic region
Location of Motifs
−log10(q_value)
5.05
5.10
5.15
Location of motif
Coding region
Intergenic region

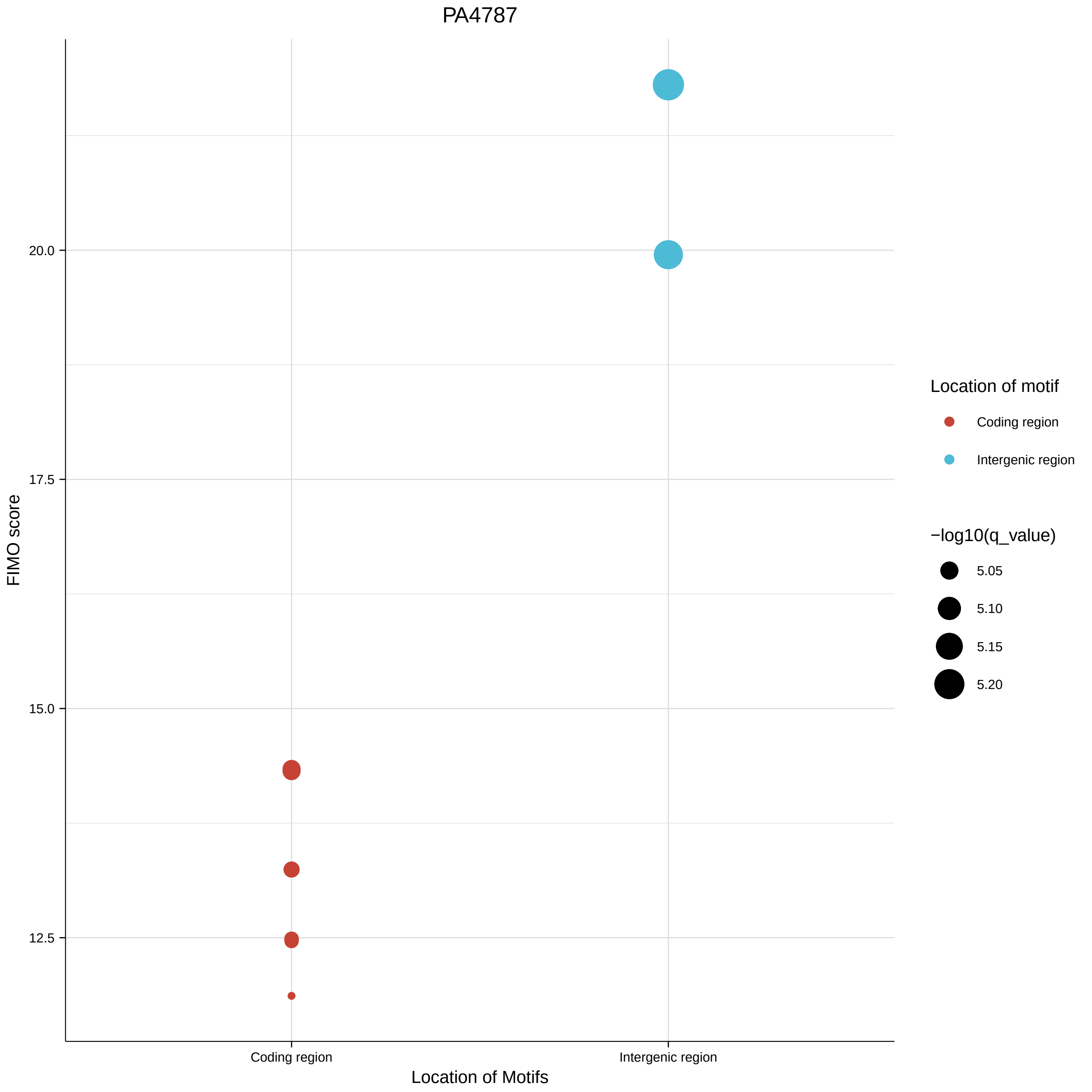

PA4787
FIMO score
20.0
17.5
15.0
12.5
Coding region
Intergenic region
Location of Motifs
Location of motif
Coding region
Intergenic region
−log10(q_value)
5.05
5.10
5.15
5.20

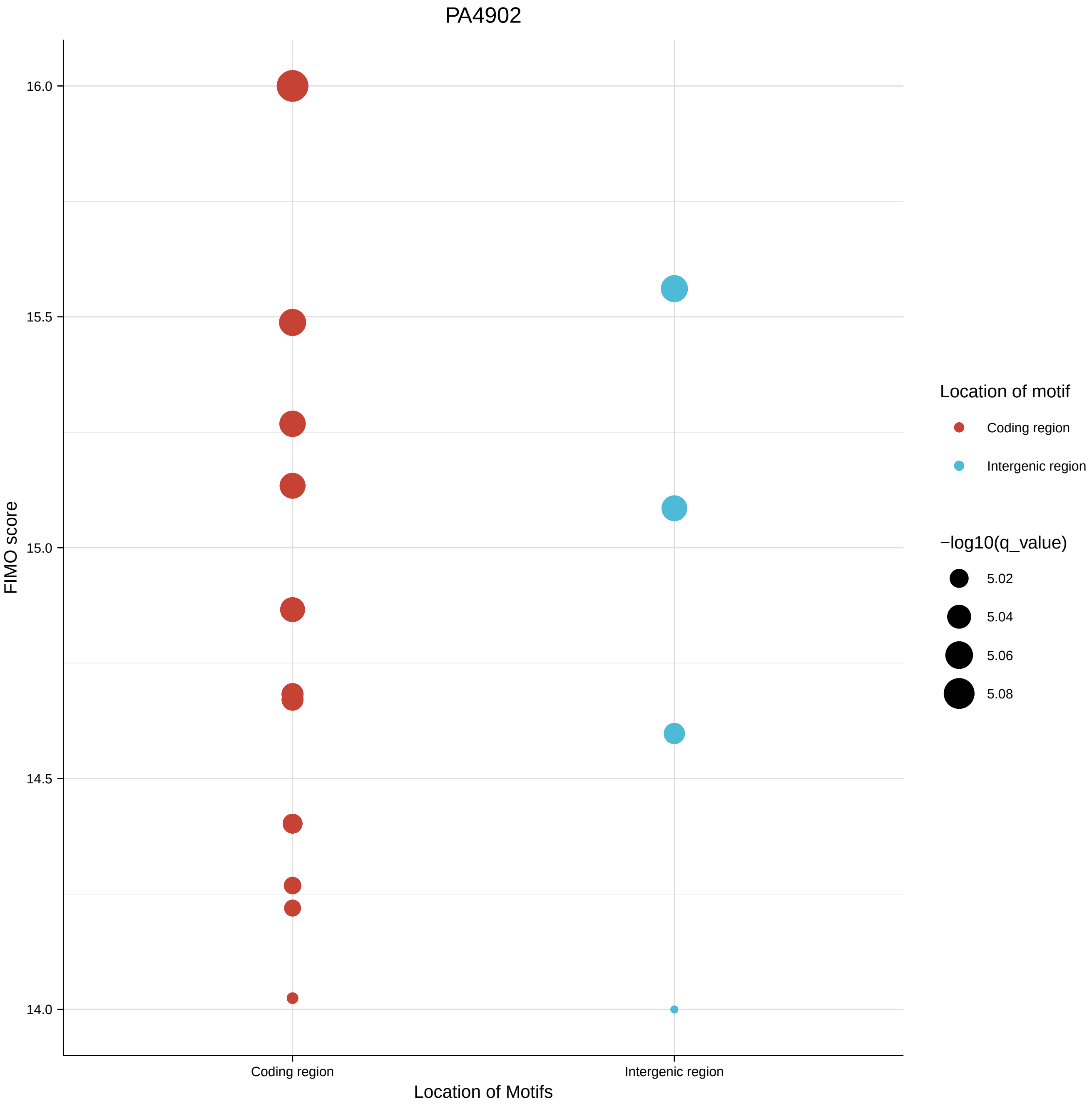

PA4902
FIMO score
16.0
15.5
15.0
14.5
14.0
Coding region
Intergenic region
Location of Motifs
Location of motif
Coding region
Intergenic region
−log10(q_value)
5.02
5.04
5.06
5.08

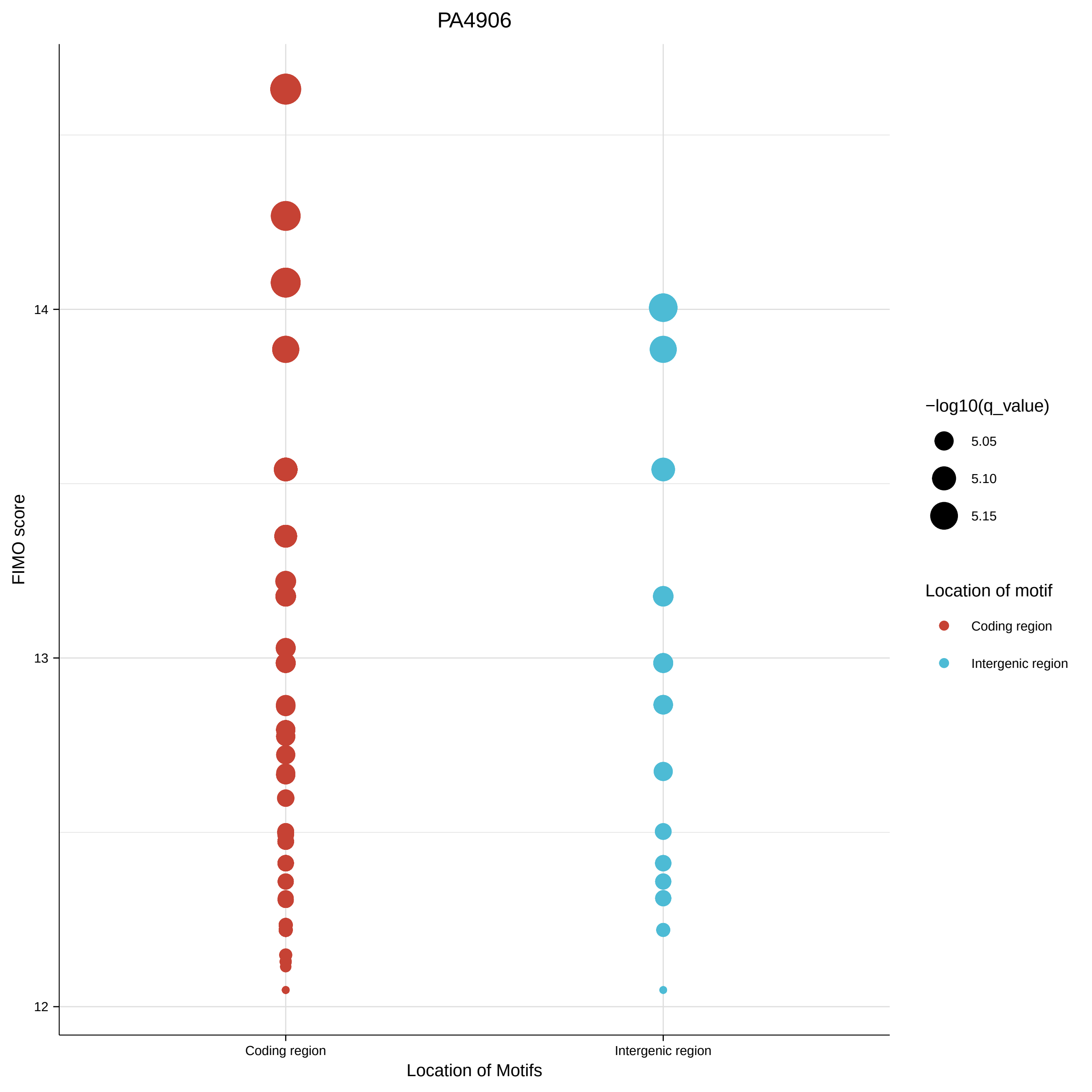
PA4906
FIMO score
14
13
12
Coding region
Intergenic region
Location of Motifs
−log10(q_value)
5.05
5.10
5.15
Location of motif
Coding region
Intergenic region

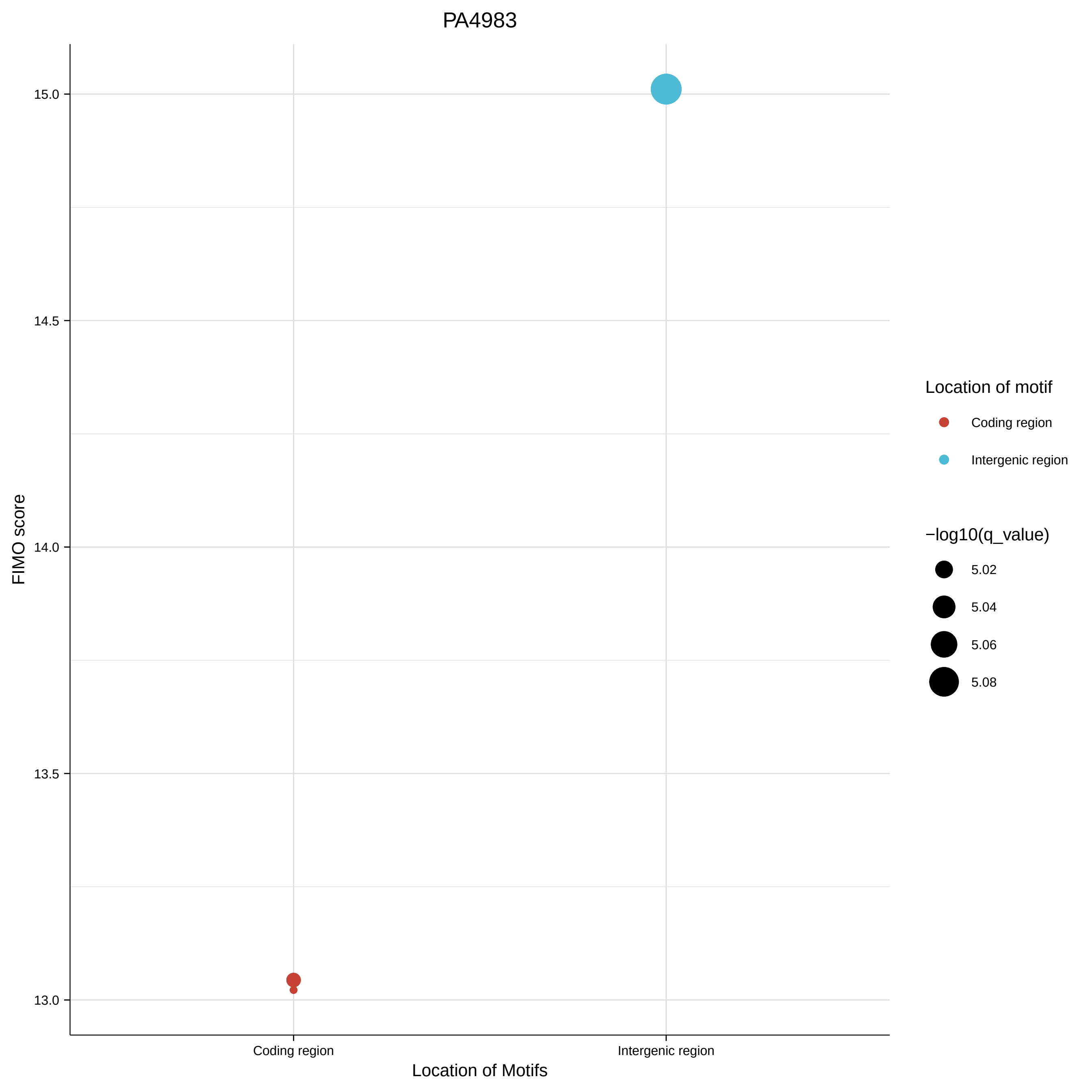
PA4983
FIMO score
15.0
14.5
14.0
13.5
13.0
Coding region
Intergenic region
Location of Motifs
Location of motif
Coding region
Intergenic region
−log10(q_value)
5.02
5.04
5.06
5.08

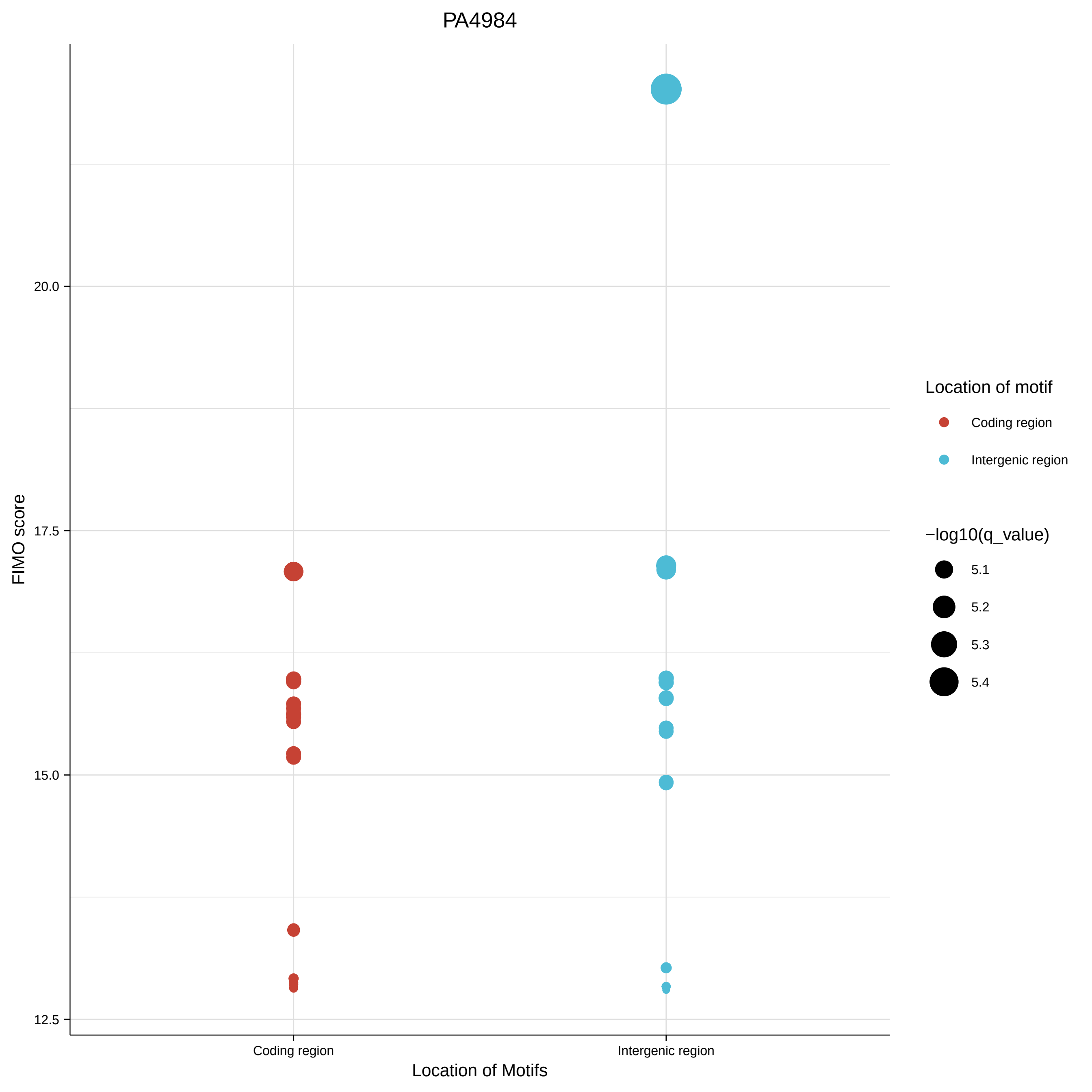
PA4984
FIMO score
20.0
17.5
15.0
12.5
Coding region
Intergenic region
Location of Motifs
Location of motif
Coding region
Intergenic region
−log10(q_value)
5.1
5.2
5.3
5.4

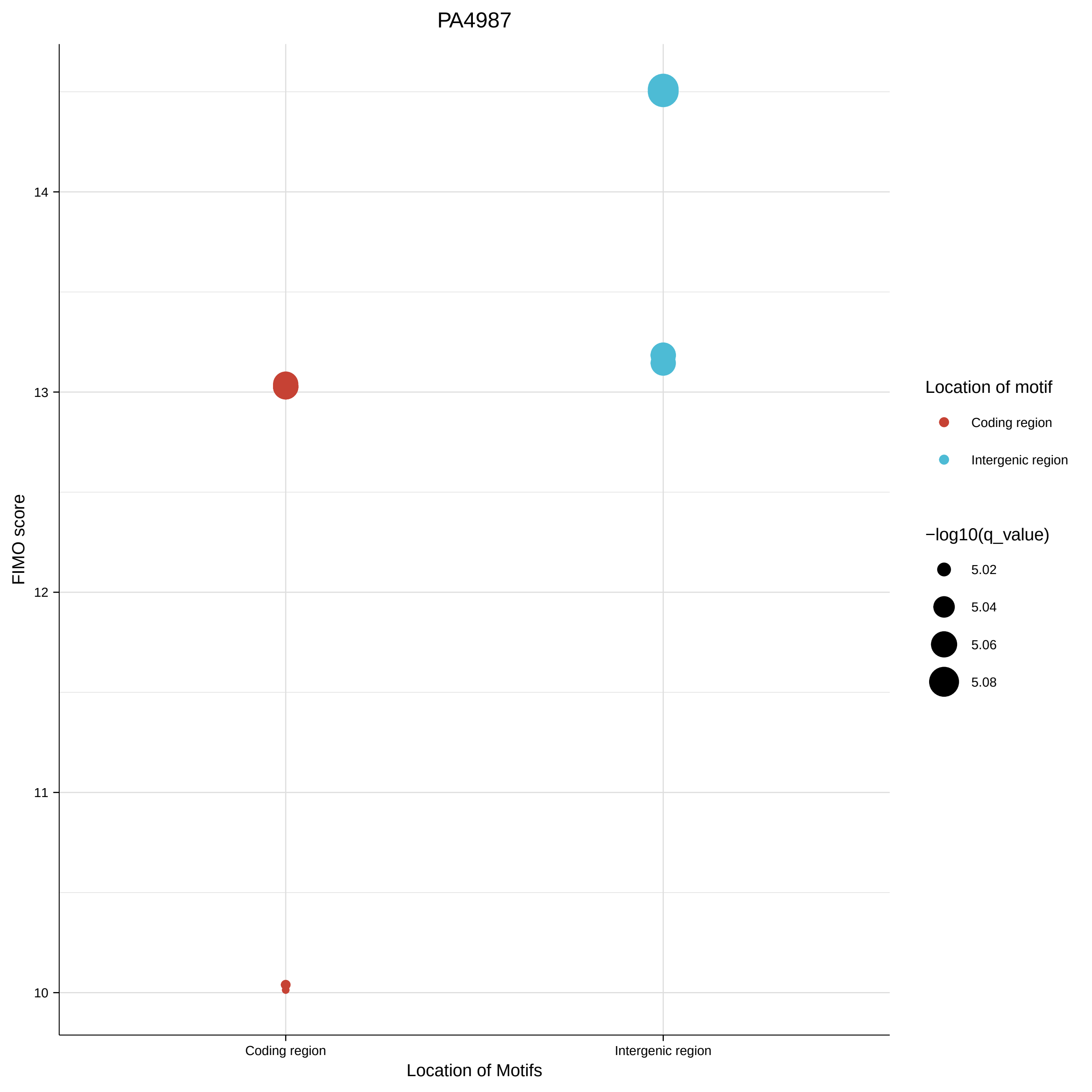
PA4987
FIMO score
14
13
12
11
10
Coding region
Intergenic region
Location of Motifs
Location of motif
Coding region
Intergenic region
−log10(q_value)
5.02
5.04
5.06
5.08

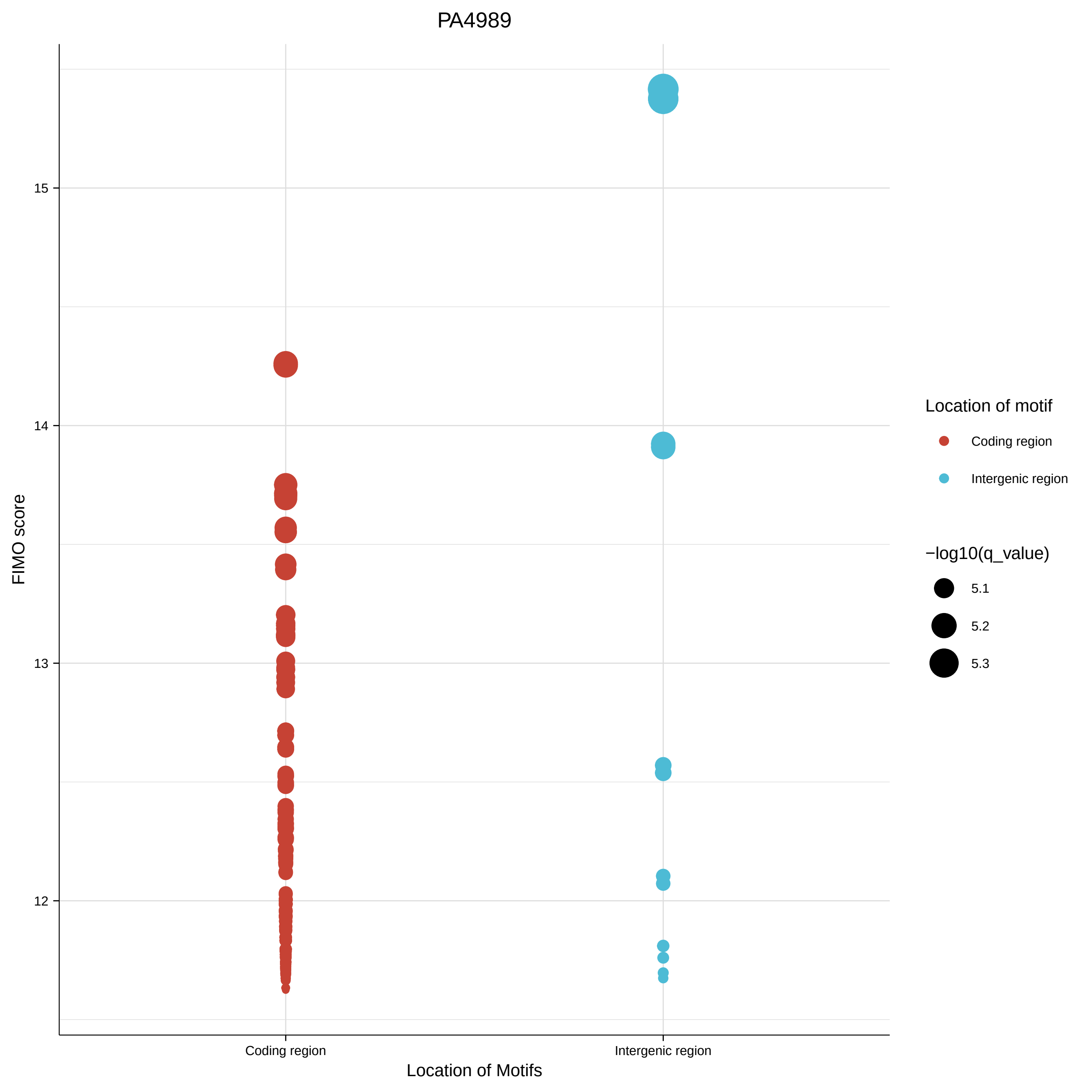

PA4989
FIMO score
15
14
13
12
Coding region
Intergenic region
Location of Motifs
Location of motif
Coding region
Intergenic region
−log10(q_value)
5.1
5.2
5.3

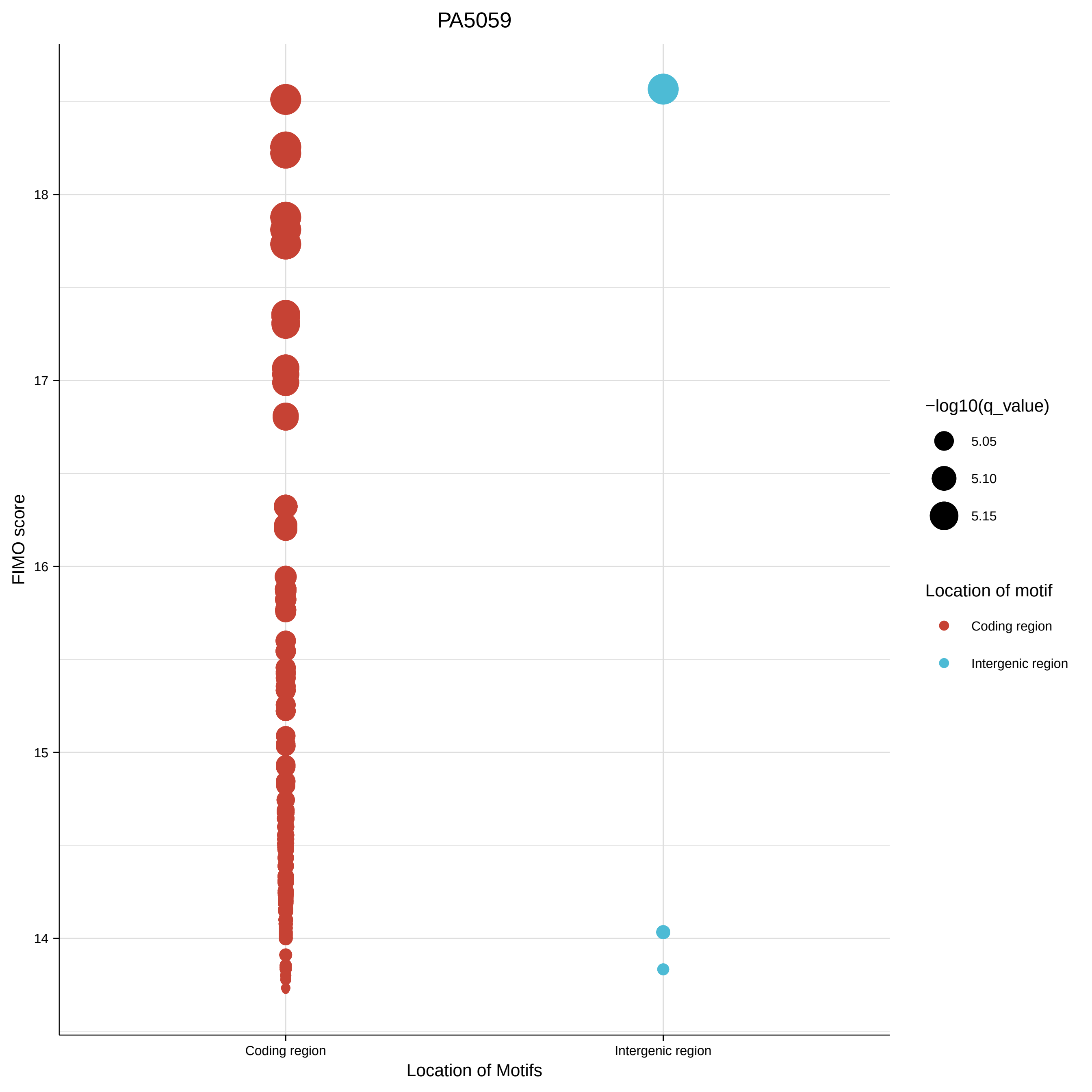
PA5059
FIMO score
18
17
16
15
14
Coding region
Intergenic region
Location of Motifs
−log10(q_value)
5.05
5.10
5.15
Location of motif
Coding region
Intergenic region

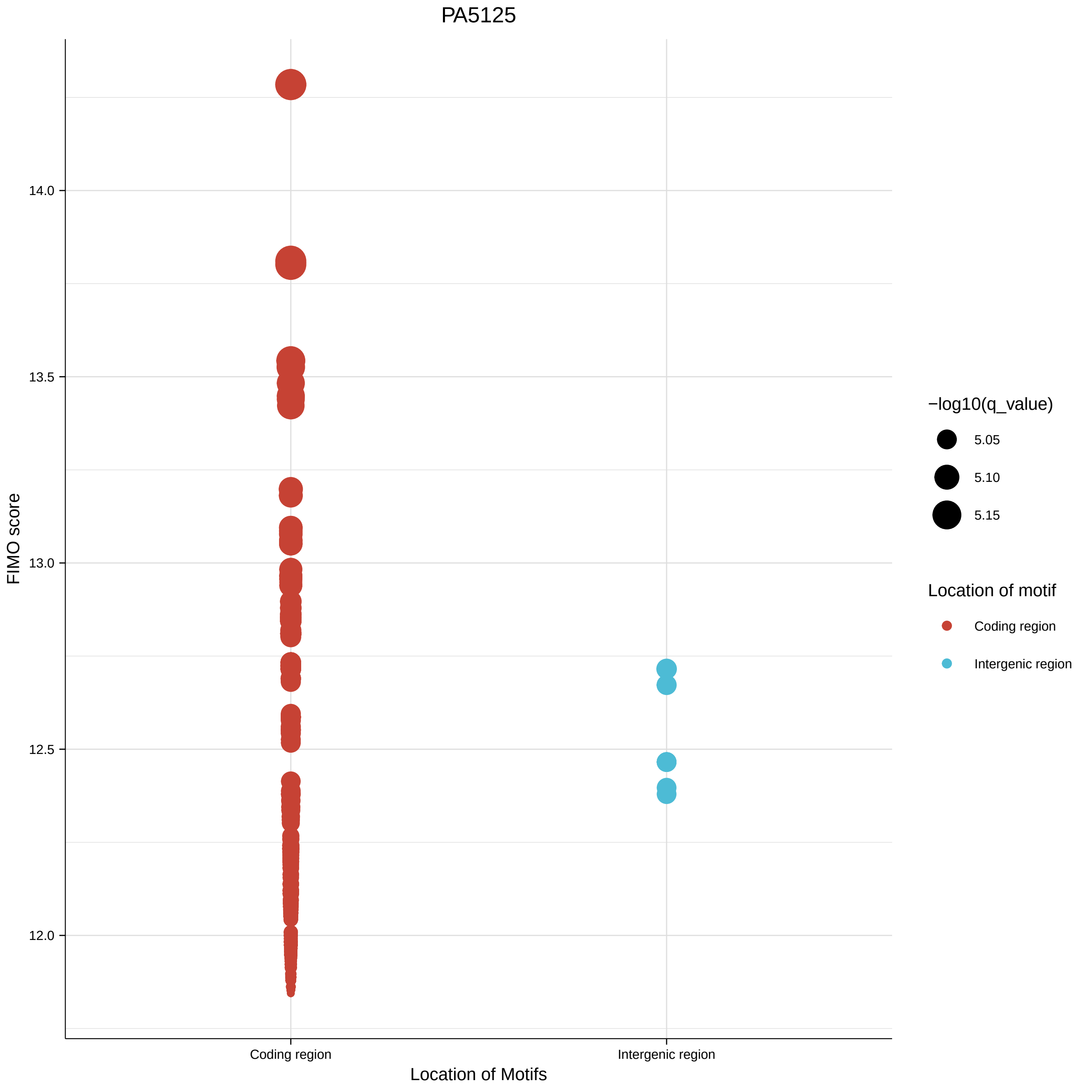
PA5125
FIMO score
14.0
13.5
13.0
12.5
12.0
Coding region
Intergenic region
Location of Motifs
−log10(q_value)
5.05
5.10
5.15
Location of motif
Coding region
Intergenic region

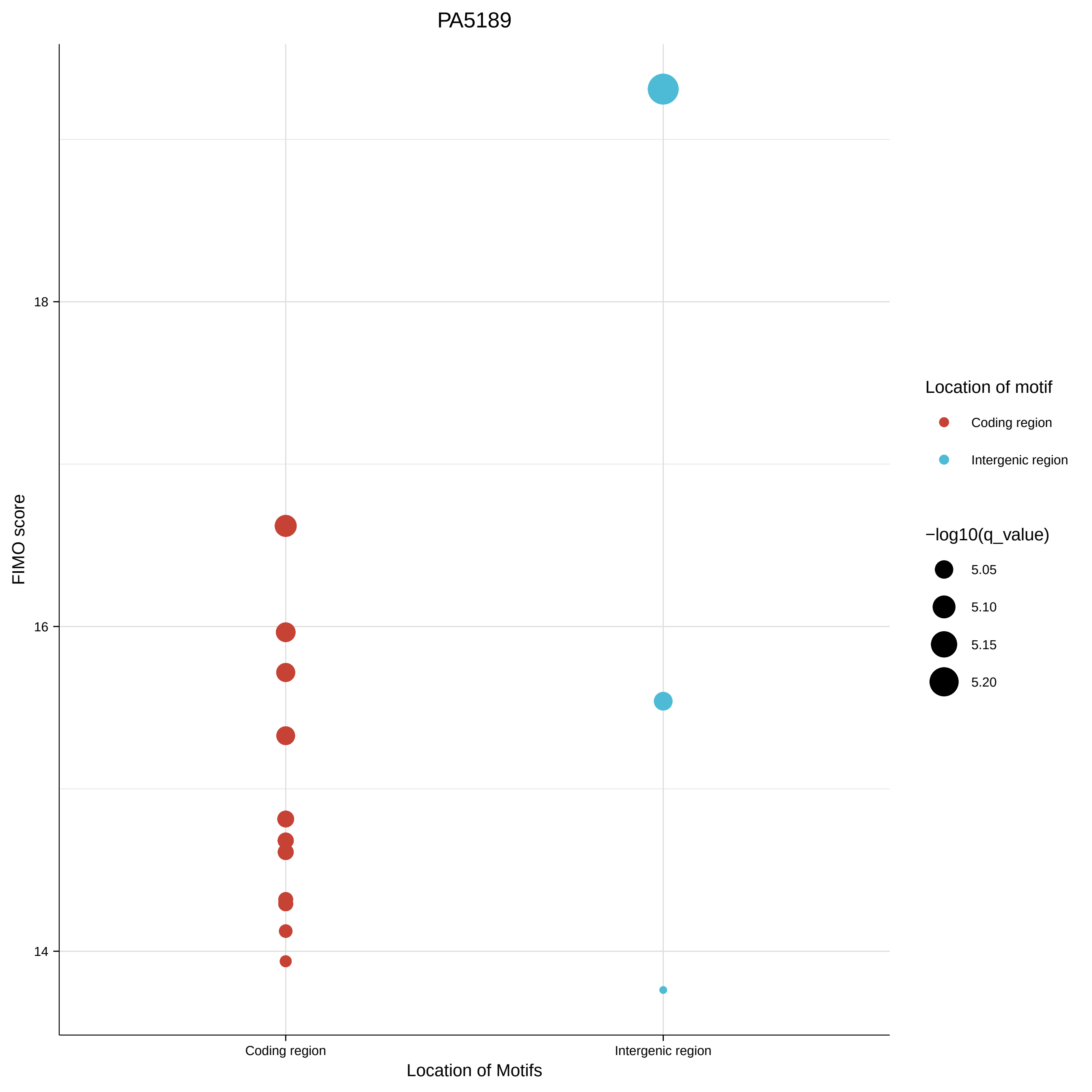
PA5189
FIMO score
18
16
14
Coding region
Intergenic region
Location of Motifs
Location of motif
Coding region
Intergenic region
−log10(q_value)
5.05
5.10
5.15
5.20

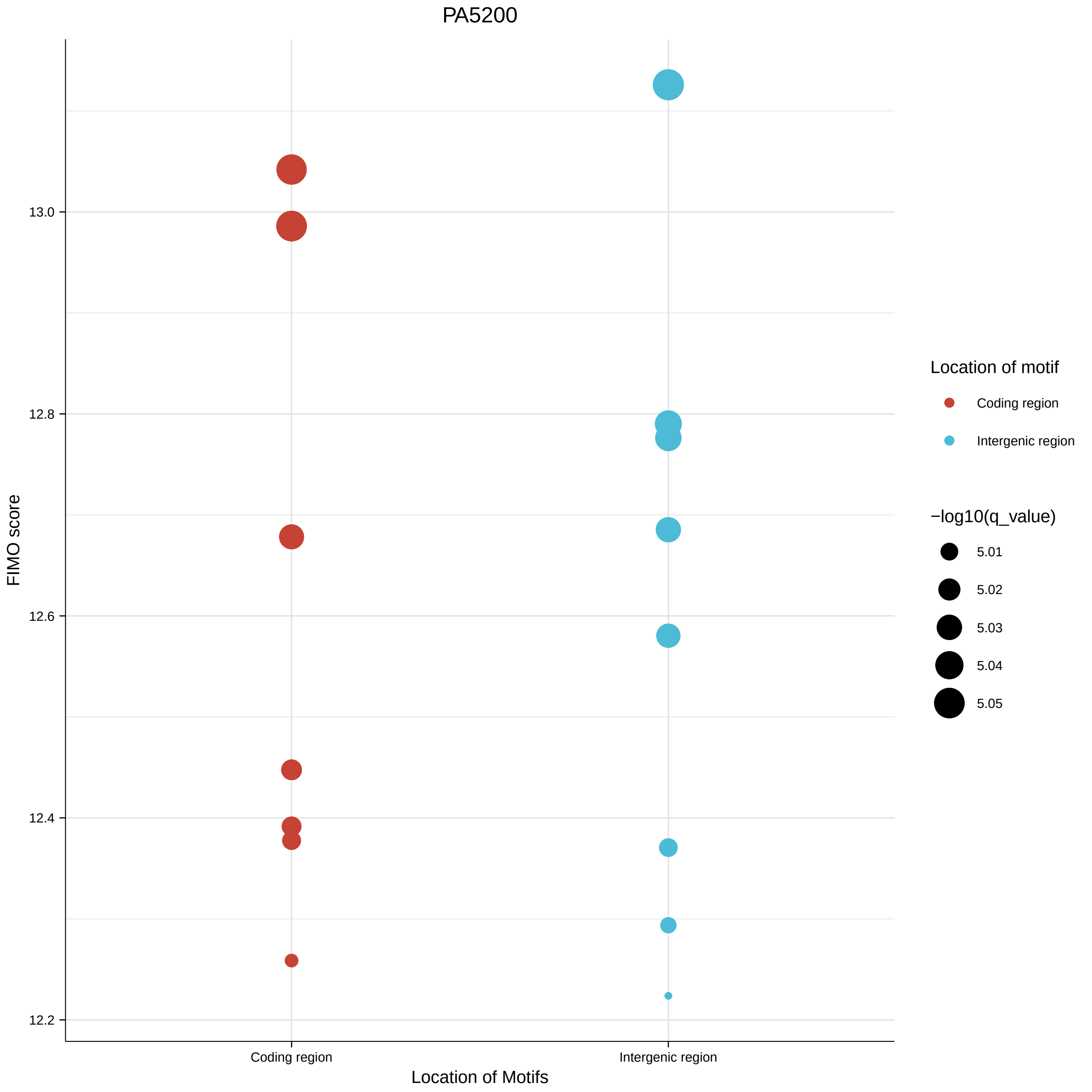
PA5200
FIMO score
13.0
12.8
12.6
12.4
12.2
Coding region
Intergenic region
Location of Motifs
Location of motif
Coding region
Intergenic region
−log10(q_value)
5.01
5.02
5.03
5.04
5.05

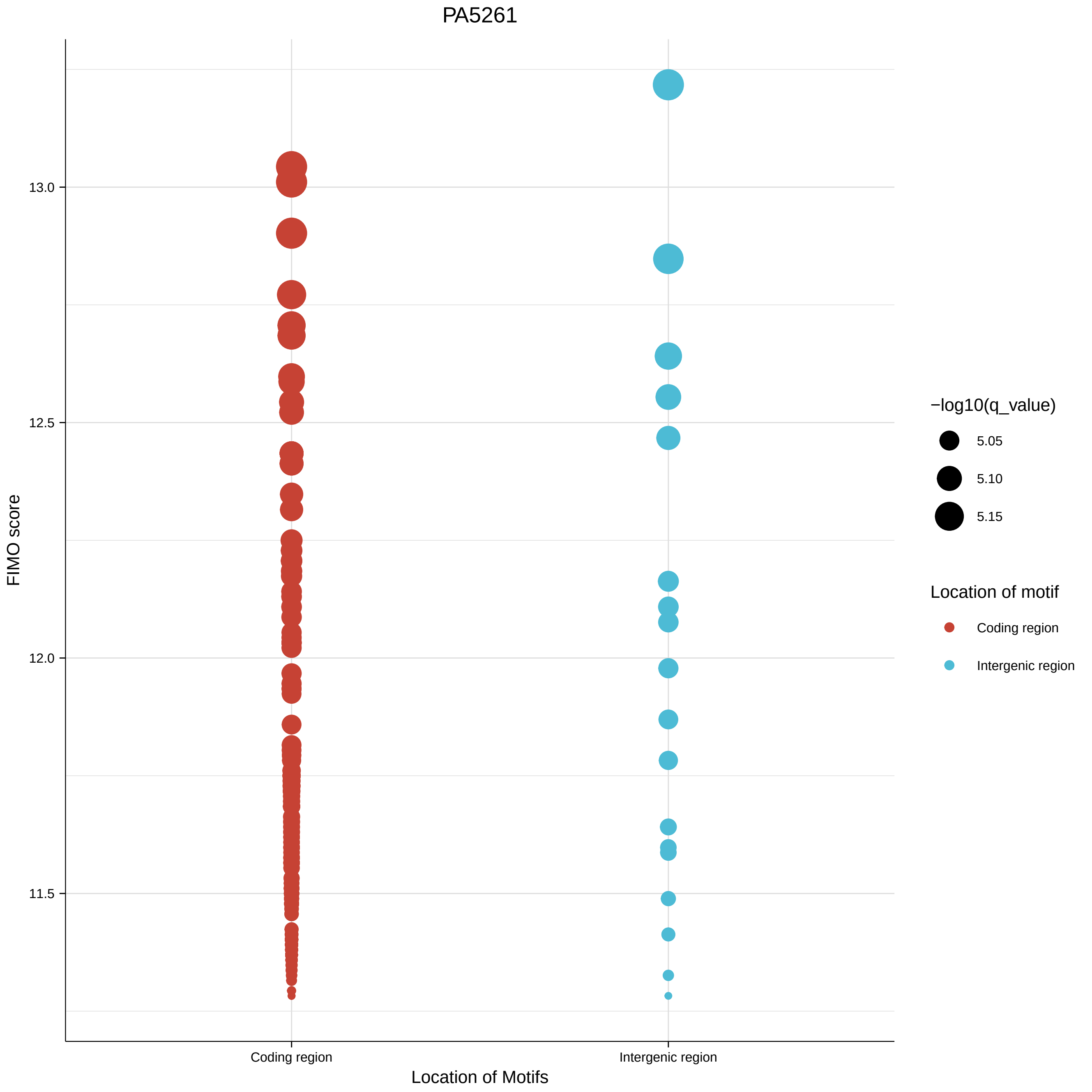
PA5261
FIMO score
13.0
12.5
12.0
11.5
Coding region
Intergenic region
Location of Motifs
−log10(q_value)
5.05
5.10
5.15
Location of motif
Coding region
Intergenic region

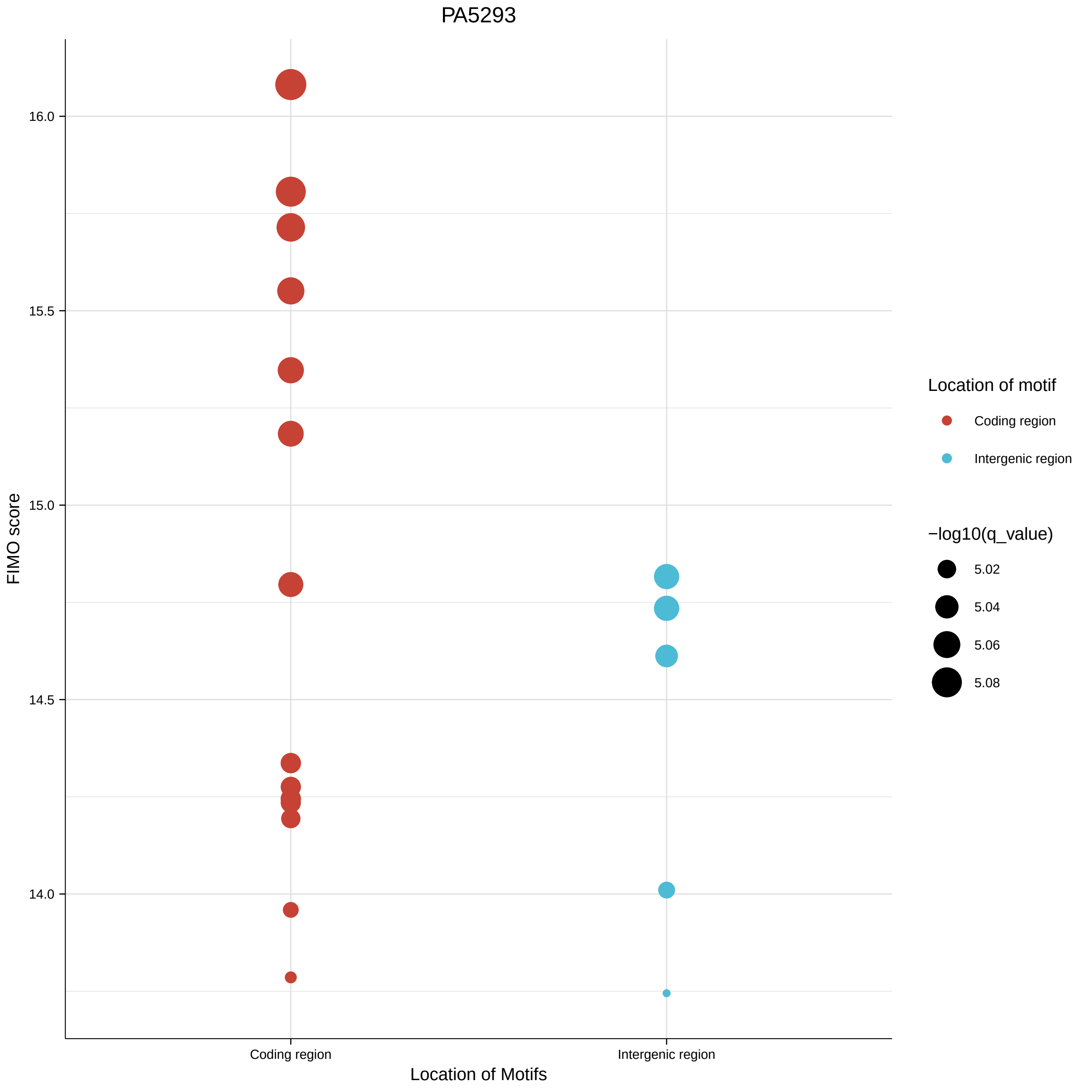
PA5293
FIMO score
16.0
15.5
15.0
14.5
14.0
Coding region
Intergenic region
Location of Motifs
Location of motif
Coding region
Intergenic region
−log10(q_value)
5.02
5.04
5.06
5.08

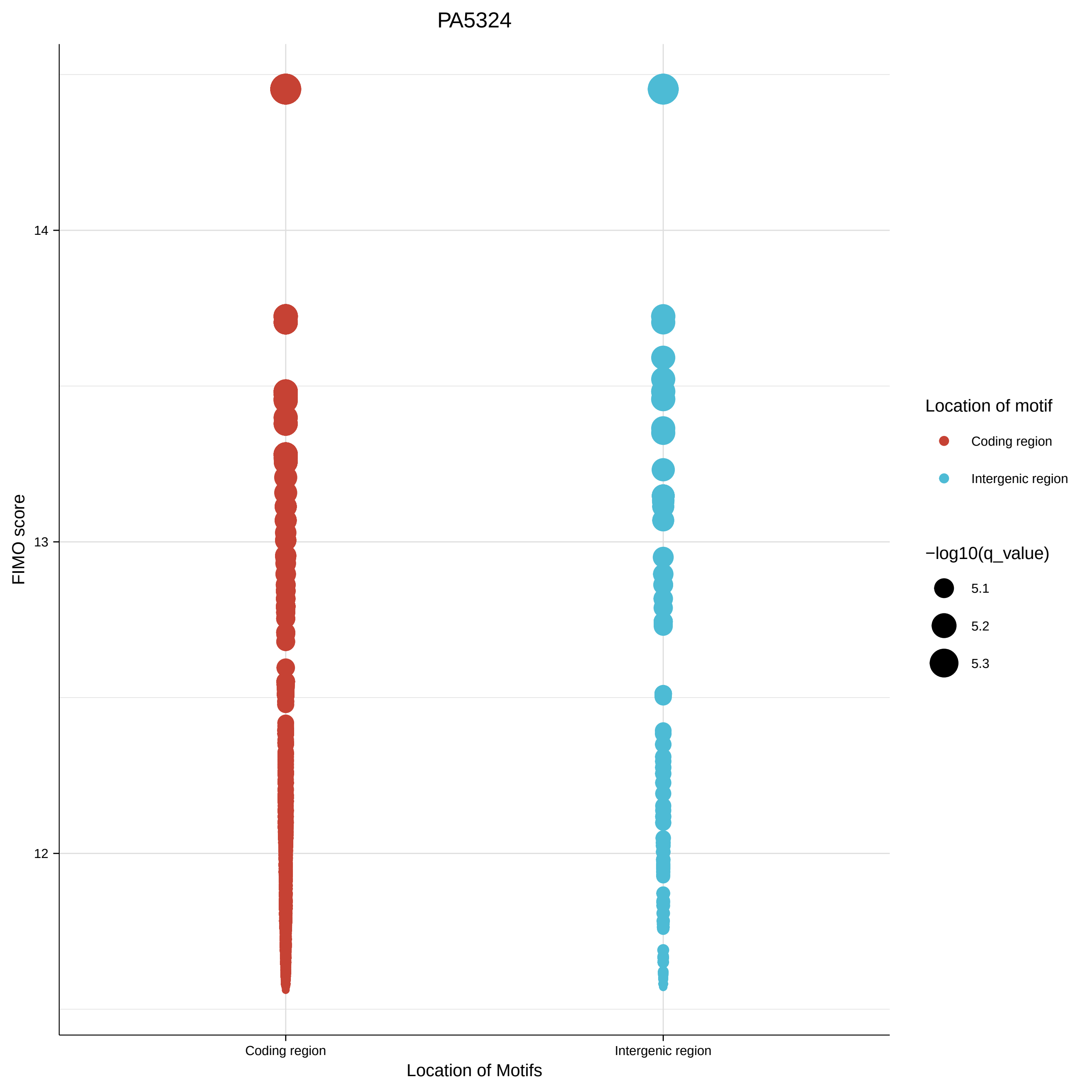
PA5324
FIMO score
14
13
12
Coding region
Intergenic region
Location of Motifs
Location of motif
Coding region
Intergenic region
−log10(q_value)
5.1
5.2
5.3

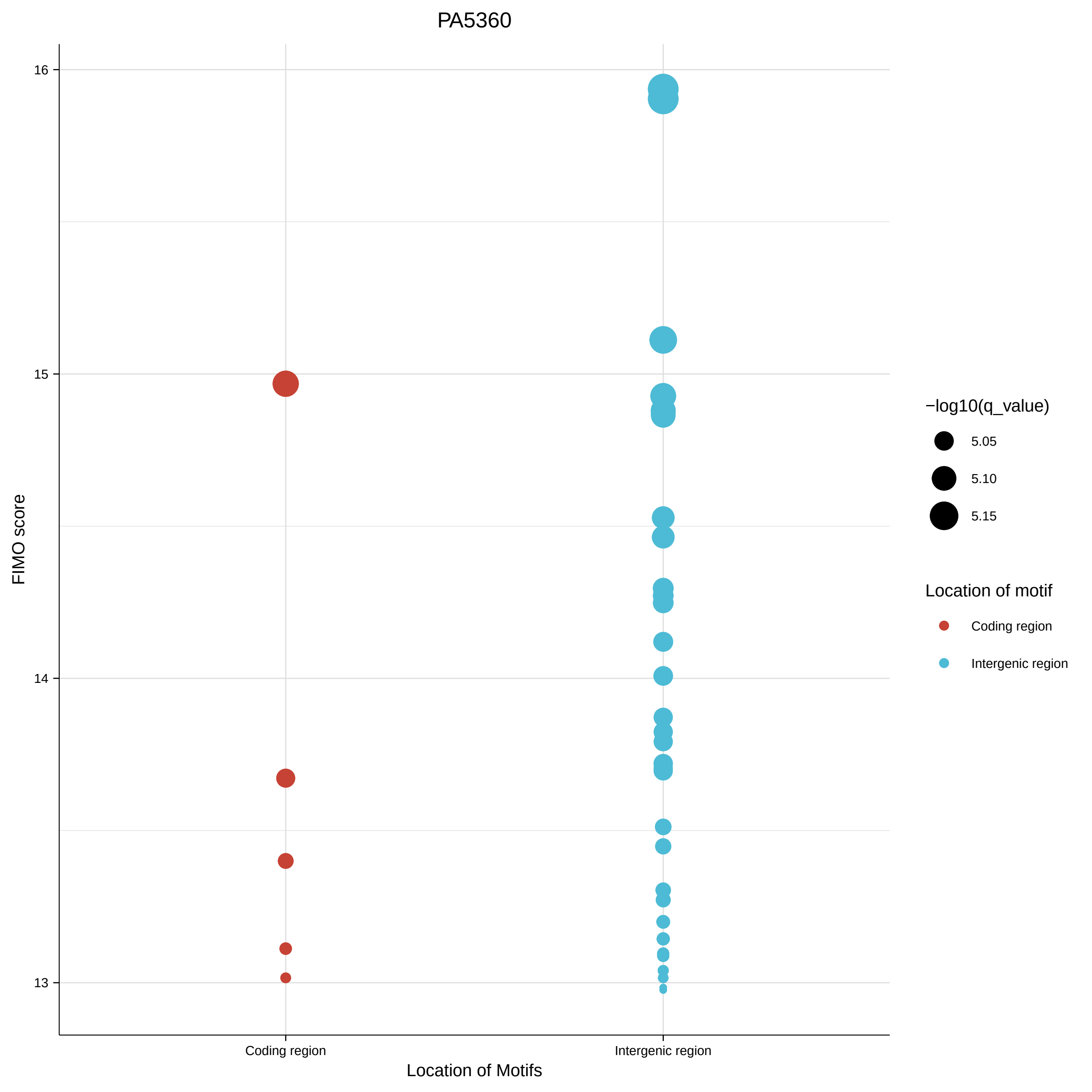

PA5360
FIMO score
16
15
14
13
Coding region
Intergenic region
Location of Motifs
−log10(q_value)
5.05
5.10
5.15
Location of motif
Coding region
Intergenic region

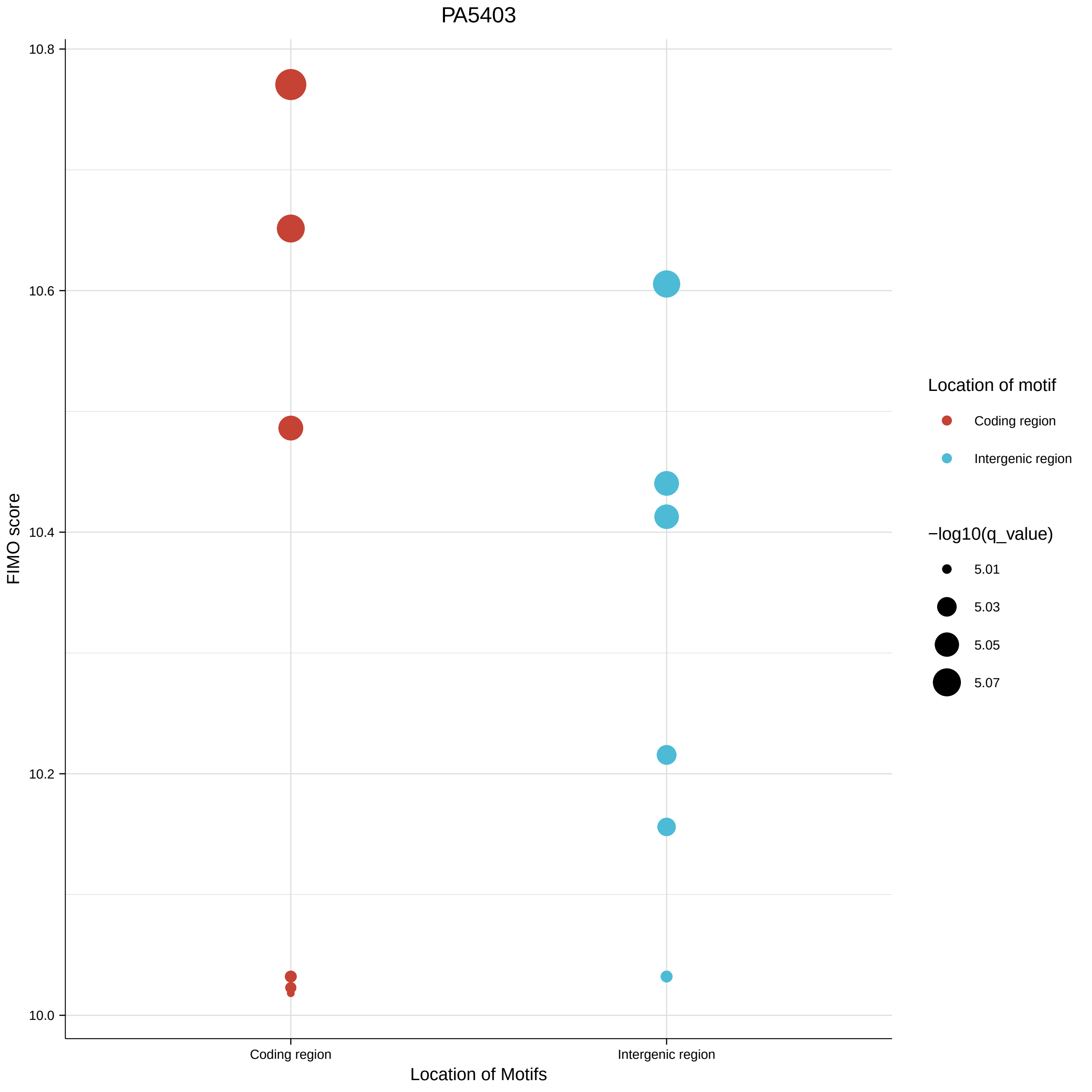
PA5403
FIMO score
10.8
10.6
10.4
10.2
10.0
Coding region
Intergenic region
Location of Motifs
Location of motif
Coding region
Intergenic region
−log10(q_value)
5.01
5.03
5.05
5.07

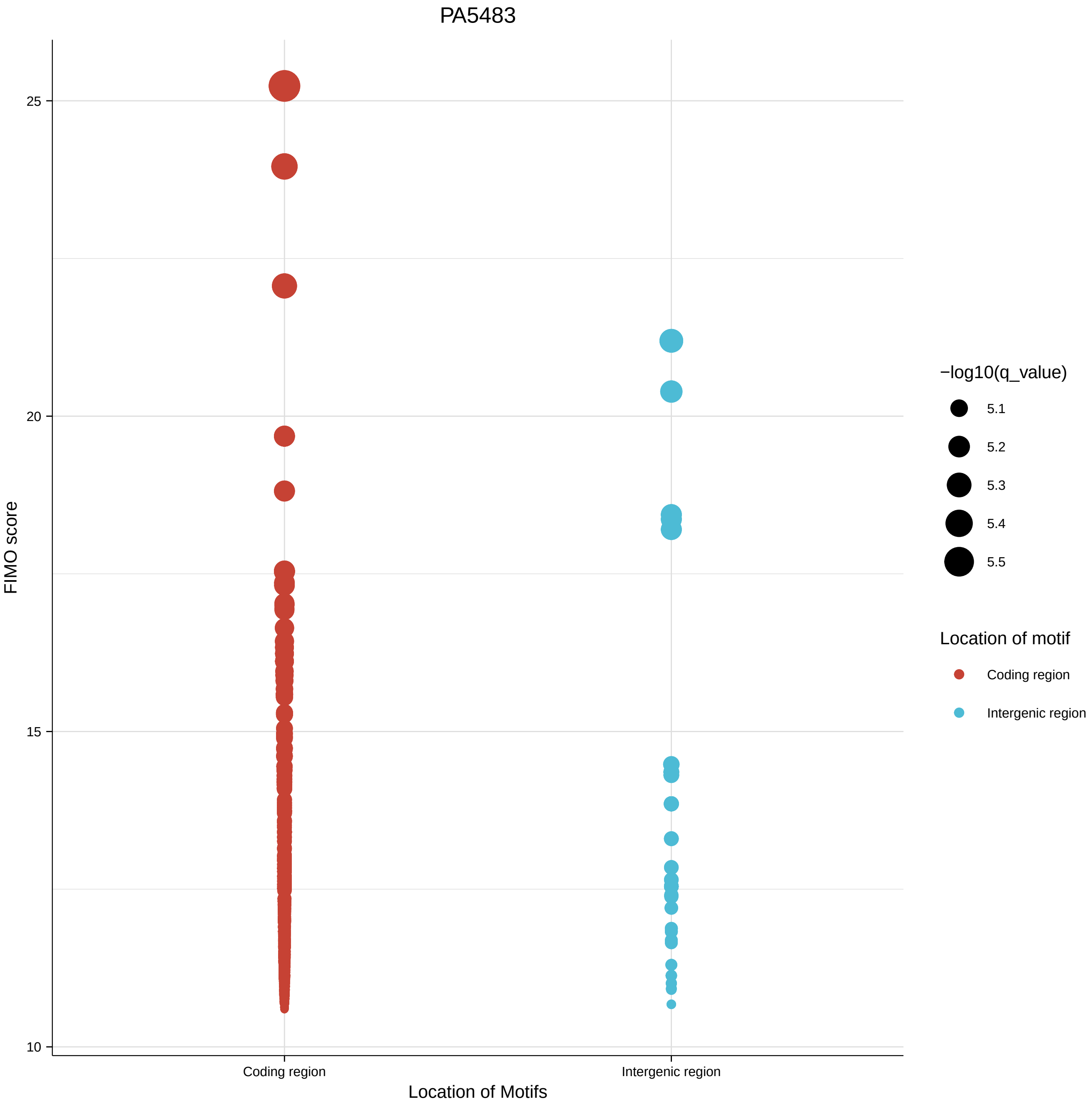
PA5483
FIMO score
25
20
15
10
Coding region
Intergenic region
Location of Motifs
−log10(q_value)
5.1
5.2
5.3
5.4
5.5
Location of motif
Coding region
Intergenic region

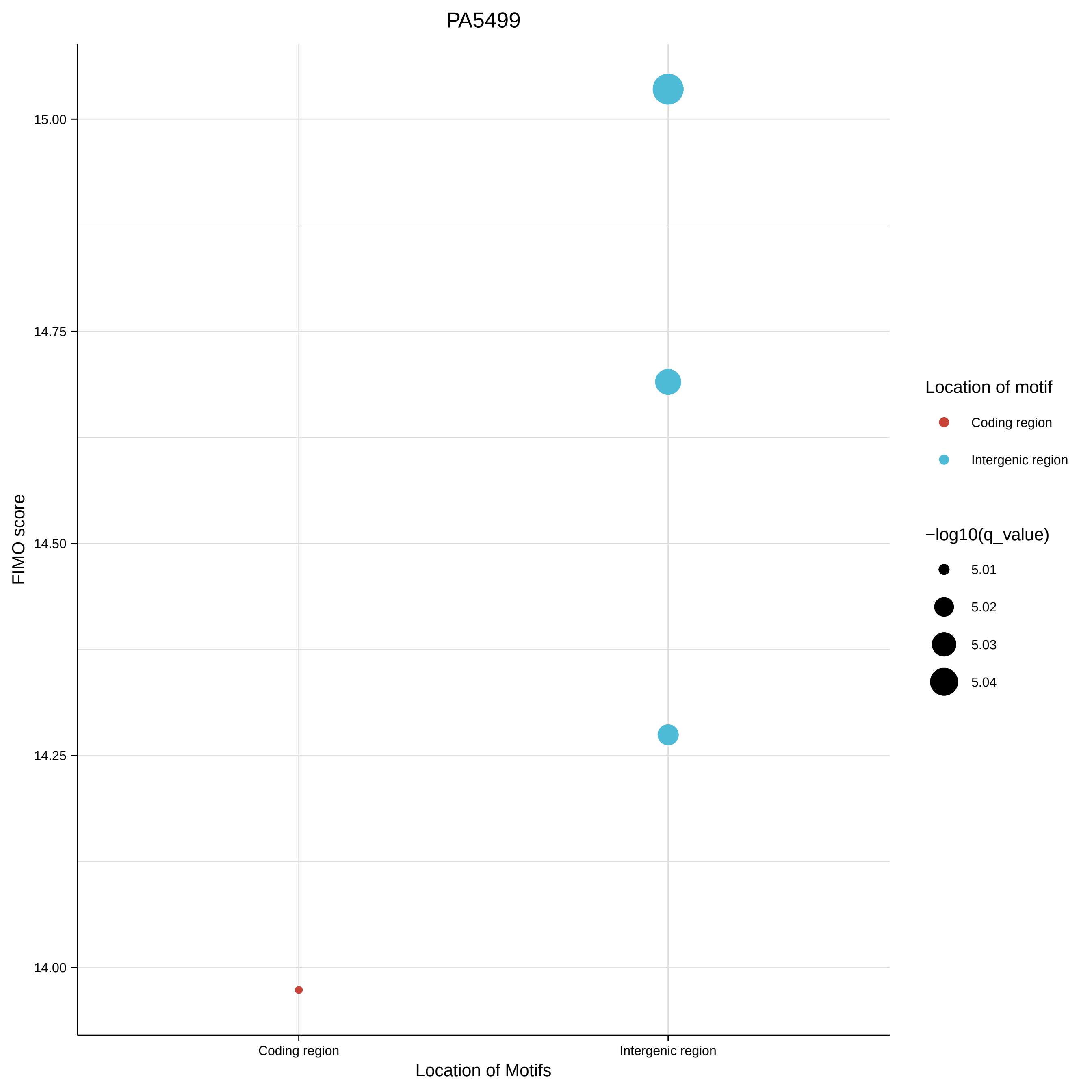
PA5499
FIMO score
15.00
14.75
14.50
14.25
14.00
Coding region
Intergenic region
Location of Motifs
Location of motif
Coding region
Intergenic region
−log10(q_value)
5.01
5.02
5.03
5.04

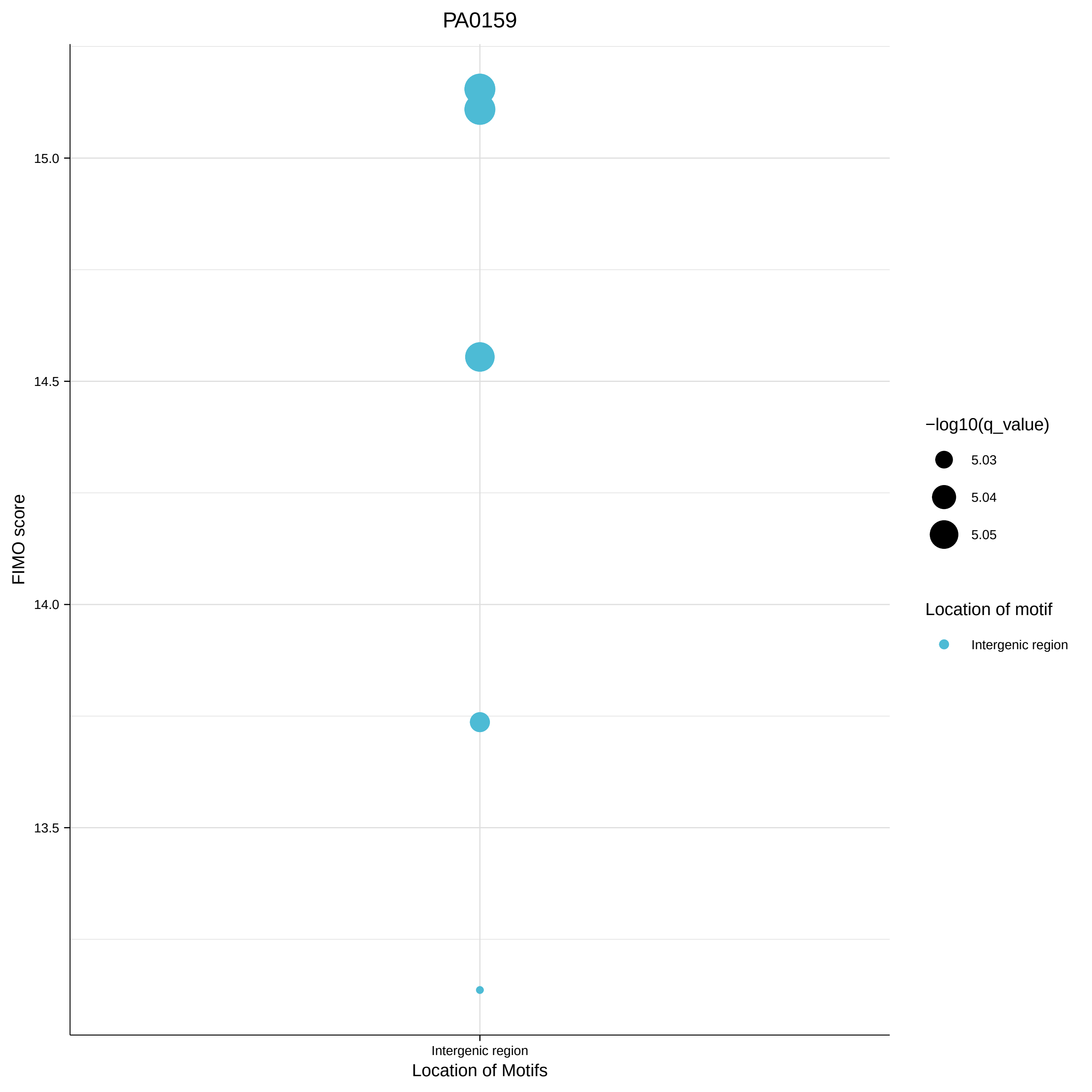
PA0159
FIMO score
15.0
14.5
14.0
13.5
Intergenic region
Location of Motifs
−log10(q_value)
5.03
5.04
5.05
Location of motif
Intergenic region

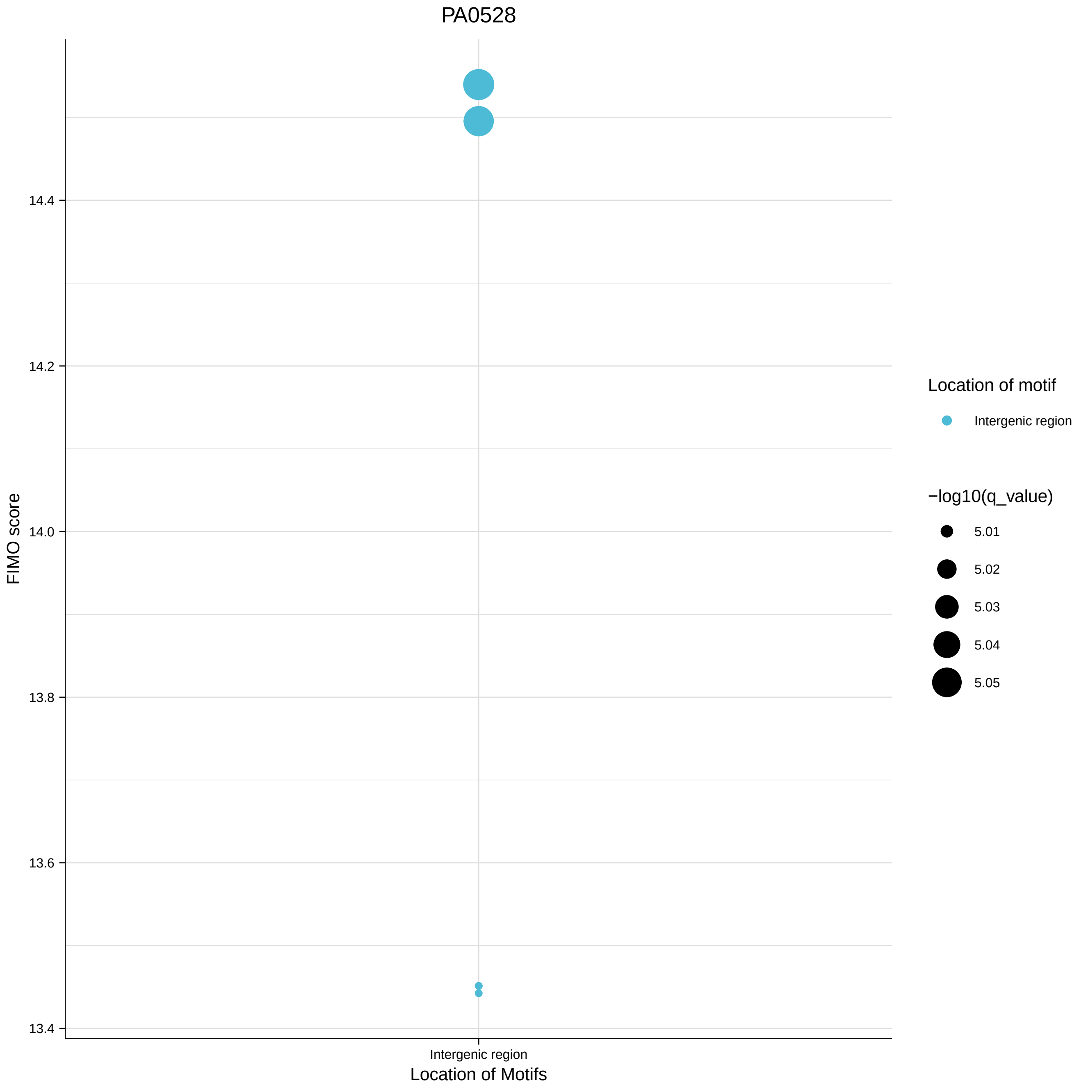
PA0528
FIMO score
14.4
14.2
14.0
13.8
13.6
13.4
Intergenic region
Location of Motifs
Location of motif
Intergenic region
−log10(q_value)
5.01
5.02
5.03
5.04
5.05

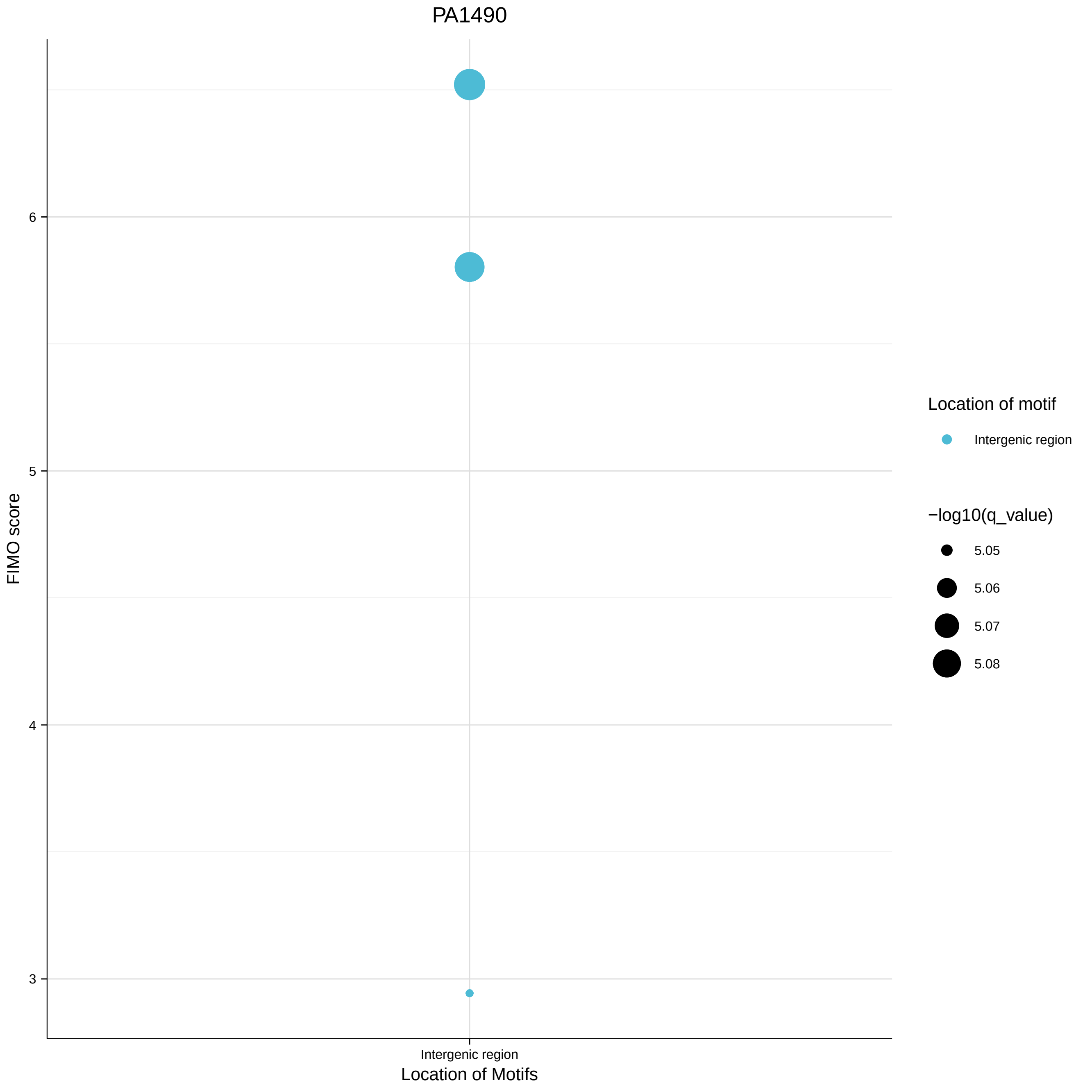
PA1490
FIMO score
6
5
4
3
Intergenic region
Location of Motifs
Location of motif
Intergenic region
−log10(q_value)
5.05
5.06
5.07
5.08

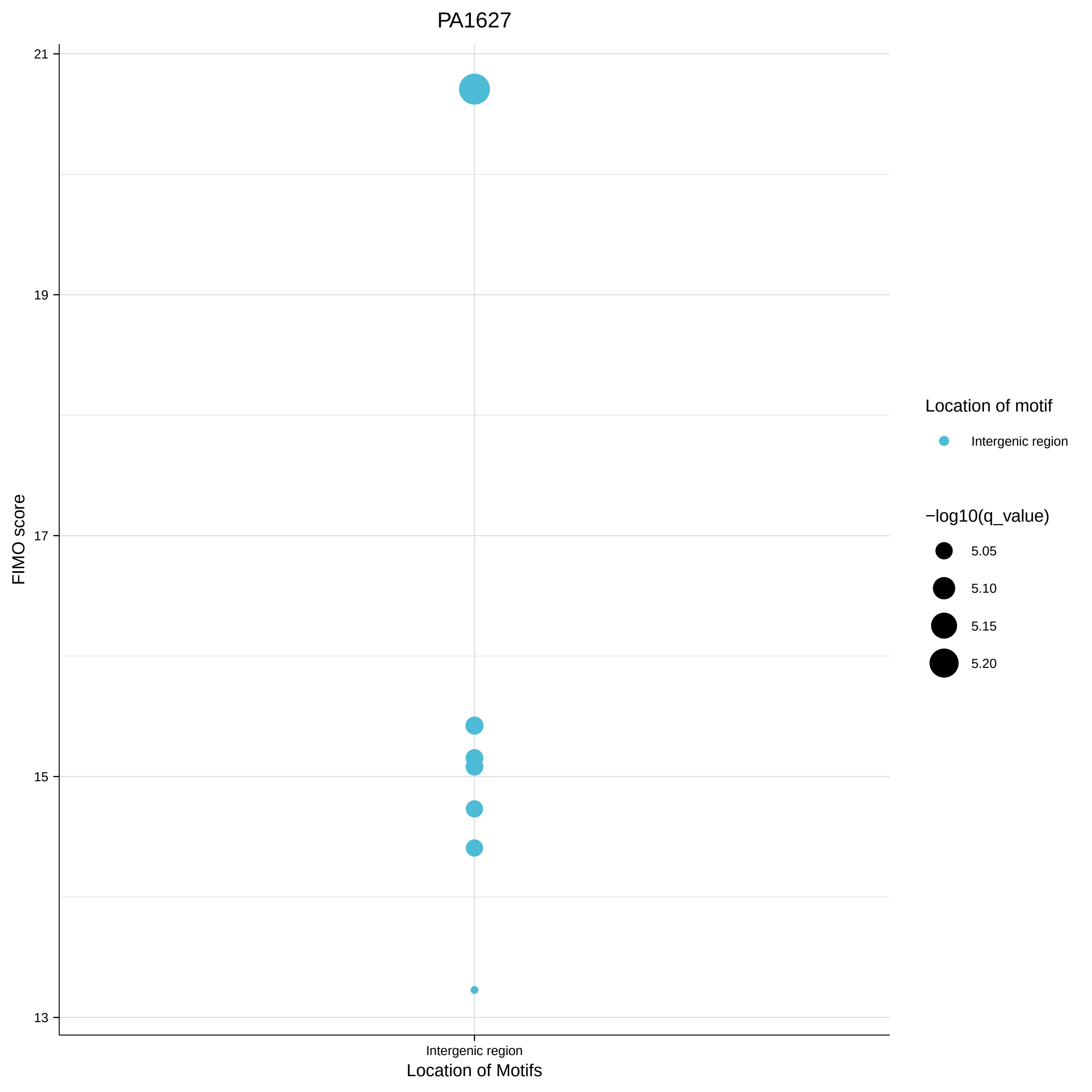
PA1627
21
19
17
15
13
FIMO score
Intergenic region
Location of Motifs
Location of motif
Intergenic region
−log10(q_value)
5.05
5.10
5.15
5.20

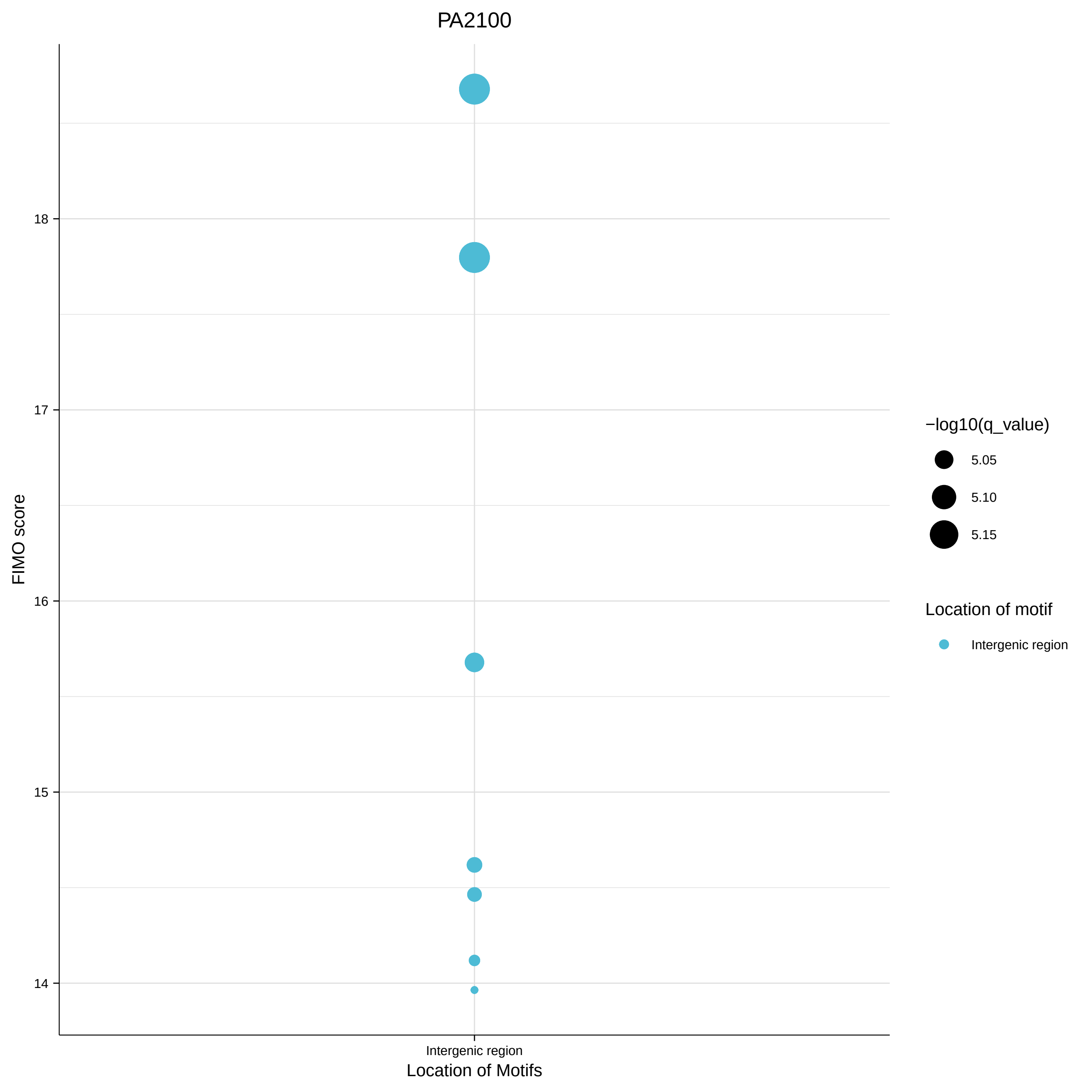
PA2100
FIMO score
18
17
16
15
14
Intergenic region
Location of Motifs
−log10(q_value)
5.05
5.10
5.15
Location of motif
Intergenic region

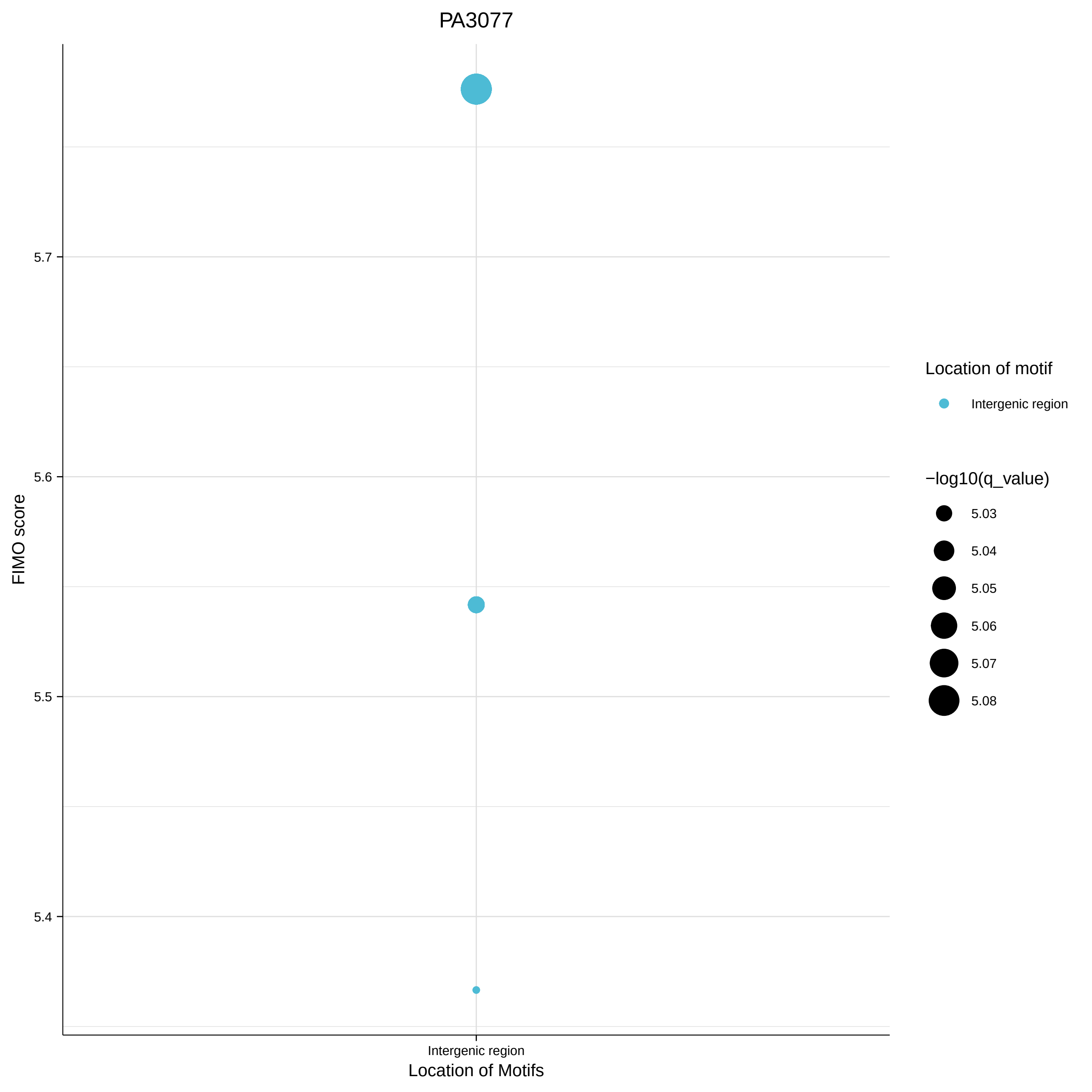
PA3077
FIMO score
5.7
5.6
5.5
5.4
Intergenic region
Location of Motifs
Location of motif
Intergenic region
−log10(q_value)
5.03
5.04
5.05
5.06
5.07
5.08

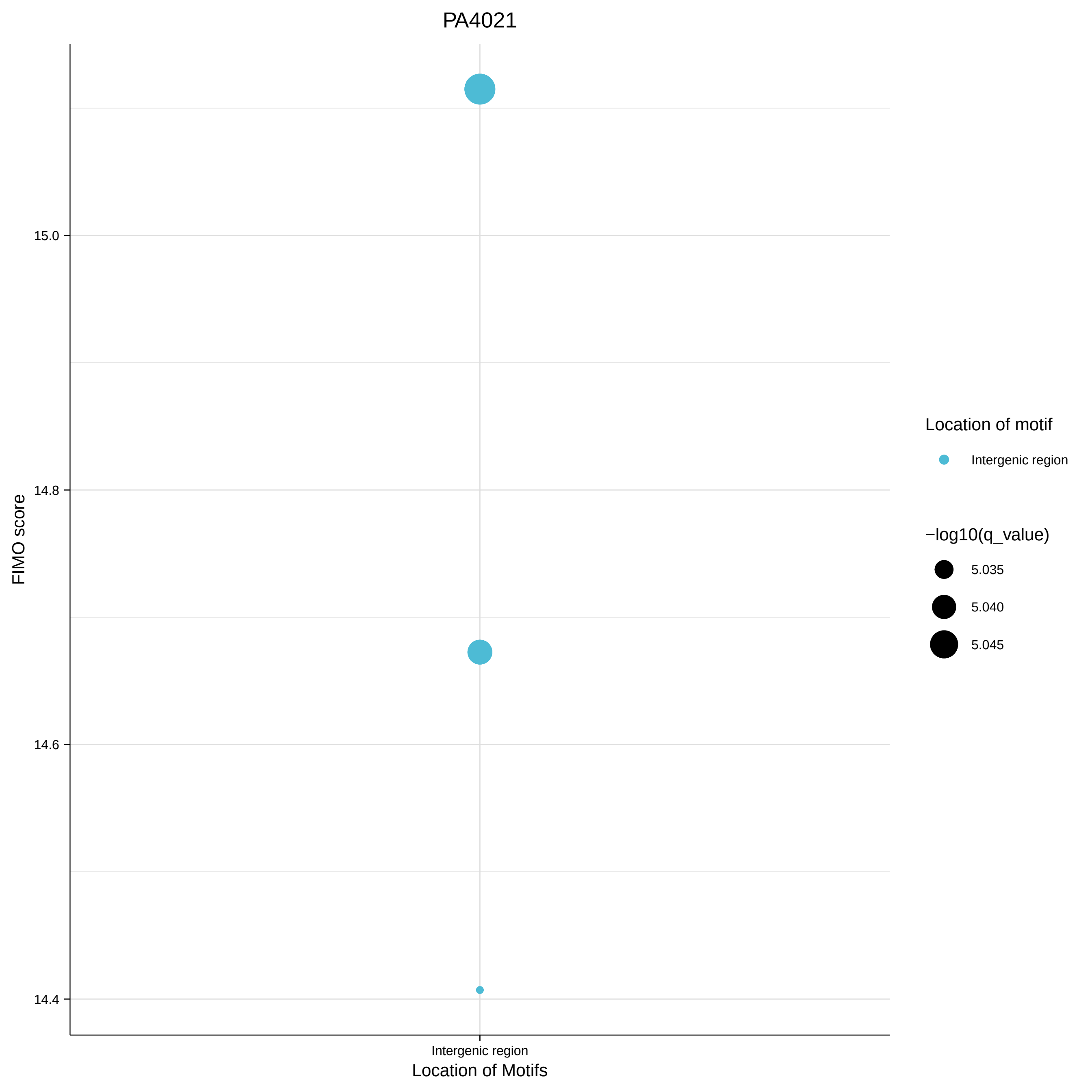
PA4021
FIMO score
15.0
14.8
14.6
14.4
Intergenic region
Location of Motifs
Location of motif
Intergenic region
−log10(q_value)
5.035
5.040
5.045

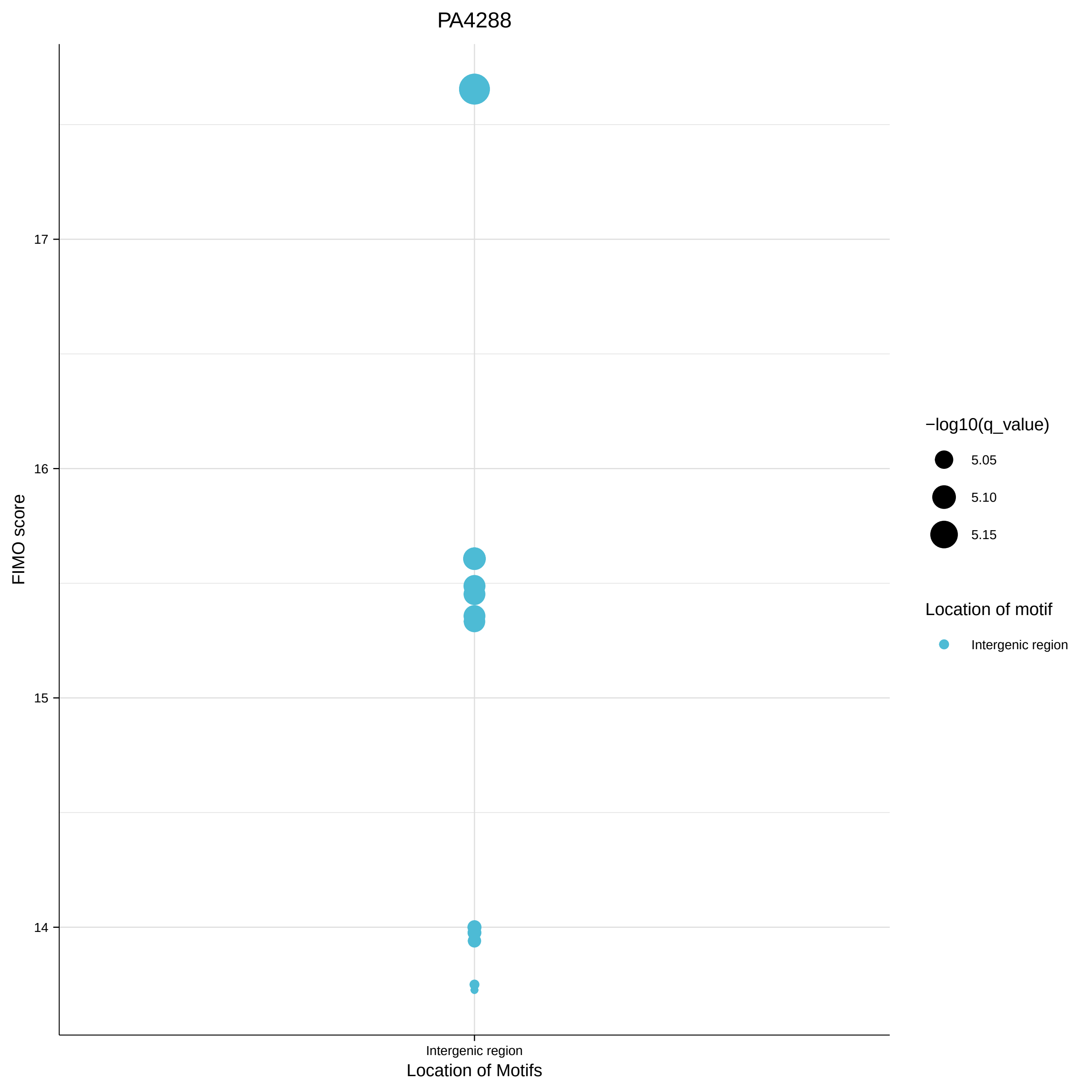
PA4288
FIMO score
17
16
15
14
Intergenic region
Location of Motifs
−log10(q_value)
5.05
5.10
5.15
Location of motif
Intergenic region

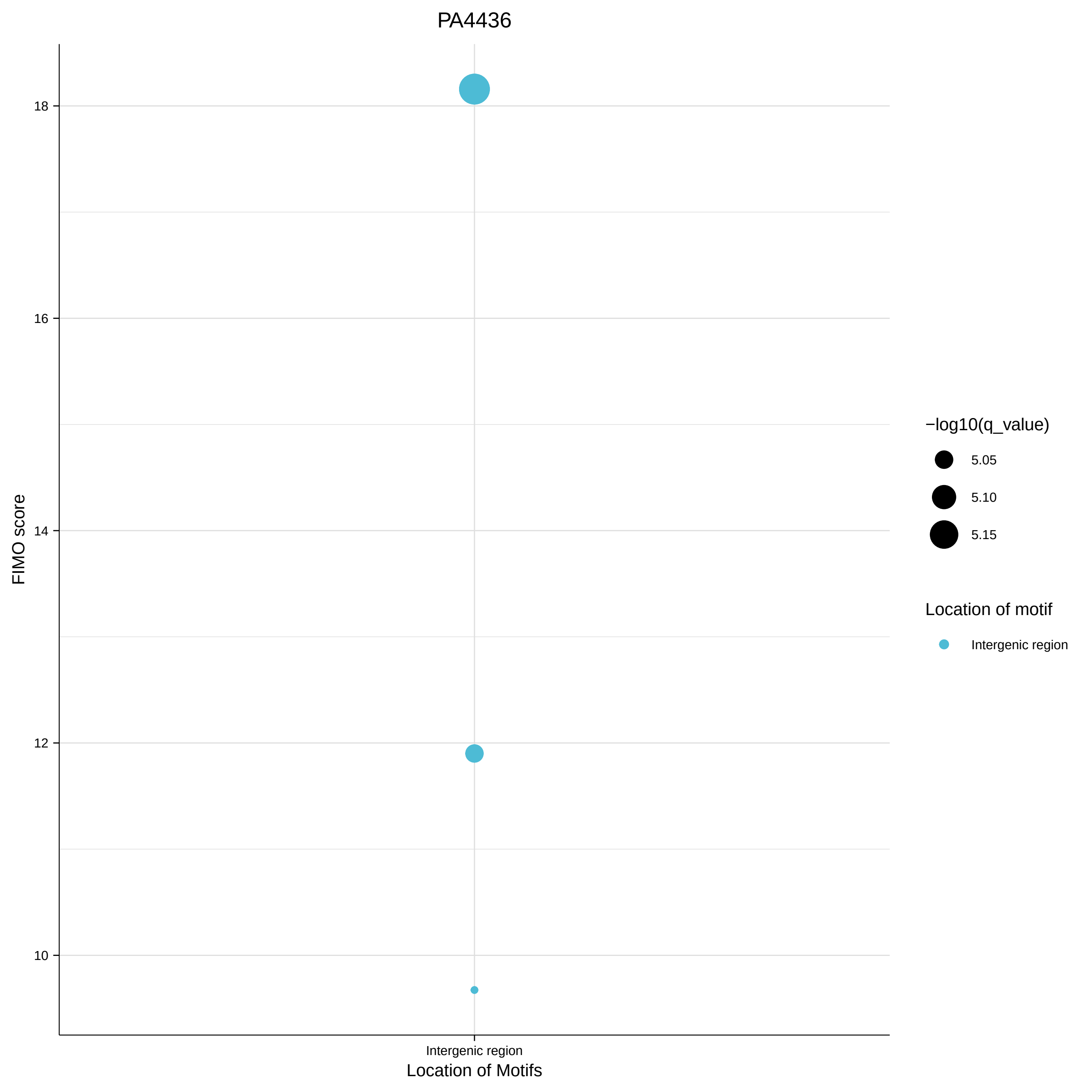
PA4436
FIMO score
18
16
14
12
10
Intergenic region
Location of Motifs
−log10(q_value)
5.05
5.10
5.15
Location of motif
Intergenic region

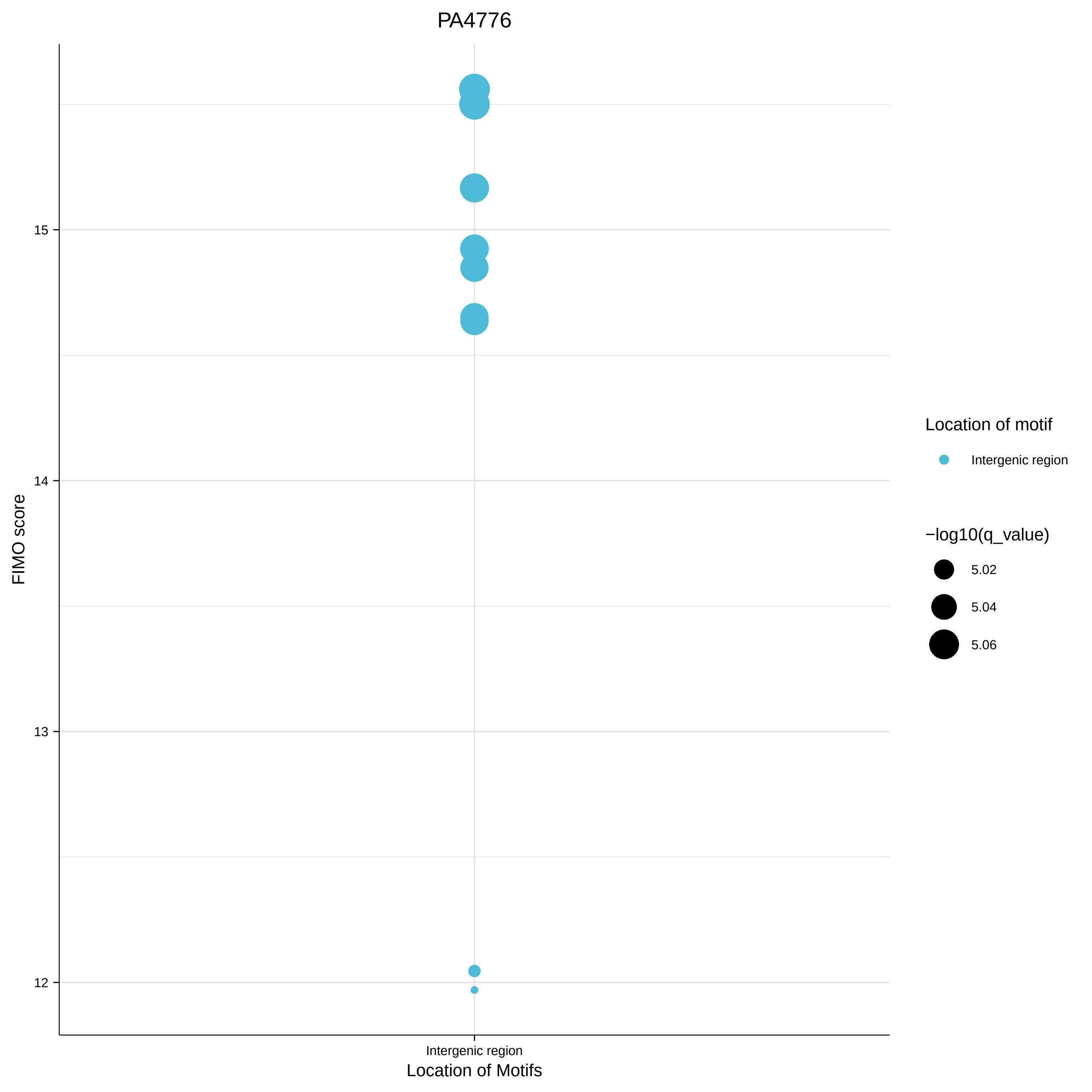
PA4776
FIMO score
15
14
13
12
Intergenic region
Location of Motifs
Location of motif
Intergenic region
−log10(q_value)
5.02
5.04
5.06

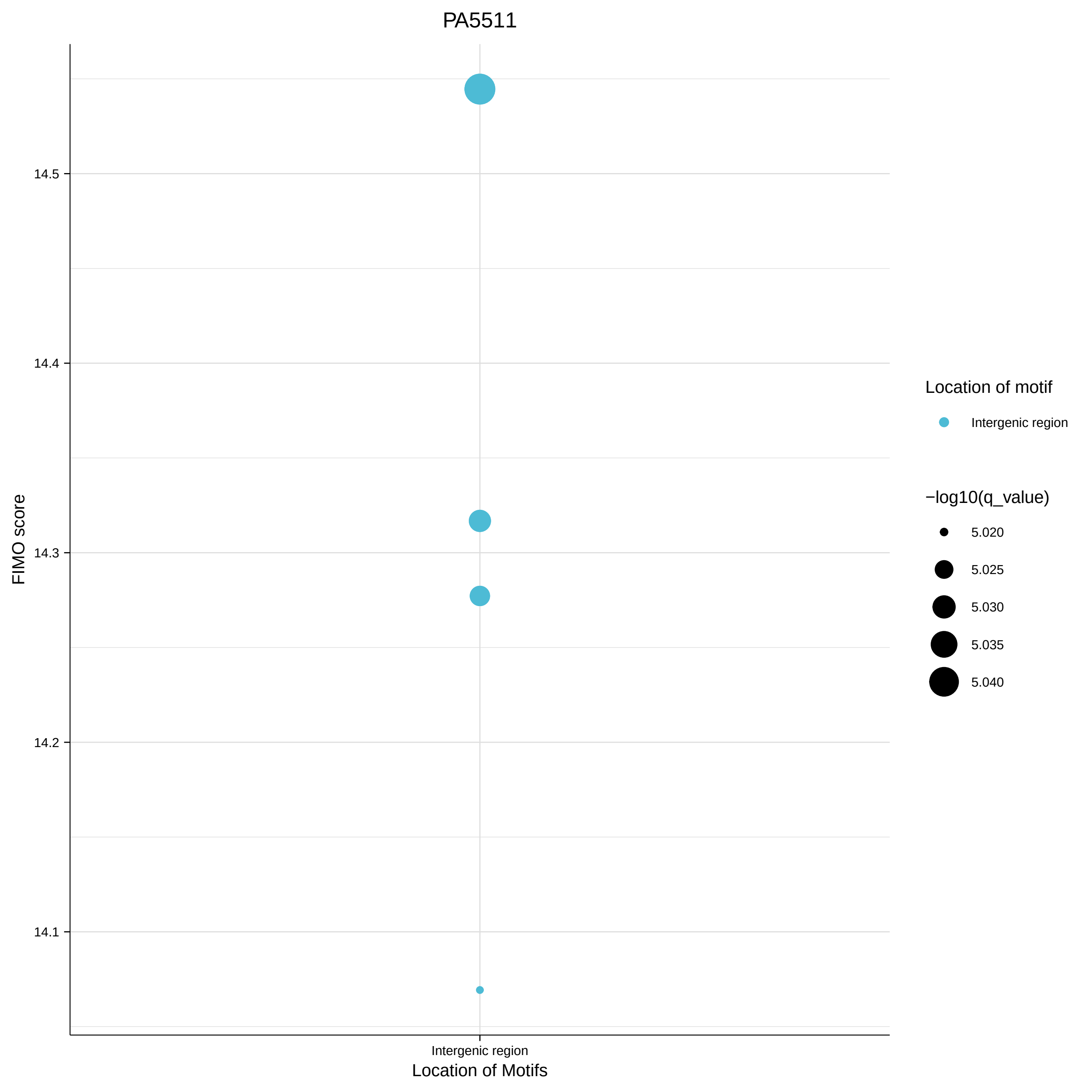
PA5511
FIMO score
14.5
14.4
14.3
14.2
14.1
Intergenic region
Location of Motifs
Location of motif
Intergenic region
−log10(q_value)
5.020
5.025
5.030
5.035
5.040
