# Supplementary material for: Bacterial Transcription Factors Bind to Coding Regions and Regulate Internal Cryptic Promoters
Source: mBio. 2022 Oct 6;13(5):e01643-22. doi: 10.1128/mbio.01643-22 (PMC9600179; doi:10.1128/mbio.01643-22)
Supplement: DATA SET S2 [file mbio.01643-22-s0008.pdf]

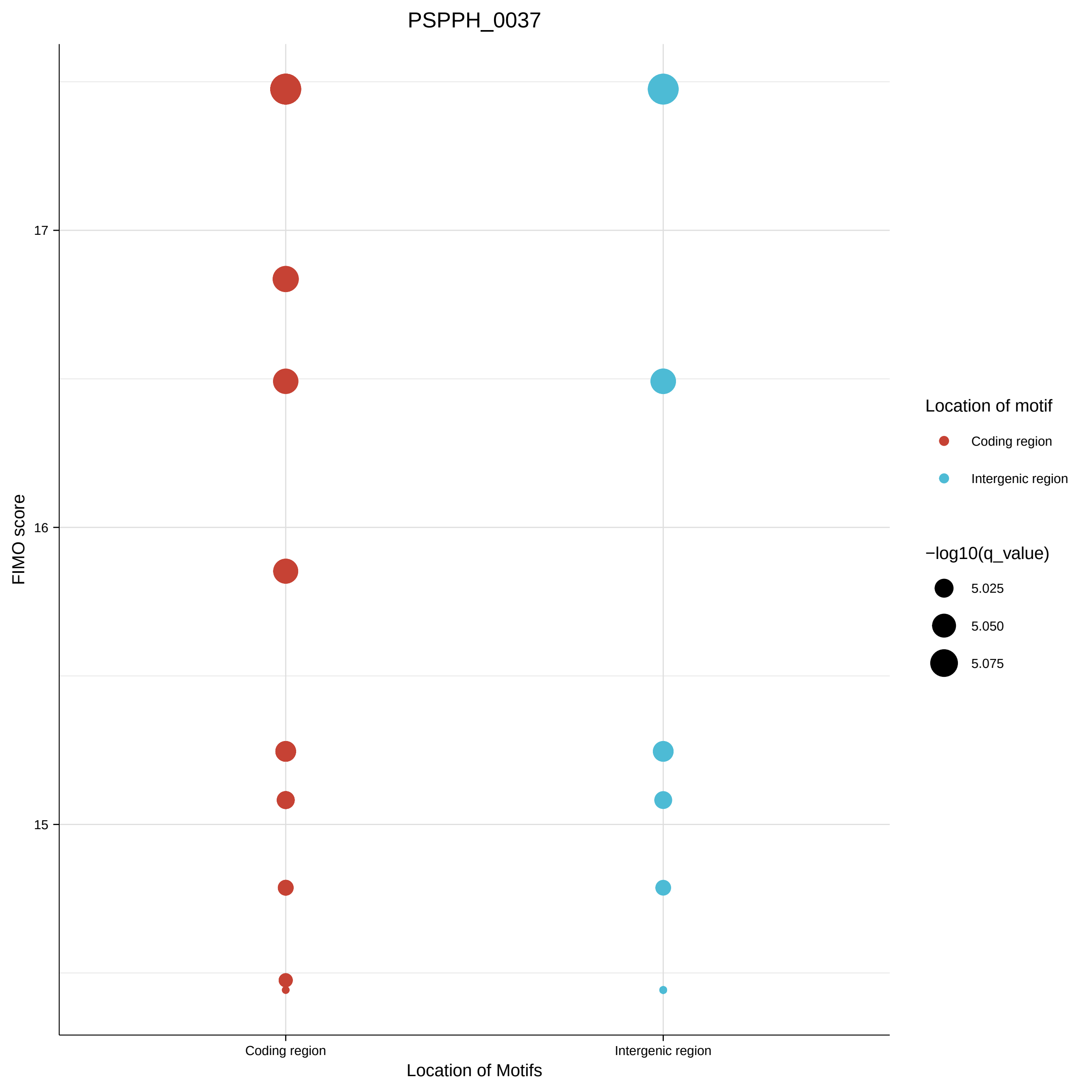

PSPPH_0037
FIMO score
17
16
15
Coding region
Intergenic region
Location of Motifs
Location of motif
Coding region
Intergenic region
−log10(q_value)
5.025
5.050
5.075

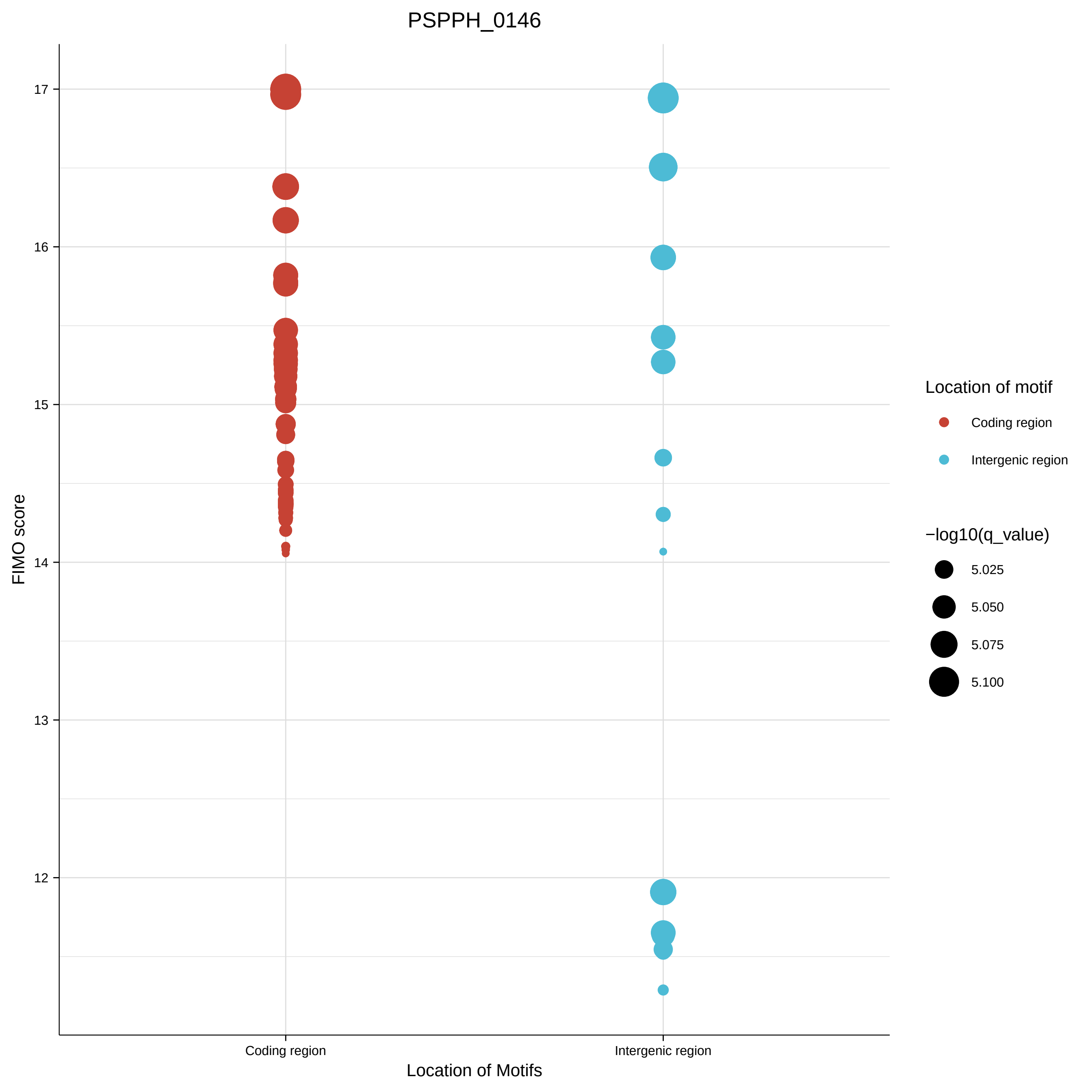

PSPPH_0146
FIMO score
17
16
15
14
13
12
Coding region
Intergenic region
Location of Motifs
Location of motif
Coding region
Intergenic region
−log10(q_value)
5.025
5.050
5.075
5.100

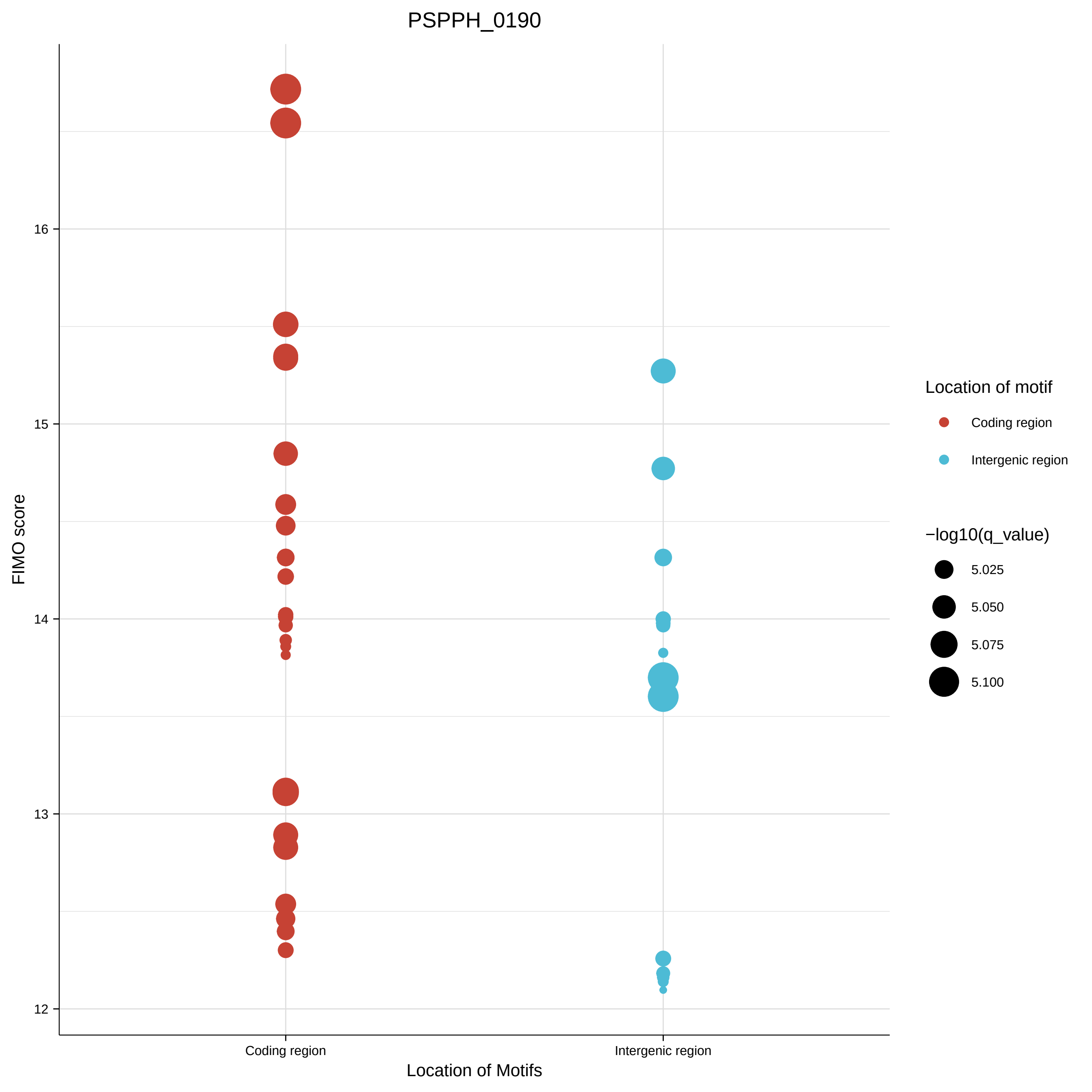
PSPPH_0190
FIMO score
16
15
14
13
12
Coding region
Intergenic region
Location of Motifs
Location of motif
Coding region
Intergenic region
−log10(q_value)
5.025
5.050
5.075
5.100

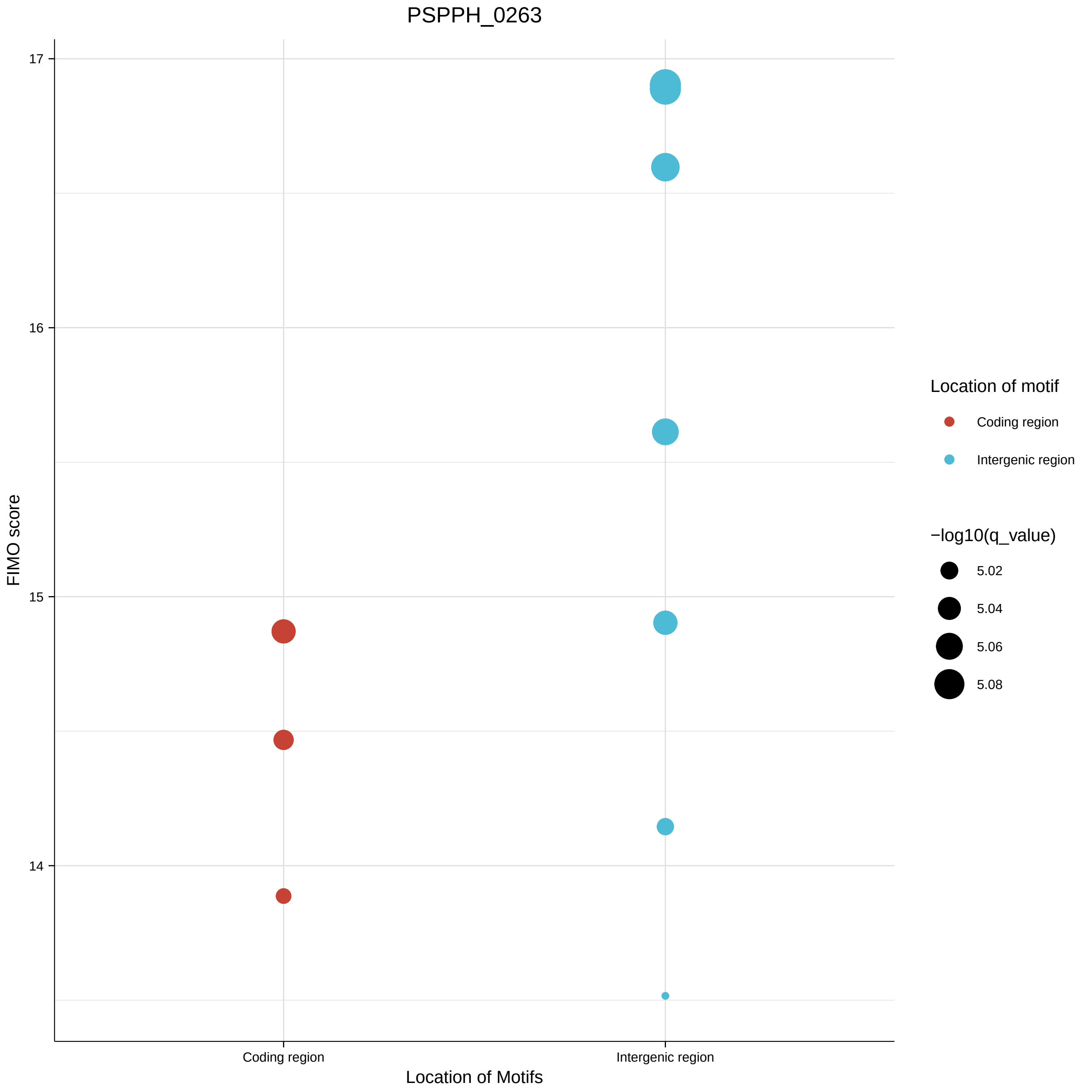

PSPPH_0263
17
16
15
14
FIMO score
Coding region
Intergenic region
Location of Motifs
Location of motif
Coding region
Intergenic region
−log10(q_value)
5.02
5.04
5.06
5.08

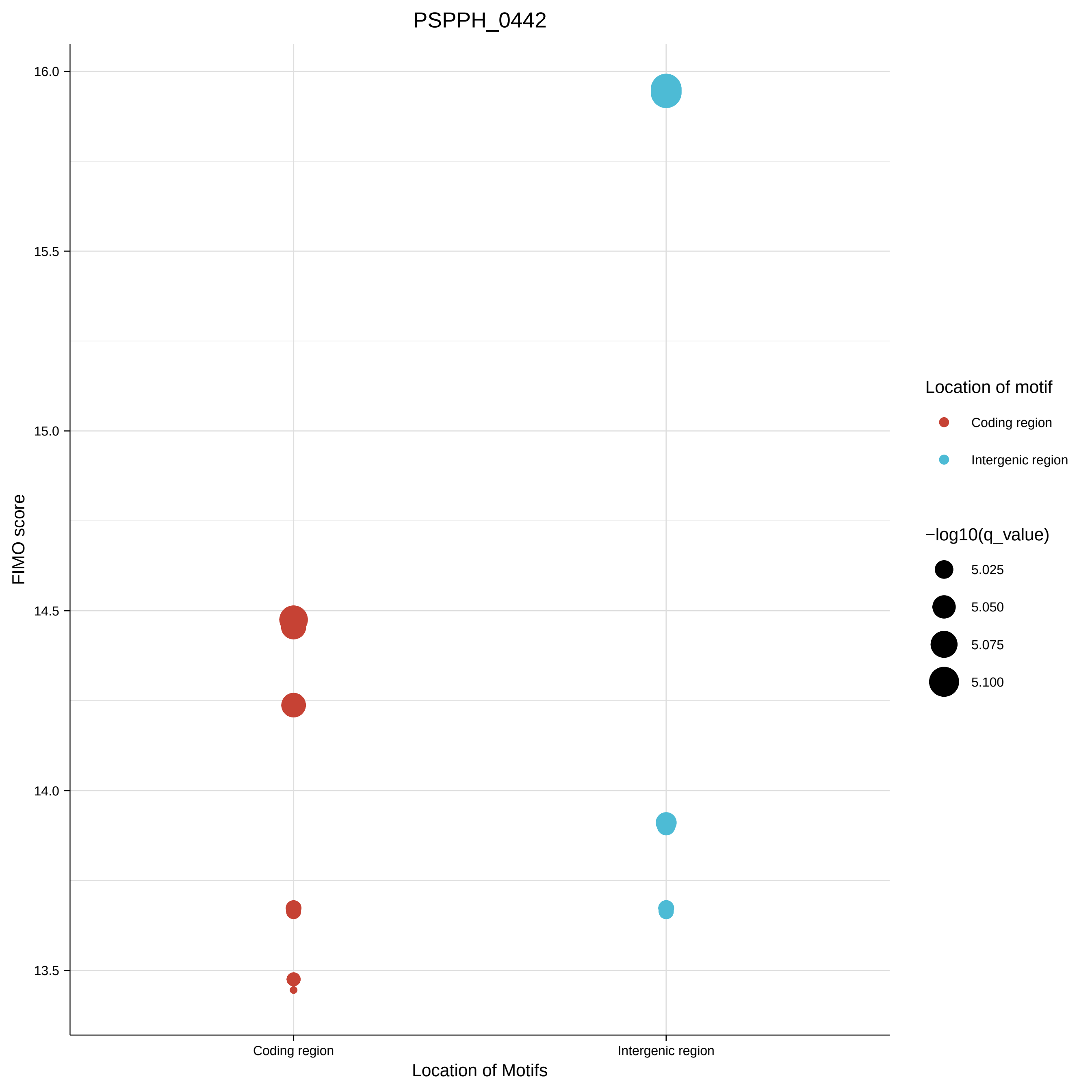
PSPPH_0442
FIMO score
16.0
15.5
15.0
14.5
14.0
13.5
Coding region
Intergenic region
Location of Motifs
Location of motif
Coding region
Intergenic region
−log10(q_value)
5.025
5.050
5.075
5.100

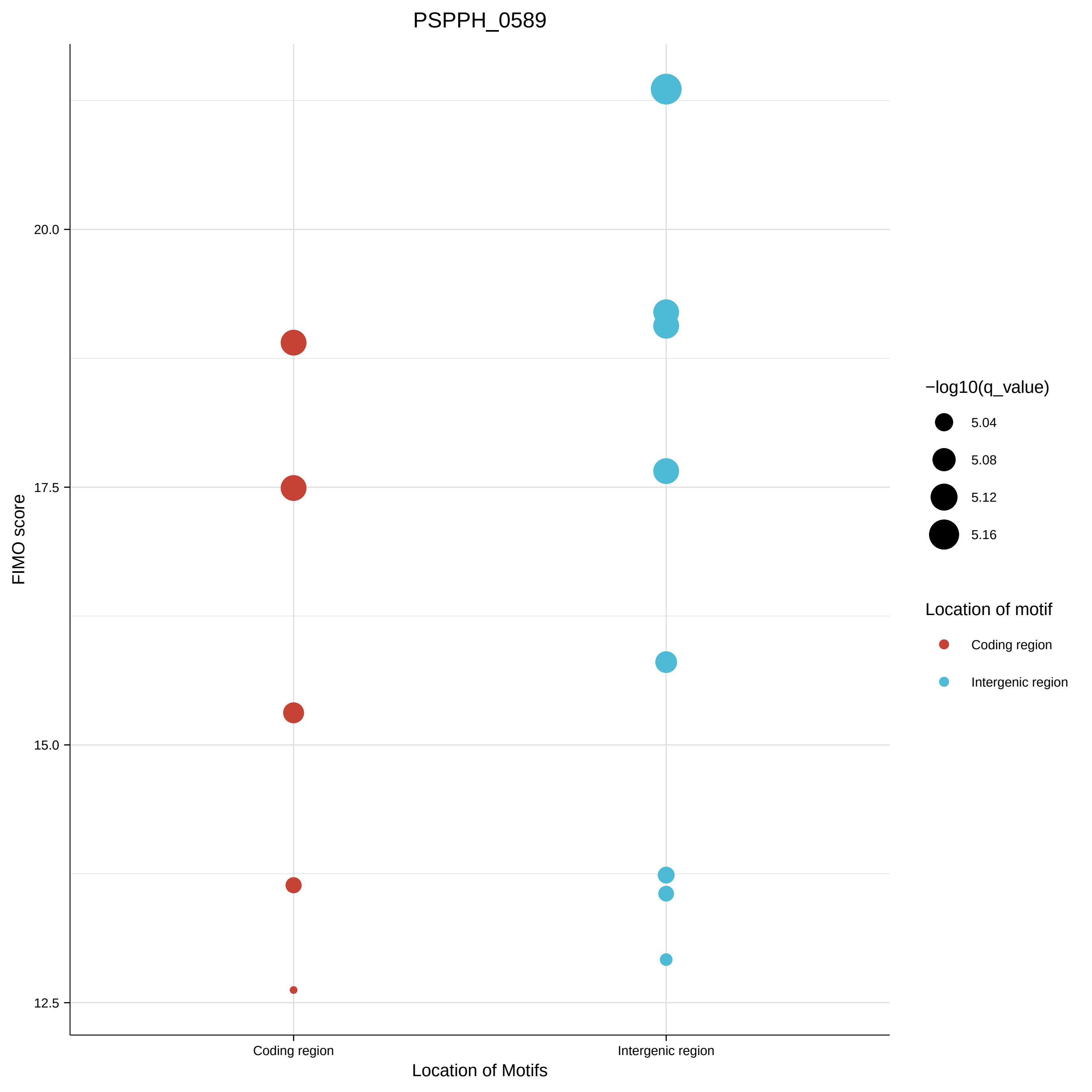
PSPPH_0589
FIMO score
20.0
17.5
15.0
12.5
Coding region
Intergenic region
Location of Motifs
−log10(q_value)
5.04
5.08
5.12
5.16
Location of motif
Coding region
Intergenic region

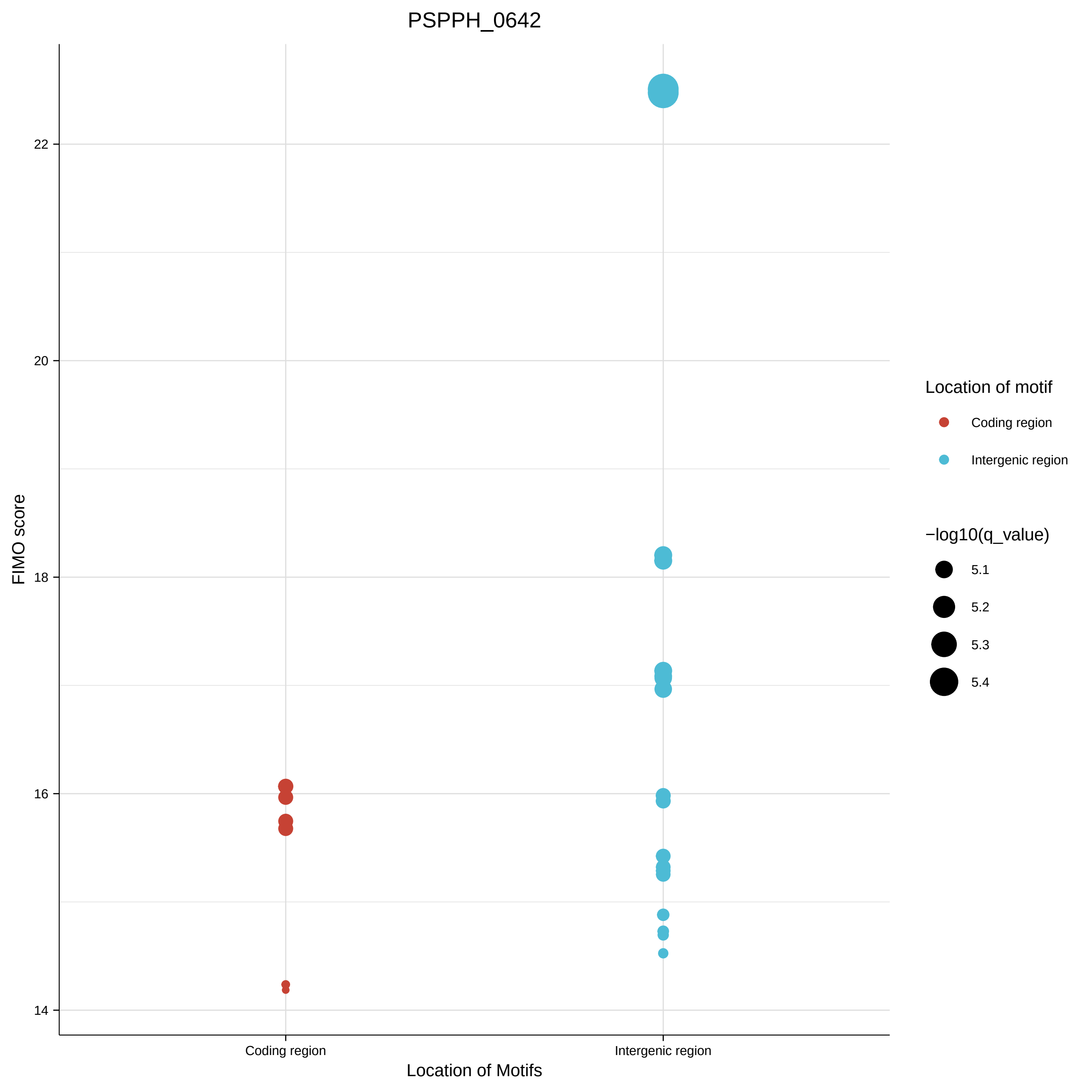

PSPPH_0642
FIMO score
22
20
18
16
14
Coding region
Intergenic region
Location of Motifs
Location of motif
Coding region
Intergenic region
−log10(q_value)
5.1
5.2
5.3
5.4

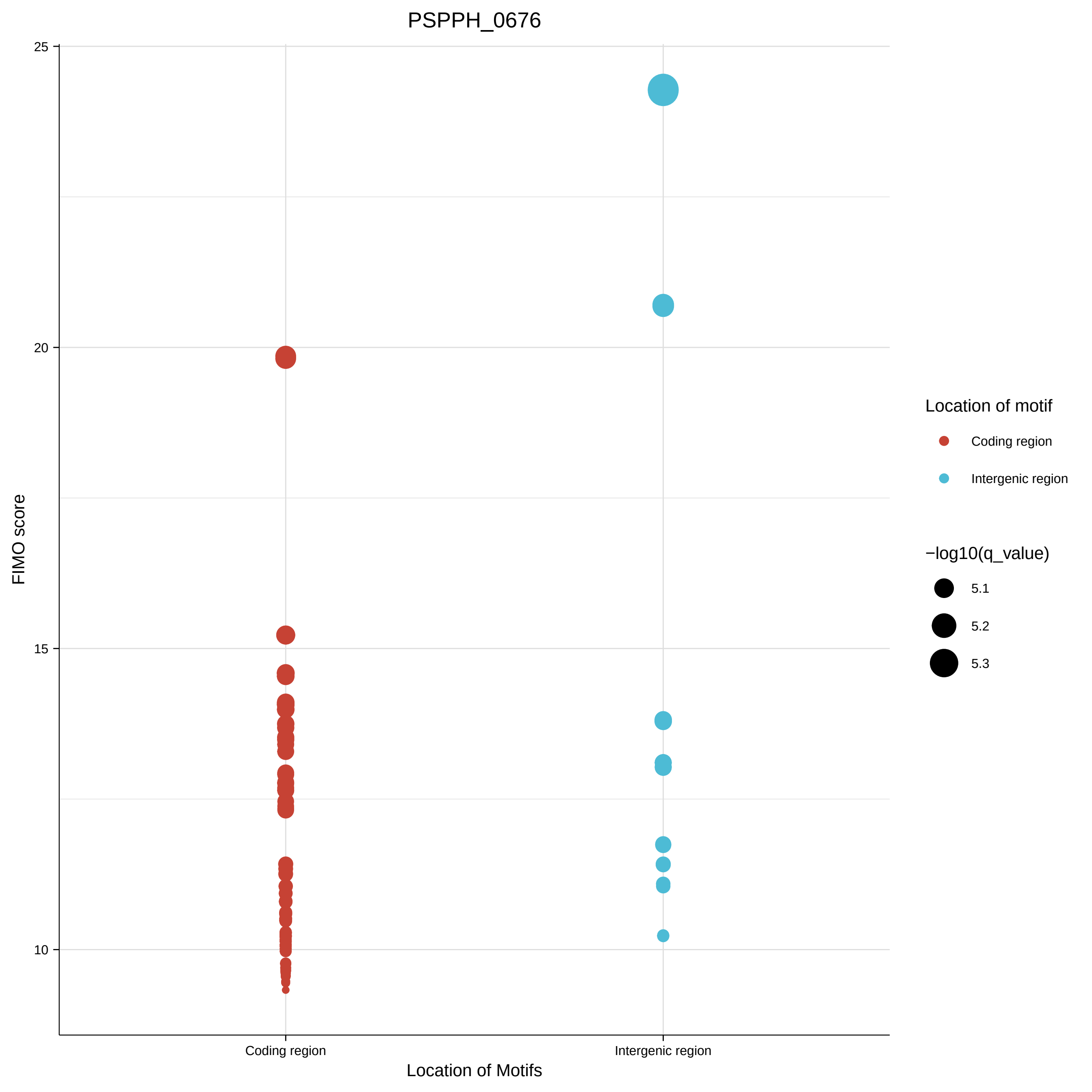

PSPPH_0676
FIMO score
25
20
15
10
Coding region
Intergenic region
Location of Motifs
Location of motif
Coding region
Intergenic region
−log10(q_value)
5.1
5.2
5.3

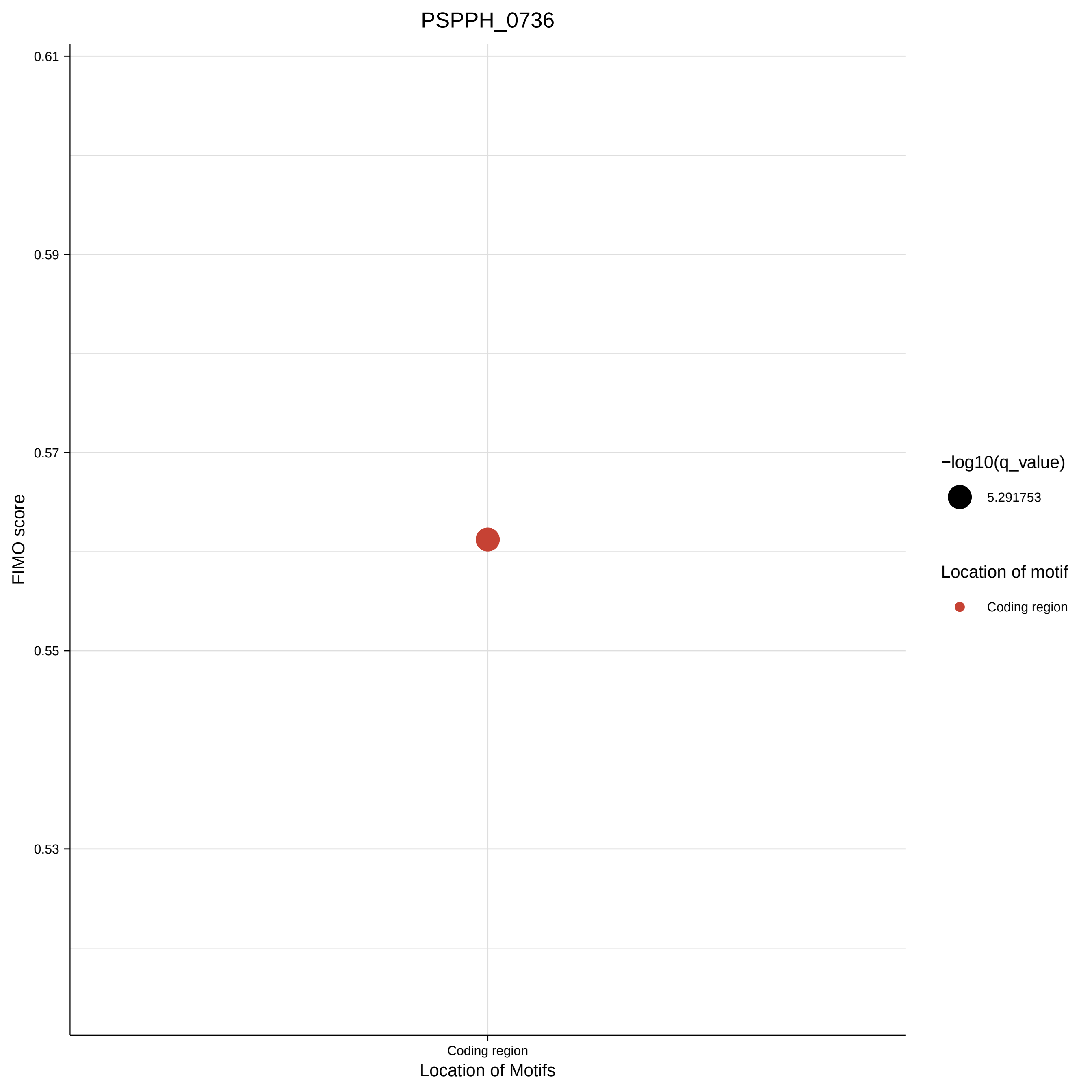
PSPPH_0736
0.61
0.59
0.57
0.55
0.53
FIMO score
Coding region
Location of Motifs
−log10(q_value)
5.291753
Location of motif
Coding region

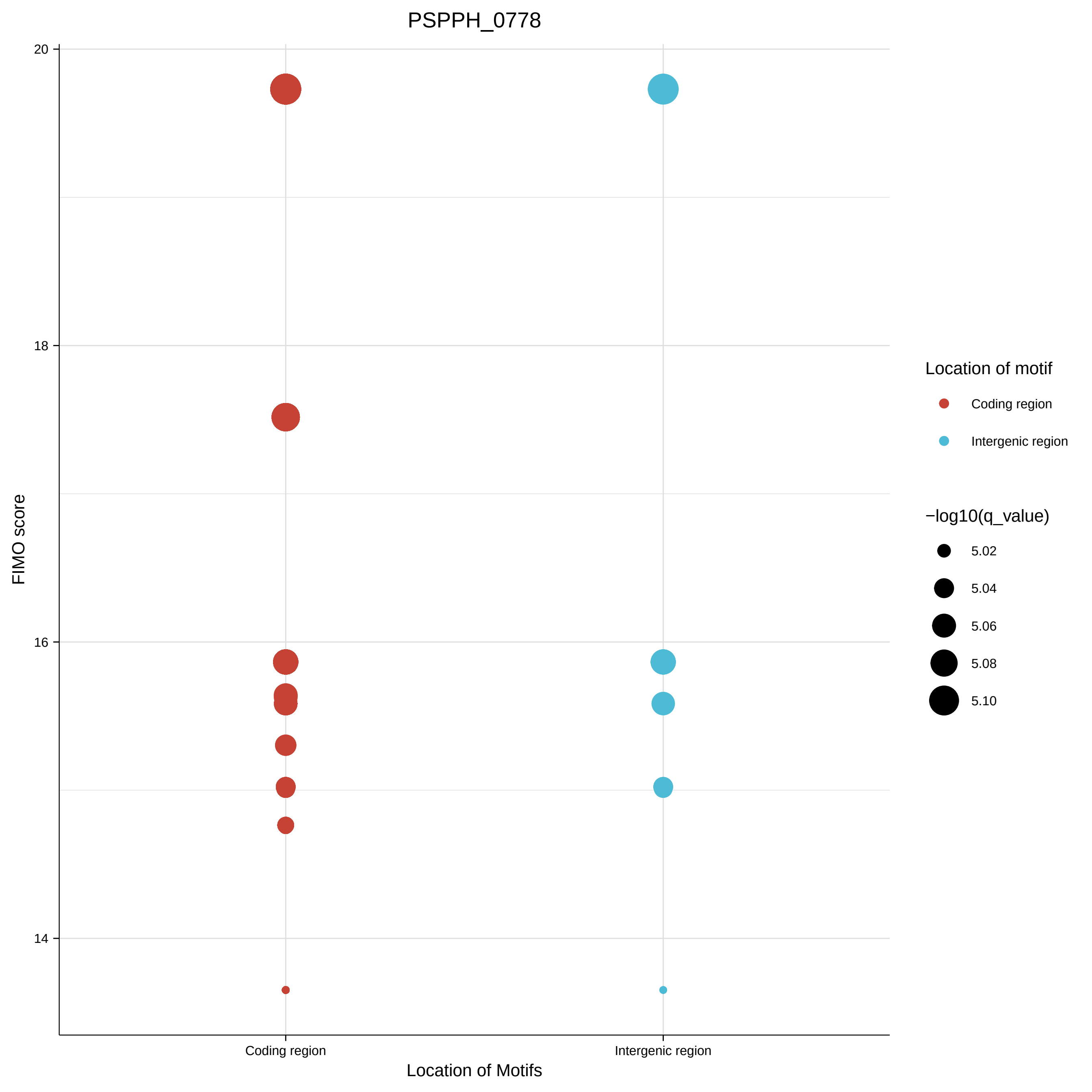

PSPPH_0778
20
18
16
14
FIMO score
Coding region
Intergenic region
Location of Motifs
Location of motif
Coding region
Intergenic region
−log10(q_value)
5.02
5.04
5.06
5.08
5.10

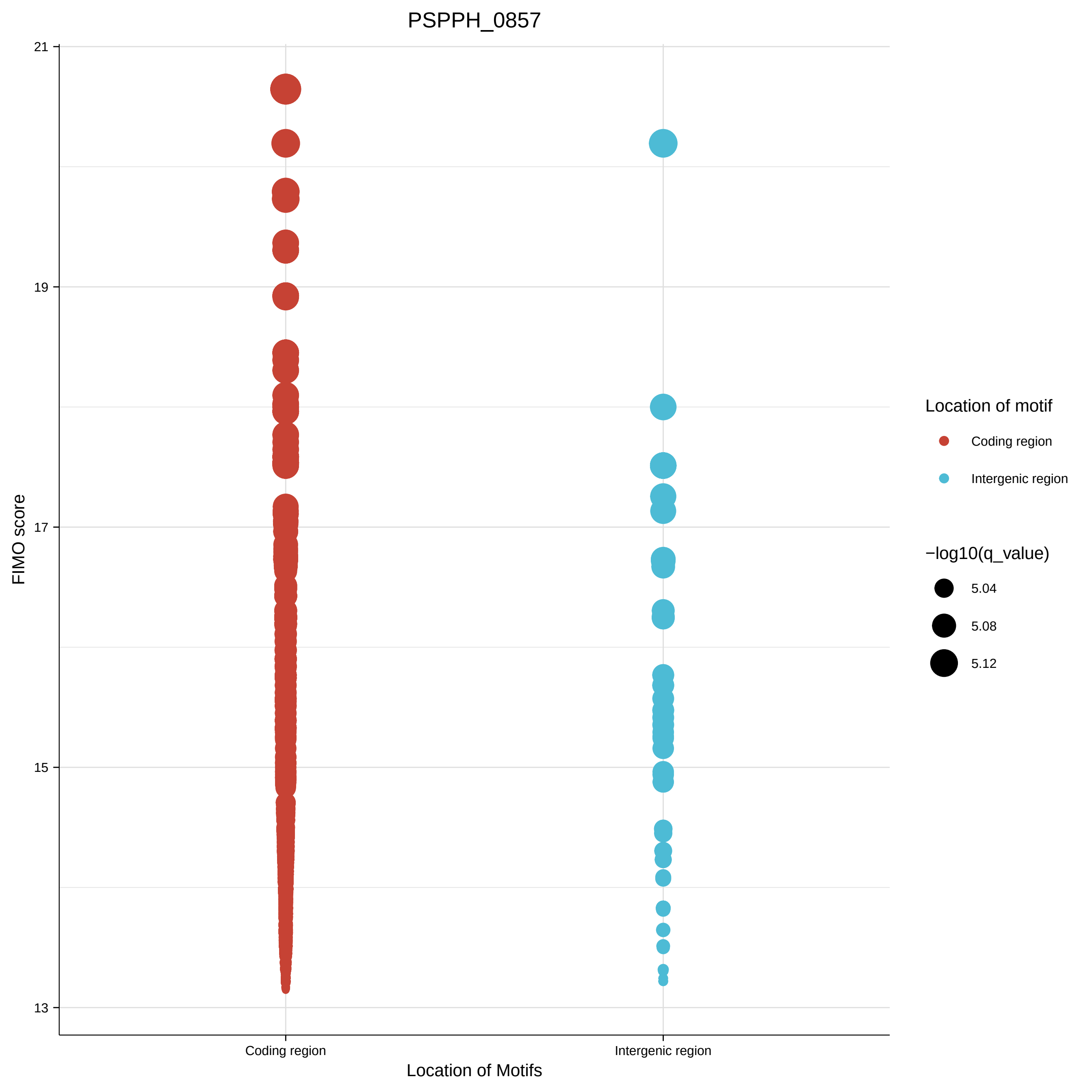
PSPPH_0857
FIMO score
21
19
17
15
13
Coding region
Intergenic region
Location of Motifs
Location of motif
Coding region
Intergenic region
−log10(q_value)
5.04
5.08
5.12

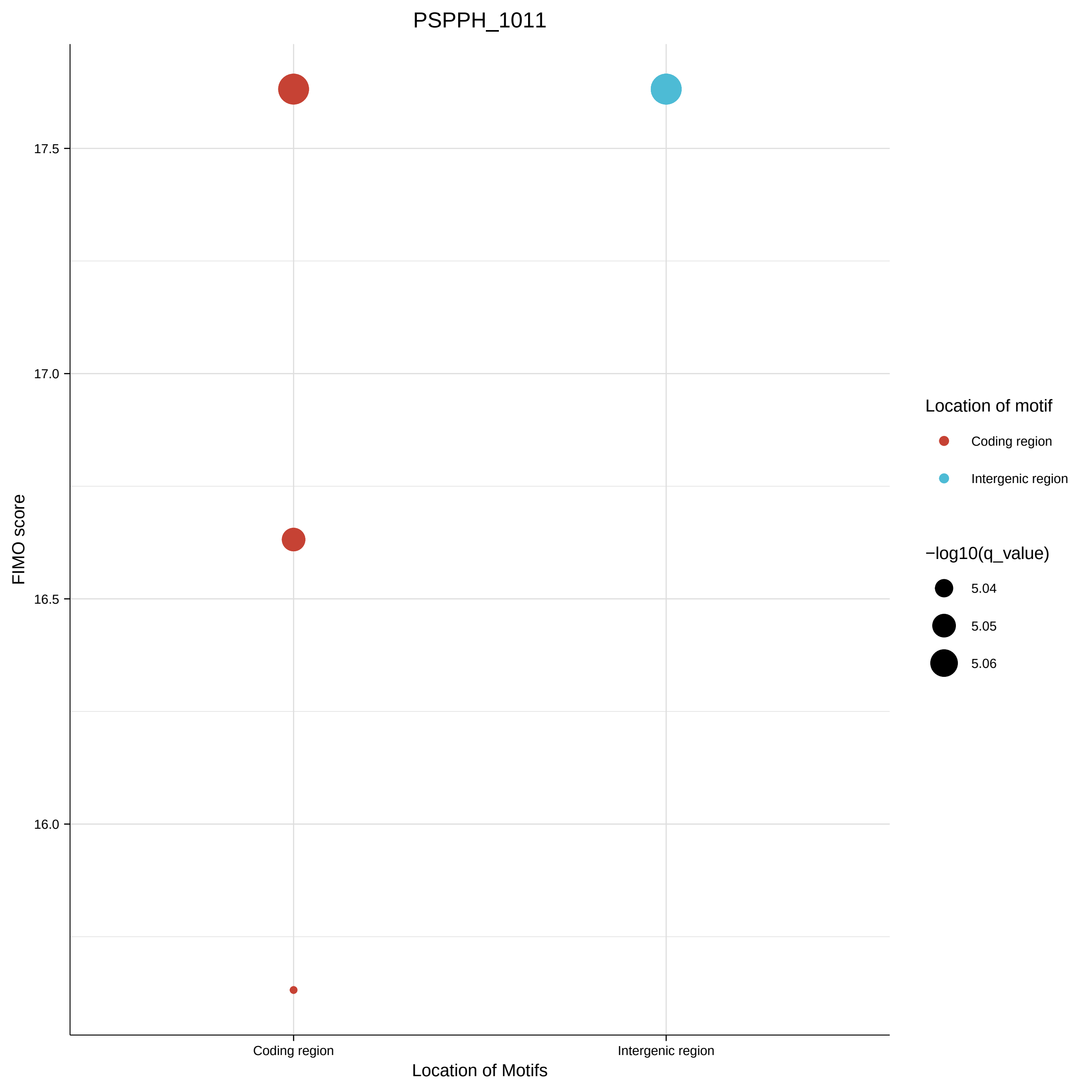
PSPPH_1011
FIMO score
17.5
17.0
16.5
16.0
Coding region
Intergenic region
Location of Motifs
Location of motif
Coding region
Intergenic region
−log10(q_value)
5.04
5.05
5.06

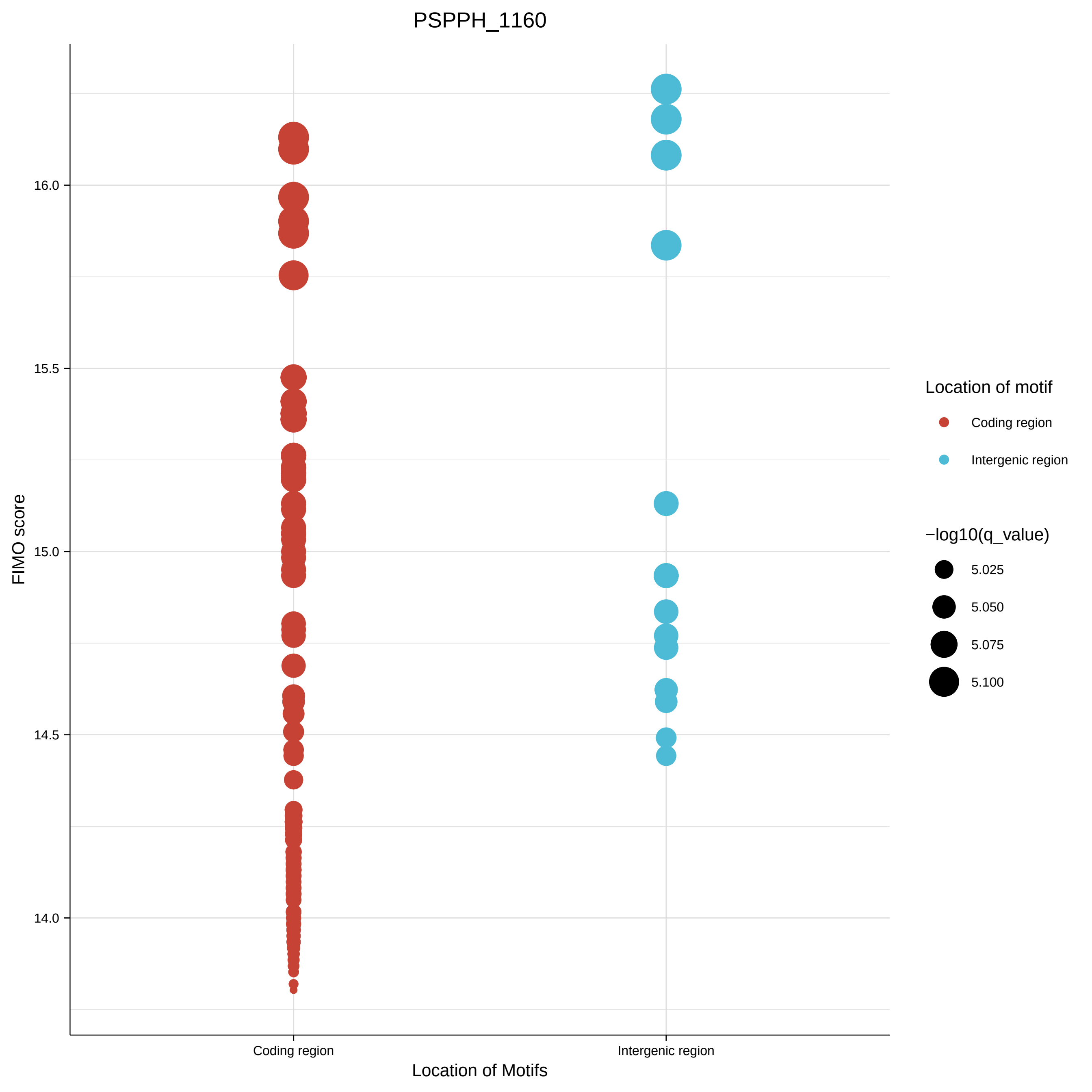

PSPPH_1160
FIMO score
16.0
15.5
15.0
14.5
14.0
Coding region
Intergenic region
Location of Motifs
Location of motif
Coding region
Intergenic region
−log10(q_value)
5.025
5.050
5.075
5.100

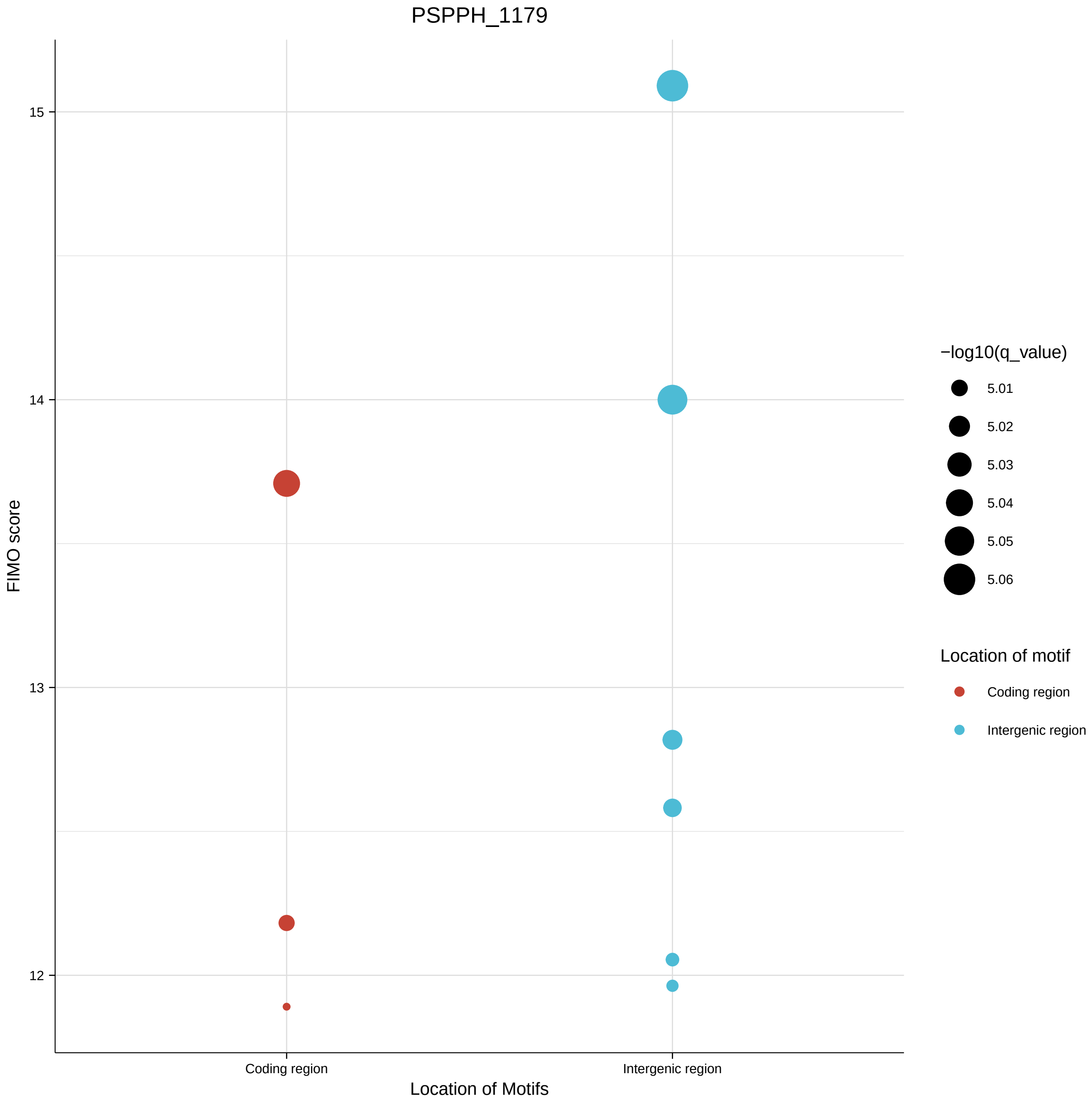

PSPPH_1179
FIMO score
15
14
13
12
Coding region
Intergenic region
Location of Motifs
−log10(q_value)
5.01
5.02
5.03
5.04
5.05
5.06
Location of motif
Coding region
Intergenic region

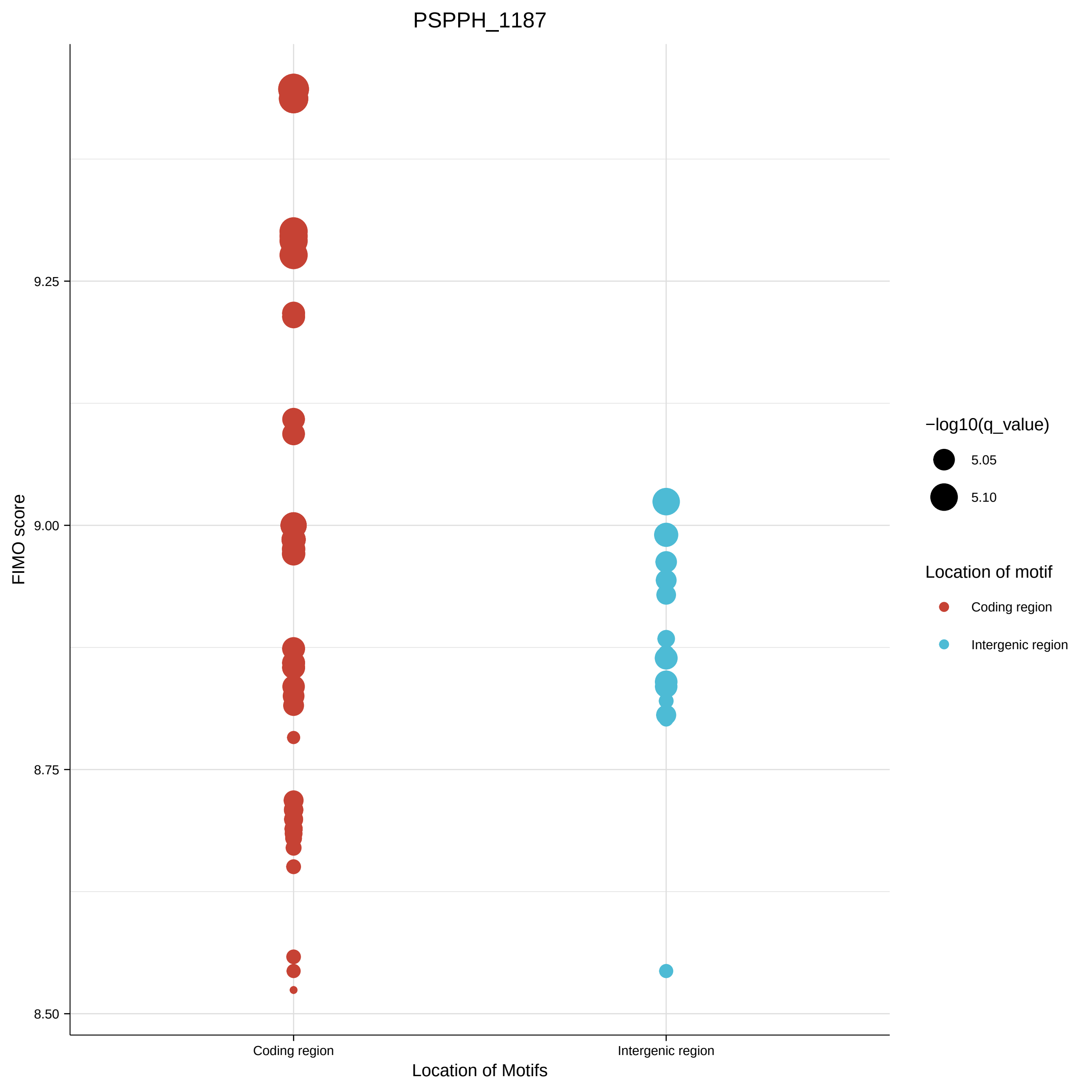
PSPPH_1187
FIMO score
9.25
9.00
8.75
8.50
Coding region
Intergenic region
Location of Motifs
−log10(q_value)
5.05
5.10
Location of motif
Coding region
Intergenic region

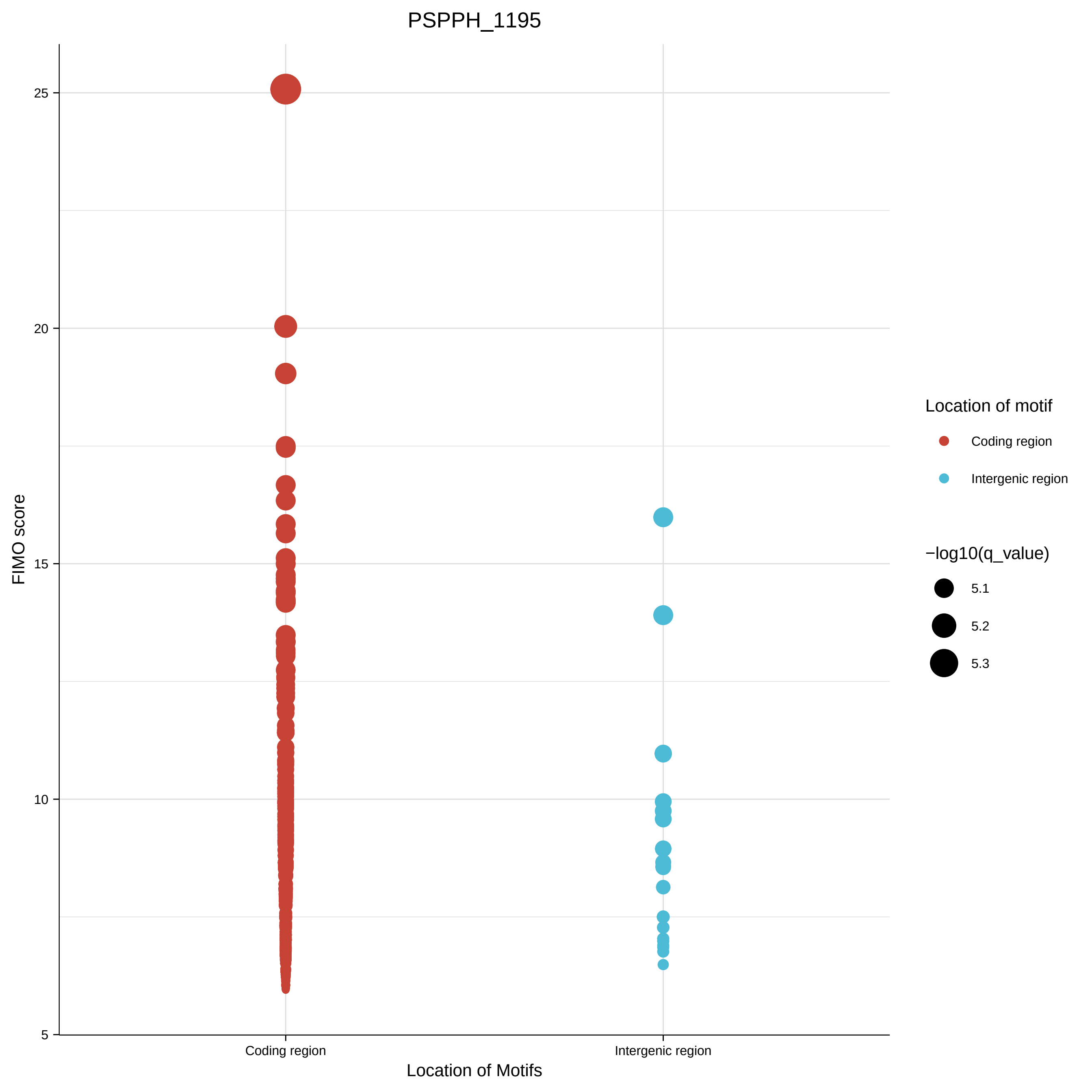
PSPPH_1195
FIMO score
25
20
15
10
5
Coding region
Intergenic region
Location of Motifs
Location of motif
Coding region
Intergenic region
−log10(q_value)
5.1
5.2
5.3

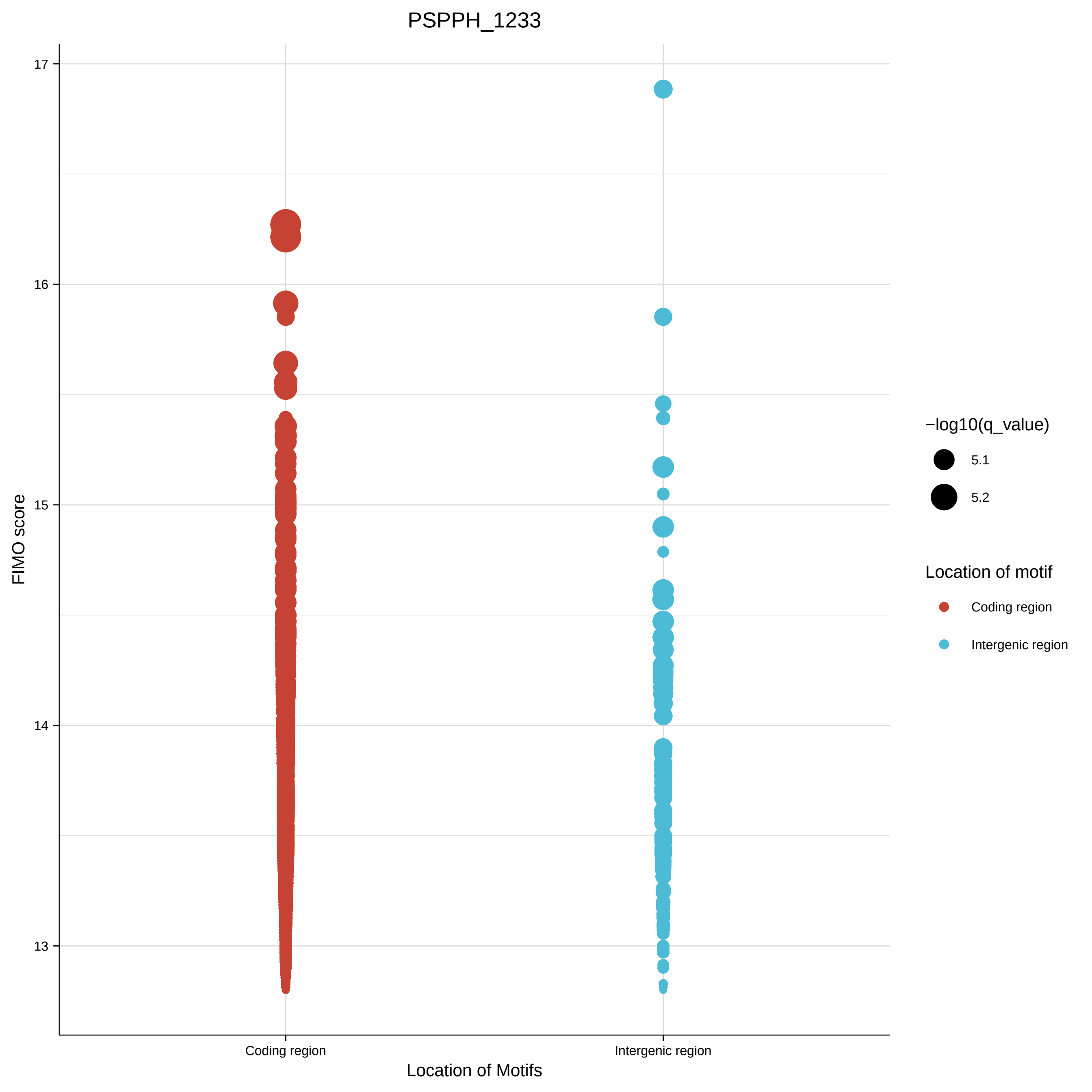

PSPPH_1233
FIMO score
17
16
15
14
13
Coding region
Intergenic region
Location of Motifs
−log10(q_value)
5.1
5.2
Location of motif
Coding region
Intergenic region

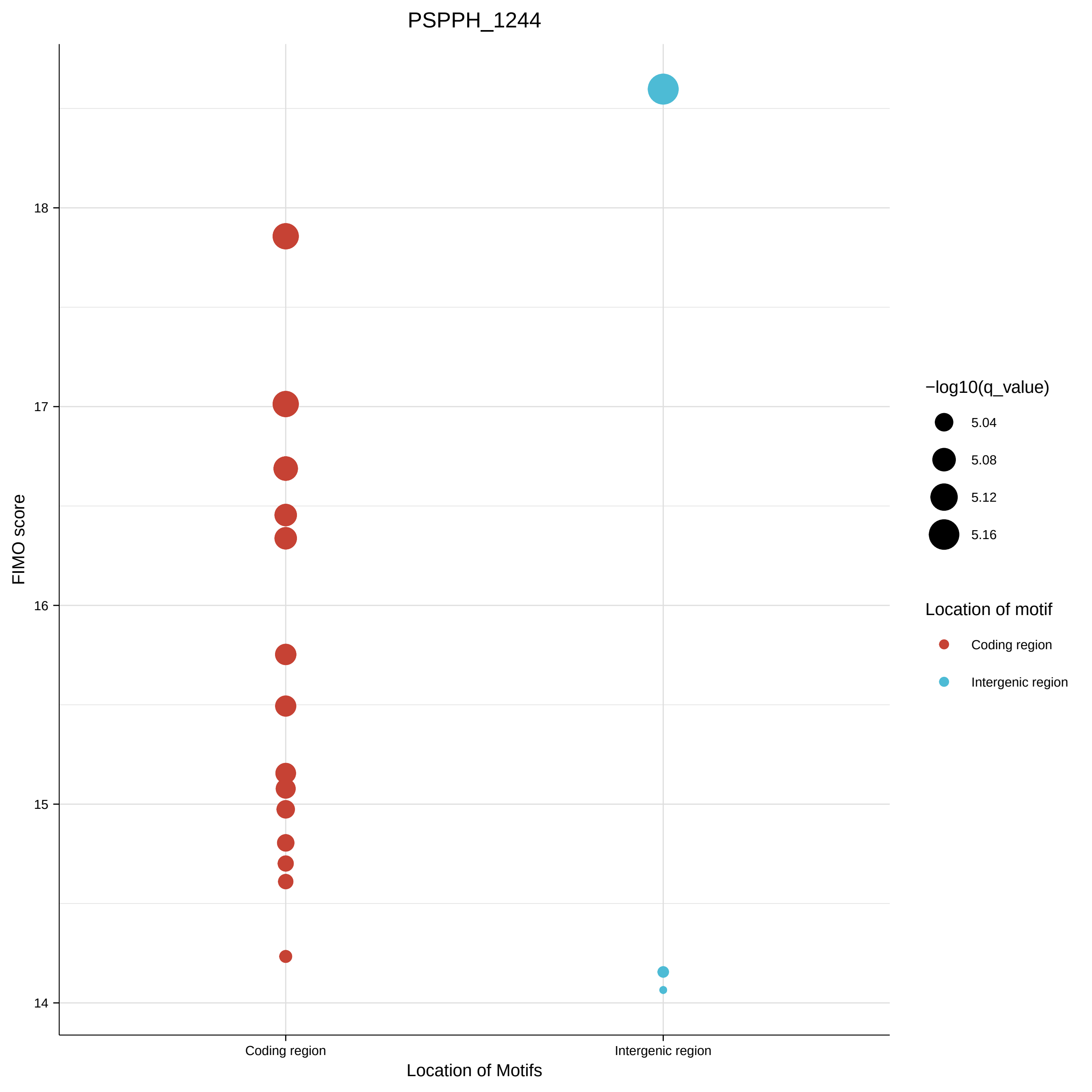
PSPPH_1244
FIMO score
18
17
16
15
14
Coding region
Intergenic region
Location of Motifs
−log10(q_value)
5.04
5.08
5.12
5.16
Location of motif
Coding region
Intergenic region

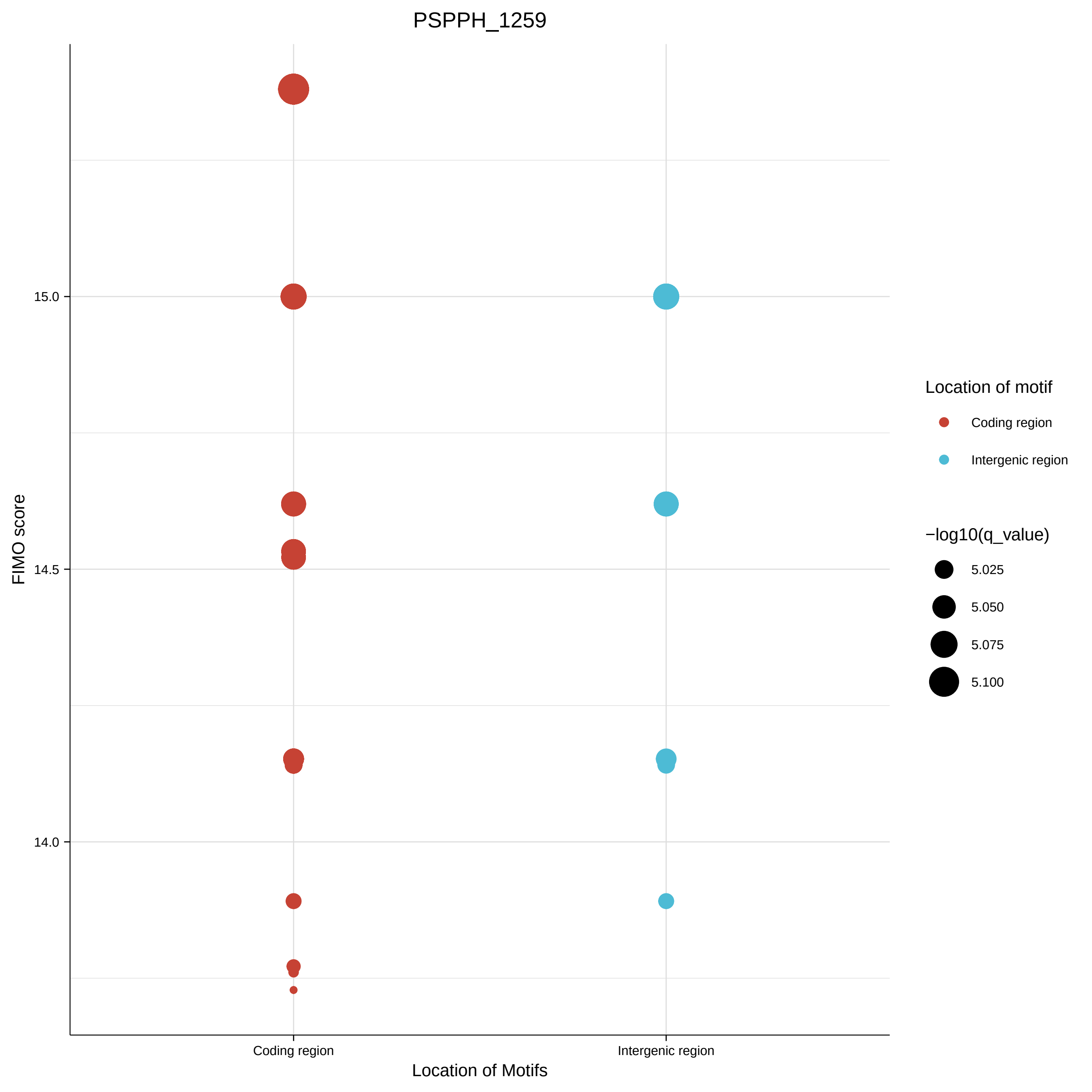

PSPPH_1259
FIMO score
15.0
14.5
14.0
Coding region
Intergenic region
Location of Motifs
Location of motif
Coding region
Intergenic region
−log10(q_value)
5.025
5.050
5.075
5.100

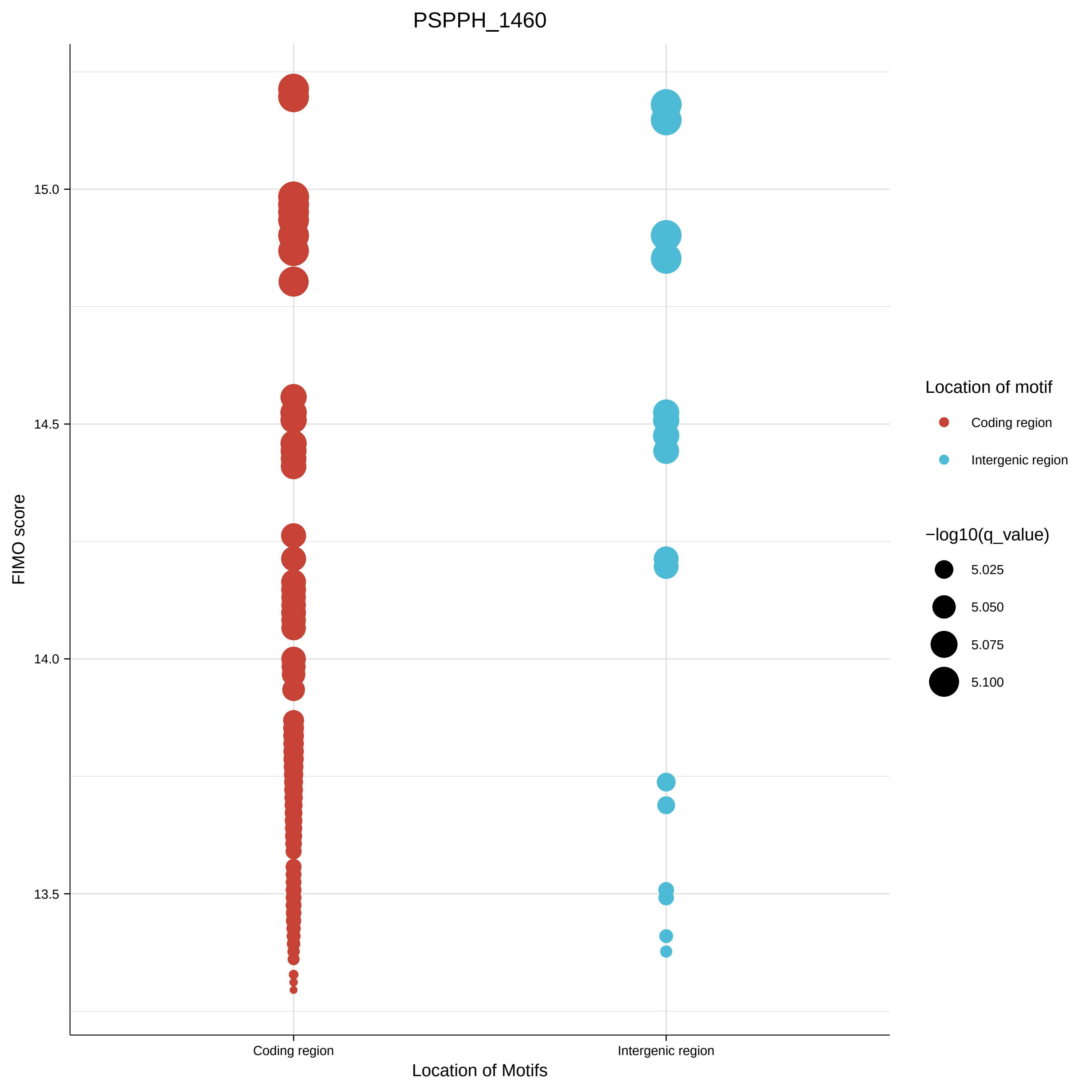
PSPPH_1460
FIMO score
15.0
14.5
14.0
13.5
Coding region
Intergenic region
Location of Motifs
Location of motif
Coding region
Intergenic region
−log10(q_value)
5.025
5.050
5.075
5.100

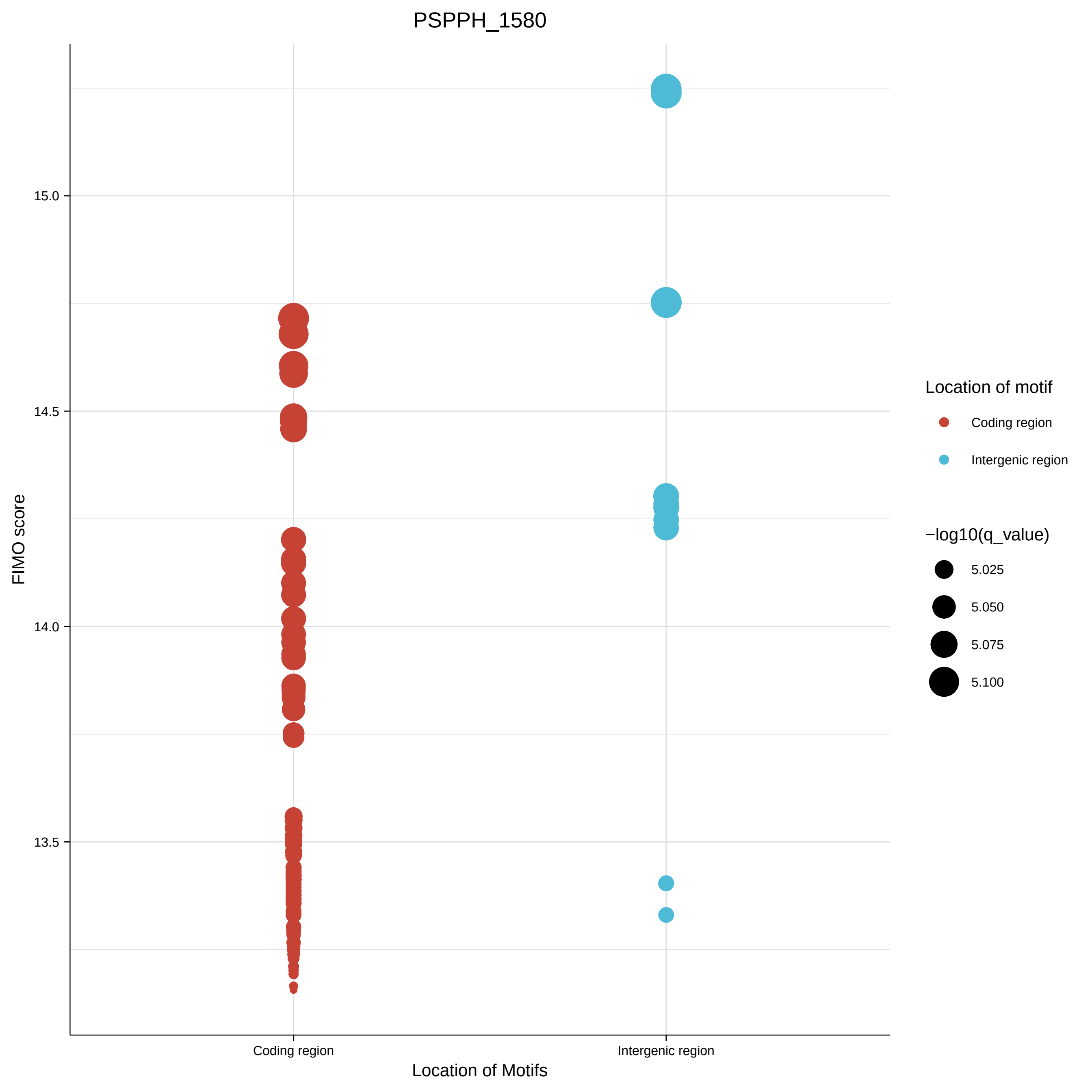
PSPPH_1580
FIMO score
15.0
14.5
14.0
13.5
Coding region
Intergenic region
Location of Motifs
Location of motif
Coding region
Intergenic region
−log10(q_value)
5.025
5.050
5.075
5.100

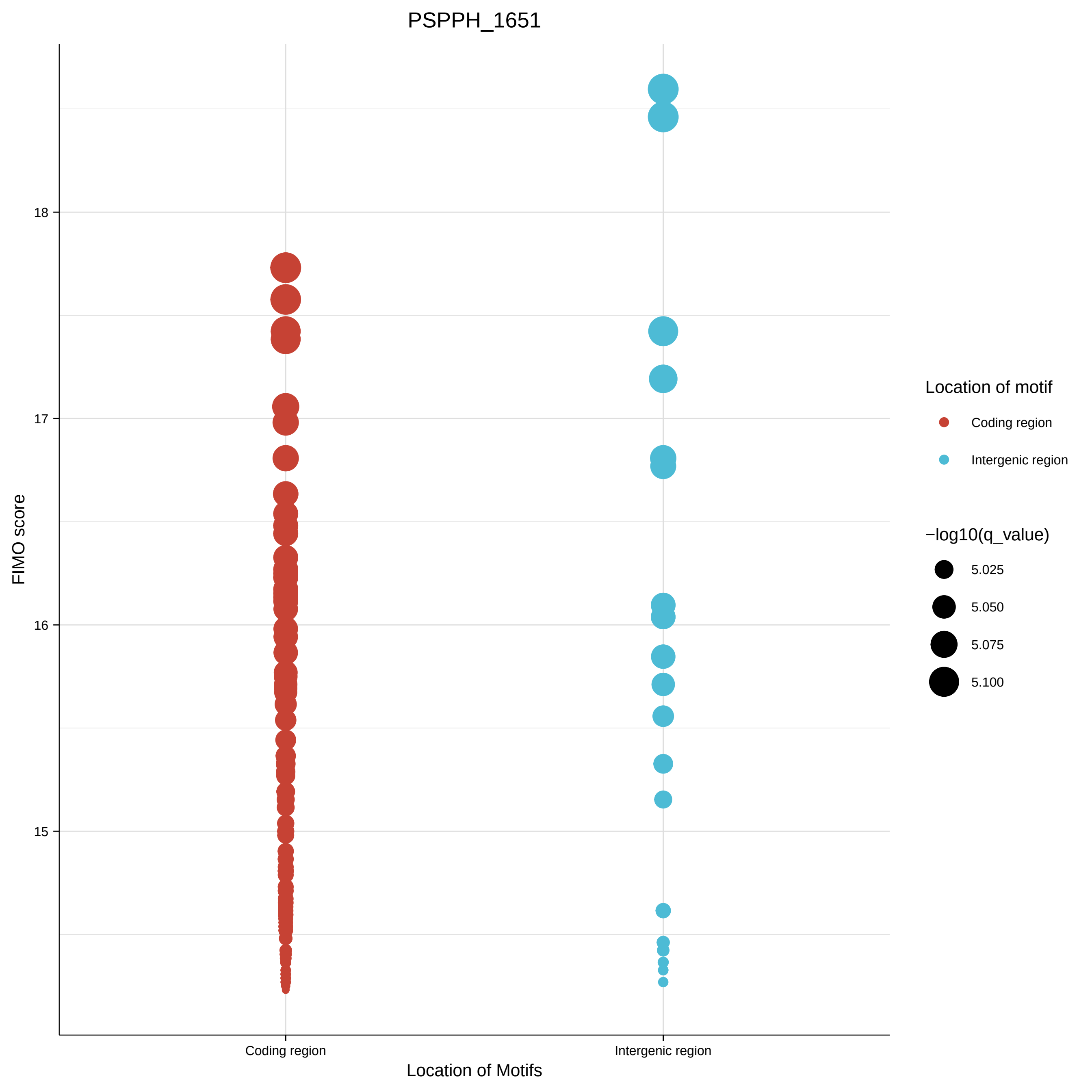
PSPPH_1651
FIMO score
18
17
16
15
Coding region
Intergenic region
Location of Motifs
Location of motif
Coding region
Intergenic region
−log10(q_value)
5.025
5.050
5.075
5.100

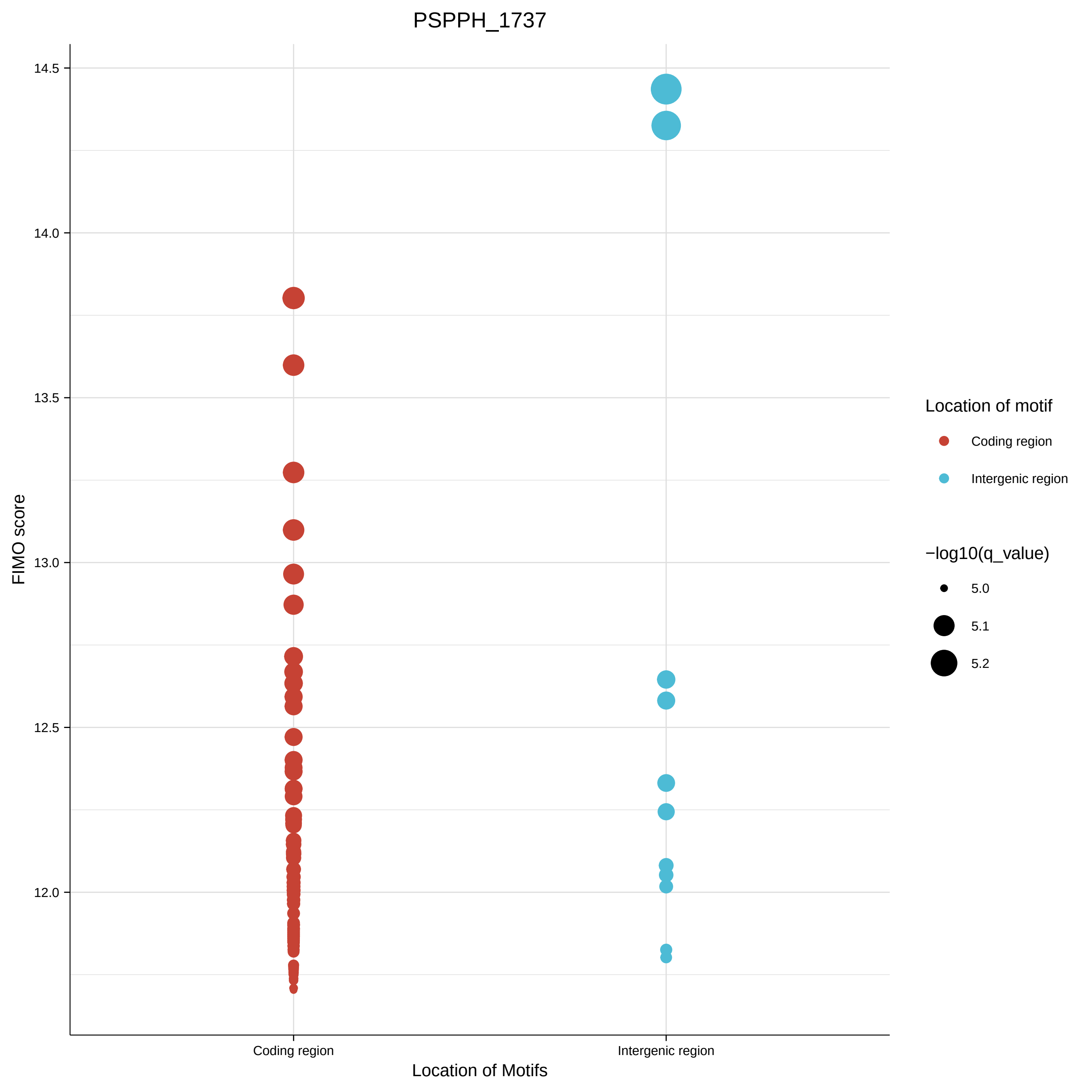

PSPPH_1737
FIMO score
14.5
14.0
13.5
13.0
12.5
12.0
Coding region
Intergenic region
Location of Motifs
Location of motif
Coding region
Intergenic region
−log10(q_value)
5.0
5.1
5.2

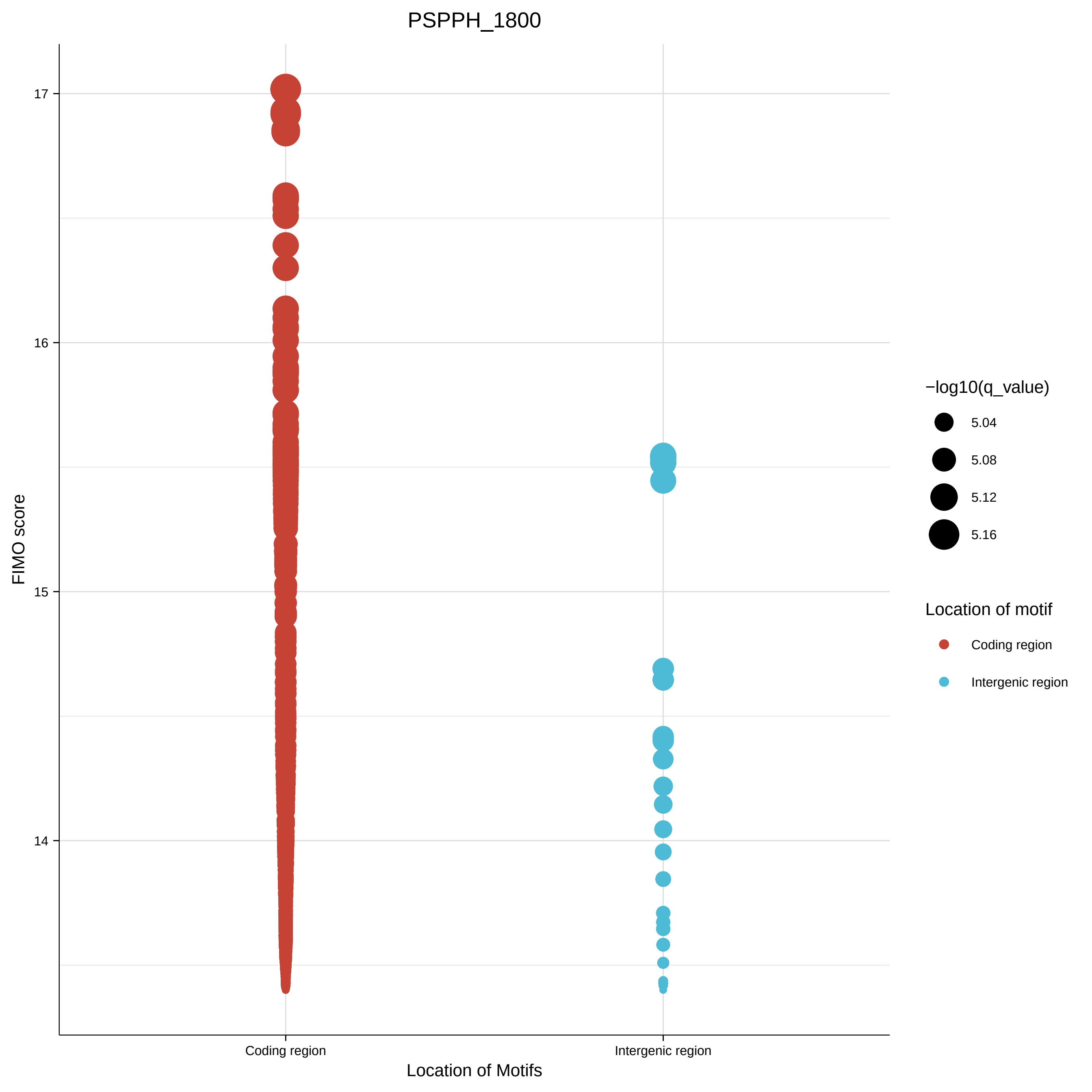

PSPPH_1800
FIMO score
17
16
15
14
Coding region
Intergenic region
Location of Motifs
−log10(q_value)
5.04
5.08
5.12
5.16
Location of motif
Coding region
Intergenic region

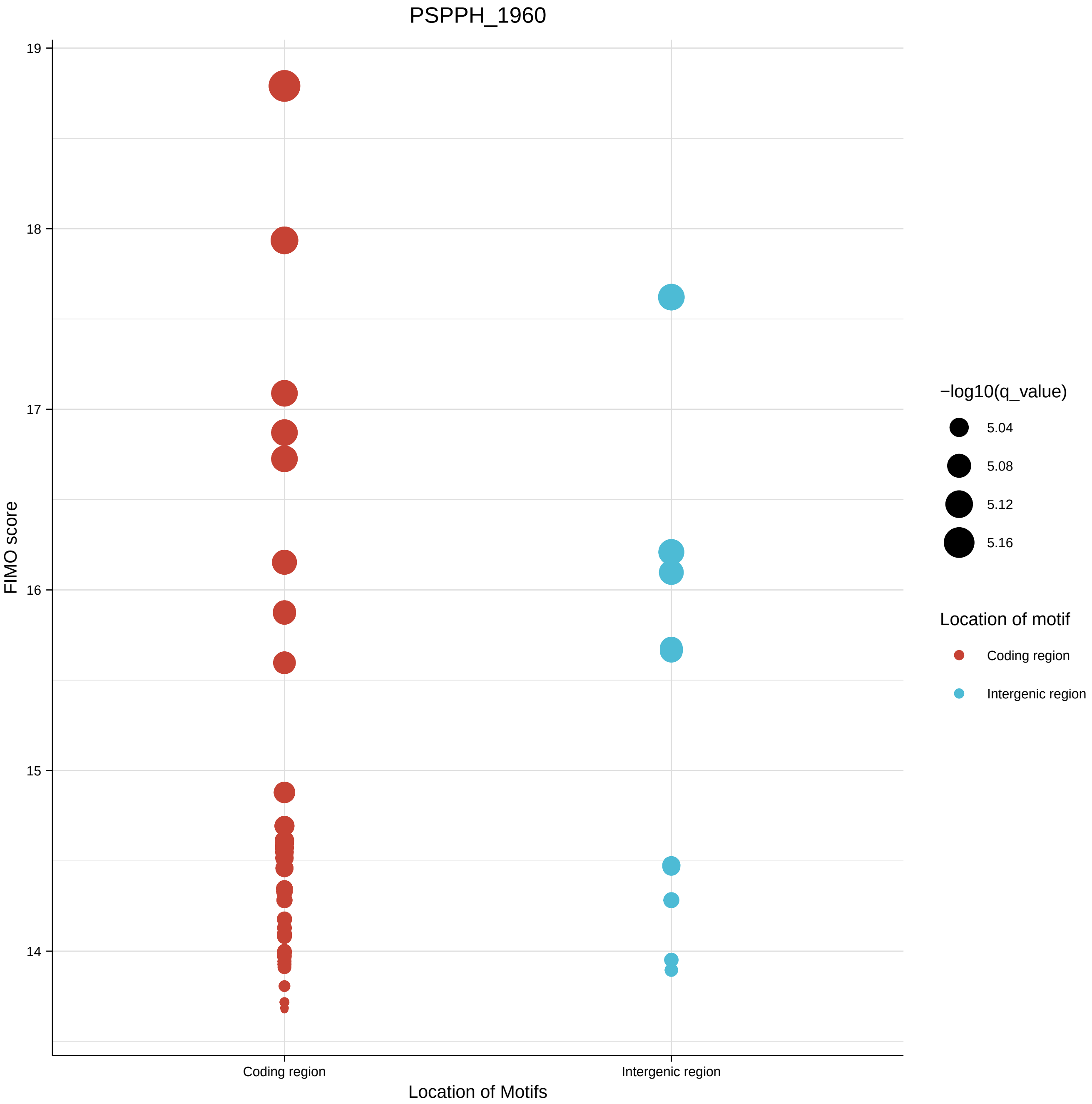
PSPPH_1960
FIMO score
19
18
17
16
15
14
Coding region
Intergenic region
Location of Motifs
−log10(q_value)
5.04
5.08
5.12
5.16
Location of motif
Coding region
Intergenic region

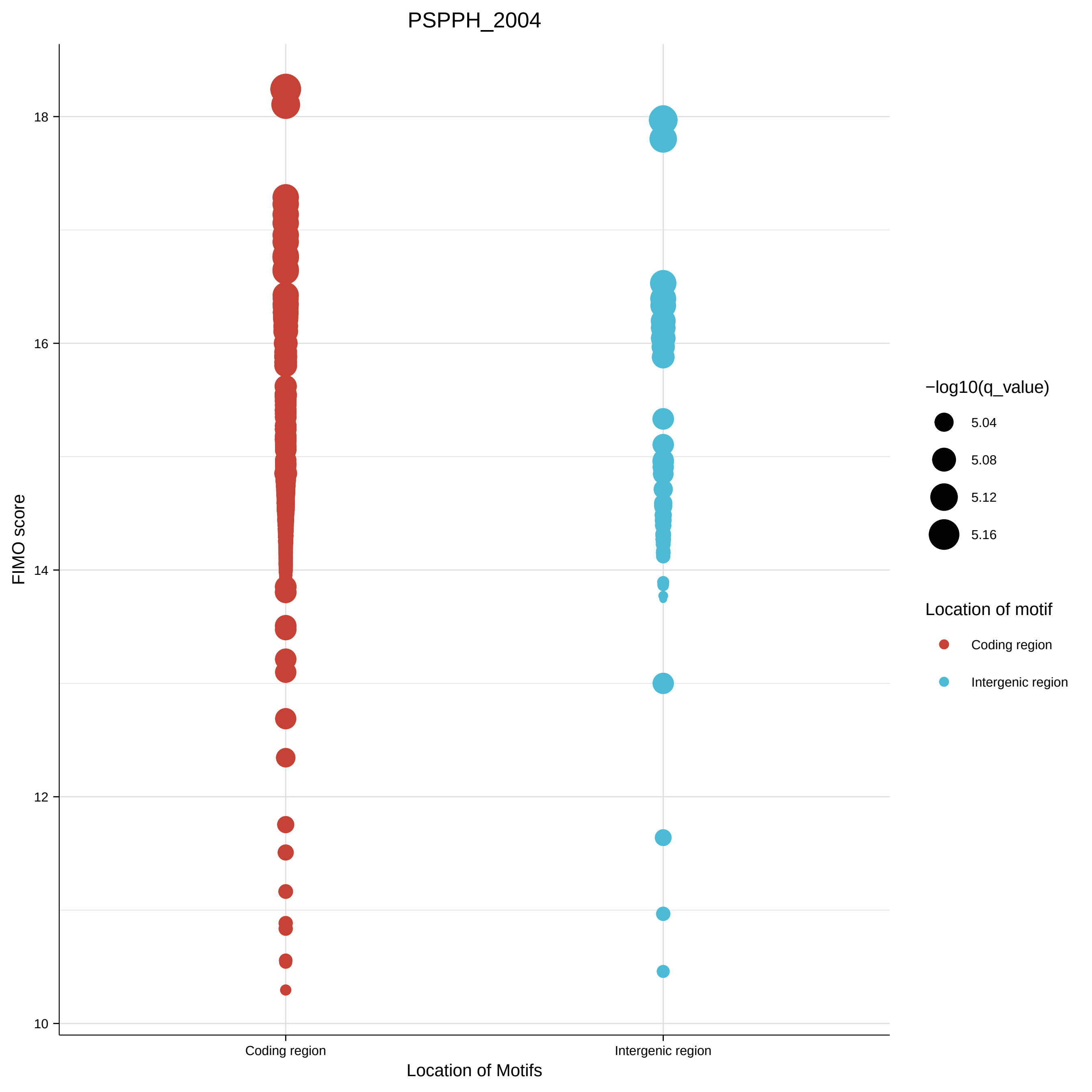
PSPPH_2004
FIMO score
18
16
14
12
10
Coding region
Intergenic region
Location of Motifs
−log10(q_value)
5.04
5.08
5.12
5.16
Location of motif
Coding region
Intergenic region

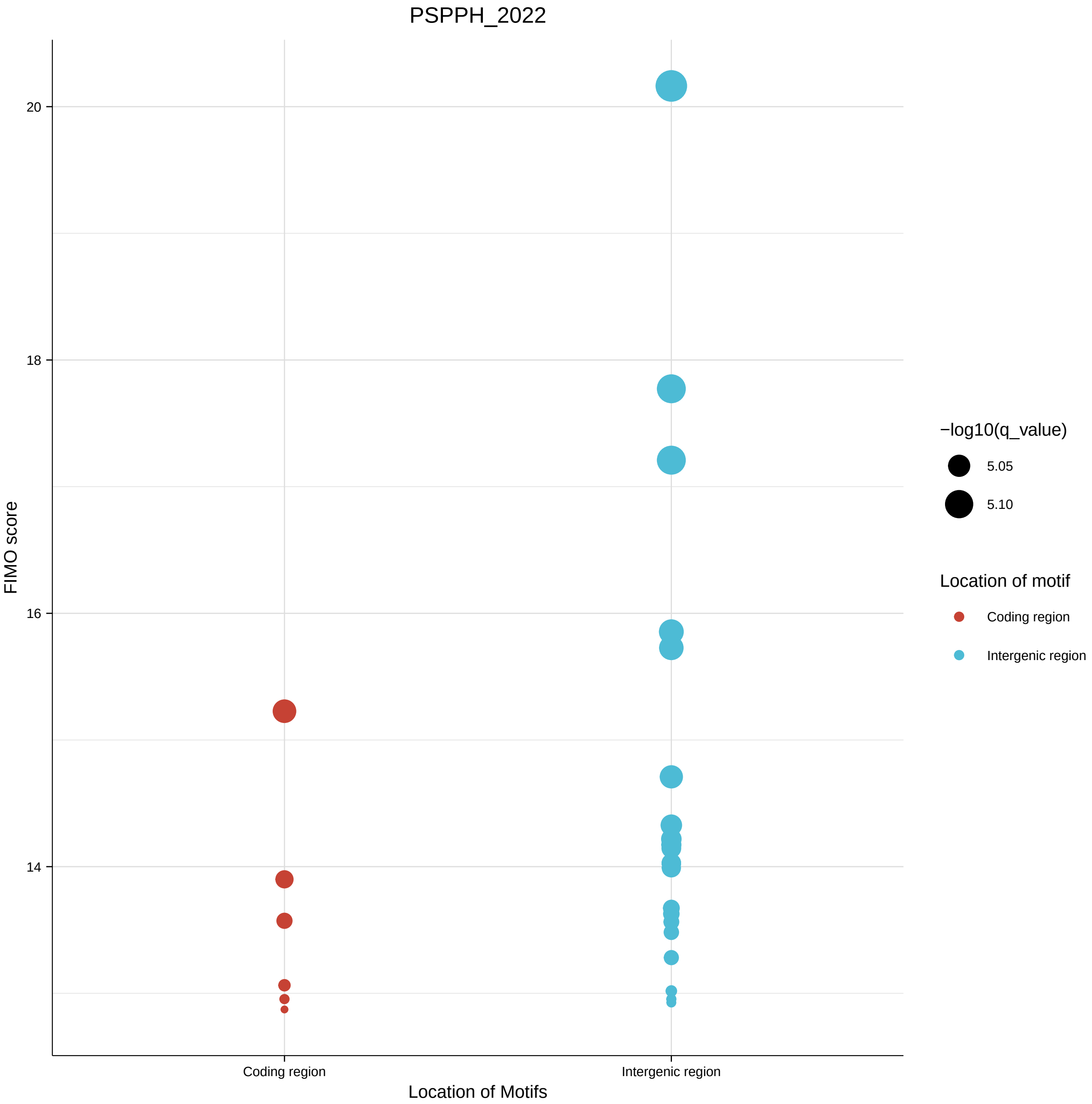

PSPPH_2022
FIMO score
20
18
16
14
Coding region
Intergenic region
Location of Motifs
−log10(q_value)
5.05
5.10
Location of motif
Coding region
Intergenic region

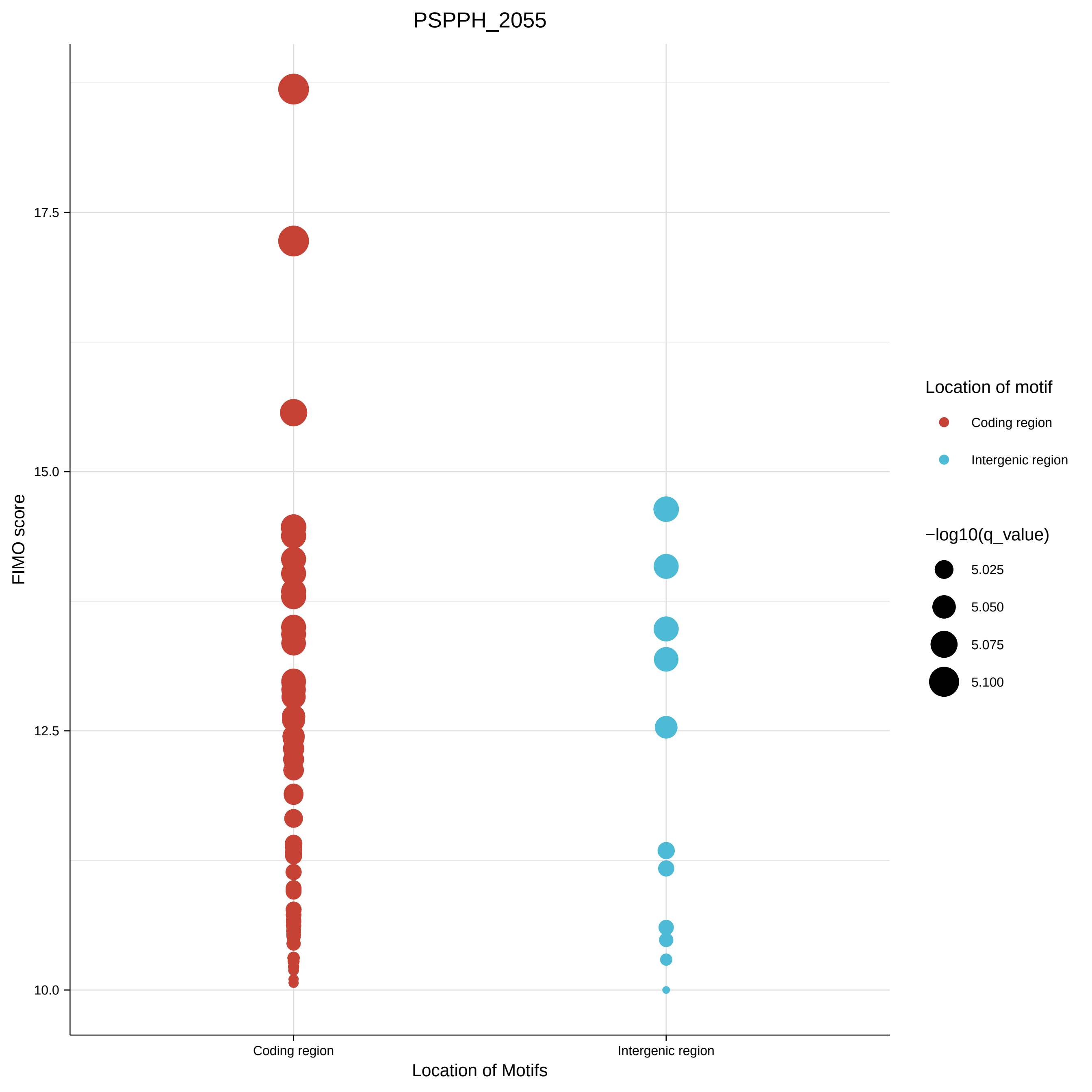
PSPPH_2055
FIMO score
17.5
15.0
12.5
10.0
Coding region
Intergenic region
Location of Motifs
Location of motif
Coding region
Intergenic region
−log10(q_value)
5.025
5.050
5.075
5.100

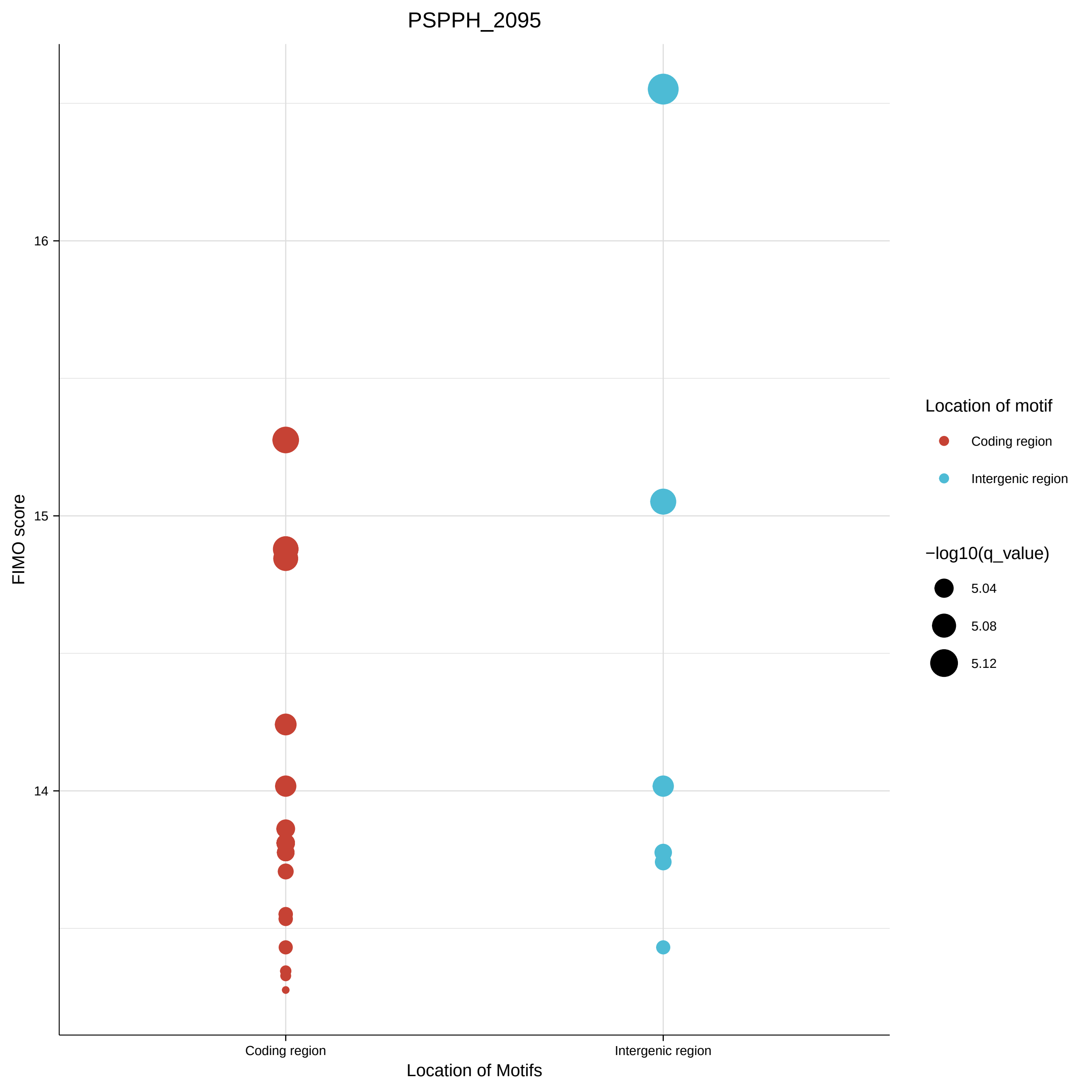
PSPPH_2095
FIMO score
16
15
14
Coding region
Intergenic region
Location of Motifs
Location of motif
Coding region
Intergenic region
−log10(q_value)
5.04
5.08
5.12

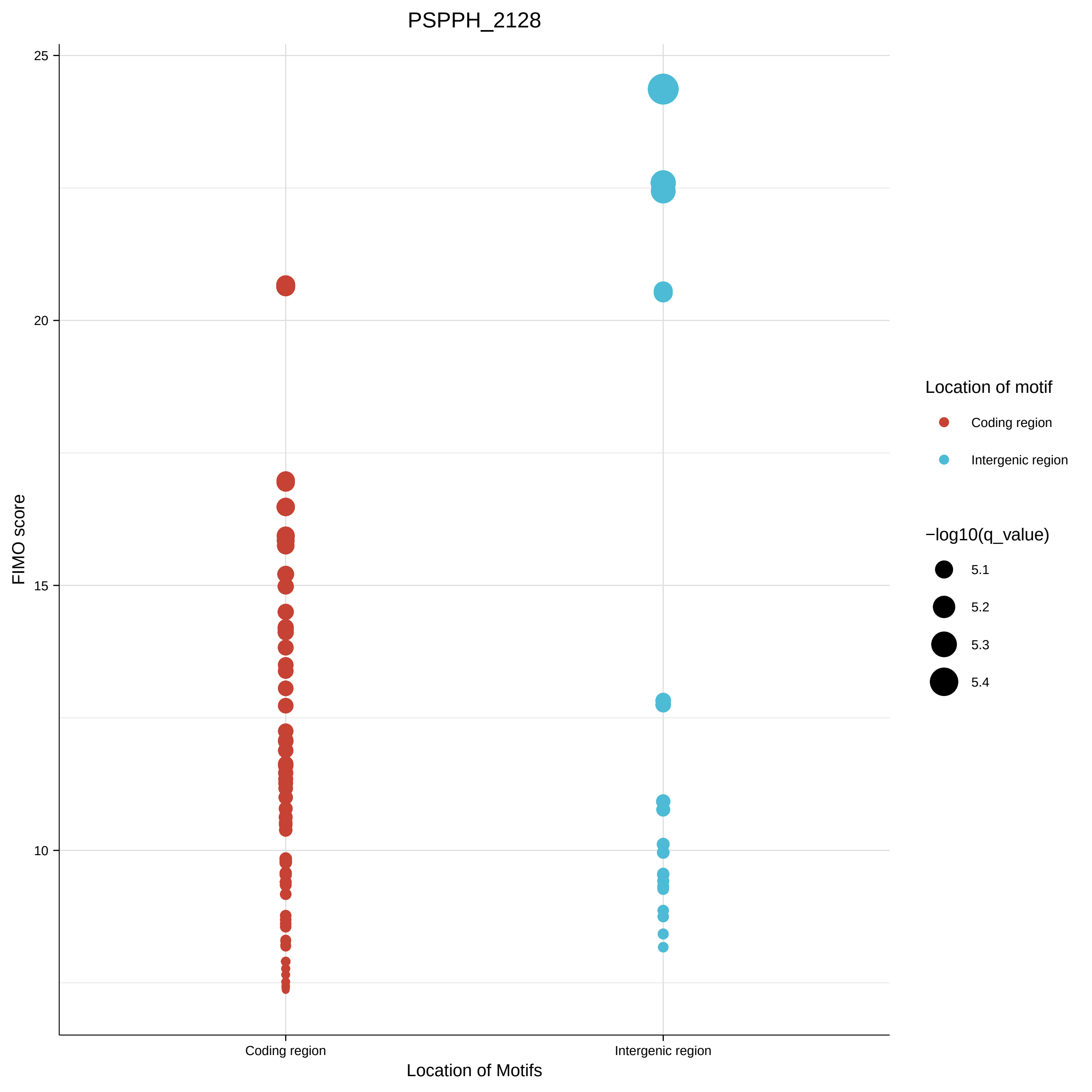

PSPPH_2128
FIMO score
25
20
15
10
Coding region
Intergenic region
Location of Motifs
Location of motif
Coding region
Intergenic region
−log10(q_value)
5.1
5.2
5.3
5.4

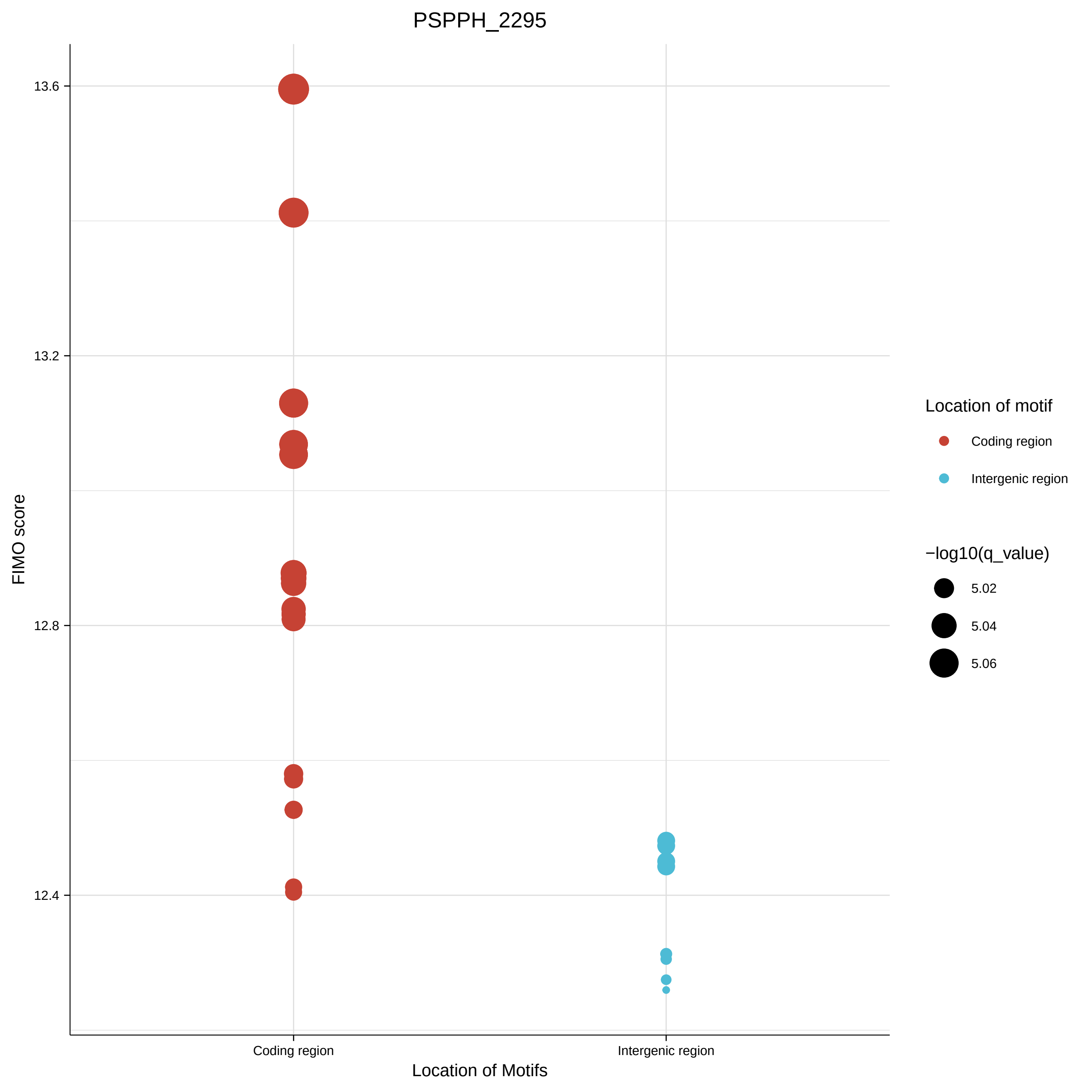
PSPPH_2295
13.6
13.2
12.8
12.4
FIMO score
Coding region
Intergenic region
Location of Motifs
Location of motif
Coding region
Intergenic region
−log10(q_value)
5.02
5.04
5.06

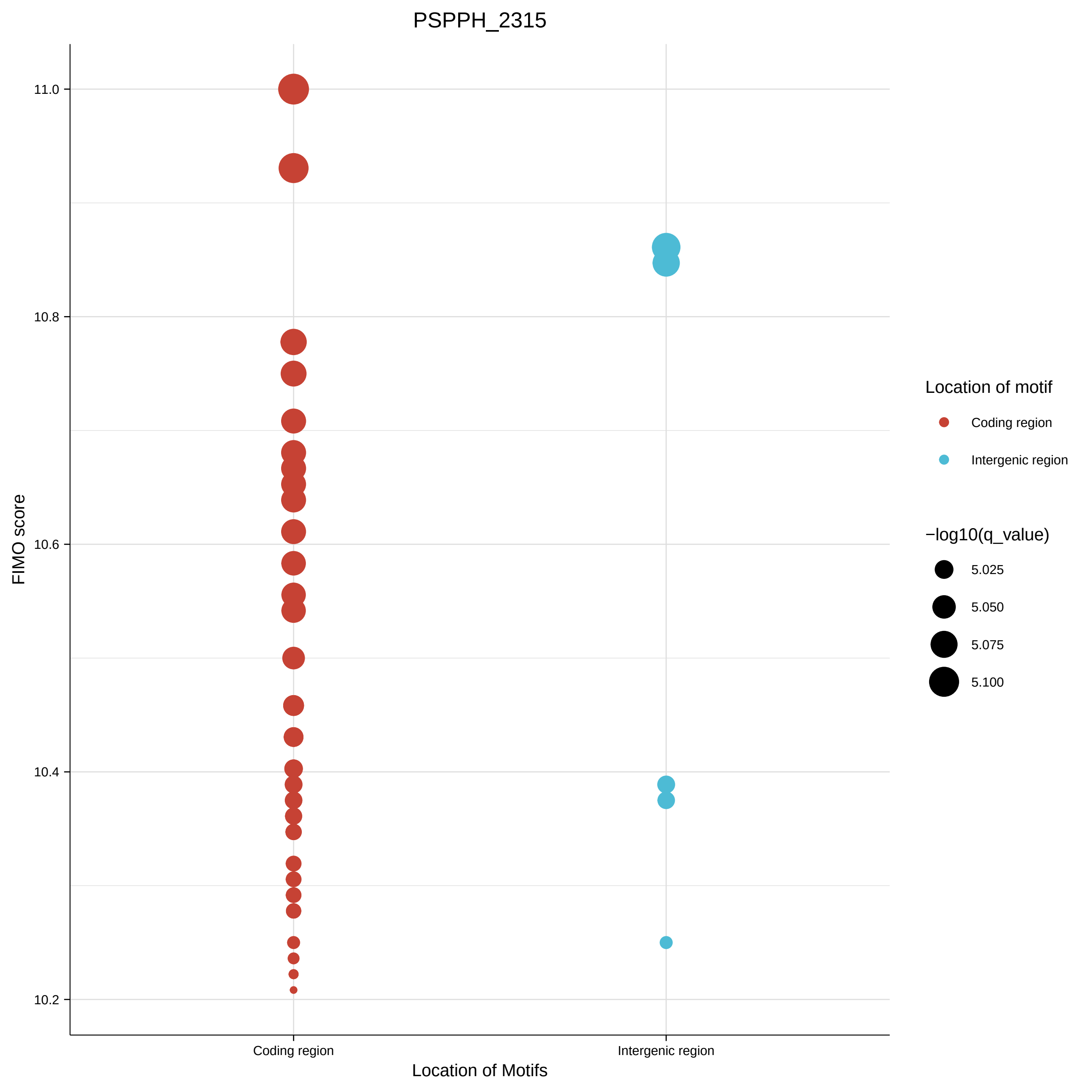

PSPPH_2315
FIMO score
11.0
10.8
10.6
10.4
10.2
Coding region
Intergenic region
Location of Motifs
Location of motif
Coding region
Intergenic region
−log10(q_value)
5.025
5.050
5.075
5.100

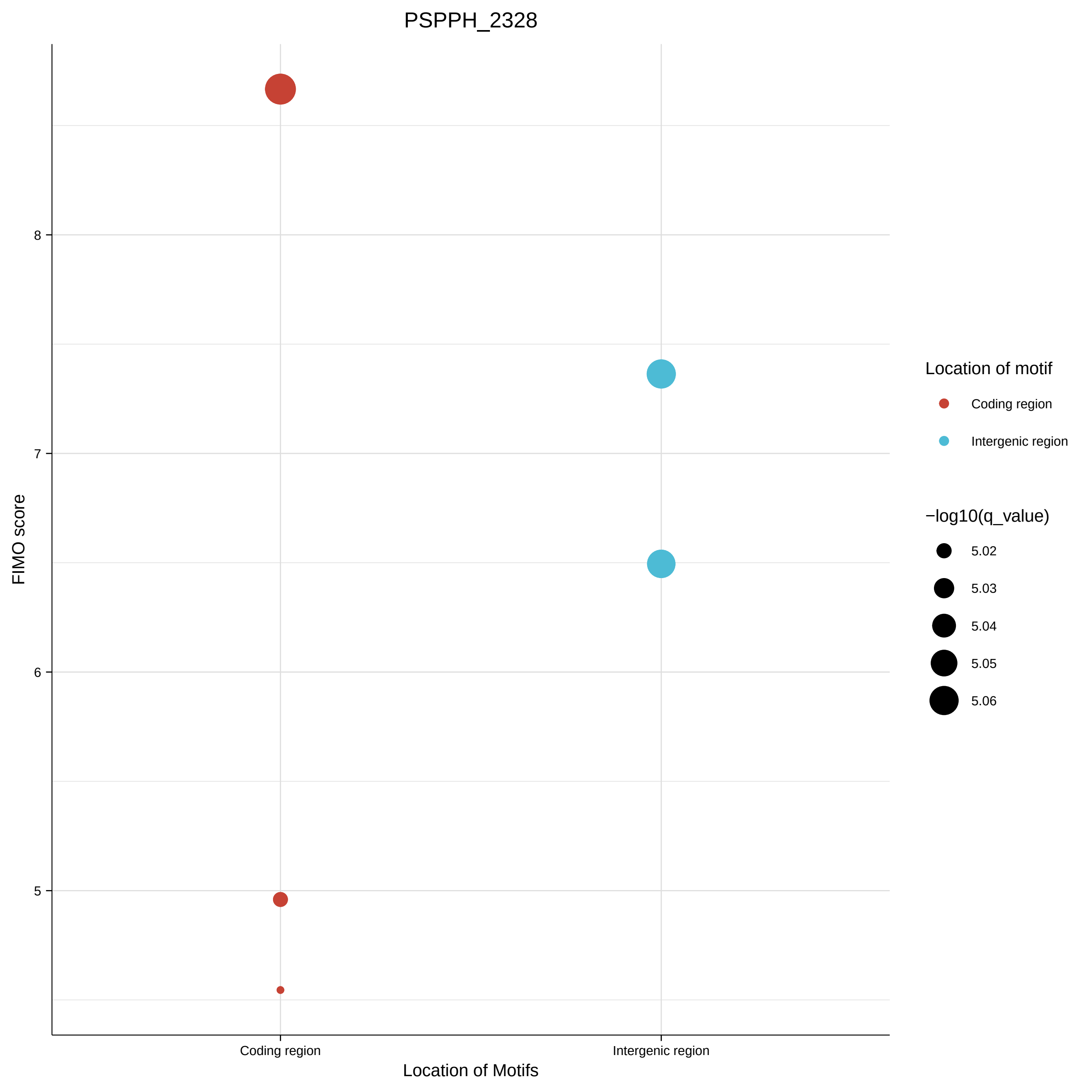
PSPPH_2328
FIMO score
8
7
6
5
Coding region
Intergenic region
Location of Motifs
Location of motif
Coding region
Intergenic region
−log10(q_value)
5.02
5.03
5.04
5.05
5.06

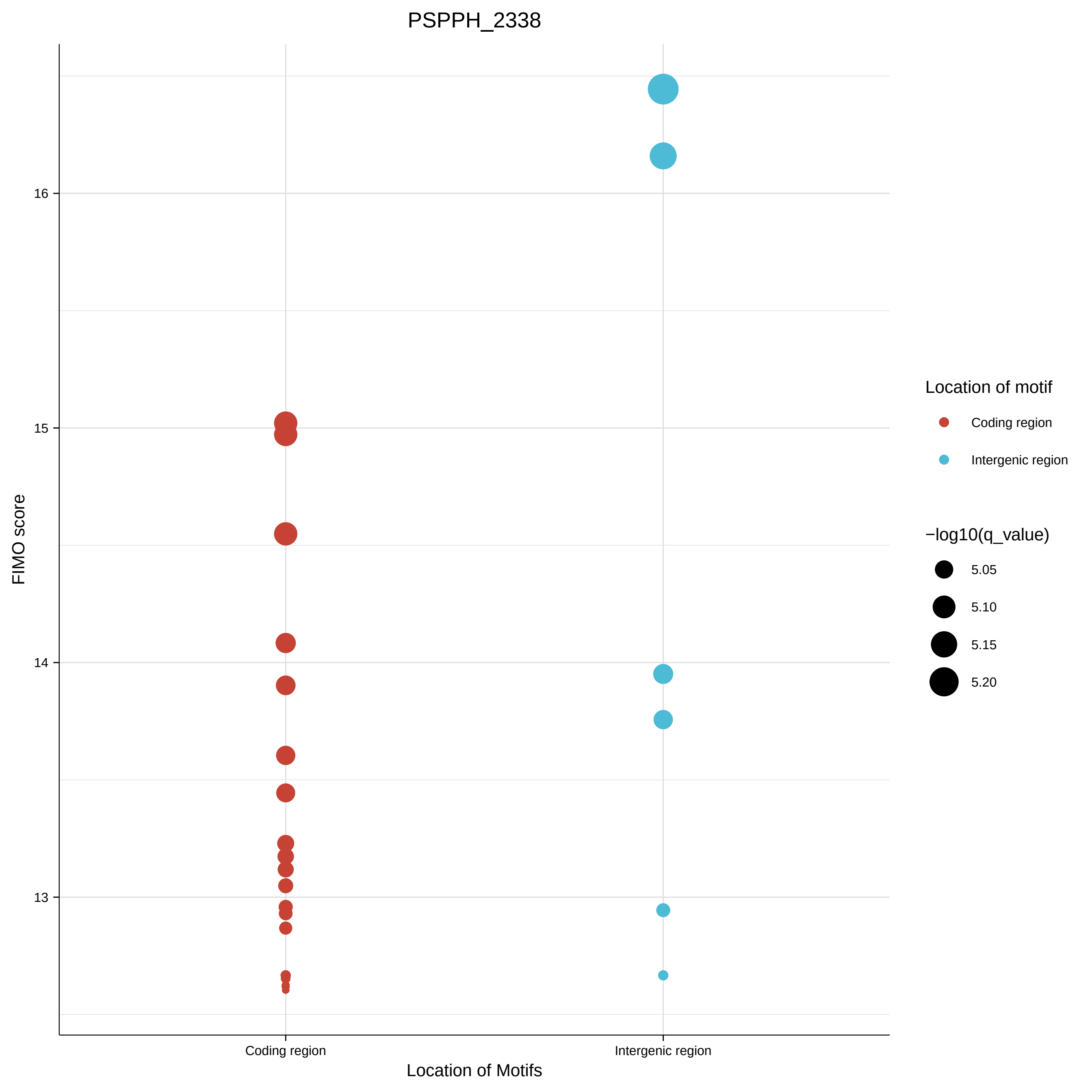

PSPPH_2338
FIMO score
16
15
14
13
Coding region
Intergenic region
Location of Motifs
Location of motif
Coding region
Intergenic region
−log10(q_value)
5.05
5.10
5.15
5.20

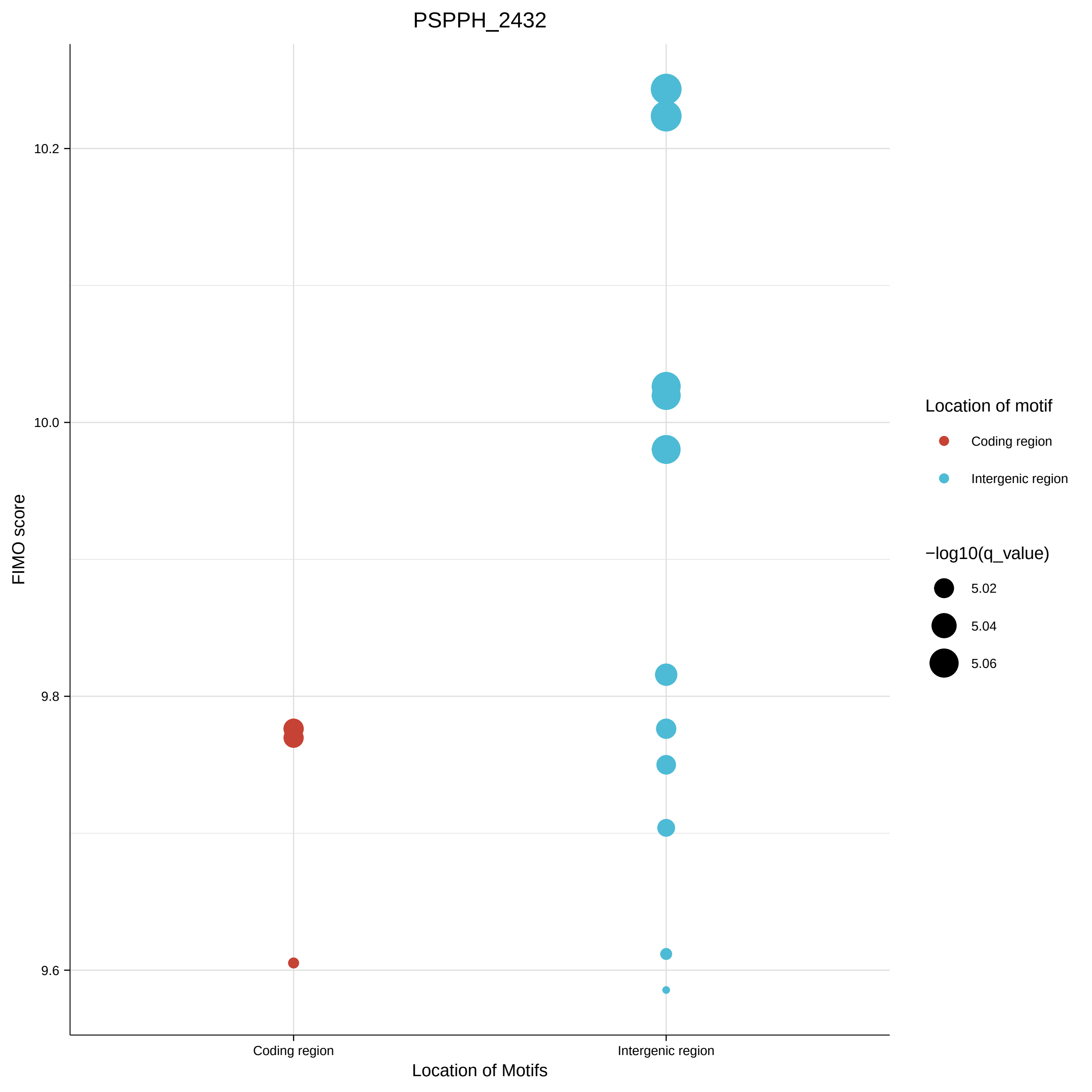

PSPPH_2432
FIMO score
10.2
10.0
9.8
9.6
Coding region
Intergenic region
Location of Motifs
Location of motif
Coding region
Intergenic region
−log10(q_value)
5.02
5.04
5.06

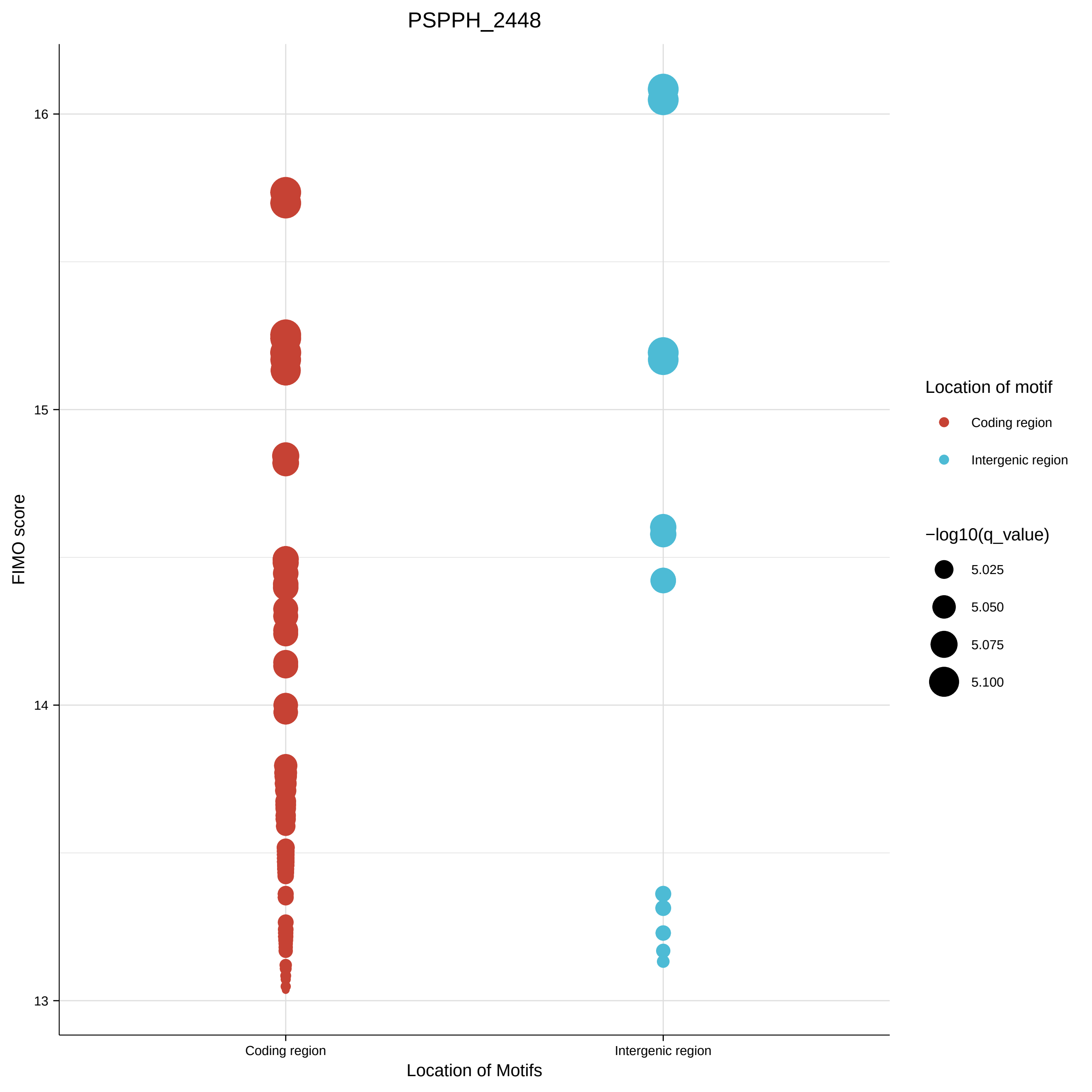
PSPPH_2448
FIMO score
16
15
14
13
Coding region
Intergenic region
Location of Motifs
Location of motif
Coding region
Intergenic region
−log10(q_value)
5.025
5.050
5.075
5.100

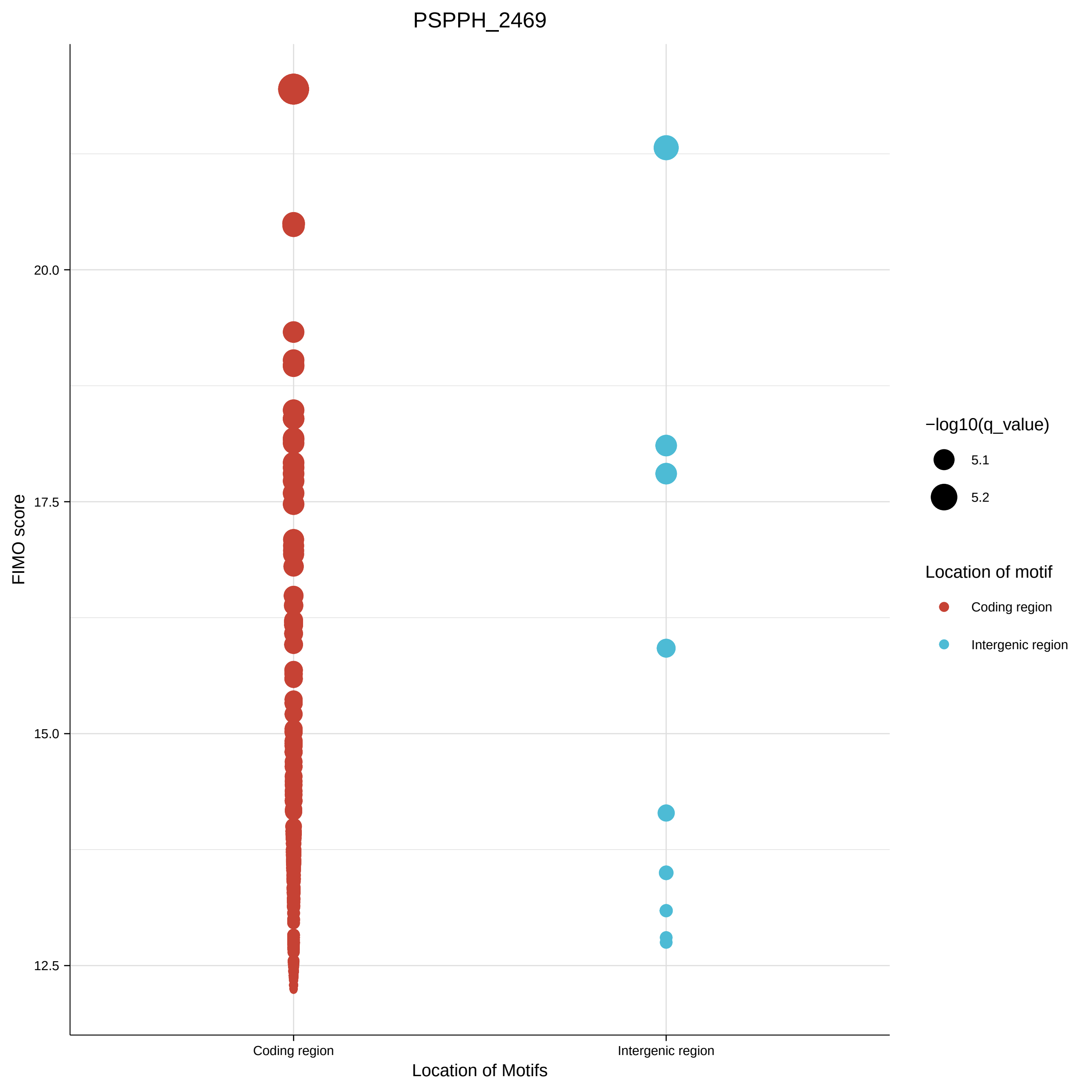

PSPPH_2469
FIMO score
20.0
17.5
15.0
12.5
Coding region
Intergenic region
Location of Motifs
−log10(q_value)
5.1
5.2
Location of motif
Coding region
Intergenic region

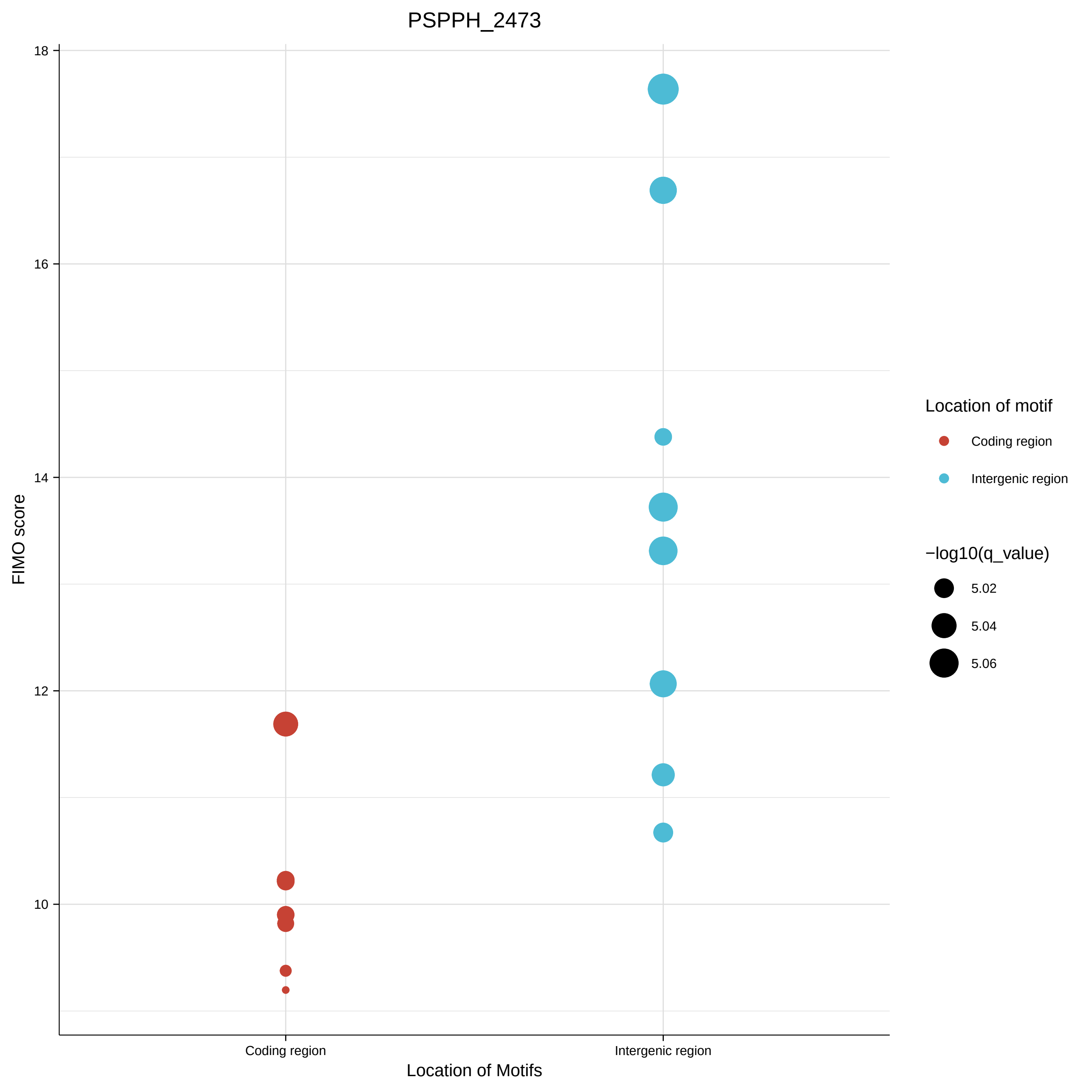

PSPPH_2473
FIMO score
18
16
14
12
10
Coding region
Intergenic region
Location of Motifs
Location of motif
Coding region
Intergenic region
−log10(q_value)
5.02
5.04
5.06

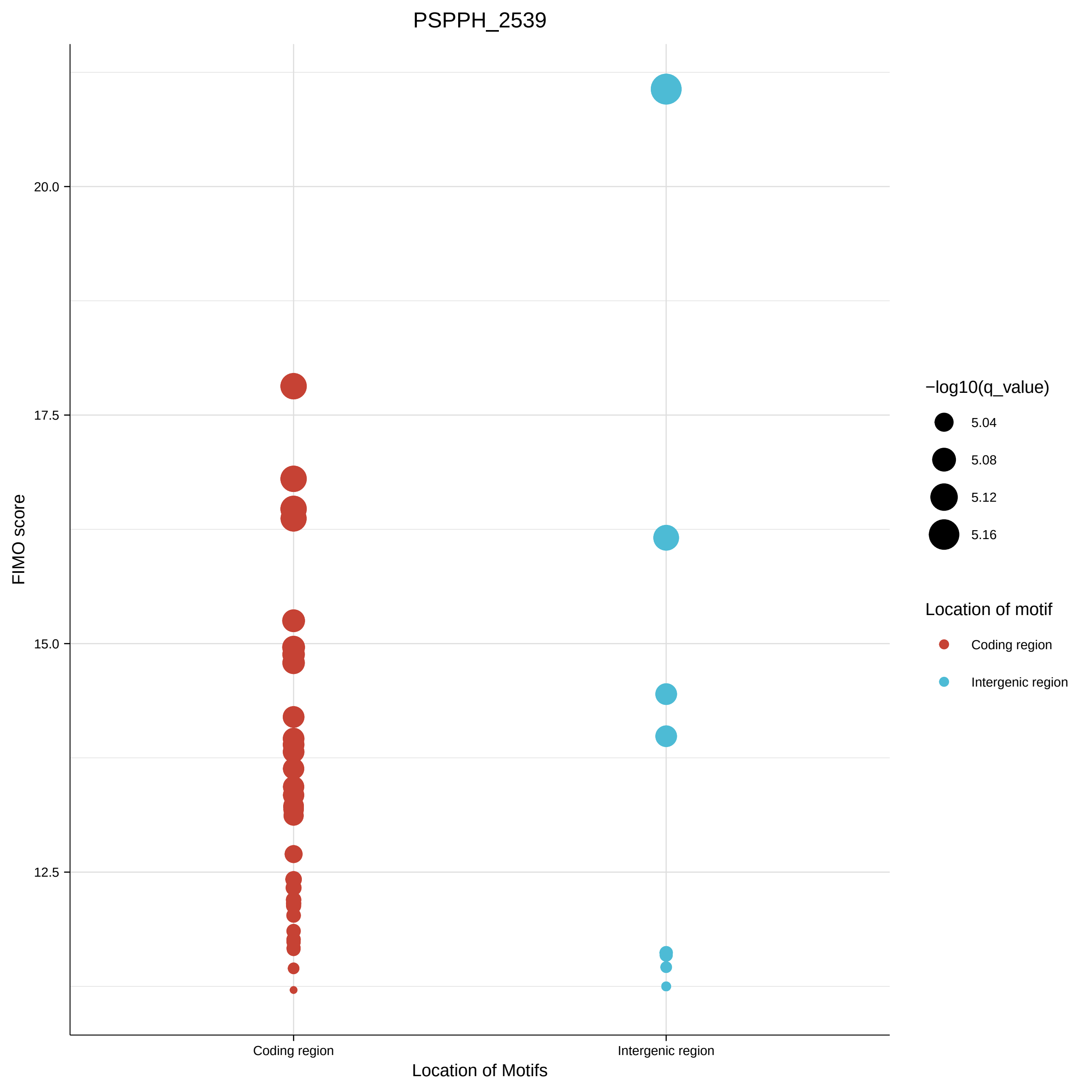
PSPPH_2539
FIMO score
20.0
17.5
15.0
12.5
Coding region
Intergenic region
Location of Motifs
−log10(q_value)
5.04
5.08
5.12
5.16
Location of motif
Coding region
Intergenic region

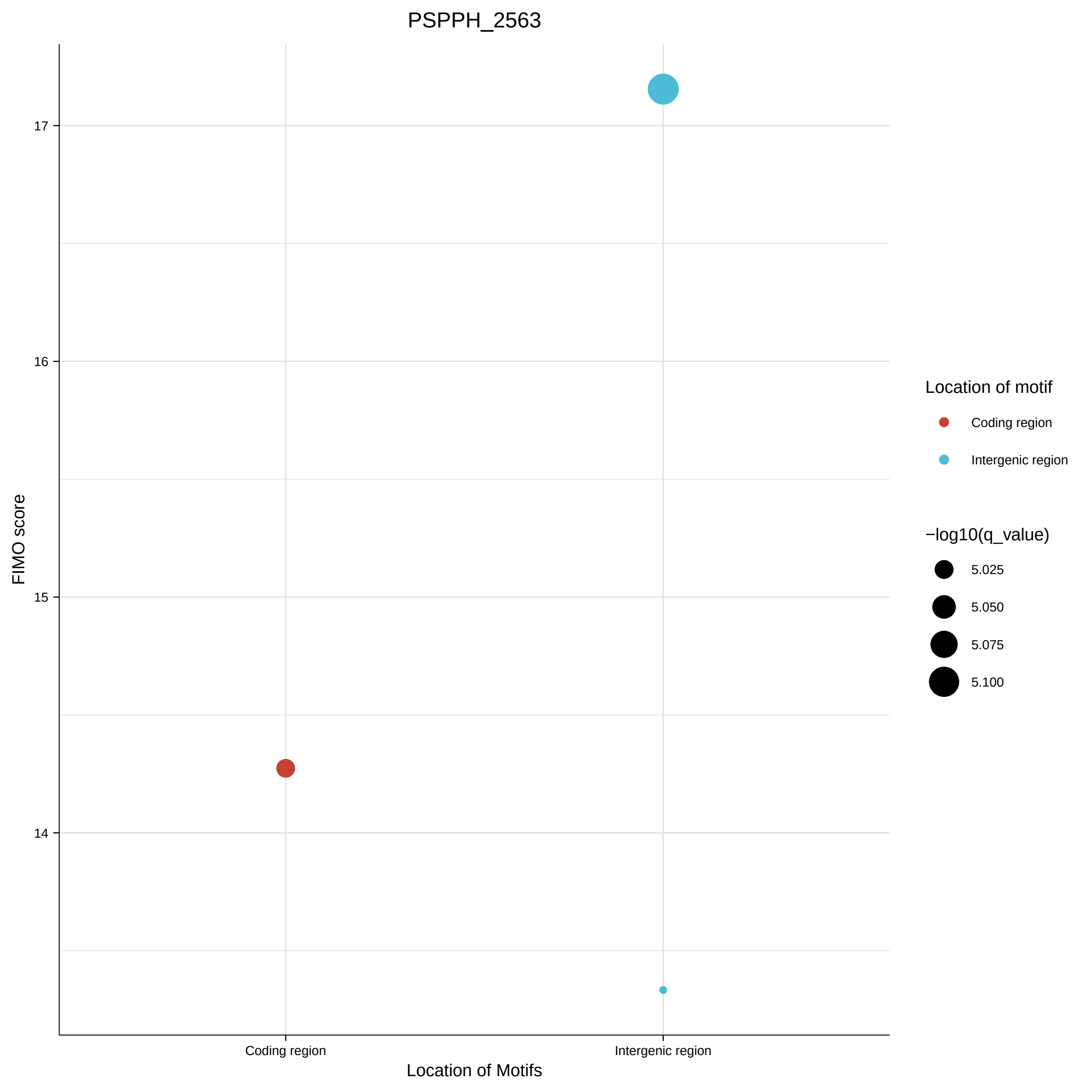
PSPPH_2563
FIMO score
17
16
15
14
Coding region
Intergenic region
Location of Motifs
Location of motif
Coding region
Intergenic region
−log10(q_value)
5.025
5.050
5.075
5.100

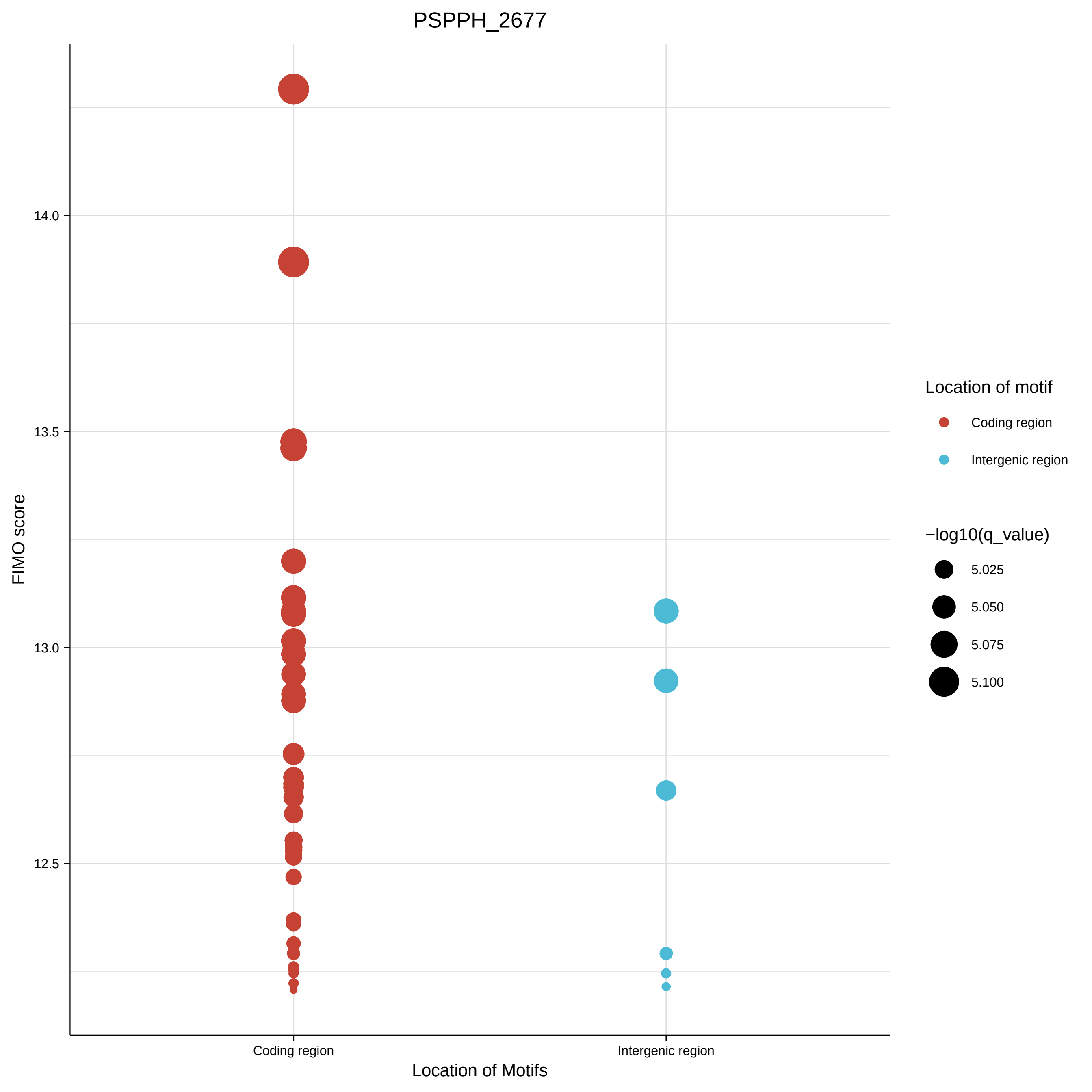

PSPPH_2677
FIMO score
14.0
13.5
13.0
12.5
Coding region
Intergenic region
Location of Motifs
Location of motif
Coding region
Intergenic region
−log10(q_value)
5.025
5.050
5.075
5.100

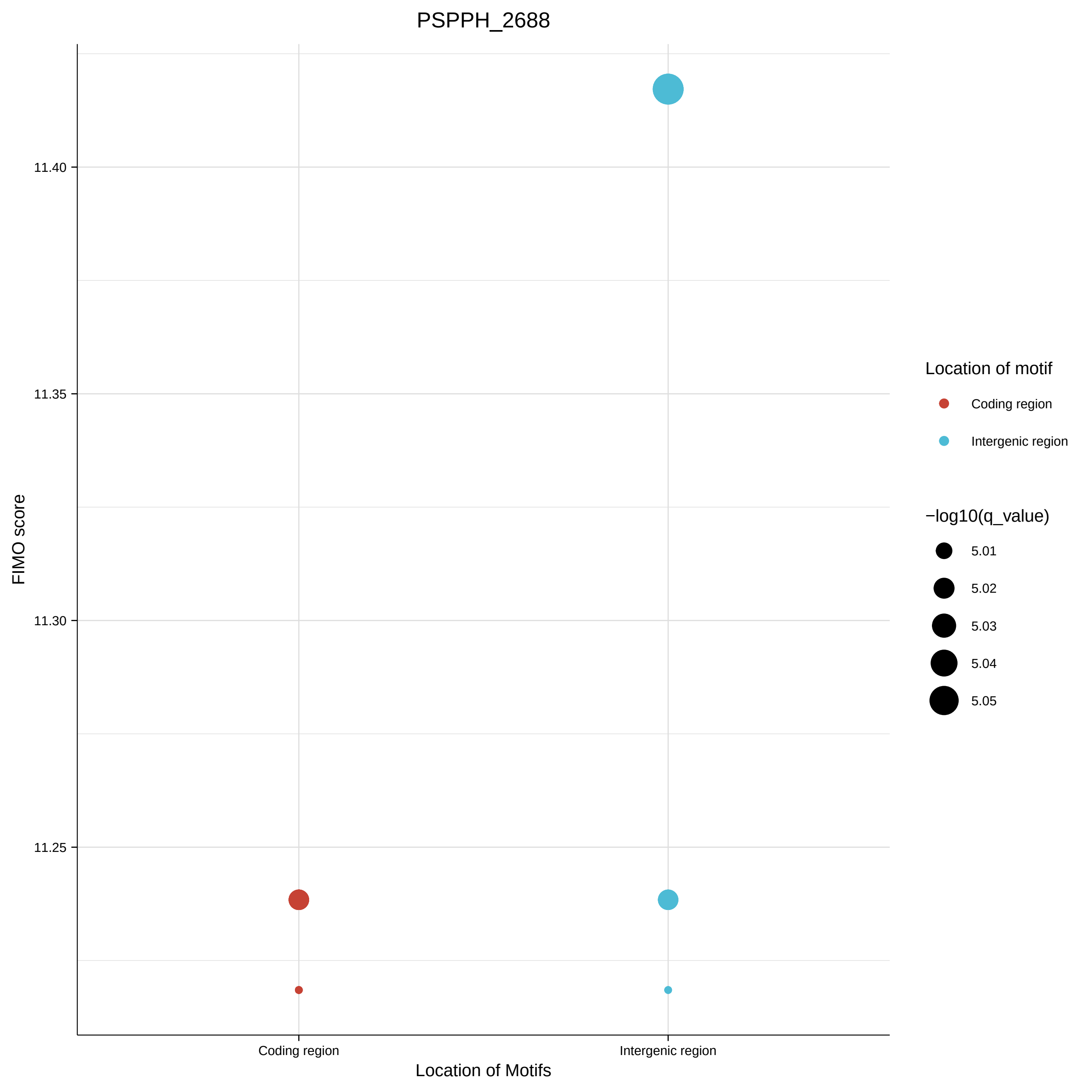

PSPPH_2688
FIMO score
11.40
11.35
11.30
11.25
Coding region
Intergenic region
Location of Motifs
Location of motif
Coding region
Intergenic region
−log10(q_value)
5.01
5.02
5.03
5.04
5.05

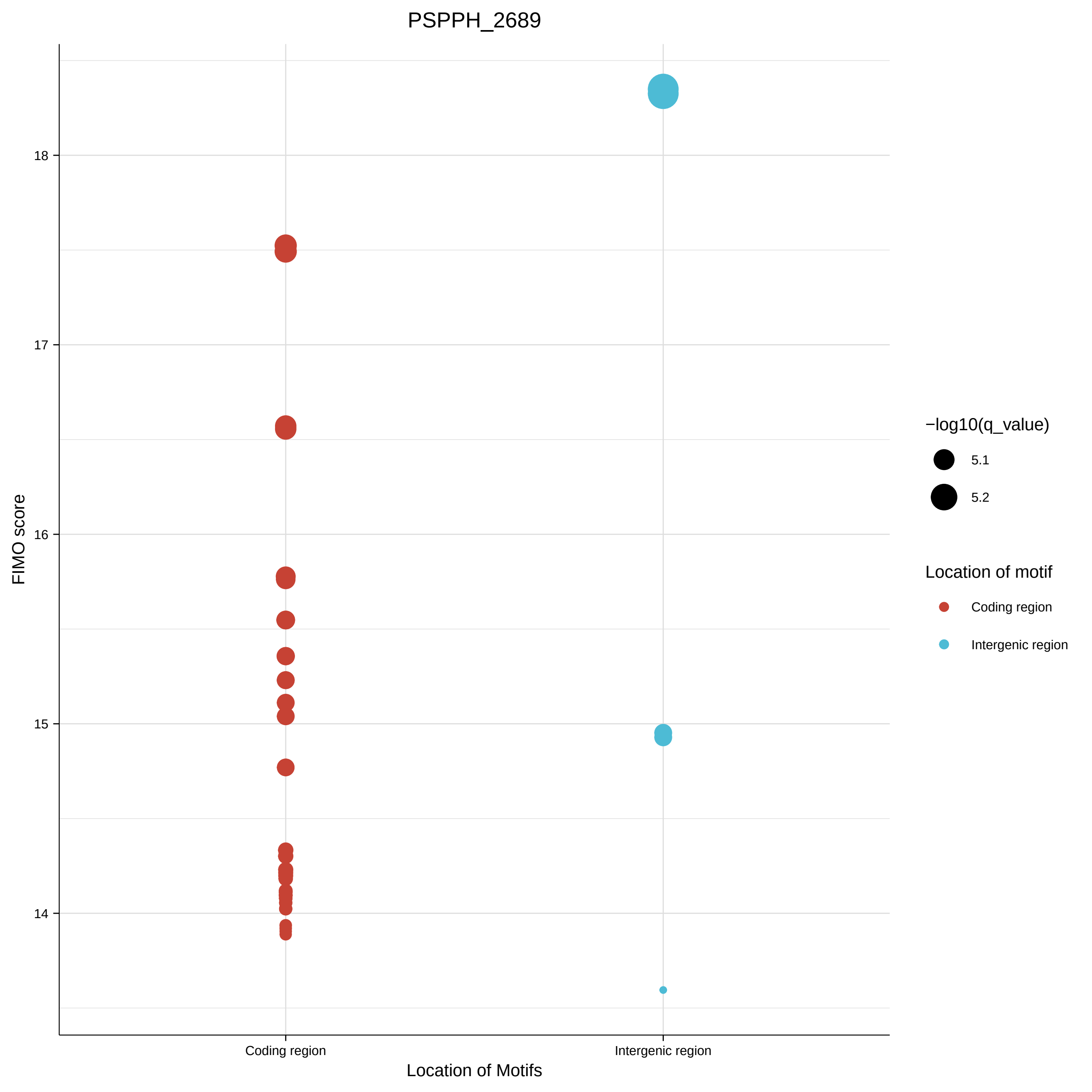

PSPPH_2689
FIMO score
18
17
16
15
14
Coding region
Intergenic region
Location of Motifs
−log10(q_value)
5.1
5.2
Location of motif
Coding region
Intergenic region

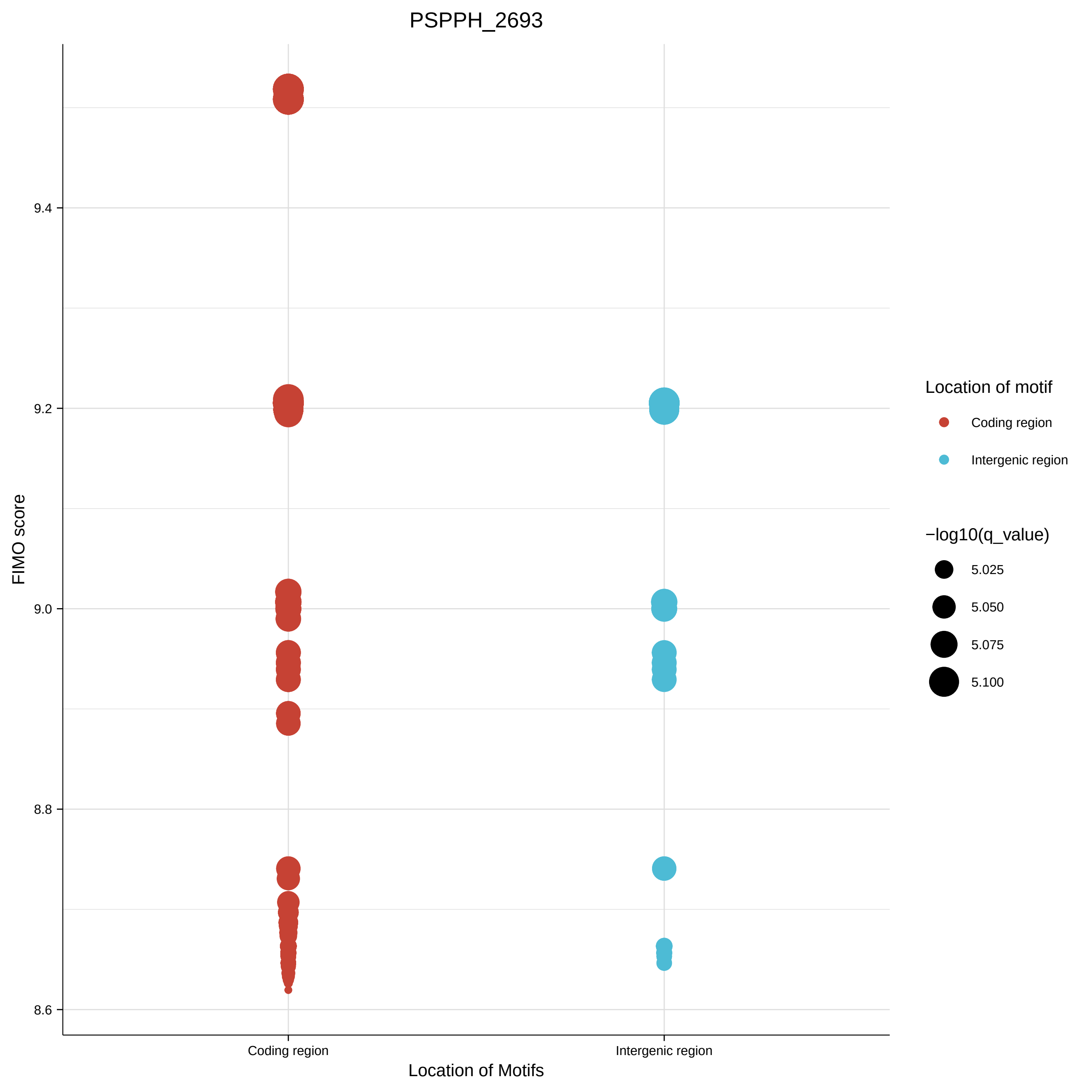

PSPPH_2693
FIMO score
9.4
9.2
9.0
8.8
8.6
Coding region
Intergenic region
Location of Motifs
Location of motif
Coding region
Intergenic region
−log10(q_value)
5.025
5.050
5.075
5.100

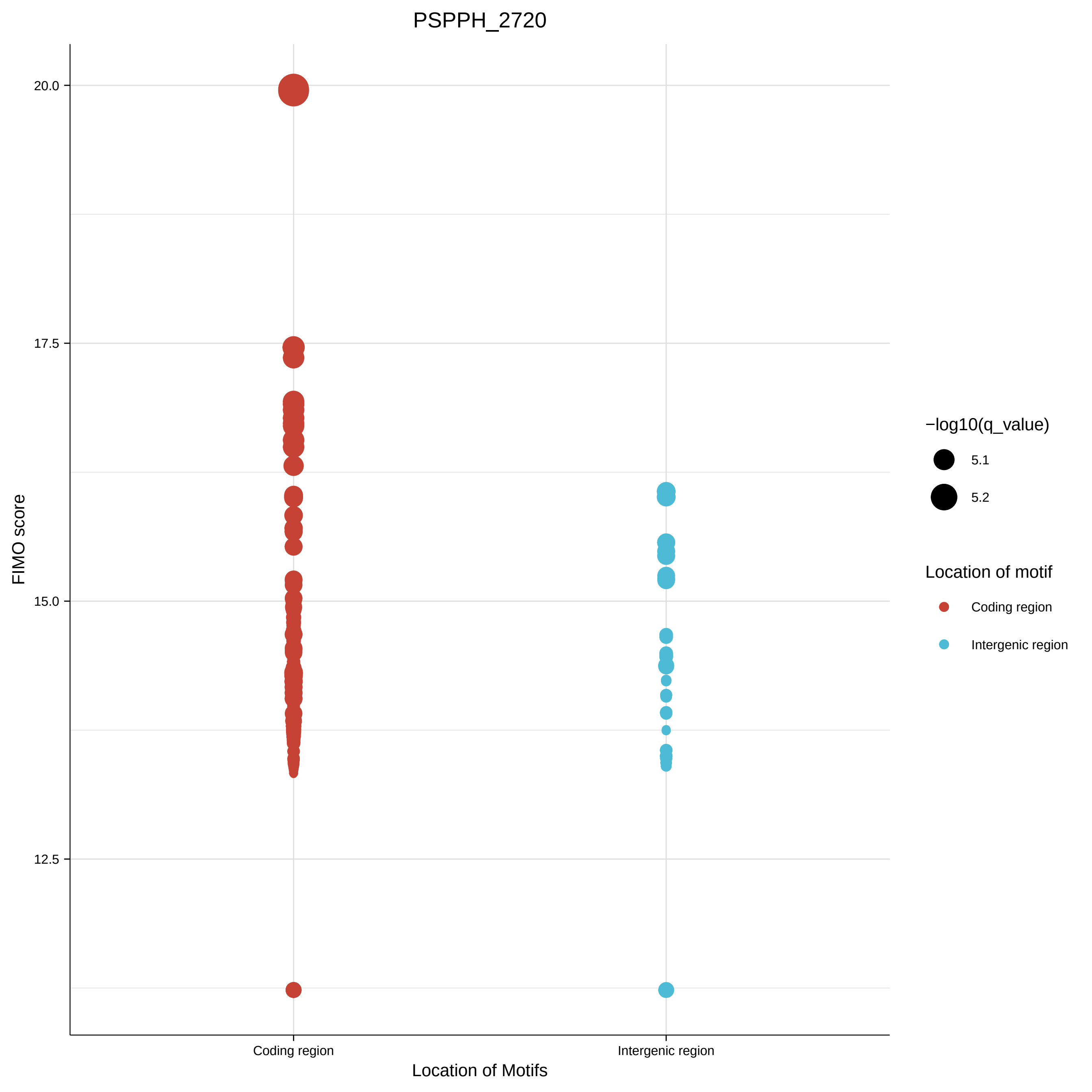
PSPPH_2720
FIMO score
20.0
17.5
15.0
12.5
Coding region
Intergenic region
Location of Motifs
−log10(q_value)
5.1
5.2
Location of motif
Coding region
Intergenic region

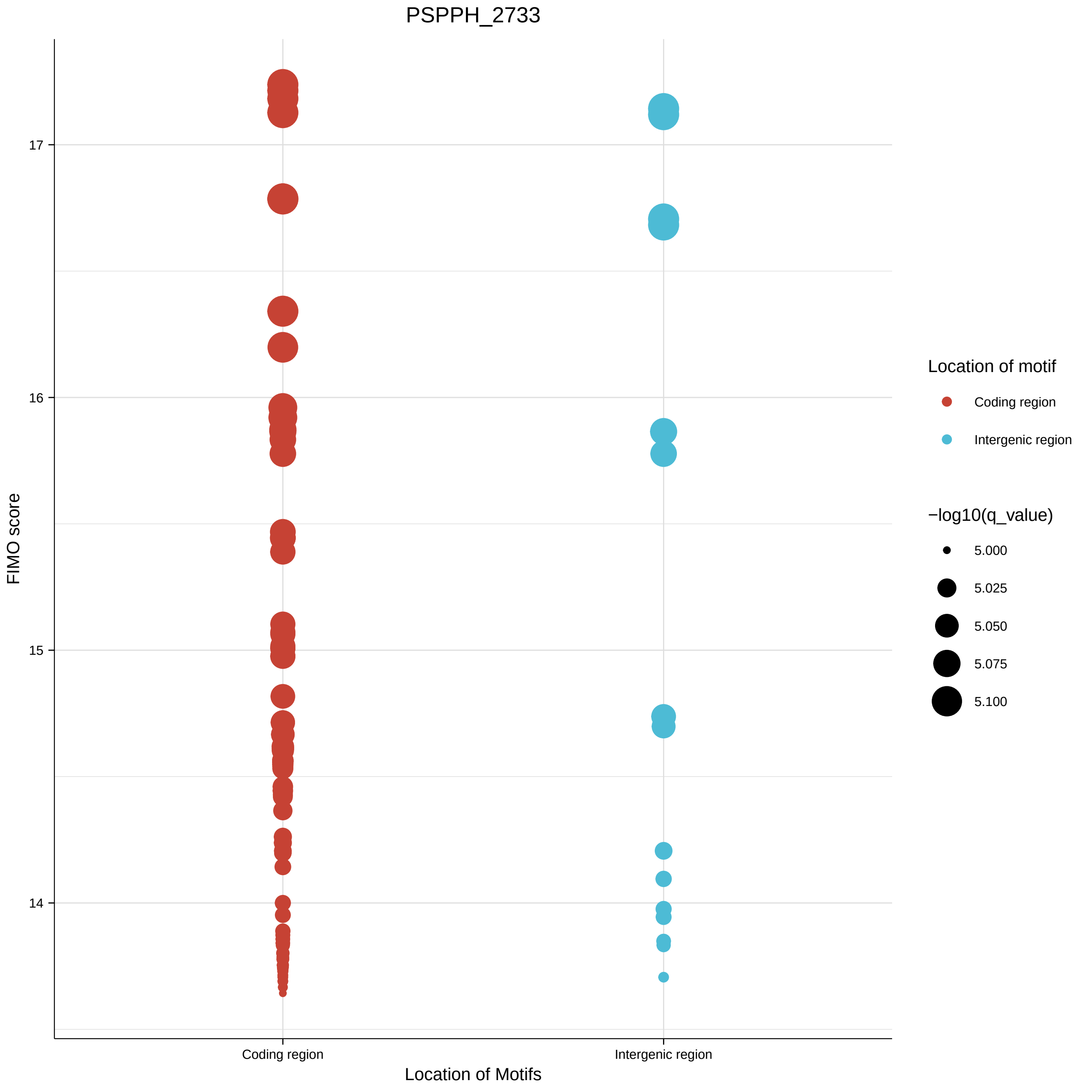

PSPPH_2733
FIMO score
17
16
15
14
Coding region
Intergenic region
Location of Motifs
Location of motif
Coding region
Intergenic region
−log10(q_value)
5.000
5.025
5.050
5.075
5.100

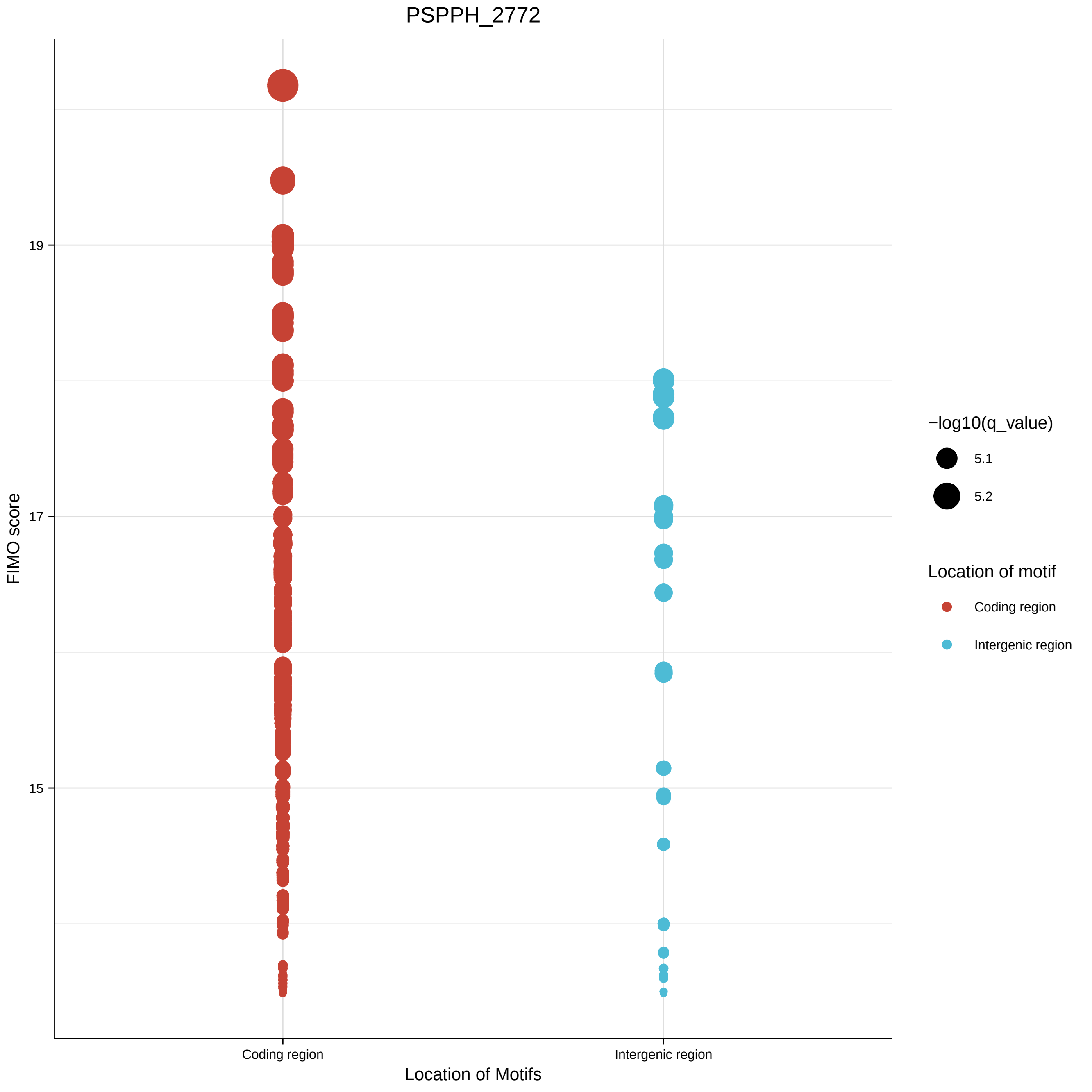
PSPPH_2772
FIMO score
19
17
15
Coding region
Intergenic region
Location of Motifs
−log10(q_value)
5.1
5.2
Location of motif
Coding region
Intergenic region

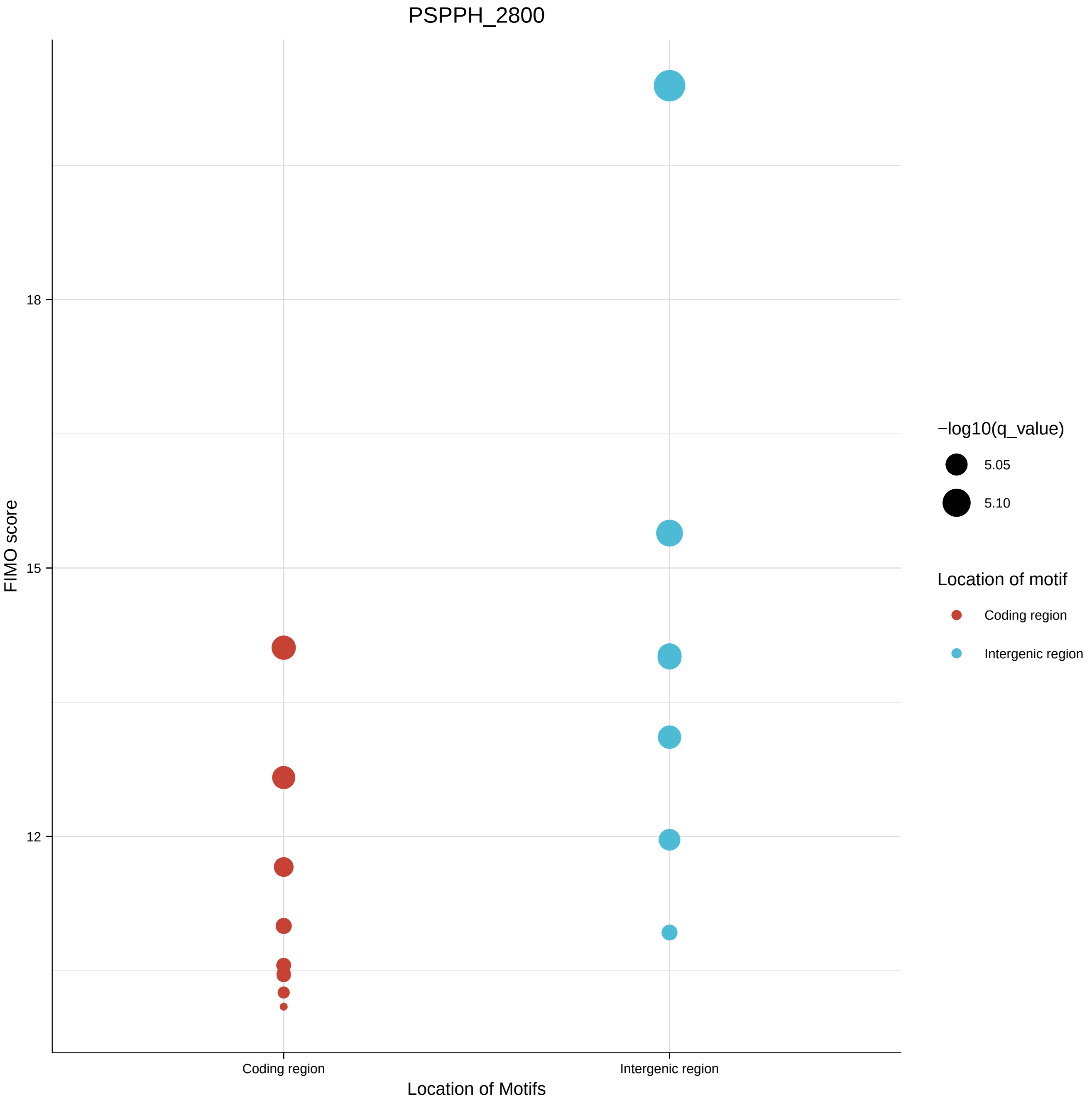

PSPPH_2800
FIMO score
18
15
12
Coding region
Intergenic region
Location of Motifs
−log10(q_value)
5.05
5.10
Location of motif
Coding region
Intergenic region

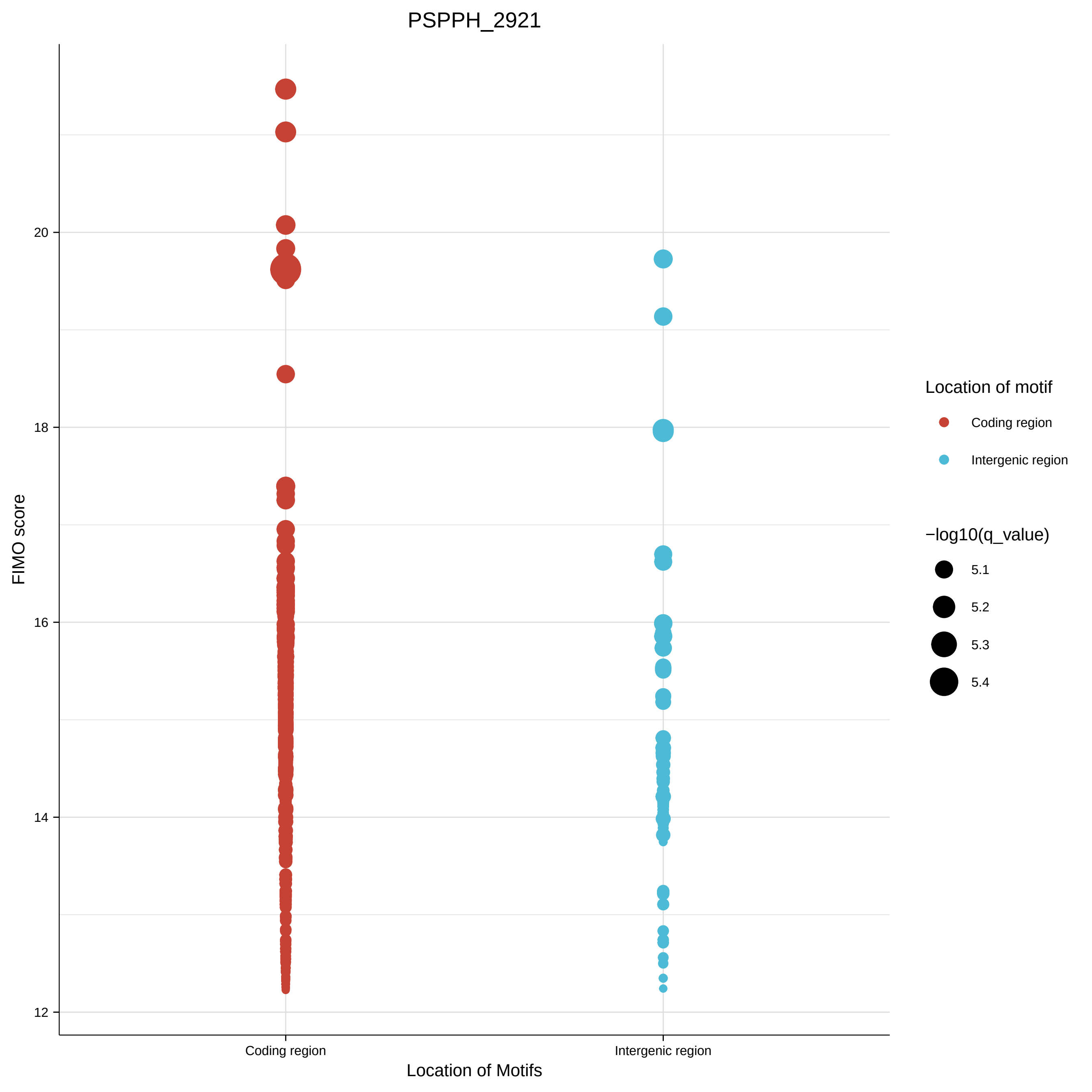
PSPPH_2921
FIMO score
20
18
16
14
12
Coding region
Intergenic region
Location of Motifs
Location of motif
Coding region
Intergenic region
−log10(q_value)
5.1
5.2
5.3
5.4

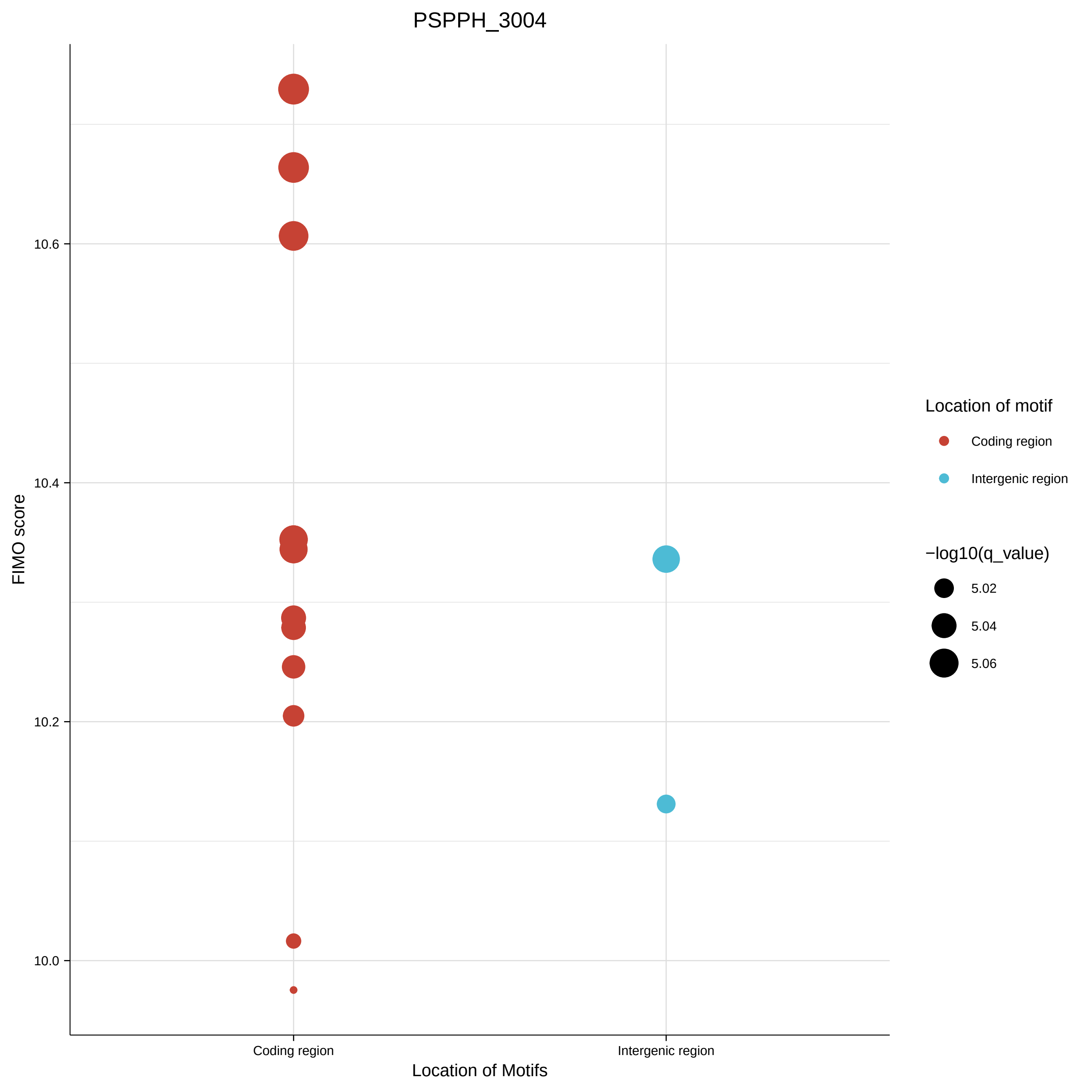

PSPPH_3004
FIMO score
10.6
10.4
10.2
10.0
Coding region
Intergenic region
Location of Motifs
Location of motif
Coding region
Intergenic region
−log10(q_value)
5.02
5.04
5.06

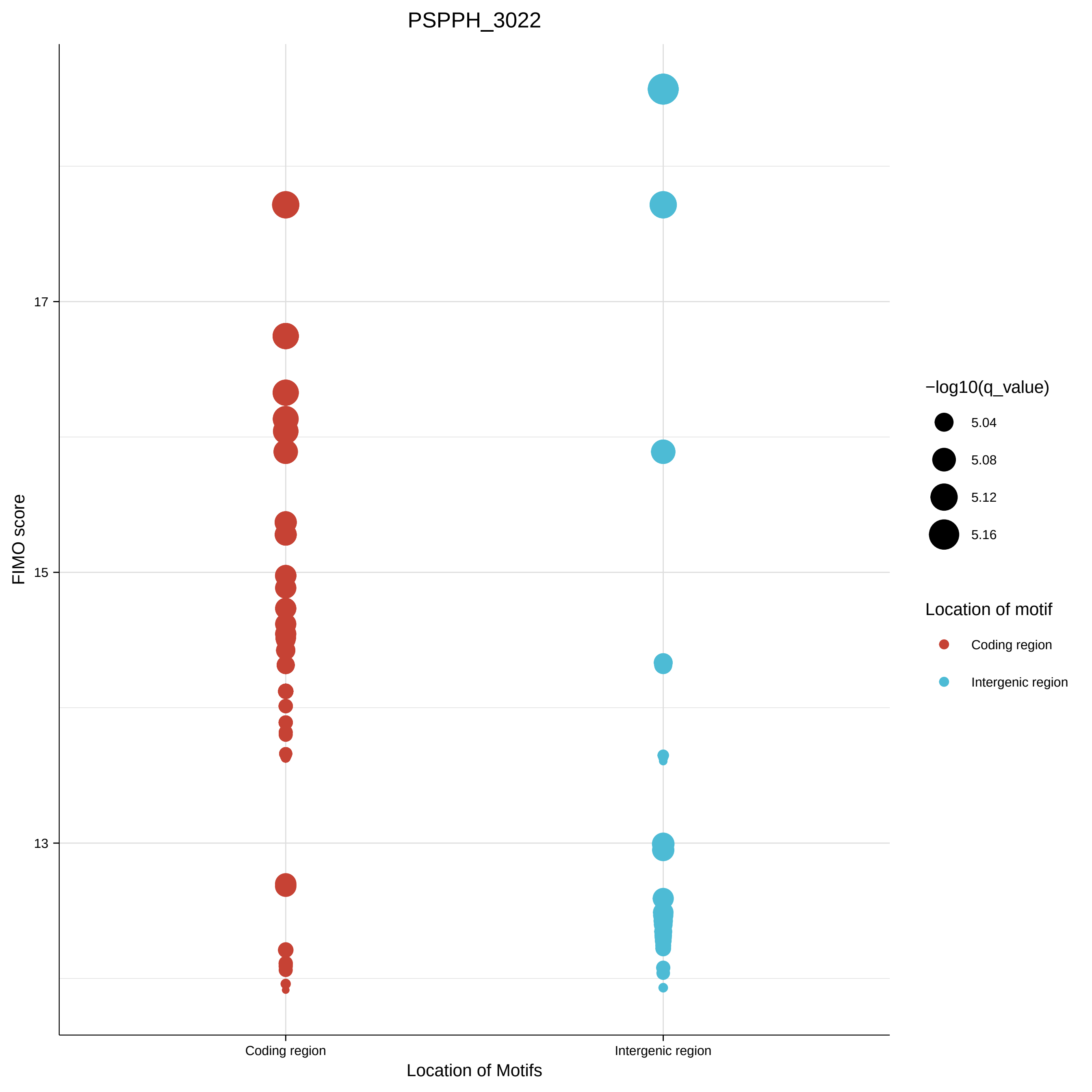
PSPPH_3022
FIMO score
17
15
13
Coding region
Intergenic region
Location of Motifs
−log10(q_value)
5.04
5.08
5.12
5.16
Location of motif
Coding region
Intergenic region

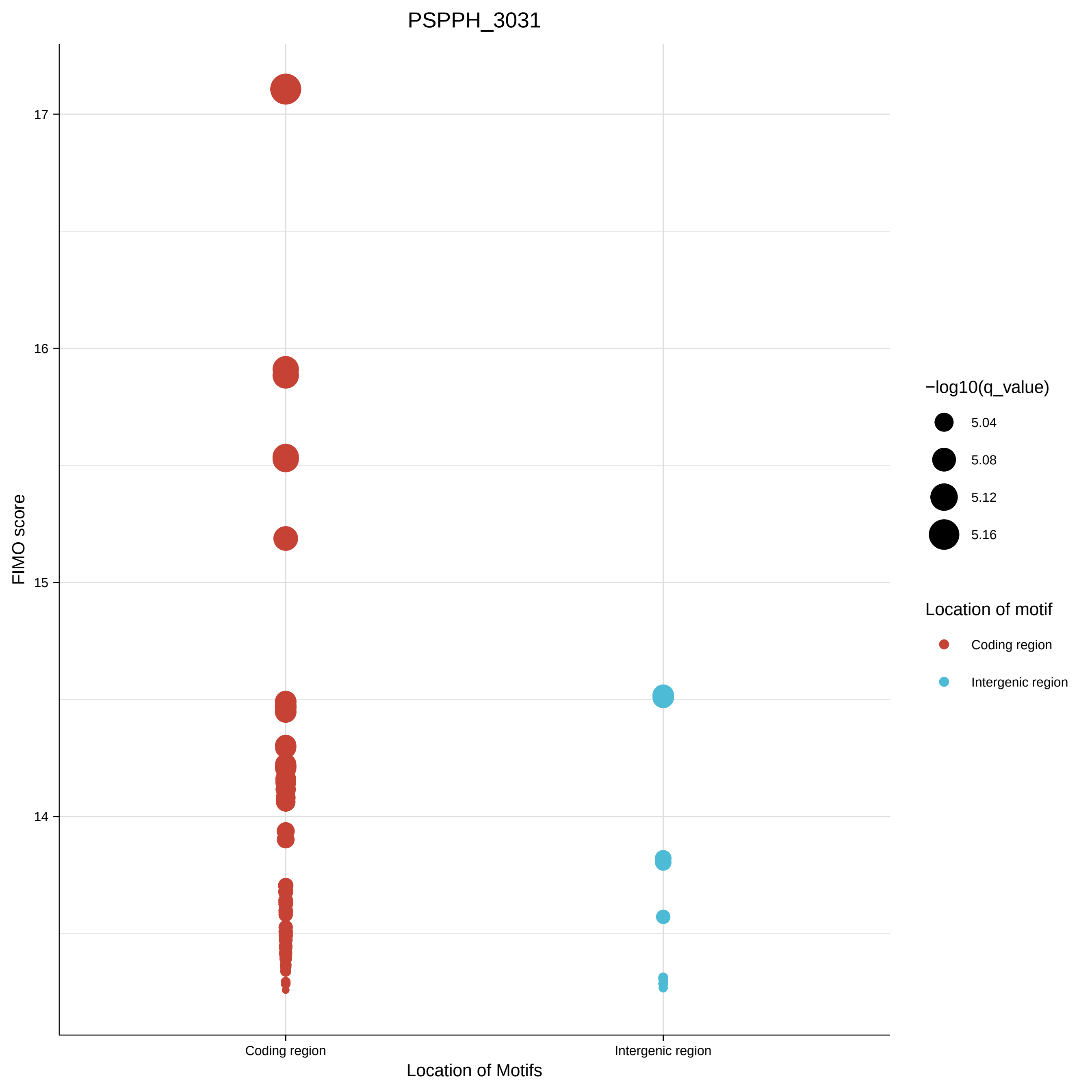

PSPPH_3031
FIMO score
17
16
15
14
Coding region
Intergenic region
Location of Motifs
−log10(q_value)
5.04
5.08
5.12
5.16
Location of motif
Coding region
Intergenic region

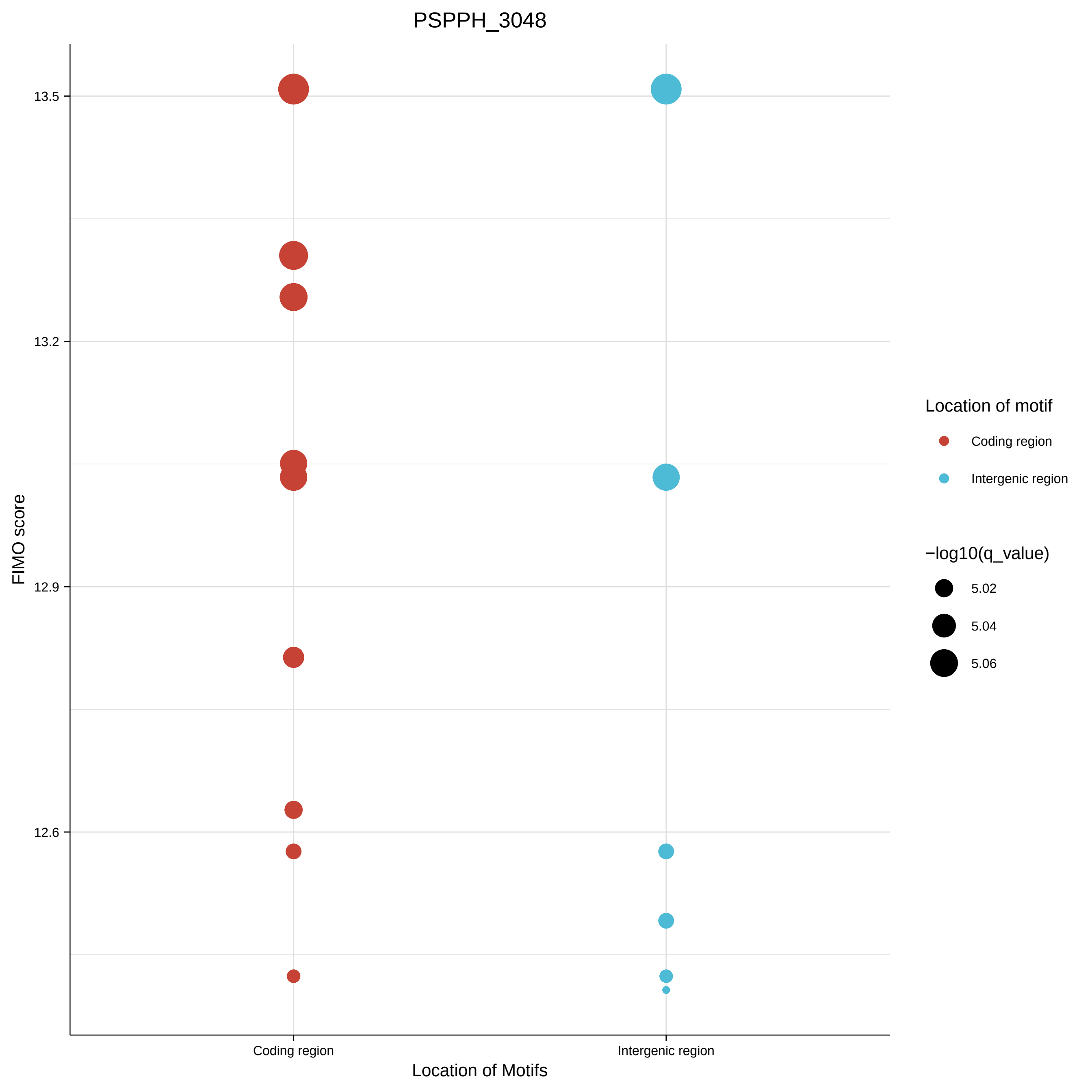

PSPPH_3048
FIMO score
13.5
13.2
12.9
12.6
Coding region
Intergenic region
Location of Motifs
Location of motif
Coding region
Intergenic region
−log10(q_value)
5.02
5.04
5.06

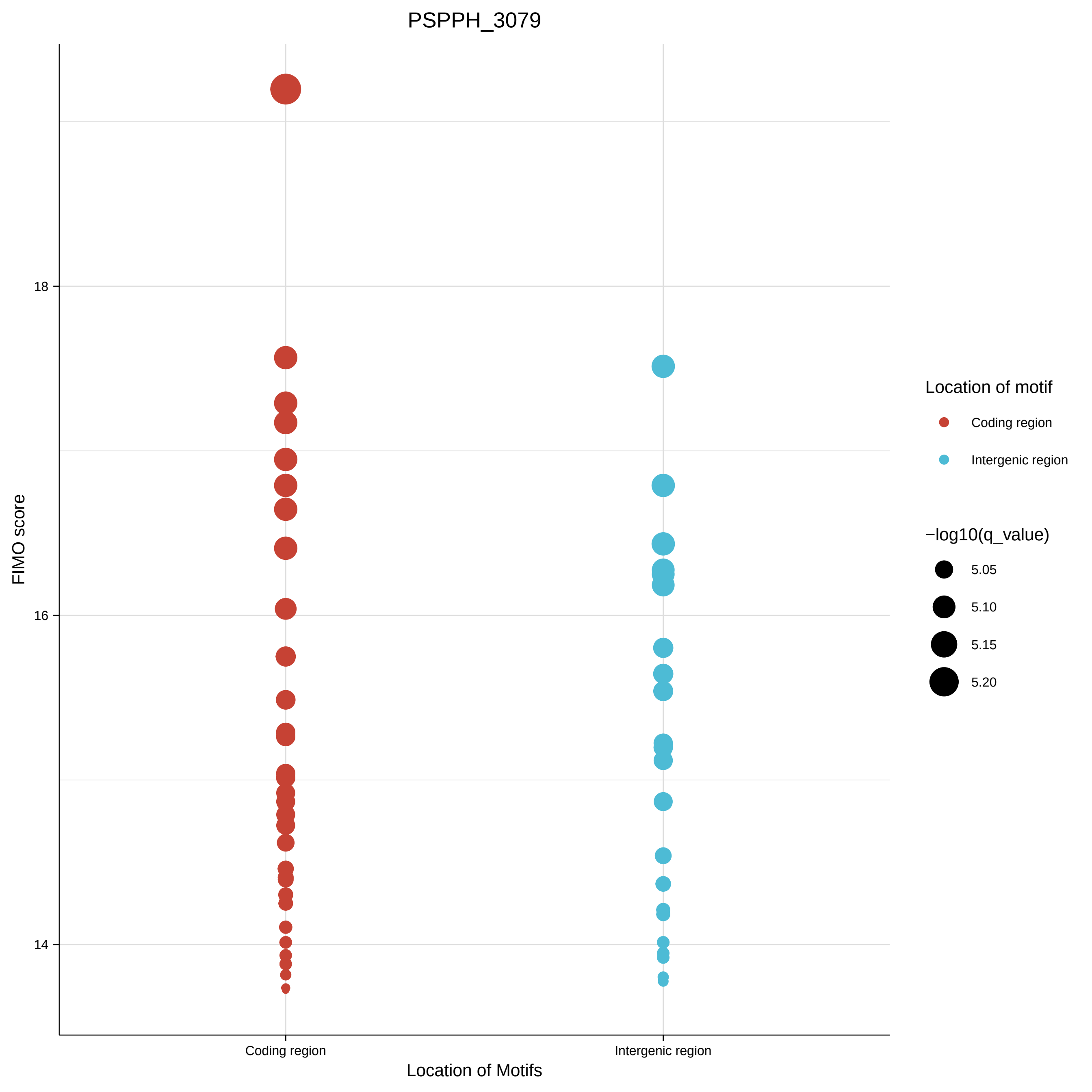

PSPPH_3079
FIMO score
18
16
14
Coding region
Intergenic region
Location of Motifs
Location of motif
Coding region
Intergenic region
−log10(q_value)
5.05
5.10
5.15
5.20

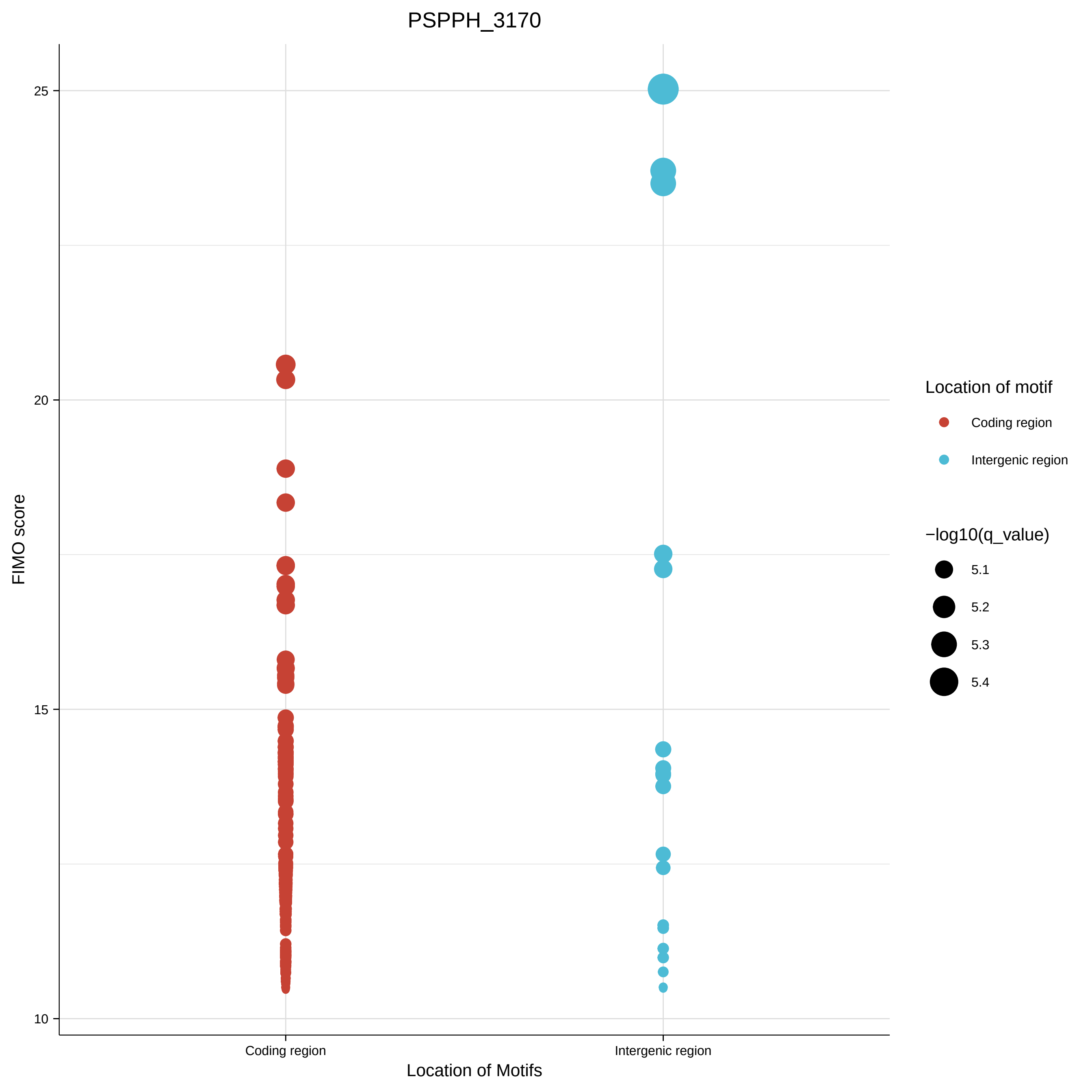
PSPPH_3170
FIMO score
25
20
15
10
Coding region
Intergenic region
Location of Motifs
Location of motif
Coding region
Intergenic region
−log10(q_value)
5.1
5.2
5.3
5.4

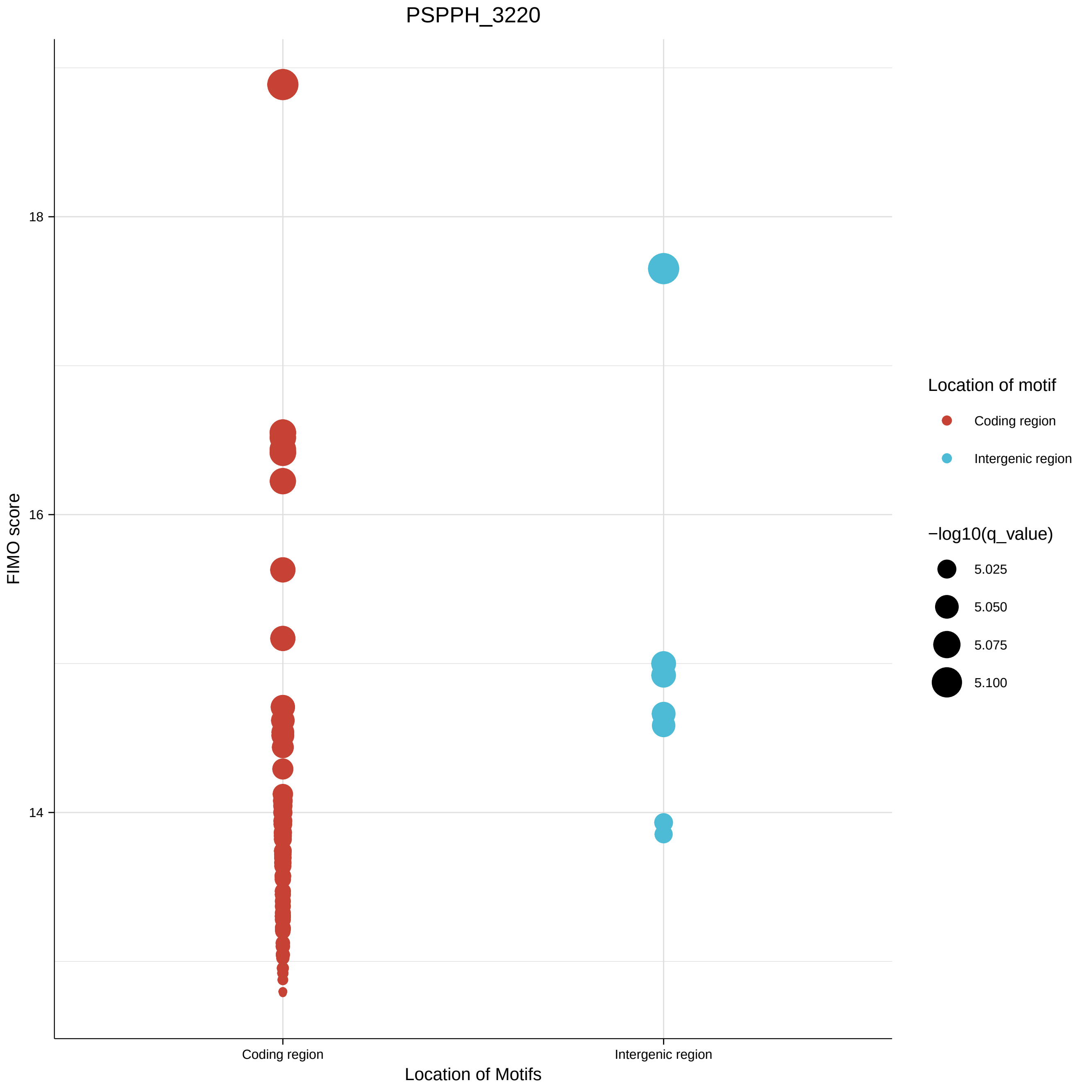
PSPPH_3220
FIMO score
18
16
14
Coding region
Intergenic region
Location of Motifs
Location of motif
Coding region
Intergenic region
−log10(q_value)
5.025
5.050
5.075
5.100

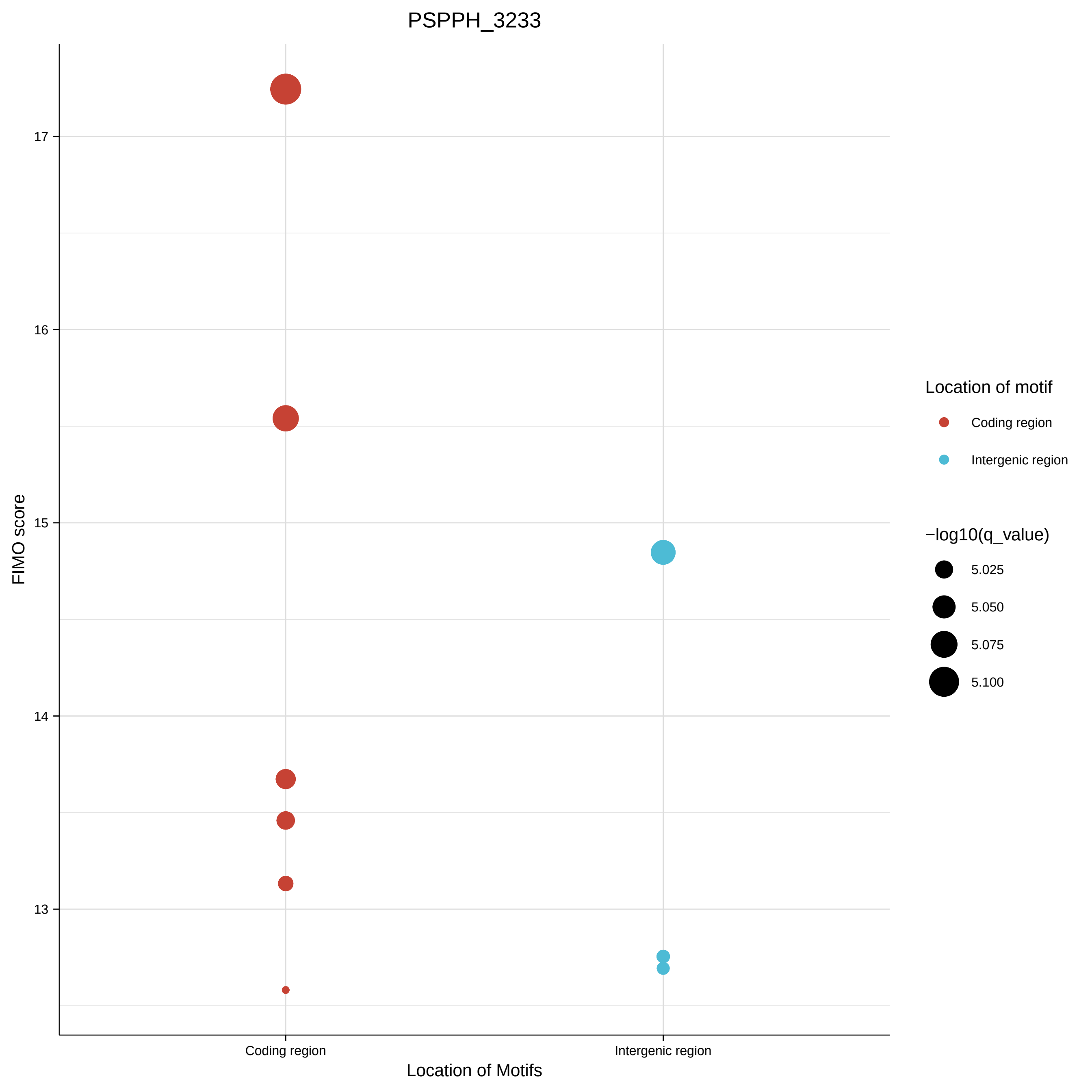
PSPPH_3233
FIMO score
17
16
15
14
13
Coding region
Intergenic region
Location of Motifs
Location of motif
Coding region
Intergenic region
−log10(q_value)
5.025
5.050
5.075
5.100

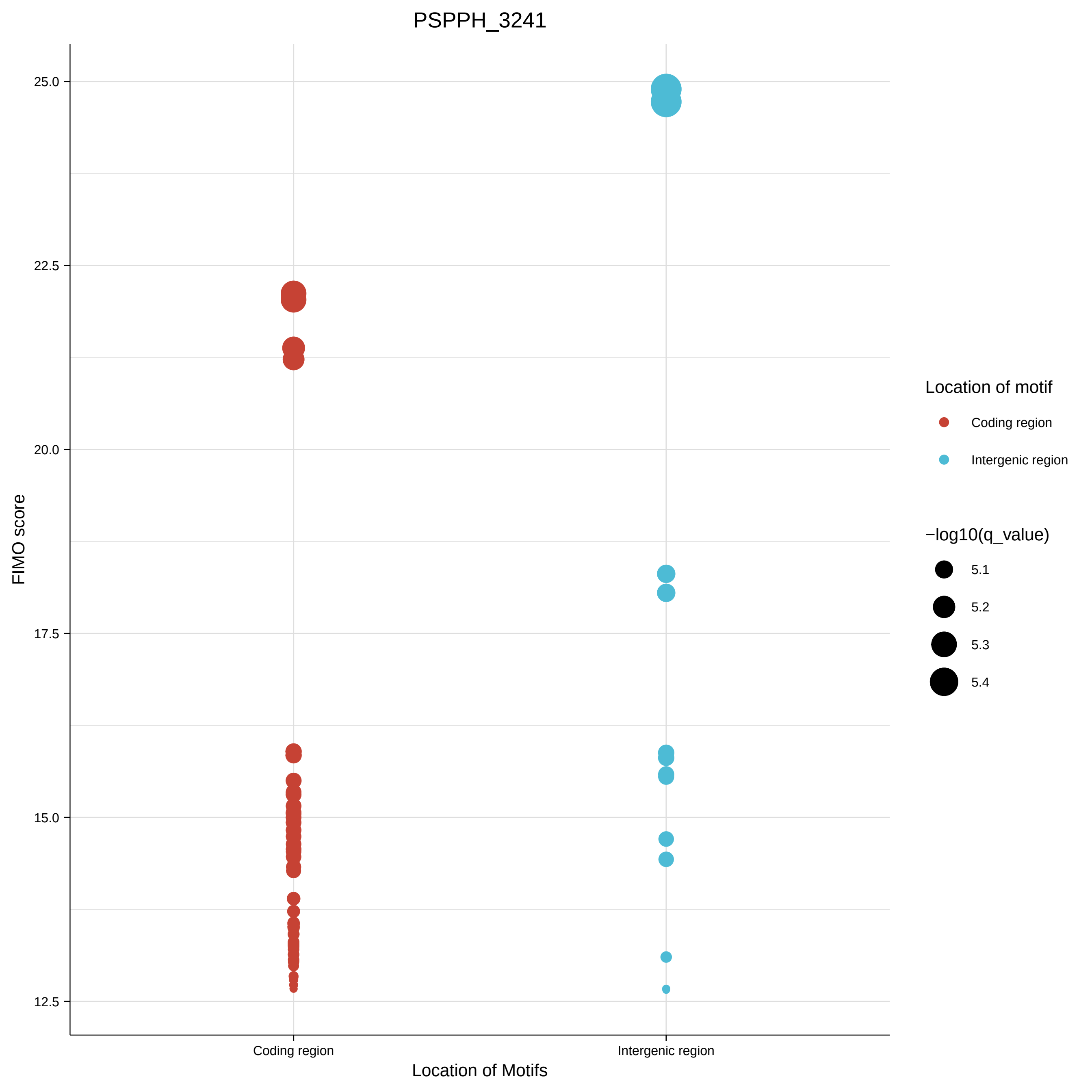

PSPPH_3241
FIMO score
25.0
22.5
20.0
17.5
15.0
12.5
Coding region
Intergenic region
Location of Motifs
Location of motif
Coding region
Intergenic region
−log10(q_value)
5.1
5.2
5.3
5.4

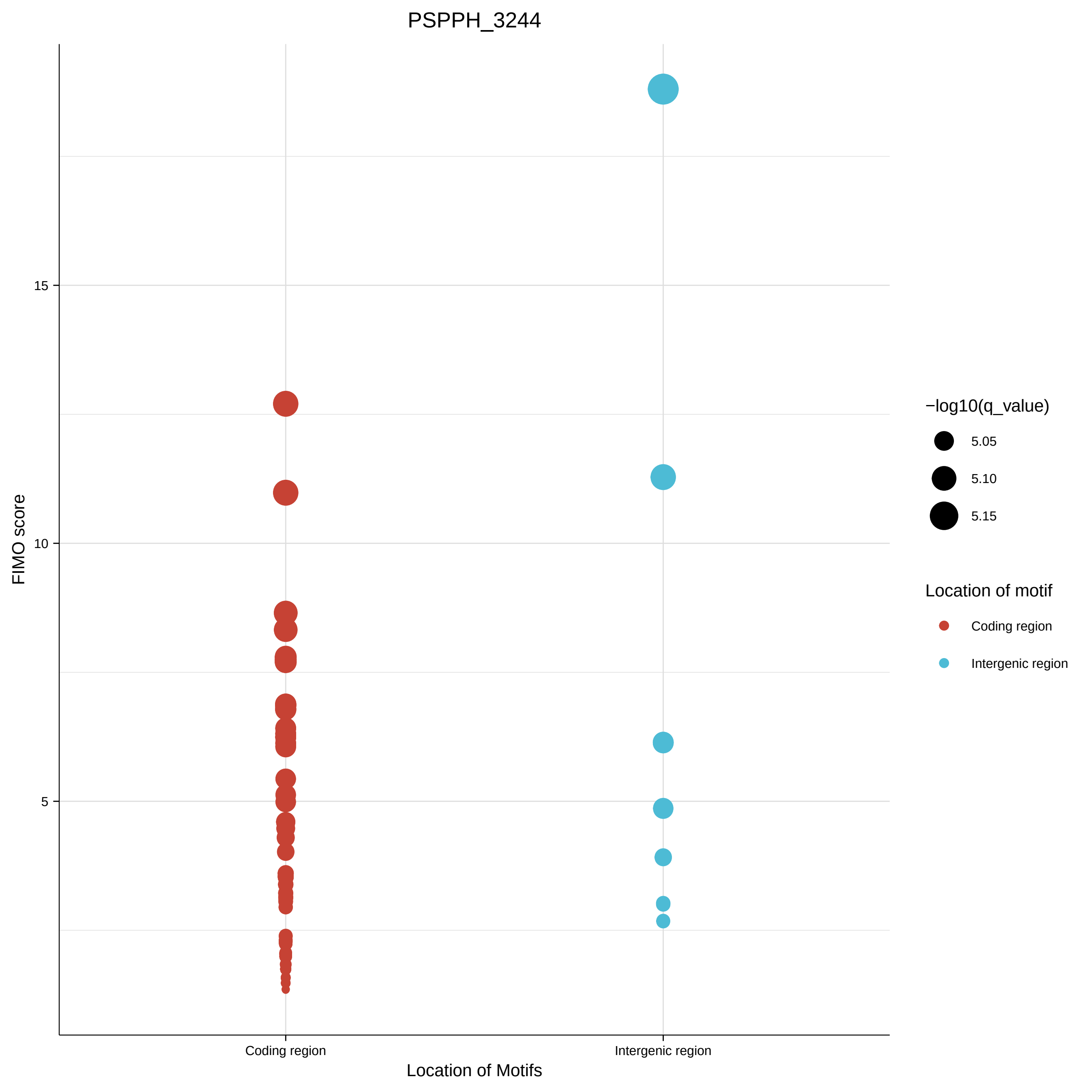

PSPPH_3244
FIMO score
15
10
5
Coding region
Intergenic region
Location of Motifs
−log10(q_value)
5.05
5.10
5.15
Location of motif
Coding region
Intergenic region

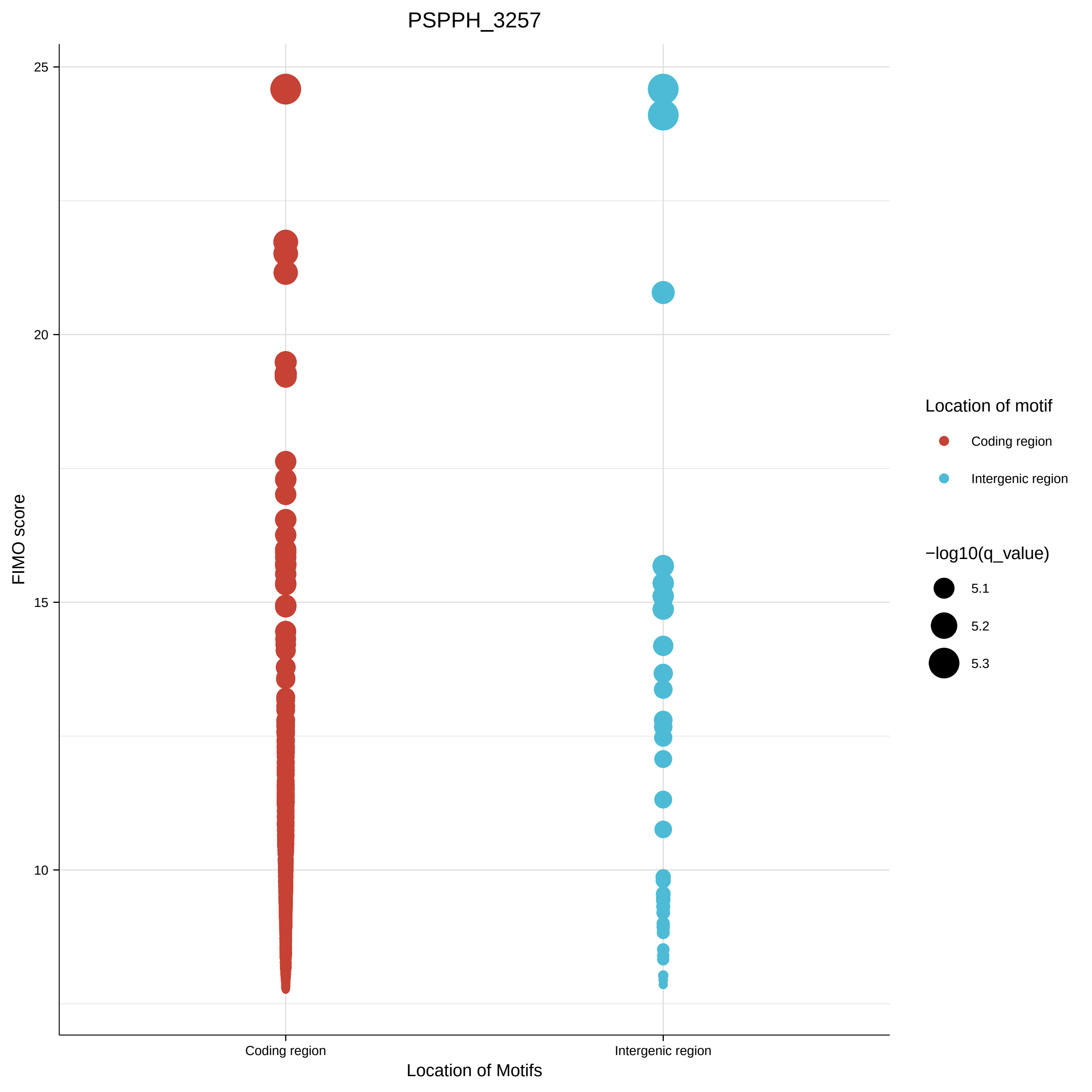

PSPPH_3257
FIMO score
25
20
15
10
Coding region
Intergenic region
Location of Motifs
Location of motif
Coding region
Intergenic region
−log10(q_value)
5.1
5.2
5.3

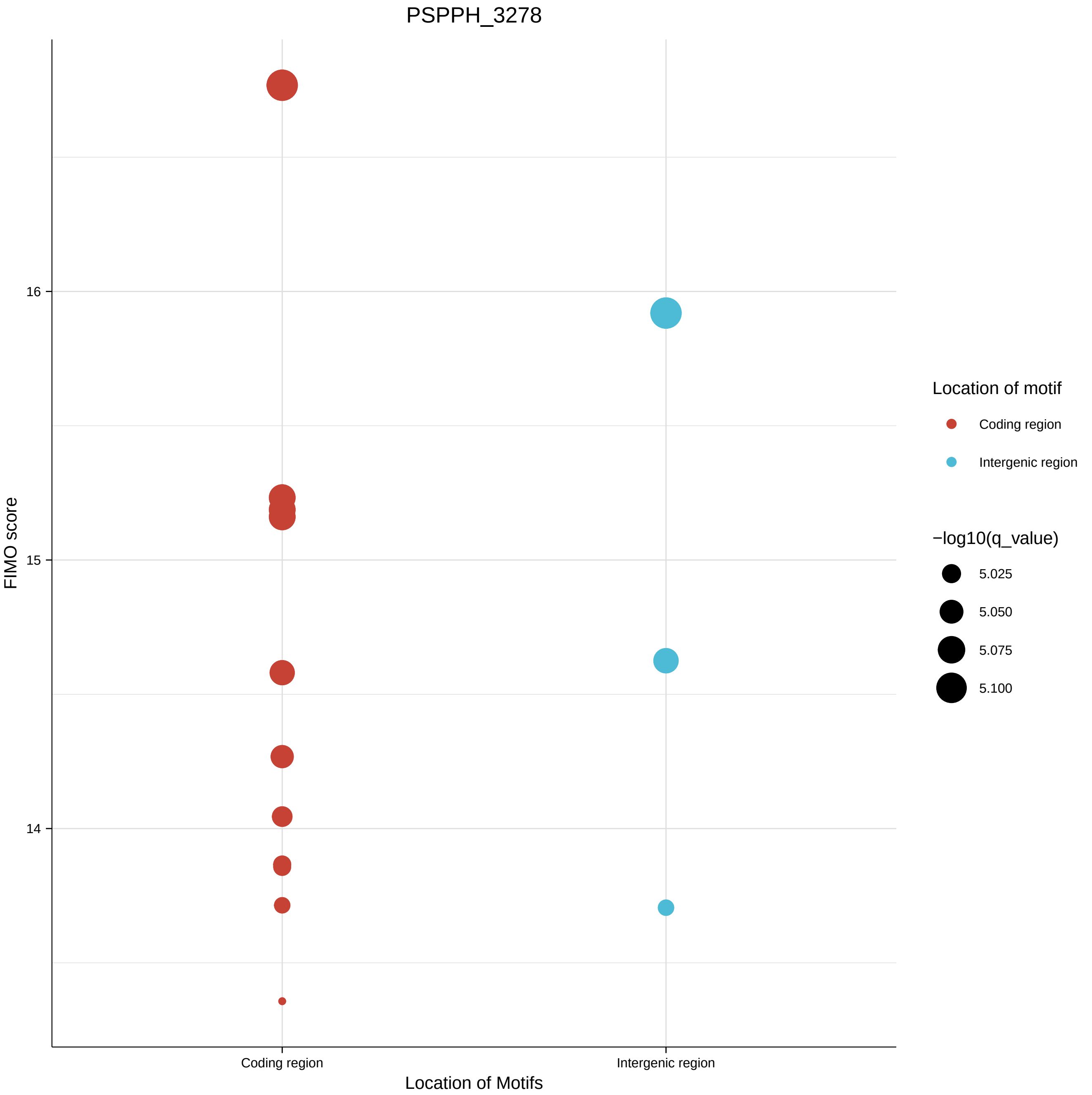

PSPPH_3278
FIMO score
16
15
14
Coding region
Intergenic region
Location of Motifs
Location of motif
Coding region
Intergenic region
−log10(q_value)
5.025
5.050
5.075
5.100

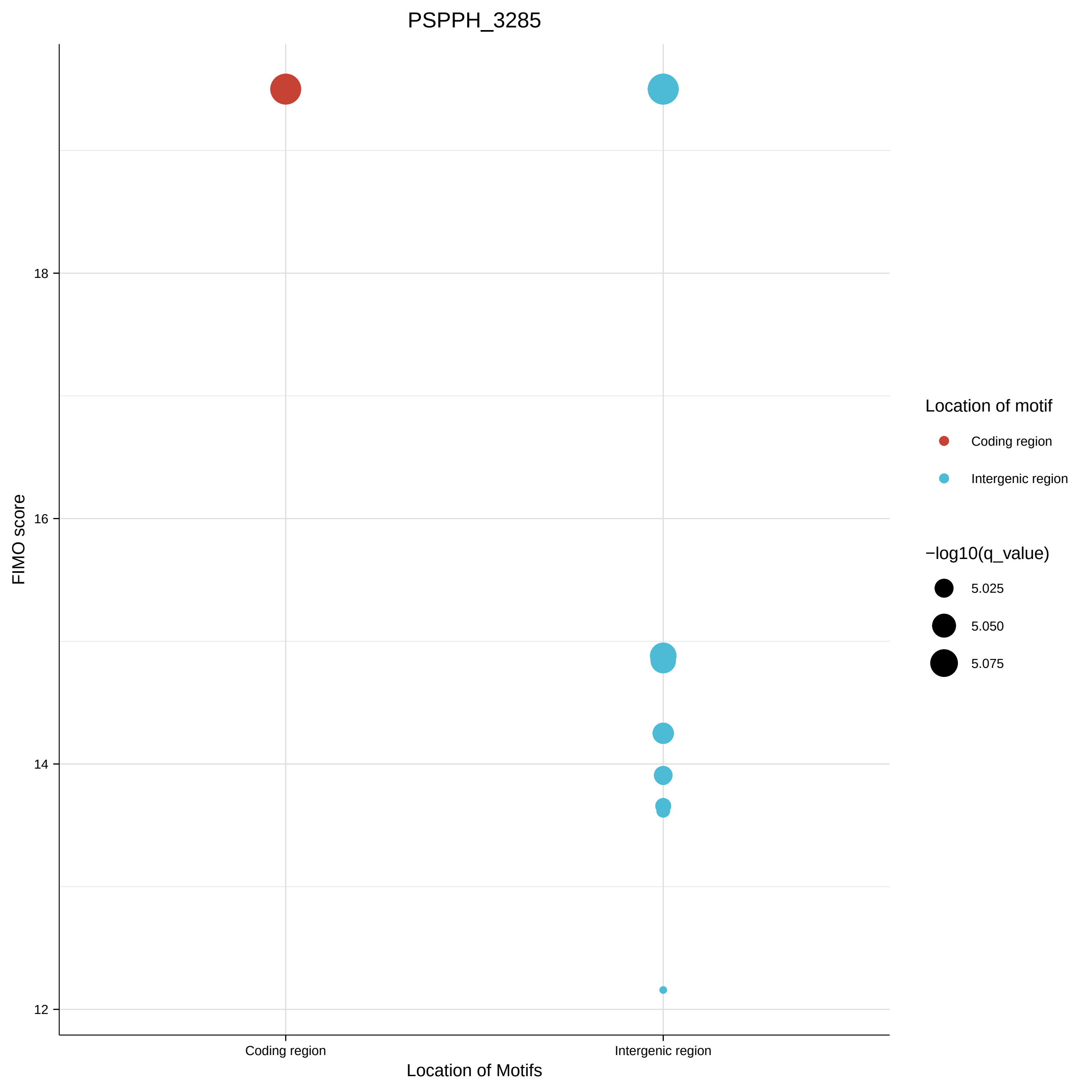

PSPPH_3285
FIMO score
18
16
14
12
Coding region
Intergenic region
Location of Motifs
Location of motif
Coding region
Intergenic region
−log10(q_value)
5.025
5.050
5.075

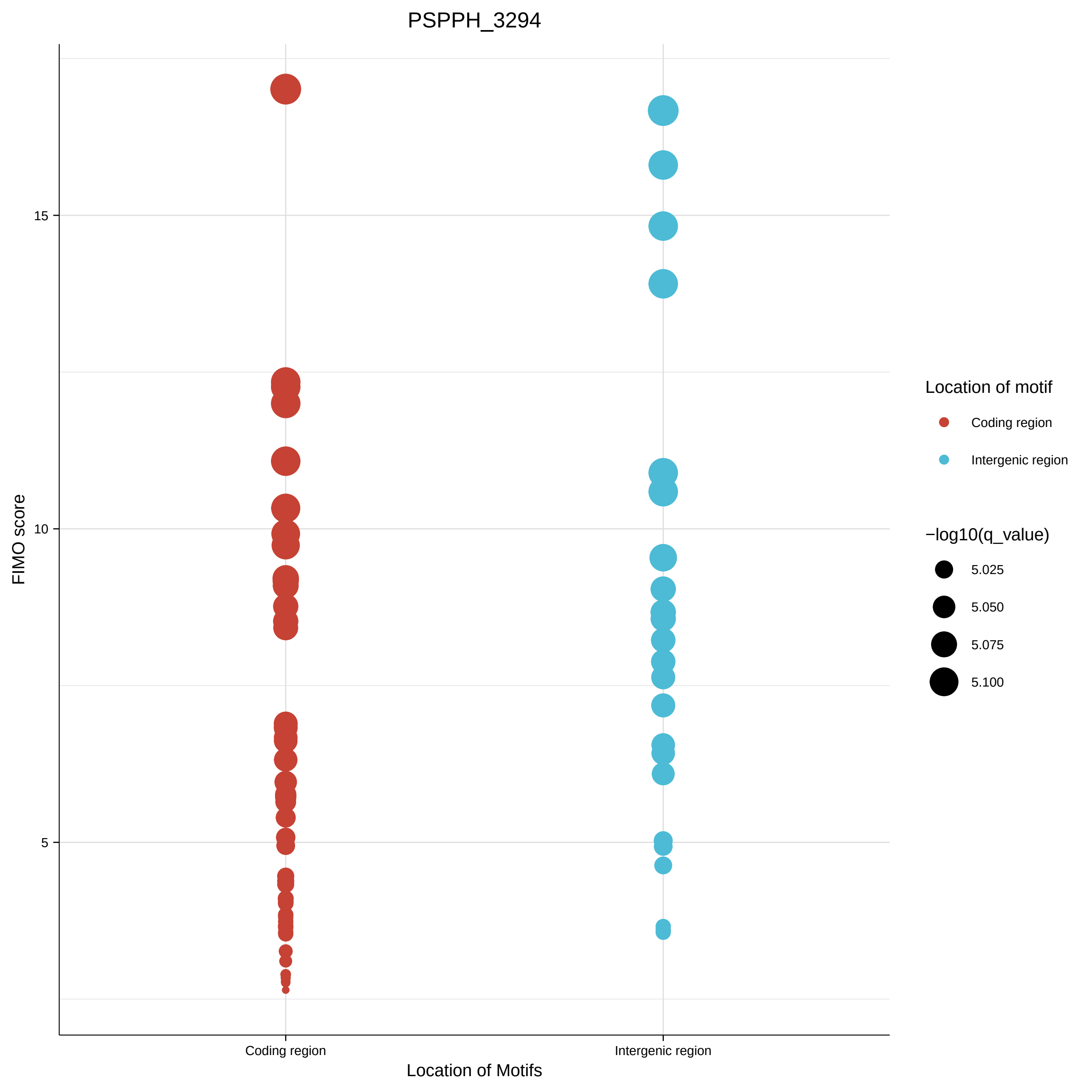

PSPPH_3294
FIMO score
15
10
5
Coding region
Intergenic region
Location of Motifs
Location of motif
Coding region
Intergenic region
−log10(q_value)
5.025
5.050
5.075
5.100

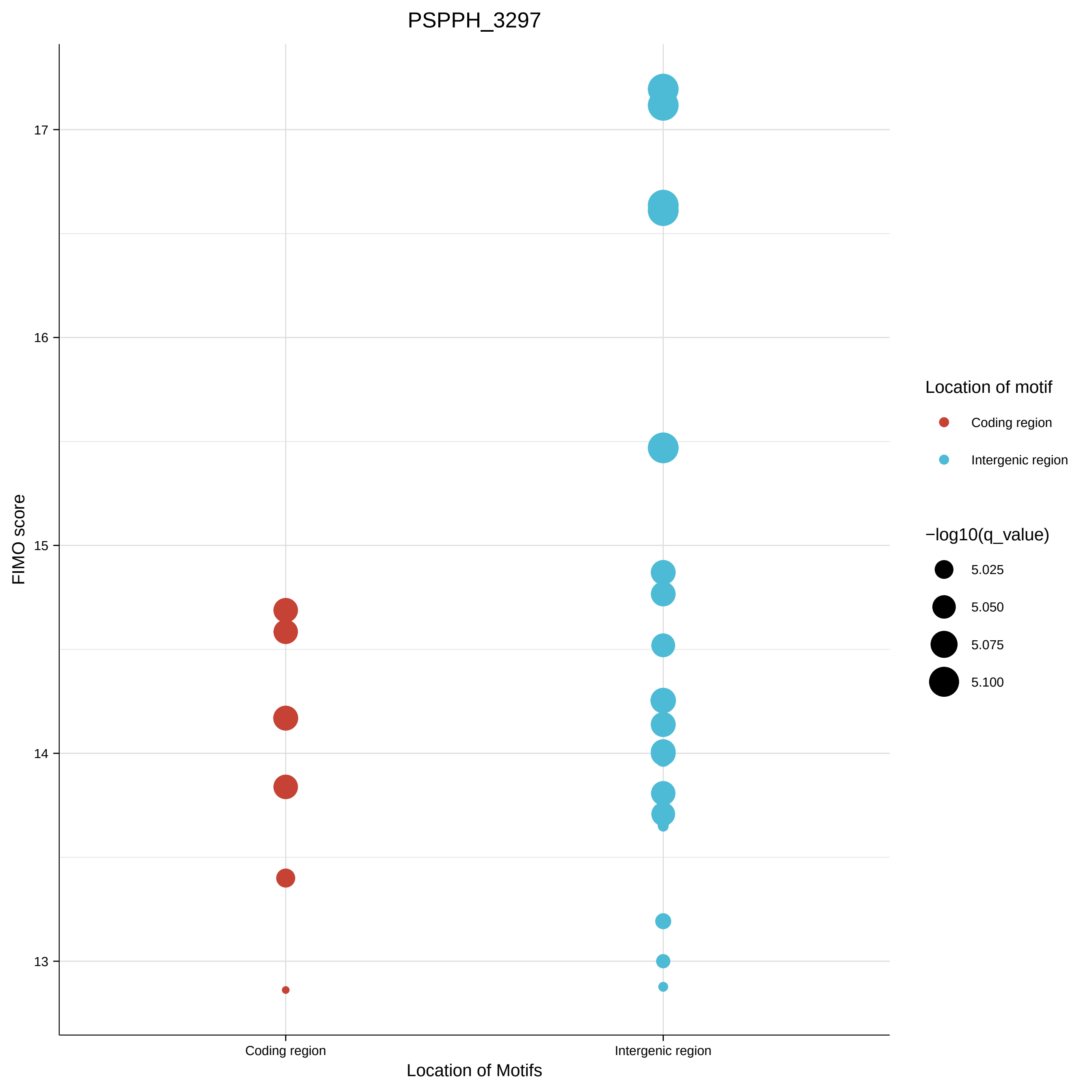
PSPPH_3297
FIMO score
17
16
15
14
13
Coding region
Intergenic region
Location of Motifs
Location of motif
Coding region
Intergenic region
−log10(q_value)
5.025
5.050
5.075
5.100

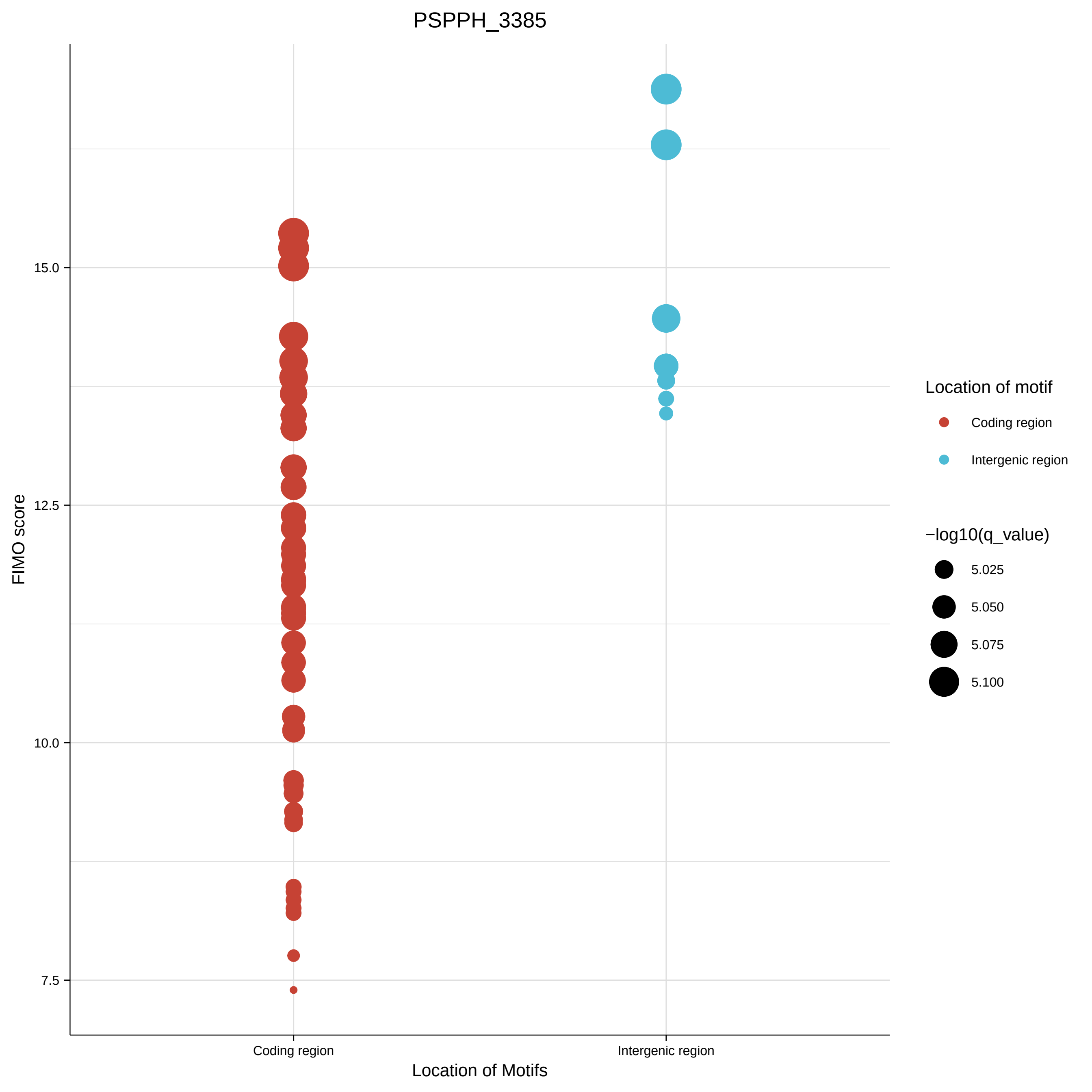

PSPPH_3385
FIMO score
15.0
12.5
10.0
7.5
Coding region
Intergenic region
Location of Motifs
Location of motif
Coding region
Intergenic region
−log10(q_value)
5.025
5.050
5.075
5.100

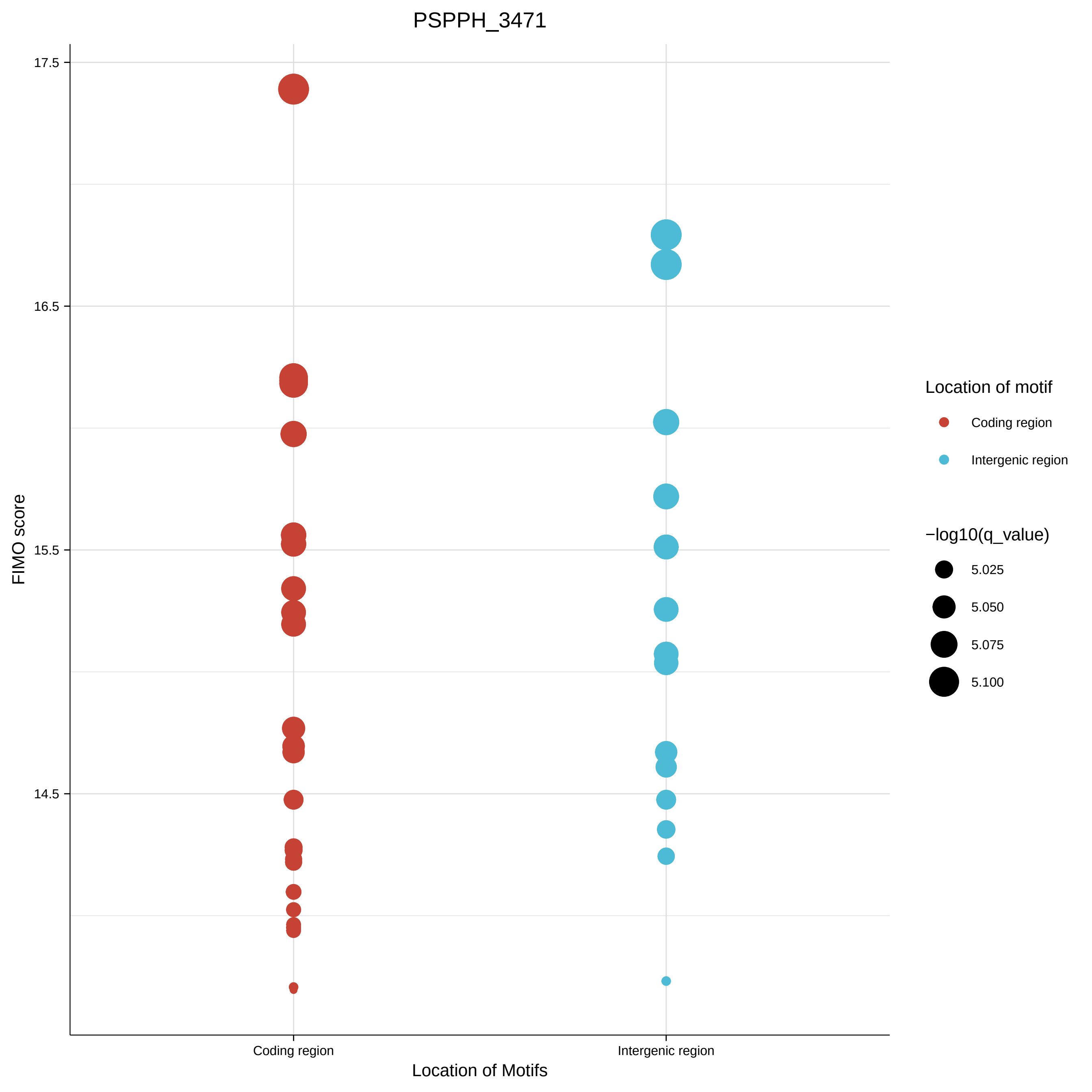

PSPPH_3471
FIMO score
17.5
16.5
15.5
14.5
Coding region
Intergenic region
Location of Motifs
Location of motif
Coding region
Intergenic region
−log10(q_value)
5.025
5.050
5.075
5.100

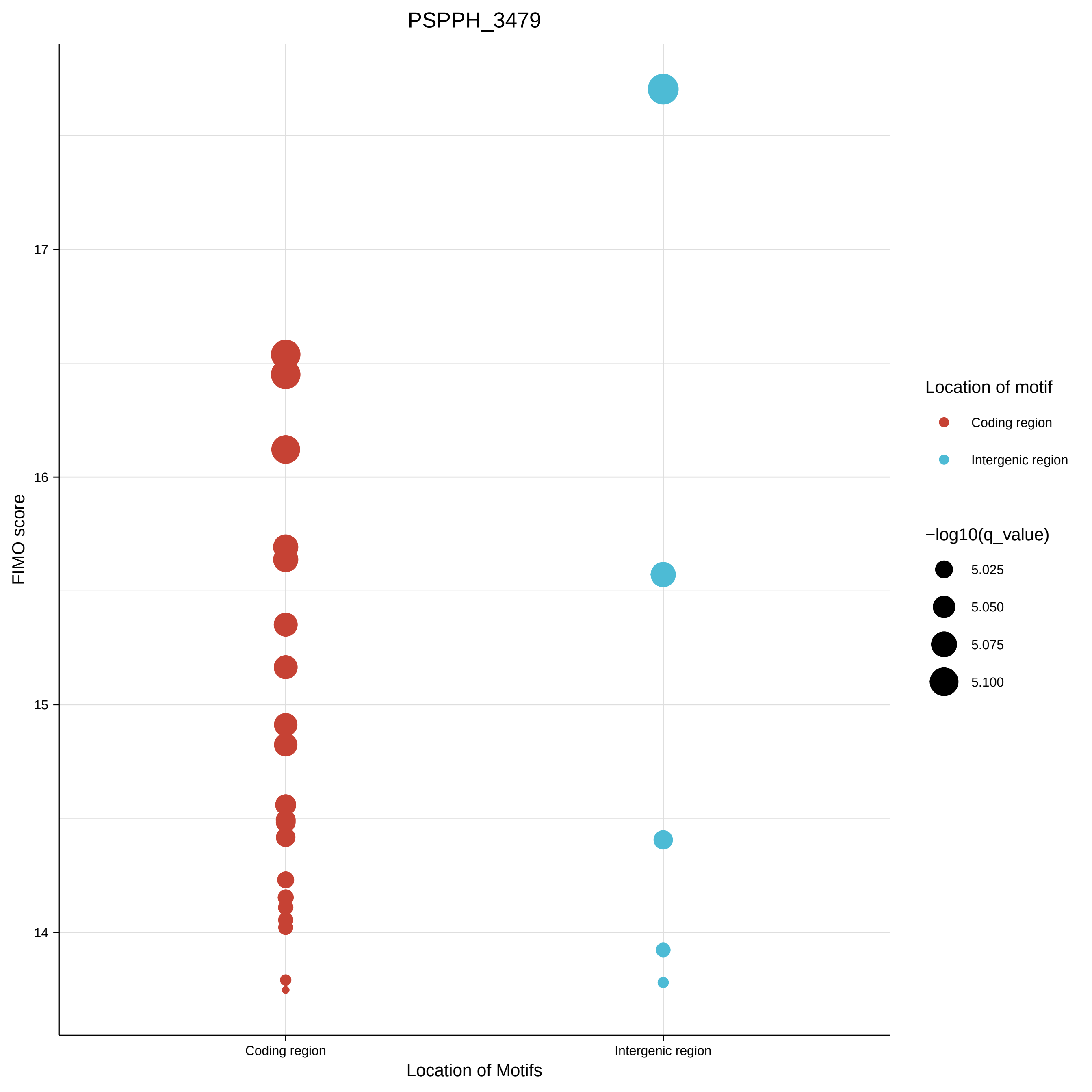
PSPPH_3479
FIMO score
17
16
15
14
Coding region
Intergenic region
Location of Motifs
Location of motif
Coding region
Intergenic region
−log10(q_value)
5.025
5.050
5.075
5.100

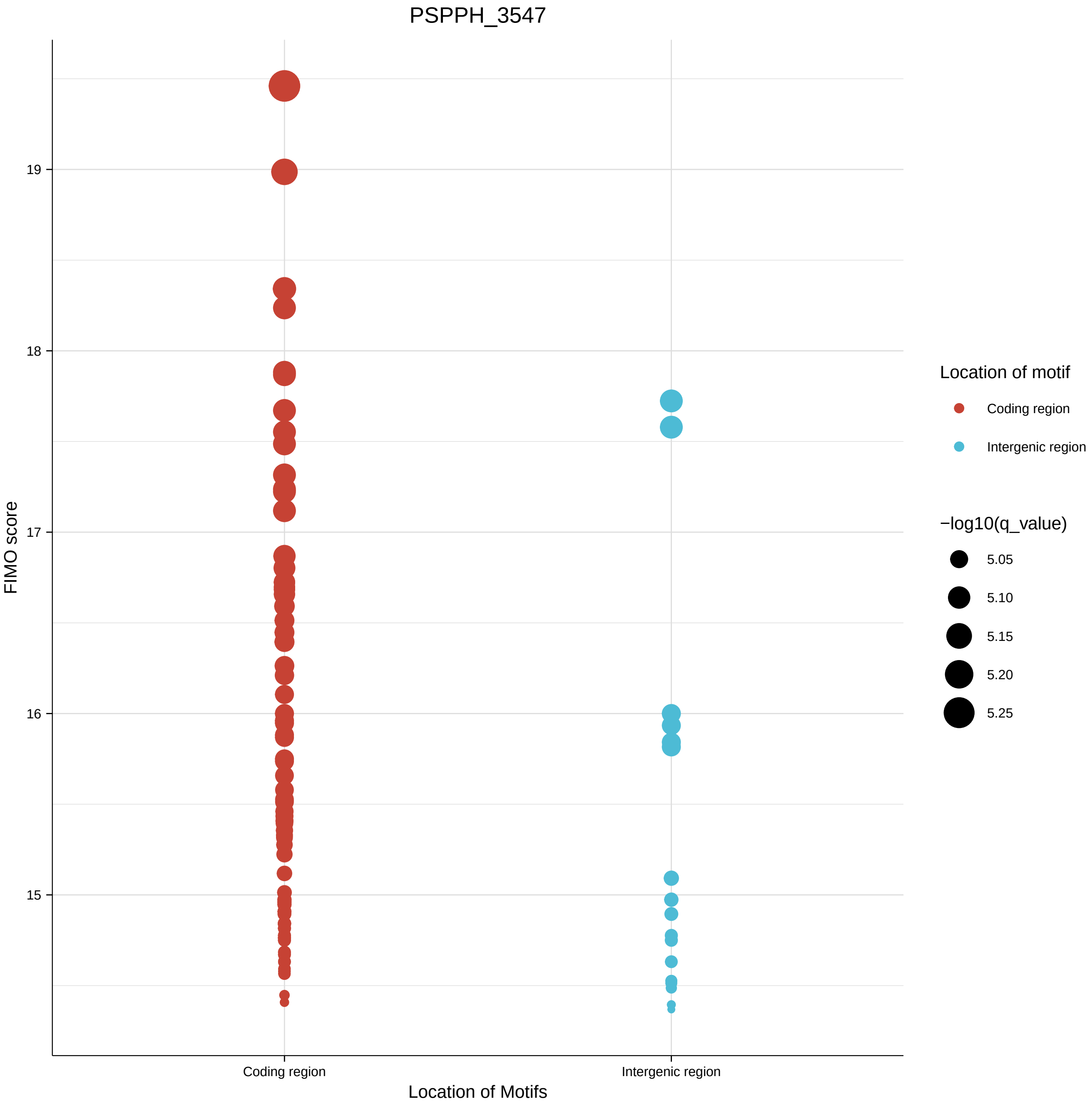
PSPPH_3547
FIMO score
19
18
17
16
15
Coding region
Intergenic region
Location of Motifs
Location of motif
Coding region
Intergenic region
−log10(q_value)
5.05
5.10
5.15
5.20
5.25

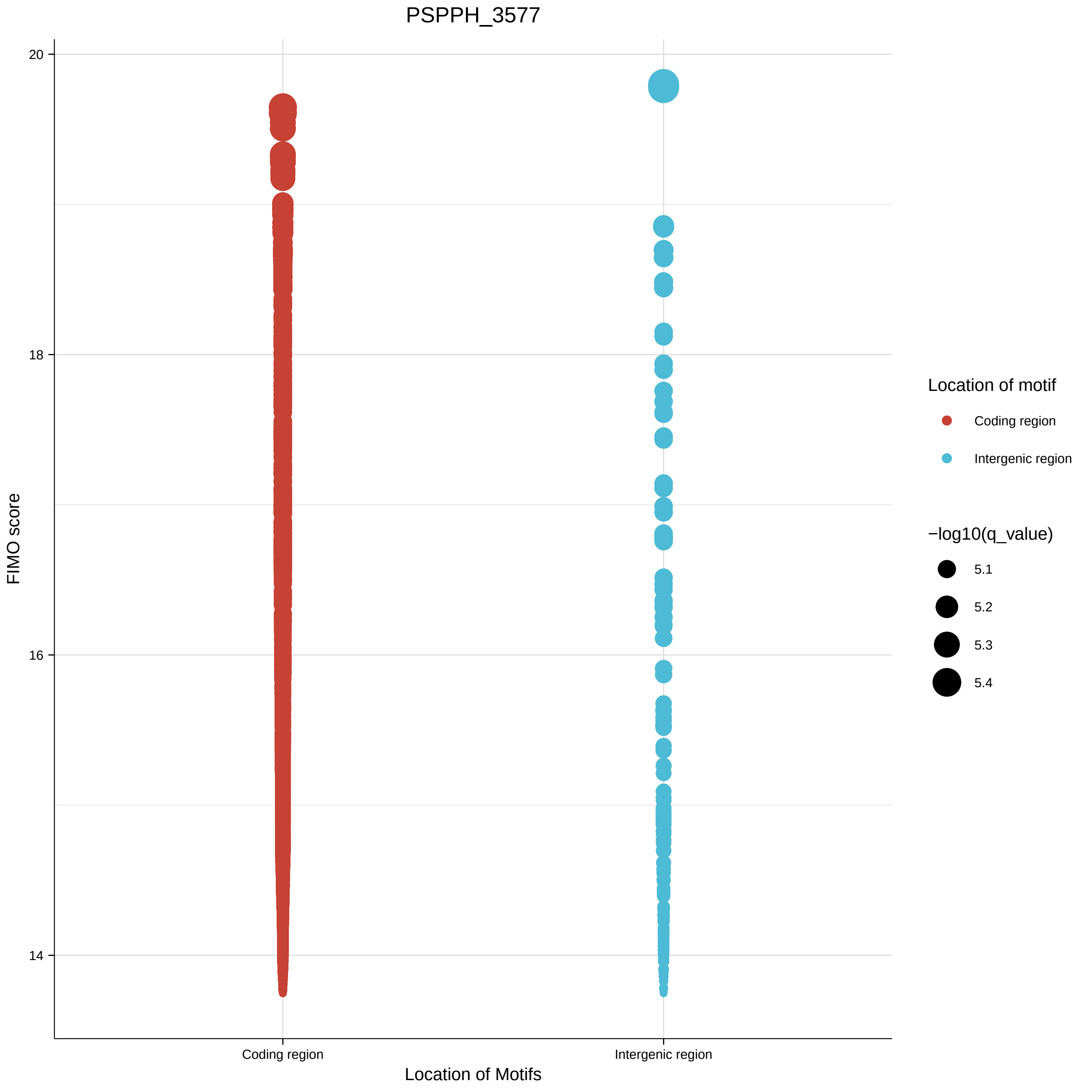

PSPPH_3577
FIMO score
20
18
16
14
Coding region
Intergenic region
Location of Motifs
Location of motif
Coding region
Intergenic region
−log10(q_value)
5.1
5.2
5.3
5.4

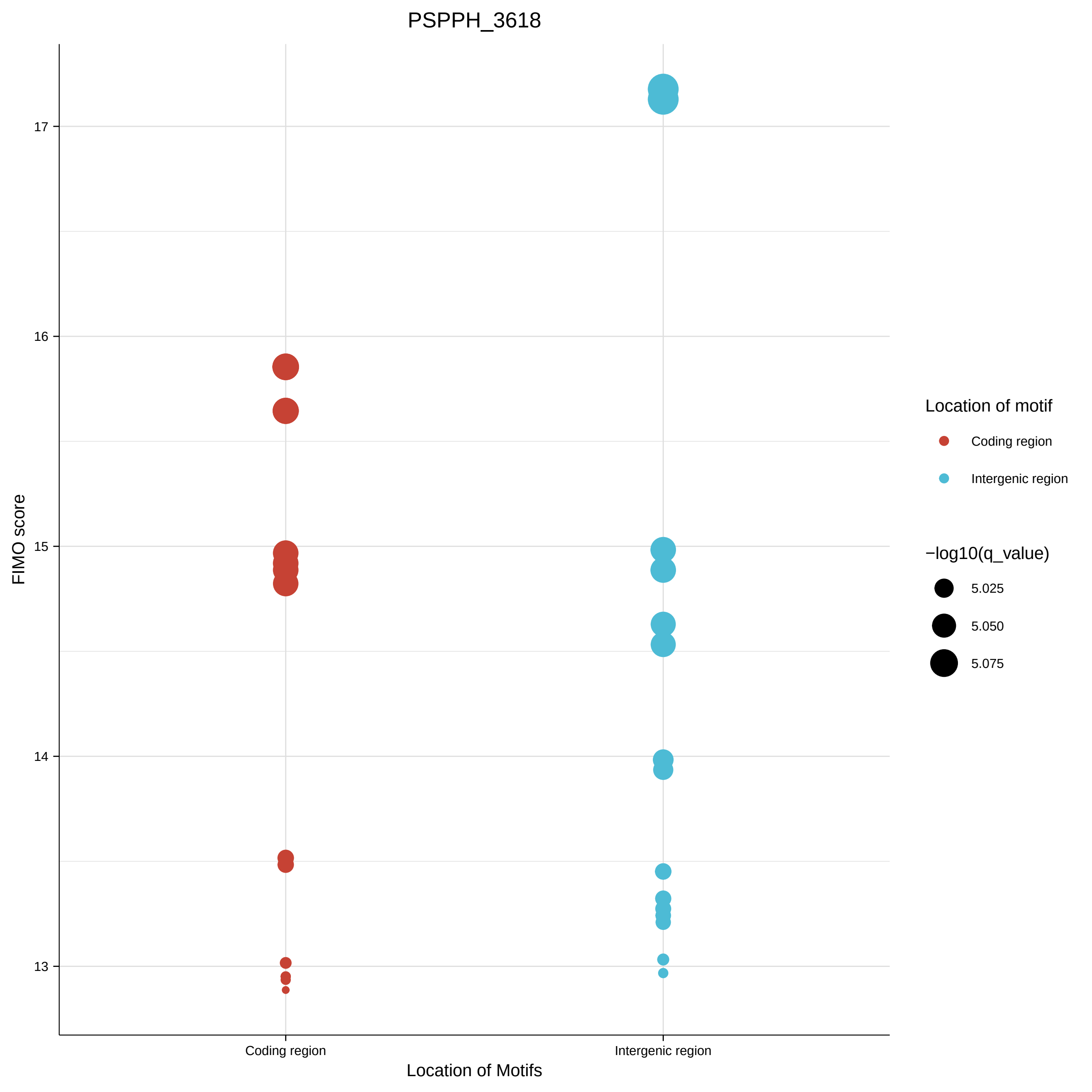

PSPPH_3618
FIMO score
17
16
15
14
13
Coding region
Intergenic region
Location of Motifs
Location of motif
Coding region
Intergenic region
−log10(q_value)
5.025
5.050
5.075

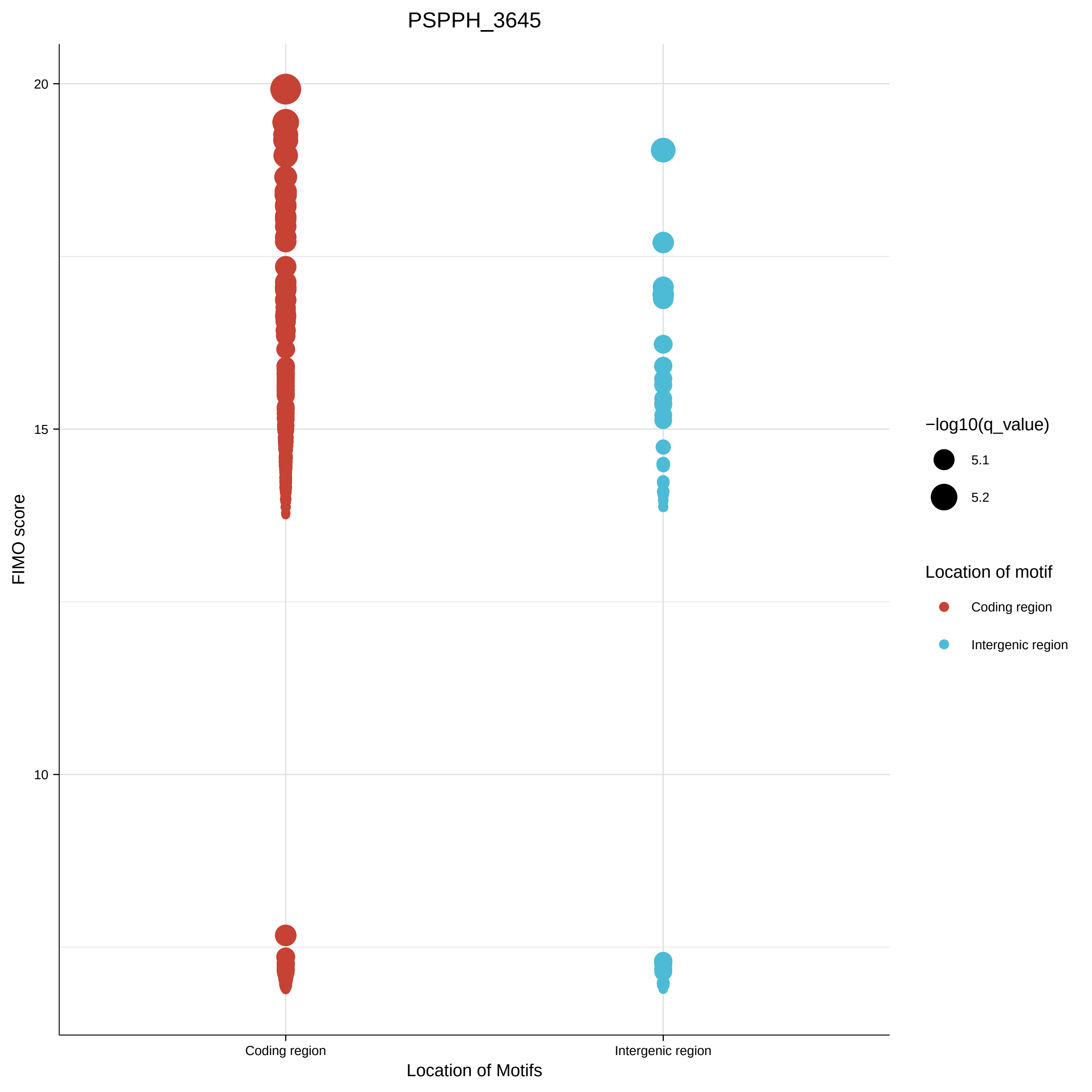

PSPPH_3645
FIMO score
20
15
10
Coding region
Intergenic region
Location of Motifs
−log10(q_value)
5.1
5.2
Location of motif
Coding region
Intergenic region

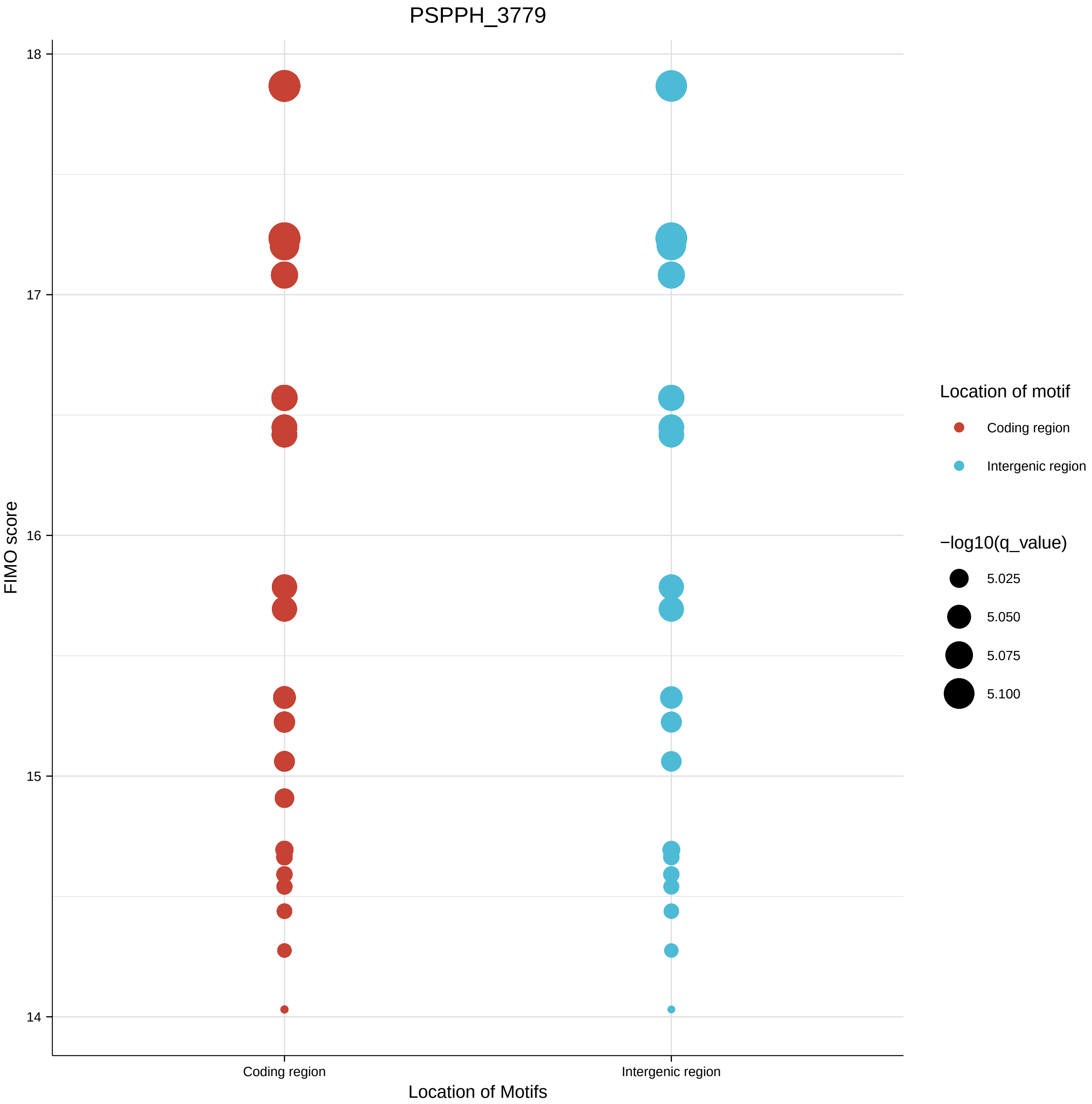

PSPPH_3779
FIMO score
18
17
16
15
14
Coding region
Intergenic region
Location of Motifs
Location of motif
Coding region
Intergenic region
−log10(q_value)
5.025
5.050
5.075
5.100

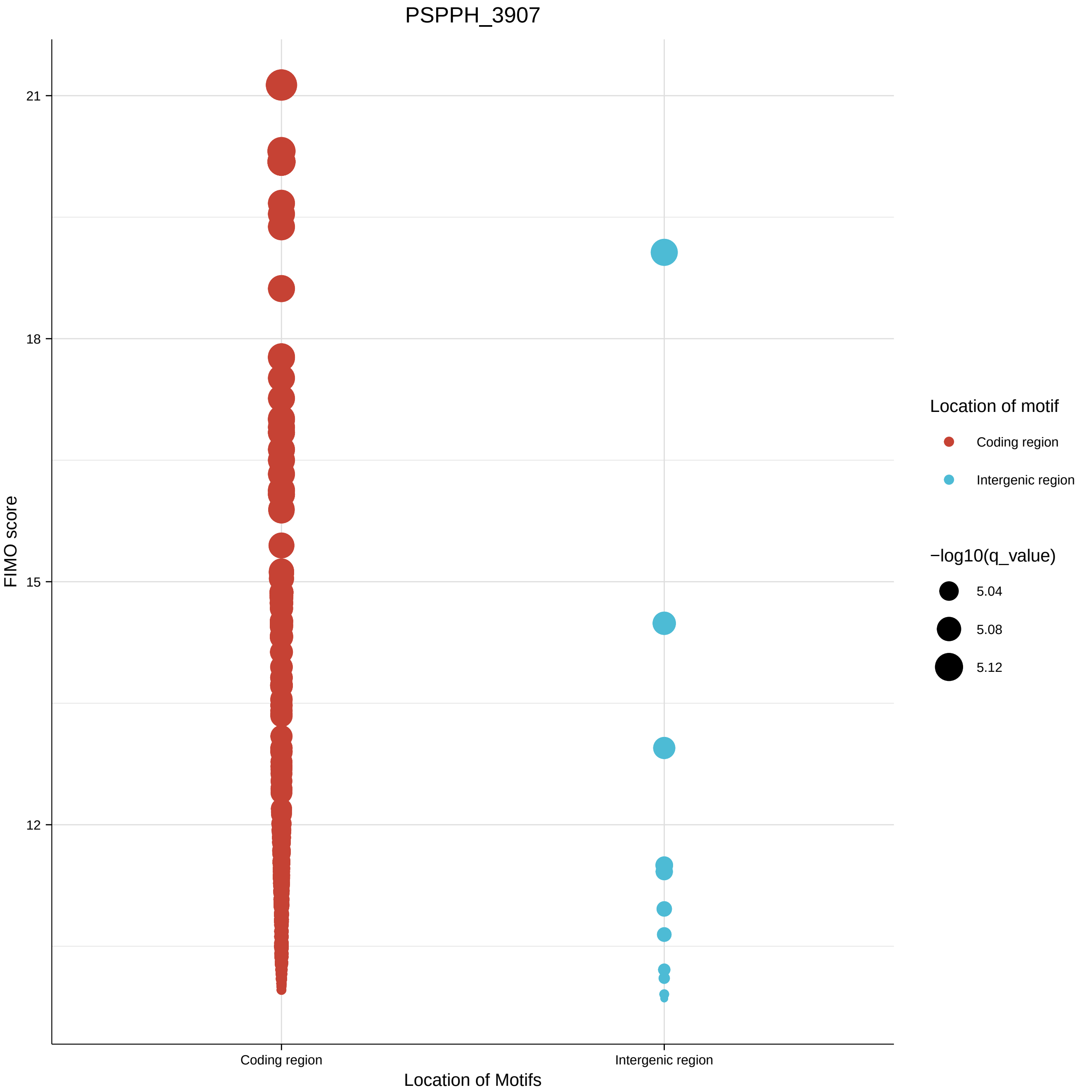

PSPPH_3907
21
18
15
12
FIMO score
Coding region
Intergenic region
Location of Motifs
Location of motif
Coding region
Intergenic region
−log10(q_value)
5.04
5.08
5.12

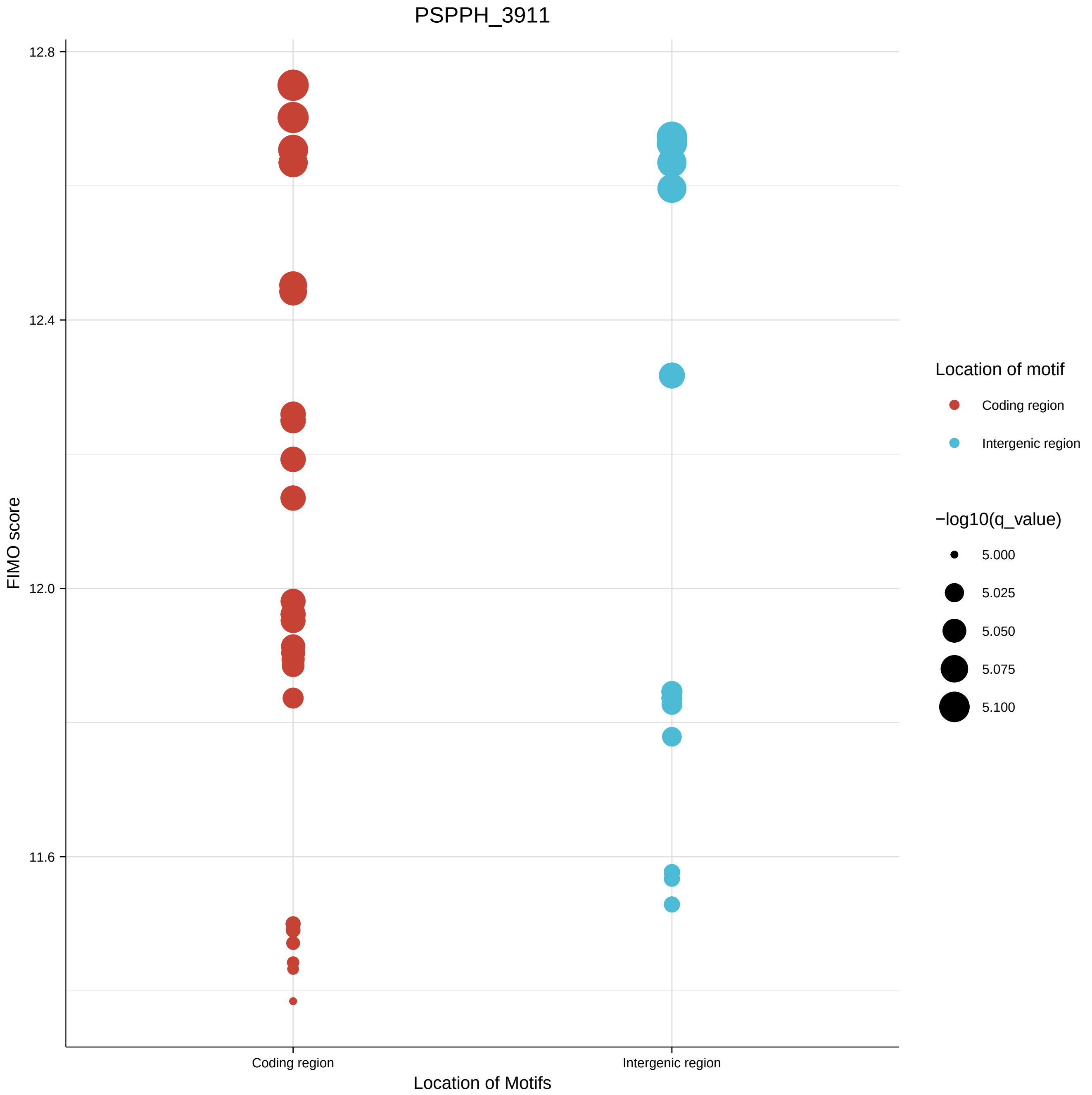
PSPPH_3911
FIMO score
12.8
12.4
12.0
11.6
Coding region
Intergenic region
Location of Motifs
Location of motif
Coding region
Intergenic region
−log10(q_value)
5.000
5.025
5.050
5.075
5.100

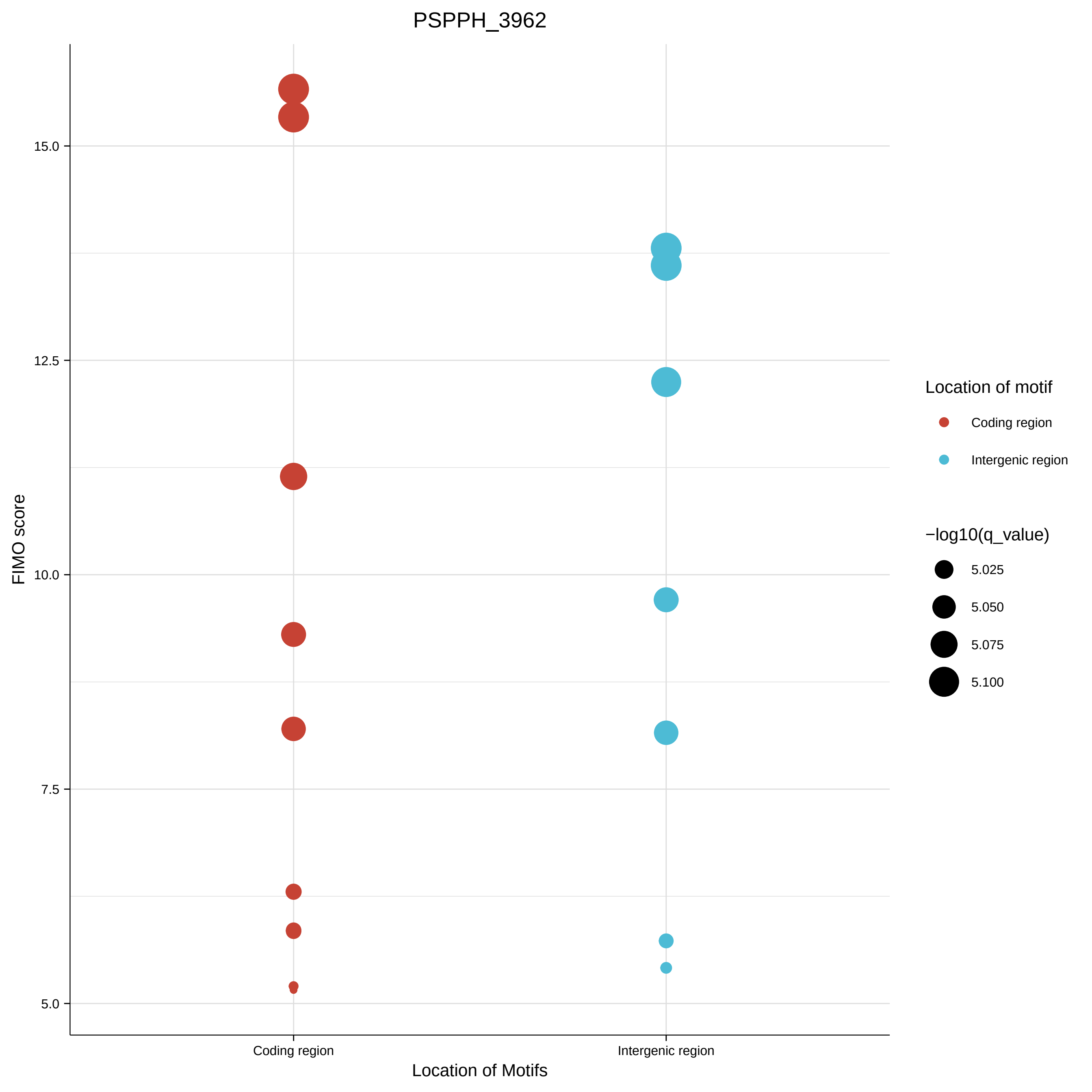

PSPPH_3962
FIMO score
15.0
12.5
10.0
7.5
5.0
Coding region
Intergenic region
Location of Motifs
Location of motif
Coding region
Intergenic region
−log10(q_value)
5.025
5.050
5.075
5.100

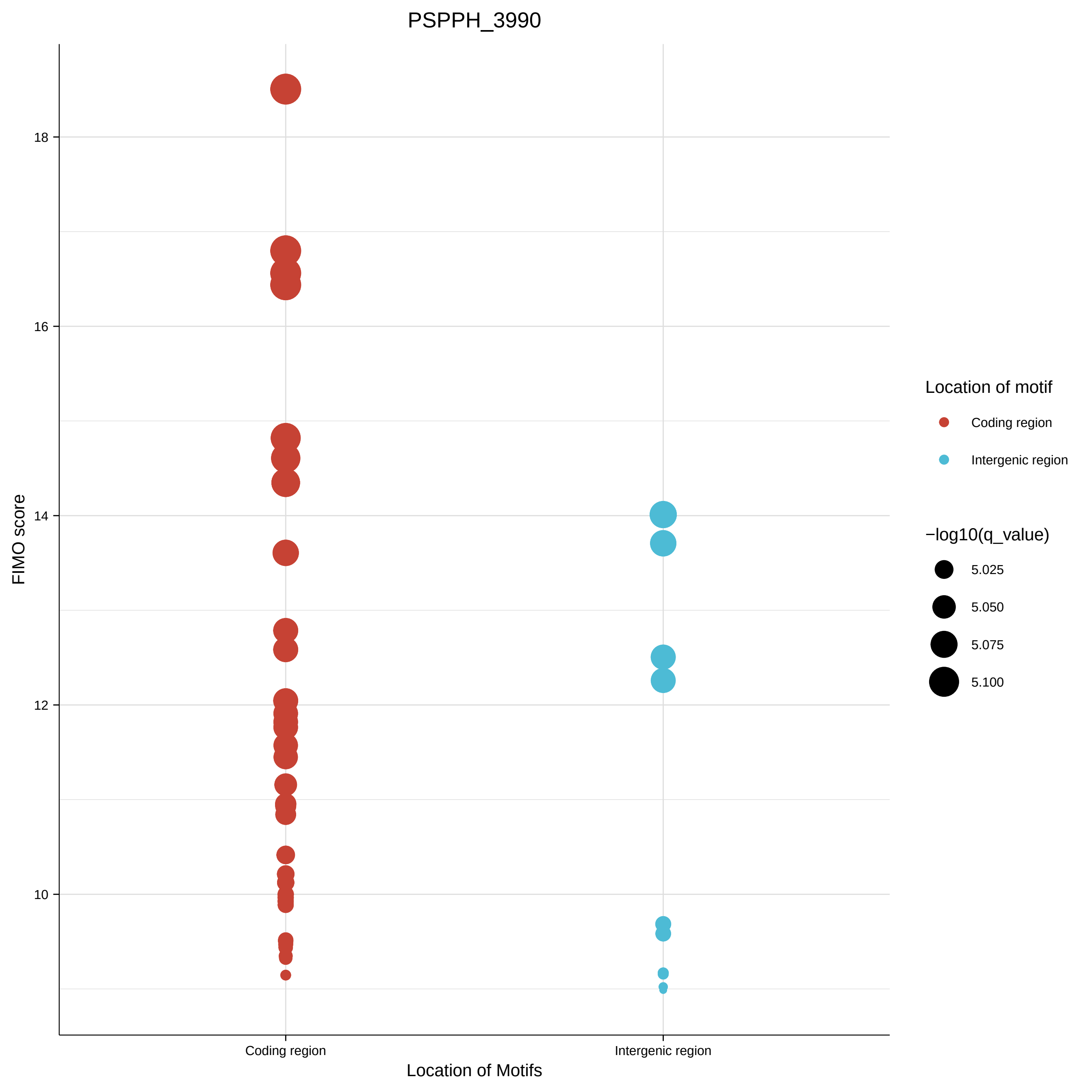
PSPPH_3990
FIMO score
18
16
14
12
10
Coding region
Intergenic region
Location of Motifs
Location of motif
Coding region
Intergenic region
−log10(q_value)
5.025
5.050
5.075
5.100

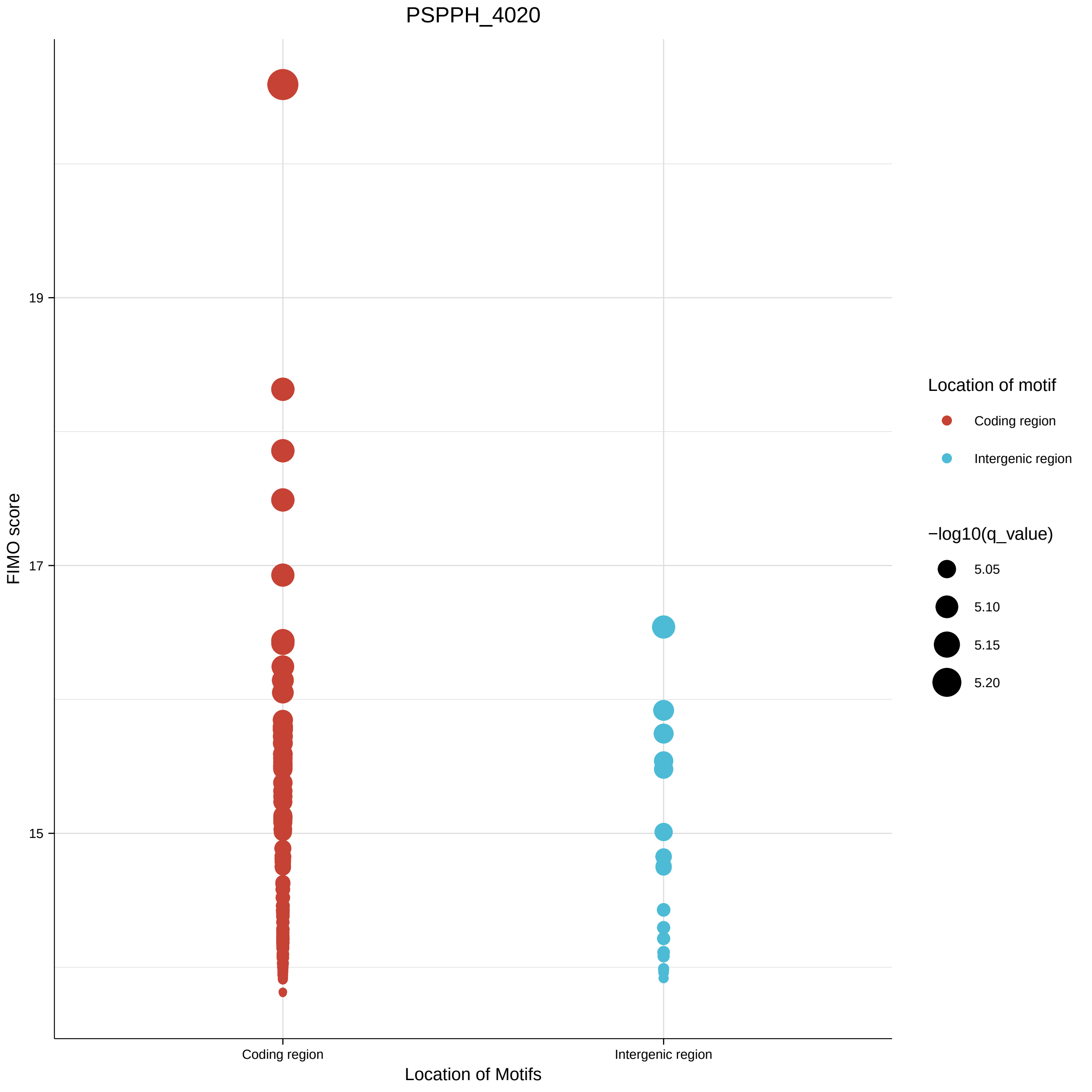
PSPPH_4020
FIMO score
19
17
15
Coding region
Intergenic region
Location of Motifs
Location of motif
Coding region
Intergenic region
−log10(q_value)
5.05
5.10
5.15
5.20

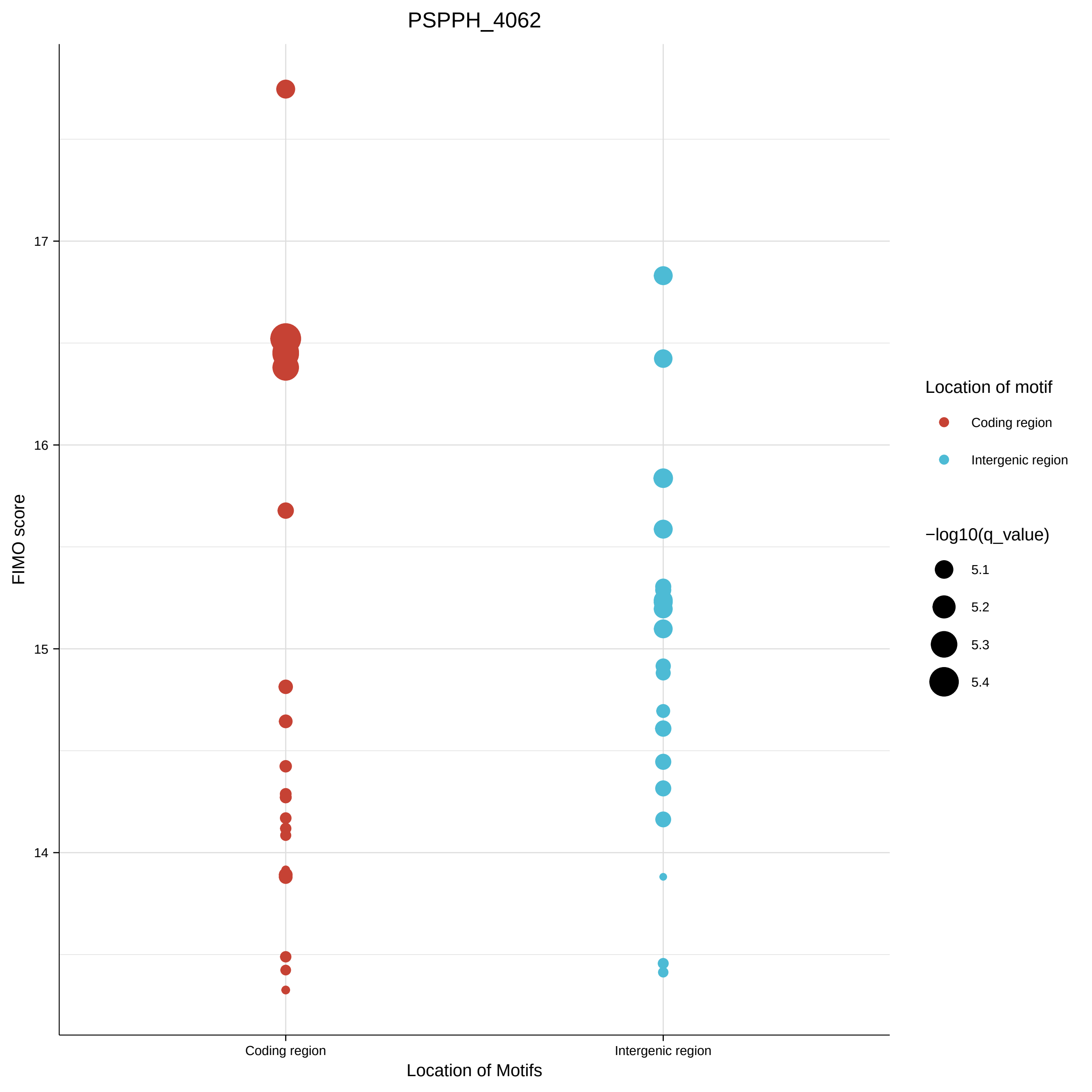

PSPPH_4062
FIMO score
17
16
15
14
Coding region
Intergenic region
Location of Motifs
Location of motif
Coding region
Intergenic region
−log10(q_value)
5.1
5.2
5.3
5.4

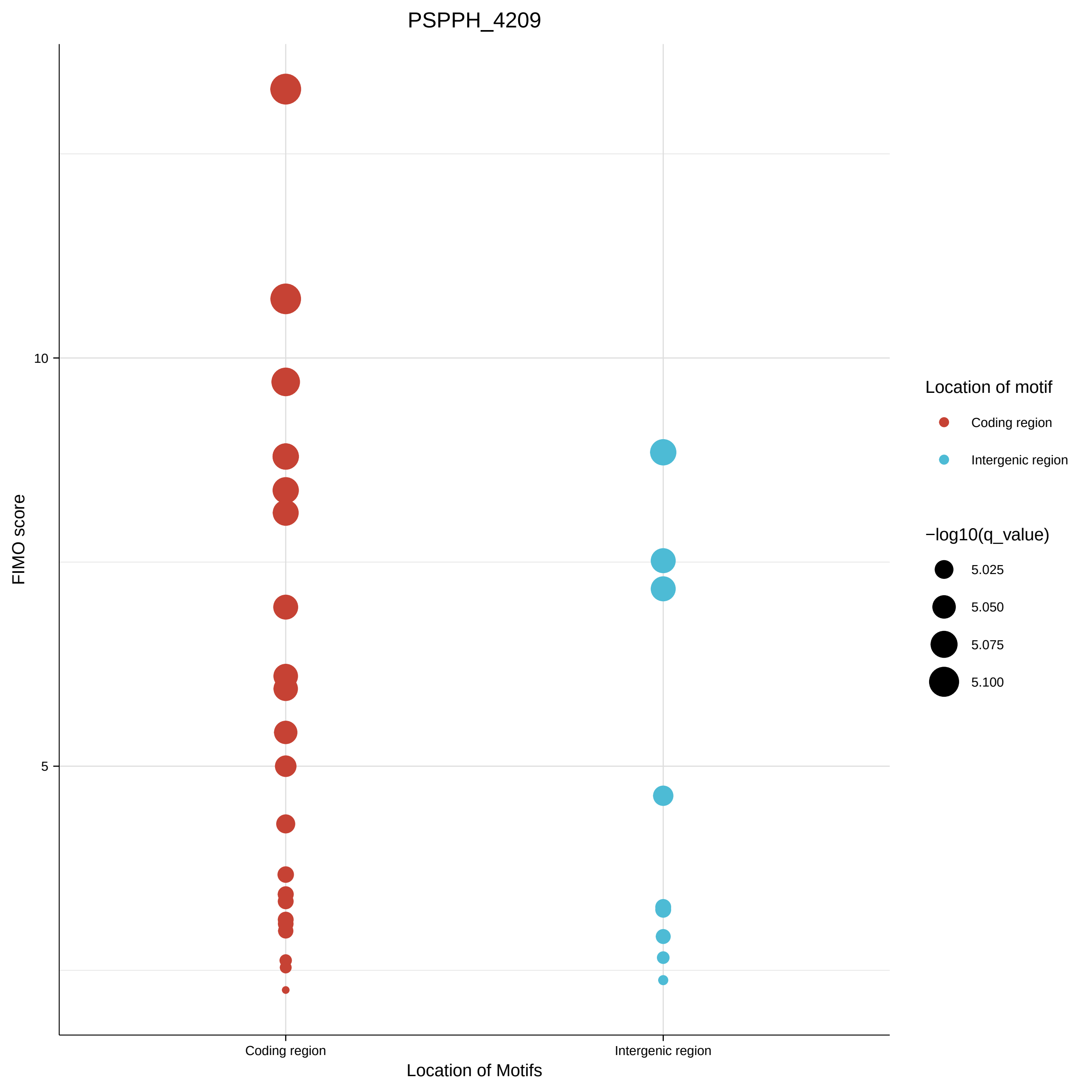
PSPPH_4209
FIMO score
10
5
Coding region
Intergenic region
Location of Motifs
Location of motif
Coding region
Intergenic region
−log10(q_value)
5.025
5.050
5.075
5.100

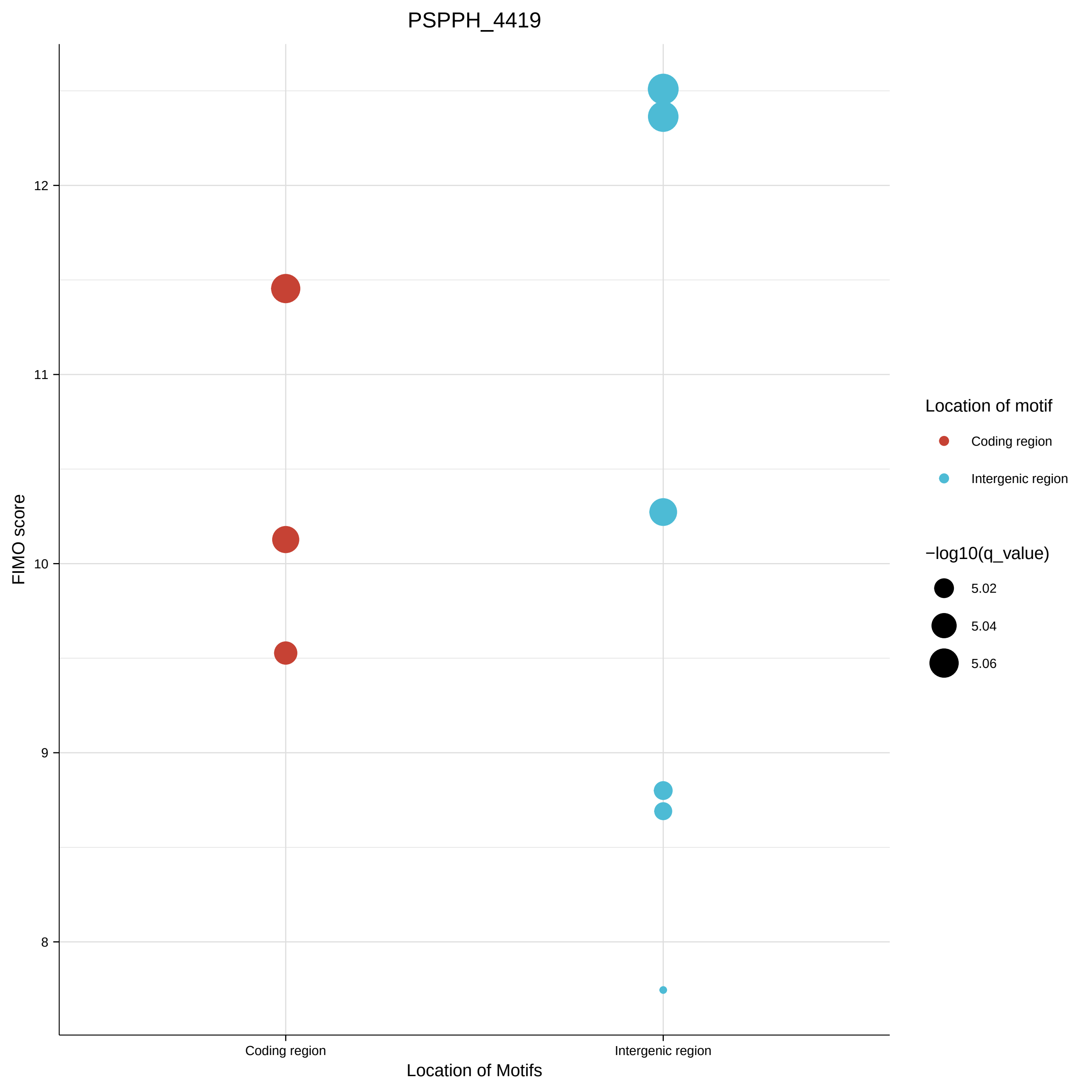

PSPPH_4419
FIMO score
12
11
10
9
8
Coding region
Intergenic region
Location of Motifs
Location of motif
Coding region
Intergenic region
−log10(q_value)
5.02
5.04
5.06

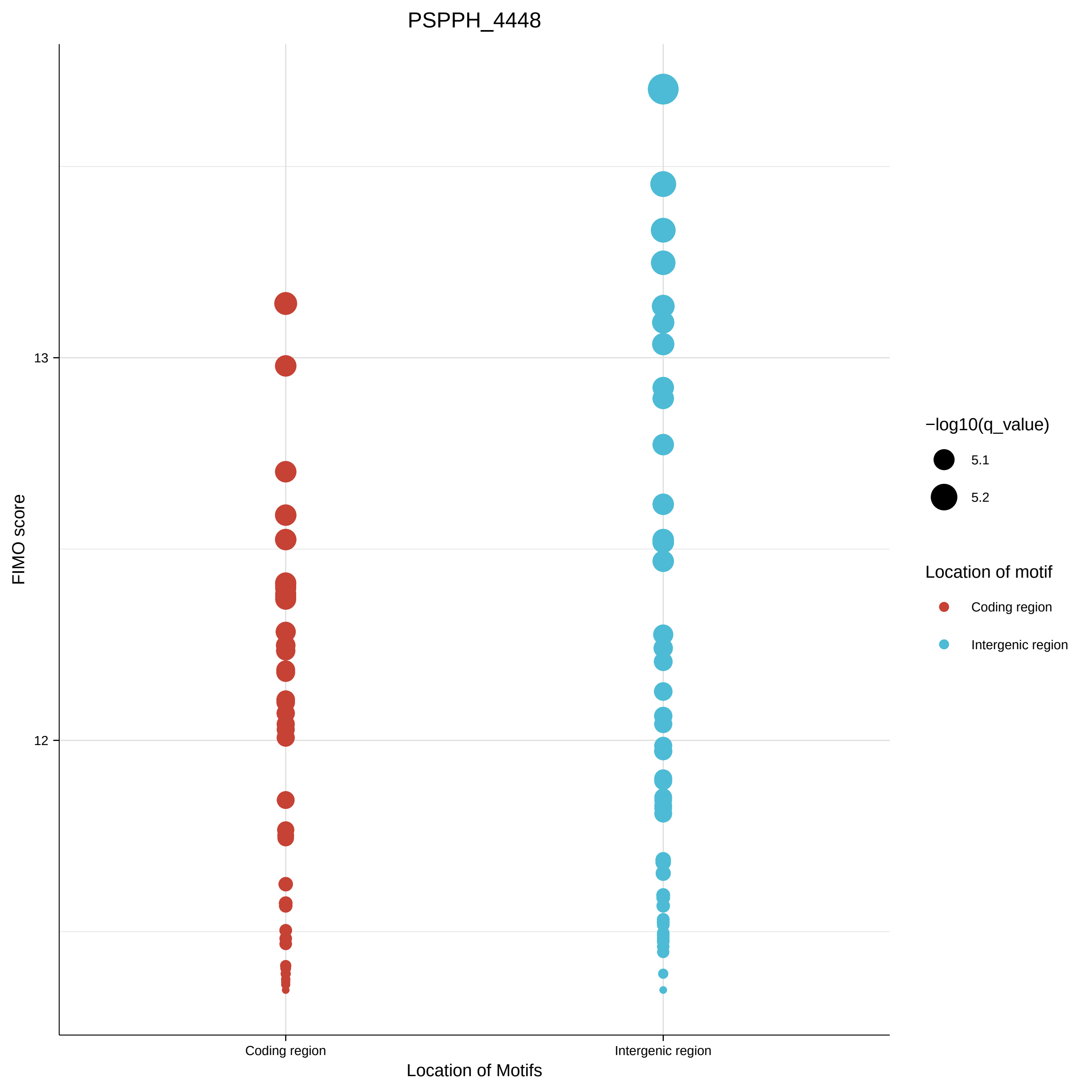

PSPPH_4448
FIMO score
13
12
Coding region
Intergenic region
Location of Motifs
−log10(q_value)
5.1
5.2
Location of motif
Coding region
Intergenic region

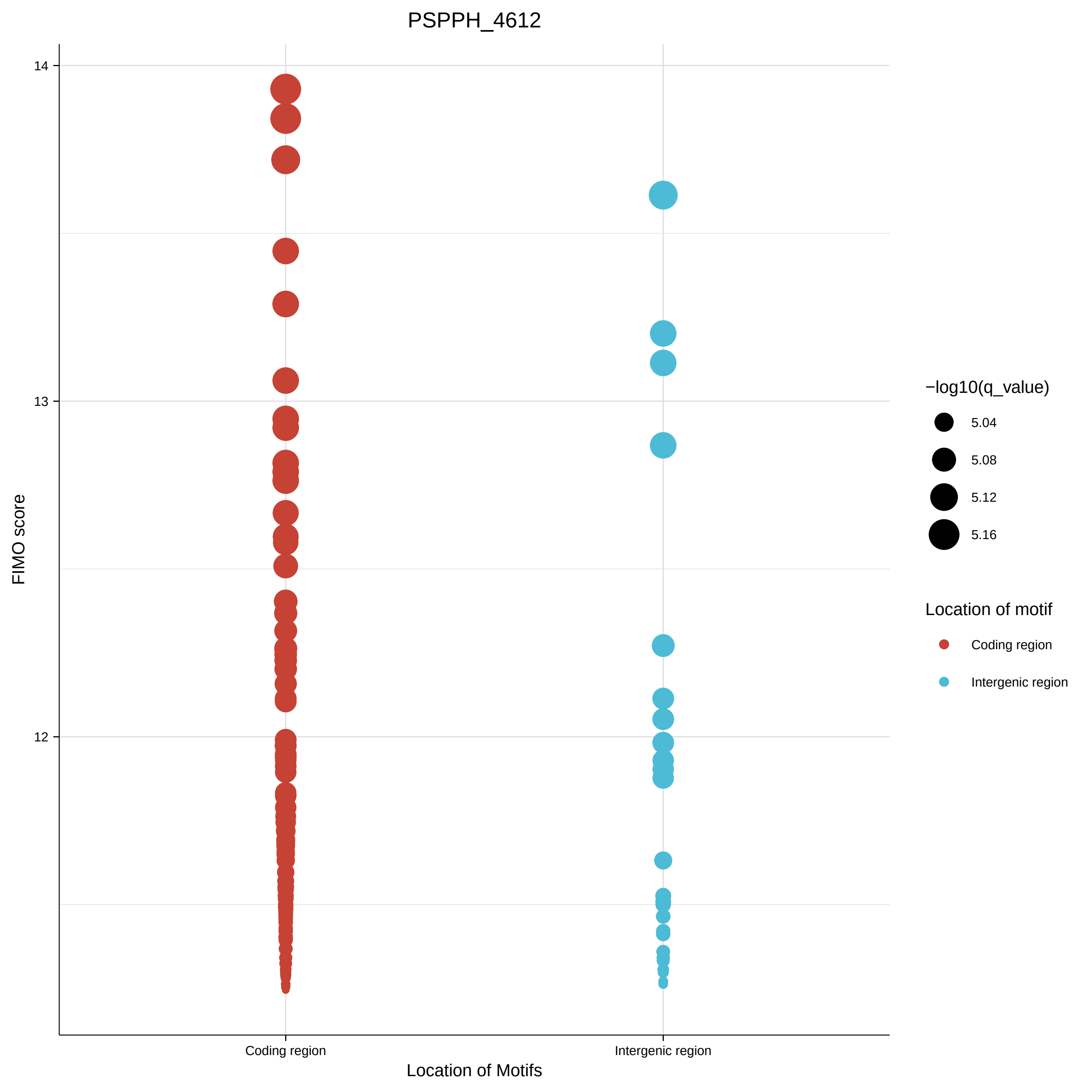
PSPPH_4612
FIMO score
14
13
12
Coding region
Intergenic region
Location of Motifs
−log10(q_value)
5.04
5.08
5.12
5.16
Location of motif
Coding region
Intergenic region

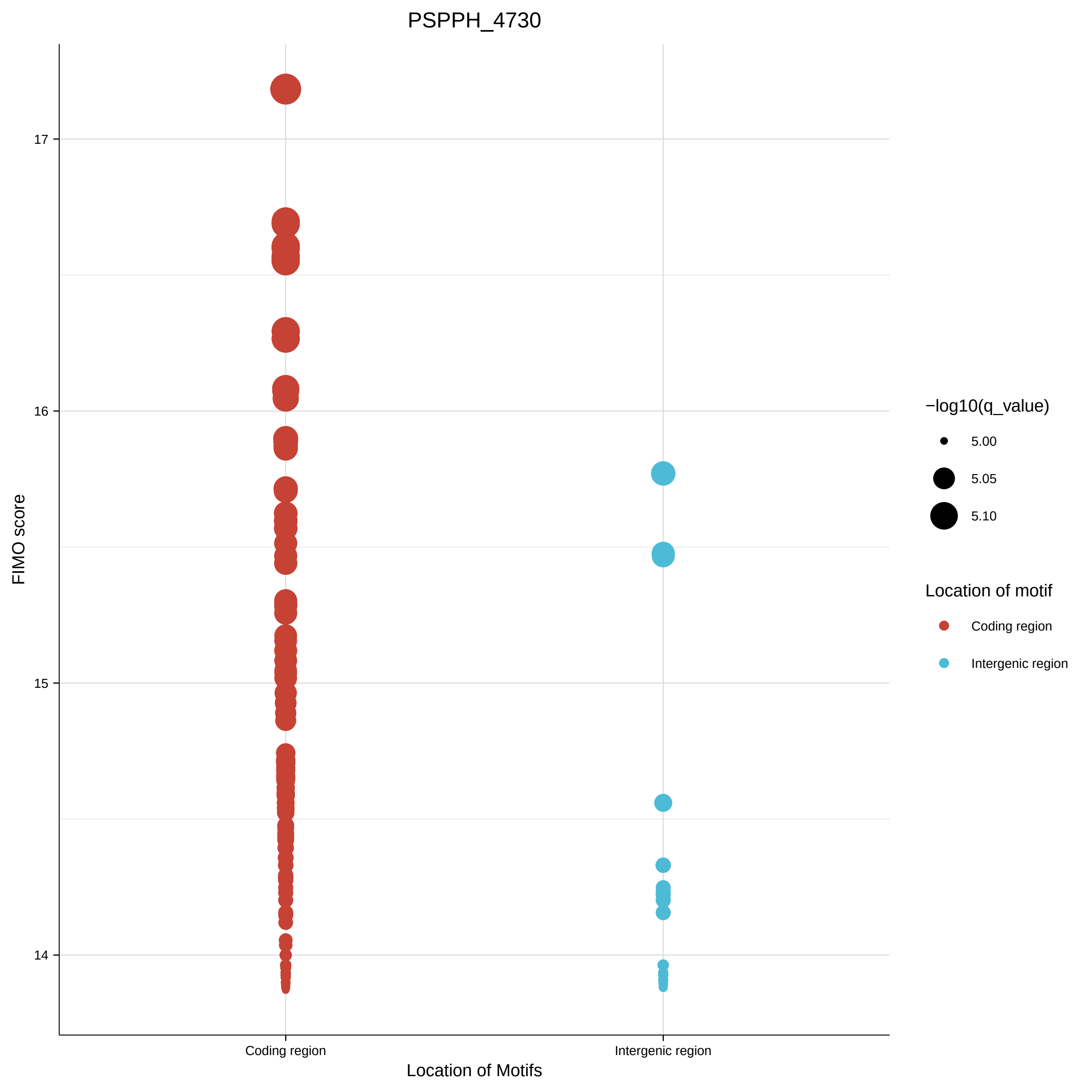
PSPPH_4730
FIMO score
17
16
15
14
Coding region
Intergenic region
Location of Motifs
−log10(q_value)
5.00
5.05
5.10
Location of motif
Coding region
Intergenic region

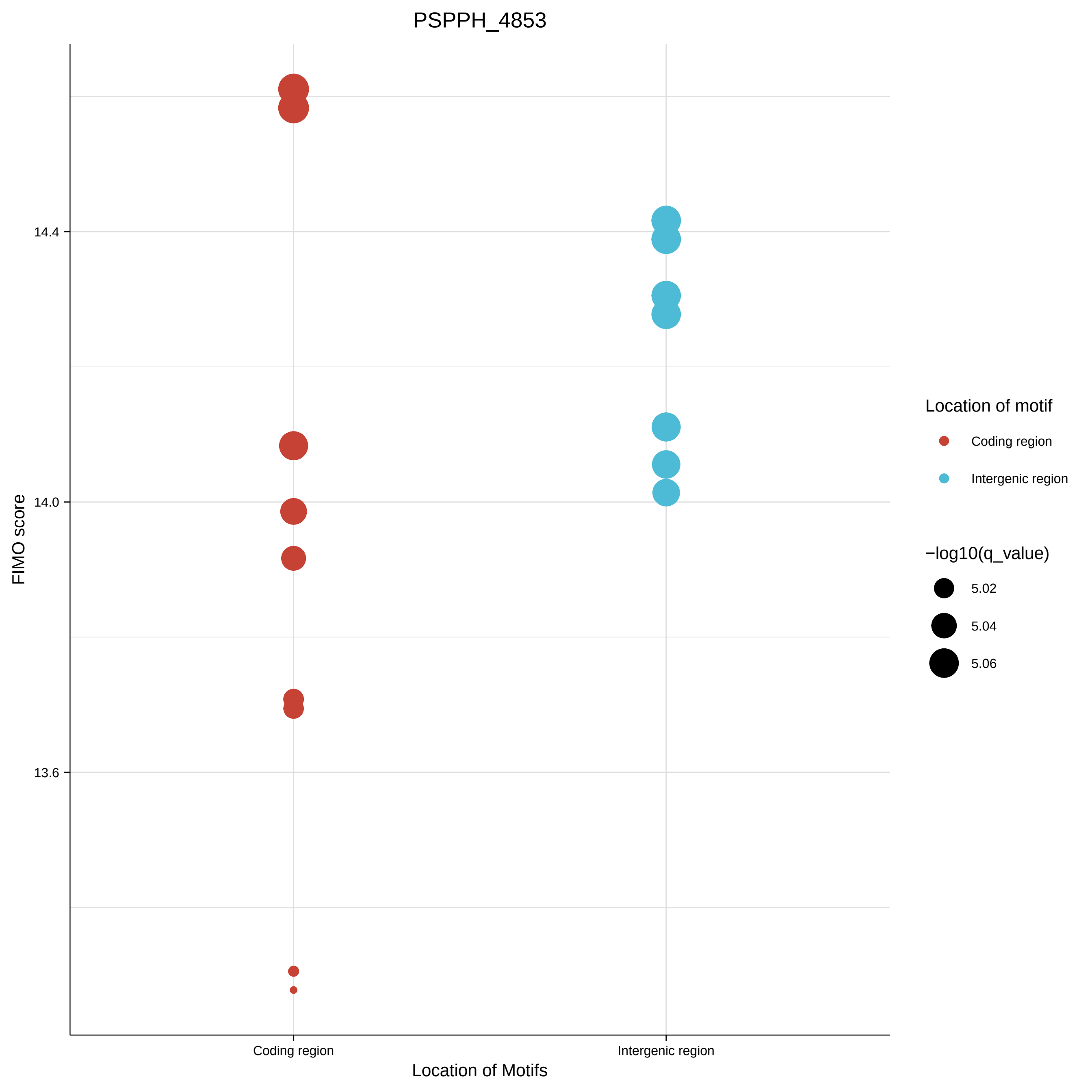
PSPPH_4853
FIMO score
14.4
14.0
13.6
Coding region
Intergenic region
Location of Motifs
Location of motif
Coding region
Intergenic region
−log10(q_value)
5.02
5.04
5.06

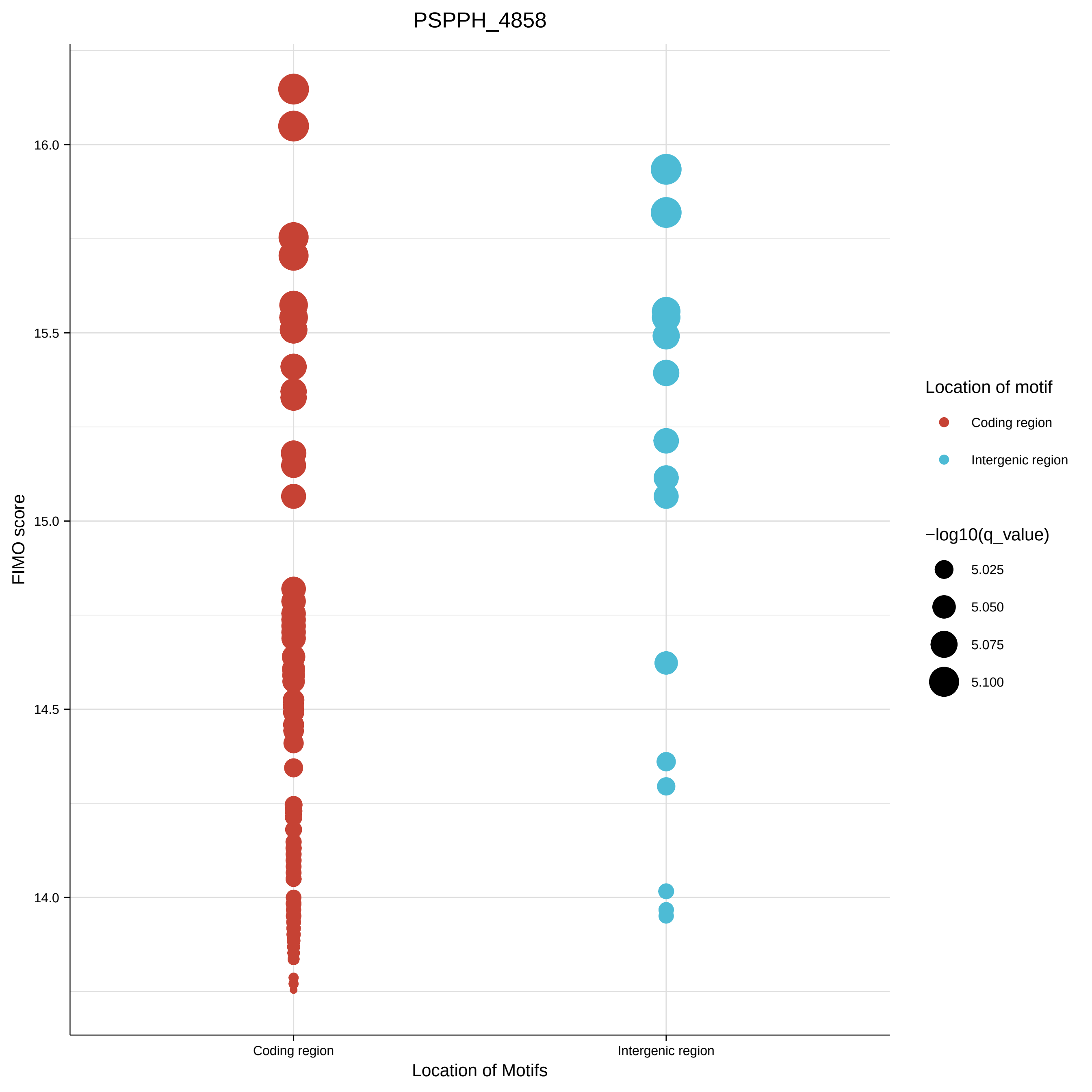

PSPPH_4858
FIMO score
16.0
15.5
15.0
14.5
14.0
Coding region
Intergenic region
Location of Motifs
Location of motif
Coding region
Intergenic region
−log10(q_value)
5.025
5.050
5.075
5.100

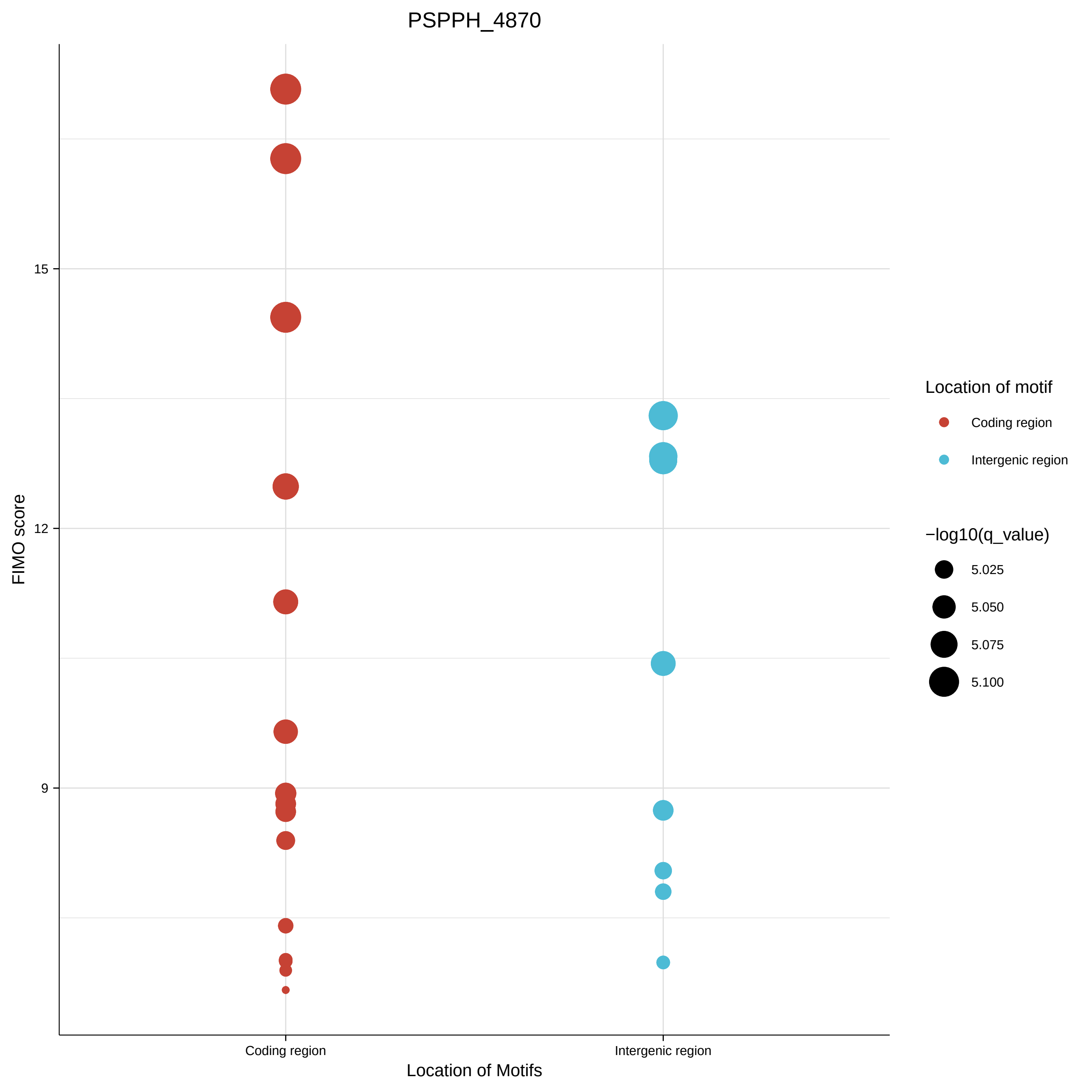

PSPPH_4870
FIMO score
15
12
9
Coding region
Intergenic region
Location of Motifs
Location of motif
Coding region
Intergenic region
−log10(q_value)
5.025
5.050
5.075
5.100

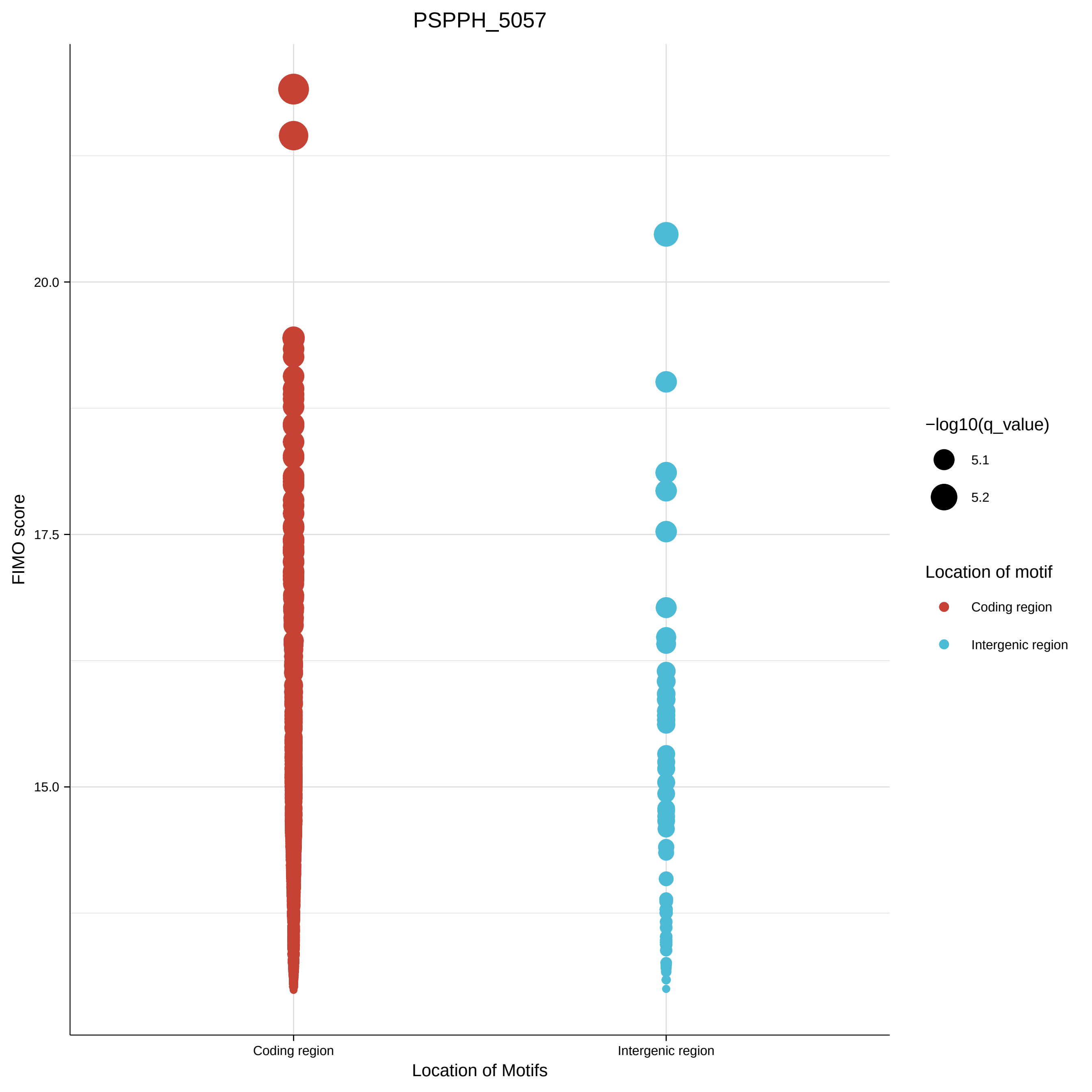

PSPPH_5057
20.0
17.5
15.0
FIMO score
Coding region
Intergenic region
Location of Motifs
−log10(q_value)
5.1
5.2
Location of motif
Coding region
Intergenic region

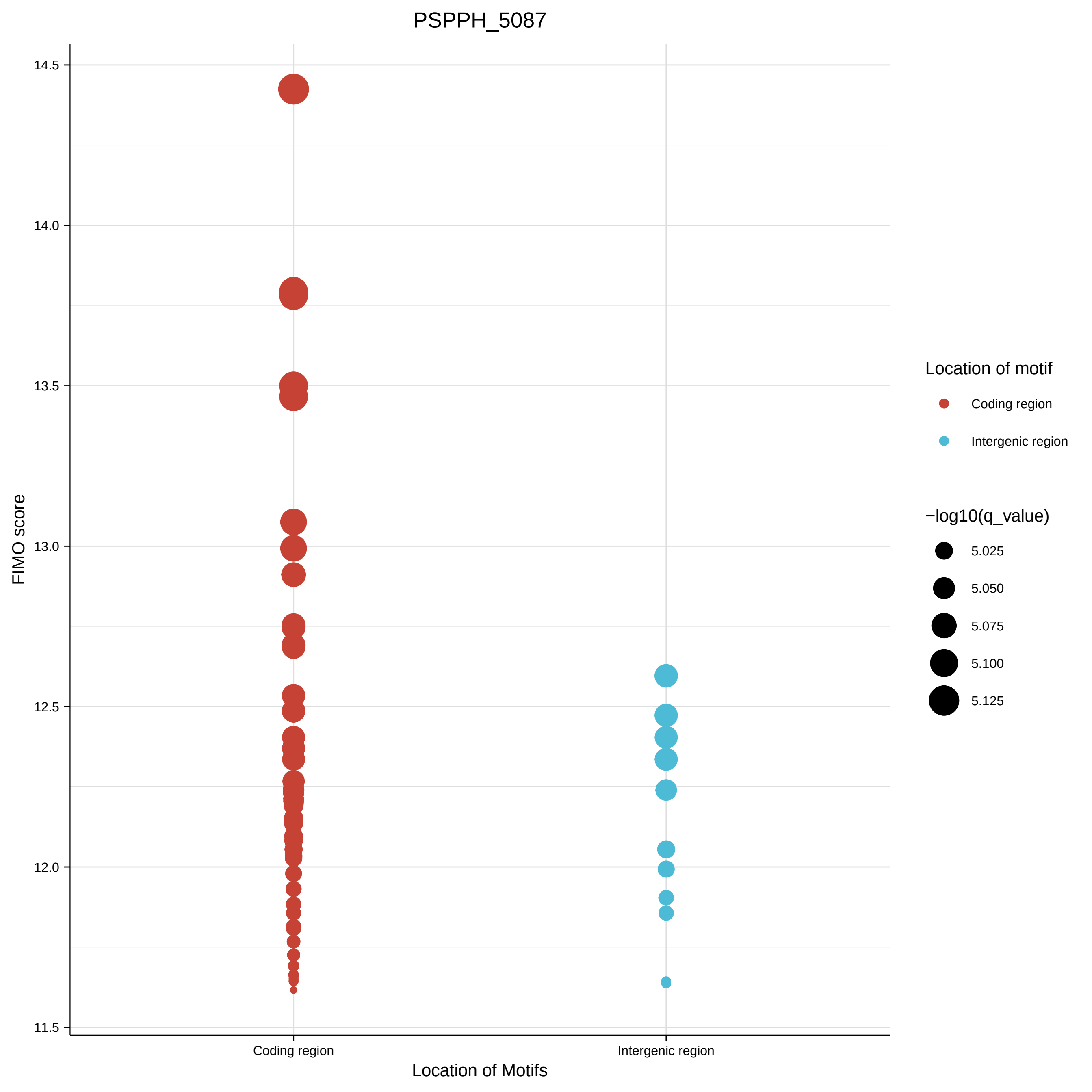
PSPPH_5087
FIMO score
14.5
14.0
13.5
13.0
12.5
12.0
11.5
Coding region
Intergenic region
Location of Motifs
Location of motif
Coding region
Intergenic region
−log10(q_value)
5.025
5.050
5.075
5.100
5.125

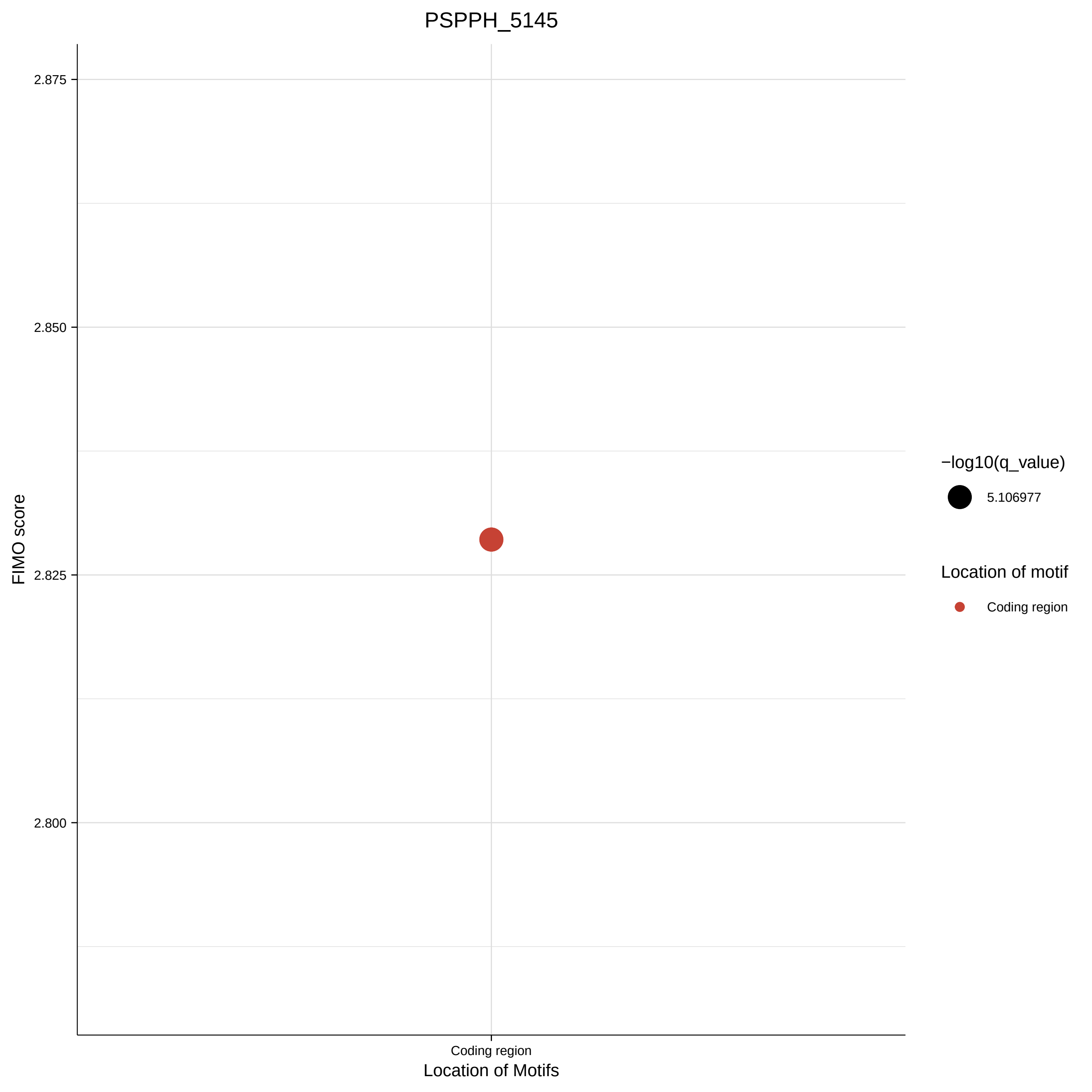

PSPPH_5145
FIMO score
2.875
2.850
2.825
2.800
Coding region
Location of Motifs
−log10(q_value)
5.106977
Location of motif
Coding region

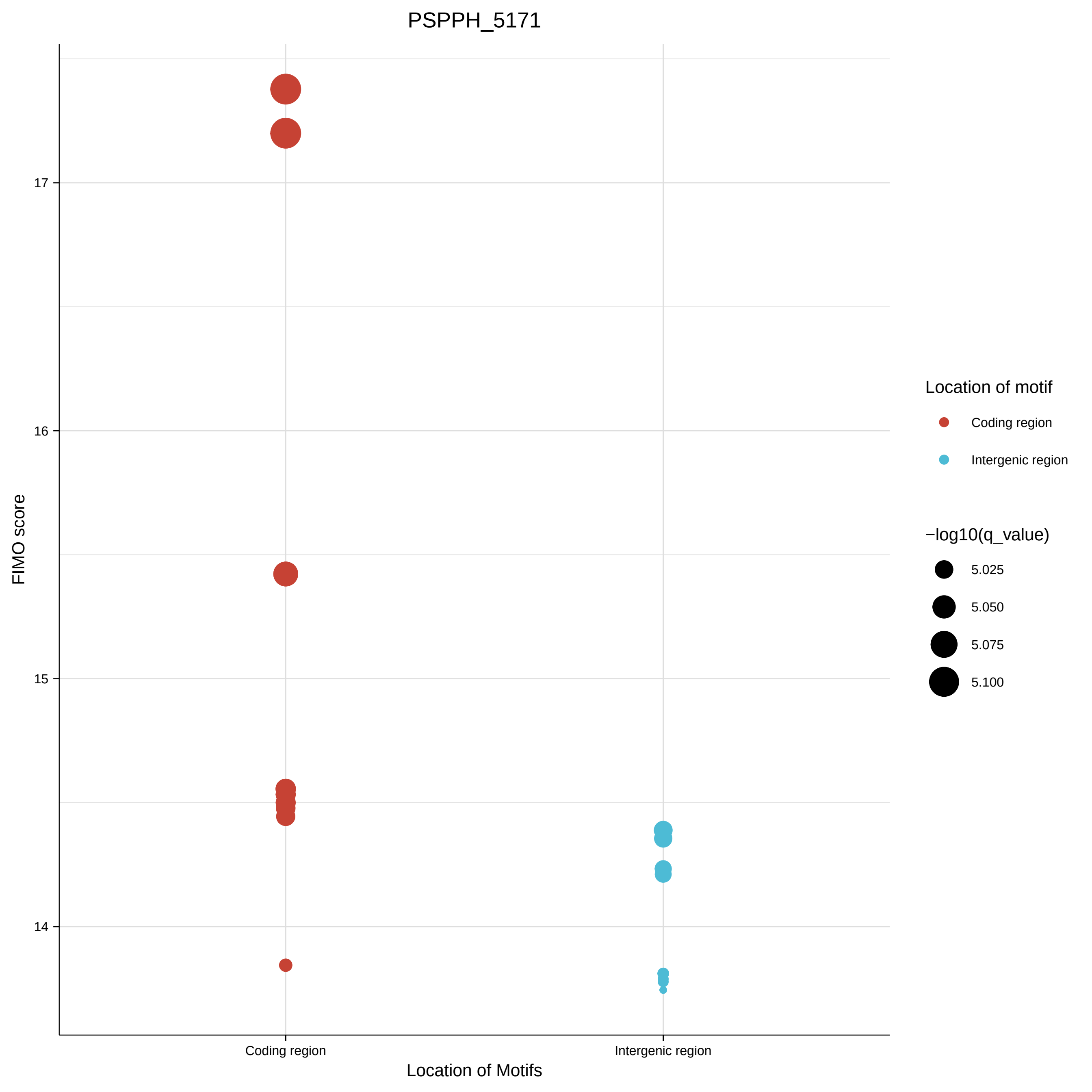

PSPPH_5171
FIMO score
17
16
15
14
Coding region
Intergenic region
Location of Motifs
Location of motif
Coding region
Intergenic region
−log10(q_value)
5.025
5.050
5.075
5.100

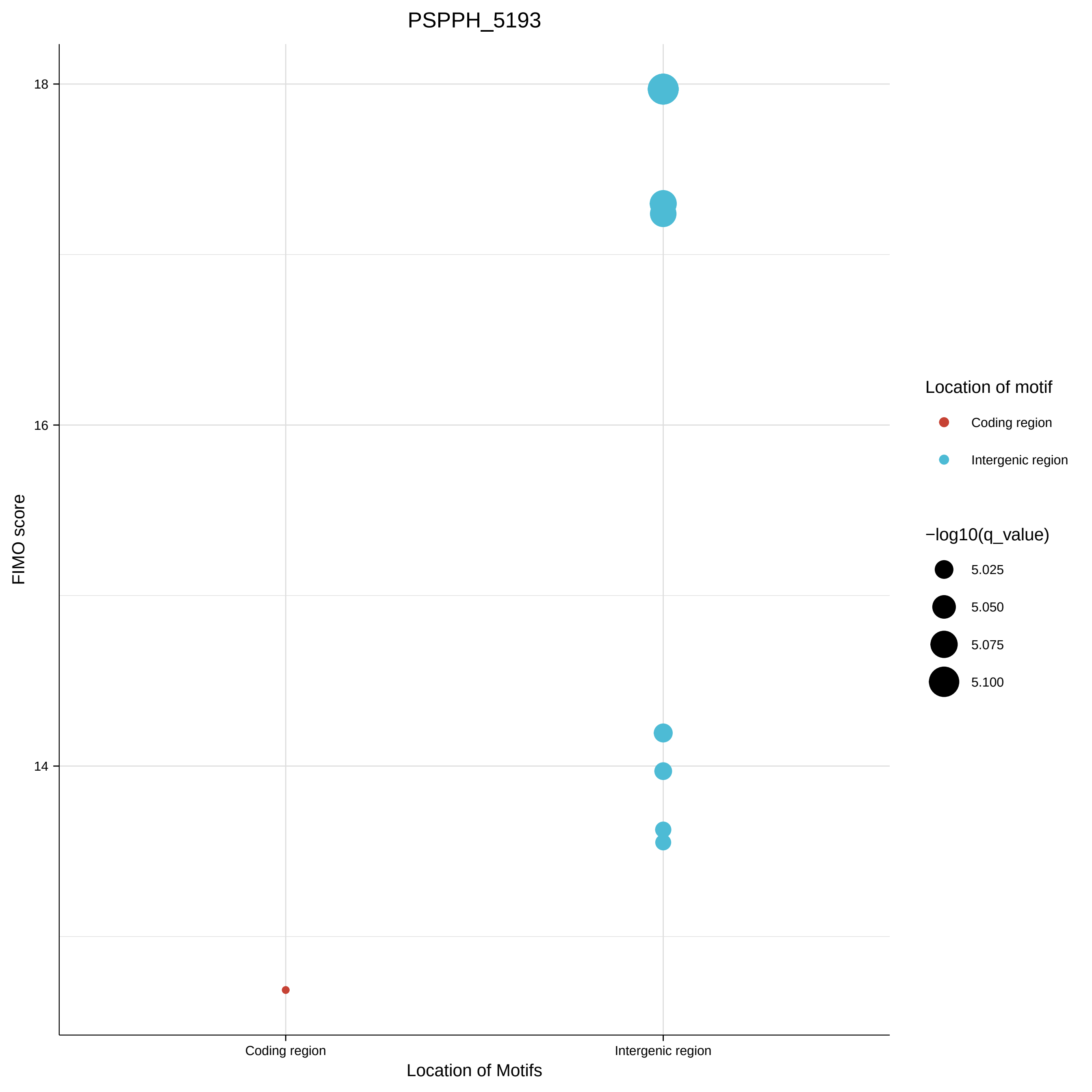

PSPPH_5193
FIMO score
18
16
14
Coding region
Intergenic region
Location of Motifs
Location of motif
Coding region
Intergenic region
−log10(q_value)
5.025
5.050
5.075
5.100

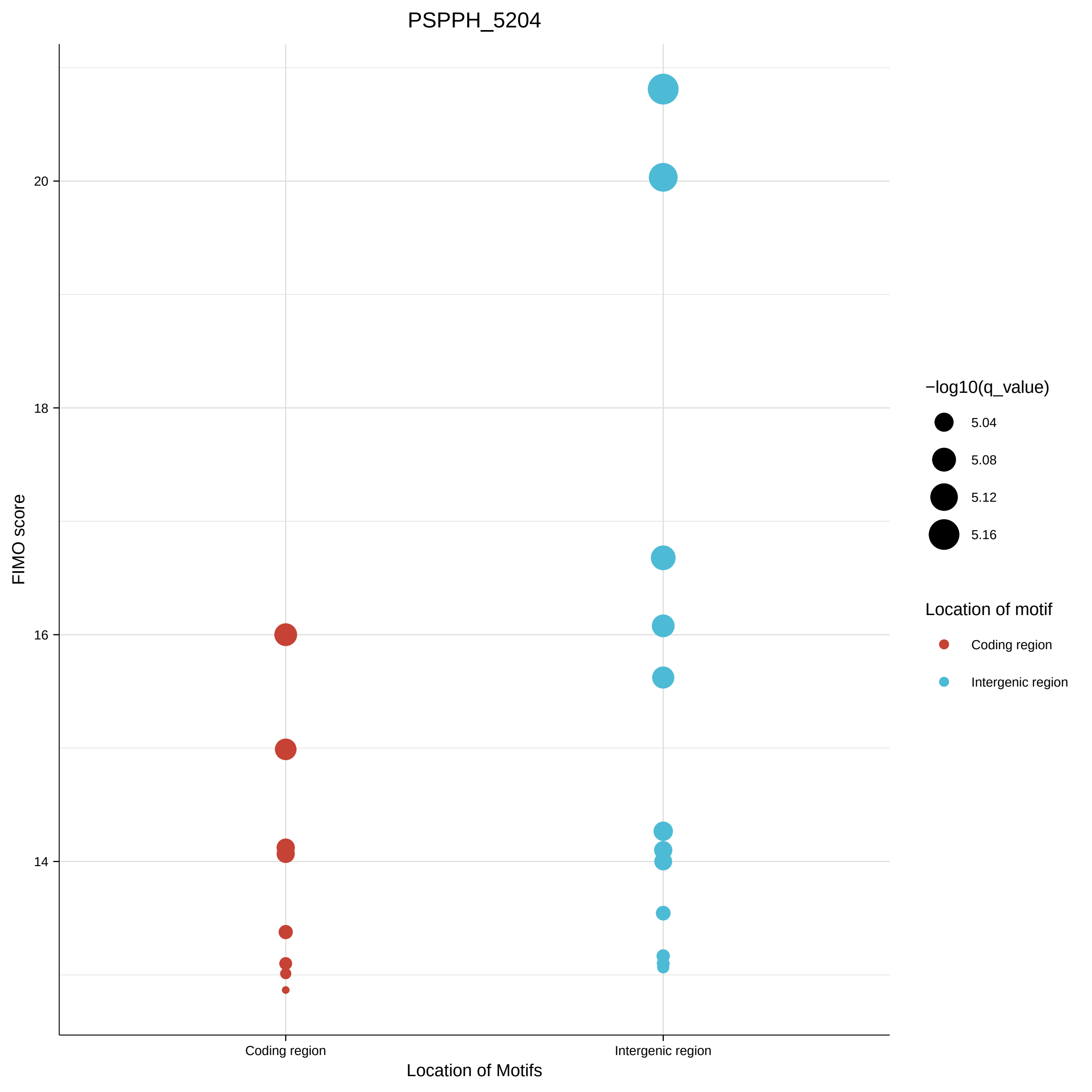

PSPPH_5204
FIMO score
20
18
16
14
Coding region
Intergenic region
Location of Motifs
−log10(q_value)
5.04
5.08
5.12
5.16
Location of motif
Coding region
Intergenic region

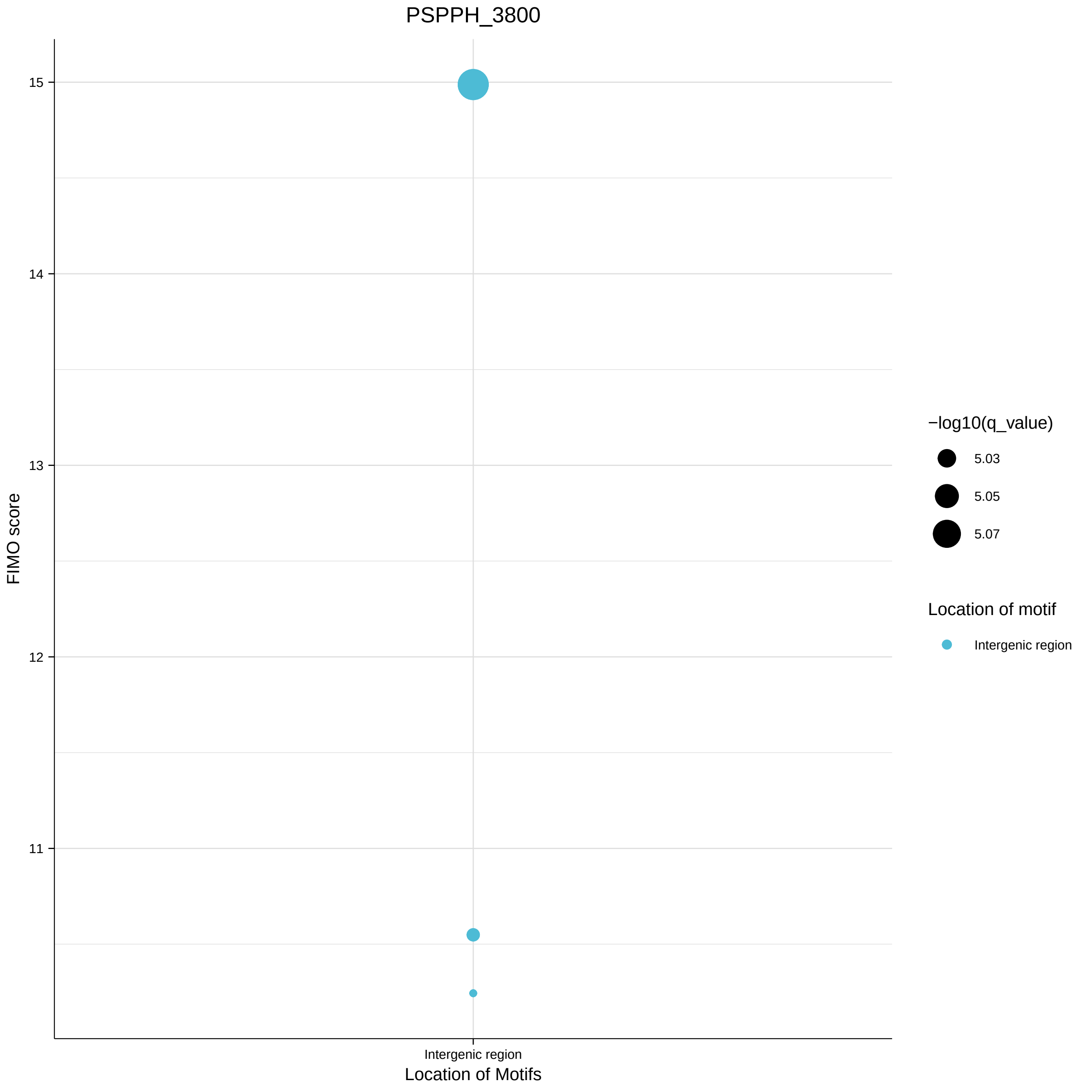
PSPPH_3800
FIMO score
15
14
13
12
11
Intergenic region
Location of Motifs
−log10(q_value)
5.03
5.05
5.07
Location of motif
Intergenic region

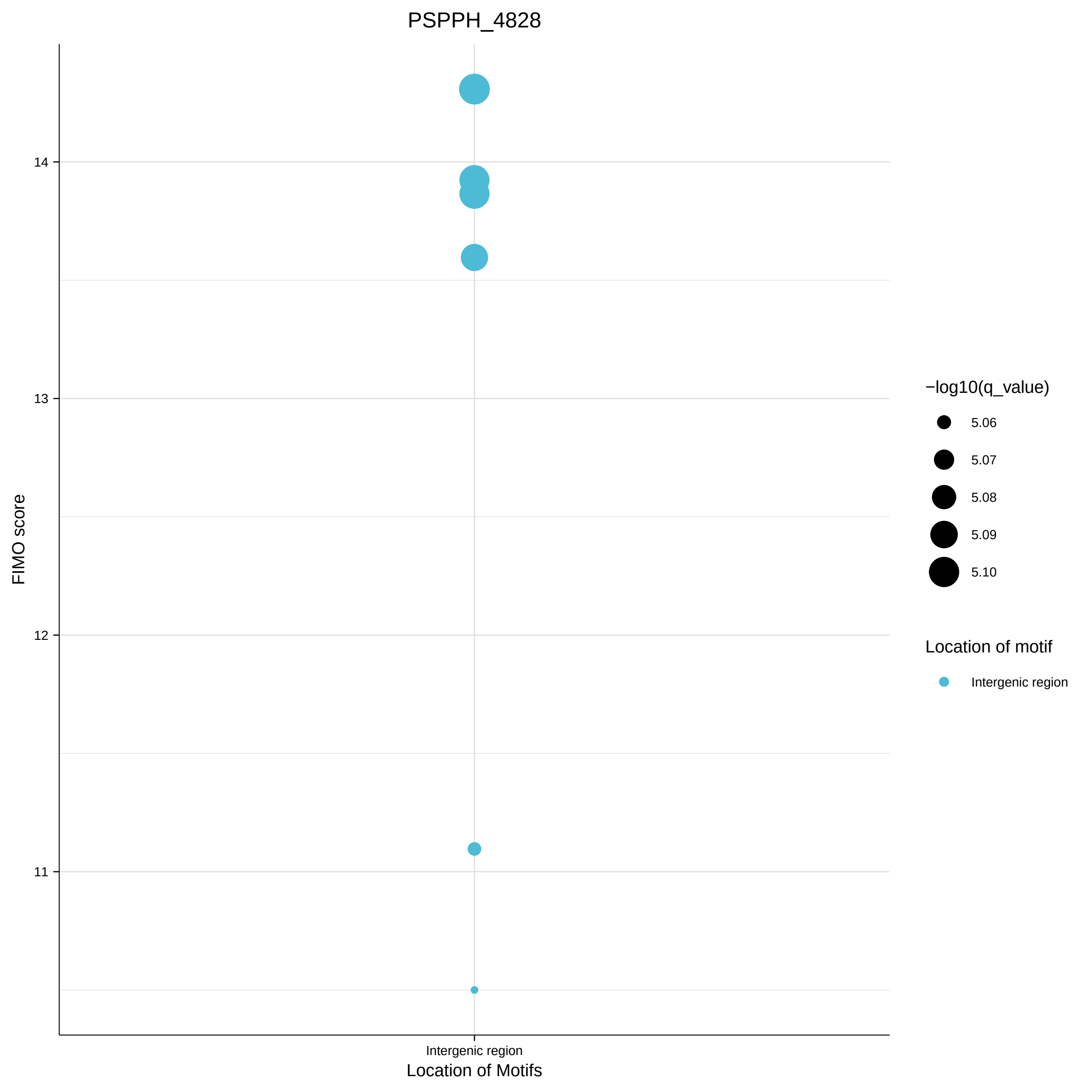
PSPPH_4828
FIMO score
14
13
12
11
Intergenic region
Location of Motifs
−log10(q_value)
5.06
5.07
5.08
5.09
5.10
Location of motif
Intergenic region
