# Supplementary material for: Bacterial Transcription Factors Bind to Coding Regions and Regulate Internal Cryptic Promoters
Source: mBio. 2022 Oct 6;13(5):e01643-22. doi: 10.1128/mbio.01643-22 (PMC9600179; doi:10.1128/mbio.01643-22)
Supplement: TABLE S1 [file mbio.01643-22-s0006.docx]

| Supplementary Table 1. Strains, plasmids and primers used in this study | | |
| --- | --- | --- |
| Strains | Description | Reference or Application |
| *E. coli* Strains |  |  |
| *E. coli* DH5α | supE44 ΔlacU169(φ80lacZΔM15) hsdR17recA1 endA1 gyrA96thi-1 relA1λpir | Stratagene |
| *E. coli* BL21(DE3) | F^-^ ompT hsdS_B_ (r_B_^-^m_B_^-^) gal dcm met (DE3) | Invitrogen |
| *P. syringae* strains |  |  |
| *P. syringae* 1448A | Wild type | Lab stock |
| *P. syringae* 1448A Δ*rhpS* | Clean deletion of *rhpS* | (Xie et al., 2019) |
| *P. aeruginosa* strains |  |  |
| *P. aeruginosa* PAO1 | Wild type | Lab stock |
| *P. aeruginosa* PAO1 ∆*algR* | deletion of *algR* | (Huang et al., 2019) |
| *P. aeruginosa* PAO1 ∆*vqsM* | deletion of *vqsM* | (Huang et al., 2019) |
|  |  |  |
| Primers | Sequences |  |
| RT-qPCR Primer |  |  |
| RhpR_4417-F | GCTGGGTTTCAACGTCAGTT | |
| RhpR_4417-R | TCGCTTGTGCAGTAAGGTTG | |
|  |  |  |
| RhpR_0738-F | TGGTTCGCTTACTCATGTGG | |
| RhpR_0738-R | GCTGCAATACCATTGCTGTG | |
|  |  |  |
| RhpR_3403-F | CTGTCGGAGGAGGAAGACTG | |
| RhpR_3403-R | GGTTGATACGTTTGCCAGGT | |
|  |  |  |
| RhpR_3404-F | GTGAGTGACGGTGGGTCTCT | |
| RhpR_3404-R | ACGTGTCAATCTGCAAGTCG | |
|  |  |  |
| RhpR_PilR_F | CGCTCTGTGAAAACGATGAA | |
| RhpR_PilR_R | GCTCGATGCTTTCCAGGTAG | |
|  |  |  |
| RhpR_flgF_F | GTCGACCGTATCAAGCTGGT | |
| RhpR_flgF_R | GTTACTCGCCTGCAAAAAGC | |
|  |  |  |
| RhpR_PilS_F | GGTTGCTGGCTGTATCTGGT | |
| RhpR_PilS_R | ATGATTGCGTCAGTCATTGC | |
|  |  |  |
| RhpR_4418_F | GTTTGTAATTTGCGCCGAAC | |
| RhpR_4418_R | AATGTCAAGCCCAGTTCCAG | |
|  |  |  |
| AlgR_PA2933_F | TCACCCCCTACACCTACCTG | |
| AlgR_PA2933_R | CCATCAGCAGGACGAACAG | |
|  |  |  |
| AlgR_morB_F | ACCTGATCGACCAGTTCCTC | |
| AlgR_morB_R | GTGCATGTCGTTGATCGTG | |
|  |  |  |
| AlgR_PA2705_F | CCGGACTACAAGGTGGTGTT | |
| AlgR_PA2705_R | TCCAGGTGTCCTTCGGATAG | |
|  |  |  |
| AlgR_PA2706_F | GCGCGGTAAGCTATGAAATC | |
| AlgR_PA2706_R | GGCAGAAGGTACGGGTGAC | |
|  |  |  |
| AlgR_PA4501_F | CGAGACCGACTGGAGCTACT | |
| AlgR_PA4501_R | CCCAGTTCGTGGTGTTTCTC | |
|  |  |  |
| AlgR_PA4502_F | CATCAACACCCAACACAAGC | |
| AlgR_PA4502_R | CAGTCCTGGATCGAATGGTT | |
|  |  |  |
| AlgR_PA4503_F | ACCTGGACTATGTCGGCAAC | |
| AlgR_PA4503_R | CGTGGTCAAAAATCGAACCT | |
|  |  |  |
| AlgR_pta_F | CTTCCACCTGGCAAAGAGTC | |
| AlgR_pta_R | CGGACCTTGTTGAGGATCAC | |
|  |  |  |
| VqsM_dadX_F | CGGTGATGACCCTAGAATCG | |
| VqsM_dadX_R | GTAAGGTCGACGGTGAGCAT | |
|  |  |  |
|  |  |  |
| AlgR_0930_F | CGTCATTCGCTGTTCTGGC | |
| AlgR_0930_R | CATCGTCGAGCCAGGCAC | |
|  |  |  |
| AlgR_3087_F | GATATCGTCTTCCAGCCCGA | |
| AlgR_3087_R | TACATCACCGCGCTGTAGTC | |
|  |  |  |
| RhpR_2788_F | AAAGGCGACCGTTCTTTCAC | |
| RhpR_2788_R | GGGGTTGATGTCGACGAATG | |
|  |  |  |
| AlgR_PA0323_F | TGGGCAATCTGAAGGAAAAC | |
| AlgR_PA0323_R | ATGGAGTTCAGAGCCACCAC | |
|  |  |  |
| AlgR_PA0325_F | GGCTGCATCCTGGTATTCAT | |
| AlgR_PA0325_R | AGCGCAGGTTGTAGATCAGC | |
|  |  |  |
| AlgR_PA0326_F | AACGAATTCTTCACCCTCCTC | |
| AlgR_PA0326_R | CAGCGCATAACTCTGGAACA | |
|  |  |  |
| AlgR_PA0324_F | CCTGAAGCTGTCGTTGTTCA | |
| AlgR_PA0324_F | GCAGGGTGATTTTCCAGAAG | |
| EMSA Primer |  |  |
| RhpR_EMSA_PSPPH_4418_F | TTGATGAAATTATTTCTTCTGCTTGTC | |
| RhpR_EMSA_PSPPH_4418_R | AATGTCAAGCCCAGTTCCAG | |
|  |  |  |
| RhpR_EMSA_pilS_F | CCTGCACCAGCATTGCG | |
| RhpR_EMSA_pilS_R | TTCCGTAGCAATCAGCAATGT | |
|  |  |  |
| AlgR_EMSA_pta | GACAGCACCGGCAGGCCG | |
| AlgR_EMSA_pta | GCCCGCGCCGTGCC | |
|  |  |  |
| AlgR_EMSA_morB | GAACGCTTCCGCGCG | |
| AlgR_EMSA_morB | TAATCGGTGTAGCCGGCAG | |
|  |  |  |
| VqsM_EMSA_dadX | GGCGGATACCGGCCTCGC | |
| VqsM_EMSA_dadX | TGCGCCCCGCCCGTG | |
|  |  |  |
| AlgR_EMSA_PA0324 | AGGATGGTGAACAACGACAGC | |
| AlgR_EMSA_PA0324 | AACTCGCTGCTCGTCGC | |
|  |  |  |
| AlgR_EMSA_PA0325 | TAGATCAGCATCGCCAGCAG | |
| AlgR_EMSA_PA0325 | CCGCTGGCGATGCCG | |
|  |  |  |
| AlgR_EMSA_PA2706 | AGGGTGCCGAGGGCGAT | |
| AlgR_EMSA_PA2706 | AGCCAGTGCCGCAAGGC | |
|  |  |  |
| AlgR_EMSA_dppA4 | ATGGCGATGCCCTTCGC | |
| AlgR_EMSA_dppA4 | AGGGTAATGGCGAACACCAG | |
|  |  |  |
| RhpR_EMSA_pilS_F | CCTGCACCAGCATTGCGG | |
| RhpR_EMSA_pilS_R | TTCCGTAGCAATCAGCAATGTATTGC | |
|  |  |  |
| RhpR_EMSA_3403_F | TCCTCCTCCGACAGACGC | |
| RhpR_EMSA_3403_R | CCGAAGGGCATCATCGAATG | |
|  |  |  |
| PSPPH_2787_EMSA_F | CATATCGCCGCCGAGCAA | |
| PSPPH_2787_EMSA_R | CGTGGTTGCTGACAGCAG | |
|  |  |  |
| AlgR_EMSA_PA0930 | TGAAGGGTTACTACCTGTCCG | |
| AlgR_EMSA_PA0930 | CCACCCGGCCTTGCTC | |
|  |  |  |
| PA3086_EMSA_F | GATATCGTCTTCCAGCCCGA | |
| PA3086_EMSA_R | TACATCACCGCGCTGTAGTC | |
|  |  |  |
| PSPPH_0405_EMSA_F | GACATGCTGCCCAAGATCAG | |
| PSPPH_0405_EMSA_R | TCATCCATGTGATTGCTGCG | |
|  |  |  |
| VqsM_dadX_EMSA_F | TCCAGGCCCTCCTCGAT | |
| VqsM_dadX_EMSA_R | AGAGACCCGTCGCCATG | |
|  |  |  |
| AlgR_tse5_EMSA_F | CGGGTCTTCGTAGGCGT | |
| AlgR_tse5_EMSA_R | GCTCGAGGAGGAGTCGC | |
|  |  |  |
| AlgR_pqsA_EMSA_F | AAGTCCCGCGAGCAGG |  |
| AlgR_pqsA_EMSA_R | CTGGCGATGGAAGGCAC | |
|  |  | |
| AlgR_rocsS2_EMSA_F | GTACCTCGTCCGGCTCG |  |
| AlgR_rocsS2_EMSA_R | TGGCGCTGGAGCTCTG |  |
|  |  | |
|  |  | |

**Reference**

Huang, H., Shao, X., Xie, Y., Wang, T., Zhang, Y., Wang, X., and Deng, X. (2019). An integrated genomic regulatory network of virulence-related transcriptional factors in *Pseudomonas aeruginosa*. Nat Commun *10*, 2931.

Xie, Y., Shao, X., Zhang, Y., Liu, J., Wang, T., Zhang, W., Hua, C., and Deng, X. (2019). *Pseudomonas savastanoi* Two-Component System RhpRS Switches between Virulence and Metabolism by Tuning Phosphorylation State and Sensing Nutritional Conditions. mBio *10*.
